# Global landscape of COVID-19 and epilepsy research: A bibliometric analysis

**DOI:** 10.3389/fneur.2022.1034070

**Published:** 2022-10-24

**Authors:** Guangxin Wang, Lian Bai, Mingxue Zhao, Shumei Wang

**Affiliations:** ^1^Shandong Innovation Center of Intelligent Diagnosis, Jinan Central Hospital, Shandong University, Jinan, China; ^2^Department of Pediatics, Central Hospital Affiliated to Shandong First Medical University, Jinan, China; ^3^General Medical Department, Jinan Central Hospital, Shandong University, Jinan, China

**Keywords:** COVID-19, epilepsy, bibliometric analysis, Web of Science, VOSviewer software

## Abstract

**Background:**

A large number of papers regarding coronavirus disease 2019 (COVID-19) and epilepsy have been published since the declaration of the COVID-19 pandemic. However, there is no bibliometric analysis on these papers. In this study, we aimed to analyze the bibliometric characteristics of these papers, thus identifying the trends and future directions of COVID-19 and epilepsy research.

**Methods:**

Scientific papers regarding COVID-19 and epilepsy were retrieved through searches of the Web of Science Core Collection database. Title, authors, contributing institute, country, source journal, times cited, and additional information were extracted from each selected paper. Microsoft Excel 2019 and GraphPad Prism 8 were used to analyze the extracted data and export the bar charts and tables whilst VOSviewer software was used to perform and visualize co-authorship analysis and co-occurrence analysis of keywords.

**Results:**

A total of 317 papers regarding COVID-19 and epilepsy were included in the final analysis. *Epilepsy & Behavior* published the largest number of papers (*n* = 84). J. Helen Cross and Naoto Kuroda were the most prolific authors (*n* = 13 each). The United States (*n* = 88) and the University of London (*n* = 23) were the country and organization with the most contributions, respectively. The strongest authors' collaborations were between Giovanni Assenza and Jacopo Lanzone and between J. Helen Cross and Nathalie Jette. Selected author keywords were organized into seven clusters, and the keywords in clusters 1 and cluster 4 had the largest average appearing year of any clusters.

**Conclusion:**

This is the first bibliometric analysis of papers regarding COVID-19 and epilepsy. Our results showed that the United States was the leading country whilst J. Helen Cross was the most influential scholar in COVID-19 and epilepsy research. psychological consequences of COVID-19, and the safety of COVID-19 vaccines for people with epilepsy, are possible areas for future research on COVID-19 and epilepsy.

## Introduction

Coronavirus disease 2019 (COVID-19) is a novel infectious disease caused by the 2019 novel coronavirus (2019-nCoV) or severe acute respiratory syndrome coronavirus 2 (SARS-CoV-2) which was identified through sequencing on 7 January, 2020 (1, 2). COVID-19 is one of the most serious epidemics in human history (3). According to the WHO Situation Report (https://covid19.who.int/) published at 5:59 pm CEST, 27 September 2022, 612,724,171 cases of COVID-19 including 6,517,123 deaths globally, had been confirmed.

Shortly after the declaration of the COVID-19 pandemic, suggestions and guidelines for the prevention and control of the novel coronavirus were released by related journals and major associations. Thus far, researchers worldwide have published a large number of papers regarding COVID-19 and epilepsy. Various epilepsies were increasingly being documented in numerous COVID-19 cases. In Italy, a report described a patient affected by COVID-19 whose primary presentation was a focal status epilepticus (4). Abdulsalam and colleagues reported a case of generalized status epilepticus as a possible initial manifestation of COVID-19 infection (5). These secondary epilepsies were mainly due to either the entry of pro-inflammatory cytokines into the nervous system or the production of these cytokines by microglia and astrocytes. Pro-inflammatory cytokines could cause blood-brain barrier disruption, increase in glutamate and aspartate and reduce GABA levels, impaired the function of ion channels, and finally, high levels of cytokines could cause epilepsy (6). Apart from new-onset epilepsies due to 2019-nCoV infection, the impact of COVID-19 on patients with pre-existing epilepsy had been immense. Several studies have reported on changes in seizure frequency among people with epilepsy (PWE) during the COVID-19 pandemic, regardless of whether these patients were infected with COVID-19 (7–10). There were theoretical risks of seizure worsening in PWE with a 2019-nCoV infection, for example, seizures triggered by fever. Moreover, a severe disease course and advanced disease stages could, for instance, result in hypoxic encephalopathy, cerebrovascular events, and cytokine storm, which may trigger the development of acute seizures. This was further confirmed by reports of occasional seizures in COVID-19 patients with epilepsy (11). In addition, the barriers to obtaining anti-seizure medications, care and clinic visits, and social restrictions leading to psychological distress, exposed PWE to an increase both in seizure frequency and severity (12). In short, many papers related to COVID-19 and epilepsy have been published, but no systematic analysis of the knowledge structure and development status on this issue has been conducted.

Bibliometric analysis is a statistical method which could quantitative analysis the research papers concerned about one special topic *via* mathematical ways. It could also access the quality of the studies, analysis the dominant areas of researches and predict the direction of future studies (13). The Web of Science (WOS) online database, which contains almost all the important research papers, is considered one of the most complete and available bibliometric tools (13, 14). What is more, the search results in WOS formats can be imported to a software for further analysis like VOSviewer (15).

Several bibliometric papers were published to investigate the status and trends of COVID-19 research in the medical field such as emergency medicine, depressive disorders, health disparities and related polymer technology (16–19). However, there is no bibliometric analysis of papers regarding COVID-19 and epilepsy. In this study, we performed a comprehensive analysis of the content and bibliometric characteristics of papers related to COVID-19 and epilepsy from a bibliometric perspective and then identified the research hotspots and future directions in this field.

## Materials and methods

### Database and searching strategy

In this study, the WoS Core Collections was used to search for papers regarding COVID-19 and epilepsy. The paper search strategy was as follows: [(Topic = COVID-19 OR Topic = SARS-CoV-2 OR Topic = coronavirus OR Topic = 2019-nCoV) AND Topic = epileps^*^]. The time span was set from 1 January 2020 to 31 August 2022.

**Figure 1:**
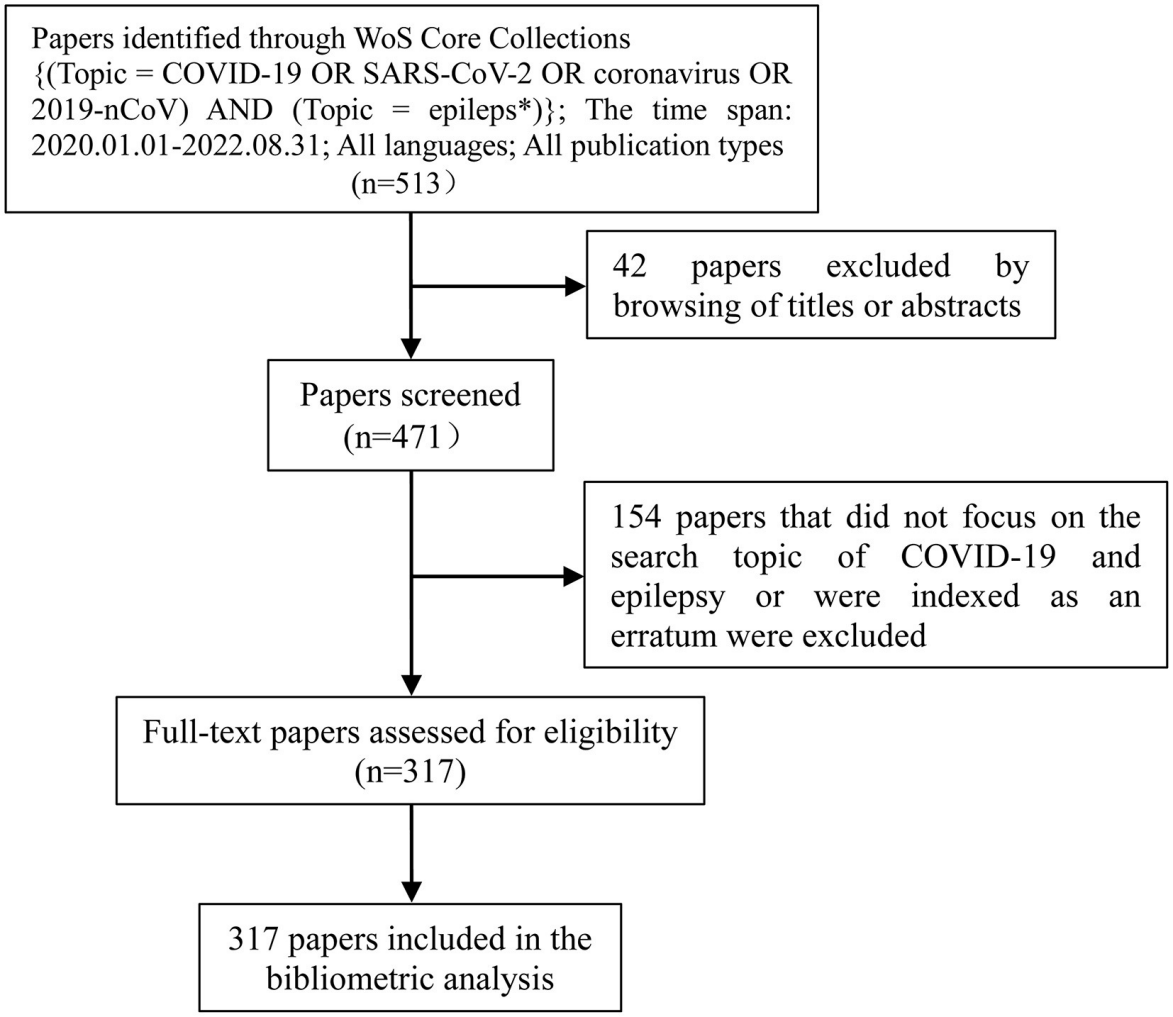
A frame flow diagram. The diagram showed detailed search strategy and the selection criteria for papers regarding COVID-19 and epilepsy from WoS core collections.

The authors independently searched the literature and examined each content on 11 June 2022 and updated on 19 September 2022. The inclusion and exclusion of the papers had been summarized in a frame flow diagram (Figure 1). We normalized the bibliographic information in these 317 papers using a manual review process and transcription of relevant information for each paper. Names of authors, institutions and their countries of origin, and keywords were all normalized to a standard format. In the case of ambiguity, cross-checking with the literature and the consensus among authors were conducted. Data including the titles of the papers, authors, publication years, contributing organizations, countries, source journals, keywords, abstracts, and others were extracted and saved to EndNote Desktop and Microsoft Excel 2019, respectively.

The data sources for this research were obtained from a public database. Ethics committee approval was not required.

### Data analysis and visualization

Microsoft Excel 2019 and GraphPad Prism 8 were used to analyze the selected papers and export the histograms and tables showing the paper type, language, source journals, categories, top 10 authors/organizations/countries, and most cited papers.

VOSviewer software (version 1.6.18) was used for bibliometric analysis, such as co-authorship analysis and keyword co-occurrence analysis, to realize data visualization.

## Results

### Paper type and language

Through the search strategy, we identified 317 papers regarding COVID-19 and epilepsy (Supplementary Data 1). The largest number of papers was observed to be original articles (*n* = 210), followed by reviews (*n* = 41). The remaining paper types were labeled as meeting abstract (*n* = 32), letter (*n* = 21), editorial material (*n* = 13), and early access (*n* = 10).

Of four paper languages, English was predominant with 310 papers (97.8% of all papers). Five papers were written in Spanish (1.6%), one was in Hungarian (0.3%), and one was in Turkish (0.3%).

### Analysis of source journals and categories

A total of 97 journals published 317 retrieved papers related to COVID-19 and epilepsy. The journals with 10 or more papers are reported in Table 1. These six journals (6.2% of the total journals) published 169 papers (53.3% of all papers). Among all the journals, 70 (72.2%) published only one paper. *Epilepsy & Behavior* published the largest number of papers, followed by *Epilepsia* (26.5 and 9.8% of all papers, respectively). The papers published in *Epilepsy & Behavior* were cited the most times (*n* = 755).

**Table 1:** List of journals with 10 or more papers related to COVID-19 and epilepsy.

**Journals**	**Number of papers**	**Times of citations**
Epilepsy & Behavior	84	755
Epilepsia	31	257
Seizure-European Journal of Epilepsy	20	310
Frontiers in Neurology	12	184
Acta Neurologica Scandinavica	11	121
Neurological Sciences	11	233

**Figure 2:**
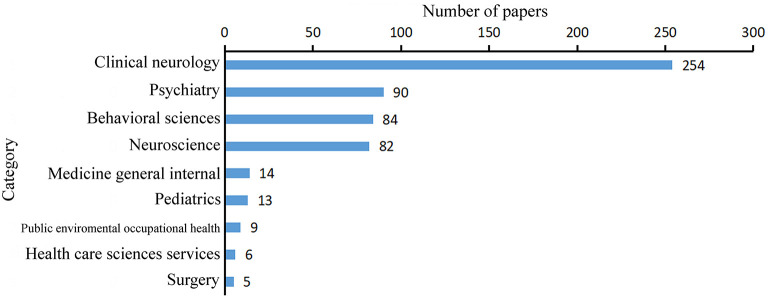
WoS categories with five or more papers related to COVID-19 and epilepsy.

Although WoS presented 254 categories, only 9 categories had five or more papers related to epilepsy and COVID-19 (Figure 2). “Clinical neurology” was predominant with 254 papers (80.1%), followed by “Psychiatry” (28.4%).

### Top 10 authors/organizations/countries ranked by the number of papers regarding COVID-19 and epilepsy

Table 2 shows the top 10 authors/organizations/countries ranked by the number of papers regarding COVID-19 and epilepsy. J Helen Cross and Naoto Kuroda published the largest number of papers (*n* = 13 each), followed by A. A. Asadi-pooya with 12 papers. The University of London was the organization with the largest number of papers (*n* = 23), followed by the University College London with 21 papers. Regarding countries, the United States ranked first with 88 papers, followed by Italy with 47 papers and China with 32 papers.

**Table 2:** Top 10 authors/organizations/countries ranked by the number of papers related to COVID-19 and epilepsy.

**Author**			**Organization**			**Country and region**		
**Name**	**Number of papers**	**Times of citations**	**Name**	**Number of papers**	**Times of citations**	**Name**	**Number of papers**	**Times of citations**
Cross JH	13	216	University of London	23	252	USA	88	918
Kuroda N	13	155	University College London	21	252	Italy	47	679
Asadi-pooya AA	12	127	Wayne State University	15	168	China	32	403
Sander JW	7	124	Shiraz University of Medical Science	13	128	England	27	327
Assenza G	6	123	Jefferson University	12	127	Canada	23	149
Specchio N	6	70	ERN EpiCARE	9	100	Spain	22	354
Baykan B	6	24	UDICE-French Research Universities	9	74	Japan	21	123
Brigo F	5	146	Tohoku University	9	67	India	20	226
Jette N	5	123	Great Ormond Street Hospital for Children	8	162	Iran	19	322
Zhou D	5	113	Sichuan University	8	121	Brazil	19	161

### Most cited papers regarding COVID-19 and epilepsy

There were 2,784 times of citations for 317 papers, with 9 papers having a minimum of 50 times of citations. The top 10 most cited papers related to COVID 19 and epilepsy are listed in Table 3. For papers with the same times of citations, recent papers were ranked higher because they had less chance to be cited. The 10 most cited papers included 7 original articles, 2 reviews, and 1 letter.

**Table 3:** Top 10 most cited papers related to COVID-19 and epilepsy.

**Title of the paper**	**References**	**Year published**	**Times of citations**	**Rank**
The neurological manifestations of COVID-19: a review article	Niazkar et al. (20)	2020	116	1
Focal status epilepticus as unique clinical feature of COVID-19: a case report	Vollono et al. (4)	2020	113	2
Keeping people with epilepsy safe during the COVID-19 pandemic	French et al. (21)	2020	87	3
Severe psychological distress among patients with epilepsy during the COVID-19 outbreak in southwest China	Hao et al. (22)	2020	84	4
Epilepsy care in the time of COVID-19 pandemic in Italy: Risk factors for seizure worsening	Assenza et al. (23)	2020	77	5
COVID-19 outbreak: the impact of stress on seizures in patients with epilepsy	Huang et al. (24)	2020	57	6
Epilepsy and COVID-19: associations and important considerations	Kuroda (25)	2020	57	6
COVID-19-associated neurological disorders: the potential route of CNS invasion and blood-brain barrier relevance	Achar et al. (26)	2020	55	8
Epilepsy in time of COVID-19: A survey-based study	Fonseca et al. (27)	2020	51	9
Incidence and case fatality rate of COVID-19 in patients with active epilepsy	Cabezudo-Garcia et al. (28)	2020	47	10

### Co-authorship analysis

Co-authorship analysis involves the evaluation of the relationship among authors/organizations/countries on the basis of the number of papers in which they occur together, which is considered as one of the most tangible indicators for assessing trends of collaboration and identifying leading countries, organizations, and researchers. In the visualization maps constructed by VOSviewer, different nodes represent different authors/organizations/countries, and the size of the nodes on the map is proportional to the number of papers. The lines between nodes represent cooperation relationships, and the line thickness measured by the indicator of link strength (LS) corresponds to number of papers co-authored. Total link strength (TLS) is the sum of all LS of a given author/organization/country and indicates how strongly the author/organization/country is connected to others (29). The distance and thickness of the connecting curves between nodes reflect the relevance and strength of their co-authorship, respectively.

**Figure 3:**
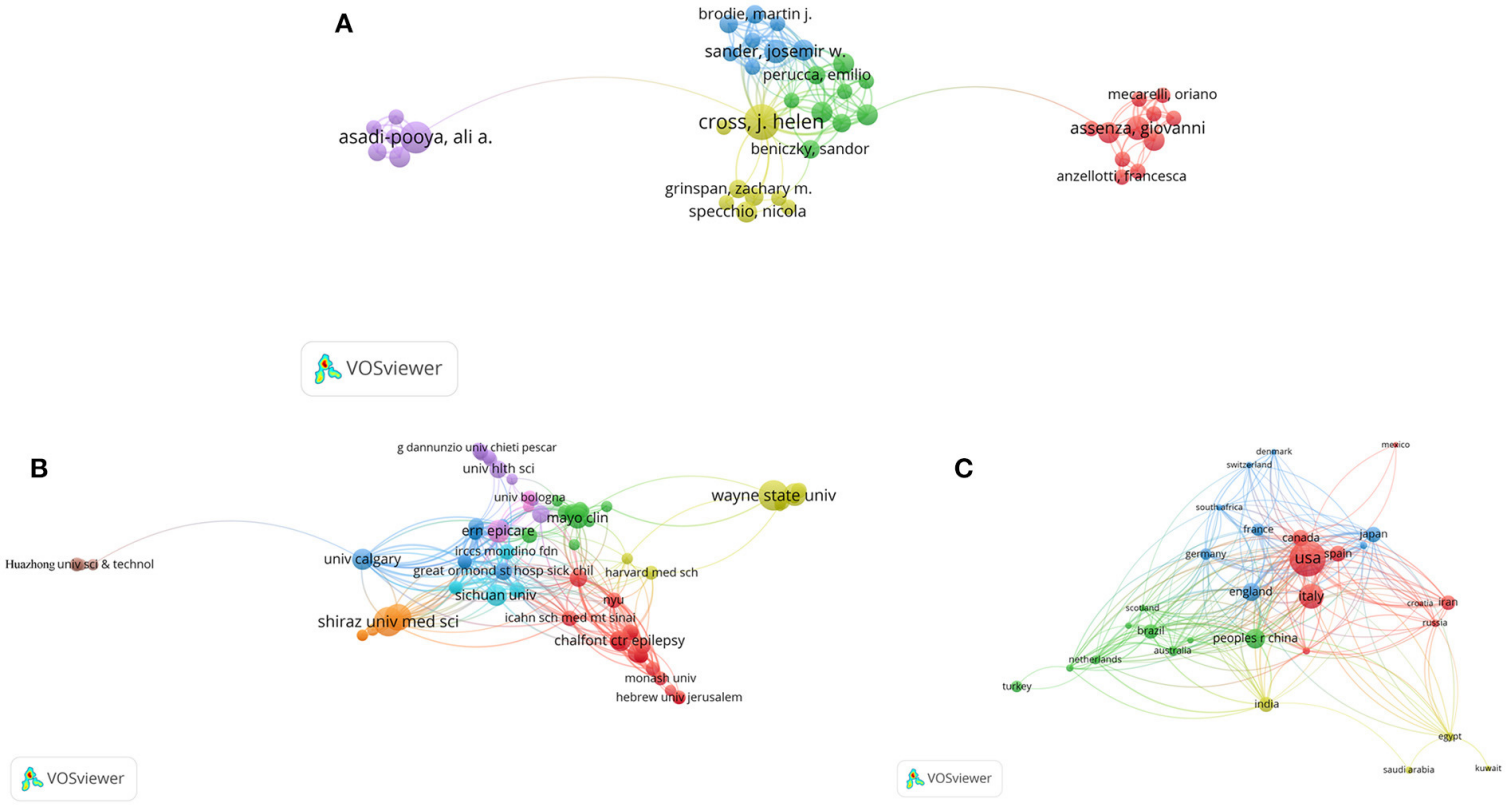
Network visualization of co-authorship analysis of COVID-19 and epilepsy research. Network visualization of **(A)** authors, **(B)** organizations, and **(C)** countries in the research field. The sizes of the points represent co-authorship frequency. The line between two points indicates that two authors/organizations/countries had established collaboration, and a thicker line indicates closer collaboration. The authors/organizations/countries whose collaboration was high are depicted by the colors of the circles.

For the purpose of authors' co-authorship analysis, the minimum number of papers per author was set to 3, and the minimum times of citations of an author was set to 0. A total of 74 authors met the thresholds and were selected for co-authorship analysis. As shown in the network visualization map of authors' co- authorship (Figure 3A), J. Helen Cross showed the highest collaboration performance with a TLS of 61, followed by Nathalie Jette (TLS = 41), Josemir W. Sander (TLS = 27), Eugen Trinka (TLS = 27), and Samuel Wiebe (TLS = 27). The strongest collaboration was between Giovanni Assenza and Jacopo Lanzone (LS = 5) and between J. Helen Cross and Nathalie Jette (LS = 5).

Similarly, we conducted co-authorship analysis on 795 organizations involved in COVID-19 and epilepsy research. The minimum number of papers of an organization was set to 3, and the minimum times of citations of an organization was set to 0. A total of 91 organizations finally met the thresholds and were selected for co-authorship analysis. As shown in Figure 3B, the ERN EpiCARE (France) had the highest collaboration performance with a TLS of 65, followed by the Icahn School of Medicine at Mount Sinai (USA) (TLS = 60), Great Ormond Street Hospital for Children (UK) (TLS = 59), and UCL NIHR BRC Great Ormond Street Institute of Child Health (UK) (TLS = 54). The strongest collaboration was between Tohoku University (Japan) and Wayne State University (USA) (LS = 9), followed by that between Chalfont Center for Epilepsy (UK) and UCL Queen Square Institute of Neurology (UK) (LS = 5).

In terms of countries, the minimum number of papers in a country was set to 3, and the minimum times of citations of a country was set to 0. A total of 34 countries met the thresholds and were selected for analysis. Researchers from the United States showed the highest collaboration performance with a TLS of 130, followed those from England (TLS = 108), Canada (TLS = 86), China (TLS = 76), and Italy (TLS = 64). The international collaboration network is presented in Figure 3C. The strongest collaboration was between the United States and Japan (LS = 14), the United States and England (LS = 13), and the United States and Iran (LS = 12).

### Keyword co-occurrence analysis

Keyword co-occurrence analysis is a quantitative study of keywords and their basic characteristics, such as the frequency of appearance, development, and evolution. This allows for an understanding of the research focus and development trends in related fields. In the network visualization map *via* VOSviewer, a node indicates a keyword, and the size of each node indicates how many papers include the keyword. Larger nodes indicate greater keyword popularity. The keyword relationship is indicated by the link line between two nodes. The LS between two nodes reflects the frequency of the keyword's co-occurrence. A keyword's TLS is the sum of its LSs. Keywords that are closely related are grouped into a cluster represented by a specific color through clustering analysis. This cluster reflects a core research field that the keywords refer to. In the overlay visualization map, nodes are colored according to the average appearing year (AAY) of the keywords.

**Figure 4:**
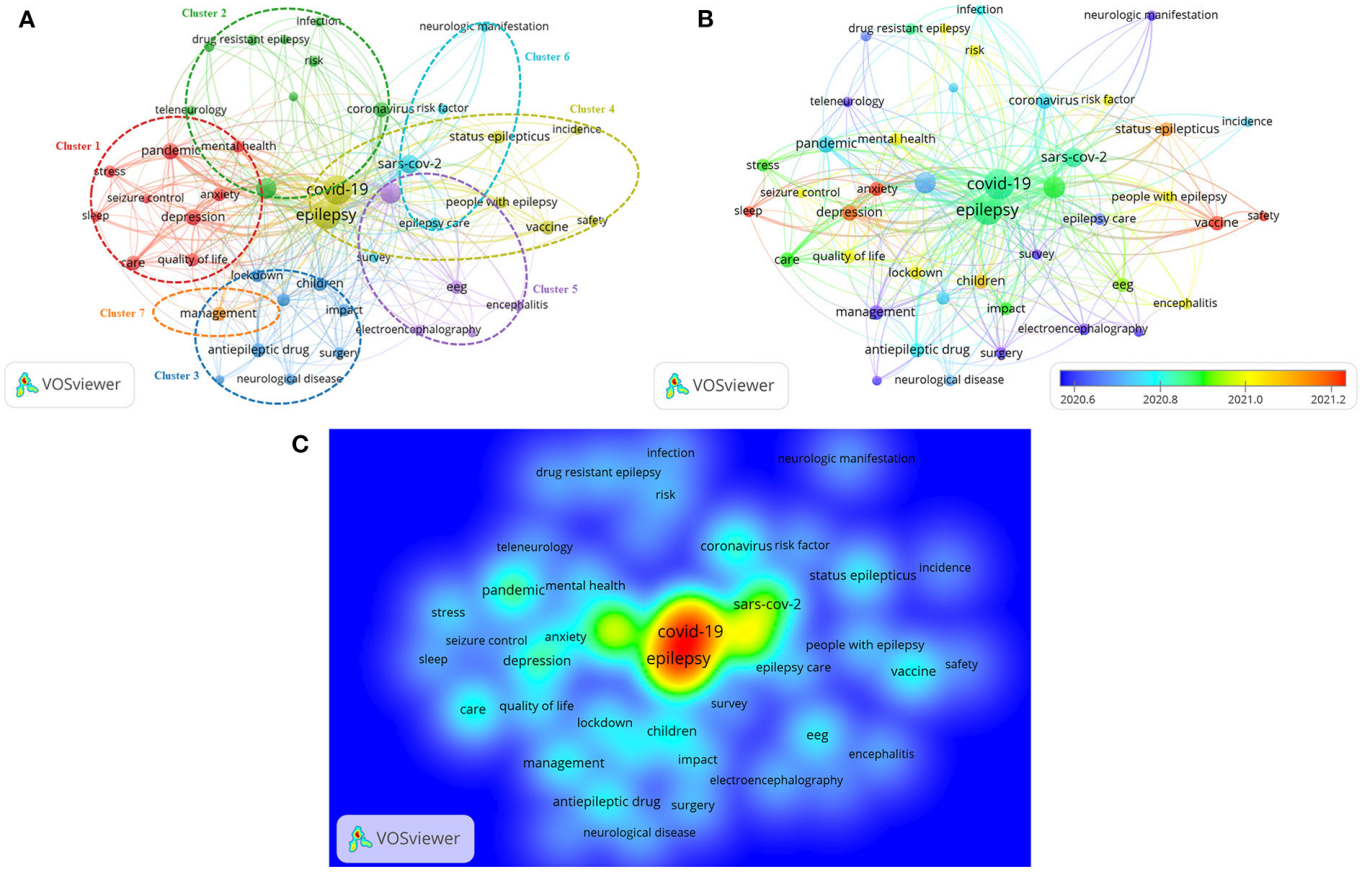
Keyword co-occurrence analysis. **(A)** Network visualization map of keyword co-occurrence analysis. The sizes of the points represent the frequency of keyword co-occurrence, and the keywords are divided into seven clusters: psychological consequences of COVID-19 in PWE (cluster 1), telemedicine for epilepsy in the COVID-19 era (cluster 2), impact of lockdown on children with epilepsy (cluster 3), safety of COVID-19 vaccines in PWE (cluster 4), EEG of seizure related to COVID-19 (cluster 5), risk factors for SARS-CoV-2 infection in PWE (cluster 6), and the epilepsy management during the period of COVID-19 (cluster 7). **(B)** Overlay visualization map of keyword co-occurrence analysis. The colors of the nodes indicate the AAY of the keyword co-occurrences. Keywords in white appeared earlier than those in yellow whilst keywords in red appeared the latest. **(C)** Density visualization map of keyword co-occurrence analysis. The map shows the distribution of keywords based on their average frequency of appearance. Keywords in red appeared most frequently, followed by those in yellow, green, cyan and blue.

A total of 472 author keywords were collected from 317 papers regarding COVID-19 and epilepsy. In addition to COVID-19, epilepsy, and their alternative titles, the main keywords included seizure (occurrences: 74), telemedicine (occurrences: 66), depression (occurrences: 24), care (occurrences: 20), and vaccine (occurrences: 18). For keyword co-occurrence analysis, the minimum number of occurrences of an author keyword was set to 4. A total of 44 author keywords met the thresholds. As shown in the network visualization map (Figure 4A), all selected keywords were organized into seven clusters. Cluster 1 was the largest cluster that represented the current research focus. In this cluster, the prominent keywords were mental health, stress, depression and anxiety. As for cluster 2, the frequently used keywords were telemedicine and teleneurology. In cluster 3, the primary keywords were lockdown, impact and children. Cluster 4 consisted of keywords such as safety and vaccine. In cluster 5, the dominant keyword was EEG. The prominent keyword in cluster 6 was risk factor. The keyword “management” was frequently used in cluster 7 (Table 4).

**Table 4:** The clusters organized by keyword co-occurrence analysis.

**Clusters**	**Research hotspots**	**Number of items**	**Main keywords**
Cluster 1	Psychological consequences of COVID-19 in PWE	9	Mental health, stress, anxiety, depression, sleep,
Cluster 2	Telemedicine for epilepsy in the COVID-19 era	9	Telemedicine, ketogenic diet, SUDEP, teleneurology
Cluster 3	Impact of lockdown on children with epilepsy	8	Impact, lockdown, children
Cluster 4	Safety of COVID-19 vaccines in PWE	7	Safety, COVID-19, vaccine, PWE
Cluster 5	EEG of seizure related to COVID-19	5	EEG, seizure, encephalopathy
Cluster 6	Risk factors for SARS-CoV-2 infection in PWE	5	Risk factor, SARS-CoV-2, neurologic manifestation
Cluster 7	The epilepsy management during the period of COVID-19	1	Management

In Figure 4B, the overlay visualization map shows the changes in keywords over time; here, the keywords are assigned different colors based on their AAY. The blue nodes indicated that the keywords appeared earlier in the time course, whereas the red nodes indicate the recent occurrence of keywords. Early research in this field is mainly focused on “Risk factors for SARS-CoV-2 infection in PWE” and “Impact of lockdown on children with epilepsy.” Afterwards, keywords such as “EEG,” “telemedicine” and “risk factor” started to emerge. The keywords in clusters 1 and 4 had the largest AAY, indicating that the topics in these two clusters had gained increased amounts of attention. Furthermore, the keywords “safety,” “sleep,” “anxiety” and “vaccine” showed relatively recent AAYs of 2021.33, 2021.33, 2021.29 and 2021.27, respectively; these results indicate that they have become more common and are likely to remain major areas of research in the future.

In the density visualization, the keywords are distributed according to their average frequency of appearance. The keywords in red occurred with the highest frequency, followed by those in yellow, green, cyan and blue (Figure 4C).

## Discussion

### Status of COVID-19 and epilepsy research

Scientific research plays an important role in preventing and managing diseases, especially during the COVID-19 pandemic (30, 31). Although a large body of scientific literature on COVID-19 and epilepsy has been produced since the COVID-19 outbreak, a more profound understanding of the global landscape of COVID-19 and epilepsy research is necessary.

In this study, the bibliometric analysis summarized 317 papers on COVID-19 and epilepsy and provided insights into the research status through assessment of the following bibliometric parameters: source journals, leading authors/organizations/countries and their collaborations, citation analysis, and term clustering. *Epilepsy & Behavior* published the highest number of papers regarding COVID-19 and epilepsy (26.5% of the 317 papers), followed by *Epilepsia* (9.8% of the papers). J. Helen Cross from the United Kingdom and Naoto Kuroda from the United States published the largest number of papers. Moreover, J. Helen Cross's papers recorded the most times of citations, and author's co-authorship analysis showed that J. Helen Cross had the highest number of collaborations with other researchers, suggesting that J. Helen Cross was the most influential scholar on COVID-19 and epilepsy research.

The organizations that published relevant papers mainly come from the United Kingdom and the United States. Previous studies found that the United States and the United Kingdom had the similar trend in epilepsy research before COVID-19 pandemic (32, 33). The University of London in the United Kingdom published the largest number of papers on COVID-19 and epilepsy possibly because of several different factors, including its status as one of the largest universities in the United Kingdom, its considerable resources for medical research and prolific research groups, and its location in a country that has been significantly affected by the pandemic. Meanwhile, the United States produced the most outputs regarding COVID-19 and epilepsy with a rate of over 25% of the total papers related to COVID-19 and epilepsy; this result is consistent with previous bibliometric analyses of the COVID-19 literature in medicine (18, 34). This finding may be attributed to several factors, including the large size of its financial support or population, its status as one of the most severely affected by the pandemic, and its record as being the most active in the global development of scientific research and international cooperation networks. According to continents, 193 papers originated from the countries of Europe, including the United Kingdom, Italy, Spain, Germany, Switzerland, Denmark, France, Austria and Sweden, 139 papers originated from the Asia, including China, Japan, India and Iran, 115 papers originated from the North American continent, including the United States, Canada and Mexico, and only 11 papers originated from the countries on Oceania, including Australia and New Zealand.

### Top 10 most cited papers related to COVID-19 and epilepsy

Our results identified the top 10 most cited papers regarding COVID-19 and epilepsy, including 7 original articles, 1 letter, and 2 reviews.

Among the seven original articles, the article “Focal status epilepticus as unique clinical feature of COVID-19: a case report” published in *Seizure* received the second most times of citations; this article described the first patient who developed focal status epilepticus as initial presentation of COVID-19. In the third most cited paper, information was provided on the impact of the COVID-19 pandemic on PWE, and consensus recommendations were developed on how to provide the best possible care for PWE during the pandemic. The fourth and fifth most cited papers focused on the mental health of patients with epilepsy during the pandemic. The sixth most cited paper concluded that stress might be an independent factor for triggering seizures in some patients with epilepsy. The eighth most cited paper found that the COVID-19 pandemic affected patients with epilepsy and that the frequency of seizures was higher in patients with insomnia, drug-resistant epilepsy, and economic difficulties. The tenth most cited paper showed that the cumulative incidence of COVID-19 in epilepsy patients was higher than that in the population without epilepsy.

The seventh most cited paper, a letter to the editor, was written by N. Kuroda from Wayne State University in the United States. It reviewed COVID-19's association with epilepsy and highlighted suggestions from medical societies. The author concluded that the effect of COVID-19 on individuals with epilepsy remains unclear.

Two reviews primarily discussed the correlation between COVID-19 and epilepsy, and highlighted its possible mechanisms (4, 27). On one aspect, they concluded that it is not clear whether patients with epilepsy are at higher risk of COVID-19 than others. One cross-sectional study found that active epilepsy would be an independent risk factor for COVID-19 incidence and mortality (26). However, another study conducted in Spain and Italy showed that out of 5,700 patients with epilepsy, only 14 tested positive for COVID-19 (35). It is also possible that COVID-19 may cause epilepsy as epilepsy is being papered in numerous COVID-19 cases. In Italy, a report described a patient affected by COVID-19 whose main manifestation was focal status epilepticus (20). Abdulsalam et al. reported a case of generalized status epilepticus as a primary presentation of COVID-19 infection (5). It was hypothesized that the pathology of epilepsy originating from SARS-CoV-2 infection is mainly due to either the entry of pro-inflammatory cytokines into the nervous system or the production of these cytokines by microglia and astrocytes (6, 36). Pro-inflammatory cytokines disrupted the blood-brain barrier, increase glutamate and aspartate levels, and decrease gamma-aminobutyric acid levels, and impair ion channel function, thus leading to epilepsy (9). Apart from new-onset epilepsies due to SARS-CoV-2 infection, patients with pre-existing epilepsy have been greatly affected by COVID-19. Several studies have found that seizures in PWE worsened during the pandemic regardless of whether these patients were infected with COVID-19 (7, 9, 10). There are theoretical risks of seizure worsening in PWE with a SARS-CoV-2 infection. Fever, for instance, could trigger seizures. Additionally, the severe course and advanced stages of the disease could lead to hypoxic encephalopathy, which may also trigger the occurrence of acute seizures. This is further confirmed by reports of occasional seizures in PWE infected with COVID-19 (11). Social restriction exposes PWE to an increase in seizure frequency and severity. In addition to the barriers to obtaining medications, care and clinic visits, PWE also suffered from isolation, thus leading to feelings of loneliness, boredom, anger, and anxiety (37).

### Research hotspots and future directions

Through the co-occurrence analysis of keywords, we constructed and visualized a keyword network to explore the hotspots and future directions of COVID-19 and epilepsy research.

Of the seven organized clusters, cluster 1 showed one of the current research hotspots on COVID-19 and epilepsy, that is, psychological comorbidities in PWE during the COVID-19 pandemic, with the keywords being depression, anxiety, sleep, and psychological distress. Psychological problems from COVID-19 have been reported to be greater in patients with epilepsy than in healthy people; such issues might increase the seizure frequency of patients with epilepsy (38, 39). The paper titled “The effects of coronavirus disease 2019 (COVID-19) pandemic on people with epilepsy (PwE): an online survey-based study” published in *Acta Neurol Belg* in 1997 by Abokalawa et al. was cited 13 times. This study showed that two-thirds of the patients with epilepsy (66.2%) reported depression, 72.2% reported anxiety, and 75.5% reported stress (40). The basic principles for psychological crisis interventions for PWE during COVID-19 should be established. Cluster 2 showed another research hotspot: telemedicine for epilepsy in the COVID-19 era. Telemedicine could promote remote clinical consultations for new and follow-up patients with epilepsy whilst reducing the risk of infection of patients and healthcare staff. von Wrede et al., article named “Counseling of people with epilepsy *via* telemedicine: Experiences at a German tertiary epilepsy center during the COVID-19 pandemic” was published in *Epilepsy Behav* in 2020. In this retrospective study, PWE appeared to be satisfied with telemedical counseling (41). Cluster 3 focused on the impact of lockdown on children with epilepsy. *Seizure* published an article in 2021 written by Wanigasinghe et al., whose name was “Experience during COVID-19 lockdown and self-managing strategies among caregivers of children with epilepsy: A study from low middle income country.” This study concludeded that lockdown status for COVID-19 did not significantly affect the control of epilepsy in children though it posed difficulties for regular reviews and obtaining medications, and self-management strategies would help caregivers to adopt to new-normal status and potential future outbreaks (42). In the fourth cluster, the safety of COVID-19 vaccines in PWE was emphasized. A cross-sectional study carried by Massoud F. found that the two vaccines under consideration (BNT162b2 and ChAdOx1nCoV-19) had a good safety profile and a low risk of epilepsy worsening among a cohort of PwE in Kuwait (43). Cluster 5 covered EEG of seizure related to COVID-19. Antony AR et al. performed a systematic study of the EEG findings in patients with COVID-19 and found that EEG abnormalities were common in COVID-19 related encephalopathy and correlated with disease severity, preexisting neurological conditions including epilepsy and prolonged EEG monitoring. They also found that frontal findings were frequent and had been proposed as a biomarker for COVID-19 encephalopathy (44). Cluster 6 was related to the risk factors for SARS-CoV-2 infection in PWE. An article named “COVID-19 among patients with epilepsy: Risk factors and course of the disease” was published in *Epilepsy Behav* in 2021. This research found that patients with epilepsy might be at increased risk of SARS-CoV-2 infection and meanwhile provided reassuring findings related to the low risk of seizure exacerbation in PWE during the course of COVID-19 (45). In the seventh cluster, the epilepsy management during the period of COVID-19 was focused on. Jain et al.' paper named “Management of COVID-19 in patients with seizures: Mechanisms of action of potential COVID-19 drug treatments and consideration for potential drug-drug interactions with anti-seizure medications” demonstrated the management of acute seizures in patients with COVID-19 as well as management of PWE and COVID19 needed to consider potential drug-drug interactions between antiseizure drugs and candidate drugs currently assessed as therapeutic options for COVID-19 (46).

In terms of the future directions, an overlay visualization showed the evolution of keywords over time in each cluster, from the initial research focus on “Risk factors for SARS-CoV-2 infection in PWE” and “Impact of lockdown on children with epilepsy” to the current dimension of “psychological consequences of COVID-19 in PWE” and “Safety of COVID-19 vaccines in PWE.” The visualization indicated that the latter two clusters of topics are likely to become a research hotspot in the future. Density visualization also identified a number of “remote” keywords, such as vaccine, safety and anxiety. Although these keywords may not represent the whole field, they still open up important questions and indicate that future research is likely to focus on these topics.

### Strengths and limitations of this study

This is the first research to evaluate the bibliometric characteristics of papers related to COVID-19 and epilepsy. Compared with the traditional literature review, the bibliometric analysis by using VOSviewer is more comprehensive, objective and intuitive. However, there are several limitations that need to be considered when interpreting our findings. Firstly, our analysis was based on the WoS Core Collection database; as other databases, such as PubMed, Scopus, and Google Scholar, were not included, selection bias may emerge. Secondly, bibliometric data change over time, and different conclusions may consequently be drawn as time passes. Hence, future updates are needed for this study. Thirdly, it is possible that this bibliometric analysis does not accurately reflect reality. For example, some relatively new high-quality publications may not be given sufficient attention due to the lower citation frequency, whilst older articles tend to accumulate more citations. A multi-method evaluation is needed to gain a deeper understanding of this research field.

## Conclusions

To the best of our knowledge, this is the first bibliometric analysis of scientific papers regarding COVID-19 and epilepsy. Our results showed that the United States was the leading country whilst J. Helen Cross was the most influential scholar in COVID-19 and epilepsy research. Psychological consequences of COVID-19 and safety of COVID-19 vaccines for PWE are possible future directions in the study of COVID-19 and epilepsy.

## Data availability statement

The original contributions presented in the study are included in the article/Supplementary material, further inquiries can be directed to the corresponding author/s.

## Author contributions

GW and SW contributed toward conception, design, planning of the study, and interpretation of the results. GW, LB, and MZ contributed toward analysis of data. GW, MZ, and SW contributed toward drafting of the manuscript. All authors contributed toward acquisition of data, critical revision of the manuscript for important intellectual content, approval of the final version of the manuscript, agree to be accountable for all aspects of the work, and ensure that any questions related to the accuracy or integrity of any part of the work will be appropriately investigated and resolved.

## Funding

This work was supported by Science and Technology Project of Jinan (Grant Number 202019139) and the Natural Science Foundation of Shandong Province, China (Grant Number ZR2019MH043). The funding sources had no role in study planning, implementation, and reporting of study results or interpretation.

## Conflict of interest

The authors declare that the research was conducted in the absence of any commercial or financial relationships that could be construed as a potential conflict of interest.

## Publisher's note

All claims expressed in this article are solely those of the authors and do not necessarily represent those of their affiliated organizations, or those of the publisher, the editors and the reviewers. Any product that may be evaluated in this article, or claim that may be made by its manufacturer, is not guaranteed or endorsed by the publisher.
