# Supplementary material for: Global landscape of COVID-19 and epilepsy research: A bibliometric analysis
Source: Front Neurol. 2022 Oct 24;13:1034070. doi: 10.3389/fneur.2022.1034070 (PMC9637663; doi:10.3389/fneur.2022.1034070)
Supplement: Supplementary file 1 [file Data_Sheet_1.PDF]

Supplementary Data S1. Data of 317 papers regarding COVID-19 and epilepsy

FN Clarivate Analytics Web of Science
VR 1.0
PT J
AU Armeno, M
Caballero, E
Verini, A
Reyes, G
Galarza, N
Cresta, A
Caraballo, RH
AF Armeno, M.
Caballero, E.
Verini, A.
Reyes, G.
Galarza, N.
Cresta, A.
Caraballo, R. H.
TI Telemedicine- versus outpatient-based initiation and management of ketogenic diet therapy in children with drug-resistant epilepsy during the COVID-19 pandemic
SO SEIZURE-EUROPEAN JOURNAL OF EPILEPSY
DT Article
DE Telemedicine; Ketogenic diet; Drug resistant epilepsy; COVID-19
ID INTERNATIONAL LEAGUE; ILAE COMMISSION; POSITION PAPER; CLASSIFICATION; EFFICACY
AB Introduction: Initiation of ketogenic diet therapies (KDT) for pediatric epilepsy is usually done on an inpatient basis and the diet is managed during clinical appointments following a protocol of visits and routine tests. Because of the 2019 coronavirus disease (COVID-19) pandemic and the associated lock-down measures, we switched from outpatient to telemedicine-based KDT initiation. Objective: To explore the feasibility, effectiveness, and safety of online KDT initiation and follow-up by comparing a group of children with drug-resistant epilepsy that was managed by telemedicine compared to a group that was treated on an outpatient basis. Materials and Methods: An observational study was conducted in two groups of patients with drug-resistant epilepsy who initiated KDT and were followed up with an online versus an outpatient modality by the interdisciplinary KDT team of Hospital Pediatria JP Garrahan in Buenos Aires, Argentina. Dietary compliance, ketosis, retention rate, adverse effects, number of contacts, and clinical outcome were evaluated at 1, 3, and 6 months on the diet. Results: Overall, 37 patients were included, of whom 18 started the KD by telemedicine and 19 on an outpatient basis. Minimum follow-up of the patients was 6 months. All patients received the classic ketogenic diet. No statistical differences between the two groups regarding efficacy and safety of the diet were found. Conclusions: Our results support the feasibility and safety of initiating and management of KDT by telemedicine. Patients and their families should be carefully selected in order to guarantee a good outcome.

C1 [Armeno, M.; Verini, A.] Hosp Pediat JP Garrahan, Dpt Clin Nutr, Buenos Aires, DF, Argentina.
[Caballero, E.; Galarza, N.; Cresta, A.] Hosp Pediat JP Garrahan, Food Serv Area, Buenos Aires, DF, Argentina.
[Reyes, G.; Caraballo, R. H.] Hosp Pediat JP Garrahan, Dpt Neurol, Buenos Aires, DF, Argentina.
C3 Hospital de Pediatria Doctor Juan Garrahan; University of Buenos Aires;
Hospital de Pediatria Doctor Juan Garrahan; University of Buenos Aires;
Hospital de Pediatria Doctor Juan Garrahan; University of Buenos Aires
RP Armeno, M (通讯作者), Hosp Pediat JP Garrahan, Dpt Clin Nutr, Buenos Aires, DF, Argentina.
EM marmeno@garrahan.gov.ar
TC 0
Z9 0
PD MAY
PY 2022
VL 98
BP 37
EP 43
DI 10.1016/j.seizure.2022.03.023
WC Clinical Neurology; Neurosciences
ER

PT J
AU Qin, LL
Chen, S
Feng, XL
Luo, BG
Chen, YW
AF Qin, Lulu
Chen, Si
Feng, Xianglin
Luo, Bangan
Chen, Yiwei
TI Patient-Perceived Health System Responsiveness of the Epilepsy
Management Project in Rural China during the Period of COVID-19
SO HEALTHCARE
DT Article
DE responsiveness; HSR; epilepsy; Management; COVID-19
ID GLOBAL CAMPAIGN; CARE SERVICES
AB Health system responsiveness (HSR) measures the experience of health-system users in terms of the non-clinical domains of the health system, which has been regarded as the three major goals of health performance evaluation. Good HSR may promote the use of health services and ultimately the health of patients. However, the HSR has not been measured as the main goal of the Epilepsy Management Project (EMP) in rural China. This study aims to evaluate the levels and distributions of the patient-perceived HSR of the EMP in rural China during the period of COVID-19 and identify its relevant factors

so as to provide advice on the improvement of further strategies. Based on the key informant survey (KIS) of responsiveness from the World Health Organization proposal, we conducted a cross-sectional survey of 420 epilepsy patients selected proportional randomly from seven rural areas in the Hunan province of China in 2021. Eight domains of patients-perceived HSR were assessed by face-to-face interview. The overall HSR scored at a fairly "good" level of 8.3 (8.3 out of a maximum of 10.0). During the COVID-19 period, the scores of responsiveness domains were highest at 8.66 to 8.93 in "confidentiality", "dignity" and "choice of providers", while lowest at 8.38 to 8.53 in "prompt attention", "social support" and "basic amenities". The representative responsiveness equality index (REI) was 0.732, indicating the moderately balanced distributions of responsiveness of the EMP in rural China. Female, old age, and low education were significantly related to the lower HSR scores of rural EMP ($p < 0.05$). The HSR of EMP in rural China was fairly good. However, measures to improve the patient-perceived HSR are still needed, especially including better service, higher social support, and more comfortable medical environments.
C1 [Qin, Lulu; Chen, Si; Feng, Xianglin] Hunan Normal Univ, Sch Med, Dept Social Med & Hlth Management, Changsha 410013, Peoples R China.
[Luo, Bangan] Brain Hosp Hunan Prov, Dept Mental Hlth, Changsha 410007, Peoples R China.
[Luo, Bangan] Cent South Univ, Xiangya Sch Publ Hlth, Dept Social Med & Hlth Management, Changsha 410078, Peoples R China.
[Chen, Yiwei] Cent South Univ, Xiangya Hosp 3, Dept Neurol, Changsha 410017, Peoples R China.
C3 Hunan Normal University; Central South University; Central South University
RP Luo, BG (通讯作者), Brain Hosp Hunan Prov, Dept Mental Hlth, Changsha 410007, Peoples R China.; Luo, BG (通讯作者), Cent South Univ, Xiangya Sch Publ Hlth, Dept Social Med & Hlth Management, Changsha 410078, Peoples R China.; Chen, YW (通讯作者), Cent South Univ, Xiangya Hosp 3, Dept Neurol, Changsha 410017, Peoples R China.
EM powerestlulu@163.com; miraitowasi@163.com; xianglin9310000@163.com; luo276@126.com; cyw9778@163.com
TC 0
Z9 0
PD MAY
PY 2022
VL 10
IS 5
AR 799
DI 10.3390/healthcare10050799
WC Health Care Sciences & Services; Health Policy & Services
ER

PT J
AU Watila, MM
Duncan, C
Mackay, G
AF Watila, Musa Mamman

Duncan, Callum
Mackay, Graham
TI Evaluation of telemedicine for new outpatient neurological consultations
SO BMJ NEUROLOGY OPEN
DT Article
DE clinical neurology; COVID-19; telemedicine
ID EPILEPSY; SAFETY; TRIAL; CARE
AB Objective The COVID-19 pandemic has broadened the use of teleneurology, how this compares with face-to-face (F2F) clinics is unclear. This study compared virtual with F2F new neurological consultations. Methods We retrospectively evaluated new outpatient consultations in neurology clinics in Aberdeen Royal Infirmary. We compared sociodemographic data, time to consultation, time to diagnosis, the need for reassessment and re-investigation between traditional F2F and virtual clinics using the web-based Attend Anywhere platform or telephone into patients' own homes (or chosen location) without a trained assistant. We calculated the relative risk (RR) of the need for reassessment and re-investigation over 6-month periods by the suspected neurological diagnosis. Results 73% of consultations were virtual (Attend Anywhere or telephone) between June and October 2020, this was almost non-existent (<0.1%) in June-October 2019. We analysed 352 F2F (June-July 2019) and 225 virtual consultations (June-July 2020). Compared with F2F clinics, virtual clinics had a longer time to diagnosis (p=0.019), were more likely to be reassessed (RR: 2.2, 95% CI: 1.5 to 3.2; p<0.0001) and re-investigated (RR: 1.50, 95% CI: 0.88 to 2.54; p=0.133), this was likelier in those aged >= 60 years. Patients with headaches and suspected seizures were less likely to need reassessment or re-investigation following virtual clinics than multiple sclerosis and neuroinflammatory disorders, spinal cord disorders and functional neurological disorders. Conclusion This study demonstrates that virtual clinics have higher rates of reassessment and re-investigation than F2F clinics. As virtual clinics become a potential consultation alternative, this study should instruct the selection of patients for either consultation type.
C1 [Watila, Musa Mamman; Duncan, Callum; Mackay, Graham] Aberdeen Royal Infirm, Dept Neurol, Aberdeen, Scotland.
[Watila, Musa Mamman] UCL Queen Sq Inst Neurol, Dept Clin & Expt Epilepsy, London, England.
C3 University of Aberdeen; University of London; University College London
RP Watila, MM (通讯作者)，Aberdeen Royal Infirm, Dept Neurol, Aberdeen, Scotland.; Watila, MM (通讯作者)，UCL Queen Sq Inst Neurol, Dept Clin & Expt Epilepsy, London, England.
EM musa.watila@nhs.scot
TC 0
Z9 0
PD MAY
PY 2022
VL 4
IS 1
AR e000260
DI 10.1136/bmjno-2021-000260
WC Clinical Neurology

ER

PT J
AU Romozzi, M
Rollo, E
Quintieri, P
Dono, F
Evangelista, G
Consoli, S
Veleno, L
Anzellotti, F
Calvello, C
Costa, C
Servidei, S
Calabresi, P
Vollono, C
AF Romozzi, Marina
Rollo, Eleonora
Quintieri, Paolo
Dono, Fedele
Evangelista, Giacomo
Consoli, Stefano
Veleno, Luigi
Anzellotti, Francesca
Calvello, Carmen
Costa, Cinzia
Servidei, Serenella
Calabresi, Paolo
Vollono, Catello
TI Impact of COVID-19 vaccine on epilepsy in adult subjects: an Italian multicentric experience
SO NEUROLOGICAL SCIENCES
DT Article
DE COVID-19; Coronavirus; Epilepsy; Vaccine; Seizure
ID SEIZURES
AB Objectives To investigate the safety and tolerability of COVID-19 vaccines in people with epilepsy (PwE). Methods In this multicentric observational cohort study, we recruited adult patients (age > 18 years old) with epilepsy who attended the Outpatient Epilepsy Clinic from 1st July to 30th October 2021. We administered to the patients a structured questionnaire and interview on demographic and epilepsy characteristics, current treatment, previous SARS-CoV-2 infection, vaccine characteristics, post-vaccine seizure relapse, other side effect, variation of sleep habits, caffeine, or alcohol intake. Seizure frequency worsening was defined as a ratio between mean monthly frequency post-vaccination and mean monthly frequency pre-vaccination superior to 1. Patients were categorized in two groups: patients with seizure frequency worsening (WORSE) and patients with seizure stability (STABLE). Results A total of 358 people participated with a mean age of 47.46 +/- 19.04. Focal seizure (79.1%), generalized

epilepsy (20.4%), and unknown types of epilepsy (0.5%) were detected among participants. In total, 31 (8.7%) people expressed that they were not willing to receive a COVID-19 vaccine; 302 patients (92.35%) did not experience an increase in the seizure frequency (STABLE-group) whereas 25 patients (7.65%) had a seizure worsening (WORSE-group). Post-vaccine seizures occurred mainly in the 7 days following the administration of the vaccine. Patients in the WORSE-group were treated with a mean higher number of anti-seizure medication (ASMs) ($p = 0.003$) and had a higher pre-vaccine seizure frequency ($p = 0.009$) compared with patients in the STABLE-group. Drug-resistant epilepsy was also associated with seizure worsening ($p = 0.01$). One-year pre-vaccination seizure frequency pattern demonstrated that patients in the WORSE-group had a higher frequency pattern ($p < 0.001$). Multivariate analysis of the vaccinated group showed that only the seizure frequency pattern (confidence interval [CI] = 1.257-2.028; $p < 0.001$) was significantly associated with seizure worsening. Conclusion In our cohort of vaccinated PwE, only a little percentage had a transient short-term increase of seizure frequency. The present study demonstrates that COVID-19 vaccines have a good safety and tolerability profile in the short term in PwE.
C1 [Romozzi, Marina; Rollo, Eleonora; Quintieri, Paolo; Servidei, Serenella; Calabresi, Paolo; Vollono, Catello] Univ Cattolica Sacro Cuore, Dipartimento Univ Neurosci, Rome, Italy.

[Romozzi, Marina; Rollo, Eleonora; Calabresi, Paolo] Fdn Policlin Univ Agostino Gemelli IRCCS, Neurol, Dipartimento Sci Invecchiamento Neurol Ortoped &, Rome, Italy.

[Dono, Fedele; Evangelista, Giacomo; Consoli, Stefano; Veleno, Luigi; Anzellotti, Francesca] G DAnnunzio Univ Chieti Pescara, Dipartimento Neurosci Imaging & Sci Clin, Chieti, Italy.

[Dono, Fedele] Univ G dAnnunzio, Ctr Adv Studies & Technol CAST, Behav Neurol Unit, Chieti, Italy.

[Dono, Fedele] Univ G dAnnunzio, Ctr Adv Studies & Technol CAST, Mol Neurol Unit, Chieti, Italy.

[Calvello, Carmen; Costa, Cinzia] Univ Perugia, Osped S Maria Misericordia, Dipartimento Med & Chirurg, Sez Neurol, Perugia, Italy.

[Servidei, Serenella; Vollono, Catello] Fdn Policlin Univ Agostino Gemelli IRCCS, Neurofisiopatol, Dipartimento Sci Invecchiamento Neurol Ortoped &, Rome, Italy.

C3 Catholic University of the Sacred Heart; IRCCS Policlinico Gemelli; Catholic University of the Sacred Heart; IRCCS Policlinico Gemelli; G d'Annunzio University of Chieti-Pescara; G d'Annunzio University of Chieti-Pescara; G d'Annunzio University of Chieti-Pescara; Hospital Santa Maria della Misericordia; University of Perugia; Catholic University of the Sacred Heart; IRCCS Policlinico Gemelli

RP Dono, F (通讯作者), G DAnnunzio Univ Chieti Pescara, Dipartimento Neurosci Imaging & Sci Clin, Chieti, Italy.; Dono, F (通讯作者), Univ G dAnnunzio, Ctr Adv Studies & Technol CAST, Behav Neurol Unit, Chieti, Italy.; Dono, F (通讯作者), Univ G dAnnunzio, Ctr Adv Studies & Technol CAST, Mol Neurol Unit, Chieti, Italy.

EM fedele.dono@unich.it

TC 1

Z9 1

PD AUG

PY 2022

VL 43
IS 8
BP 4627
EP 4634
DI 10.1007/s10072-022-06100-0
EA MAY 2022
WC Clinical Neurology; Neurosciences
ER

PT J
AU Prelack, M
Fridinger, S
Gonzalez, AK
Kaufman, MC
Xian, J
Galer, PD
Craig, S
Abend, NS
Helbig, I
AF Prelack, Marisa
Fridinger, Sara
Gonzalez, Alexander K.
Kaufman, Michael C.
Xian, Julie
Galer, Peter D.
Craig, Sansanee
Abend, Nicholas S.
Helbig, Ingo
CA CHOP Neurosci Ctr
VOC Res Grp
TI Visits of concern in child neurology telemedicine
SO DEVELOPMENTAL MEDICINE AND CHILD NEUROLOGY
DT Article; Early Access
DE telemedicine; epilepsy; COVID-19
ID HEALTH
AB Aim To characterize child neurology telemedicine visits flagged as requiring in-person evaluation during the COVID-19 pandemic. Method We analyzed 7130 audio-video telemedicine visits between March and November 2020. Visits of concern (VOCs) were defined as telemedicine visits where the clinical scenario necessitated in-person follow-up evaluation sooner than if the visit had been conducted in-person. Results VOCs occurred in 5% (333/7130) of visits for 292 individuals (148 females, 144 males). Providers noted technical challenges more often in VOCs (40%; 133/333) than visits without concern (non-VOCs) (28%; 1922/6797) ($p < 0.05$). The median age was younger in VOCs (9 years 3 months, interquartile range [IQR] 2 years 0 months-14 years 3 months) than non-VOCs (11 years 3 months, IQR 5 years 10 months-15 years 10 months) ($p < 0.05$). Median household income was lower for patients with VOCs ($74 K, IQR $55 K-$97 K) compared to non-VOCs ($80 K, IQR $61 K-$100 K) ($p < 0.05$). Compared with all other race

categories, families who self-identified as Black were more likely to have a VOC (odds ratio 1.53, 95% confidence interval 1.21-2.06). Epilepsy and headache represented the highest percentages of VOCs, while neuromuscular disorders and developmental delay had a higher proportion of VOCs than other neurological disorders. Interpretation These findings suggest that telemedicine is an effective platform for most child neurology visits. Younger children and those with neuromuscular disorders or developmental delays are more likely to require in-person evaluation.
C1 [Prelack, Marisa; Fridinger, Sara; Gonzalez, Alexander K.; Kaufman, Michael C.; Xian, Julie; Galer, Peter D.; Abend, Nicholas S.; Helbig, Ingo] Childrens Hosp Philadelphia, Div Neurol, Philadelphia, PA 19104 USA.
[Prelack, Marisa; Fridinger, Sara; Abend, Nicholas S.; Helbig, Ingo] Univ Penn, Dept Neurol, Perelman Sch Med, Philadelphia, PA 19104 USA.
[Gonzalez, Alexander K.; Kaufman, Michael C.; Xian, Julie; Galer, Peter D.; Craig, Sansanee; Helbig, Ingo] Childrens Hosp Philadelphia, Dept Biomed & Hlth Informat, Philadelphia, PA 19104 USA.
[Gonzalez, Alexander K.; Kaufman, Michael C.; Xian, Julie; Galer, Peter D.; Abend, Nicholas S.; Helbig, Ingo] Childrens Hosp Philadelphia, Epilepsy NeuroGenet Initiat, Philadelphia, PA 19104 USA.
[Abend, Nicholas S.] Univ Penn, Dept Biostat Epidemiol & Informat, Perelman Sch Med, Philadelphia, PA 19104 USA.
[Abend, Nicholas S.] Univ Penn, Dept Anesthesia & Crit Care, Perelman Sch Med, Philadelphia, PA 19104 USA.
C3 University of Pennsylvania; Childrens Hospital of Philadelphia; University of Pennsylvania; University of Pennsylvania; Childrens Hospital of Philadelphia; University of Pennsylvania; Childrens Hospital of Philadelphia; University of Pennsylvania; University of Pennsylvania
RP Helbig, I (通讯作者), Univ Penn, Childrens Hosp Philadelphia, Perelman Sch Med, Div Neurol, Philadelphia, PA 19104 USA.
EM helbigi@chop.edu
TC 1
Z9 1
DI 10.1111/dmcn.15256
EA MAY 2022
WC Clinical Neurology; Pediatrics
ER

PT J
AU Loescher, W
Howe, CL
AF Loescher, Wolfgang
Howe, Charles L.
TI Molecular Mechanisms in the Genesis of Seizures and Epilepsy Associated With Viral Infection
SO FRONTIERS IN MOLECULAR NEUROSCIENCE
DT Review
DE blood-brain barrier; neuroinflammation; hippocampal damage; herpesviruses; SARS-CoV-2; flaviruses; picornaviruses; status epilepticus

ID FEBRILE STATUS EPILEPTICUS; TEMPORAL-LOBE EPILEPSY; CENTRAL-NERVOUS-SYSTEM; THEILERS VIRUS-INFECTION; IONOTROPIC GLUTAMATE RECEPTORS; HIPPOCAMPAL NEURON MORPHOLOGY; ENTEROVIRUS 71 INFECTION; BRAIN-BARRIER BREAKDOWN; DRUG-RESISTANT EPILEPSY; LA-CROSSE ENCEPHALITIS

AB Seizures are a common presenting symptom during viral infections of the central nervous system (CNS) and can occur during the initial phase of infection ("early" or acute symptomatic seizures), after recovery ("late" or spontaneous seizures, indicating the development of acquired epilepsy), or both. The development of acute and delayed seizures may have shared as well as unique pathogenic mechanisms and prognostic implications. Based on an extensive review of the literature, we present an overview of viruses that are associated with early and late seizures in humans. We then describe potential pathophysiologic mechanisms underlying ictogenesis and epileptogenesis, including routes of neuroinvasion, viral control and clearance, systemic inflammation, alterations of the blood-brain barrier, neuroinflammation, and inflammation-induced molecular reorganization of synapses and neural circuits. We provide clinical and animal model findings to highlight commonalities and differences in these processes across various neurotropic or neuropathogenic viruses, including herpesviruses, SARS-CoV-2, flaviviruses, and picornaviruses. In addition, we extensively review the literature regarding Theiler's murine encephalomyelitis virus (TMEV). This picornavirus, although not pathogenic for humans, is possibly the best-characterized model for understanding the molecular mechanisms that drive seizures, epilepsy, and hippocampal damage during viral infection. An enhanced understanding of these mechanisms derived from the TMEV model may lead to novel therapeutic interventions that interfere with ictogenesis and epileptogenesis, even within non-infectious contexts.

C1 [Loescher, Wolfgang] Univ Vet Med, Dept Pharmacol Toxicol & Pharm, Hannover, Germany.
[Loescher, Wolfgang] Ctr Syst Neurosci, Hannover, Germany.
[Howe, Charles L.] Mayo Clin, Dept Neurol, Div Expt Neurol, Rochester, MN USA.
[Howe, Charles L.] Mayo Clin, Ctr Multiple Sclerosis & Autoimmune Neurol, Rochester, MN USA.
C3 University of Veterinary Medicine Hannover, Foundation; Mayo Clinic; Mayo Clinic
RP Loescher, W (通讯作者), Univ Vet Med, Dept Pharmacol Toxicol & Pharm, Hannover, Germany.; Loescher, W (通讯作者), Ctr Syst Neurosci, Hannover, Germany.
EM wolfgang.loescher@tiho-hannover.de

TC 1
Z9 1
PD MAY 9
PY 2022
VL 15
AR 870868
DI 10.3389/fnmol.2022.870868
WC Neurosciences
ER

PT J
AU Yoo, J

Kim, JH
Jeon, J
Kim, J
Song, TJ
AF Yoo, Joonsang
Kim, Jee Hyun
Jeon, Jimin
Kim, Jinkwon
Song, Tae-Jin
TI Risk of COVID-19 Infection and of Severe Complications Among People With Epilepsy A Nationwide Cohort Study
SO NEUROLOGY
DT Article
DE Risk; COVID-19; Severe Complications; Epilepsy
ID COMORBIDITY
AB Background and Objectives
The goal of this work was to evaluate whether patients with epilepsy were more susceptible to coronavirus disease 2019 (COVID-19) infection and at greater risk of severe complications when infected with COVID-19 compared with patients without epilepsy.
Methods
Weincluded participants who underwent at least 1 severe acute respiratory syndrome coronavirus 2 real-time reverse-transcription PCR test between January 1 and June 4, 2020, from the Korean nationwideCOVID-19 dataset. Epilepsy was defined according to the presence of diagnostic code in health claims data before the COVID-19 diagnosis. To investigate the association between epilepsy and the susceptibility for or severe complications of COVID-19, a 1:6 ratio propensity score matching (PSM) and logistic regression analysis were performed. Severe complications withCOVID-19 infection were defined as a composite of the incidence ofmechanical ventilation, intensive care unit admission, and death within 2 months after COVID-19 diagnosis.
Results
Among 212,678 study participants who underwent a COVID-19 test, 3,919 (1.8%) had a history of epilepsy. After PSM, there was no significant difference in COVID-19 PCR positivity according to epilepsy history (odds ratio [OR] 0.86, 95% CI 0.67-1.11). Of the 7,713 individuals with confirmed COVID-19 infection, 72 (0.9%) had a history of epilepsy. Among the patients with COVID-19, severe complications occurred in 444 (5.8%) individuals. After PSM, the presence of epilepsy was associated with the occurrence of severe complications after COVID-19 infection (OR 2.05, 95% CI 1.04-4.04). Mortality after COVID-19 infection did not differ according to the presence of epilepsy history (OR 1.55, 95% CI 0.65-3.70).
Discussion
The presence of epilepsy was not associated with increased susceptibility to COVID-19 infection or mortality related to the infection. However, there was an increased risk of severe complications with COVID-19 in patients with epilepsy; therefore, careful management and monitoring may be necessary.
C1 [Yoo, Joonsang; Jeon, Jimin; Kim, Jinkwon] Yonsei Univ, Coll Med, Yongin Severance Hosp, Dept Neurol, Seoul, South Korea.

[Kim, Jee Hyun; Song, Tae-Jin] Ewha Womans Univ, Dept Neurol, Seoul Hosp, Coll Med, Seoul, South Korea.
C3 Yonsei University; Yonsei University Health System; Ewha Womans University
RP Kim, J (通讯作者), Yonsei Univ, Coll Med, Yongin Severance Hosp, Dept Neurol, Seoul, South Korea.
EM antithrombus@gmail.com
TC 3
Z9 3
PD MAY 10
PY 2022
VL 98
IS 19
BP E1886
EP E1892
DI 10.1212/WNL.0000000000200195
WC Clinical Neurology
ER

PT J
AU Ahrens, SM
Ostendorf, AP
Lado, FA
Arnold, ST
Bai, SS
Bensalem-Owen, MK
Chapman, KE
Clarke, DF
Eisner, M
Fountain, NB
Gray, JM
Gunduz, MT
Hopp, JL
Riker, E
Schuele, SU
Small, B
Herman, ST
AF Ahrens, Stephanie M.
Ostendorf, Adam P.
Lado, Fred Alexander
Arnold, Susan T.
Bai, Shasha
Bensalem-Owen, Meriem K.
Chapman, Kevin E.
Clarke, Dave F.
Eisner, Mariah
Fountain, Nathan B.

Gray, Johanna M.
Gunduz, Muhammed Talha
Hopp, Jennifer L.
Riker, Ellen
Schuele, Stephan U.
Small, Barbara
Herman, Susan T.
TI Impact of the COVID-19 Pandemic on Epilepsy Center Practice in the United States
SO NEUROLOGY
DT Article
DE Impact; COVID-19; Epilepsy
ID SURGERY; TELEMEDICINE; CARE
AB Background and Objectives
Persons with epilepsy, especially those with drug resistant epilepsy (DRE), may benefit from inpatient services such as admission to the epilepsy monitoring unit (EMU) and epilepsy surgery. The COVID-19 pandemic caused reductions in these services within the US during 2020. This article highlights changes in resources, admissions, and procedures among epilepsy centers accredited by the National Association of Epilepsy Centers (NAEC).
Methods
We compared data reported in 2019, prior to the COVID-19 pandemic, and 2020 from all 260 level 3 and level 4 NAEC accredited epilepsy centers. Data were described using frequency for categorical variables and median for continuous variables and were analyzed by center level, center population category, and geographical location. Qualitative responses from center directors to questions regarding the impact from COVID-19 were summarized utilizing thematic analysis. Responses from the NAEC center annual reports as well as a supplemental COVID-19 survey were included.
Results
EMU admissions declined 23% (-21,515) in 2020, with largest median reductions in level 3 centers [-55 admissions (-44%)] and adult centers [-57 admissions (-39%)]. The drop in admissions was more substantial in the East North Central, East South Central, Mid Atlantic, and New England US Census divisions. Survey respondents attributed reduced admissions to re-assigning EMU beds, restrictions on elective admissions, reduced staffing, and patient reluctance for elective admission. Treatment surgeries declined by 371 cases (5.7%), with the largest reduction occurring in VNS implantations [-486 cases (-19%)] and temporal lobectomies [-227 cases (-16%)]. All other procedure volumes increased, including a 35% (54 cases) increase in corpus callosotomies.
Discussion
In the US, access to care for persons with epilepsy declined during the COVID-19 pandemic in 2020. Adult patients, those relying on level 3 centers for care, and many persons in the eastern half of the US were most affected.
C1 [Ahrens, Stephanie M.; Ostendorf, Adam P.; Gunduz, Muhammed Talha] Nationwide Childrens Hosp, Dept Pediat, Columbus, OH 43205 USA.
[Ahrens, Stephanie M.; Ostendorf, Adam P.; Gunduz, Muhammed Talha] Ohio State Univ, Columbus, OH 43210 USA.
[Lado, Fred Alexander] Hofstra Northwell Comprehens Epilepsy Ctr, Dept Neurol, Great

Neck, NY USA.
[Arnold, Susan T.] Childrens Med Ctr, Dept Pediat, Dallas, TX 75235 USA.
[Bai, Shasha] Emory Univ, Pediat Biostat Core, Sch Med, Atlanta, GA 30322 USA.
[Bensalem-Owen, Meriem K.] Univ Kentucky, Dept Neurol, Lexington, KY 40536 USA.
[Chapman, Kevin E.] Univ Arizona, Dept Child Hlth, Coll Med, Tucson, AZ 85721 USA.
[Chapman, Kevin E.] Phoenix Childrens Hosp, Barrow Neurol Inst, Phoenix, AZ USA.
[Clarke, Dave F.] Univ Texas Austin, Dell Med Sch, Dept Neurol, Div Pediat Neurol, Austin, TX 78712 USA.
[Eisner, Mariah] Nationwide Childrens Hosp, Biostat Resource, Columbus, OH USA.
[Fountain, Nathan B.] Univ Virginia, Comprehens Epilepsy Program, Dept Neurol, Charlottesville, VA USA.
[Gray, Johanna M.; Riker, Ellen; Small, Barbara] Natl Assoc Epilepsy Ctr, Washington, DC USA.
[Hopp, Jennifer L.] Univ Maryland, Med Ctr, Baltimore, MD 21201 USA.
[Schuele, Stephan U.] Northwestern Univ, Feinberg Sch Med, Ken & Ruth Davee Dept Neurol, Chicago, IL 60611 USA.
[Herman, Susan T.] Barrow Neurol Inst, Dept Neurol, Phoenix, AZ 85013 USA.
C3 Ohio State University; Ohio State University; Emory University;
University of Kentucky; University of Arizona; Barrow Neurological
Institute; Phoenix Children's Hospital; University of Texas System;
University of Texas Austin; Ohio State University; University of
Virginia; University System of Maryland; University of Maryland
Baltimore; Northwestern University; Feinberg School of Medicine; Barrow
Neurological Institute
RP Ahrens, SM (通讯作者), Nationwide Childrens Hosp, Dept Pediat, Columbus, OH 43205 USA.; Ahrens, SM (通讯作者), Ohio State Univ, Columbus, OH 43210 USA.
EM stephanie.ahrens@nationwidechildrens.org
TC 0
Z9 0
PD MAY 10
PY 2022
VL 98
IS 19
BP E1893
EP E1901
DI 10.1212/WNL.0000000000200285
WC Clinical Neurology
ER

PT J
AU Berg, AT
Jobst, B
AF Berg, Anne T.
Jobst, Barbara
TI Epilepsy and COVID-19's Double-Edged Sword More Severe Disease and
Delayed Epilepsy Care
SO NEUROLOGY

DT Editorial Material
DE Epilepsy; COVID-19; Epilepsy care
C1 [Berg, Anne T.] Northwestern Univ, Feinberg Sch Med, Dept Neurol, Chicago, IL 60611 USA.
[Jobst, Barbara] Geisel Sch Med Dartmouth, Dept Neurol, Hanover, NH USA.
C3 Northwestern University; Feinberg School of Medicine; Dartmouth College
RP Berg, AT (通讯作者), Northwestern Univ, Feinberg Sch Med, Dept Neurol, Chicago, IL 60611 USA.
EM a-berg@northwestern.edu
TC 0
Z9 0
PD MAY 10
PY 2022
VL 98
IS 19
BP 779
EP 780
DI 10.1212/WNL.0000000000200367
WC Clinical Neurology
ER

PT J
AU Valencia, I
Berg, AT
Hirsch, LJ
Lopez, MR
Melmed, K
Rosengard, JL
Tatum, WO
Jobst, BC
AF Valencia, Ignacio
Berg, Anne T.
Hirsch, Lawrence J.
Lopez, Maria Raquel
Melmed, Kara
Rosengard, Jillian L.
Tatum, William O.
Jobst, Barbara C.
TI Epilepsy and COVID 2021
SO EPILEPSY CURRENTS
DT Article; Early Access
DE COVID-19; epilepsy
ID SEIZURE SEMIOLOGY; DIAGNOSIS; VIDEOS
AB Coronavirus 19 (COVID-19) has infected over 400 million people worldwide. Although COVID-19 causes predominantly respiratory symptoms, it can affect other organs including the brain, producing neurological symptoms. People with epilepsy (PWE) have been particularly impacted during the pandemic with decreased access to care, increased

stress, and worsening seizures in up to 22% of them probably due to multiple factors. COVID-19 vaccines were produced in a record short time and have yielded outstanding protection with very rare serious side effects. Studies have found that COVID-19 vaccination does not increase seizures in the majority of PWE. COVID-19 does not produce a pathognomonic EEG or seizure phenotype, but rather 1 that can be seen in other types of encephalopathy. COVID-19 infection and its complications can lead to seizures, status epilepticus and post-COVID inflammatory syndrome with potential multi-organ damage in people without pre-existing epilepsy. The lack of access to care during the pandemic has forced patients and doctors to rapidly implement telemedicine. The use of phone videos and smart telemedicine are helping to treat patients during this pandemic and are becoming standard of care. Investment in infrastructure is important to make sure patients can have access to care even during a pandemic.
C1 [Valencia, Ignacio] Drexel Univ, Dept Pediat, Coll Med, St Christophers Hosp Children,Sect Neurol, Philadelphia, PA 19134 USA.
[Berg, Anne T.] COMBINEDBrain, Brentwood, TN USA.
[Berg, Anne T.] Northwestern Univ, Feinberg Sch Med, Chicago, IL 60611 USA.
[Hirsch, Lawrence J.] Yale Univ, Sch Med, Dept Neurol, Comprehens Epilepsy Ctr, New Haven, CT 06510 USA.
[Lopez, Maria Raquel] Univ Miami, Miller Sch Med, Dept Neurol, Epilepsy Div, Miami, FL 33136 USA.
[Melmed, Kara] NYU, Dept Neurol, Langone Med Ctr, Div Neurocrit Care, New York, NY 10016 USA.
[Melmed, Kara] NYU, Dept Neurosurg, Langone Med Ctr, Div Neurocrit Care, 550 1St Ave, New York, NY 10016 USA.
[Rosengard, Jillian L.] Montefiore Med Ctr, Albert Einstein Coll Med, Saul R Korey Dept Neurol, Bronx, NY 10467 USA.
[Tatum, William O.] Mayo Clin, Dept Neurol, Jacksonville, FL 32224 USA.
[Jobst, Barbara C.] Dartmouth Hlth, Geisel Sch Med, Hanover, NH USA.
C3 Drexel University; Northwestern University; Feinberg School of Medicine;
Yale University; University of Miami; New York University; NYU Langone
Medical Center; New York University; NYU Langone Medical Center;
Montefiore Medical Center; Yeshiva University; Albert Einstein College
of Medicine; Mayo Clinic; Dartmouth College
RP Valencia, I (通讯作者), Drexel Univ, Dept Pediat, Coll Med, St Christophers Hosp Children,Sect Neurol, Philadelphia, PA 19134 USA.
EM lgnacio.valencia@towerhealth.org
TC 0
Z9 0
AR 15357597221101268
DI 10.1177/15357597221101268
EA MAY 2022
WC Clinical Neurology
ER

PT J
AU Thongsing, A
Eizadkhah, D

Fields, C
Ballaban-Gil, K
AF Thongsing, Apirada
Eizadkhah, Donya
Fields, Cheryl
Ballaban-Gil, Karen
TI Provoked seizures and status epilepticus in a pediatric population with COVID-19 disease
SO EPILEPSIA
DT Article
DE COVID-19; febrile seizure; status epilepticus; provoked seizure; children
ID INFECTION
AB We are reporting 16 pediatric patients (ages 0-18-years-old) who presented to our urban hospital emergency room with seizures and coronavirus disease 2019 (COVID-19) during the surge of the Omicron variant. There was an increased number of pediatric patients with seizures and COVID-19 during this period as compared to prior COVID-19 surges. The 16 patients ranged in age from 3 months to 12 years of age. Five of the 16 patients (31%) had a prior history of epilepsy. Eight patients (50%) presented in status epilepticus, and in six patients (38%) the seizures appeared to have focal features. Fourteen patients (88%) presented with a complex provoked seizure defined as exhibiting either focality, seizure >5 min in length, or more than one seizure in 24 h. We suggest that in the pediatric population, when compared to prior variants of severe acute respiratory syndrome coronavirus 2 (SARS-CoV-2), the Omicron variant is more likely to be associated with neurologic symptoms, including complex provoked seizures.
C1 [Ballaban-Gil, Karen] Montefiore Med Ctr, Dept Neurol, Bronx, NY USA.
Montefiore Med Ctr, Dept Pediat, Bronx, NY USA.
Albert Einstein Coll Med, Bronx, NY 10467 USA.
C3 Montefiore Medical Center; Yeshiva University; Albert Einstein College of Medicine; Montefiore Medical Center; Yeshiva University; Albert Einstein College of Medicine; Yeshiva University; Albert Einstein College of Medicine
RP Ballaban-Gil, K (通讯作者), Albert Einstein Coll Med, Div Child Neurol, Saul R Korey Dept Neurol, Bronx, NY 10461 USA.; Ballaban-Gil, K (通讯作者), Montefiore Med Ctr, 111 E 210th St, Bronx, NY 10467 USA.
EM kballaba@montefiore.org
TC 0
Z9 0
PD AUG
PY 2022
VL 63
IS 8
BP E86
EP E91
DI 10.1111/epi.17293
EA MAY 2022
WC Clinical Neurology

ER

PT J
AU Streltzov, NA
Schmidt, SS
Schommer, LM
Zhao, W
Tosteson, TD
Mazanec, MT
Kiriakopoulos, ET
Chu, F
Henninger, HL
Nagle, K
Roth, RM
Jobst, B
AF Streltzov, Nicholas A.
Schmidt, Samantha S.
Schommer, Lindsay M.
Zhao, Wenyan
Tosteson, Tor D.
Mazanec, Morgan T.
Kiriakopoulos, Elaine T.
Chu, Felicia
Henninger, Heidi L.
Nagle, Keith
Roth, Robert M.
Jobst, Barbara
TI Effectiveness of a Self-Management Program to Improve Cognition and Quality of Life in Epilepsy A Pragmatic, Randomized, Multicenter Trial
SO NEUROLOGY
DT Article
DE Effectiveness; Self-Management; Management; Quality of Life; Epilepsy
ID ADULT COGNITION; HEALTH; INTERVENTION; DEPRESSION; MEMORY; COST; COMORBIDITIES; METAANALYSIS; IMPAIRMENT; TELEPHONE
AB Background and Objectives
We conducted a multisite, pragmatic replication trial at 4 New England epilepsy centers to determine the effectiveness of Home-Based Self-Management and Cognitive Training Changes Lives (HOBSCOTCH) in a real-world setting and to assess feasibility of a virtual intervention.
Methods
HOBSCOTCH is an 8-session intervention addressing cognitive impairment and quality of life (QoL) for people with epilepsy (PWE). Participants were recruited from epilepsy centers in 4 states and block-randomized into the following groups: in-person HOBSCOTCH (H-IP), virtual HOBSCOTCH (H-V), and waitlist control. Outcome measures were assessed for all groups at baseline, 3 months, and 6 months; intervention groups received long-term follow-up at 9 and 12 months.
Results

A total of 108 participants were recruited, of whom 85 were included in this analysis (age at baseline 47.5 +/- 11.5 years; 68% female). Participants completing the in-person intervention (H-IP) had a 12.4-point improvement in QoL score compared with controls ($p < 0.001$). Pairwise comparisons found a 6.2-point treatment effect for subjective cognition in the H-IP group ($p < 0.001$). There were no meaningful group differences in objective cognition or health care utilization at any time points and the treatment effect for QoL diminished by 6 months. The virtual intervention demonstrated feasibility but did not significantly improve outcomes compared with controls. Within-group analysis found improvements in QoL for both H-V and H-IP.
Discussion
This study replicated the effectiveness of the HOBSCOTCH program in improving QoL for PWE. The study was conducted prior to the COVID-19 pandemic, but the distance-delivered intervention may be particularly well-suited for the current environment. Future research will explore modifications designed to improve the efficacy of H-V and the sustainability of HOBSCOTCH's treatment effect.
Classification of Evidence
This study provides Class III evidence that in-person HOBSCOTCH delivery improved subjective measures of cognition in persons with epilepsy.
C1 [Streltzov, Nicholas A.; Schmidt, Samantha S.; Schommer, Lindsay M.; Mazanec, Morgan T.; Kiriakopoulos, Elaine T.; Jobst, Barbara] Dartmouth Hitchcock Med Ctr, Dept Neurol, Lebanon, NH 03766 USA.
[Roth, Robert M.] Dartmouth Hitchcock Med Ctr, Dept Psychiat, Lebanon, NH 03766 USA.
[Schommer, Lindsay M.; Zhao, Wenyan; Tosteson, Tor D.; Kiriakopoulos, Elaine T.; Roth, Robert M.; Jobst, Barbara] Dartmouth Coll, Geisel Sch Med, Hanover, NH 03755 USA.
[Chu, Felicia] UMass Med Sch, Dept Neurol, Worcester, MA USA.
[Henninger, Heidi L.] Maine Med Ctr, Dept Neurol, Scarborough, ME USA.
[Nagle, Keith] Univ Vermont, Med Ctr, Dept Neurol, Burlington, VT USA.
C3 Dartmouth College; Dartmouth College; Dartmouth College; University of Massachusetts System; University of Massachusetts Worcester; Maine Medical Center; University of Vermont; University of Vermont Medical Center
RP Streltzov, NA (通讯作者), Dartmouth Hitchcock Med Ctr, Dept Neurol, Lebanon, NH 03766 USA.
EM nastreltzov@gmail.com
TC 1
Z9 1
PD MAY 24
PY 2022
VL 98
IS 21
BP E2174
EP E2184
DI 10.1212/WNL.0000000000200346
WC Clinical Neurology
ER

PT J

AU Sapkota, S
Caruso, E
Kobau, R
Radhakrishnan, L
Jobst, B
DeVies, J
Tian, N
Hogan, RE
Zack, MM
Pastula, DM
AF Sapkota, Sanjeeb
Caruso, Elise
Kobau, Rosemarie
Radhakrishnan, Lakshmi
Jobst, Barbara
DeVies, Jourdan
Tian, Niu
Hogan, R. Edward
Zack, Matthew M.
Pastula, Daniel M.
TI Seizure- or Epilepsy-Related Emergency Department Visits Before and During the COVID-19 Pandemic - United States, 2019-2021
SO MMWR-MORBIDITY AND MORTALITY WEEKLY REPORT
DT Article
DE COVID-19; Risk; hospitalization; death; epilepsy
C1 [Sapkota, Sanjeeb] CDC, Ctr Global Hlth, Off Director, Atlanta, GA 30333 USA.
[Caruso, Elise] CDC, Natl Ctr HIV AIDS Viral Hepatitis STD & TB Preven, Div TB Eliminat, Atlanta, GA 30333 USA.
[Kobau, Rosemarie; Tian, Niu; Zack, Matthew M.; Pastula, Daniel M.] CDC, Natl Ctr Chron Dis Prevent & Hlth Promot, Div Populat Hlth, Atlanta, GA 30333 USA.
[Radhakrishnan, Lakshmi] CDC, Ctr Surveillance Epidemiol & Lab Serv, Div Hlth Informat & Surveillance, Atlanta, GA 30333 USA.
[Jobst, Barbara] Univ Dartmouth, Geisel Sch Med, Hanover, NH USA.
[DeVies, Jourdan] ICF Int, Fairfax, VA USA.
[Hogan, R. Edward] Washington Univ, St Louis, MO 63110 USA.
[Pastula, Daniel M.] Univ Colorado, Sch Med, Aurora, CO USA.
[Pastula, Daniel M.] Colorado Sch Publ Hlth, Aurora, CO USA.
C3 Centers for Disease Control & Prevention - USA; Centers for Disease Control & Prevention - USA; Centers for Disease Control & Prevention - USA; Centers for Disease Control & Prevention - USA; Dartmouth College; Washington University (WUSTL); University of Colorado System; University of Colorado Anschutz Medical Campus; Colorado School of Public Health
RP Sapkota, S (通讯作者), CDC, Ctr Global Hlth, Off Director, Atlanta, GA 30333 USA.
EM auu6@cdc.gov
TC 0
Z9 0
PD MAY 27

PY 2022
VL 71
IS 21
BP 703
EP 708
WC Public, Environmental & Occupational Health
ER

PT J
AU Al Jandale, O
   Almoshantaf, MB
   Qaddah, A
   Bakkour, A
   Ahmed, EMS
AF Al Jandale, Omar
   Almoshantaf, Mohammad Badr
   Qaddah, Aya
   Bakkour, Agyad
   Ahmed, Eman Mohammed sharif
TI Tonic non-colonic convulsions-status epilepticus- as the presenting
   complaint of COVID-19
SO ANNALS OF MEDICINE AND SURGERY
DT Article
DE COVID-19; Coronavirus; status epilepticus
AB Introduction: COVID19 might present as neurological symptoms including headaches, dizziness, anosmia, stroke, and loss of consciousness. Cases with severe COVID-19 are more likely to be complicated by neurological symptoms, but tonic non-colonic convulsion is still a rare presentation of COVID19 as an initial symptom. Case presentation: A 23-years old male presented to the ambulance with a complaint of loss of consciousness for more than 1 h and tonic convulsions without clonic movements. The investigations including computed tomography for the brain and chest and lumbar puncture were within normal range and the diagnostic workup concluded that COVID-19 is the cause of the status epilepticus. Discussion: Our case demonstrates a tonic non-clonic convulsion as a possible complication for COVID-19 infection as some respiratory viruses can cause neurological symptoms. After excluding the co-incidence of other pathological etiologies, we highly suspect that the seizures in our case are generated by COVID-19 infection. Conclusion: This case represents a rare case in the literature review which can increase the awareness of tonic non-clonic seizures and other neurological manifestations as the presenting symptom of the COVID19.
C1 [Al Jandale, Omar] Damascus Univ, Dept Cardiol, Damascus, Syria.
   [Almoshantaf, Mohammad Badr] Ibn Al Nafees Hosp, Dept Neurosurg, Damascus, Syria.
   [Qaddah, Aya] Damascus Univ, Dept Neurol, Damascus, Syria.
   [Bakkour, Agyad] Albaath Univ, Fac Med, Homs, Syria.
   [Ahmed, Eman Mohammed sharif] Nile Vally Univ, Atbra, Sudan.
RP Almoshantaf, MB (通讯作者), Ibn Al Nafees Hosp, Dept Neurosurg, Damascus, Syria.
EM baderalmoushantaf1995@gmail.com
TC 0

Z9 0
PD JUN
PY 2022
VL 78
AR 103744
DI 10.1016/j.amsu.2022.103744
WC Medicine, General & Internal
ER

PT J
AU Hatano, K
Fujimoto, A
Sato, K
Yamamoto, T
Enoki, H
AF Hatano, Keisuke
Fujimoto, Ayataka
Sato, Keishiro
Yamamoto, Takamichi
Enoki, Hideo
TI Omitting Hyperventilation in Electroencephalogram during the COVID-19 Pandemic May Reduce Interictal Epileptiform Discharges in Patients with Juvenile Myoclonic Epilepsy
SO BRAIN SCIENCES
DT Article
DE EEG; hyperventilation (HV); interictal epileptiform discharge (IED); COVID-19 epilepsy
ID CLINICAL-FEATURES; EEG; SEIZURES; CLASSIFICATION; EPIDEMIOLOGY; ACTIVATION
AB Background: To prevent the spread of coronavirus disease 2019 (COVID-19), hyperventilation (HV) activation has been avoided in electroencephalograms (EEGs) since April 2020. The influence of omitting HV in EEG on epilepsy diagnosis remains uncertain for patients with epilepsies other than child absence epilepsy. We hypothesized that EEGs with HV would show more interictal epileptiform discharges (IEDs) than EEGs without HV in patients with juvenile myoclonic epilepsy (JME). Methods: We reviewed the EEGs of seizure-free patients with JME who underwent EEG, both with and without HV, from January 2019 to October 2021, in our institution, and compared IEDs between EEG with and without HV. Results: This study analyzed 23 JME patients. The IED-positive rate was significantly higher in EEG with HV (65.2%) than in EEG without HV (34.8%, p = 0.016). The mean +/- standard deviation number of IEDs per minute was significantly larger during HV (1.61 +/- 2.25 x 10(-1)) than during non-activation of both first EEG (0.57 +/- 0.93 x 10(-1), p = 0.039) and second EEG (0.39 +/- 0.76 x 10(-1), p = 0.009). Conclusions: In JME patients, performing HV during EEG may increase IEDs and appears to facilitate the accurate diagnosis of epilepsy.
C1 [Hatano, Keisuke; Fujimoto, Ayataka; Sato, Keishiro; Yamamoto, Takamichi; Enoki, Hideo] Seirei Hamamatsu Gen Hosp, Comprehens Epilepsy Ctr, Naka Ku, 2-12-12 Sumiyoshi, Hamamatsu, Shizuoka 4308558, Japan.

[Fujimoto, Ayataka; Yamamoto, Takamichi] Seirei Hamamatsu Gen Hosp, Dept Neurosurg, Naka Ku, 2-12-12 Sumiyoshi, Hamamatsu, Shizuoka 4308558, Japan.
RP Fujimoto, A (通讯作者), Seirei Hamamatsu Gen Hosp, Comprehens Epilepsy Ctr, Naka Ku, 2-12-12 Sumiyoshi, Hamamatsu, Shizuoka 4308558, Japan.; Fujimoto, A (通讯作者), Seirei Hamamatsu Gen Hosp, Dept Neurosurg, Naka Ku, 2-12-12 Sumiyoshi, Hamamatsu, Shizuoka 4308558, Japan.
EM hatakenosuke@gmail.com; afujimotoscienceacademy@gmail.com;
k-sato@sis.seirei.or.jp; taka-yamamd@sis.seirei.or.jp;
enokih.neuropediatr@gmail.com
TC 0
Z9 0
PD JUN
PY 2022
VL 12
IS 6
AR 769
DI 10.3390/brainsci12060769
WC Neurosciences
ER

PT J
AU Yeni, K
Tulek, Z
Ozer, A
Cavusoglu, A
Inan, GS
Baykan, B
Bebek, N
AF Yeni, Kubra
Tulek, Zeliha
Ozer, Arif
Cavusoglu, Aysel
Inan, Gorkem Sirin
Baykan, Betul
Bebek, Nerses
TI COVID-19 pandemic in Istanbul: Seizure frequency and psychosocial outcomes in patients with epilepsy
SO NEUROLOGY ASIA
DT Article
DE Epilepsy; COVID-19; outcome; psychosocial outcomes; mental health
ID QUALITY-OF-LIFE; ANXIETY; PEOPLE; DEPRESSION; IMPACT
AB Objective: The aim of this study was to examine seizure frequency and psychosocial outcomes in patients with epilepsy (PwE) during the COVID-19 pandemic. Methods: This cross-sectional casecontrol study included 319 adult PwE and 327 healthy controls (HC). The data were collected using a (BDI-II), and the Pittsburg Sleep Quality Index (PSQI). Results: Seventy-four (23.1%) patients had an increase in seizure frequency during the pandemic. The mean age of PWE with increased seizure frequency (ISF) was lower

(32.8??9.3) than PWE with unchanged seizure frequency (USF) (37.0??11.4). When psychosocial impacts of the pandemic were compared between the groups, the ISF group was psychologically affected more than the USF and HC groups. WAQ and BDI-II scores were higher in ISF than USF and higher in USF than HC group. Also, the analysis revealed that the seizure frequency during the pandemic was almost similar before the pandemic. However, only those who had frequent seizures before the pandemic had worsened seizure frequency during the pandemic. The analysis showed that factors such as having trouble in drug supply, having more than 4-6 seizures in a year, worrying about seizure intervention, and being depressed, were related to being psychologically affected by the pandemic. Conclusion: It was observed that the psychosocial outcomes during the pandemic were worse in PwE than healthy controls, especially in those with increased seizure frequency. Therefore, it is essential to increase the medical and psychosocial support to PwE during the pandemic.
C1 [Yeni, Kubra] Ondokuz Mayis Univ, Fac Hlth Sci, OMU Kurupelit Kampusu, TR-55200 Samsun, Turkey.

[Tulek, Zeliha] Istanbul Univ Cerrahpasa, Florence Nightingale Fac Nursing, Istanbul, Turkey.

[Ozer, Arif] Hacettepe Univ, Fac Educ, Dept Guidance & Psychol Counseling, Ankara, Turkey.

[Cavusoglu, Aysel; Inan, Gorkem Sirin; Baykan, Betul; Bebek, Nerses] Istanbul Univ, Istanbul Fac Med, Dept Neurol, Istanbul, Turkey.

[Baykan, Betul; Bebek, Nerses] Istanbul Univ, Epilepsy Res Ctr, Istanbul, Turkey.

C3 Ondokuz Mayis University; Istanbul University - Cerrahpasa; Hacettepe University; Istanbul University; Istanbul University

RP Yeni, K (通讯作者), Ondokuz Mayis Univ, Fac Hlth Sci, OMU Kurupelit Kampusu, TR-55200 Samsun, Turkey.

EM akdagyeni@hotmail.com
TC 0

Z9 0

PD JUN

PY 2022

VL 27

IS 2

BP 291

EP 300

DI 10.54029/2022ycf

WC Clinical Neurology

ER

PT J

AU Kuroda, N

Kubota, T

Horinouchi, T

Ikegaya, N

Kitazawa, Y

Kodama, S

Kuramochi, I

   Matsubara, T
   Nagino, N
   Neshige, S
   Soga, T
   Takayama, Y
   Sone, D
AF Kuroda, Naoto
   Kubota, Takafumi
   Horinouchi, Toru
   Ikegaya, Naoki
   Kitazawa, Yu
   Kodama, Satoshi
   Kuramochi, Izumi
   Matsubara, Teppei
   Nagino, Naoto
   Neshige, Shuichiro
   Soga, Temma
   Takayama, Yutaro
   Sone, Daichi
CA IMPACT-J EPILEPSY In-depth Multict
TI Impact of COVID-19 pandemic on epilepsy care in Japan: A national-level multicenter retrospective cohort study
SO EPILEPSIA OPEN
DT Article
DE epilepsy; epilepsy care; neurology; Impact; SARS-CoV-2
ID DISPARITIES; DIAGNOSIS; CLAIMS; EEG
AB Objective The impact of the coronavirus disease 2019 (COVID-19) pandemic on epilepsy care across Japan was investigated by conducting a multicenter retrospective cohort study. Methods This study included monthly data on the frequency of (1) visits by outpatients with epilepsy, (2) outpatient electroencephalography (EEG) studies, (3) telemedicine for epilepsy, (4) admissions for epilepsy, (5) EEG monitoring, and (6) epilepsy surgery in epilepsy centers and clinics across Japan between January 2019 and December 2020. We defined the primary outcome as epilepsy center-specific monthly data divided by the 12-month average in 2019 for each facility. We determined whether the COVID-19 pandemic-related factors (such as year [2019 or 2020], COVID-19 cases in each prefecture in the previous month, and a state of emergency) were independently associated with these outcomes. Results In 2020, the frequency of outpatient EEG studies (-10.7%, P < .001) and cases with telemedicine (+2608%, P = .031) were affected. The number of COVID-19 cases was an independent associated factor for epilepsy admission (-3.75 x 10(-3)% per case, P < .001) and EEG monitoring (-3.81 x 10(-3)% per case, P = .004). Furthermore, a state of emergency was an independent factor associated with outpatient with epilepsy (-11.9%, P < .001), outpatient EEG (-32.3%, P < .001), telemedicine for epilepsy (+12,915%, P < .001), epilepsy admissions (-35.3%; P < .001), EEG monitoring (-24.7%: P < .001), and epilepsy surgery (-50.3%, P < .001). Significance We demonstrated the significant impact that the COVID-19 pandemic had on epilepsy care. These results support those of previous studies and clarify the effect size of each pandemic-related factor on epilepsy care.

C1 [Kuroda, Naoto; Kubota, Takafumi; Horinouchi, Toru; Ikegaya, Naoki; Kitazawa, Yu; Kodama, Satoshi; Kuramochi, Izumi; Matsubara, Teppei; Nagino, Naoto; Neshige, Shuichiro; Soga, Temma; Takayama, Yutaro; Sone, Daichi] Japan Young Epilepsy Sect YES Japan, Tokyo, Japan.

[Kuroda, Naoto] Wayne State Univ, Dept Pediat, 3901 Beaubien St, Detroit, MI 48201 USA.

[Kubota, Takafumi] Case Western Reserve Univ, Cleveland Med Ctr, Univ Hosp, Dept Neurol, Cleveland, OH 44106 USA.

[Horinouchi, Toru] Hokkaido Univ, Dept Psychiat & Neurol, Grad Sch Med, Sapporo, Hokkaido, Japan.

[Ikegaya, Naoki] Yokohama City Univ, Grad Sch Med, Dept Neurosurg, Yokohama, Kanagawa, Japan.

[Kitazawa, Yu] Yokohama City Univ, Dept Neurol & Stroke Med, Grad Sch Med, Yokohama, Kanagawa, Japan.

[Kodama, Satoshi] Univ Tokyo, Grad Sch Med, Dept Neurol, Tokyo, Japan.

[Kuramochi, Izumi] Saitama Med Univ, Saitama Med Ctr, Dept Psychiat, Saitama, Japan.

[Matsubara, Teppei] Massachusetts Gen Hosp, Athinoula A Martinos Ctr Biomed Imaging, Charlestown, MA USA.

[Nagino, Naoto] Tpilepsy Ctr, TMG Asaka Med Ctr, Saitama, Japan.

[Neshige, Shuichiro] Hiroshima Univ, Grad Sch Biomed & Hlth Sci, Dept Clin Neurosci & Therapeut, Hiroshima, Japan.

[Soga, Temma] Tohoku Univ, Dept Epileptol, Grad Sch Med, Sendai, Miyagi, Japan.

[Takayama, Yutaro] Natl Ctr Hosp, Natl Ctr Neurol & Psychiat, Dept Neurosurg, Tokyo, Japan.

[Sone, Daichi] UCL Inst Neurol, Dept Clin & Expt Epilepsy, London, England.

C3 Wayne State University; Case Western Reserve University; Case Western Reserve University Hospital; University Hospitals of Cleveland; US Department of Veterans Affairs; Veterans Health Administration (VHA); Louis Stokes Cleveland Veterans Affairs Medical Center; Hokkaido University; Yokohama City University; Yokohama City University; University of Tokyo; Saitama Medical University; Harvard University; Massachusetts General Hospital; Hiroshima University; Tohoku University; National Center for Neurology & Psychiatry - Japan; University of London; University College London

RP Kuroda, N (通讯作者), Wayne State Univ, Dept Pediat, 3901 Beaubien St, Detroit, MI 48201 USA.; Ikegaya, N (通讯作者), Yokohama City Univ, Grad Sch Med, Dept Neurosurg, Kanazawa Ku, 3-9 Fukuura, Yokohama, Kanagawa, Japan.

EM naoto.kuroda@wayne.edu; nikegaya@yokohama-cu.ac.jp

TC 0

Z9 0

PD SEP

PY 2022

VL 7

IS 3

BP 431

EP 441

DI 10.1002/epi4.12616

EA JUN 2022
WC Clinical Neurology; Neurosciences
ER

PT J
AU Madaan, P
Saini, L
Dhir, P
Vikas, S
Bhagwat, C
Goel, M
Soni, A
Sahu, JK
AF Madaan, Priyanka
Saini, Lokesh
Dhir, Pooja
Vikas, Sahil
Bhagwat, Chandana
Goel, Mallika
Soni, Akshita
Sahu, Jitendra Kumar
TI COVID-19 in Children with West Syndrome: An Ambispective Study
SO INDIAN JOURNAL OF PEDIATRICS
DT Article; Early Access
DE West Syndrome; Children; epilepsy; COVID-2019
ID INFANTILE SPASMS; MANAGEMENT
AB Objectives To study the course of West syndrome (WS) and coronavirus disease-19 (COVID-19) in children with WS who contracted SARS-CoV-2 infection. Methods This ambispective study was conducted at a tertiary-care center in North India between December 2020 and August 2021 after approval from the Institute Ethics Committee. Five children with WS, positive for COVID-19 based on RT-PCR, fulfilled the inclusion criteria. Results One child with COVID-19 during the first wave was retrospectively included while four children (of the 70 children screened) were prospectively enrolled. The median age at onset of epileptic spasms was 7 mo (2 boys), and that at presentation with COVID-19 was 18.5 mo. Three had underlying acquired structural etiology. Three were in remission following standard therapy, while two had ongoing spasms at the time of COVID-19 illness. During the illness, two of those in remission continued to be in remission while one child had a relapse. The children with ongoing epileptic spasms had variable course [one had persistent spasms and other had transient cessation lasting 3 wk from day 2 of COVID-19 illness, but electroencephalography (on day 8 of COVID-19 illness) continued to show hypsarrhythmia]. Fever was the most typical symptom (and sometimes the only symptom) of COVID-19, with a duration ranging from 1-8 d. Two children had moderate COVID-19 illness requiring hospitalization, while the rest had a mild illness. All the affected children had complete recovery from COVID-19. Conclusion The severity of COVID-19 illness in children with WS is often mild, while the subsequent course of WS is variable.
C1 [Madaan, Priyanka; Saini, Lokesh; Dhir, Pooja; Bhagwat, Chandana; Sahu, Jitendra

Kumar] Inst Med Educ & Res PGIMER, Pediat Neurol Unit, Dept Pediat, Chandigarh 160012, India.
[Vikas, Sahil; Goel, Mallika; Soni, Akshita] PGIMER, Dept Pediat, Chandigarh, India.
C3 Post Graduate Institute of Medical Education & Research (PGIMER), Chandigarh; Post Graduate Institute of Medical Education & Research (PGIMER), Chandigarh
RP Sahu, JK (通讯作者), Inst Med Educ & Res PGIMER, Pediat Neurol Unit, Dept Pediat, Chandigarh 160012, India.
EM jsh2003@gmail.com
TC 0
Z9 0
DI 10.1007/s12098-022-04201-4
EA JUN 2022
WC Pediatrics
ER

PT J
AU Milan, A
Salles, P
Pelayo, C
Uribe-San-Martin, R
AF Milan, Anna
Salles, Philippe
Pelayo, Carolina
Uribe-San-Martin, Reinaldo
TI Acute to Chronic Electro-Clinical Manifestations of Neuro-COVID and the Long-Haul Consequences in People With Epilepsy: A Review
SO CUREUS JOURNAL OF MEDICAL SCIENCE
DT Review
DE people with epilepsy; eeg; encephalitis; seizure; epilepsy; covid-19
ID NEUROLOGICAL MANIFESTATIONS; SARS-COV-2 INFECTION; PROGNOSTIC-FACTORS; ENCEPHALITIS; EEG; DIAGNOSIS; SEIZURES; SYSTEM
AB Severe acute respiratory syndrome coronavirus 2 (SARS-COV-2) infection can involve the central nervous system (CNS). Acute symptomatic seizures or epileptiform discharges have not been commonly reported in patients with altered mental status related to coronavirus disease 2019 (COVID-19) infection. However, long-term neurological symptoms have been reported after COVID-19 infection (i.e., brain fog, cognitive complaints, and confusion), suggesting chronic encephalopathy. People with epilepsy (PWE) have been specifically affected by the COVID-19 pandemic, with changes in their seizure frequency, quality of life, health care accessibility, and medication interactions. This narrative review highlights possible pathophysiological mechanisms of COVID-19 on the brain, related to short- and long-term epileptiform activity and the impact of this infection on PWE.
C1 [Milan, Anna] Liga Chilena La Epilepsia, Epilepsy, Santiago, Chile.
[Salles, Philippe] Ctr Trastornos Movimiento CETRAM, Movement Disorders, Santiago, Chile.
[Pelayo, Carolina] Pontificia Univ Catolica Chile, Neuroimmunol, Santiago, Chile.

[Uribe-San-Martin, Reinaldo] Pontificia Univ Catolica Chile, Epilepsy, Santiago, Chile.
C3 Pontificia Universidad Catolica de Chile; Pontificia Universidad
   Catolica de Chile
RP Milan, A (通讯作者), Liga Chilena La Epilepsia, Epilepsy, Santiago, Chile.
EM milan.anna@gmail.com
TC 0
Z9 0
PD JUN 16
PY 2022
VL 14
IS 6
AR e26020
DI 10.7759/cureus.26020
WC Medicine, General & Internal
ER

PT J
AU Sehlo, MG
   Mohamed, WS
   Youssef, UM
   Lotfi, SE
   El-deen, GMS
AF Sehlo, Mohammad Gamal
   Mohamed, Wafaa Samir
   Youssef, Usama Mahmoud
   Lotfi, Shrouk Esam
   El-deen, Ghada Mohamed Salah
TI Prevalence and determinants of anxiety in patients with epilepsy during
   COVID-19 pandemic
SO EGYPTIAN JOURNAL OF NEUROLOGY PSYCHIATRY AND NEUROSURGERY
DT Article
DE COVID-19; Epilepsy; Anxiety; risk factor
AB Background Epilepsy is one of the most frequent and serious brain disorders. The nature of the disorder and the unpredictability of seizures usually puts patients in a state of apprehension and anticipation, which creates a continuous condition of anxiety. COVID-19 pandemic has created a state of generalized anxiety all over the world. It is expected that patients with epilepsy (PWE) will suffer from more anxiety during the pandemic. This cross-sectional study was applied on 290 PWE. Data were collected by personal interview with each patient using GAD-7 scale for diagnosing anxiety and assessing its severity. We aimed to assess the prevalence of anxiety and to assess its risk factors in PWE during COVID-19 pandemic. Results We found that 52.4% of PWE suffered from anxiety. Not working, low financial status, fear of infection and death by COVID-19, fear of job loss, had job changes during pandemic, increased seizures rate during pandemic, increased ER visits, and lack of drug adherence during the pandemic, are significantly associated with increased risk of anxiety. Conclusions COVID-19 pandemic has a serious effect on the psychological and the physical wellbeing of PWE. There was

an increased rate of anxiety during COVID-19 pandemic in PWE with its subsequent burden on those patients. So, these patients are in a high need of care and support during the pandemic.
C1 [Sehlo, Mohammad Gamal; Youssef, Usama Mahmoud; El-deen, Ghada Mohamed Salah] Zagazig Univ, Fac Med, Psychiat Dept, POB 44519, Zagazig, Egypt.
[Mohamed, Wafaa Samir] Zagazig Univ, Fac Med, Neurol Dept, Zagazig, Egypt.
[Lotfi, Shrouk Esam] Neuropsychiat Resident Abbaseya Hosp Mental Illne, Cairo, Egypt.
C3 Egyptian Knowledge Bank (EKB); Zagazig University; Egyptian Knowledge Bank (EKB); Zagazig University
RP Sehlo, MG (通讯作者), Zagazig Univ, Fac Med, Psychiat Dept, POB 44519, Zagazig, Egypt.
EM sehlo68@gmail.com
TC 0
Z9 0
PD JUN 23
PY 2022
VL 58
IS 1
AR 78
DI 10.1186/s41983-022-00513-6
WC Neurosciences
ER

PT J
AU Lu, Q
Wang, YY
Wang, QH
Tang, LN
Yang, XY
Dun, S
Zou, LP
AF Lu, Qian
Wang, Yang-Yang
Wang, Qiu-Hong
Tang, Li-Na
Yang, Xiao-Yan
Dun, Shuo
Zou, Li-Ping
TI Safety of inactivated COVID-19 vaccine in tuberous sclerosis complex patients with epilepsy treated with rapamycin
SO SEIZURE-EUROPEAN JOURNAL OF EPILEPSY
DT Article
DE COVID-19; vaccine; Tuberous sclerosis complex; Epilepsy; Safety; Rapamycin
AB Purpose: To assess the safety of inactivated coronavirus 2019 disease (COVID-19) vaccine in tuberous sclerosis complex (TSC) patients with epilepsy.
Methods: All patients with epilepsy were selected from Efficacy and Safety of Sirolimus in Pediatric Patients with Tuberous Sclerosis (ESOSPIT) project and younger

than 17 years old. The patients were treated with mTOR inhibitors (rapamycin). A total of 44 patients who completed the two-dose inactivated COVID-19 vaccine between July 7, 2021, and January 1, 2022, were enrolled.
Results: The median age of seizure onset was 23 months. About two-thirds of patients have focal seizures. Thirty-three patients use antiseizure medications. The mean duration of rapamycin treatment was 55.59 +/- 18.42 months. Adverse reactions within 28 days after injection occurred in 11 patients (25%), all were under 12 years old. Injection site pain was the most reported event (20.45%), which was mild in severity and improved within one day. All patients had no seizure-related changes after vaccination.
Conclusion: This study shows that the inactivated COVID-19 vaccine was well tolerated and safe in TSC patients with epilepsy, as well as for those treated with mTOR inhibitors.
C1 [Lu, Qian; Wang, Yang-Yang; Wang, Qiu-Hong; Tang, Li-Na; Yang, Xiao-Yan; Dun, Shuo; Zou, Li-Ping] Chinese Peoples Liberat Army Gen Hosp, Med Ctr 1, Dept Pediat, Senior Dept Pediat, Beijing 100853, Peoples R China.
[Tang, Li-Na] Fosun Hlth, Beijing, Peoples R China.
[Zou, Li-Ping] Capital Med Univ, Ctr Brain Disorders Res, Beijing Inst Brain Disorders, Beijing, Peoples R China.
C3 Chinese People's Liberation Army General Hospital; Capital Medical
University
RP Zou, LP (通讯作者), Chinese Peoples Liberat Army Gen Hosp, Med Ctr 1, Dept Pediat, Senior Dept Pediat, Beijing 100853, Peoples R China.
EM zouliping21@hotmail.com
TC 1
Z9 1
PD JUL
PY 2022
VL 99
BP 71
EP 74
DI 10.1016/j.seizure.2022.05.010
WC Clinical Neurology; Neurosciences
ER

PT J
AU Dairbayeva, L
Kabi, A
AF Dairbayeva, L.
Kabi, A.
TI Epilepsy care during COVID-19 pandemic lockdown: how to get access to
Epilepsy Clinic
SO EUROPEAN JOURNAL OF NEUROLOGY
DT Meeting Abstract
DE COVID-19; Epilepsy care; lockdown
C1 [Dairbayeva, L.; Kabi, A.] Reg Neurol & Epilepsy Ctr, Alma Ata, Kazakhstan.
TC 0

Z9 0
PD JUL
PY 2022
VL 29
SU 1
MA EPO-551
BP 729
EP 729
WC Clinical Neurology; Neurosciences
ER

PT J
AU Vignatelli, L
   Muccioli, L
   Zenesini, C
   Taruffi, L
   Baccari, F
   Licchetta, L
   Mostacci, B
   Pasini, E
   Michelucci, R
   Tinuper, P
   Nonino, F
   Bisulli, F
AF Vignatelli, L.
   Muccioli, L.
   Zenesini, C.
   Taruffi, L.
   Baccari, F.
   Licchetta, L.
   Mostacci, B.
   Pasini, E.
   Michelucci, R.
   Tinuper, P.
   Nonino, F.
   Bisulli, F.
TI Risk of hospitalization and death for COVID-19 in persons with epilepsy:
   the EpiLink Bologna cohort, Italy
SO EUROPEAN JOURNAL OF NEUROLOGY
DT Meeting Abstract
DE Hospitalization; death; COVID-19; epilepsy
C1 [Vignatelli, L.; Zenesini, C.; Baccari, F.; Licchetta, L.; Mostacci, B.; Pasini, E.; Michelucci, R.; Nonino, F.] IRCCS, Ist Sci Neurol Bologna, Bologna, Italy.
   [Muccioli, L.; Taruffi, L.; Tinuper, P.; Bisulli, F.] Univ Bologna, Dept Biomed & Neuromotor Sci, Bologna, Italy.
C3 IRCCS Istituto delle Scienze Neurologiche di Bologna (ISNB); University
   of Bologna

TC 0
Z9 0
PD JUL
PY 2022
VL 29
SU 1
MA EPO-057
BP 407
EP 407
WC Clinical Neurology; Neurosciences
ER

PT J
AU Kumar, H
Gupta, R
AF Kumar, Hemant
Gupta, Rachna
TI Neuroinvasion of severe acute respiratory syndrome corona virus-2 (SARS-CoV-2): future risk of epilepsy
SO INTERNATIONAL JOURNAL OF NEUROSCIENCE
DT Review; Early Access
DE SARS-CoV-2; neuroinvasion; seizure; epileptogenesis; risk; epilepsy
ID STATUS EPILEPTICUS; SYNAPTIC PLASTICITY; CEREBRAL VEIN; DISEASE 2019; COVID-19; RECEPTOR; EPILEPTOGENESIS; MANIFESTATIONS; NEUROPATHOLOGY; COMPLICATIONS
AB Central nervous system (CNS) infection is one of the important risk factors for epilepsy. COVID-19 pandemic, which is caused by severe acute respiratory syndrome coronavirus-2 (SARS-CoV-2) has primarily been considered to involve respiratory system only, but it can also affect the CNS. A wide range of neurological manifestations have been reported in SARS-CoV-2 infected patients including seizures, status epilepticus, stroke, which are considered as important risk factors for the development of epilepsy. In post-mortem, brain tissue samples of COVID-19 patients have shown neuropathological changes and presence of SARS-CoV-2 RNA and viral proteins. In this review, mechanisms of SARS-CoV-2 neuroinvasion like neuronal retrograde trans-synaptic route and vascular route are described along with important neurological manifestations in COVID-19 patients such as seizures and cerebrovascular diseases, which have been found to be associated with the development of epilepsy. Hence, an increased risk of future burden of epilepsy in susceptible COVID-19 survivors has been proposed and preventive measures are suggested. The present review highlights about the possible association between neurological manifestations and future risk of epilepsy in COVID-19 patients.
C1 [Kumar, Hemant; Gupta, Rachna] Univ Delhi, Univ Coll Med Sci, Dept Pharmacol, Delhi, India.
C3 University of Delhi; University College of Medical Sciences
RP Gupta, R (通讯作者), Univ Delhi, Univ Coll Med Sci, Dept Pharmacol, Delhi, India.
EM drrachna1@rediffmail.com
TC 0
Z9 0

DI 10.1080/00207454.2022.2100784
EA JUL 2022
WC Neurosciences
ER

PT J
AU Zalpoor, H
Akbari, A
Nabi-Afjadi, M
Forghaniesfidvajani, R
Tavakol, C
Barzegar, Z
Iravanpour, F
Hosseini, M
Mousavi, SR
Farrokhi, MR
AF Zalpoor, Hamidreza
Akbari, Abdullatif
Nabi-Afjadi, Mohsen
Forghaniesfidvajani, Razieh
Tavakol, Chanour
Barzegar, Zohreh
Iravanpour, Farideh
Hosseini, Mahshid
Mousavi, Seyed Reza
Farrokhi, Majid Reza
TI Hypoxia-inducible factor 1 alpha (HIF-1 alpha) stimulated and P2X7 receptor activated by COVID-19, as a potential therapeutic target and risk factor for epilepsy
SO HUMAN CELL
DT Review
DE Epilepsy; risk factor; Seizure; COVID-19; SARS-CoV-2; HIF-1 alpha
ID CENTRAL-NERVOUS-SYSTEM; EXTRACELLULAR ATP; P2X(7) RECEPTOR; EXPRESSION; INFECTION; INFLAMMATION; ANTAGONISTS; SEIZURES
AB Based on available evidence, severe acute respiratory syndrome coronavirus 2 (SARS-CoV-2) is a neuroinvasive virus. According to the centers for disease control and prevention (CDC), coronavirus disease 2019 (COVID-19) may cause epilepsy. In this line, COVID-19 can stimulate hypoxia-inducible factor-1 alpha (HIF-1 alpha) and activate P2X7 receptor. Both HIF-1 alpha and P2X7 receptors are linked to epileptogenesis and seizures. Therefore, in the current study, we suggested that COVID-19 may have a role in epileptogenesis and seizure through HIF-1 alpha stimulation and P2X7 receptor activation. Consequently, pharmacological targeting of these factors could be a promising therapeutic approach for such patients.
C1 [Zalpoor, Hamidreza; Akbari, Abdullatif; Barzegar, Zohreh; Iravanpour, Farideh; Hosseini, Mahshid; Mousavi, Seyed Reza; Farrokhi, Majid Reza] Shiraz Univ Med Sci, Shiraz Neurosci Res Ctr, Dept Neurosurg, Shiraz, Iran.
[Zalpoor, Hamidreza; Akbari, Abdullatif; Forghaniesfidvajani, Razieh] Universal

Sci Educ & Res Network USERN, Network Immun Infect Malignancy & Autoimmun NIIMA, Tehran, Iran.
[Nabi-Afjadi, Mohsen] Tarbiat Modares Univ, Fac Biol Sci, Dept Biochem, Tehran, Iran.
[Tavakol, Chanour] Univ Tehran Med Sci, Med Sch, Tehran, Iran.
[Mousavi, Seyed Reza; Farrokhi, Majid Reza] Shiraz Univ Med Sci, Dept Neurosurg, Shiraz, Iran.
C3 Shiraz University of Medical Science; Tarbiat Modares University; Tehran University of Medical Sciences; Shiraz University of Medical Science
RP Zalpoor, H; Farrokhi, MR (通讯作者), Shiraz Univ Med Sci, Shiraz Neurosci Res Ctr, Dept Neurosurg, Shiraz, Iran.; Zalpoor, H (通讯作者), Universal Sci Educ & Res Network USERN, Network Immun Infect Malignancy & Autoimmun NIIMA, Tehran, Iran.; Farrokhi, MR (通讯作者), Shiraz Univ Med Sci, Dept Neurosurg, Shiraz, Iran.
EM hamidreza.zlpr1998@gmail.com; farrokhimr@yahoo.com
TC 1
Z9 1
PD SEP
PY 2022
VL 35
IS 5
BP 1338
EP 1345
DI 10.1007/s13577-022-00747-9
EA JUL 2022
WC Cell Biology
ER

PT J
AU Muccioli, L
Zenesini, C
Taruffi, L
Licchetta, L
Mostacci, B
Di Vito, L
Pasini, E
Volpi, L
Riguzzi, P
Ferri, L
Baccari, F
Nonino, F
Michelucci, R
Tinuper, P
Vignatelli, L
Bisulli, F
AF Muccioli, Lorenzo
Zenesini, Corrado
Taruffi, Lisa

Licchetta, Laura
Mostacci, Barbara
Di Vito, Lidia
Pasini, Elena
Volpi, Lilia
Riguzzi, Patrizia
Ferri, Lorenzo
Baccari, Flavia
Nonino, Francesco
Michelucci, Roberto
Tinuper, Paolo
Vignatelli, Luca
Bisulli, Francesca
TI Risk of hospitalization and death for COVID-19 in persons with epilepsy over a 20-month period: The EpiLink Bologna cohort, Italy
SO EPILEPSIA
DT Article; Early Access
DE antiseizure medication; epidemiology; encephalopathy; epilepsy; outcome
ID EPIDEMIOLOGY; PEOPLE
AB Objective Data on COVID-19 outcomes in persons with epilepsy (PWE) are scarce and inconclusive. We aimed to study the risk of hospitalization and death for COVID-19 in a large cohort of PWE from March 1, 2020 to October 31, 2021. Methods The historical cohort design (EpiLink Bologna) compared adult PWE grouped into people with focal epilepsy (PFE), idiopathic generalized epilepsy (PIGE), and developmental and/or epileptic encephalopathy (PDEE), and a population cohort matched (ratio 1:10) for age, sex, residence, and comorbidity (assessed with the multisource comorbidity score), living in the local health trust of Bologna (approximately 800 000 residents). Clinical data were linked to health administrative data. Results In both cohorts (EpiLink: n = 1575 subjects, 1128 PFE, 267 PIGE, 148 PDEE, 32 other; controls: n = 15 326 subjects), 52% were females, and the mean age was 50 years (SD = 18). Hospital admissions for COVID-19 in the whole period were 49 (3.1%) in PWE and 225 (1.5%) in controls. The adjusted hazard ratio (aHR) in PWE was 1.9 (95% confidence interval [CI] = 1.4-2.7). The subgroups at higher risk were PFE (aHR = 1.9, 95% CI = 1.3-2.8) and PDEE (aHR = 3.9, 95% CI = 1.7-8.7), whereas PIGE had a risk comparable to the controls (aHR = 1.1, 95% CI = .3-3.5). Stratified analyses of the two main epidemic waves (March-May 2020, October 2020-May 2021) disclosed a higher risk of COVID-19-related hospitalization during the first epidemic wave (March-May 2020; aHR = 3.8, 95% CI = 2.2-6.7). Polytherapy with antiseizure medications contributed to a higher risk of hospital admission. Thirty-day risk of death after hospitalization was 14% in both PWE and controls. Significance During the first 20 months since the outbreak of COVID-19 in Bologna, PWE had a doubled risk of COVID-19 hospital admission compared to a matched control population. Conversely, epilepsy did not represent a risk factor for COVID-19-related death.
C1 [Muccioli, Lorenzo; Taruffi, Lisa; Ferri, Lorenzo; Tinuper, Paolo; Bisulli, Francesca] Univ Bologna, Dept Biomed & Neuromotor Sci, Bologna, Italy.
[Zenesini, Corrado; Licchetta, Laura; Mostacci, Barbara; Di Vito, Lidia; Pasini, Elena; Volpi, Lilia; Riguzzi, Patrizia; Baccari, Flavia; Nonino, Francesco; Michelucci,

Roberto; Tinuper, Paolo; Vignatelli, Luca; Bisulli, Francesca] IRCCS Ist Sci Neurol Bologna, European Reference Network EpiCARE, Sci Inst Res & Hlth Care, Bologna, Italy.
C3 University of Bologna; IRCCS Istituto delle Scienze Neurologiche di Bologna (ISNB)
RP Bisulli, F (通讯作者), IRCCS Ist Sci Neurol Bologna, 3 Altura St, Bologna, Italy.
EM francesca.bisulli@unibo.it
TC 0
Z9 0
DI 10.1111/epi.17356
EA JUL 2022
WC Clinical Neurology
ER

PT J
AU Wanounou, M
Caraco, Y
Levy, RH
Bialer, M
Perucca, E
AF Wanounou, Maor
Caraco, Yoseph
Levy, Rene H.
Bialer, Meir
Perucca, Emilio
TI Clinically Relevant Interactions Between Ritonavir-Boosted Nirmatrelvir and Concomitant Antiseizure Medications: Implications for the Management of COVID-19 in Patients with Epilepsy
SO CLINICAL PHARMACOKINETICS
DT Review
DE Ritonavir-Boosted Nirmatrelvir; antiseizure medication; Management; COVID-19; epilepsy
ID LOW-DOSE RITONAVIR; DRUG-INTERACTIONS; P-GLYCOPROTEIN; PHARMACOKINETIC ENHANCEMENT; PLASMA-CONCENTRATIONS; ANTIEPILEPTIC DRUGS; PROTEASE INHIBITORS; CYP3A INHIBITION; HEPATIC CYP3A; LOPINAVIR/RITONAVIR
AB Ritonavir-boosted nirmatrelvir (RBN) has been authorized recently in several countries as an orally active anti-SARS-CoV-2 treatment for patients at high risk of progressing to severe COVID-19 disease. Nirmatrelvir is the active component against the SARS-CoV-2 virus, whereas ritonavir, a potent CYP3A inhibitor, is intended to boost the activity of nirmatrelvir by increasing its concentration in plasma to ensure persistence of antiviral concentrations during the 12-hour dosing interval. RBN is involved in many clinically important drug-drug interactions both as perpetrator and as victim, which can complicate its use in patients treated with antiseizure medications (ASMs). Interactions between RBN and ASMs are bidirectional. As perpetrator, RBN may increase the plasma concentration of a number of ASMs that are CYP3A4 substrates, possibly leading to toxicity. As victims, both nirmatrelvir and ritonavir are subject to metabolic induction by concomitant treatment with potent enzyme-inducing ASMs (carbamazepine, phenytoin, phenobarbital and primidone). According to US and European

prescribing information, treatment with these ASMs is a contraindication to the use of RBN. Although remdesivir is a valuable alternative to RBN, it may not be readily accessible in some settings due to cost and/or need for intravenous administration. If remdesivir is not an appropriate option, either bebtelovimab or molnupiravir may be considered. However, evidence about the clinical efficacy of bebtelovimab is still limited, and molnupiravir, the only orally active alternative, is deemed to have appreciably lower efficacy than RBN and remdesivir.
C1 [Wanounou, Maor; Caraco, Yoseph] Hadassah Hebrew Univ, Div Med, Clin Pharmacol Unit, Med Ctr, Jerusalem, Israel.
[Levy, Rene H.] Univ Washington, Dept Pharmaceut & Neurol Surg, Seattle, WA 98195 USA.
[Wanounou, Maor; Bialer, Meir] Hebrew Univ Jerusalem, Fac Med, Sch Pharm, Inst Drug Res, Jerusalem, Israel.
[Bialer, Meir] Hebrew Univ Jerusalem, David R Bloom Ctr Pharm, Jerusalem, Israel.
[Perucca, Emilio] Univ Melbourne, Dept Med Austin Hlth, Melbourne, Vic, Australia.
[Perucca, Emilio] Monash Univ, Dept Neurosci, Melbourne, Vic, Australia.
C3 Hebrew University of Jerusalem; Hadassah University Medical Center; University of Washington; University of Washington Seattle; Hebrew University of Jerusalem; Hebrew University of Jerusalem; University of Melbourne; Monash University
RP Bialer, M (通讯作者), Hebrew Univ Jerusalem, Fac Med, Sch Pharm, Inst Drug Res, Jerusalem, Israel.; Bialer, M (通讯作者), Hebrew Univ Jerusalem, David R Bloom Ctr Pharm, Jerusalem, Israel.
EM meirb@ekmd.huji.ac.il
TC 0
Z9 0
PD SEP
PY 2022
VL 61
IS 9
BP 1219
EP 1236
DI 10.1007/s40262-022-01152-z
EA JUL 2022
WC Pharmacology & Pharmacy
ER

PT J
AU Aragon-Nogales, R
Zurita-Cruz, J
Vazquez-Rosales, G
Arias-Flores, R
Gomez-Gonzlez, C
Montano-Luna, V
Samano-Avina, M
Pacheco-Rosas, D
Flores-Ruiz, E

Villasis-Keever, M
Miranda-Novales, G
AF Aragon-Nogales, Ranferi
Zurita-Cruz, Jessie
Vazquez-Rosales, Guillermo
Arias-Flores, Rafael
Gomez-Gonzlez, Claudia
Montano-Luna, Victoria
Samano-Avina, Mariana
Pacheco-Rosas, Daniel
Flores-Ruiz, Eric
Villasis-Keever, Miguel
Miranda-Novales, Guadalupe
TI Clinical presentation of pediatric patients with symptomatic SARS-CoV-2 infection during the first months of the COVID-19 pandemic in a single center in Mexico City
SO FRONTIERS IN PEDIATRICS
DT Article
DE COVID-19; SARS-CoV-2; children; adolescents; mortality; prognosis
ID CHILDREN; DEATH; RISK
AB BackgroundThe clinical spectrum of COVID-19 is broad, from asymptomatic to severe cases and death. The objective of this study is to analyze the clinical course of patients attended during the first months of the SARS-CoV-2 pandemic in a third-level pediatric hospital. MethodsDesign: prospective cohort study. Patients with viral respiratory disease or suspected cases of COVID-19 were evaluated at the Pediatric Hospital, National Medical Center XXI Century, Mexico City, from 21 March 2020 to 13 January 2021. Statistical analysis: Chi-square test and Fisher's exact test were used for comparisons; a logistic regression model was constructed to identify clinical or laboratory characteristics associated with critical disease. A p-value < 0.05 was considered statistically significant. ResultsA total of 697 patients met the operational definition of viral respiratory disease or suspected cases of COVID-19 and underwent real-time reverse transcription polymerase chain reaction (rRT-PCR) SARS-CoV-2 testing. Patients with a positive result were included. Of the 181 patients (26%), 121 (66.8%) had mild disease and were treated as outpatients and 60 (33.1%) were hospitalized. A total of six patients met the criteria for multisystem inflammatory syndrome in children (MIS-C). Of the 60 inpatients, 65% were males, and 82% had one or more comorbidities. The main comorbidities were cancer (42%) and overweight (15%). The median hospital stay was 9 days. The inpatients had a higher frequency of fever, general malaise, dyspnea, chills, polypnea, and cyanosis than the outpatients ($p < 0.05$). Only 21.4% of the outpatients had one or more comorbidities, which were lower than in the hospitalized patients ($p < 0.001$). Laboratory data at admission were similar between critically ill and those with moderate and severe disease. The patients who developed pneumonia were at higher risk of critical disease, while older age was associated with a better prognosis. A total of 13 of the 60 inpatients died (mortality 7.1%). All but one had one or more comorbidities: four had cancer, four congenital heart disease, one chronic kidney disease and epilepsy, one Epstein-Barr virus-induced hemophagocytic lymphohistiocytosis, one obesity, and one diabetes mellitus. ConclusionHospital

mortality is high, especially in children with comorbidities. Despite 2 years having passed since the beginning of the COVID-19 pandemic, the epidemiological and clinical data on children are still helpful to improve their prognosis.
C1 [Aragon-Nogales, Ranferi; Vazquez-Rosales, Guillermo; Montano-Luna, Victoria; Samano-Avina, Mariana; Pacheco-Rosas, Daniel; Flores-Ruiz, Eric] Mexican Inst Social Secur, Natl Med Ctr 21 Century, Infect Dis Dept, Pediat Hosp, Mexico City, Mexico.
[Zurita-Cruz, Jessie] Univ Nacl Autonoma Mexico, Pediat Hosp Federico Gomez, Fac Med, Mexico City, Mexico.
[Arias-Flores, Rafael; Gomez-Gonzlez, Claudia] Mexican Inst Social Secur, Hosp Epidemiol Div, Natl Med Ctr 21 Century, Pediat Hosp, Mexico City, Mexico.
[Villasis-Keever, Miguel; Miranda-Novales, Guadalupe] Mexican Inst Social Secur, Natl Med Ctr 21 Century, Anal & Synth Evidence Res Unit, Mexico City, Mexico.
C3 Instituto Mexicano del Seguro Social; Universidad Nacional Autonoma de Mexico; Instituto Mexicano del Seguro Social; Instituto Mexicano del Seguro Social
RP Miranda-Novales, G (通讯作者), Mexican Inst Social Secur, Natl Med Ctr 21 Century, Anal & Synth Evidence Res Unit, Mexico City, Mexico.
EM mirandaguadalupe2707@yahoo.com
TC 0
Z9 0
PD JUL 28
PY 2022
VL 10
AR 912784
DI 10.3389/fped.2022.912784
WC Pediatrics
ER

PT J
AU Asadi-Pooya, AA
Karimi, A
Razavizadegan, SMA
Ashjazadeh, N
Nemati, H
AF Asadi-Pooya, Ali A.
Karimi, Afrooz
Razavizadegan, Seyed Mohammad Ali
Ashjazadeh, Nahid
Nemati, Hamid
TI COVID-19 vaccine-related frequently asked questions (FAQs) by people with epilepsy and carers in Iran; educational video is included
SO EPILEPSY & BEHAVIOR
DT Article
DE Coronavirus; COVID-19; Epilepsy; Seizure; Vaccine; carer
AB Objective: The aim of the current study was to inquire the questions and concerns of people with epilepsy (PWE) about COVID-19 vaccines in order to provide a more realistic list of their frequently asked questions (FAQs).Methods: We surveyed all the

consecutive PWE or their care-givers who were referred to our neurology clinics (Shiraz University of Medical Sciences) during January-February 2022. We collected their questions and concerns in relation to COVID vaccines based on a predesigned brief questionnaire. Informed consent to participate in the study was obtained from the participants.Results: In total, 452 people participated in the study; 291 people (64.4%) did not have any questions or concerns with regard to the COVID-19 vaccination. Having any questions or concerns about COVID-19 vaccination was significantly associated with not being vaccinated. Questions and concerns about the adverse effects of COVID-19 vaccines [seizure worsening, general adverse effects, long-term effects (e.g., infertility, cognitive dysfunction)] were by far the most common questions by people with epilepsy and their carers.Conclusion: Our findings may be used by policy-makers to prepare appropriate educational materials to provide the best targeted and tailored information to people with epilepsy and their carers to convince them of the necessity and safety of COVID-19 vaccination. Such an educational material must include enough information on the associated adverse effects of COVID-19 vaccines and should also discuss some other important issues such as indications of these vaccines in special populations and drug-vaccine interactions. (c) 2022 Elsevier Inc. All rights reserved.
C1 [Asadi-Pooya, Ali A.; Karimi, Afrooz; Razavizadegan, Seyed Mohammad Ali; Nemati, Hamid] Shiraz Univ Med Sci, Epilepsy Res Ctr, Shiraz, Iran.
[Asadi-Pooya, Ali A.] Thomas Jefferson Univ, Jefferson Comprehens Epilepsy Ctr, Dept Neurol, Philadelphia, PA 19107 USA.
[Ashjazadeh, Nahid] Shiraz Univ Med Sci, Dept Neurol, Shiraz, Iran.
C3 Shiraz University of Medical Science; Jefferson University; Shiraz University of Medical Science
RP Asadi-Pooya, AA (通讯作者), Shiraz Univ Med Sci, Epilepsy Res Ctr, Shiraz, Iran.
EM aliasadipooya@yahoo.com
TC 0
Z9 0
PD AUG
PY 2022
VL 133
AR 108763
DI 10.1016/j.yebeh.2022.108763
WC Behavioral Sciences; Clinical Neurology; Psychiatry
ER

PT J
AU Correale, C
Falamesca, C
Tondo, I
Borgi, M
Cirulli, F
Truglio, M
Papa, O
Vagnoli, L
Arzilli, C
Venturino, C

Pellegrini, M
Manfredi, V
Sterpone, R
Capitello, TG
Gentile, S
Cappelletti, S
AF Correale, Cinzia
Falamesca, Chiara
Tondo, Ilaria
Borgi, Marta
Cirulli, Francesca
Truglio, Mauro
Papa, Oriana
Vagnoli, Laura
Arzilli, Cinzia
Venturino, Cristina
Pellegrini, Michele
Manfredi, Valentina
Sterpone, Rossella
Grimaldi Capitello, Teresa
Gentile, Simonetta
Cappelletti, Simona
TI Depressive Anxiety Symptoms in Hospitalized Children with Chronic Illness during the First Italian COVID-19 Lockdown
SO CHILDREN-BASEL
DT Article
DE COVID-19; lockdown; mental health; chronic illness; anxiety; depression; children
ID CHRONIC HEALTH CONDITIONS; MENTAL-HEALTH; CHILDHOOD EPILEPSY; ADOLESCENTS; PREVALENCE; COMORBIDITY; DISORDERS; SEVERITY; RISK
AB COVID-19 is continuing to spread around the world, having a direct impact on people's daily lives and health. Although the knowledge of the impact of the COVID-19 pandemic on mental health in the general population is now well established, there is less information on its effect on specific and vulnerable populations, such as children with chronic illness (CI). We conducted a multi-centered cross-sectional study among pediatric patients in six public children's hospitals in Italy during the first lockdown, with the aim of assessing the proportion of children with CI presenting anxiety and depressive symptoms, and the clinical and demographic characteristics affecting such symptomatology. We included children with at least one chronic condition, with no cognitive delay, aged between 11 and 18 years. Brief standardized questionnaires were administered during medical scheduled visits to screen anxiety and depressive symptoms. We found a very high proportion of children showing mild to severe depressive and anxiety symptomatology (approximately 68% and 63%, respectively). Our results highlight the need of ensuring tailored psychological interventions to protect children with CI from the effect of the pandemic (and related restrictive measures such as quarantine and social distancing), with the final aim of promoting mental health and psychological well-being in this vulnerable population.
C1 [Correale, Cinzia; Falamesca, Chiara; Tondo, Ilaria; Grimaldi Capitello, Teresa;

Cappelletti, Simona] IRCCS Bambino Gesu Children Hosp, Clin Psychol Unit, I-00146 Rome, Italy.
[Borgi, Marta; Cirulli, Francesca] Ist Super Sanita, Ctr Behav Sci & Mental Hlth, I-00161 Rome, Italy.
[Truglio, Mauro] Massey Univ, Sch Fundamental Sci, Palmerston North 4442, New Zealand.
[Papa, Oriana] Reg Pediat Hosp G Salesi, Childrens Neuropsychiat Ward, I-60123 Ancona, Italy.
[Vagnoli, Laura; Arzilli, Cinzia] Meyer Childrens Hosp, Pediat Psychol, I-50139 Florence, Italy.
[Venturino, Cristina] IRCCS Ist Giannina Gaslini, Psychol Unit, I-16147 Genoa, Italy.
[Pellegrini, Michele] Azienda Osped Univ Policlin Giovanni XXIII, I-70124 Bari, Italy.
[Manfredi, Valentina; Sterpone, Rossella] AOSS Antonio Biagio & C Arrigo Hosp, Psychol Unit, I-15121 Alessandria, Italy.
[Gentile, Simonetta] LUMSA Univ, Dept Humanities, I-00193 Rome, Italy.
C3 IRCCS Bambino Gesu; Istituto Superiore di Sanita (ISS); Massey University; University of Florence; Azienda Ospedaliera Universitaria (AOU) MEYER; University of Genoa; IRCCS Istituto Giannina Gaslini; Universita LUMSA
RP Correale, C (通讯作者), IRCCS Bambino Gesu Children Hosp, Clin Psychol Unit, I-00146 Rome, Italy.
EM cinzia.correale@opbg.net; chiara.falamesca@opbg.net; ilaria.tondo@opbg.net; marta.borgi@iss.it; francesca.cirulli@iss.it; mauro.truglio@gmail.com; oriana.papa@ospedaliriuniti.marche.it; laura.vagnoli@meyer.it; cinzia.arzilli@meyer.it; cristinaventurino@gaslini.org; michele.pellegrini@policlinico.ba.it; valentina.manfredi@ospedale.al.it; rsterpone@ospedale.al.it; teresa.grimaldi@opbg.net; s.gentile6@lumsa.it; simona.cappelletti@opbg.net
TC 0
Z9 0
PD AUG
PY 2022
VL 9
IS 8
AR 1156
DI 10.3390/children9081156
WC Pediatrics
ER

PT J
AU Yalcin, N
Allegaert, K
AF Yalcin, Nadir
Allegaert, Karel

TI COVID-19 and antiepileptic drugs: an approach to guide practices when nirmatrelvir/ritonavir is co-prescribed
SO EUROPEAN JOURNAL OF CLINICAL PHARMACOLOGY
DT Article
DE SARS-CoV-2; Seizure; Anticonvulsants; Nirmatrelvir; Ritonavir; Dosing; Drug interaction
AB Management and dose adjustment are a major concern for clinicians in the absence of specific clinical outcome data for patients on antiepileptic drugs (AEDs), in the event of short-term (5 days) nirmatrelvir/ritonavir co-exposure. Therefore, in this report, we identified drugs that require dose adjustment because of drug-drug interactions (DDIs) between nirmatrelvir/ ritonavir and AEDs. We hereby used four databases (Micromedex Drug Interaction, Liverpool Drug Interaction Group for COVID-19 Therapies, Medscape Drug Interaction Checker, and Lexicomp Drug Interactions) and DDI-Predictor.

In the light of applying the DDI-Predictor, for carbamazepine, clobazam, oxcarbazepine, eslicarbazepine, phenytoin, phenobarbital, pentobarbital, rufinamide, and valproate as CYP3A4 inducers, we recommend that a dose adjustment of short-term nirmatrelvir/ritonavir as a substrate (victim) drug would be more appropriate instead of these AEDs to avoid impending DDI-related threats in patients with epilepsy.
C1 [Yalcin, Nadir] Hacettepe Univ, Fac Pharm, Dept Clin Pharm, Ankara, Turkey.
[Yalcin, Nadir; Allegaert, Karel] Erasmus MC, Dept Clin Pharm, Rotterdam, Netherlands.
[Allegaert, Karel] Katholieke Univ Leuven, Dept Pharmaceut & Pharmacol Sci, Leuven, Belgium.
[Allegaert, Karel] Katholieke Univ Leuven, Dept Dev & Regenerat, Leuven, Belgium.
C3 Hacettepe University; Erasmus University Rotterdam; Erasmus MC; KU Leuven; KU Leuven
RP Yalcin, N (通讯作者), Hacettepe Univ, Fac Pharm, Dept Clin Pharm, Ankara, Turkey.; Yalcin, N (通讯作者), Erasmus MC, Dept Clin Pharm, Rotterdam, Netherlands.
EM nadir.yalcin@hotmail.com; karel.allegaert@uzleuven.be
TC 0
Z9 0
PD OCT
PY 2022
VL 78
IS 10
BP 1697
EP 1701
DI 10.1007/s00228-022-03370-7
EA AUG 2022
WC Pharmacology & Pharmacy
ER

PT J
AU Chan, CCH
Choi, CH
Lui, WT

Ip, B
Ma, KKY
Ma, SH
Fan, FSY
Au, L
Lau, A
Chan, AYY
Ip, V
Soo, Y
Leung, T
Mok, V
Leung, H
AF Chan, Charlie C. H.
Choi, Chun-Ho
Lui, Wai Ting
Ip, Bonaventure
Ma, Karen K. Y.
Ma, Sze Ho
Fan, Florence S. Y.
Au, Lisa
Lau, Alexander
Chan, Anne Y. Y.
Ip, Vincent
Soo, Yannie
Leung, Thomas
Mok, Vincent
Leung, Howan
TI A cross-sectional study of COVID-19 vaccination patterns among patients with epilepsy in Hong Kong
SO EPILEPSIA OPEN
DT Article; Early Access
DE COVID-19; epilepsy; vaccine
AB Objective As Hong Kong faced the 5th wave of the COVID-19 pandemic, the facilitators and hurdles toward effective vaccination is important for healthcare professionals to understand the vaccination gap among patients with epilepsy. Methods A cross-sectional, pragmatic study of COVID-19 vaccination was performed at a tertiary epilepsy center with regards to patterns of vaccination and any unusually high rate of adverse events. Patients having recent visits at the epilepsy center (4 months) had their anonymized electronic linkage records examined 12 months after the inception of vaccination program for types of vaccines, seizure demographics, and adverse events following immunization (AEFI). Results A total of 200 patients with epilepsy and their anonymized data were analyzed. The vaccine uptake was approximately 60% of that of the general population. Twice as many patients with epilepsy chose to receive mRNA vaccine as compared with inactivated vaccine. The proportion of patients who kept up-to-date with all available dosing was 7%. Patients with epilepsy with genetic etiology were least likely to receive vaccination (13/38, 34%, P = .02). There was no unreasonably high rate of unacceptable side effects after vaccination among patients with epilepsy. Only

3 patients reported worsening of seizures without meeting the criteria for AEFI. Refractory epilepsy, allergy to antiseizure medications and elder age (>= 65) did not confer any significant difference in vaccination patterns or adverse effects. Significance A vaccination gap exists among epilepsy patients which calls for actionable strategies for improving vaccine uptake, including education and outreach programs.
C1 [Chan, Charlie C. H.; Choi, Chun-Ho; Lui, Wai Ting; Ip, Bonaventure; Ma, Karen K. Y.; Ma, Sze Ho; Fan, Florence S. Y.; Au, Lisa; Lau, Alexander; Chan, Anne Y. Y.; Ip, Vincent; Soo, Yannie; Leung, Thomas; Mok, Vincent; Leung, Howan] Prince Wales Hosp, Dept Med & Therapeut, Div Neurol, Hong Kong, Peoples R China.
[Leung, Howan] Prince Wales Hosp, Dept Med & Therapeut, Hong Kong, Peoples R China.
C3 Chinese University of Hong Kong; Prince of Wales Hospital; Chinese University of Hong Kong; Prince of Wales Hospital
RP Leung, H (通讯作者), Prince Wales Hosp, Shatin, 9-F Clin Sci Bldg, 33 Ngan Shing St, Hong Kong, Peoples R China.
EM howanleung@cuhk.edu.hk
TC 0
Z9 0
DI 10.1002/epi4.12629
EA AUG 2022
WC Clinical Neurology; Neurosciences
ER

PT J
AU Miocinovic, S
Ostrem, JL
Okun, MS
Bullinger, KL
Riva-Posse, P
Gross, RE
Buetefisch, CM
AF Miocinovic, Svjetlana
Ostrem, Jill L.
Okun, Michael S.
Bullinger, Katie L.
Riva-Posse, Patricio
Gross, Robert E.
Buetefisch, Cathrin M.
TI Recommendations for Deep Brain Stimulation Device Management During a Pandemic Comment
SO JOURNAL OF PARKINSONS DISEASE
LA English
DT Editorial Material
DE COVID-19; Management; battery depletion; telemedicine; DBS withdrawal
ID TREATMENT-RESISTANT DEPRESSION; BATTERY DEPLETION; STATUS DYSTONICUS
AB Most medical centers are postponing elective procedures and deferring non-urgent clinic visits to conserve hospital resources and prevent spread of COVID-19. The

pandemic crisis presents some unique challenges for patients currently being treated with deep brain stimulation (DBS). Movement disorder (Parkinson's disease, essential tremor, dystonia), neuropsychiatric disorder (obsessive compulsive disorder, Tourette syndrome, depression), and epilepsy patients can develop varying degrees of symptom worsening from interruption of therapy due to neurostimulator battery reaching end of life, device malfunction or infection. Urgent intervention to maintain or restore stimulation may be required for patients with Parkinson's disease who can develop a rare but potentially life-threatening complication known as DBS-withdrawal syndrome. Similarly, patients with generalized dystonia can develop status dystonicus, patients with obsessive compulsive disorder can become suicidal, and epilepsy patients can experience potentially life-threatening worsening of seizures as a result of therapy cessation. DBS system infection can require urgent, and rarely emergent surgery. Elective interventions including new implantations and initial programming should be postponed. For patients with existing DBS systems, the battery status and electrical integrity interrogation can now be performed using patient programmers, and employed through telemedicine visits or by phone consultations. The decision for replacement of the implantable pulse generator to prevent interruption of DBS therapy should be made on a case-by-case basis taking into consideration battery status and a patient's tolerance to potential therapy disruption. Scheduling of the procedures, however, depends heavily on the hospital system regulations and on triage procedures with respect to safety and resource utilization during the health crisis.
C1 [Miocinovic, Svjetlana; Bullinger, Katie L.; Buetefisch, Cathrin M.] Emory Univ, Dept Neurol, 12 Execut Pk NE, Atlanta, GA 30329 USA.
[Ostrem, Jill L.] Univ Calif San Francisco, Dept Neurol, Weill Inst Neurosci, San Francisco, CA USA.
[Okun, Michael S.] Univ Florida, Dept Neurol, Norman Fixel Inst Neurol Dis, Gaineseville, FL USA.
[Riva-Posse, Patricio] Emory Univ, Dept Psychiat & Behav Sci, Atlanta, GA 30322 USA.
[Gross, Robert E.] Emory Univ, Dept Neurosurg, Atlanta, GA 30322 USA.
[Buetefisch, Cathrin M.] Emory Univ, Dept Rehabil Med, Atlanta, GA 30322 USA.
C3 Emory University; University of California System; University of California San Francisco; State University System of Florida; University of Florida; Emory University; Emory University; Emory University
RP Miocinovic, S (通讯作者), Emory Univ, Dept Neurol, 12 Execut Pk NE, Atlanta, GA 30329 USA.
EM Svjetlana.Miocinovic@emory.edu
TC 25
Z9 27
PY 2020
VL 10
IS 3
BP 903
EP 910
DI 10.3233/JPD-202072
WC Neurosciences
ER

PT J
AU Meshkat, S
Salimi, A
Joshaghanian, A
Sedighi, S
Sedighi, S
Aghamollaii, V
AF Meshkat, Shakila
Salimi, Amir
Joshaghanian, Asef
Sedighi, Sogol
Sedighi, Saman
Aghamollaii, Vajiheh
TI Chronic neurological diseases and COVID-19: Associations and considerations
SO TRANSLATIONAL NEUROSCIENCE
LA English
DT Review
DE COVID-19; coronavirus; epilepsy; Parkinson's disease; dementia; multiple sclerosis; SARS-CoV-2; chronic neurological diseases; telemedicine
ID MULTIPLE-SCLEROSIS; PARKINSONS-DISEASE; EPILEPSY; PEOPLE
AB The 2019 novel coronavirus pandemic, severe acute respiratory syndrome CoV-2 (COVID-19), has been a worldwide urgent public health threat, resulting in six- hundred seventy thousand deaths to date. The COVID-19 pandemic has led to a series of public health challenges. One such challenge is the management of diseases such as chronic neurological diseases during an epidemic event. COVID-19 affects all kinds of people, including older people with chronic underlying diseases, who are particularly at risk of severe infection or even death. Chronic neurological diseases such as epilepsy, dementia, Parkinson's disease (PD), and multiple sclerosis (MS) are frequently associated with comorbidities; thus, these patients are in the high-risk category. Therefore, in this article, we review associations and challenges the people with epilepsy, dementia, PD, and MS faces during the COVID-19 pandemic and suggest approaches to provide consensus recommendations on how to provide the best possible care.
C1 [Meshkat, Shakila] Univ Tehran Med Sci, Dept Med, Tehran, Iran.
[Salimi, Amir] Shahid Beheshti Univ Med Sci, Dept Med, Tehran, Iran.
[Joshaghanian, Asef] Hamedan Univ Med Sci, Dept Med, Tehran, Iran.
[Sedighi, Sogol] Shiraz Univ Med Sci, Dept Med, Tehran, Iran.
[Sedighi, Saman] Azad Univ Med Sci, Dept Med, Tehran, Iran.
[Aghamollaii, Vajiheh] Univ Tehran Med Sci, Roozbeh Psychiat Hosp, Neurol Dept, Tehran 1333715914, Iran.
C3 Tehran University of Medical Sciences; Shahid Beheshti University Medical Sciences; Islamic Azad University; Tehran University of Medical Sciences
RP Aghamollaii, V (通讯作者), Univ Tehran Med Sci, Roozbeh Psychiat Hosp, Neurol Dept, Tehran 1333715914, Iran.
EM vajiheh102@gmail.com
TC 7

Z9 7
PD JAN
PY 2020
VL 11
IS 1
BP 294
EP 301
DI 10.1515/tnscl-2020-0141
WC Neurosciences
ER

PT J
AU Yasri, S
   Wiwanikit, V
AF Yasri, Sora
   Wiwanikit, Viroj
TI COVID-19 and Epilepsy
SO ANNALS OF INDIAN ACADEMY OF NEUROLOGY
LA English
DT Letter
DE COVID-19; epilepsy
C1 [Yasri, Sora] Sanitat Med Acad Ctr, Bangkok, Thailand.
   [Wiwanikit, Viroj] Dr DY Patil Univ, Pune, Maharashtra, India.
   [Wiwanikit, Viroj] Hainan Med Univ, Haikou, Hainan, Peoples R China.
C3 Dr DY Patil Vidyapeeth Pune; Hainan Medical University
RP Yasri, S (通讯作者), Sanitat Med Acad Ctr, Bangkok, Thailand.
EM beuyjoob@hotmail.com
TC 9
Z9 10
PD APR
PY 2020
VL 23
IS 7
SU S
BP S43
EP S43
DI 10.4103/aian.AIAN_254_20
WC Clinical Neurology
ER

PT J
AU Hernando-Requejo, V
   Huertas-Gonzalez, N
   Lapena-Motilva, J
   Ogando-Duran, G
AF Hernando-Requejo, V.
   Huertas-Gonzalez, N.

Lapena-Motilva, J.
Ogando-Duran, G.
TI The epilepsy unit during the COVID-19 epidemic: The role of telemedicine and the effects of confinement on patients with epilepsy
SO NEUROLOGIA
LA Spanish
DT Letter
DE COVID-19; epilepsy unit; epilepsy; telemedicine
C1 [Hernando-Requejo, V.; Huertas-Gonzalez, N.; Lapena-Motilva, J.; Ogando-Duran, G.] Hosp Univ Severo Ochoa, Serv Neurol, Madrid, Spain.
[Hernando-Requejo, V.] Hosp Univ HM Sanchinarro, Serv Neurol, Madrid, Spain.
[Hernando-Requejo, V.] Univ CEU San Pablo, Fac Med, Dept Ciencias Med Clin, Madrid, Spain.
C3 Severo Ochoa University Hospital; San Pablo CEU University
RP Hernando-Requejo, V (通讯作者), Hosp Univ Severo Ochoa, Serv Neurol, Madrid, Spain.; Hernando-Requejo, V (通讯作者), Hosp Univ HM Sanchinarro, Serv Neurol, Madrid, Spain.; Hernando-Requejo, V (通讯作者), Univ CEU San Pablo, Fac Med, Dept Ciencias Med Clin, Madrid, Spain.
EM virgitiohernandorequejo@gmail.com
TC 7
Z9 7
PD APR
PY 2020
VL 35
IS 4
BP 274
EP 276
DI 10.1016/j.nrl.2020.04.014
WC Clinical Neurology
ER

PT J
AU Santos-Peyret, A
Duron, RM
Sebastian-Diaz, MA
Melendez, DC
Gomez-Ventura, S
Briceno-Gonzalez, E
Rito, Y
Martinez-Juarez, IE
AF Santos-Peyret, Andrea
Duron, Reyna M.
Sebastian-Diaz, Mario A.
Crail Melendez, Daniel
Gomez-Ventura, Sandra
Briceno-Gonzalez, Eduardo
Rito, Yamel

Martinez-Juarez, Iris E.
TI E-health tools to overcome the gap in epilepsy care before, during and after COVID-19 pandemics
SO REVISTA DE NEUROLOGIA
LA Spanish
DT Review
DE COVID-19; epilepsy care; information technology; SARS-CoV-2; telemedicine
ID FOLLOW-UP; TELEMEDICINE
AB Introduction. Epilepsy is a common chronic neurological disorder that affects around 50 million worldwide and there is an abundance of literature on the health care gap for this sector of the population. This gap will increase with the current pandemic due to COVID-19.

Aim. To evaluate the current availability of digital health tools for the care of people with epilepsy according to the world medical literature and their use during said pandemic.

Development. We reviewed the publications in scientific journals in the last decade that had as their main topic the use of digital health tools or telemedicine focused on the care of patients with epilepsy, including 4 months after the national quarantines due to the appearance of the virus SARS-CoV-2. Seventeen publications were found on the use of telemedicine focused on epilepsy. The most widely used tools internationally are online platforms, followed by mobile applications, videoconferences, epileptic seizure capture systems, checklists, algorithms for understanding medical data, phone calls, tele-encephalography and text messages. None was published during the COVID-19 current pandemic.

Conclusions. Although there is little literature on the use of digital health tools focused on epilepsy, there are several that can be used to fight the attention gap, especially in this global pandemic by COVID-19 that forces quarantines of people and communities for long periods. It is necessary to remove barriers and facilitate patient access to these new information technologies.
C1 [Santos-Peyret, Andrea] Fac Estudios Super, Zaragoza, Spain.

[Santos-Peyret, Andrea] Univ Nacl Autonoma Mexico, Mexico City, DF, Mexico.

[Santos-Peyret, Andrea; Martinez-Juarez, Iris E.] Inst Nacl Neurol & Neurocirugia Manuel Velasco Su, Clin Epilepsia, Mexico City, DF, Mexico.

[Duron, Reyna M.; Crail Melendez, Daniel; Gomez-Ventura, Sandra] Inst Nacl Neurol & Neurocirugia Manuel Velasco Su, Dept Neuropsiquiatria, Mexico City, DF, Mexico.

[Briceno-Gonzalez, Eduardo; Rito, Yamel] Inst Nacl Neurol & Neurocirugia Manuel Velasco Su, Consulta Externa, Mexico City, DF, Mexico.

[Sebastian-Diaz, Mario A.] Univ Anahuac Norte, Programa Doctorado Ciencias Salud, Mexico City, DF, Mexico.

[Martinez-Juarez, Iris E.] Coordinac Ctr Atenc Integral 1 Programa Prioritar, Mexico City, DF, Mexico.

[Duron, Reyna M.; Gomez-Ventura, Sandra] Univ Tecnol Ctr Amer, Fac Ciencias Salud, Tegucigalpa, Honduras.
C3 Universidad Nacional Autonoma de Mexico; Universidad Anahuac
RP Martinez-Juarez, IE (通讯作者), INNN Manuel Velasco Suarez, Avda Insurgentes Sur 3877,Ext 2028, Ciudad De Mexico 14269, Mexico.
EM imartinez@innn.edu.mx

TC 7
Z9 7
PD MAY 1
PY 2020
VL 70
IS 9
BP 323
EP 328
DI 10.33588/rn.7009.2020173
WC Clinical Neurology
ER

PT J
AU Vollono, C
Rollo, E
Romozzi, M
Frisullo, G
Servidei, S
Borghetti, A
Calabresi, P
AF Vollono, Catello
Rollo, Eleonora
Romozzi, Marina
Frisullo, Giovanni
Servidei, Serenella
Borghetti, Alberto
Calabresi, Paolo
TI Focal status epilepticus as unique clinical feature of COVID-19: A case report
SO SEIZURE-EUROPEAN JOURNAL OF EPILEPSY
LA English
DT Article
DE status epilepticus; epilepsy; COVID-19; SARS-CoV-2
AB SARS-CoV-2, a novel zoonotic coronavirus, is currently spreading all over the world, causing a pandemic disease defined coronavirus disease 2019 (COVID-19). The spectrum of COVID-19 ranges from asymptomatic or mild infection to rapidly progressive, acute respiratory distress syndrome and death [1].To the best of our knowledge, status epilepticus has never been described as initial presentation of COVID-19. We report a patient affected by COVID-19 whose primary presentation was a focal status epilepticus.
C1 [Vollono, Catello; Servidei, Serenella] Fdn Policlin Univ Agostino Gemelli IRCCS, Neurofisiopatol, Rome, Italy.
[Rollo, Eleonora; Romozzi, Marina; Servidei, Serenella; Calabresi, Paolo] Univ Cattolica Sacro Cuore, Neurol, Dipartimento Neurosci, Rome, Italy.
[Frisullo, Giovanni; Calabresi, Paolo] Fdn Policlin Univ Agostino Gemelli IRCCS, Neurol, Rome, Italy.
[Borghetti, Alberto] Fdn Policlin Univ Agostino Gemelli IRCCS, UOC Malattie Infett,

Rome, Italy.
C3 Catholic University of the Sacred Heart; IRCCS Policlinico Gemelli;
   Catholic University of the Sacred Heart; IRCCS Policlinico Gemelli;
   Catholic University of the Sacred Heart; IRCCS Policlinico Gemelli;
   Catholic University of the Sacred Heart; IRCCS Policlinico Gemelli
RP Vollono, C （通讯作者）, Fdn Policlin Univ Agostino Gemelli IRCCS, Unit Neurophysiopathol, Largo Agostino Gemelli 8, I-00168 Rome, Italy.
EM lvol@libero.it
TC 105
Z9 109
PD MAY
PY 2020
VL 78
BP 109
EP 112
DI 10.1016/j.seizure.2020.04.009
WC Clinical Neurology; Neurosciences
ER

PT J
AU Aledo-Serrano, A
   Mingorance, A
   Jimenez-Huete, A
   Toledano, R
   Garcia-Morales, I
   Anciones, C
   Gil-Nagel, A
AF Aledo-Serrano, Angel
   Mingorance, Ana
   Jimenez-Huete, Adolfo
   Toledano, Rafael
   Garcia-Morales, Irene
   Anciones, Carla
   Gil-Nagel, Antonio
TI Genetic epilepsies and COVID-19 pandemic: Lessons from the caregiver
   perspective
SO EPILEPSIA
LA English
DT Letter
DE COVID-19; epilepsy; SARS-CoV-2; caregiver
C1 [Aledo-Serrano, Angel; Jimenez-Huete, Adolfo; Toledano, Rafael; Garcia-Morales, Irene; Anciones, Carla; Gil-Nagel, Antonio] Ruber Int Hosp, Dept Neurol, Epilepsy Unit, Madrid, Spain.
   [Aledo-Serrano, Angel] Corachan Clin, Dept Neurosci, Epilepsy Unit, Barcelona, Spain.
   [Mingorance, Ana] Loulou Fdn, London, England.
   [Toledano, Rafael] Ramon & Cajal Univ Hosp, Dept Neurol, Epilepsy Unit, Madrid,

Spain.
[Garcia-Morales, Irene] Clin San Carlos Univ Hosp, Dept Neurol, Epilepsy Unit, Madrid, Spain.
C3 Hospital Universitario Ramon y Cajal; Hospital Clinico San Carlos
RP Aledo-Serrano, A (通讯作者), Ruber Int Hosp, Dept Neurol, Epilepsy Unit, Madrid, Spain.; Aledo-Serrano, A (通讯作者), Corachan Clin, Dept Neurosci, Epilepsy Unit, Barcelona, Spain.
EM aaledo@neurologiaclinica.es
TC 24
Z9 24
PD JUN
PY 2020
VL 61
IS 6
BP 1312
EP 1314
DI 10.1111/epi.16537
EA MAY 2020
WC Clinical Neurology
ER

PT J
AU Hao, XT
Zhou, D
Li, Z
Zeng, GJ
Hao, NY
Li, EZ
Li, WJ
Deng, AP
Lin, MT
Yan, B
AF Hao, Xiaoting
Zhou, Dong
Li, Zhe
Zeng, Guojun
Hao, Nanya
Li, Enzhi
Li, Wenjing
Deng, Aiping
Lin, Mintao
Yan, Bo
TI Severe psychological distress among patients with epilepsy during the COVID-19 outbreak in southwest China
SO EPILEPSIA
LA English
DT Article

DE COVID-19; drug resistant epilepsy; epilepsy; outbreak; psychological distress

ID SERIOUS MENTAL-ILLNESS; PSYCHIATRIC COMORBIDITY; ILAE COMMISSION; RISK-FACTORS; TASK-FORCE; POPULATION; SUICIDE; ANXIETY; STRESS; PEOPLE

AB Objective To compare the severity of psychological distress between patients with epilepsy and healthy controls during the COVID-19 outbreak in southwest China, as well as identify potential risk factors of severe psychological distress among patients with epilepsy.

Methods This cross-sectional case-control study examined a consecutive sample of patients older than 15 years treated at the epilepsy center of West China Hospital between February 1 and February 29, 2020. As controls, sex- and age-matched healthy visitors of inpatients (unrelated to the patients) were also enrolled during the same period. Data on demographics and attention paid to COVID-19 were collected by online questionnaire, data on epilepsy features were collected from electronic medical records, and psychological distress was evaluated using the 6-item Kessler Psychological Distress Scale (K-6). Potential risk factors of severe psychological distress were identified using multivariate logistic regression.

Results The 252 patients and 252 controls in this study were similar along all demographic variables except family income. Patients with epilepsy showed significantly higher K-6 scores than healthy controls and spent significantly more time following the COVID-19 outbreak (both P < .001). Univariate analyses associated both diagnosis of drug-resistant epilepsy and time spent paying attention to COVID-19 with severe psychological distress (defined as K-6 score >12; both P <= .001). Multivariate logistic regression identified two independent predictors of severe psychological distress: time spent paying attention to COVID-19 (odds ratio [OR] = 1.172, 95% confidence interval [CI] = 1.073-1.280) and diagnosis of drug-resistant epilepsy (OR = 0.283, 95% CI = 0.128-0.623).

Significance During public health outbreaks, clinicians and caregivers should focus not only on seizure control but also on mental health of patients with epilepsy, especially those with drug-resistant epilepsy. K-6 scores > 12 indicate severe psychological distress. This may mean, for example, encouraging patients to engage in other activities instead of excessively following media coverage of the outbreak.

C1 [Hao, Xiaoting; Zhou, Dong; Hao, Nanya; Li, Enzhi; Yan, Bo] Sichuan Univ, West China Hosp, Dept Neurol, Chengdu 610000, Sichuan, Peoples R China.

[Li, Zhe] Sichuan Univ, West China Hosp, Mental Hlth Ctr, Chengdu, Sichuan, Peoples R China.

[Zeng, Guojun] Sichuan Univ, Dept Vasc Surg, West China Hosp, Chengdu, Sichuan, Peoples R China.

[Li, Enzhi; Li, Wenjing; Deng, Aiping] Sichuan Univ, West China Sch Nursing, Chengdu, Sichuan, Peoples R China.

[Li, Wenjing; Deng, Aiping] Sichuan Univ, West China Hosp, Outpatient Dept, Chengdu, Sichuan, Peoples R China.

[Lin, Mintao] Sichuan Univ, West China Sch Med, Chengdu, Sichuan, Peoples R China.

C3 Sichuan University; Sichuan University; Sichuan University; Sichuan University; Sichuan University; Sichuan University

RP Yan, B (通讯作者), Sichuan Univ, West China Hosp, Dept Neurol, Chengdu 610000, Sichuan, Peoples R China.

EM yanbo_huaxi@126.com
TC 78
Z9 80
PD JUN
PY 2020
VL 61
IS 6
BP 1166
EP 1173
DI 10.1111/epi.16544
EA MAY 2020
WC Clinical Neurology
ER

PT J
AU Ozdag Acarli, AN
   Samanci, B
   Ekizoglu, E
   Cakar, A
   Sirin, NG
   Gunduz, T
   Parman, Y
   Baykan, B
AF Ozdag Acarli, Ayse Nur
   Samanci, Bedia
   Ekizoglu, Esme
   Cakar, Arman
   Sirin, Nermin Gorkem
   Gunduz, Tuncay
   Parman, Yesim
   Baykan, Betul
TI Coronavirus Disease 2019 (COVID-19) From the Point of View of
   Neurologists: Observation of Neurological Findings and Symptoms During
   the Combat Against a Pandemic
SO NOROPSIKIYATRI ARSIVI-ARCHIVES OF NEUROPSYCHIATRY
LA English
DT Review
DE COVID-19; SARS-CoV-2; neurologic manifestation
ID ACUTE RESPIRATORY SYNDROME; CENTRAL-NERVOUS-SYSTEM; POTENTIAL ROLE;
   TISSUE; INFECTION; BRAIN; SARS
AB Some respiratory viruses have long been known to cause neurological involvement. A novel coronavirus, leading to severe acute respiratory syndrome, also called coronavirus disease 19 (COVID-19), seems to be a new member of neuroinvasive viruses. While severe acute respiratory syndrome coronavirus 2 (SARS-CoV-2) keeps on spreading around the world rapidly, reports about the neurological manifestations associated with SARS-CoV-2, increases day by day. It is reported that a variety of symptoms and syndromes such as headache, dizziness, confusion, ataxia, epilepsy, ischemic stroke, neuropathic

pain and myopathy are common especially in more severe COVID-19 patients. It is also suggested that the development of neurological complications is strongly associated with a poor outcome. On the other hand, hyposmia can be the unique symptom in COVID-19 carriers and this can serve as a marker for identifying the otherwise asymptomatically infected patients. It is thought that SARS-CoV-2 may cause neurological symptoms through direct or indirect mechanisms. Nevertheless, neuroinvasion capability of SARS-CoV2 is confirmed by the presence of the virus, in the cerebrospinal fluid of a COVID-19 patient with encephalitis, and this is proven by gene sequencing. In conclusion, during the COVID-19 pandemic, it is crucial to be aware of the possible neurological complications of the disease. Therefore, in this review, we aimed to report neurological manifestations associated with SARS-CoV-2 and possible underlying pathophysiological mechanisms. Due to the high homology of SARS-CoV-2 with other human coronaviruses such as SARS-CoV or Middle East Respiratory Syndrome (MERS)-CoV, reviewing the neurological involvement also associated with these coronaviruses will provide an idea about the longterm complications of COVID-19.
C1 [Ozdag Acarli, Ayse Nur; Samanci, Bedia; Ekizoglu, Esme; Cakar, Arman; Sirin, Nermin Gorkem; Gunduz, Tuncay; Parman, Yesim; Baykan, Betul] Istanbul Univ, Istanbul Fac Med, Dept Neurol, Istanbul, Turkey.
C3 Istanbul University
RP Ozdag Acarli, AN (通讯作者), Istanbul Univ, Istanbul Fac Med, Dept Neurol, Istanbul, Turkey.
EM nur_ozdag_87@hotmail.com
TC 19
Z9 19
PD JUN
PY 2020
VL 57
IS 2
BP 154
EP 159
DI 10.29399/npa.26148
WC Clinical Neurology
ER

PT J
AU Beniczky, S
Blumcke, I
Rampp, S
Shisler, P
Biesel, E
Wiebe, S
AF Beniczky, Sandor
Blumcke, Ingmar
Rampp, Stefan
Shisler, Priscilla
Biesel, Eva
Wiebe, Samuel

TI e-learning comes of age: Web-based education provided by the International League Against Epilepsy
SO EPILEPTIC DISORDERS
LA English
DT Article
DE epilepsy; e-learning; ILAE; online education
ID MEDICAL-EDUCATION; UNDERGRADUATE; COURSES; EBRAIN
AB Education tools and programs using interactive digital content, distributed on the internet, are increasingly becoming an integral part of postgraduate medical education. The coronavirus pandemic and global lockdown hoisted a major challenge for traditional teaching courses. A timely solution is to focus attention and reinforce web-based teaching programs. For more than 15 years, the ILAE has been developing and managing a wide range of e-learning programs. This paper provides an overview on the e-learning portfolio of the ILAE, including tutored e-courses, self-paced interactive e-courses and online multimedia resources, all linked to the ILAE curriculum and learning objectives addressing specific levels of professional experience. All e-learning programs will become available through the new ILAE Academy platform (www.ilae-academy.org), in July 2020. e-learning is an important tool for reaching the global educational mission of the ILAE.
C1 [Beniczky, Sandor] Danish Epilepsy Ctr, Dept Clin Neurophysiol, Aarhus, Denmark.
[Beniczky, Sandor] Aarhus Univ Hosp, Aarhus, Denmark.
[Beniczky, Sandor] Aarhus Univ, Dept Clin Med, ERN EpiCARE, Aarhus, Denmark.
[Blumcke, Ingmar] Univ Hosp Erlangen, Dept Neuropathol, Collaborating Partner ERN EpiCARE, Erlangen, Germany.
[Rampp, Stefan] Univ Hosp Erlangen, Dept Neurosurg, Erlangen, Germany.
[Rampp, Stefan] Univ Hosp Halle Saale, Dept Neurosurg, Halle, Germany.
[Shisler, Priscilla] ILAE Headquarters Off, Flower Mound, TX USA.
[Biesel, Eva] ILAE Acad, Flower Mound, TX USA.
[Wiebe, Samuel] Univ Calgary, Cumming Sch Med, Dept Clin Neurosci, Calgary, AB, Canada.
[Wiebe, Samuel] Univ Calgary, Cumming Sch Med, Clin Res Unit, Calgary, AB, Canada.
C3 Aarhus University; Aarhus University; University of Erlangen Nuremberg; University of Erlangen Nuremberg; Martin Luther University Halle Wittenberg; University of Calgary; University of Calgary
RP Beniczky, S (通讯作者), Visby Alle 5, DK-4293 Dianalund, Denmark.
EM sbz@filadelfia.dk
TC 7
Z9 7
PD JUN
PY 2020
VL 22
IS 3
BP 237
EP 244
DI 10.1684/epd.2020.1157
WC Clinical Neurology
ER

PT J
AU Niazkar, HR
Zibaee, B
Nasimi, A
Bahri, N
AF Niazkar, Hamid Reza
Zibaee, Behdad
Nasimi, Ali
Bahri, Narjes
TI The neurological manifestations of COVID-19: a review article
SO NEUROLOGICAL SCIENCES
LA English
DT Review
DE Coronavirus; SARS-COV-2; COVID-19; neurologic manifestation
ID CORONAVIRUS DISEASE 2019; EPILEPSY
AB Results Various neurological manifestations have been reported in the literature associated with COVID-19, which in the current study are classified into Central Nervous System (CNS) related manifestations including headache, dizziness, impaired consciousness, acute cerebrovascular disease, epilepsy, and Peripheral Nervous System (PNS) related manifestations such as hyposmia/anosmia, hypogeusia/ageusia, muscle pain, and Guillain-Barre syndrome. Conclusion During the current context of COVID-19 pandemic, physicians should be aware of wide spectrum of neurological COVID-19 sign and symptoms for early diagnosis and isolation of patients. In this regard, COVID-19 has been associated with many neurological manifestations such as confusion, anosmia, and ageusia. Also, various evidences support the possible CNS roles in the COVID-19 pathophysiology. In this regard, further investigation of CNS involvement of SARS-COV-2 is suggested.
C1 [Niazkar, Hamid Reza; Zibaee, Behdad; Nasimi, Ali] Gonabad Univ Med Sci, Fac Med, Student Res Comm, Gonabad, Iran.
[Bahri, Narjes] Gonabad Univ Med Sci, Social Dev & Hlth Promot Res Ctr, Fac Med, Dept Midwifery, Gonabad, Iran.
RP Bahri, N (通讯作者), Gonabad Univ Med Sci, Social Dev & Hlth Promot Res Ctr, Fac Med, Dept Midwifery, Gonabad, Iran.
EM hm185@hotmail.com; behdad.zbe73@gmail.com; alsnasimi@gmail.com;
nargesbahri@yahoo.com
TC 90
Z9 99
PD JUL
PY 2020
VL 41
IS 7
BP 1667
EP 1671
DI 10.1007/s10072-020-04486-3
EA JUN 2020
WC Clinical Neurology; Neurosciences

ER

PT J
AU French, JA
   Brodie, MJ
   Caraballo, R
   Devinsky, O
   Ding, D
   Jehi, L
   Jette, N
   Kanner, A
   Modi, AC
   Newton, CR
   Patel, AA
   Pennell, PB
   Perucca, E
   Sander, JW
   Scheffer, IE
   Singh, G
   Williams, E
   Wilmshurst, J
   Cross, JH
AF French, Jacqueline A.
   Brodie, Martin J.
   Caraballo, Roberto
   Devinsky, Orrin
   Ding, Ding
   Jehi, Lara
   Jette, Nathalie
   Kanner, Andres
   Modi, Avani C.
   Newton, Charles R.
   Patel, Archana A.
   Pennell, Page B.
   Perucca, Emilio
   Sander, Josemir W.
   Scheffer, Ingrid E.
   Singh, Gagandeep
   Williams, Emma
   Wilmshurst, Jo
   Cross, J. Helen
TI Keeping people with epilepsy safe during the COVID-19 pandemic
SO NEUROLOGY
LA English
DT Article
DE COVID-19; Epilepsy; safe
AB Objectives To provide information on the effect of the coronavirus disease of 2019

(COVID-19) pandemic on people with epilepsy and provide consensus recommendations on how to provide the best possible care for people with epilepsy while avoiding visits to urgent care facilities and hospitalizations during the novel coronavirus pandemic.MethodsThe authors developed consensus statements in 2 sections. The first was "How should we/clinicians modify our clinical care pathway for people with epilepsy during the COVID-19 pandemic?" The second was "What general advice should we give to people with epilepsy during this crisis? The authors individually scored statements on a scale of -10 (strongly disagree) to +10 (strongly agree). Five of 11 recommendations for physicians and 3/5 recommendations for individuals/families were rated by all the authors as 7 or above (strongly agree) on the first round of rating. Subsequently, a teleconference was held where statements for which there was a lack of strong consensus were revised.ResultsAfter revision, all consensus recommendations received a score of 7 or above. The recommendations focus on administration of as much care as possible at home to keep people with epilepsy out of health care facilities, where they are likely to encounter COVID-19 (including strategies for rescue therapy), as well as minimization of risk of seizure exacerbation through adherence, and through ensuring a regular supply of medication. We also provide helpful links to additional helpful information for people with epilepsy and health providers.ConclusionThese recommendations may help health care professionals provide optimal care to people with epilepsy during the coronavirus pandemic.
C1 [French, Jacqueline A.] NYU, Dept Neurol, NYU Grossman Sch Med, New York, NY 10003 USA.
[Brodie, Martin J.] Scottish Epilepsy Initiat, Int Bur Epilepsy, Epilepsy Unit, Glasgow, Lanark, Scotland.
[Caraballo, Roberto] Hosp JP Garrahan, Neurol, Buenos Aires, DF, Argentina.
[Devinsky, Orrin] NYU Grossman Sch Med, Dept Neurol, New York, NY USA.
[Ding, Ding] Fudan Univ, Huashan Hosp, Inst Neurol, Shanghai, Peoples R China.
[Jehi, Lara] Cleveland Clin, Epilepsy Ctr, Cleveland, OH 44106 USA.
[Jette, Nathalie] Icahn Sch Med Mt Sinai, Dept Neurol, New York, NY 10029 USA.
[Kanner, Andres] Univ Miami, Miller Sch Med, Dept Neurol, Div Epilepsy, Coral Gables, FL 33124 USA.
[Modi, Avani C.] Univ Cincinnati, Sch Med, Cincinnati Childrens Hosp Med Ctr, Cincinnati, OH 45221 USA.
[Newton, Charles R.] KEMRI Wellcome Programme, Kilifi, Kenya.
[Newton, Charles R.] Univ Oxford, Dept Psychiat, Oxford, England.
[Patel, Archana A.] Harvard Med Sch, Boston Childrens Hosp, Dept Neurol, Div Epilepsy & Clin Neurophysiol, Boston, MA 02115 USA.
[Pennell, Page B.] Harvard Med Sch, Brigham & Womens Hosp, Boston, MA 02115 USA.
[Perucca, Emilio] Univ Pavia, Dept Internal Med & Therapeut, Pavia, Italy.
[Perucca, Emilio] IRCCS Mondino Fdn, ERN EpiCARE, Pavia, Italy.
[Sander, Josemir W.] UCL, Inst Neurol, Queen Sq, London, England.
[Sander, Josemir W.] Stichting Epilepsie Instelligen Nederland SEIN, Heemstede, Netherlands.
[Scheffer, Ingrid E.] Univ Melbourne, Austin Hosp, Florey Inst, Melbourne, Vic, Australia.
[Scheffer, Ingrid E.] Univ Melbourne, Royal Childrens Hosp, Florey Inst, Melbourne, Vic, Australia.

[Scheffer, Ingrid E.] Univ Melbourne, Austin Hosp, Murdoch Childrens Res Inst, Melbourne, Vic, Australia.
[Scheffer, Ingrid E.] Univ Melbourne, Royal Childrens Hosp, Murdoch Childrens Res Inst, Melbourne, Vic, Australia.
[Singh, Gagandeep] Dayanand Med Coll, Ludhiana, Punjab, India.
[Williams, Emma] Matthews Friends Ketogen Dietary Therapies, London, England.
[Wilmshurst, Jo] Red Cross War Mem Childrens Hosp, Neurosci Inst, Dept Paediat Neurol, Cape Town, South Africa.
[Cross, J. Helen] Great Ormond St Hosp Sick Children, UCL NIHR BRC Great Ormond St Inst Child Hlth, London, England.
[Cross, J. Helen] Young Epilepsy, ERN EpiCARE, Lingfield, England.
C3 New York University; Hospital de Pediatria Doctor Juan Garrahan; University of Buenos Aires; Fudan University; Cleveland Clinic Foundation; Icahn School of Medicine at Mount Sinai; University of Miami; Cincinnati Children's Hospital Medical Center; University of Cincinnati; League of European Research Universities - LERU; University of Oxford; Harvard University; Boston Children's Hospital; Harvard Medical School; Harvard University; Brigham & Women's Hospital; Harvard Medical School; University of Pavia; IRCCS Fondazione Casimiro Mondino; University of London; University College London; Florey Institute of Neuroscience & Mental Health; University of Melbourne; Florey Institute of Neuroscience & Mental Health; Royal Children's Hospital Melbourne; University of Melbourne; Florey Institute of Neuroscience & Mental Health; Murdoch Children's Research Institute; University of Melbourne; Murdoch Children's Research Institute; Royal Children's Hospital Melbourne; University of Melbourne; Dayanand Medical College & Hospital; University of London; University College London; Great Ormond Street Hospital for Children NHS Foundation Trust
RP French, JA (通讯作者), NYU, Dept Neurol, NYU Grossman Sch Med, New York, NY 10003 USA.
EM Jacqueline.french@nyulangone.org
TC 81
Z9 82
PD JUN 9
PY 2020
VL 94
IS 23
BP 1032
EP 1037
DI 10.1212/WNL.0000000000009632
WC Clinical Neurology
ER

PT J
AU Grinspan, ZM
Mytinger, JR
Baumer, FM

Ciliberto, MA
Cohen, BH
Dlugos, DJ
Harini, C
Hussain, SA
Joshi, SM
Keator, CG
Knupp, KG
McGoldrick, PE
Nickels, KC
Park, JT
Pasupuleti, A
Patel, AD
Shahid, AM
Shellhaas, RA
Shrey, DW
Singh, RK
Wolf, SM
Yozawitz, EG
Yuskaitis, CJ
Waugh, JL
Pearl, PL
AF Grinspan, Zachary M.
Mytinger, John R.
Baumer, Fiona M.
Ciliberto, Michael A.
Cohen, Bruce H.
Dlugos, Dennis J.
Harini, Chellamani
Hussain, Shaun A.
Joshi, Sucheta M.
Keator, Cynthia G.
Knupp, Kelly G.
McGoldrick, Patricia E.
Nickels, Katherine C.
Park, Jun T.
Pasupuleti, Archana
Patel, Anup D.
Shahid, Asim M.
Shellhaas, Renee A.
Shrey, Daniel W.
Singh, Rani K.
Wolf, Steven M.
Yozawitz, Elissa G.
Yuskaitis, Christopher J.
Waugh, Jeff L.
Pearl, Phillip L.

CA Child Neurology Soc
Practice Comm Executive Board
Pediat Epilepsy Res Comm
Infantile Spasms Special Interest
Steering Comm
TI Management of Infantile Spasms During the COVID-19 Pandemic
SO JOURNAL OF CHILD NEUROLOGY
LA English
DT Article
DE infantile spasm; West syndrome; antiepileptic drug; EEG; electroencephalography
ID CARDIAC RHABDOMYOMA; TREATMENT RESPONSE; EPILEPSY SYNDROMES; MEDICAL-TREATMENT; AMERICAN-ACADEMY; WEST-SYNDROME; NEUROLOGY; CHILDREN; TOPIRAMATE; MULTICENTER
AB Circumstances of the COVID-19 pandemic have mandated a change to standard management of infantile spasms. On April 6, 2020, the Child Neurology Society issued an online statement of immediate recommendations to streamline diagnosis and treatment of infantile spasms with utilization of telemedicine, outpatient studies, and selection of first-line oral therapies as initial treatment. The rationale for the recommendations and specific guidance including follow-up assessment are provided in this manuscript. These recommendations are indicated as enduring if intended to outlast the pandemic, and limited if intended only for the pandemic health care crisis but may be applicable to future disruptions of health care delivery.
C1 [Grinspan, Zachary M.] Weill Cornell Med, New York, NY USA.
[Mytinger, John R.; Patel, Anup D.] Nationwide Childrens Hosp, Columbus, OH USA.
[Baumer, Fiona M.] Stanford Univ, Sch Med, Palo Alto, CA 94304 USA.
[Ciliberto, Michael A.] Univ Iowa Hosp, Iowa City, IA USA.
[Cohen, Bruce H.] Childrens Hosp, Med Ctr Akron, Akron, OH 44308 USA.
[Dlugos, Dennis J.] Childrens Hosp Philadelphia, Philadelphia, PA 19104 USA.
[Harini, Chellamani; Yuskaitis, Christopher J.; Pearl, Phillip L.] Boston Childrens Hosp, Dept Neurol, 300 Longwood Ave, Boston, MA 02115 USA.
[Hussain, Shaun A.] Univ Calif Los Angeles, Mattel Childrens Hosp, Los Angeles, CA USA.
[Joshi, Sucheta M.; Shellhaas, Renee A.] Univ Michigan Pediat Neurol, Ann Arbor, MI USA.
[Keator, Cynthia G.] Cook Childrens Med Ctr, Ft Worth, TX USA.
[Knupp, Kelly G.] Childrens Hosp Colorado, Aurora, CO USA.
[McGoldrick, Patricia E.; Wolf, Steven M.] Boston Childrens Hlth Phys, Hartsdale, NY USA.
[Nickels, Katherine C.] Mayo Clin, Rochester, MN USA.
[Park, Jun T.; Shahid, Asim M.] Univ Hosp Rainbow Babies & Childrens Hosp, Cleveland, OH USA.
[Pasupuleti, Archana] Childrens Natl Hosp, Washington, DC USA.
[Shrey, Daniel W.] Childrens Hosp Orange Cty, Orange, CA 92668 USA.
[Singh, Rani K.] Levine Childrens Hosp Atrium Hlth Syst, Charlotte, NC USA.
[Yozawitz, Elissa G.] Montefiore Med Syst, Bronx, NY USA.
[Waugh, Jeff L.] Univ Texas Southwestern Med Ctr Southwestern, Dallas, TX USA.
C3 Cornell University; Ohio State University; Stanford University;

University of Iowa; Akron Children's Hospital; University of Pennsylvania; Childrens Hospital of Philadelphia; Harvard University; Boston Children's Hospital; University of California System; University of California Los Angeles; Cook Children's Medical Center; Children's Hospital Colorado; Mayo Clinic; Case Western Reserve University; Case Western Reserve University Hospital; University Hospitals of Cleveland; Rainbow Babies & Children's Hospital; Children's National Health System; Childrens Hospital of Orange County; Montefiore Medical Center

RP Pearl, PL (通讯作者), Boston Childrens Hosp, Dept Neurol, 300 Longwood Ave, Boston, MA 02115 USA.

EM phillip.pearl@childrens.harvard.edu

TC 15

Z9 15

PD OCT

PY 2020

VL 35

IS 12

BP 828

EP 834

AR 0883073820933739

DI 10.1177/0883073820933739

EA JUN 2020

WC Clinical Neurology; Pediatrics

ER

PT J

AU Bhaskar, S
Bradley, S
Israeli-Korn, S
Menon, B
Chattu, VK
Thomas, P
Chawla, J
Kumar, R
Prandi, P
Ray, D
Golla, S
Surya, N
Yang, H
Martinez, S
Ozgen, MH
Codrington, J
Gonzalez, EMJ
Toosi, M
Mohan, NH
Menon, KV
Chahidi, A

Hengstl, SM
AF Bhaskar, Sonu
Bradley, Sian
Israeli-Korn, Simon
Menon, Bindu
Chattu, Vijay Kumar
Thomas, Pravin
Chawla, Jasvinder
Kumar, Rajeev
Prandi, Paolo
Ray, Daniel
Golla, Sailaja
Surya, Nirmal
Yang, Harvey
Martinez, Sandra
Ozgen, Mihriban Heval
Codrington, John
Jimenez Gonzalez, Eva Maria
Toosi, Mandana
Mohan, Nithya Hariya
Menon, Koravangattu Valsraj
Chahidi, Abderrahmane
Mederer Hengstl, Susana
TI Chronic Neurology in COVID-19 Era: Clinical Considerations and
Recommendations From the REPROGRAM Consortium
SO FRONTIERS IN NEUROLOGY
LA English
DT Article
DE COVID-19; chronic neurological disease; healthcare; guideline; recommendation;
neurodegenerative disorder; protocol
ID MIGRAINE
AB With the rapid pace and scale of the emerging coronavirus 2019 (COVID-19) pandemic, a growing body of evidence has shown a strong association of COVID-19 with pre- and post- neurological complications. This has necessitated the need to incorporate targeted neurological care for this subgroup of patients which warrants further reorganization of services, healthcare workforce, and ongoing management of chronic neurological cases. The social distancing and the shutdown imposed by several nations in the midst of COVID-19 have severely impacted the ongoing care, access and support of patients with chronic neurological conditions such as Multiple Sclerosis, Epilepsy, Neuromuscular Disorders, Migraine, Dementia, and Parkinson disease. There is a pressing need for governing bodies including national and international professional associations, health ministries and health institutions to harmonize policies, guidelines, and recommendations relating to the management of chronic neurological conditions. These harmonized guidelines should ensure patient continuity across the spectrum of hospital and community care including the well-being, safety, and mental health of the patients, their care partners and the health professionals involved. This article provides an in-depth analysis of the impact of COVID-19 on chronic neurological

conditions and specific recommendations to minimize the potential harm to those at high risk.

C1 [Bhaskar, Sonu; Bradley, Sian; Israeli-Korn, Simon; Menon, Bindu; Chattu, Vijay Kumar; Thomas, Pravin; Chawla, Jasvinder; Kumar, Rajeev; Prandi, Paolo; Ray, Daniel; Golla, Sailaja; Surya, Nirmal; Yang, Harvey; Martinez, Sandra; Ozgen, Mihriban Heval; Codrington, John; Jimenez Gonzalez, Eva Maria; Toosi, Mandana; Mohan, Nithya Hariya; Menon, Koravangattu Valsraj; Chahidi, Abderrahmane; Mederer Hengstl, Susana] Liverpool Hosp, Chron Neurol REPROGRAM Subcomm, Pandem Hlth Syst REsilience PROGRAM REPROGRAM Con, Sydney, NSW, Australia.

[Bhaskar, Sonu] Liverpool Hosp, Dept Neurol & Neurophysiol, Sydney, NSW, Australia.

[Bhaskar, Sonu] Ingham Inst Appl Med Res, Neurovasc Imaging Lab, Sydney, NSW, Australia.

[Bhaskar, Sonu] Ingham Inst Appl Med Res, NSW Brain Clot Bank, Sydney, NSW, Australia.

[Bhaskar, Sonu] Univ New South Wales, UNSW Med, South West Sydney Clin Sch, Sydney, NSW, Australia.

[Bradley, Sian] Univ New South Wales, UNSW Med, Sydney, NSW, Australia.

[Israeli-Korn, Simon] Sheba Med Ctr, Dept Neurol, Ramat Gan, Israel.

[Israeli-Korn, Simon] Tel Aviv Univ, Sackler Sch Med, Movement Disorders Inst, Tel Aviv, Israel.

[Menon, Bindu] Apollo Hosp, Dept Neurol, Nellore, India.

[Chattu, Vijay Kumar] Univ Toronto, St Michaels Hosp, Dept Med, Toronto, ON, Canada.

[Thomas, Pravin] Univ Hosp NHS Fdn Trust, Dept Neurol, Birmingham, W Midlands, England.

[Chawla, Jasvinder] Loyola Univ Med Ctr, Dept Neurol, Chicago, IL USA.

[Chawla, Jasvinder] Hines VA Hosp, Chicago, IL USA.

[Kumar, Rajeev] Hamad Med Ctr, Dept Psychiat, Doha, Qatar.

[Kumar, Rajeev] Australian Natl Univ, Canberra, ACT, Australia.

[Prandi, Paolo] Univ Eastern Piedmont Amedeo Avogadro, Dept Neurol, Novara, Italy.

[Ray, Daniel] UCL, Farr Inst Hlth Informat, London, England.

[Ray, Daniel] NHS Fdn Trust, Birmingham, W Midlands, England.

[Golla, Sailaja] Texas Inst Neurol Disorders, Dallas, TX USA.

[Surya, Nirmal] Bombay Hosp & Med Res Ctr, Dept Neurol, Mumbai, Maharashtra, India.

[Surya, Nirmal] Epilepsy Fdn India, Mumbai, Maharashtra, India.

[Yang, Harvey] Acad Hosp Paramaribo, Dept Neurol, Paramaribo, Suriname.

[Yang, Harvey; Codrington, John] Anton de Kom Univ Suriname Fac Med Wetenschappen, Paramaribo, Suriname.

[Martinez, Sandra] Hosp Restauracao, Dept Neurol, Recife, PE, Brazil.

[Ozgen, Mihriban Heval] Parnassia Psychiat Inst, Dept Psychiat, The Hague, Netherlands.

[Ozgen, Mihriban Heval] Leiden Univ, Med Ctr, Curium, Oegstgeest, Netherlands.

[Codrington, John] Acad Hosp Paramaribo, Dept Lab Med, Paramaribo, Suriname.

[Jimenez Gonzalez, Eva Maria] Minist Justice, Dept Forens Psychol, Forens Psychol & Forens Sci Inst, Granada, Spain.

[Toosi, Mandana] LodeStone Ctr Behav Hlth, Chicago, IL USA.

[Toosi, Mandana] Eastern Illinois Univ, Chicago, IL USA.

[Mohan, Nithya Hariya] Chengalpattu Med Coll & Hosp, Chengalpattu, India.

[Menon, Koravangattu Valsraj] South London & Maudsley NHS Fdn Trust, Dept Psychiat, Kings Hlth Partners, London, England.
[Chahidi, Abderrahmane] Sorbonne Nouvelle Univ, DR 178, ED 268, Paris, France.
[Chahidi, Abderrahmane] Moroccan Soc Neurophysiol, Marrakech, Morocco.
[Chahidi, Abderrahmane] Univ Med Sch Marrakech, Morocco & Basic & Clin Neurosci Res Lab, Marrakech, Morocco.
[Mederer Hengstl, Susana] Complejo Hosp Pontevedra, Dept Neurol, Pontevedra, Spain.
C3 Liverpool Hospital; Liverpool Hospital; Ingham Institute for Applied Medical Research; Ingham Institute for Applied Medical Research; University of New South Wales Sydney; University of New South Wales Sydney; Chaim Sheba Medical Center; Shamir Medical Center (Assaf Harofeh); Tel Aviv University; Sackler Faculty of Medicine; University of Toronto; Saint Michaels Hospital Toronto; US Department of Veterans Affairs; Veterans Health Administration (VHA); Edward Hines Jr. VA Hospital; Australian National University; University of Eastern Piedmont Amedeo Avogadro; University of London; University College London; Oxford University Hospitals NHS Foundation Trust; Bombay Hospital & Medical Research Centre; Parnassia Psychiatric Institute; League of European Research Universities - LERU; Leiden University; Leiden University Medical Center (LUMC); Leiden University - Excl LUMC; South London & Maudsley NHS Trust; University of London; King's College London; Complexo Hospitalario Universitario de Pontevedra
RP Bhaskar, S (通讯作者), Liverpool Hosp, Chron Neurol REPROGRAM Subcomm, Pandem Hlth Syst REsilience PROGRAM REPROGRAM Con, Sydney, NSW, Australia.; Bhaskar, S (通讯作者), Liverpool Hosp, Dept Neurol & Neurophysiol, Sydney, NSW, Australia.
EM sonu.bhaskar@health.nsw.gov.au
TC 28
Z9 29
PD JUN 24
PY 2020
VL 11
AR 664
DI 10.3389/fneur.2020.00664
WC Clinical Neurology; Neurosciences
ER

PT J
AU Elgamasy, S
Kamel, MG
Ghozy, S
Khalil, A
Morra, ME
Islam, SMS
AF Elgamasy, Sara
Kamel, Mohamed G.
Ghozy, Sherief
Khalil, Adham

Morra, Mostafa E.
Islam, Sheikh M. S.
TI First case of focal epilepsy associated with SARS-coronavirus-2
SO JOURNAL OF MEDICAL VIROLOGY
LA English
DT Article
DE case report; coronavirus; COVID-19; epilepsy; infection; outbreak
ID SEIZURES
AB A healthy patient presented to Klinikum Altmuhlfranken Weissenburg Hospital, Germany, with two morning attacks of painful muscle spasm in the left upper and lower limbs, without altered consciousness. Full examinations, radiological imaging, electroencephalography, lumbar puncture, and autoimmune profile were either normal or not consistent with patient's complaint. Subsequent epileptic episodes were observed on admission day and the following days; thus, the patient was diagnosed with focal epilepsy. The patient started to develop a fever and severe cough on day 4, and SARS-coronavirus-2 was confirmed through a nasopharyngeal swap. She received anticonvulsants and symptomatic treatments and completely recovered. This report emphasizes the potential nervous system involvement in severe acute respiratory syndrome-coronavirus-2 pathogenesis.
C1 [Elgamasy, Sara] Klinikum Altmuhlfranken Weissenburg, Dept Internal Med, Bayern, Germany.
[Kamel, Mohamed G.] Minia Univ, Fac Med, Al Minya, Egypt.
[Ghozy, Sherief] Mansoura Univ, Fac Med, Mansoura, Egypt.
[Ghozy, Sherief] El Sheikh Zayed Specialized Hosp, Neurosurg Dept, Giza, Egypt.
[Khalil, Adham] Zagazig Univ, Fac Med, Zagazig, Egypt.
[Morra, Mostafa E.] Al Azhar Univ, Fac Med, Cairo 11884, Egypt.
[Islam, Sheikh M. S.] Deakin Univ, Sch Exercise & Nutr Sci, Inst Phys Act & Nutr IPAN, Melbourne, Vic, Australia.
C3 Egyptian Knowledge Bank (EKB); Minia University; Egyptian Knowledge Bank (EKB); Mansoura University; Egyptian Knowledge Bank (EKB); Zagazig University; Egyptian Knowledge Bank (EKB); Al Azhar University; Deakin University
RP Morra, ME (通讯作者), Al Azhar Univ, Fac Med, Cairo 11884, Egypt.; Islam, SMS (通讯作者), Deakin Univ, Sch Exercise & Nutr Sci, Inst Phys Act & Nutr IPAN, Melbourne, Vic, Australia.
EM mostafamorra.stu.6@azhar.edu.eg; shariful.islam@deakin.edu.au
TC 26
Z9 29
PD OCT
PY 2020
VL 92
IS 10
BP 2238
EP 2242
DI 10.1002/jmv.26113
EA JUN 2020
WC Virology

ER

PT J
AU Kuroda, N
AF Kuroda, Naoto
TI Decision Making on Telemedicine for Patients With Epilepsy During the
   Coronavirus Disease 2019 (COVID-19) Crisis
SO FRONTIERS IN NEUROLOGY
LA English
DT Editorial Material
DE epilepsy; COVID-19; telemedicine; decision making; algorithm; surgery;
   novel coronavirus
C1 [Kuroda, Naoto] Wayne State Univ, Dept Pediat, Childrens Hosp Michigan, Detroit,
MI 48202 USA.
C3 Children's Hospital of Michigan; Wayne State University
RP Kuroda, N (通讯作者), Wayne State Univ, Dept Pediat, Childrens Hosp Michigan, Detroit,
MI 48202 USA.
EM naoto.kuroda@wayne.edu
TC 14
Z9 14
PD JUN 26
PY 2020
VL 11
AR 722
DI 10.3389/fneur.2020.00722
WC Clinical Neurology; Neurosciences
ER

PT J
AU Asadi-Pooya, AA
   Attar, A
   Moghadami, M
   Karimzadeh, I
AF Asadi-Pooya, Ali A.
   Attar, Armin
   Moghadami, Mohsen
   Karimzadeh, Iman
TI Management of COVID-19 in people with epilepsy: drug considerations
SO NEUROLOGICAL SCIENCES
LA English
DT Article
DE Antiepileptic drug; COVID-19; Epilepsy; Interaction; Seizure; Management
ID CONDUCTION
AB People with epilepsy (PWE) are neither more likely to be infected by the coronavirus nor are they more likely to have severe COVID-19 manifestations because they suffer from epilepsy. However, management of COVID-19 in PWE may be more complicated than that in other individuals. Drug-drug interactions could pose significant challenges and

cardiac, hepatic, or renal problems, which may happen in patients with severe COVID-19, may require adjustment to antiepileptic drugs (AEDs). In this review, we first summarize the potential drug-drug interactions between AEDs and drugs currently used in the management of COVID-19. We then summarize other challenging issues that may happen in PWE, who have COVID-19 and are receiving treatment.
C1 [Asadi-Pooya, Ali A.] Shiraz Univ Med Sci, Epilepsy Res Ctr, Shiraz, Iran.
[Asadi-Pooya, Ali A.] Thomas Jefferson Univ, Dept Neurol, Jefferson Comprehens Epilepsy Ctr, Philadelphia, PA 19107 USA.
[Attar, Armin] Shiraz Univ Med Sci, Dept Cardiovasc Med, Shiraz, Iran.
[Moghadami, Mohsen] Shiraz Univ Med Sci, Inst Hlth, Hlth Policy Res Ctr, Shiraz, Iran.
[Karimzadeh, Iman] Shiraz Univ Med Sci, Dept Clin Pharm, Shiraz, Iran.
C3 Shiraz University of Medical Science; Jefferson University; Shiraz University of Medical Science; Shiraz University of Medical Science; Shiraz University of Medical Science
RP Asadi-Pooya, AA (通讯作者), Shiraz Univ Med Sci, Epilepsy Res Ctr, Shiraz, Iran.; Asadi-Pooya, AA (通讯作者), Thomas Jefferson Univ, Dept Neurol, Jefferson Comprehens Epilepsy Ctr, Philadelphia, PA 19107 USA.
EM aliasadipooya@yahoo.com; attar_armin@yahoo.com; mohsen168@gmail.com; karimzadehiman@yahoo.com
TC 25
Z9 25
PD AUG
PY 2020
VL 41
IS 8
BP 2005
EP 2011
DI 10.1007/s10072-020-04549-5
EA JUN 2020
WC Clinical Neurology; Neurosciences
ER

PT J
AU Alotaibi, F
Althani, Z
Aljaafari, D
Tayeb, HO
Baarmah, D
Aljalal, N
Muthaffar, O
Alqulaiti, K
Alhameed, M
Ali, B
Qureshi, S
Albaradie, R
AF Alotaibi, Faisal

Althani, Ziyad
Aljaafari, Danah
Tayeb, Haythum O.
Baarmah, Duaa
Aljalal, Norah
Muthaffar, Osama
Alqulaiti, Khalid
Alhameed, Majed
Ali, Baleegh
Qureshi, Shireen
Albaradie, Raidah
TI Saudi Epilepsy Society consensus on epilepsy management during the COVID-19 Pandemic
SO NEUROSCIENCES
LA English
DT Article
DE management; COVID-19; Epilepsy
AB Coronavirus disease 2019 (COVID-19) emerged in late 2019 in Wuhan, China.(1) The outbreak was confirmed as a pandemic on February 11, 2020, by the World Health Organization (WHO). By the end of April 2020, more than 3.5 million people were infected worldwide. The number of affected people in Saudi Arabia has reached more than 28,000 at the time of this report's submission. Since the early phase of the novel coronavirus epidemic, the Ministry of Health (MOH) in Saudi Arabia has been working to develop strict measures to contain the virus spread, going as far as locking down certain cities and restricting movement within and between others. The pandemic has affected all medical specialists' ability to practice efficiently and has resulted in a major change in the clinical pathways of managing people with epilepsy worldwide.(1) The Saudi Epilepsy Society (SES), in collaboration with the Saudi Patient Safety Center, has initiated a priority care classification in epilepsy management practice. In addition, the SES COVID-19 task force has established a consensus for the best evidence-based practice related to epilepsy during the pandemic.
C1 [Alotaibi, Faisal] King Faisal Specialist Hosp & Res Ctr, Dept Neurosci, Riyadh, Saudi Arabia.
[Baarmah, Duaa] Minist Natl Guard, Dept Pediat, Div Neurol, King Abdullah Specialized Children Hosp, Riyadh, Saudi Arabia.
[Alhameed, Majed] King Fahad Med City, Natl Neurosci Inst, Dept Neurol, Riyadh, Saudi Arabia.
[Ali, Baleegh] King Saud Med City, Dept Pediat Neurol, Riyadh, Saudi Arabia.
[Althani, Ziyad] King Fahad Specialist Hosp, Neurosci Ctr, Riyadh, Saudi Arabia.
[Aljaafari, Danah] Imam Abdulrahman Bin Faisal Univ, Coll Med, Dept Neurol, Dammam, Saudi Arabia.
[Tayeb, Haythum O.] King Abdulaziz Univ, Coll Med, Jeddah, Saudi Arabia.
[Muthaffar, Osama] King Abdulaziz Univ, Dept Pediat, Jeddah, Saudi Arabia.
[Alqulaiti, Khalid] Taibah Univ, Coll Med, Al Munawarrah, Saudi Arabia.
C3 King Faisal Specialist Hospital & Research Center; King Saud Bin Abdulaziz University for Health Sciences; King Abdullah Specialist Childrens Hospital (KASCH); Ministry of National Guard - Health Affairs;

King Fahad Medical City; King Saud Medical City; Imam Abdulrahman Bin Faisal University; King Abdulaziz University; King Abdulaziz University; Taibah University
RP Alotaibi, F (通讯作者), King Faisal Specialist Hosp & Res Ctr, Dept Neurosci, Riyadh, Saudi Arabia.
EM faisalruwais@gmail.com
TC 1
Z9 1
PD JUL
PY 2020
VL 25
IS 3
BP 222
EP 225
DI 10.17712/nsj.2020.3.20200066
WC Clinical Neurology
ER

PT J
AU Adan, GH
Mitchell, JW
Marson, T
AF Adan, Guleed H.
Mitchell, James W.
Marson, Tony
TI Epilepsy care in the COVID-19 era
SO CLINICAL MEDICINE
LA English
DT Article
DE COVID-19; epilepsy; seizure; telemedicine; Epilepsy care
ID ACCESS
AB The COVID-19 pandemic will impact on how care for chronic conditions is delivered. We use epilepsy to exemplify how care for patients will be affected, and suggest ways in which healthcare systems can respond to deliver the most effective care. Where face-to-face outpatient appointments have been cancelled, telemedicine can facilitate remote clinical consultations for new and follow-up epilepsy clinic patients while reducing the risk of infection to both patients and healthcare staff. First-seizure patients will need investigation pathways rationalised, while those with chronic epilepsy will need to have reliable alternative avenues to access clinical advice. At the same time, neurologists should support emergency departments and acute medical units, advising on appropriate management of seizures and other acute neurological presentations. Ultimately, the revolution in our clinical practice is unlikely to cease after this pandemic, with reconfiguration of services likely to bring improvements in efficiency and convenience, and a reduced environmental impact.
C1 [Adan, Guleed H.; Mitchell, James W.] Inst Syst Mol & Integrat Biol, Liverpool, Merseyside, England.
[Adan, Guleed H.; Mitchell, James W.; Marson, Tony] Walton Ctr NHS Fdn Trust,

Liverpool, Merseyside, England.
[Marson, Tony] Inst Syst Mol & Integrat Biol, Neurol, Liverpool, Merseyside, England.
C3 Walton Centre
RP Adan, GH (通讯作者), Univ Liverpool, Clin Sci Ctr, Lower Lane, Liverpool L9 7LJ, Merseyside, England.
EM guleed.adan@liverpool.ac.uk
TC 20
Z9 21
PD JUL
PY 2020
VL 20
IS 4
BP E104
EP E106
DI 10.7861/clinmed.2020-0207
WC Medicine, General & Internal
ER

PT J
AU Kuroda, N
AF Kuroda, Naoto
TI Epilepsy and COVID-19: Associations and important considerations
SO EPILEPSY & BEHAVIOR
LA English
DT Letter
ID SUDDEN UNEXPECTED DEATH; RISK
DE COVID-19; Epilepsy
C1 [Kuroda, Naoto] Wayne State Univ, Dept Pediat, Childrens Hosp Michigan, Detroit, MI 48202 USA.
[Kuroda, Naoto] Wayne State Univ, Detroit Med Ctr, Childrens Hosp Michigan, Dept Pediat, 3901 Beaubien St, Detroit, MI 48201 USA.
C3 Children's Hospital of Michigan; Wayne State University; Children's Hospital of Michigan; Detroit Medical Center; Wayne State University
RP Kuroda, N (通讯作者), Wayne State Univ, Dept Pediat, Childrens Hosp Michigan, Detroit, MI 48202 USA.; Kuroda, N (通讯作者), Wayne State Univ, Detroit Med Ctr, Childrens Hosp Michigan, Dept Pediat, 3901 Beaubien St, Detroit, MI 48201 USA.
EM naoto.kuroda@wayne.edu
TC 50
Z9 53
PD JUL
PY 2020
VL 108
AR 107122
DI 10.1016/j.yebeh.2020.107122
WC Behavioral Sciences; Clinical Neurology; Psychiatry
ER

PT J
AU Assenza, G
   Lanzone, J
   Brigo, F
   Coppola, A
   Di Gennaro, G
   Di Lazzaro, V
   Ricci, L
   Romigi, A
   Tombini, M
   Mecarelli, O
AF Assenza, Giovanni
   Lanzone, Jacopo
   Brigo, Francesco
   Coppola, Antonietta
   Di Gennaro, Giancarlo
   Di Lazzaro, Vincenzo
   Ricci, Lorenzo
   Romigi, Andrea
   Tombini, Mario
   Mecarelli, Oriano
TI Epilepsy Care in the Time of COVID-19 Pandemic in Italy: Risk Factors
   for Seizure Worsening
SO FRONTIERS IN NEUROLOGY
LA English
DT Article
DE epilepsy; COVID-19; Epilepsy care; Risk factor
ID GENERALIZED ANXIETY DISORDER; SLEEP QUALITY INDEX; OVERTREATMENT;
   EPIDEMIOLOGY; DEPRIVATION; PREVALENCE; COMMUNITY; PEOPLE; AGE
AB Objective:In early 2020, Italy struggled with an unprecedented health emergency related to the COVID-19 pandemic. Medical care of chronic neurological diseases, such as epilepsy, is being sorely neglected. In this national survey, we aimed at understanding the impact of COVID-19 lockdown on the care of people with epilepsy (PwE) and identifying PwE risk factors for seizure worsening to direct telemedicine efforts. Methods:We administered a 48-items online survey (published on April 11, 2020) including socio-demographic, epilepsy-related, and psychometric variables (BDI-II for depression, GAD-7 for anxiety, and PSQI for sleep) to PwE and people without epilepsy (PwoE). Regression analysis identified predictors of seizure worsening. Results:We collected responses from 456 PwE (344 females) and 472 PwoE (347 females). Outpatient examinations of PwE were postponed in 95% of cases. One-third of PwE complained of issues with epilepsy management, but only 71% of them reached the treating physician and solved their problems. PwE had worse depressive and anxiety symptoms (higher BDI-II and GAD-7 scores;p< 0.001) than PwoE. Sleep quality was equally compromised in both groups (47 and 42%). Sixty-seven PwE (18%) reported seizure worsening, which was best explained by the number of anti-seizure medications (ASM) of chronic therapy and the severity of sleep disorder. Conclusions:During the current COVID-19 pandemic, a significant

percentage of PwE experienced difficulties in follow-up and a seizure number increase, in particular those chronically taking more ASMs and with poor sleep quality. This dramatic experience outlines the urgent need for validation and implementation of telemedicine services for epileptic patients in order to provide regular follow-up.
C1 [Assenza, Giovanni; Lanzone, Jacopo; Di Lazzaro, Vincenzo; Ricci, Lorenzo; Tombini, Mario] Univ Campus Biomed Rome, Unit Neurol, Dept Med, Neurophysiol,Neurobiol, Rome, Italy.
[Brigo, Francesco] Franz Tappeiner Hosp, Div Neurol, Merano, Italy.
[Coppola, Antonietta] Univ Federico II Naples, Dept Neurosci Reprod Sci & Odontostomatol, Naples, Italy.
[Di Gennaro, Giancarlo] IRCCS NEUROMED, Epilepsy Surg Ctr, Pozzilli, Italy.
[Romigi, Andrea] IRCCS NEUROMED, Sleep Med Ctr, Pozzilli, Italy.
[Mecarelli, Oriano] Sapienza Univ Rome, Dept Human Neurosci, Rome, Italy.
C3 University Campus Bio-Medico - Rome Italy; Ospedale Franz Tappeiner;
University of Naples Federico II; IRCCS Neuromed; IRCCS Neuromed;
Sapienza University Rome
RP Assenza, G （通讯作者）, Univ Campus Biomed Rome, Unit Neurol, Dept Med, Neurophysiol,Neurobiol, Rome, Italy.
EM g.assenza@unicampus.it
TC 67
Z9 67
PD JUL 3
PY 2020
VL 11
AR 737
DI 10.3389/fneur.2020.00737
WC Clinical Neurology; Neurosciences
ER

PT J
AU Wirrell, EC
Grinspan, ZM
Knupp, KG
Jiang, YW
Hammeed, B
Mytinger, JR
Patel, AD
Nabbout, R
Specchio, N
Cross, JH
Shellhaas, RA
AF Wirrell, Elaine C.
Grinspan, Zachary M.
Knupp, Kelly G.
Jiang, Yuwu
Hammeed, Biju
Mytinger, John R.

Patel, Anup D.
Nabbout, Rima
Specchio, Nicola
Cross, J. Helen
Shellhaas, Renee A.
TI Care Delivery for Children With Epilepsy During the COVID-19 Pandemic: An International Survey of Clinicians
SO JOURNAL OF CHILD NEUROLOGY
LA English
DT Article
DE epilepsy; surgery; telemedicine; Care; Children
ID INFANTILE SPASMS; KETOGENIC DIET; SURGERY; ONSET; MULTICENTER; VIGABATRIN; NEUROLOGY; EFFICACY; SEIZURES; AGE
AB Objective: To evaluate the effect of the COVID-19 pandemic on global access to care and practice patterns for children with epilepsy. Methods: We conducted a cross-sectional, online survey of pediatric neurologists across the world affiliated with the International Child Neurology Association, the Chinese Child Neurology Society, the Child Neurology Society, and the Pediatric Epilepsy Research Consortium. Results were analyzed in relation to regional burden of COVID-19 disease. Results: From April 10 to 24, 2020, a sample of 212 respondents from 49 countries indicated that the COVID-19 pandemic has dramatically changed many aspects of pediatric epilepsy care, with 91.5% reporting changes to outpatient care, 90.6% with reduced access to electroencephalography (EEG), 37.4% with altered management of infantile spasms, 92.3% with restrictions in ketogenic diet initiation, 93.4% with closed or severely limited epilepsy monitoring units, and 91.3% with canceled or limited epilepsy surgery. Telehealth use had increased, with 24.7% seeing patients exclusively via telehealth. Changes in practice were related both to COVID-19 burden and location. Conclusions: In response to COVID-19, pediatric epilepsy programs have implemented crisis standards of care that include increased telemedicine, decreased EEG use, changes in treatments of infantile spasms, and cessation of epilepsy surgery. The long-term impact of these abrupt changes merit careful study.
C1 [Wirrell, Elaine C.] Mayo Clin, Dept Neurol, Div Child & Adolescent Neurol, Rochester, MN USA.
[Wirrell, Elaine C.] Mayo Clin, Dept Neurol, Div Epilepsy, Rochester, MN USA.
[Grinspan, Zachary M.] Weill Cornell Med, Dept Populat Sci, New York, NY USA.
[Grinspan, Zachary M.] Weill Cornell Med, Dept Pediat, New York, NY USA.
[Knupp, Kelly G.] Univ Colorado, Dept Pediat & Neurol, Anschutz Med Campus, Aurora, CO USA.
[Jiang, Yuwu] Peking Univ, Dept Pediat, Hosp 1, Beijing, Peoples R China.
[Hammeed, Biju] Great Ormond St Childrens Hosp, Paediat Neurosci, London, England.
[Mytinger, John R.; Patel, Anup D.] Ohio State Univ, Dept Pediat, Nationwide Childrens Hosp, Div Pediat Neurol, Columbus, OH 43210 USA.
[Nabbout, Rima] Paris Descartes Univ, Imagine Inst, Necker Enfants Malad Hosp, Ctr Reference Epilepsies Rares,Dept Pediat Neurol, Paris, France.
[Specchio, Nicola] IRCCS, Bambino Gesu Childrens Hosp, Dept Neurosci, Rare & Complex Epilepsy Unit, Rome, Italy.
[Specchio, Nicola; Cross, J. Helen] European Reference Network EpiCARE, London,

England.
[Cross, J. Helen] UCL NIHR BRC Great Ormond St Inst Child Hlth, Dev Neurosci, London WC1N 1EH, England.
[Shellhaas, Renee A.] Univ Michigan, Michigan Med, Dept Pediat Pediat Neurol, Ann Arbor, MI 48109 USA.
C3 Mayo Clinic; Mayo Clinic; Cornell University; Cornell University;
University of Colorado System; University of Colorado Anschutz Medical
Campus; Peking University; University of London; University College
London; Great Ormond Street Hospital for Children NHS Foundation Trust;
Ohio State University; Assistance Publique Hopitaux Paris (APHP);
Hopital Universitaire Necker-Enfants Malades - APHP; UDICE-French
Research Universities; Universite de Paris; IRCCS Bambino Gesu;
University of Michigan System; University of Michigan
RP Shellhaas, RA (通讯作者), CS Mott Childrens Hosp, Room 12-733,1540 E Hosp Dr, Ann Arbor, MI 48109 USA.
EM shellhaa@med.umich.edu
TC 23
Z9 23
PD NOV
PY 2020
VL 35
IS 13
BP 924
EP 933
AR 0883073820940189
DI 10.1177/0883073820940189
EA JUL 2020
WC Clinical Neurology; Pediatrics
ER

PT J
AU Grippo, A
Assenza, G
Scarpino, M
Broglia, L
Cilea, R
Galimberti, CA
Lanzo, G
Michelucci, R
Tassi, L
Vergari, M
Di Lazzaro, V
Mecarelli, O
AF Grippo, Antonello
Assenza, Giovanni
Scarpino, Maenia
Broglia, Lidia

Cilea, Rosalia
Galimberti, Carlo Andrea
Lanzo, Giovanni
Michelucci, Roberto
Tassi, Laura
Vergari, Maurizio
Di Lazzaro, Vincenzo
Mecarelli, Oriano
CA SINC
LICE
AITN
TI Electroencephalography during SARS-CoV-2 outbreak: practical recommendations from the task force of the Italian Society of Neurophysiology (SINC), the Italian League Against Epilepsy (LICE), and the Italian Association of Neurophysiology Technologists (AITN)
SO NEUROLOGICAL SCIENCES
LA English
DT Article
DE EEG; COVID-19; Electroencephalography; Recommendation
AB Background During COVID-19 lockdown, non-urgent medical procedures were suspended. Grade of urgency of electroencephalography (EEG) may vary according to the clinical indication, setting, and status of infection of SARS-CoV-2 virus. "Italian Society of Clinical Neurophysiology" (SINC), "Italian League Against Epilepsy" (LICE), and the "Italian Association of Neurophysiology Technologists" (AITN) aimed to provide clinical and technical recommendation for EEG indications and recording standards in this pandemic era. Methods Presidents of SINC, LICE, and AITN endorsed three members per each society to formulate recommendations: classification of the degree of urgency of EEG clinical indications, management and behavior of physicians and neurophysiology technologists, hygiene and personal protection standards, and use of technical equipment. Results Scientific societies endorsed a paper conveying the recommendation for EEG execution in accordance with clinical urgency, setting (inpatients/outpatients), status of SARS-CoV-2 virus infection (positive, negative and uncertain), and phase of governmental restrictions (phase 1 and 2). Briefly, in phase 1, EEG was recommended only for those acute/subacute neurological symptoms where EEG is necessary for diagnosis, prognosis, or therapy. Outpatient examinations should be avoided in phase 1, while they should be recommended in urgent cases in phase 2 when they could prevent an emergency room access. Reduction of staff contacts must be encouraged through rescheduling job shifts. The use of disposable electrodes and dedicated EEG devices for COVID-19-positive patients are recommended. Conclusions During the different phases of COVID-19 pandemic, the EEG should be reserved for patients really benefiting from its execution in terms of diagnosis, treatment, prognosis, and avoidance of emergency room access.
C1 [Grippo, Antonello; Scarpino, Maenia; Lanzo, Giovanni] AOU Careggi, SODc Neurophysiopathol, Florence, Italy.
[Grippo, Antonello; Scarpino, Maenia] IRCCS Don Carlo Gnocchi Fdn, Florence, Italy.
[Assenza, Giovanni; Di Lazzaro, Vincenzo] Univ Campus Biomed Rome, Dept Med, Unit Neurol Neurophysiol Neurobiol, Via Alvaro del Fortino 200, I-00128 Rome, Italy.

[Broglia, Lidia] San Filippo Neri Hosp, ASL Rome 1, UOC Neurol, Rome, Italy.
[Cilea, Rosalia; Michelucci, Roberto] IRCSS Neurol Sci, Bologna, Italy.
[Galimberti, Carlo Andrea] IRCCS Mondino Fdn, Pavia, Italy.
[Tassi, Laura] ASST Niguarda Hosp, Claudio Munari Epilepsy Surg Ctr, Milan, Italy.
[Vergari, Maurizio] Osped Maggiore, IRCCS Fdn Ca Granda, Milan, Italy.
[Mecarelli, Oriano] Sapienza Univ Rome, Dept Human Neurosciencies, Rome, Italy.
C3 University of Florence; Azienda Ospedaliero Universitaria Careggi;
University Campus Bio-Medico - Rome Italy; San Filippo Neri Hospital;
IRCCS Fondazione Casimiro Mondino; IRCCS Ca Granda Ospedale Maggiore
Policlinico; Sapienza University Rome
RP Assenza, G (通讯作者), Univ Campus Biomed Rome, Dept Med, Unit Neurol Neurophysiol Neurobiol, Via Alvaro del Fortino 200, I-00128 Rome, Italy.
EM g.assenza@unicampus.it
TC 14
Z9 14
PD SEP
PY 2020
VL 41
IS 9
BP 2345
EP 2351
DI 10.1007/s10072-020-04585-1
EA JUL 2020
WC Clinical Neurology; Neurosciences
ER

PT J
AU Asadi-Pooya, AA
Farazdaghi, M
Bazrafshan, M
AF Asadi-Pooya, Ali A.
Farazdaghi, Mohsen
Bazrafshan, Mehdi
TI Impacts of the COVID-19 pandemic on Iranian patients with epilepsy
SO ACTA NEUROLOGICA SCANDINAVICA
LA English
DT Editorial Material
DE COVID-19; epilepsy; impact; seizure
ID ECONOMIC SANCTIONS
AB Objective: To investigate the effects of COVID-19 pandemic on patients' perceptions of hardship in obtaining their drugs and if this pandemic and the social restrictions in response to that has resulted in any changes in their seizure control status. We also investigated factors potentially associated with the perceptions of difficulty in obtaining their drugs (eg, polytherapy vs monotherapy, taking imported drugs, and seizure status worsening).

Methods: We surveyed a random sample of patients with epilepsy, who were registered in our database at Shiraz Epilepsy Center, Iran, on their perceptions on two issues:

(a) What has been your experience on obtaining your antiseizure medications in the past 4 weeks (compared to before)? (b) Have you experienced any changes in your seizure control status in the past 4 weeks?
Results: We included 100 patients (53 male and 47 female patients). In response to the question "Have you had any difficulties in the past 4 weeks to obtain your drugs?," 31 people (31%) expressed hardship obtaining their drugs. In response to the question "How has been your seizure control status compared with before?," six people (6%) expressed worsening of their seizure control status in the past 4 weeks. None of the patients reported symptoms of coronavirus infection.
Conclusion: About one-third of patients with epilepsy expressed significant hardship obtaining their drugs after the intensification of the COVID-19 outbreak in Iran. The current COVID-19 pandemic could be considered as a major shock to a nation that has already been under significant pressure (ie, Iran).
C1 [Asadi-Pooya, Ali A.; Farazdaghi, Mohsen; Bazrafshan, Mehdi] Shiraz Univ Med Sci, Epilepsy Res Ctr, Shiraz, Iran.
[Asadi-Pooya, Ali A.] Thomas Jefferson Univ, Dept Neurol, Jefferson Comprehens Epilepsy Ctr, Philadelphia, PA 19107 USA.
C3 Shiraz University of Medical Science; Jefferson University
RP Asadi-Pooya, AA (通讯作者), Shiraz Univ Med Sci, Epilepsy Res Ctr, Shiraz, Iran.
EM aliasadipooya@yahoo.com
TC 14
Z9 15
PD OCT
PY 2020
VL 142
IS 4
BP 392
EP 395
DI 10.1111/ane.13310
EA JUL 2020
WC Clinical Neurology
ER

PT J
AU Asadi-Pooya, AA
Cross, JH
AF Asadi-Pooya, Ali A.
Cross, J. Helen
TI Is wearing a face mask safe for people with epilepsy?
SO ACTA NEUROLOGICA SCANDINAVICA
LA English
DT Review
DE coronavirus; COVID-19; epilepsy; mask; seizure
ID HYPERVENTILATION
AB Since December 2019, the world has been experiencing a catastrophic pandemic of coronavirus disease (COVID-19) caused by SARS-CoV-2. This virus primarily targets the human respiratory system. Available information suggests that people with epilepsy (PWE)

are not at higher risk of being infected by the virus, nor of more severe COVID-19 manifestations, as a result of the epilepsy alone. However, COVID-19 is a serious disease that currently has no effective treatment or vaccine. A face mask is probably effective in preventing the spread of a respiratory pathogen, at least to some extent. So, should we recommend wearing a face mask to all during a pandemic of respiratory infectious disease (eg, COVID-19) without any precautions or exemptions? While concrete evidence is lacking, if we consider that wearing a face mask may simulate hyperventilation, at least to some extent, we would probably avoid recommending this practice indiscriminately to all PWE. On the other hand, in the absence of any proven treatment or vaccine to combat COVID-19, prevention is the best available strategy and it is probably not reasonable to suggest avoid wearing face masks in PWE under any circumstances. Logically, PWE do not need to wear a face mask most of the time, as long as there is no close contact with others, especially during intense physical activities such as exercise. To the contrary, it is probably more advantageous to wear a face mask in crowded locations, with intermittent breaks in safe locations, away from others.
C1 [Asadi-Pooya, Ali A.] Shiraz Univ Med Sci, Epilepsy Res Ctr, Shiraz, Iran.
[Asadi-Pooya, Ali A.] Thomas Jefferson Univ, Dept Neurol, Jefferson Comprehens Epilepsy Ctr, Philadelphia, PA 19107 USA.
[Cross, J. Helen] UCL Great Ormond St Inst Child Hlth, Dev Neurosci, London, England.
C3 Shiraz University of Medical Science; Jefferson University; University of London; University College London
RP Asadi-Pooya, AA (通讯作者), Shiraz Univ Med Sci, Epilepsy Res Ctr, Shiraz, Iran.
EM aliasadipooya@yahoo.com
TC 4
Z9 4
PD OCT
PY 2020
VL 142
IS 4
BP 314
EP 316
DI 10.1111/ane.13316
EA JUL 2020
WC Clinical Neurology
ER

PT J
AU Granata, T
Bisulli, F
Arzimanoglou, A
Rocamora, R
AF Granata, Tiziana
Bisulli, Francesca
Arzimanoglou, Alexis
Rocamora, Rodrigo
TI Did the COVID-19 pandemic silence the needs of people with epilepsy?
SO EPILEPTIC DISORDERS

LA English
DT Article
DE COVID-19; epilepsy; chronic disease; epilepsy care; SARS-CoV-2; people with epilepsy
AB Aims. The COVID-19 pandemic shook European healthcare systems, with unavoidable gaps in the management of patients with chronic diseases. We describe the impact of the pandemic on epilepsy care in three tertiary epilepsy centres from Spain and Italy, the most affected European countries.

Methods. The three epilepsy centres, members of the European EpiCARE network, manage more than 5,700 people with epilepsy. In Bologna and Barcelona, the hospitals housing the epilepsy centres were fully converted into COVID-19 units. We describe the reorganization of the clinics and report on the frequency of SARS-CoV-2 in people with epilepsy as well as the frequency of seizures in patients admitted to the COVID units. Finally, we elaborate on critical issues regarding the second phase of the pandemic.

Results. The activities related to epilepsy care were reduced to less than 10% and were deprioritized. Discharges were expedited and elective epilepsy surgeries, including vagal nerve stimulator implantations, cancelled. Hospitalizations and EEG examinations were limited to emergencies. The outpatient visits for new patients were postponed, and follow-up visits mostly managed by telehealth. Antiseizure medication weaning plans and changes in vagal nerve stimulator settings were halted. Among the 5,700 people with epilepsy managed in our centres, only 14 tested positive for SARS-CoV-2, without obvious impact on their epilepsy. None of the 2,122 patients admitted to COVID units experienced seizures among the early symptoms.

Conclusion. Epilepsy care was negatively impacted by the pandemic, irrespective of COVID-19 epidemiology or conversion of the hospital into a COVID-19 centre. The pandemic did not silence the needs of people with epilepsy, and this must be considered in the planning of the second phase.
C1 [Granata, Tiziana] Fdn IRCCS Ist Neurol Carlo Besta, Dept Pediat Neurosci, Epilepsy Unit, Milan, Italy.

[Granata, Tiziana; Bisulli, Francesca; Arzimanoglou, Alexis; Rocamora, Rodrigo] ERN EpiCARE, Milan, Italy.

[Bisulli, Francesca] IRCCS Ist Sci Neurol Bologna, Bologna, Italy.

[Bisulli, Francesca] Univ Bologna, Dept Biomed & Neuromotor Sci, Bologna, Italy.

[Arzimanoglou, Alexis] Univ Hosp Lyon HCL, Dept Paediat Clin Epileptol Sleep Disorders & Fun, Lyon, France.

[Arzimanoglou, Alexis] Lyon Neurosci Res Ctr, Brain Dynam & Cognit Team DYCOG, INSERM, U1028,CNRS,UMR5292, Lyon, France.

[Arzimanoglou, Alexis] San Juan de Dios Childrens Hosp, Epilepsy Unit, Barcelona, Spain.

[Arzimanoglou, Alexis] Univ Barcelona, Barcelona, Spain.

[Rocamora, Rodrigo] Hosp del Mar, Dept Neurol, Epilepsy Monitoring Unit, Barcelona, Spain.

[Rocamora, Rodrigo] Hosp del Mar, Med Res Inst IMIM, Barcelona, Spain.

C3 IRCCS Istituto Neurologico Besta; IRCCS Istituto delle Scienze Neurologiche di Bologna (ISNB); University of Bologna; CHU Lyon; Centre National de la Recherche Scientifique (CNRS); CNRS - National Institute for Biology (INSB); Institut National de la Sante et de la Recherche Medicale (Inserm); League of European Research Universities - LERU;

University of Barcelona; Institut Hospital del Mar d'Investigacions
Mediques (IMIM); Hospital del Mar; Institut Hospital del Mar
d'Investigacions Mediques (IMIM); Hospital del Mar
RP Bisulli, F (通讯作者), IRCCS Ist Sci Neurol Bologna, Bologna, Italy.; Bisulli, F (通讯作者), Univ Bologna, Dept Biomed & Neuromotor Sci, Bologna, Italy.
EM francesca.bisulli@unibo.it
TC 23
Z9 24
PD AUG
PY 2020
VL 22
IS 4
BP 439
EP 442
DI 10.1684/epd.2020.1175
WC Clinical Neurology
ER

PT J
AU Huang, SS
Wu, CM
Jia, YC
Li, G
Zhu, Z
Lu, K
Yang, YY
Wang, FR
Zhu, SQ
AF Huang, Shanshan
Wu, Chunmei
Jia, Yuchao
Li, Guo
Zhu, Zhou
Lu, Kai
Yang, Yuyan
Wang, Furong
Zhu, Suiqiang
TI COVID-19 outbreak: The impact of stress on seizures in patients with epilepsy
SO EPILEPSIA
LA English
DT Article
DE COVID-19; epilepsy; impact; seizure; stress
ID LONG-TERM OUTCOMES; FREQUENCY; ANXIETY; DEPRESSION; PREVALENCE; EVENTS;
CARE; WAR
AB Objective Stress is a known trigger for seizures in patients with epilepsy (PWE). However, the association between stress and seizures has not been thoroughly

investigated. In December 2019, an outbreak of coronavirus disease (COVID-19) occurred in Wuhan, Hubei province, China, causing tremendous collateral stress. This study was designed to evaluate the influence of the COVID-19 outbreak on seizures in PWE in the most severely affected area, Wuhan, and its surrounding cities. Methods In this single-center, cross-sectional study, PWE were surveyed via online questionnaires between February 23 and March 5, 2020. Collected data included demographic information, epilepsy-related characteristics (seizure type, frequency, antiepileptic drugs [AEDs], and medication management), direct and perceived threat of COVID-19, and changes in seizures during the outbreak. Psychological comorbidities were evaluated by the Patient Health Questionnaire-9, Generalized Anxiety Disorder-7 items, and Insomnia Severity Index (ISI). Multivariate logistic regression was used to identify precipitants for seizure exacerbation. Results We received 362 completed questionnaires after excluding 12 duplicates (response rate = 63.51%). A total of 31 (8.56%) patients had increased seizures during the outbreak. Exposure history to COVID-19 (P = .001), uncontrolled seizure after AED therapy (P = .020), seizure frequency of two or more times per month before the outbreak (P = .005), change of AED regimen during the outbreak (AED reduction, withdrawal, replacement, skipping altogether;P = .002), and worry about the adverse effect of the outbreak on overall seizure-related issues (severity = moderate to critical;P = .038) were risk factors for increased seizures. Significance A minority of PWE experienced seizure exacerbation during the outbreak of COVID-19. Stress, uncontrolled seizures, and inappropriate change in AED regimen were associated with increased seizures. Based on these findings, stress might be an independent precipitant for triggering seizures in some PWE.
C1 [Huang, Shanshan; Wu, Chunmei; Jia, Yuchao; Li, Guo; Zhu, Zhou; Lu, Kai; Yang, Yuyan; Wang, Furong; Zhu, Suiqiang] Huazhong Univ Sci & Technol, Tongji Med Coll, Tongji Hosp, Dept Neurol, 1095 Jiefang Ave, Wuhan 430030, Hubei, Peoples R China.
C3 Huazhong University of Science & Technology
RP Wang, FR; Zhu, SQ (通讯作者), Huazhong Univ Sci & Technol, Tongji Med Coll, Tongji Hosp, Dept Neurol, 1095 Jiefang Ave, Wuhan 430030, Hubei, Peoples R China.
EM wangfurong.china@163.com; zhusuiqiang@163.com
TC 44
Z9 45
PD SEP
PY 2020
VL 61
IS 9
BP 1884
EP 1893
DI 10.1111/epi.16635
EA AUG 2020
WC Clinical Neurology
ER

PT J
AU Power, K
McCrea, Z
White, M

Breen, A
Dunleavy, B
O'Donoghue, S
Jacquemard, T
Lambert, V
El-Naggar, H
Delanty, N
Doherty, C
Fitzsimons, M
AF Power, Kevin
McCrea, Zita
White, Maire
Breen, Annette
Dunleavy, Brendan
O'Donoghue, Sean
Jacquemard, Tim
Lambert, Veronica
El-Naggar, Hany
Delanty, Norman
Doherty, Colin
Fitzsimons, Mary
TI The development of an epilepsy electronic patient portal: Facilitating both patient empowerment and remote clinician-patient interaction in a post-COVID-19 world
SO EPILEPSIA
LA English
DT Article
DE COVID-19; electronic health records; epilepsy; patient portal
ID CENTERED CARE; HEALTH; QUALITY; SAFETY; SYSTEM
AB Objectives The current coronavirus disease 2019 (COVID-19) pandemic stresses an urgency to accelerate much-needed health service reform. Rapid and courageous changes being made to address the immediate impact of the pandemic are demonstrating that the means and technology to enable new models of health care exist. For example, innovations such as electronic patient portals (ePortal) can facilitate (a) radical reform of outpatient care; (b) cost containment in the economically constrained aftermath of the pandemic; (c) environmental sustainability by reduction of unnecessary journeys/transport. Herein, the development of Providing Individualised Services and Care in Epilepsy (PiSCES), an ePortal to the Irish National Epilepsy Electronic Patient Record, is demonstrated. This project, which pre-dates the COVID-19 crisis, aims to facilitate better patient- and family-centered epilepsy care. Methods A combination of ethnographic research, document analysis, and joint application design sessions was used to elicit PiSCES requirements. From these, a specification of desired modules of functionality was established and guided the software development. Results PiSCES functional features include "My Epilepsy Care Summary," "My Epilepsy Care Goals," "My Epilepsy Clinic Letters," "Help Us Measure Your Progress," "Prepare For Your Clinic Visit," "Information for Your Healthcare Provider." The system provides people with epilepsy access to, and engages them as co-authors of, their own medical record. It

can promote improved patient-clinician partnerships and facilitate patient self-management. Significance In the aftermath of COVID-19, it is highly unlikely that the healthcare sector will return to a "business as usual" way of delivering services. The pandemic is expected to accelerate adoption of innovations like PiSCES. It is therefore a catalyst for change that will deliver care that is more responsive to individual patient needs and preferences.
C1 [Power, Kevin; McCrea, Zita; White, Maire; Jacquemard, Tim; El-Naggar, Hany; Delanty, Norman; Doherty, Colin; Fitzsimons, Mary] Royal Coll Surgeons Ireland, Future Neuro SFI Res Ctr, Dublin, Ireland.
[White, Maire; Breen, Annette; El-Naggar, Hany; Delanty, Norman] Beaumont Hosp, Dept Neurol, Dublin, Ireland.
[Dunleavy, Brendan; O'Donoghue, Sean] ERGO IT Solut, Dublin 3, Ireland.
[Lambert, Veronica] Dublin City Univ, Sch Nursing, Psychotherapy & Community Hlth, Dublin, Ireland.
[El-Naggar, Hany; Delanty, Norman; Fitzsimons, Mary] Royal Coll Surgeons Ireland, Sch Pharm & Biomol Sci PBS, Dublin, Ireland.
[Doherty, Colin] St James Hosp, Dept Neurol, Dublin, Ireland.
C3 Royal College of Surgeons - Ireland; Dublin City University; Royal College of Surgeons - Ireland; League of European Research Universities - LERU; Trinity College Dublin
RP Fitzsimons, M (通讯作者), RCSI, FutureNeuro Ctr, Dublin 2, Ireland.
EM marybfitzsimons@rcsi.ie
TC 12
Z9 12
PD SEP
PY 2020
VL 61
IS 9
BP 1894
EP 1905
DI 10.1111/epi.16627
EA AUG 2020
WC Clinical Neurology
ER

PT J
AU Lanzone, J
Cenci, C
Tombini, M
Ricci, L
Tufo, T
Piccioli, M
Marrelli, A
Mecarelli, O
Assenza, G
AF Lanzone, Jacopo
Cenci, Cristina

Tombini, Mario
Ricci, Lorenzo
Tufo, Tommaso
Piccioli, Marta
Marrelli, Alfonso
Mecarelli, Oriano
Assenza, Giovanni
TI Glimpsing the Impact of COVID19 Lock-Down on People With Epilepsy: A Text Mining Approach
SO FRONTIERS IN NEUROLOGY
LA English
DT Article
DE COVID-19; epilepsy; impact; neuropsychology; lockdown
ID DEPRESSION
AB Objectives:To describe how the recent lock-down, related to SARS-COV-II outbreak in Italy, affected People With Epilepsy (PwE), we designed a survey focused on subjective reactions. Using Natural Language Processing (NLP), we analyzed words PwE and People without Epilepsy (PwoE) chose to express their reactions. Methods:As a subset of a larger survey, we collected from both PwE (427) and PwoE (452) single words (one per subject) associated to the period of lock down. The survey was spread thanks to the efforts of Italian league against epilepsy Foundation during the days of maximum raise of the pandemic. Data were analyzed via bag of word and sentiment analysis techniques in R. Results:PwoE and PwE showed significantly different distribution in word choice (X-2,p= 4.904e-13). A subset of subject used positive words to describe this period, subjects with positive feelings about the lock down were more represented in the PwE group (X-2,p= 0.045). Conclusion:PwoE developed reactive stress response to the restrictions enacted during lock-down. PwE, instead, chose words expressing sadness and concern with their disease. PwE appear to internalize more the trauma of lock down. Interestingly PwE also expressed positive feelings about this period of isolation more frequently than PwoE. Our study gives interesting insights on how People with Epilepsy react to traumatic events, using methods that evidence features that do not emerge with psychometric scales.
C1 [Lanzone, Jacopo; Tombini, Mario; Ricci, Lorenzo; Assenza, Giovanni] Univ Campus Biomed Rome, Dept Med, Neurobiol, Unit Neurol,Neurophysiol, Rome, Italy.
[Cenci, Cristina] DNM Digital Narrat Med, Rome, Italy.
[Tufo, Tommaso] Policlin Gemelli Fdn Univ Hosp IRCSS, Rome, Italy.
[Piccioli, Marta] AO S Filippo Neri, Unit Neurol, Rome, Italy.
[Marrelli, Alfonso] AO S Salvatore, Unit Neurol, Laquila, Italy.
[Mecarelli, Oriano] Sapienza Univ Rome, Dept Neurol & Psychiat, Rome, Italy.
C3 University Campus Bio-Medico - Rome Italy; San Filippo Neri Hospital; Sapienza University Rome
RP Lanzone, J (通讯作者), Univ Campus Biomed Rome, Dept Med, Neurobiol, Unit Neurol,Neurophysiol, Rome, Italy.
EM jacopo.lanzone@gmail.com
TC 7
Z9 7
PD AUG 19

PY 2020
VL 11
AR 870
DI 10.3389/fneur.2020.00870
WC Clinical Neurology; Neurosciences
ER

PT J
AU Hogan, RE
Grinspan, Z
Axeen, E
Marquis, B
Day, BK
AF Hogan, Robert Edward
Grinspan, Zachary
Axeen, Erika
Marquis, Belinda
Day, B. Keith
TI COVID-19 in Patients With Seizures and Epilepsy: Interpretation of Relevant Knowledge of Presenting Signs and Symptoms
SO EPILEPSY CURRENTS
LA English
DT Article
DE COVID-19; seizure; epilepsy; signs; symptom
AB There are an increasing number of clinical studies for COVID-19, with several large cohort studies documenting initial signs and symptoms. Realizing the need for current information, this summary provides a focused summary of pertinent clinical diagnostic information about neurological involvement of SARS-CoV-2 virus and clinical presentation of COVID-19, especially in relationship to patients with seizures and epilepsy. There is no evidence from cohort studies in the general population that seizures are worsened in COVID-19. However, relative lack of cohort studies in patients with a history of epileptic seizures limit conclusions about effects of COVID-19 patients with epilepsy. Overall, findings indicate seizures and epilepsy are rare, especially in mild COVID-19 cases, but may occur in more severe cases later in the disease course. Caregivers should be vigilant in assessing for possible seizures, especially in patients with systemic effects of severe COVID-19 infections.
C1 [Hogan, Robert Edward] Washington Univ, Sch Med, Dept Neurol, St Louis, MO 63110 USA.
[Grinspan, Zachary] Weill Cornell Med, Populat Hlth Sci & Pediat, New York, NY USA.
[Axeen, Erika] Univ Virginia Hlth, Charlottesville, VA USA.
[Marquis, Belinda] Weill Cornell Med, Pediat, New York, NY USA.
[Day, B. Keith] Washington Univ, Sch Med, St Louis, MO USA.
C3 Washington University (WUSTL); Cornell University; University of Virginia; Cornell University; Washington University (WUSTL)
RP Hogan, RE (通讯作者), Washington Univ, Sch Med, Adult Epilepsy Ctr, Robert Edward Hogan, St Louis, MO 63130 USA.
EM hoganre@wustl.edu

TC 9
Z9 9
PD SEP
PY 2020
VL 20
IS 5
BP 312
EP 315
AR 1535759720948549
DI 10.1177/1535759720948549
EA AUG 2020
WC Clinical Neurology
ER

PT J
AU Chen, BY
   Kessi, M
   Chen, SM
   Xiong, J
   Wu, LW
   Deng, XL
   Yang, LF
   He, F
   Yin, F
   Peng, J
AF Chen, Baiyu
   Kessi, Miriam
   Chen, Shimeng
   Xiong, Juan
   Wu, Liwen
   Deng, Xiaolu
   Yang, Lifen
   He, Fang
   Yin, Fei
   Peng, Jing
TI The Recommendations for the Management of Chinese Children With Epilepsy
   During the COVID-19 Outbreak
SO FRONTIERS IN PEDIATRICS
LA English
DT Article
DE COVID-19; SARS-CoV2; epilepsy; children; management; recommendation
AB The coronavirus disease (COVID-19) is the most severe public health problem facing the world currently. Social distancing and avoidance of unnecessary movements are preventive strategies that are being advocated to prevent the spread of the causative virus [severe acute respiratory syndrome (SARS)-CoV2]. It is known that epileptic children need long term treatments (antiepileptic drugs and/or immunosuppressive agents) as well as close follow up due to the nature of the disease. In addition, it

is clear that epilepsy can concur with other chronic illnesses which can lower body immunity. As a result, epileptic children have high risk of acquiring this novel disease due to weak/immature immune system. Of concern, the management of children with epilepsy has become more challenging during this outbreak due to the prevention measures that are being taken. Although children with controlled seizures can be managed at home, it is challenging for pediatricians when it comes to cases with uncontrolled seizures/severe cases. To this end, we provide recommendations for the management of epileptic children at home, outpatient and inpatient settings.
C1 [Chen, Baiyu; Kessi, Miriam; Chen, Shimeng; Xiong, Juan; Wu, Liwen; Deng, Xiaolu; Yang, Lifen; He, Fang; Yin, Fei; Peng, Jing] Cent South Univ, Xiangya Hosp, Dept Pediat, Changsha, Peoples R China.
[Chen, Baiyu; Kessi, Miriam; Chen, Shimeng; Xiong, Juan; Wu, Liwen; Deng, Xiaolu; Yang, Lifen; He, Fang; Yin, Fei; Peng, Jing] Hunan Intellectual & Dev Disabil Res Ctr, Changsha, Peoples R China.
C3 Central South University
RP Yin, F; Peng, J (通讯作者), Cent South Univ, Xiangya Hosp, Dept Pediat, Changsha, Peoples R China.; Yin, F; Peng, J (通讯作者), Hunan Intellectual & Dev Disabil Res Ctr, Changsha, Peoples R China.
EM yf2323@hotmail.com; pengjing4346@163.com
TC 2
Z9 2
PD AUG 25
PY 2020
VL 8
AR 495
DI 10.3389/fped.2020.00495
WC Pediatrics
ER

PT J
AU Mirro, EA
Halpern, CH
AF Mirro, Emily A.
Halpern, Casey H.
TI Using Continuous Intracranial Electroencephalography Monitoring to Manage Epilepsy Patients During COVID-19
SO NEUROSURGERY
LA English
DT Letter
DE COVID-19; epilepsy; electroencephalography; management
C1 [Mirro, Emily A.] NeuroPace Inc, Mountain View, CA 94043 USA.
[Halpern, Casey H.] Stanford Univ, Dept Neurosurg, Sch Med, Stanford, CA 94305 USA.
C3 Stanford University
RP Mirro, EA (通讯作者), NeuroPace Inc, Mountain View, CA 94043 USA.
TC 3
Z9 3
PD SEP

PY 2020
VL 87
IS 3
BP E409
EP E410
DI 10.1093/neuros/nyaa244
WC Clinical Neurology; Surgery
ER

PT J
AU Gunal, O
   Ture, E
   Bayburtlu, M
   Arslan, U
   Demirag, MD
   Taskin, MH
   Kilic, S
AF Gunal, Ozgur
   Ture, Eda
   Bayburtlu, Meryem
   Arslan, Ugur
   Demirag, Mehmet Derya
   Taskin, Mehmet Hakan
   Kilic, Sirri
TI Evaluation of Patients Diagnosed with COVID-19 in Terms of Risk Factors
SO MIKROBIYOLOJI BULTENI
LA Turkish
DT Article
DE Coronaviruse; COM-19; risk factor; comorbidity
ID WUHAN
AB Coronaviruses are RNA viruses that can cause disease in the upper and lower respiratory tract in humans and animals. Lately, a new coronavirus causing pneumonia cases was detected in Wuhan, China in December 2019. Soon after, the name of the virus was identified as the "severe acute respiratory syndrome coronavirus-2", and the World Health Organization named the disease coronavirus disease-2019 (COVID-19). In our country, the first cases began to appear in the second week of March. In this study, we aimed to investigate the demographic characteristics and risk factors of patients with the diagnosis of COVID-19. A total of 100 patients (53 female and 47 male) were included in our study. The patients included in the study were randomly selected from the registration system and their information was evaluated retrospectively. The mean age of the patients was 54.42 (Age range= 20-90). When the risk factors for catching the disease were evaluated; it was determined that there was at least one risk factor in 46 patients; 30 patients had close contact with the COVID-19 patient in the social environment (30%) and 16 patients had a travel history outside the city in the last 14 days (16%). The most common symptoms in our patients were; cough (93%), fever (42%), dyspnea (22%), weakness (8%), sore throat (7%), diarrhea (6%), headache (5%) and sputum (2%). The most common comorbid conditions in our patients were detected as hypertension

(42%), diabetes mellitus (DM) (21%), congestive heart failure (10%), allergic asthma (7%), chronic obstructive pulmonary disease (6%), rheumatoid arthritis (3%), coronary artery disease (2%), solid organ tumour (2%), depression (1%) and epilepsy (1%). The mean age of our 15 patients who were monitored in intensive care unit was 65 y (+/- 11.46), the mean age of 85 patients followed in the service was 52.55 (+/- 16.35) and this difference was statistically significant (p= 0.006). When these two groups were compared in terms of comorbid diseases, the presence of DM was 40% higher (n= 6) in intensive care patients, and this difference was statistically significant (p= 0.05). In addition, the majority [11 patients (73%)] of the patients hospitalized in the intensive care unit were male (p= 0.03). When smoking was evaluated as a risk factor for serious illness, 4 of 11 patients (26%) in intensive care unit had a smoking history, while none of the patients who have died due to COVID-19 had a smoking history. These findings suggested to us that smoking does not increase the severity of COVID-19 disease. As a result, knowledge about the disease should be increased rapidly by sharing the studies on risk factors, transmission routes and clinical features of COVID-19 infection, which affects the whole world.
C1 [Gunal, Ozgur; Kilic, Sirri] Hlth Sci Univ, Samsun Training & Res Hosp, Clin Infect Dis & Clin Microbiol, Samsun, Turkey.
[Ture, Eda; Bayburtlu, Meryem] Hlth Sci Univ, Samsun Training & Res Hosp, Clin Family Med, Samsun, Turkey.
[Arslan, Ugur] Hlth Sci Univ, Samsun Training & Res Hosp, Clin Cardiol, Samsun, Turkey.
[Demirag, Mehmet Derya] Hlth Sci Univ, Samsun Training & Res Hosp, Clin Rheumatol, Clin Internal Med, Samsun, Turkey.
[Taskin, Mehmet Hakan] Hlth Sci Univ, Samsun Training & Res Hosp, Clin Clin Microbiol, Samsun, Turkey.
C3 Samsun Training & Research Hospital; University of Health Sciences Turkey; Samsun Training & Research Hospital; University of Health Sciences Turkey; Samsun Training & Research Hospital; University of Health Sciences Turkey; Samsun Training & Research Hospital; University of Health Sciences Turkey; Samsun Training & Research Hospital; University of Health Sciences Turkey
RP Gunal, O (通讯作者), Hlth Sci Univ, Samsun Training & Res Hosp, Clin Infect Dis & Clin Microbiol, Samsun, Turkey.
EM ozgurgop@yahoo.com
TC 8
Z9 8
PD SEP
PY 2020
VL 54
IS 4
BP 575
EP 582
DI 10.5578/mb.69811
WC Microbiology
ER

PT J
AU Brigo, F
   Bonavita, S
   Leocani, L
   Tedeschi, G
   Lavorgna, L
AF Brigo, Francesco
   Bonavita, Simona
   Leocani, Letizia
   Tedeschi, Gioacchino
   Lavorgna, Luigi
TI Telemedicine and the challenge of epilepsy management at the time of
   COVID-19 pandemic
SO EPILEPSY & BEHAVIOR
LA English
DT Letter
DE COVID-19; Epilepsy; Mobile devices; Seizure; management; Telemedicine
C1 [Brigo, Francesco] Franz Tappeiner Hosp, Dept Neurol, Merano, Italy.
   [Bonavita, Simona; Tedeschi, Gioacchino; Lavorgna, Luigi] Univ Campania Luigi Vanvitelli, Dept Adv Med & Surg Sci, Clin Neurol 1, Naples, Italy.
   [Leocani, Letizia] IRCCS San Raffaele Hosp, Dept Neurorehabil, Milan, Italy.
   [Leocani, Letizia] Univ Vita Salute San Raffaele, Milan, Italy.
C3 Ospedale Franz Tappeiner; Universita della Campania Vanvitelli; IRCCS
   Ospedale San Raffaele; Vita-Salute San Raffaele University; Vita-Salute
   San Raffaele University
RP Lavorgna, L (通讯作者), Italian Soc Neurol, Digital Technol, Web & Social Media Study Grp, Piazza Miraglia2, I-80138 Naples, Italy.
EM luigi.lavorgna@policliniconapoli.it
TC 27
Z9 27
PD SEP
PY 2020
VL 110
AR 107164
DI 10.1016/j.yebeh.2020.107164
WC Behavioral Sciences; Clinical Neurology; Psychiatry
ER

PT J
AU Vancini, RL
   de Lira, CAB
   Andrade, MS
   Arida, RM
AF Vancini, Rodrigo Luiz
   Lira, Claudio Andre Barbosa de
   Andrade, Marilia Santos
   Arida, Ricardo Mario

TI CoVID-19 vs. epilepsy: It is time to move, act, and encourage physical
   exercise
SO EPILEPSY & BEHAVIOR
LA English
DT Letter
DE COVID-19; epilepsy; physical exercise
ID COPING STRATEGY; ANTIEPILEPTIC DRUGS; PEOPLE; DEPRESSION; MEDICINE
C1 [Vancini, Rodrigo Luiz] Univ Fed Espirito Santo, Ctr Educ Fis & Desportos, Campus Univ,Ave Fernando Ferrari 514, BR-29075810 Vitoria, ES, Brazil.
   [Lira, Claudio Andre Barbosa de] Univ Fed Goias, Setor Fisiol Humana & Exercicio, Lab Avaliacao Movimento, Humano,Fac Educ Fis & Danca, Goiania, Go, Brazil.
   [Andrade, Marilia Santos; Arida, Ricardo Mario] Univ Fed Sao Paulo, Dept Fisiol, Sao Paulo, Brazil.
C3 Universidade Federal do Espirito Santo; Universidade Federal de Goias;
   Universidade Federal de Sao Paulo (UNIFESP)
RP Vancini, RL (通讯作者), Univ Fed Espirito Santo, Ctr Educ Fis & Desportos, Campus Univ,Ave Fernando Ferrari 514, BR-29075810 Vitoria, ES, Brazil.
EM rodrigoluizvancini@gmail.com
TC 7
Z9 7
PD SEP
PY 2020
VL 110
AR 107154
DI 10.1016/j.yebeh.2020.107154
WC Behavioral Sciences; Clinical Neurology; Psychiatry
ER

PT J
AU Pati, S
   Houston, T
AF Pati, Sandipan
   Houston, Thomas
TI Assessing the risk of seizures with chloroquine or hydroxychloroquine
   therapy for COVID-19 in persons with epilepsy
SO EPILEPSY RESEARCH
LA English
DT Article
DE Chloroquine; Hydroxychloroquine; COVID-19; Epilepsy; risk; seizure
ID INVOLVEMENT
AB Background: The goal of this systematic review is to assess the published literature for seizure risk with chloroquine or hydroxychloroquine therapy in persons with and without epilepsy. With the COVID-19 pandemic, there is a desperate need for treatment against the SARS CoV-2 virus. Chloroquine or hydroxychloroquine is one proposed medication that has received substantial public attention. However, the package insert states that these medications may provoke seizures in patients with epilepsy, and this has resulted in increased questions and anxiety in the epilepsy community.

Methods: PubMed (1970 to March 27, 2020) and the Embase (1970 to March 27, 2020) were searched with the terms chloroquine or hydroxychloroquine and seizure or epilepsy, convulsions, or status epilepticus. Selected studies were reviewed, and the adverse drug reaction was classified.

Results: Only eleven out of 31 studies were deemed eligible for systematic analysis. For chloroquine, eligible studies were- one prospective study(n = 109), two case series(n = 6), and six case reports. The dose of chloroquine ranged between 100-500 mg/day, except in one patient with a seizure, who was after taking 1000 mg. For hydroxychloroquine, there was one prospective observational study(n = 631) and one case report. The clinical trials failed to find any significant relation between seizures and chloroquine or hydroxychloroquine.

Conclusion: Although the package insert describes an increased risk of seizure, the systematic review highlights that such a statement is not supported by class I evidence. Clinicians, therefore, need to understand that data regarding this specific topic is limited to case series and case reports. There is no substantial evidence to suggest that these medications can increase seizure risk.
C1 [Pati, Sandipan; Houston, Thomas] Univ Alabama Birmingham, Dept Neurol, Birmingham, AL 35294 USA.

[Pati, Sandipan; Houston, Thomas] Univ Alabama Birmingham, Epilepsy & Cognit Neurophysiol Lab, Birmingham, AL 35294 USA.

C3 University of Alabama System; University of Alabama Birmingham;

University of Alabama System; University of Alabama Birmingham

RP Pati, S (通讯作者), Univ Alabama Birmingham, Dept Neurol, Epilepsy & Cognit Neurophysiol Lab, CIRC 312 1719,6th Ave South, Birmingham, AL 35294 USA.

EM spati@uabmc.edu
TC 2

Z9 3

PD SEP

PY 2020

VL 165

AR 106399

DI 10.1016/j.eplepsyres.2020.106399

WC Clinical Neurology

ER

PT J

AU Fonseca, E

Quintana, M

Lallana, S

Restrepo, JL

Abraira, L

Santamarina, E

Seijo-Raposo, I

Toledo, M

AF Fonseca, Elena

Quintana, Manuel

Lallana, Sofia

Luis Restrepo, Juan
Abraira, Laura
Santamarina, Estevo
Seijo-Raposo, Ivan
Toledo, Manuel
TI Epilepsy in time of COVID-19: A survey-based study
SO ACTA NEUROLOGICA SCANDINAVICA
LA English
DT Article
DE epilepsy; quality of life; seizure; management; COVID-19; risk
ID PEOPLE
AB Objectives Collateral damage may occur in epilepsy management during the coronavirus (COVID-19) pandemic. We aimed to establish the impact of this pandemic on epilepsy patients in terms of patient-reported seizure control and emerging symptoms. Materials & Methods This is a cross-sectional study including consecutive patients assessed by telephone contact in an epilepsy clinic during the first month of confinement. Demographic and clinical characteristics were recorded, and a 19-item questionnaire was systematically completed. Data regarding the impact of confinement, economic effects of the pandemic, and subjective perception of telemedicine were recorded. Additional clinical data were obtained in patients with a COVID-19 diagnosis. Results Two hundred and fifty-five patients were recruited: mean age 48.2 +/- 19.8 years, 121 (47.5%) women. An increase in seizure frequency was reported by 25 (9.8%) patients. Sixty-eight (26.7%) patients reported confinement-related anxiety, 22 (8.6%) depression, 31 (12.2%) both, and 72 (28.2%) insomnia. Seventy-three (28.6%) patients reported a reduction in economic income. Logistic regression analysis showed that tumor-related epilepsy etiology [OR = 7.36 (95% CI 2.17-24.96)], drug-resistant epilepsy [OR = 3.44 (95% CI 1.19-9.95)], insomnia [OR = 3.25 (95% CI 1.18-8.96)], fear of epilepsy [OR = 3.26 (95% CI 1.09-9.74)], and income reduction [OR = 3.65 (95% CI 1.21-10.95)] were associated with a higher risk of increased seizure frequency. Telemedicine was considered satisfactory by 214 (83.9%) patients. Five patients were diagnosed with COVID-19, with no changes in seizure frequency. Conclusions The COVID-19 pandemic has effects in epilepsy patients. Patients with tumor-related, drug-resistant epilepsy, insomnia, and economic difficulties are at a higher risk of increased seizure frequency. Telemedicine represents a suitable tool in this setting.
C1 [Fonseca, Elena; Quintana, Manuel; Lallana, Sofia; Luis Restrepo, Juan; Abraira, Laura; Santamarina, Estevo; Seijo-Raposo, Ivan; Toledo, Manuel] Vall dHebron Univ Hosp, Epilepsy Unit, Neurol Dept, Barcelona, Spain.
[Fonseca, Elena; Quintana, Manuel; Lallana, Sofia; Luis Restrepo, Juan; Abraira, Laura; Santamarina, Estevo; Seijo-Raposo, Ivan; Toledo, Manuel] Univ Autonoma Barcelona, Med Dept, Barcelona, Spain.
[Fonseca, Elena; Quintana, Manuel; Abraira, Laura; Santamarina, Estevo; Seijo-Raposo, Ivan; Toledo, Manuel] Vall dHebron Res Inst VHIR, Epilepsy Res Grp, Barcelona, Spain.
C3 Hospital Universitari Vall d'Hebron; Autonomous University of Barcelona;
Autonomous University of Barcelona; Hospital Universitari Vall d'Hebron;
Vall d'Hebron Institut de Recerca (VHIR)
RP Santamarina, E (通讯作者), Vall dHebron Univ Hosp, Epilepsy Unit, Passeig Vall

dHebron 119-129, Barcelona, Spain.
EM esantama@vhebron.net
TC 39
Z9 40
PD DEC
PY 2020
VL 142
IS 6
BP 545
EP 554
DI 10.1111/ane.13335
EA SEP 2020
WC Clinical Neurology
ER

PT J
AU Cabezudo-Garcia, P
Ciano-Petersen, NL
Mena-Vazquez, N
Pons-Pons, G
Castro-Sanchez, MV
Serrano-Castro, PJ
AF Cabezudo-Garcia, Pablo
Lundahl Ciano-Petersen, Nicolas
Mena-Vazquez, Natalia
Pons-Pons, Gracia
Victoria Castro-Sanchez, Maria
Serrano-Castro, Pedro J.
TI Incidence and case fatality rate of COVID-19 in patients with active epilepsy
SO NEUROLOGY
LA English
DT Article
DE incidence; fatality; COVID-19; epilepsy
ID COMORBIDITY
AB Objective This article estimates the incidence and fatality of coronavirus disease 2019 (COVID-19) and identifies potential risk factors for fatality in patients with active epilepsy. Methods This is a cross-sectional observational study of patients with active epilepsy and COVID-19. A control group was used to compare the cumulative incidence and case-fatality rate (CFR). The main outcomes of the study were cumulative incidence, defined as number of patients with active epilepsy and COVID-19 admitted to an emergency department divided by the total number of patients with epilepsy at risk, and CFR based on the number of deaths during the enrollment period. Multiple logistic regression analysis was performed to investigate risk factors for fatality in patients with active epilepsy. Results Of the 1,537 patients who fulfilled the inclusion criteria, 21 (1.3%) had active epilepsy. The cumulative incidence (95% confidence interval [CI]) of COVID-19 in patients with epilepsy was higher (1.2%

[0.6-2.4]) compared to the population without epilepsy (0.5% [0.5-0.5]). In reverse transcription PCR-positive patients, there were no significant differences in CFR in patients with active epilepsy compared to patients without epilepsy (33.3% vs 8.3%;p=0.266). Of the 21 patients with active epilepsy, 5 (23%) died. In multivariate analysis, the factor associated with fatality in patients with active epilepsy was hypertension (odds ratio [OR] 2.8 [95% CI 1.3-21.6]). In another model, age (OR 1.0 [95% CI 1.0-1.1]) and epilepsy (OR 5.1 [95% CI 1.3-24.0]) were associated with fatality during hospitalization. Conclusion COVID-19 cumulative incidence was higher in patients with active epilepsy. Epilepsy was associated with fatality during hospitalization. Hypertension was associated with fatality in patients with epilepsy.
C1 [Cabezudo-Garcia, Pablo; Lundahl Ciano-Petersen, Nicolas; Mena-Vazquez, Natalia; Pons-Pons, Gracia; Victoria Castro-Sanchez, Maria; Serrano-Castro, Pedro J.] Hosp Reg Univ Malaga, Inst Invest Biomed Malaga IBIMA, Malaga, Spain.
[Cabezudo-Garcia, Pablo; Lundahl Ciano-Petersen, Nicolas; Pons-Pons, Gracia; Victoria Castro-Sanchez, Maria; Serrano-Castro, Pedro J.] Hosp Reg Univ Malaga, Serv Neurol, Unidad Gest Clin Neurociencias, Malaga, Spain.
[Mena-Vazquez, Natalia] Hosp Reg Univ Malaga, Gest Clin Reumatol, Malaga, Spain.
C3 Universidad de Malaga
RP Cabezudo-Garcia, P (通讯作者), Hosp Reg Univ Malaga, Inst Invest Biomed Malaga IBIMA, Malaga, Spain.; Cabezudo-Garcia, P (通讯作者), Hosp Reg Univ Malaga, Serv Neurol, Unidad Gest Clin Neurociencias, Malaga, Spain.
EM pablocabezudo@gmail.com
TC 41
Z9 41
PD SEP 8
PY 2020
VL 95
IS 10
BP E1417
EP E1425
DI 10.1212/WNL.0000000000010033
WC Clinical Neurology
ER

PT J
AU Albert, DVF
Das, RR
Acharya, JN
Lee, JW
Pollard, JR
Punia, V
Keller, JA
Husain, AM
AF Albert, Dara V. F.
Das, Rohit R.
Acharya, Jayant N.
Lee, Jong Woo

Pollard, John R.
Punia, Vineet
Keller, Joy A.
Husain, Aatif M.
TI The Impact of COVID-19 on Epilepsy Care: A Survey of the American Epilepsy Society Membership
SO EPILEPSY CURRENTS
LA English
DT Article
DE COVID-19; SARS-CoV-2; survey; epilepsy; epilepsy care; people with epilepsy; EEG; telemedicine; seizure
ID STROKE CARE; TELEMEDICINE; TELEHEALTH
AB The COVID-19 pandemic has impacted the delivery of care to people with epilepsy (PWE) in multiple ways including limitations on in-person contact and restrictions on neurophysiological procedures. To better study the effect of the pandemic on PWE, members of the American Epilepsy Society were surveyed between April 30 and June 14, 2020. There were 366 initial responses (9% response rate) and 337 respondents remained for analysis after screening out noncompleters and those not directly involved with clinical care; the majority were physicians from the United States. About a third (30%) of respondents stated that they had patients with COVID-19 and reported no significant change in seizure frequency. Conversely, one-third of respondents reported new onset seizures in patients with COVID-19 who had no prior history of seizures. The majority of respondents felt that there were at least some barriers for PWE in receiving appropriate clinical care, neurophysiologic procedures, and elective surgery. Medication shortages were noted by approximately 30% of respondents, with no clear pattern in types of medication involved. Telehealth was overwhelmingly found to have value. Among the limitation of the survey was that it was administered at a single point in time in a rapidly changing pandemic. The survey showed that almost all respondents were affected by the pandemic in a variety of ways.
C1 [Albert, Dara V. F.] Ohio State Univ, Dept Pediat, Div Child Neurol, Nationwide Childrens Hosp, Columbus, OH 43210 USA.
[Das, Rohit R.] Univ Texas Southwestern Med Ctr Dallas, Dept Neurol, Dallas, TX USA.
[Acharya, Jayant N.] Penn State Hlth, Dept Neurol, Hershey, PA USA.
[Lee, Jong Woo] Brigham & Womens Hosp, Dept Neurol, 75 Francis St, Boston, MA 02115 USA.
[Pollard, John R.] Cristiana Care, Dept Neurol, Newark, DE USA.
[Pollard, John R.] Univ Penn, Dept Neurol, Philadelphia, PA 19104 USA.
[Punia, Vineet] Cleveland Clin Fdn, Neurol Inst, 9500 Euclid Ave, Cleveland, OH 44195 USA.
[Keller, Joy A.] Amer Epilepsy Soc, Chicago, IL USA.
[Husain, Aatif M.] Duke Univ, Med Ctr, 299B Hanes House,315 Trent Dr, Durham, NC 27710 USA.
[Husain, Aatif M.] Vet Affairs Med Ctr, 299B Hanes House,315 Trent Dr, Durham, NC 27710 USA.
C3 Ohio State University; University of Texas System; University of Texas Southwestern Medical Center Dallas; Pennsylvania Commonwealth System of Higher Education (PCSHE); Pennsylvania State University; Penn State

Health; Harvard University; Brigham & Women's Hospital; University of Pennsylvania; Cleveland Clinic Foundation; Duke University; US Department of Veterans Affairs; Veterans Health Administration (VHA)
RP Husain, AM (通讯作者), Duke Univ, Med Ctr, 299B Hanes House,315 Trent Dr, Durham, NC 27710 USA.; Husain, AM (通讯作者), Vet Affairs Med Ctr, 299B Hanes House,315 Trent Dr, Durham, NC 27710 USA.
EM aatif.husain@duke.edu
TC 20
Z9 20
PD SEP
PY 2020
VL 20
IS 5
BP 316
EP 324
AR 1535759720956994
DI 10.1177/1535759720956994
EA SEP 2020
WC Clinical Neurology
ER

PT J
AU Abdulsalam, MA
Abdulsalam, AJ
Shehab, D
AF Abdulsalam, Mohammad A.
Abdulsalam, Ahmad J.
Shehab, Diaa
TI Generalized status epilepticus as a possible manifestation of COVID-19
SO ACTA NEUROLOGICA SCANDINAVICA
LA English
DT Editorial Material
DE COVID-19; epilepsy; seizure; status epilepticus
C1 [Abdulsalam, Mohammad A.] Mubarak Al Kabeer Hosp, Dept Internal Med, Jabriya, Kuwait.
[Abdulsalam, Ahmad J.] Phys Med & Rehabil Hosp, Dept Phys Med & Rehabil, Andalous, Kuwait.
[Shehab, Diaa] Kuwait Univ, Hlth Sci Ctr, Fac Med, Jabriya, Kuwait.
C3 Mubarak Al-Kabeer Hospital; Kuwait University
RP Abdulsalam, AJ (通讯作者), Phys Med & Rehabil Hosp, Dept Phys Med & Rehabil, Andalous, Kuwait.
EM dr.ahmad.j.abdulsalam@gmail.com
TC 12
Z9 12
PD OCT
PY 2020
VL 142
IS 4

BP 297
EP 298
DI 10.1111/ane.13321
WC Clinical Neurology
ER

PT J
AU Panda, PK
   Dawman, L
   Panda, P
   Sharawat, IK
AF Panda, Prateek Kumar
   Dawman, Lesa
   Panda, Pragnya
   Sharawat, Indar Kumar
TI Feasibility and effectiveness of teleconsultation in children with
   epilepsy amidst the ongoing COVID-19 pandemic in a resource-limited
   country
SO SEIZURE-EUROPEAN JOURNAL OF EPILEPSY
LA English
DT Article
DE Teleconsultation; Epilepsy; COVID-19; Antiepileptic drug; neurology
ID FOLLOW-UP; TELEMEDICINE
AB Introduction: The ongoing COVID-19 pandemic and the lockdown measures employed by the government have forced neurologists across the world to look upon telemedicine as the only feasible and practical option to continue providing health care towards children with epilepsy in home isolation. Children with epilepsy are challenging for teleconsultation as direct information from the patient is missing, regarding seizures and adverse effects, especially behavioral and psychological side effects.

   Methods: Clinical and epilepsy-related details of telephonic consultations for children 1 month-18 years, performed between 26th March and 17th May 2020 in a tertiary care teaching hospital in Uttarakhand (a state of India known for hilly terrains with low per capita income) were recorded. Suitable changes in the dose/commercial brand of antiepileptic drug (AED) regimen were performed, along with the addition of new AED and referral to local practitioners for immediate hospitalization, when urgent health care issues were detected. Voice call, text message, picture/video message, and all other possible measures were employed to accumulate maximum clinical information in real-time.

   Results: A total of 153 children(95 males [62 %], 9.45 +/- 3.24 years, 140 lower/middle socioeconomic status) were enrolled after screening 237 children with various neurological disorders, whose caregivers contacted for teleconsultation. A total of 278 telephone consultations performed for these 153 children (1-5 telephone calls per patient). Hundred-thirteen children were identified to have a total of 152 significant clinical events (breakthrough seizure/uncontrolled epilepsy (108), AED related (13), and unrelated systemic adverse effects (24), worsening of associated co-morbidities (7). In rest of the patients, the query of the caregiver included unavailability of AED/prescribed commercial brand in the locality, query related to

the dose of drugs, proxy for a scheduled routine visit (no active issues), and concern regarding COVID-19 related symptoms and effect of COVID-19 and lockdown in children with epilepsy. Ninety-three (60 %) patients required hiking up of AED dose, whereas 29 (17 %) patients required the addition of a new AED/commercial brand. Five children were advised immediate admission to a nearby hospital. Overall, 147 (96 %) caregivers were satisfied with the quality of medical advice.

Conclusion: Teleconsultation is one of the few feasible options with good effectiveness for providing medical advice to children with epilepsy during pandemic times.
C1 [Panda, Prateek Kumar; Sharawat, Indar Kumar] All India Inst Med Sci, Dept Pediat, Pediat Neurol Div, Rishikesh 249203, Uttarakhand, India.
[Dawman, Lesa] Post Grad Inst Med Educ & Res, Dept Pediat, Chandigarh 160012, India.
[Panda, Pragnya] SCB Med Coll, Dept Med, Cuttack, Odisha, India.
C3 All India Institute of Medical Sciences (AIIMS) Rishikesh; Post Graduate Institute of Medical Education & Research (PGIMER), Chandigarh; Srirama Chandra Bhanja Medical College & Hospital
RP Sharawat, IK (通讯作者), All India Inst Med Sci, Dept Pediat, Pediat Neurol Div, Rishikesh 249203, Uttarakhand, India.
EM sherawatdrindar@gmail.com
TC 25
Z9 25
PD OCT
PY 2020
VL 81
BP 29
EP 35
DI 10.1016/j.seizure.2020.07.013
WC Clinical Neurology; Neurosciences
ER

PT J
AU Krysl, D
Beniczky, S
Franceschetti, S
Arzimanoglou, A
AF Krysl, David
Beniczky, Sandor
Franceschetti, Silvana
Arzimanoglou, Alexis
TI The COVID-19 outbreak and approaches to performing EEG in Europe
SO EPILEPTIC DISORDERS
LA English
DT Article
DE coronavirus; electroencephalography; epilepsy; surgery; video-EEG
ID EPILEPSY
AB Aims. The coronavirus SARS-CoV-2 disease (COVID-19) pandemic affects availability and performance of neurophysiological diagnostic methods, including EEG. Our objective

was to outline the current situation regarding EEG-based investigations across Europe.
Methods. A web-based survey was distributed to centres within the European Reference Network on rare and complex epilepsies (ERN EpiCARE). Responses were collected between April 9 and May 15, 2020. Results were analysed with Microsoft Excel, Python Pandas and SciPy.
Results. Representants from 47 EpiCARE centres from 22 countries completed the survey. At the time of completing the survey, inpatient video-EEGs had been stopped or restricted in most centres (61.7% vs. 36.2% for adults, and 38.3% vs. 53.2% for children). Invasive investigations and epilepsy surgery were similarly affected. Acute EEGs continued to be performed, while indications for outpatient EEGs were limited and COVID-19 triage put in place. The strictness of measures varied according to extent of the outbreak in a given country.
Conclusions. The results indicate a profound impact of COVID-19 on neurophysiological diagnostics, especially inpatient video-EEGs, invasive investigations, and epilepsy surgery. The COVID-19 pandemic may hamper care for patients in need of EEG-based investigations, particularly patients with seizure disorders. ERN EpiCARE will work on recommendations on how to rapidly adapt to such situations in order to alleviate consequences for our patients.
C1 [Krysl, David] Sahlgrens Univ Hosp, Dept Clin Neurophysiol, Bla Straket 7, S-41345 Gothenburg, Sweden.
[Krysl, David] ERN EpiCARE, Gothenburg, Sweden.
[Krysl, David] Univ Gothenburg, Sahlgrenska Acad, Inst Neurosci & Physiol, Gothenburg, Sweden.
[Beniczky, Sandor] Danish Epilepsy Ctr, Dept Clin Neurophysiol, Dianalund, Denmark.
[Beniczky, Sandor] ERN EpiCARE, Dianalund, Denmark.
[Beniczky, Sandor] Aarhus Univ Hosp, Aarhus, Denmark.
[Beniczky, Sandor] Aarhus Univ, Dept Clin Med, Aarhus, Denmark.
[Franceschetti, Silvana] Fdn IRCCS Ist Neurol Carlo Besta, Neurophysiol Unit, Milan, Italy.
[Franceschetti, Silvana] ERN EpiCARE, Milan, Italy.
[Arzimanoglou, Alexis] Univ Hosp Lyon HCL, Dept Paediat Clin Epileptol Sleep Disorders & Fun, Lyon, France.
[Arzimanoglou, Alexis] ERN EpiCARE Lyon, Lyon, France.
[Arzimanoglou, Alexis] Univ Barcelona, San Juan de Dios Childrens Hosp, Epilepsy Unit, Barcelona, Spain.
[Arzimanoglou, Alexis] ERN EpiCARE, Barcelona, Spain.
C3 Sahlgrenska University Hospital; University of Gothenburg; Aarhus University; Aarhus University; IRCCS Istituto Neurologico Besta; CHU Lyon; League of European Research Universities - LERU; University of Barcelona
RP Krysl, D (通讯作者), Sahlgrens Univ Hosp, Dept Clin Neurophysiol, Bla Straket 7, S-41345 Gothenburg, Sweden.
EM david.krysl@vgregion.se
TC 8
Z9 8
PD OCT
PY 2020

VL 22
IS 5
BP 548
EP 554
DI 10.1684/epd.2020.1208
WC Clinical Neurology
ER

PT J
AU Conde-Blanco, E
   Centeno, M
   Tio, E
   Muriana, D
   Garcia-Penas, JJ
   Serrano, P
   Nagel, AG
   Serratosa, J
   Jimenez, AP
   Toledo, M
   Donaire, A
   Manzanares, I
   Betran, O
   Carreno, M
AF Conde-Blanco, Estefania
   Centeno, Maria
   Tio, Ester
   Muriana, Desiree
   Garcia-Penas, Juan Jose
   Serrano, Pedro
   Nagel, Antonio Gil
   Serratosa, Jose
   Jimenez, Angeles Perez
   Toledo, Manuel
   Donaire, Antonio
   Manzanares, Isabel
   Betran, Olga
   Carreno, Mar
TI Emergency implementation of telemedicine for epilepsy in Spain: Results
   of a survey during SARS-CoV-2 pandemic
SO EPILEPSY & BEHAVIOR
LA English
DT Article
DE Telemedicine; Teleneurology; Epilepsy; Adult; Pediatric
ID NEUROLOGY; FUTURE
AB Teleneurology in Spain had not been implemented so far in clinical practice, except in urgent patients with stroke. Telemedicine was hardly used in epilepsy, and patients and neurologists usually preferred onsite visits. Our goal was to study impressions

of adult and pediatric epileptologists about the use of telemedicine after emergent implementation during the new coronavirus 2019 (COVID-19) pandemic.

Methods: An online survey was sent to the members of the Spanish Epilepsy Society and the members of the Epilepsy Study Group of the Catalan Neurological Society, inquiring about different aspects of telemedicine in epilepsy during the pandemic lockdown.

Results: A total of 66 neurologists responded, mostly adult neurologists (80.3%), the majority with a monographic epilepsy clinic (4 out of 5). Of all respondents, 59.1% reported to attend more than 20 patients with epilepsy (PWE) a week. During the pandemic, respondents handled their epilepsy clinics mainly with telephone calls (88%); only 4.5% used videoconference. Changes in antiseizure medications were performed less frequently than during onsite visits by 66.6% of the epileptologists. Scales were not administered during these visits, and certain types of information such as sudden expected unrelated death in epilepsy (SUDEP) were felt to be more appropriate to discuss in person. More than 4 out of 5 of the neurologists (84.8%) stated that they would be open to perform some telematic visits in the future.

Conclusions: In Spain, emergent implantation of teleneurology has shown to be appropriate for the care of many PWE. Technical improvements, extended use of videoconference and patient selection may improve results and patient and physician satisfaction. (C) 2020 Published by Elsevier Inc.
C1 [Conde-Blanco, Estefania; Centeno, Maria; Donaire, Antonio; Manzanares, Isabel; Betran, Olga; Carreno, Mar] Hosp Clin Barcelona, Inst Invest Biomed August Pi & Sunyer IDIBAPS, Epilepsy Study Grp, Catalan Neurol Soc,Spanish Epilepsy Soc,European, Barcelona, Spain.

[Tio, Ester] Hosp Univ Parc Tauli, Epilepsy Study Grp, Catalan Neurol Soc, Barcelona, Spain.

[Muriana, Desiree] Hosp Mataro, Epilepsy Study Grp, Catalan Neurol Soc, Barcelona, Spain.

[Garcia-Penas, Juan Jose; Jimenez, Angeles Perez] Hosp Infantil Univ Nino Jesus, Spanish Epilepsy Soc, Madrid, Spain.

[Serrano, Pedro] Hosp Reg Univ Malaga, Spanish Epilepsy Soc, Malaga, Spain.

[Nagel, Antonio Gil] Hosp Ruber Int, Spanish Epilepsy Soc, Madrid, Spain.

[Serratosa, Jose] Hosp Univ Fdn Jimenez Diaz, Spanish Epilepsy Soc, Madrid, Spain.

[Toledo, Manuel] Hosp Univ Vall dHebron, Spanish Epilepsy Soc, Barcelona, Spain.

C3 League of European Research Universities - LERU; University of Barcelona; Hospital Clinic de Barcelona; IDIBAPS; Autonomous University of Barcelona; Parc Tauli Hospital Universitari; Hospital Universitari Vall d'Hebron

RP Carreno, M (通讯作者), Hosp Clin Barcelona, Dept Neurol, Epilepsy Unit, Carrer Villarroel 170, Barcelona 08036, Spain.

EM mcarreno@clinic.cat
TC 21

Z9 22

PD OCT

PY 2020

VL 111

AR 107211

DI 10.1016/j.yebeh.2020.107211
WC Behavioral Sciences; Clinical Neurology; Psychiatry
ER

PT J
AU Kossoff, EH
Turner, Z
Adams, J
Bessone, SK
Avallone, J
McDonald, TJW
Diaz-Arias, L
Barron, BJ
Vizthum, D
Cervenka, MC
AF Kossoff, Eric H.
Turner, Zahava
Adams, Jamie
Bessone, Stacey K.
Avallone, Jennifer
McDonald, Tanya J. W.
Diaz-Arias, Luisa
Barron, Bobbie J.
Vizthum, Diane
Cervenka, Mackenzie C.
TI Ketogenic diet therapy provision in the COVID-19 pandemic: Dual-center experience and recommendations
SO EPILEPSY & BEHAVIOR
LA English
DT Article
DE Ketogenic Diet; Pandemic; Telemedicine; Atkins
ID EPILEPSY; TELEMEDICINE; PEOPLE; CARE
AB The current coronavirus-19 pandemic has changed dramatically how neurologists care for children and adults with epilepsy. Stay-at-home orders and resistance to hospitalizations by patients have led epileptologists to engage in telemedicine and reevaluate how to provide elective services. Ketogenic diet therapy is often started in the hospital, with families educated in hospital-based classes, but this is difficult to do in this current pandemic. At our two academic centers, both our pediatric and adult epilepsy diet centers have had to quickly consider alternative methods to both start and maintain ketogenic diet therapy. This paper provides several examples of how ketogenic diet therapy can be provided to patients in unique ways, along with recommendations from other experts and patients, learned over the past few months. (C) 2020 Elsevier Inc. All rights reserved.
C1 [Kossoff, Eric H.] Johns Hopkins Univ, Dept Pediat, Baltimore, MD 21218 USA.
[Turner, Zahava; Adams, Jamie] Johns Hopkins Univ Hosp, Dept Pediat, Div Nutr, Baltimore, MD 21287 USA.
[Bessone, Stacey K.] Johns Hopkins Univ, Sch Med, Dept Nutr Serv, Baltimore, MD 21218

USA.
[Avallone, Jennifer] Johns Hopkins Univ, Sch Med, Johns Hopkins All Childrens Hosp, Inst Brain Protect Sci, St Petersburg, FL USA.
[Kossoff, Eric H.; McDonald, Tanya J. W.; Diaz-Arias, Luisa; Cervenka, Mackenzie C.] Johns Hopkins Univ, Dept Neurol, Baltimore, MD 21218 USA.
[Barron, Bobbie J.; Vizthum, Diane] Johns Hopkins Univ, Sch Med, Inst Clin & Translat Res, Baltimore, MD USA.
C3 Johns Hopkins University; Johns Hopkins University; Johns Hopkins Medicine; Johns Hopkins University; Johns Hopkins University; Johns Hopkins Medicine; Johns Hopkins University; Johns Hopkins University
RP Kossoff, EH (通讯作者), Suite 2158,200 North Wolfe St, Baltimore, MD 21287 USA.
EM ekossoff@jhmi.edu
TC 11
Z9 11
PD OCT
PY 2020
VL 111
AR 107181
DI 10.1016/j.yebeh.2020.107181
WC Behavioral Sciences; Clinical Neurology; Psychiatry
ER

PT J
AU Kuroda, N
AF Kuroda, Naoto
TI Mental health considerations for patients with epilepsy during COVID-19 crisis
SO EPILEPSY & BEHAVIOR
LA English
DT Letter
DE COVID-19; epilepsy; mental health; depression
ID DEPRESSION; STIGMA
C1 [Kuroda, Naoto] Wayne State Univ, Dept Pediat, Childrens Hosp Michigan, Detroit, MI 48202 USA.
C3 Children's Hospital of Michigan; Wayne State University
RP Kuroda, N (通讯作者), Wayne State Univ, Dept Pediat, Childrens Hosp Michigan, Detroit, MI 48202 USA.
EM naoto.kuroda@wayne.edu
TC 11
Z9 12
PD OCT
PY 2020
VL 111
AR 107198
DI 10.1016/j.yebeh.2020.107198
WC Behavioral Sciences; Clinical Neurology; Psychiatry
ER

PT J
AU Kuroda, N
AF Kuroda, Naoto
TI What should we ask patients with epilepsy on telemedicine during the
COVID-19 crisis? A checklist for clinicians
SO EPILEPSY & BEHAVIOR
LA English
DT Letter
DE COVID-19; epilepsy; telemedicine; checklist
ID EXPOSURE
C1 [Kuroda, Naoto] Wayne State Univ, Childrens Hosp Michigan, Dept Pediat, Detroit Med Ctr, 3901 Beaubien St, Detroit, MI 48201 USA.
C3 Children's Hospital of Michigan; Detroit Medical Center; Wayne State
University
RP Kuroda, N (通讯作者), Wayne State Univ, Childrens Hosp Michigan, Dept Pediat, Detroit Med Ctr, 3901 Beaubien St, Detroit, MI 48201 USA.
EM naoto.kuroda@wayne.edu
TC 4
Z9 4
PD OCT
PY 2020
VL 111
AR 107184
DI 10.1016/j.yebeh.2020.107184
WC Behavioral Sciences; Clinical Neurology; Psychiatry
ER

PT J
AU Miller, WR
Von Gaudecker, J
Tanner, A
Buelow, JM
AF Miller, Wendy R.
Von Gaudecker, Jane
Tanner, Andrea
Buelow, Janice M.
TI Epilepsy self-management during a pandemic: Experiences of people with
epilepsy
SO EPILEPSY & BEHAVIOR
LA English
DT Article
DE Epilepsy self-management; COVID-19; Pandemic; Nursing research; management
ID SLEEP
AB The purpose of this descriptive study was to, from the perspective of adult people with epilepsy (PWE) and caregivers of PWE, explore the effects of the current pandemic and resulting societal changes on epilepsy self-management. Ninety-four respondents

completed a mixed-methods quantitative and qualitative survey focused on their epilepsy self-management experiences during the coronavirus disease-19 (COVID-19) pandemic.
Respondents noted significant disruption in epilepsy self-management. Lack of ability to obtain medications or see epilepsy providers, as well as increased stress, social isolation, and changes in routine were all reported as troublesome, and more than one-third of the sample reported an increase in seizure frequency since the onset of the pandemic. Suggestions are given regarding how to support PWE during future COVID-19 outbreaks and to better prepare PWE and their caregivers for any life-altering events, such as a pandemic, with robust self-management skills that will allow them to maintain the highest level of function possible. (C) 2020 Elsevier Inc. All rights reserved.
C1 [Miller, Wendy R.; Von Gaudecker, Jane; Tanner, Andrea; Buelow, Janice M.] Indiana Univ, Sch Nursing, Bloomington, IN 47405 USA.
C3 Indiana University System; Indiana University Bloomington
RP Miller, WR (通讯作者), Indiana Univ, Sch Nursing, Bloomington, IN 47405 USA.
EM wrtruebl@iu.edu
TC 24
Z9 24
PD OCT
PY 2020
VL 111
AR 107238
DI 10.1016/j.yebeh.2020.107238
WC Behavioral Sciences; Clinical Neurology; Psychiatry
ER

PT J
AU Lavin, B
Dormond, C
Scantlebury, MH
Frouin, PY
Brodie, MJ
AF Lavin, Bruce
Dormond, Cassie
Scantlebury, Morris H.
Frouin, Pierre-Yves
Brodie, Martin J.
TI Bridging the healthcare gap: Building the case for epilepsy virtual clinics in the current healthcare environment
SO EPILEPSY & BEHAVIOR
LA English
DT Review
DE Telemedicine; healthcare; Epilepsy
AB Access to quality healthcare remains a challenge that is complicated by mounting pressures to control costs, and now, as we witness, the unprecedented strain placed on our healthcare delivery systems due to the COVID-19 pandemic. Challenges in healthcare access have driven a need for innovative approaches ensuring connectivity

to health providers. Telehealth services and virtual clinics offer accessible disease management pathways for patients living in health resource limited areas or, as in the case of the COVID-19 pandemic, where there may be potential barriers to existing healthcare resources. Those suffering with serious chronic disorders often cannot be seen by a healthcare specialist due to their limited availability, or the lack of a specialist within a reasonable proximity. Epilepsy represents such a disorder where most of the world's population lacks the availability of necessary specialists. Virtual clinics allow for specialist care and an ability to perform necessary ambulatory electroencephalogram (EEG) monitoring by placing the technologies directly in patients' homes or at local clinics near the patients' homes. By moving the diagnostic process out of the hospital or epilepsy center, it becomes possible to overcome growing gaps in neurology services. Virtual clinics have the potential to expand access to high-quality, cost-effective care for the patient. The virtual clinic remotely connects those in need of medical support with specialists anywhere in the world, at any time of the day. Crown Copyright (C) 2020 Published by Elsevier Inc. All rights reserved.
C1 [Lavin, Bruce; Frouin, Pierre-Yves] BioSerenity Inc, Atlanta, GA USA.
[Lavin, Bruce; Frouin, Pierre-Yves] Seren Med Serv, Paris, France.
[Dormond, Cassie] High Lantern Grp, Geneva, Switzerland.
[Scantlebury, Morris H.] Univ Calgary, Dept Pediat, Cumming Sch Med, Alberta Childrens Hosp,Res Inst,Hotchkiss Brain I, Calgary, AB, Canada.
[Scantlebury, Morris H.] Univ Calgary, Dept Clin Neurosci, Cumming Sch Med, Alberta Childrens Hosp,Res Inst,Hotchkiss Brain I, Calgary, AB, Canada.
[Brodie, Martin J.] Univ Glasgow, Epilepsy Unit, Glasgow, Lanark, Scotland.
C3 Alberta Childrens Hospital; University of Calgary; Alberta Childrens Hospital; University of Calgary; University of Glasgow
RP Lavin, B (通讯作者), 3330 Cumberland Blvd,Suite 800, Atlanta, GA 30339 USA.
EM Bruce.lavin@bioserenity.com
TC 9
Z9 9
PD OCT
PY 2020
VL 111
AR 107262
DI 10.1016/j.yebeh.2020.107262
WC Behavioral Sciences; Clinical Neurology; Psychiatry
ER

PT J
AU Sattar, S
Kuperman, R
AF Sattar, Shifteh
Kuperman, Rachel
TI Telehealth in pediatric epilepsy care: A rapid transition during the COVID-19 pandemic
SO EPILEPSY & BEHAVIOR
LA English
DT Review

DE Epilepsy; children; Telemedicine; COVID-19
ID DETECTING MAJOR DEPRESSION; QUALITY-OF-LIFE; TELEMEDICINE; IMPACT;
TELENEUROLOGY; FEASIBILITY; MULTICENTER; ANXIETY
AB Telehealth's first literature reference is an article in 1879 in the Lancet about using the telephone to reduce unnecessary office visits (Institute of Medicine & Board on Health Care Services, 2012). However, providers have been slow to adopt telehealth into their clinical practice secondary to barriers such as cost and reimbursement (Kane and Gillis, 2018) [2]. The advent of shelter in place orders combined with the ongoing need defined by the Centers for Medicare & Medicaid Services (CMS) Administrator Seema Verma "for all Americans, and particularly vulnerable populations who arc at heightened risk, to be able to access their providers" has resulted in the rapid implementation of telehealth across multiple specialties. The goal of this paper is to provide a practical framework for translating quality care in epilepsy as defined by the American Academy of Neurology (AAN) guidelines into a virtual care environment. We will also discuss the use and limitations of point of care testing in epilepsy management. (C) 2020 Elsevier Inc. All rights reserved.
C1 [Sattar, Shifteh] Univ Calif San Diego, Comprehens Epilepsy Ctr, Rady Childrens Hosp, 3020 Childrens Way, San Diego, CA 92123 USA.
[Kuperman, Rachel] Eysz Inc, 107 Sandringham Rd, Piedmont, CA 94611 USA.
C3 Rady Childrens Hospital San Diego; University of California System;
University of California San Diego
RP Kuperman, R (通讯作者), Eysz Inc, 107 Sandringham Rd, Piedmont, CA 94611 USA.
EM ssattar@health.ucsd.edu; rkuperman@eyszlab.com
TC 13
Z9 13
PD OCT
PY 2020
VL 111
AR 107282
DI 10.1016/j.yebeh.2020.107282
WC Behavioral Sciences; Clinical Neurology; Psychiatry
ER

PT J
AU Casciato, S
Di Gennaro, G
AF Casciato, Sara
Di Gennaro, Giancarlo
TI The diagnosis of epilepsy in the COVID-19 era: Dealing with revolution
in clinical practice
SO EPILEPSY & BEHAVIOR
LA English
DT Letter
DE COVID-19; epilepsy; epilepsy care; telemedicine; diagnosis
C1 [Casciato, Sara; Di Gennaro, Giancarlo] IRCCS NEUROMED, Pozzilli, IS, Italy.
C3 IRCCS Neuromed
RP Di Gennaro, G (通讯作者), IRCCS NEUROMED, Pozzilli, IS, Italy.

EM gdigennaro@neuromed.it
TC 0
Z9 0
PD OCT
PY 2020
VL 111
AR 107305
DI 10.1016/j.yebeh.2020.107305
WC Behavioral Sciences; Clinical Neurology; Psychiatry
ER

PT J
AU Cagnazzo, F
Arquizan, C
Derraz, I
Dargazanli, C
Lefevre, PH
Riquelme, C
Gaillard, N
Mourand, I
Gascou, G
Bonafe, A
Costalat, V
AF Cagnazzo, Federico
Arquizan, Caroline
Derraz, Imad
Dargazanli, Cyril
Lefevre, Pierre-Henri
Riquelme, Carlos
Gaillard, Nicolas
Mourand, Isabelle
Gascou, Gregory
Bonafe, Alain
Costalat, Vincent
TI Neurological manifestations of patients infected with the SARS-CoV-2: a systematic review of the literature
SO JOURNAL OF NEUROLOGY
LA English
DT Review
DE COVID-19; SARS-CoV-2; Neurologic manifestation; Acute ischemic stroke
ID CORONAVIRUS DISEASE 2019; CLINICAL CHARACTERISTICS; COVID-19; CHALLENGES; WUHAN
AB Objective To perform an updated review of the literature on the neurological manifestations of COVID-19-infected patients Methods A PRISMA-guideline-based systematic review was conducted on PubMed, EMBASE, and SCOPUS. Series reporting neurological manifestations of COVID-19 patients were studied. Results 39 studies and 68,361 laboratory-confirmed COVID-19 patients were included. Up to 21.3% of COVID-19

patients presented neurological symptoms. Headache (5.4%), skeletal muscle injury (5.1%), psychiatric disorders (4.6%), impaired consciousness (2.8%), gustatory/olfactory dysfunction (2.3%), acute cerebrovascular events (1.4%), and dizziness (1.3%), were the most frequently reported neurological manifestations. Ischemic stroke occurred among 1.3% of COVID-19 patients. Other less common neurological manifestations were cranial nerve impairment (0.6%), nerve root and plexus disorders (0.4%), epilepsy (0.7%), and hemorrhagic stroke (0.15%). Impaired consciousness and acute cerebrovascular events were reported in 14% and 4% of patients with a severe disease, respectively, and they were significantly higher compared to non-severe patients ($p < 0.05$). Individual patient data from 129 COVID-19 patients with acute ischemic stroke (AIS) were extracted: mean age was 64.4 (SD +/- 6.2), 78.5% had anterior circulation occlusions, the mean NIHSS was 15 (SD +/- 7), and the intra-hospital mortality rate was 22.8%. Admission to the intensive care unit (ICU) was required among 63% of patients. Conclusion This updated review of literature, shows that headache, skeletal muscle injury, psychiatric disorders, impaired consciousness, and gustatory/olfactory dysfunction were the most common neurological symptoms of COVID-19 patients. Impaired consciousness and acute cerebrovascular events were significantly higher among patients with a severe infection. AIS patients required ICU admission in 63% of cases, while intra-hospital mortality rate was close to 23%.
C1 [Cagnazzo, Federico; Derraz, Imad; Dargazanli, Cyril; Lefevre, Pierre-Henri; Riquelme, Carlos; Gascou, Gregory; Bonafe, Alain; Costalat, Vincent] Montpellier Univ, Dept Neuroradiol, Hop Gui Chauliac, Med Ctr, 80 Ave Augustin Fliche, Montpellier, France.

[Arquizan, Caroline; Gaillard, Nicolas; Mourand, Isabelle] Montpellier Univ, Dept Neurol, Hop Gui Chauliac, Med Ctr, Montpellier, France.

C3 Universite de Montpellier; CHU de Montpellier; Universite de
Montpellier; CHU de Montpellier

RP Cagnazzo, F (通讯作者), Montpellier Univ, Dept Neuroradiol, Hop Gui Chauliac, Med Ctr, 80 Ave Augustin Fliche, Montpellier, France.

EM f-cagnazzo@chu-montpellier.fr
TC 22
Z9 23
PD AUG
PY 2021
VL 268
IS 8
BP 2656
EP 2665
DI 10.1007/s00415-020-10285-9
EA OCT 2020
WC Clinical Neurology
ER

PT J
AU Valiuddin, HM
Kalajdzic, A
Rosati, J

Boehm, K
Hill, D
AF Valiuddin, Hisham M.
Kalajdzic, Almir
Rosati, James
Boehm, Kevin
Hill, Dominique
TI Update on Neurological Manifestations of SARS-CoV-2
SO WESTERN JOURNAL OF EMERGENCY MEDICINE
LA English
DT Review
DE COVID-19; SARS-CoV-2; meningoencephalitis; epilepsy; neurologic Manifestation
ID COVID-19; INFECTION; CLASSIFICATION; PALSY
AB Severe acute respiratory syndrome coronavirus 2, the source of COVID-19, causes numerous clinical findings including respiratory and gastrointestinal findings. Evidence is now growing for increasing neurological symptoms. This is thought to be from direct in-situ effects in the olfactory bulb caused by the virus. Angiotensin-converting enzyme 2 receptors likely serve as a key receptor for cell entry for most coronaviridae as they are present in multiple organ tissues in the body, notably neurons, and in type 2 alveolar cells in the lung. Hematogenous spread to the nervous system has been described, with viral transmission along neuronal synapses in a retrograde fashion. The penetration of the virus to the central nervous system (CNS) allows for the resulting intracranial cytokine storm, which can result in a myriad of CNS complications. There have been reported cases of associated cerebrovascular accidents with large vessel occlusions, cerebral venous sinus thrombosis, posterior reversible encephalopathy syndrome, meningoencephalitis, acute necrotizing encephalopathy, epilepsy, and myasthenia gravis. Peripheral nervous system effects such as hyposmia, hypogeusia, ophthalmoparesis, Guillain-Barre syndrome, and motor peripheral neuropathy have also been reported. In this review, we update the clinical manifestations of COVID-19 concentrating on the neurological associations that have been described, including broad ranges in both central and peripheral nervous systems.
C1 [Valiuddin, Hisham M.] Univ Penn, Dept Emergency Med, Ground Floor Ravdin, 3400 Spruce St, Philadelphia, PA 19104 USA.
[Kalajdzic, Almir; Rosati, James; Hill, Dominique] St Mary Mercy Hosp, Dept Emergency Med, Livonia, MI USA.
[Boehm, Kevin] Broward Hlth Med Ctr, Dept Emergency Med, Ft Lauderdale, FL USA.
C3 University of Pennsylvania
RP Valiuddin, HM (通讯作者)，Univ Penn, Dept Emergency Med, Ground Floor Ravdin, 3400 Spruce St, Philadelphia, PA 19104 USA.
EM hisham.valiuddin@pennmedicine.upenn.edu
TC 10
Z9 10
PD NOV
PY 2020
VL 21
IS 6
BP 45

EP 51
DI 10.5811/westjem.2020.8.48839
WC Emergency Medicine
ER

PT J
AU Ekstein, D
Noyman, I
Fahoum, F
Herskovitz, M
Linder, I
Ben Zeev, B
Eyal, S
AF Ekstein, Dana
Noyman, Iris
Fahoum, Firas
Herskovitz, Moshe
Linder, Ilan
Ben Zeev, Bruria
Eyal, Sara
TI Treating Epilepsy Patients with Investigational Anti-COVID-19 Drugs: Recommendations by the Israeli Chapter of the ILAE
SO ISRAEL MEDICAL ASSOCIATION JOURNAL
LA English
DT Article
DE anti-COVID-19 medications; antiepileptic drug; clinically-relevant drug-drug interactions; P450; pharmacokinetics
ID ANTIEPILEPTIC DRUGS
AB The coronavirus disease-2019 (COVID-19) and its management in patients with epilepsy can be complex. Prescribers should consider potential effects of investigational anti-COVID-19 drugs on seizures, immunomodulation by anti-seizure medications (ASMs), changes in ASM pharmacokinetics, and the potential for drug-drug interactions (DDIs). The goal of the Board of the Israeli League Against Epilepsy (the Israeli Chapter of the International League Against Epilepsy, ILAE) was to outline the main principles of the pharmacological treatment of COVID-19 in patients with epilepsy. This guide was based on current literature, drug labels, and drug interaction resources. We summarized the available data related to the potential implications of anti-COVID-19 co-medication in patients treated with ASMs. Our recommendations refer to drug selection, dosing, and patient monitoring. Given the limited availability of data, some recommendations are based on general pharmacokinetic or pharmacodynamic principles and might apply to additional future drug combinations as novel treatments emerge. They do not replace evidence-based guidelines, should those become available. Awareness to drug characteristics that increase the risk of interactions can help adjust anti-COVID-19 and ASM treatment for patients with epilepsy. IMAJ 2020; 22: 599-606
C1 [Ekstein, Dana] Hadassah Hebrew Univ Med Ctr, Ginges Ctr Human Neurogenet, Dept Neurol, Jerusalem, Israel.
[Noyman, Iris] Ben Gurion Univ Negev, Soroka Univ Med Ctr, Pediat Neurol Unit, Beer

Sheva, Israel.
[Noyman, Iris] Ben Gurion Univ Negev, Fac Hlth Sci, Beer Sheva, Israel.
[Fahoum, Firas] Tel Aviv Sourasky Med Ctr, Neurol Div, Epilepsy & EEG Unit, Tel Aviv, Israel.
[Herskovitz, Moshe] Rambam Hlth Care Campus, Dept Neurol, Haifa, Israel.
[Herskovitz, Moshe] Technion Fac Med, Haifa, Israel.
[Linder, Ilan] Barzilai Govt Hosp, Pediat Epilepsy & Neurol Serv, Ashqelon, Israel.
[Ben Zeev, Bruria] Safra Pediat Hosp, Sheba Med Ctr, Pediat Neurol Unit, Tel Hashomer, Israel.
[Eyal, Sara] Hebrew Univ Jerusalem, Fac Med, Sch Pharm, Inst Drug Res, Jerusalem, Israel.
[Fahoum, Firas] Tel Aviv Univ, Sackler Fac Med, Tel Aviv, Israel.
C3 Hebrew University of Jerusalem; Ben Gurion University; Soroka Medical Center; Ben Gurion University; Tel Aviv University; Sackler Faculty of Medicine; Tel Aviv Sourasky Medical Center; Rambam Health Care Campus; Technion Israel Institute of Technology; Rappaport Faculty of Medicine; Ben Gurion University; Barzilai Medical Center; Chaim Sheba Medical Center; Hebrew University of Jerusalem; Shamir Medical Center (Assaf Harofeh); Tel Aviv University; Sackler Faculty of Medicine
RP Eyal, S (通讯作者), Hadassah Hebrew Univ Med Ctr, Inst Drug Res, Sch Pharm, Fac Med, IL-91120 Jerusalem, Israel.; Ekstein, D (通讯作者), Hadassah Hebrew Univ Med Ctr, Dept Neurol, IL-91120 Jerusalem, Israel.
EM dekstein@hadassah.org.il; sarae@ekmd.huji.ac.il
TC 0
Z9 0
PD NOV
PY 2020
VL 22
IS 11
BP 599
EP 606
WC Medicine, General & Internal
ER

PT J
AU Nikbakht, F
Mohammadkhanizadeh, A
Mohammadi, E
AF Nikbakht, Farnaz
Mohammadkhanizadeh, Ali
Mohammadi, Ekram
TI How does the COVID-19 cause seizure and epilepsy in patients? The potential mechanisms
SO MULTIPLE SCLEROSIS AND RELATED DISORDERS
LA English
DT Review
DE Covid-19; Seizure; Epilepsy; Pandemic

ID SYNAPTIC PLASTICITY; CALCIUM; CYTOKINES; RECEPTOR
AB The new coronavirus has spread throughout the world in a very short time and now has become a pandemic. Most infected people have symptoms such as dry cough, dyspnea, tiredness, and fever. However, the Covid-19 infection disrupts various organs, including the liver, kidney, and nervous system. Common neurological symptoms of the Covid-19 infection include delirium, confusion, headache, and loss of sense of smell and taste. In rare cases it can cause stroke and epilepsy. The virus enters the nervous system either directly through nerve pathways or indirectly through the ACE2 receptor. The neurological symptoms of a Covid-19 infection in the brain are mainly due to either the entry of pro-inflammatory cytokines into the nervous system or the production of these cytokines by microglia and astrocytes. Pro-inflammatory cytokines can cause blood-brain barrier disruption, increase in glutamate and aspartate and reduce GABA levels, impairs the function of ion channels, and finally, high levels of cytokines can cause epilepsy. Understanding the potential mechanisms is necessary to gain better insight into COVID-19 induced seizure pathogenesis and to design the correct treatment strategies to achieve appropriate treatment for seizure and epilepsy.
C1 [Mohammadkhanizadeh, Ali] Iran Univ Med Sci, Cellular & Mol Res Ctr, Sch Med, Tehran, Iran.
Iran Univ Med Sci, Dept Physiol, Sch Med, Tehran, Iran.
C3 Iran University of Medical Sciences; Iran University of Medical Sciences
RP Mohammadkhanizadeh, A (通讯作者), Iran Univ Med Sci, Cellular & Mol Res Ctr, Sch Med, Tehran, Iran.
EM Ali_mohammadkhanizadeh@yahoo.com
TC 36
Z9 39
PD NOV
PY 2020
VL 46
AR 102535
DI 10.1016/j.msard.2020.102535
WC Clinical Neurology
ER

PT J
AU Daniel, F
Zoltan, H
Peter, K
Anita, K
AF Daniel, Fabo
Zoltan, Horvath
Peter, Klivenyi
Anita, Kamondi
TI Changes in epilepsy care during the first medical emergency period of COVID-19 pandemic in Hungary A questionnaire survey
SO ORVOSI HETILAP
LA Hungarian
DT Article

DE COVID-19; epilepsy; management; questionnaire survey; care; emergency

AB Introduction: COVID-19 pandemic has transformed the operation of outpatient care worldwide for months. The new framework was set in Hungary by the health emergency regulations that existed from 11. 03. 2020 to 17. 06. 2020.

Objective: In the second half of the emergency period, between 22. 04. 2020 and 05. 05. 2020, we surveyed the opinion of physicians involved in epilepsy care about the extent to which patient care had changed and how they experienced the changes in person.

Method: An internet questionnaire survey was conducted among neurologists registered for the annual congress of the Hungarian Chapter of the International League Against Epilepsy. Nine singleor multiple-choice questions and 'free comment' fields were available.

Results: Of 116 neurologists contacted, 33 responded (28%), and a total of 30 comments were recorded. 73% said the changes caused a serious difficulty, 15% thought it would have serious consequences. Reception of new patients was stopped in 53%, and 25% encountered great difficulties. In 49%, the problems of the cared patients could be solved using remote visits, but 24% could not solve them properly. 68% of outpatient visits took the form of documented telephone conversations. Two-thirds of doctors feared catching the virus, 40% of whom felt they were not getting enough protection. 6% caught the infection.

Conclusion: The COVID-19 pandemic has mostly affected the issuance of new expert opinions, but care tasks have not always been adequately addressed. The damage was significantly mitigated by the flexibility of the care staff. Telephone visits, if necessary, can replace personal doctor-patient encounters in epileptology. The technical development of remote visit applications and their documentation issues are important. During the pandemic period, great care must be taken to protect staff in order to avoid infection and increase the sense of safety of doctors.

C1 [Daniel, Fabo; Anita, Kamondi] Orszagos Klin Idegtudomanyi Int, Neurol Osztaly, Budapest, Hungary.

[Daniel, Fabo; Zoltan, Horvath; Peter, Klivenyi] Szegedi Tudomanyegyet, Altalanos Orvostudomanyi Kar, Neurol Klin, Szeged, Hungary.

C3 Szeged University

RP Daniel, F (通讯作者), Orszagos Klin Idegtudomanyi Int, Neurol Osztaly, Budapest, Hungary.; Daniel, F (通讯作者), Szegedi Tudomanyegyet, Altalanos Orvostudomanyi Kar, Neurol Klin, Szeged, Hungary.

EM fabo.daniel@gmail.com

TC 0

Z9 0

PD NOV

PY 2020

VL 161

IS 46

BP 1939

EP 1943

DI 10.1556/650.2020.32003

WC Medicine, General & Internal

ER

PT J
AU Roberto, KT
Espiritu, AI
Fernandez, MLL
Gutierrez, JC
AF Roberto, Katrina T.
Espiritu, Adrian, I
Fernandez, Marc Laurence L.
Gutierrez, Josephine C.
TI Electroencephalographic findings in COVID-19 patients: A systematic review
SO SEIZURE-EUROPEAN JOURNAL OF EPILEPSY
LA English
DT Review
DE Electroencephalography; EEG; COVID-19; Coronavirus; Seizure; Epilepsy
ID EEG
AB Background: Growing evidence of neurologic involvement seen in COVID-19 infection necessitates the pooling of neurodiagnostic findings like electroencephalography (EEG) that may guide clinical management. The objective of this study was to review the EEG findings in patients diagnosed with COVID-19 infection through a systematic review of published articles.

Methods: We systematically searched until July 25, 2020 for published articles that reported on descriptive EEG findings in patients diagnosed with COVID-19 in PUBMED by Medline, EMBASE, and CENTRAL by the Cochrane Library.

Results: From a total of 94 identified records, 29 relevant articles were included in this review. A total of 177 patients with COVID-19 with descriptive EEG reports were analyzed. The most common indication for EEG was unexplained altered mental status. Disturbances of background activity such as generalized and focal slowing were seen as well epileptiform abnormalities and rhythmic or periodic discharges. There were no consistent EEG findings specific to COVID-19 infection.

Conclusion: The EEG findings in COVID-19 appear to be non-specific. Further research on the relationship of the EEG findings to the clinical state and shortor long-term prognosis of COVID-19 patients may be conducted to help clinicians discern which patients would necessitate an EEG procedure and would eventually require treatment.
C1 [Roberto, Katrina T.; Espiritu, Adrian, I; Fernandez, Marc Laurence L.; Gutierrez, Josephine C.] Univ Philippines Manila, Coll Med & Philippine Gen Hosp, Dept Neurosci, Taft Ave, Manila 1000, Philippines.

[Espiritu, Adrian, I] Univ Philippines Manila, Coll Med, Dept Clin Epidemiol, Taft Ave, Manila 1000, Philippines.
C3 University of the Philippines System; University of the Philippines Manila; University of the Philippines System; University of the Philippines Manila
RP Roberto, KT (通讯作者), Univ Philippines Manila, Coll Med & Philippine Gen Hosp, Dept Neurosci, Taft Ave, Manila 1000, Philippines.
EM ktroberto@up.edu.ph; aiespiritu@up.edu.ph; mlfernandez@up.edu.ph; jcgutierrez4@up.edu.ph
TC 9

Z9 9
PD NOV
PY 2020
VL 82
BP 17
EP 22
WC Clinical Neurology; Neurosciences
ER

PT J
AU Cheli, M
Dinoto, A
Olivo, S
Tomaselli, M
Stokelj, D
Cominotto, F
Brigo, F
Manganotti, P
AF Cheli, Marta
Dinoto, Alessandro
Olivo, Sasha
Tomaselli, Marinella
Stokelj, David
Cominotto, Franco
Brigo, Francesco
Manganotti, Paolo
TI SARS-CoV-2 pandemic and epilepsy: The impact on emergency department attendances for seizures
SO SEIZURE-EUROPEAN JOURNAL OF EPILEPSY
LA English
DT Article
DE SARS-CoV-2; Epilepsy; Management
AB Introduction: The risk of acquiring SARS-CoV-2 in a hospital setting and the need of reorganizing the Emergency Departments (EDs) to cope with infected patients have led to a reduction of ED attendances for non-infectious acute conditions and to a different management of chronic disorders.

Methods: We performed a retrospective study evaluating the frequency and features of ED attendances for sei-zures during the lockdown period (March 10th-April 30th 2020) in the University Hospital of Trieste, Italy. We studied the possible pandemic impact on the way patients with seizures sought for medical assistance by comparing the lockdown period to a matched period in 2019 and to a period of identical length preceding the lockdown (January 18th-March 9th 2020).

Results: A striking decrease in total ED attendances was observed during lockdown (4664) compared to the matched control (10424) and to the pre-lockdown (9522) periods. A similar reduction, although to a lesser extent, was detected for seizure attendances to the ED: there were 37 during lockdown and 63 and 44 respectively during the two other periods. Intriguingly, during the lockdown a higher number of patients attended the

ED with first seizures (p = 0.013), and more EEGs (p = 0.008) and CT brain scans (p = 0.018) were performed; there was a trend towards more frequent transport to the ED by ambulance (p = 0.061) in the lockdown period.

Conclusions: Our data suggest that the pandemic has affected the way patients with seizures access the Health Care System.

C1 [Cheli, Marta; Dinoto, Alessandro; Olivo, Sasha; Tomaselli, Marinella; Stokelj, David; Manganotti, Paolo] Univ Trieste, Cattinara Univ Hosp ASUGI, Clin Unit Neurol, Dept Med Surg & Hlth Sci, Str Fiume 447, I-34149 Trieste, Italy.

[Cominotto, Franco] Univ Hosp & Hlth Serv Trieste, Cattinara Univ Hosp ASUGI, Clin Unit Neurol, Dept Med Surg & Hlth Sci, Str Fiume 447, I-34149 Trieste, Italy.

[Brigo, Francesco] Franz Tappeiner Hosp, Div Neurol, Merano, Bolzano, Italy.

C3 University of Trieste; University of Trieste; Ospedale Franz Tappeiner

RP Manganotti, P (通讯作者), Osped Cattinara, Clin Neurol, Str Fiume 447, I-34149 Trieste, Italy.

EM paolo.manganotti@asugi.sanita.fvg.it

TC 10

Z9 10

PD NOV

PY 2020

VL 82

BP 23

EP 26

DI 10.1016/j.seizure.2020.08.008

WC Clinical Neurology; Neurosciences

ER

PT J

AU Alkhotani, A
Siddiqui, MI
Almuntashri, F
Baothman, R

AF Alkhotani, Amal
Siddiqui, Muhammad, I
Almuntashri, Fahad
Baothman, Renad

TI The effect of COVID-19 pandemic on seizure control and self-reported stress on patient with epilepsy

SO EPILEPSY & BEHAVIOR

LA English

DT Article

DE Epilepsy; recurrence; COVID-19; Self-reported stress; Pandemic

ID TRIGGER; PEOPLE

AB Objectives: The objective of the study was to assess if patients with epilepsy (PWE) experienced an increase in seizure frequency and self-reported stress during the COVID-19 pandemic.

Methods: This is a cross-sectional study conducted in Saudi Arabia in April 2020. An electronic self-administered questionnaire was distributed to PWE via their treating

neurologist. The variables included were demographic and baseline clinical characteristics (age, gender, living situation, occupational status, type of epilepsy, duration of epilepsy, number of antiepileptic medications (AEDs), presence of known psychiatric illness, and use of psychiatric medications), their seizure control in the month prior to the pandemic, perceived stress during this period of time, sleep changes, compliance changes, and change in seizure control during the pandemic.

Results: A total of 156 patients completed the questionnaire, with 29.5% reporting an increase in seizure frequency. Additionally, 59.4% reported an increase in self-reported stress and 71.2% experienced a significant change in their sleep during this period. Higher baseline seizure frequency, more AEDs, noncompliance, increase in self-reported stress, and sleep changes are the significant factors associated with increase in seizure frequency during the pandemic.

Conclusion: Identifying high-risk patients for seizure recurrence is important in order to provide them with adequate support to reduce such risk. (C) 2020 Elsevier Inc. All rights reserved.
C1 [Alkhotani, Amal] Umm AlQura Univ, Dept Med, Mecca, Saudi Arabia.
[Siddiqui, Muhammad, I] Umm AlQura Univ, Dept Community Med, Mecca, Saudi Arabia.
[Almuntashri, Fahad; Baothman, Renad] Umm AlQura Univ, Coll Med, Mecca, Saudi Arabia.
C3 Umm Al Qura University; Umm Al Qura University; Umm Al Qura University
RP Alkhotani, A (通讯作者), Umm AlQura Univ, Dept Med, Coll Med, Mecca 24354, Saudi Arabia.
EM amkhotani@uqu.edu.sa
TC 38
Z9 38
PD NOV
PY 2020
VL 112
AR 107323
DI 10.1016/j.yebeh.2020.107323
WC Behavioral Sciences; Clinical Neurology; Psychiatry
ER

PT J
AU Cabona, C
Deleo, F
Marinelli, L
Audenino, D
Arnaldi, D
Rossi, F
Di Giacomo, R
Buffoni, C
Rosa, GJ
Didato, G
Arboscello, E
de Curtis, M
Villani, F

AF Cabona, Corrado
Deleo, Francesco
Marinelli, Lucio
Audenino, Daniela
Arnaldi, Dario
Rossi, Francesca
Di Giacomo, Roberta
Buffoni, Claudia
Rosa, Giuseppa Jolanda
Didato, Giuseppe
Arboscello, Eleonora
de Curtis, Marco
Villani, Flavio
TI Epilepsy course during COVID-19 pandemic in three Italian epilepsy centers
SO EPILEPSY & BEHAVIOR
LA English
DT Article
DE Epilepsy; COVID-19; seizure; Status epilepticus; Telemedicine
ID SEIZURES
AB During epidemic outbreaks, epilepsy course can be modified by different physical and psychological stressors and, most importantly, by irregular therapy intake.
The effect of COVID-19 and quarantine isolation on the course of epilepsy and on incidence of new-onset seizures is still unclear.
With the aim of managing epilepsy in quarantined patients, three Italian Epilepsy Centers set up telephone consultations using a semistructured interview, allowing a prospective collection of data on seizure course and other seizure-related problems during pandemic. The collected data on seizure course were compared with the analogous period of 2019.
The level of patients' concern relating to the COVID-19 pandemic was also assessed using a numeric rating scale. To address the effect of COVID-19 pandemic on seizure incidence, data collection included the number of consultations for first seizures, relapse seizures, and status epilepticus (SE) in the emergency department of one of the participating centers.
Clinical telephone interviews suggest the absence of quarantine effect on epilepsy course in our cohort. No differences in incidence of emergency consultations for seizures over a two-month period were also observed compared with a control period.
As demonstrated in other infective outbreaks, good antiepileptic drug (AED) supplying, precise information, and reassurance are the most important factors in chronic conditions to minimize psychological and physical stress, and to avoid unplanned treatment interruptions. (C) 2020 Elsevier Inc. All rights reserved.
C1 [Cabona, Corrado; Marinelli, Lucio; Villani, Flavio] IRCCS Osped Policlin San Martino, Div Clin Neurophysiol, Largo Rosanna Benzi 10, Genoa, Italy.
[Cabona, Corrado; Marinelli, Lucio; Villani, Flavio] IRCCS Osped Policlin San Martino, Epilepsy Ctr, Largo Rosanna Benzi 10, Genoa, Italy.
[Deleo, Francesco; Di Giacomo, Roberta; Rosa, Giuseppa Jolanda; Didato, Giuseppe; de Curtis, Marco] Fdn IRCCS Ist Neurol C Besta, Epilepsy Unit, Via Celoria, Milan, Italy.

[Audenino, Daniela] EO Osped Galliera, Neurol Unit, Via Mura delle Cappuccine 14, Genoa, Italy.
[Arnaldi, Dario] IRCCS Osped Policlin San Martino, Neurol Clin, Largo Rosanna Benzi 10, Genoa, Italy.
[Marinelli, Lucio; Arnaldi, Dario] Univ Genoa, Dept Neurosci DINOGMI, Via Balbi 5, Genoa, Italy.
[Rossi, Francesca; Arboscello, Eleonora] IRCCS Osped Policlin San Martino, Emergency Dept, Largo Rosanna Benzi 10, Genoa, Italy.
[Buffoni, Claudia] IRCCS Osped Policlin San Martino, Anesthesia & Intens Care, Largo Rosanna Benzi 10, Genoa, Italy.
[Rosa, Giuseppa Jolanda] Azienda Osped Univ St Anna di Cona, Epilepsy Ctr, Via Aldo Moro 8, I-44124 Ferrara, Italy.
C3 IRCCS Istituto Neurologico Besta; Ente Ospedaliero Ospedali Galliera; University of Genoa; University of Ferrara; Arcispedale Sant'Anna
RP Cabona, C (通讯作者), IRCCS Osped Policlin San Martino, Div Clin Neurophysiol, Largo Rosanna Benzi 10, Genoa, Italy.; Cabona, C (通讯作者), IRCCS Osped Policlin San Martino, Epilepsy Ctr, Largo Rosanna Benzi 10, Genoa, Italy.
EM corrado.cabona@gmail.com
TC 10
Z9 11
PD NOV
PY 2020
VL 112
AR 107375
DI 10.1016/j.yebeh.2020.107375
WC Behavioral Sciences; Clinical Neurology; Psychiatry
ER

PT J
AU Casares, M
Wombles, C
Skinner, HJ
Westerveld, M
Gireesh, ED
AF Casares, Maritsa
Wombles, Christina
Skinner, Holly J.
Westerveld, Michael
Gireesh, Elakkat D.
TI Telehealth perceptions in patients with epilepsy and providers during the COVID-19 pandemic
SO EPILEPSY & BEHAVIOR
LA English
DT Article
DE COVID-19; Epilepsy; Telemedicine; Seizure
AB Coronavirus disease 2019 (COVID-19) has required novel solutions for issues that arise with social distancing. Telehealth has become one of those solutions in many

clinics around the US. As we look beyond, the pandemic telehealth can be utilized as an important tool for clinics in the future. Patient satisfaction will most likely affect reimbursement, while provider perception will affect implementation. We see this as a valuable tool to many epilepsy clinics. The goal of our survey was to determine the perceptions and satisfaction of patients with intractable epilepsy and providers with telehealth during the COVID-19 pandemic; we surveyed patient and providers. We evaluated the first 111 patients who participated in our telehealth visits. We conducted telephone surveys with the first 68 patients who agreed to participate. We also conducted surveys by email with our providers who participated in these telehealth visits. We found that 66% of patients and 67% of providers would use a telehealth visit in the future if given the option. Review of our patients' and providers' comments provides valuable insights for building a long-term successful intractable epilepsy telehealth clinic. (C) 2020 Elsevier Inc. All rights reserved.
C1 [Casares, Maritsa] AdventHlth Orlando Neurosci Inst, 615 E Princeton St,Suite 540, Orlando, FL 32803 USA.
[Wombles, Christina; Skinner, Holly J.; Gireesh, Elakkat D.] AdventHlth Epilepsy Orlando, 615 E Princeton St,Suite 540, Orlando, FL 32803 USA.
[Westerveld, Michael] AdventHlth Neuropsychol Orlando, 615 E Princeton St,Suite 101, Orlando, FL 32803 USA.
C3 Adventist Health Services; AdventHealth; (AdventHealth) Central Florida Division; Central Florida Hospital - South; AdventHealth Orlando; Adventist Health Services; AdventHealth; (AdventHealth) Central Florida Division; Central Florida Hospital - South; AdventHealth Orlando; Adventist Health Services; AdventHealth; (AdventHealth) Central Florida Division; Central Florida Hospital - South; AdventHealth Orlando
RP Casares, M (通讯作者), AdventHlth Orlando Neurosci Inst, 615 E Princeton St,Suite 540, Orlando, FL 32803 USA.
EM Maritsa.casares@adventhealth.com;
Christina.Wombles.ARNP@adventhealth.com;
Holly.Skinner.DO@AdventHealth.com; Michael.Westerveld@AdventHealth.com;
Elakkat.GIREESH.MD@AdventHealth.com
TC 14
Z9 14
PD NOV
PY 2020
VL 112
AR 107394
DI 10.1016/j.yebeh.2020.107394
WC Behavioral Sciences; Clinical Neurology; Psychiatry
ER

PT J
AU de Almeida, ACG
Cysneiros, RM
Scorza, CA
Finsterer, J
Scorza, FA

AF de Almeida, Antonio-Carlos G.
   Cysneiros, Roberta M.
   Scorza, Carla A.
   Finsterer, Josef
   Scorza, Fulvio A.
TI Doctors race to understand epilepsy in the time of COVID-19
SO EPILEPSY & BEHAVIOR
LA English
DT Letter
DE Epilepsy; COVID-19; SARS-CoV-2; neurological disease; sudden unexpected death
ID SUDDEN UNEXPECTED DEATH
C1 [de Almeida, Antonio-Carlos G.] Univ Fed Sao Joao del Rei UFSJ, Dept Engn Biossistemas, Lab Neurociencia Expt & Computac, Sao Joao Del Rei, MG, Brazil.
   [Cysneiros, Roberta M.] Univ Presbiteriana Mackenzie, Ctr Ciencias Biol & Saude, Programa Posgrad Disturbios Desenvolvimento, Sao Paulo, Brazil.
   [Scorza, Carla A.; Scorza, Fulvio A.] Univ Fed Sao Paulo EPM UNIFESP, Escola Paulista Med, Disciplina Neurociencia, Sao Paulo, Brazil.
   [Finsterer, Josef] Messerli Inst, Krankenanstalt Rudolfstiftung, Vienna, Austria.
C3 Universidade Federal de Sao Joao del-Rei; Universidade Presbiteriana
   Mackenzie; Universidade Federal de Sao Paulo (UNIFESP); Rudolfstiftung
   Hospital
RP Scorza, FA (通讯作者), Rua Pedro de Toledo 669,1 Andar, BR-04039032 Sao Paulo, SP, Brazil.
EM scorza@unifesp.br
TC 0
Z9 0
PD NOV
PY 2020
VL 112
AR 107356
DI 10.1016/j.yebeh.2020.107356
WC Behavioral Sciences; Clinical Neurology; Psychiatry
ER

PT J
AU Kuchenbuch, M
   D'Onofrio, G
   Wirrell, E
   Jiang, YW
   Dupont, S
   Grinspan, ZM
   Auvin, S
   Wilmshurst, JM
   Arzimanoglou, A
   Cross, JH
   Specchio, N
   Nabbout, R

AF Kuchenbuch, Mathieu
D'Onofrio, Gianluca
Wirrell, Elaine
Jiang, Yuwu
Dupont, Sophie
Grinspan, Zachary M.
Auvin, Stephane
Wilmshurst, Jo M.
Arzimanoglou, Alexis
Cross, J. Helen
Specchio, Nicola
Nabbout, Rima
TI An accelerated shift in the use of remote systems in epilepsy due to the COVID-19 pandemic
SO EPILEPSY & BEHAVIOR
LA English
DT Article
DE Telemedicine; E-health; E-teaming; Virtual meeting; Remote work system; Teleconsultation
ID CLASS ATTENDANCE; TELEMEDICINE; CARE; PERFORMANCE; SUCCESS
AB Purpose: The purpose of the study was to describe epileptologists' opinion on the increased use of remote systems implemented during the COVID-19 pandemic across clinics, education, and scientific meetings activities.

Methods: Between April and May 2020, we conducted a cross-sectional, electronic survey on remote systems use before and during the COVID-19 pandemic through the European reference center for rare and complex epilepsies (EpiCARE) network, the International and the French Leagues Against Epilepsy, and the International and the French Child Neurology Associations. After descriptive statistical analysis, we compared the results of France, China, and Italy.

Results: One hundred and seventy-two respondents from 35 countries completed the survey. Prior to the COVID-19 pandemic, 63.4% had experienced remote systems for clinical care. During the pandemic, the use of remote clinics, either institutional or personal, significantly increased ($p < 10(-4)$). Eighty-three percent used remote systems with video, either institutional (75%) or personal (25%). During the pandemic, 84.6% of respondents involved in academic activities transformed their courses to online teaching. From February to July 2020, few scientific meetings relevant to epileptologists and routinely attended was adapted to virtual meeting (median: 1 [25th-75th percentile: 0-2]). Responders were quite satisfied with remote systems in all three activity domains. Interestingly, before the COVID-19 pandemic, remote systems were significantly more frequently used in China for dinical activity compared with France or Italy. This difference became less marked during the pandemic.

Conclusion: The COVID-19 pandemic has dramatically altered how academic epileptologists carry out their core missions of clinical care, medical education, and scientific discovery and dissemination. Close attention to the impact of these changes is merited. (C) 2020 Elsevier Inc. All rights reserved.
C1 [Kuchenbuch, Mathieu; D'Onofrio, Gianluca; Nabbout, Rima] Univ Paris, Reference Ctr Rare Epilepsies, Dept Pediat Neurol, Hop Necker Enfants Malad,AP HP, Paris, France.

[Kuchenbuch, Mathieu; Nabbout, Rima] Univ Paris, Imagine Inst, Lab Translat Res Neurol Disorders, INSERM,UMR 1163, Paris, France.
[D'Onofrio, Gianluca] Univ Padua, Dept Women & Child Hlth, Pediat Residency Program, Padua, Italy.
[Wirrell, Elaine] Mayo Clin, Div Child & Adolescent Neurol, Dept Neurol, Rochester, MN USA.
[Jiang, Yuwu] Peking Univ First Hosp, Dept Pediat, Beijing, Peoples R China.
[Jiang, Yuwu] Peking Univ First Hosp, Pediat Epilepsy Ctr, Beijing, Peoples R China.
[Dupont, Sophie] Hop la Pitie Salpetriere, AP HP, Epilepsy Unit, Paris, France.
[Dupont, Sophie] Hop la Pitie Salpetriere, AP HP, Rehabil Unit, Paris, France.
[Dupont, Sophie] UMPC, Ctr Rech, Inst Cerveau & Mode Epiniere ICM, UMR 7225,CNRS,UMRS 975,Inserm, Paris, France.
[Grinspan, Zachary M.] Weill Cornell Med, Dept Populat Hlth Sci, New York, NY USA.
[Grinspan, Zachary M.] Weill Cornell Med, Dept Pediat, New York, NY USA.
[Auvin, Stephane] Hop Robert Debre, AP HP, Dept Pediat Neurol, Paris, France.
[Wilmshurst, Jo M.] Univ Cape Town, Red Cross War Mem Childrens Hosp, Neurosci Inst, Paediat Neurol Dept, Cape Town, South Africa.
[Arzimanoglou, Alexis] Univ Hosp Lyon, Dept Paediat Epilepsy Sleep Disorders & Funct Neu, Lyon, France.
[Arzimanoglou, Alexis] ERN EpiCARE, Lyon, France.
[Arzimanoglou, Alexis] Univ Barcelona, San Juan Dios Childrens Hosp, Epilepsy Unit, Barcelona, Spain.
[Cross, J. Helen] UCL NIHR BRC Great Ormond St Inst Child Hlth, London WC1N 1EH, England.
[Specchio, Nicola] Bambino Gesu Pediat Hosp, IRCCS, Dept Neurosci, Rare & Complex Epilepsy Unit, Rome, Italy.
[Specchio, Nicola] European Reference Network EpiCARE, Rome, Italy.
[Dupont, Sophie] Univ Paris Sorbonne, Paris, France.
C3 Assistance Publique Hopitaux Paris (APHP); Hopital Universitaire Necker-Enfants Malades - APHP; UDICE-French Research Universities; Universite de Paris; Institut National de la Sante et de la Recherche Medicale (Inserm); UDICE-French Research Universities; Universite de Paris; University of Padua; Mayo Clinic; Peking University; Assistance Publique Hopitaux Paris (APHP); Hopital Universitaire Pitie-Salpetriere - APHP; League of European Research Universities - LERU; UDICE-French Research Universities; Sorbonne Universite; Assistance Publique Hopitaux Paris (APHP); Hopital Universitaire Pitie-Salpetriere - APHP; League of European Research Universities - LERU; UDICE-French Research Universities; Sorbonne Universite; Centre National de la Recherche Scientifique (CNRS); CNRS - National Institute for Biology (INSB); Institut National de la Sante et de la Recherche Medicale (Inserm); UDICE-French Research Universities; League of European Research Universities - LERU; Sorbonne Universite; Universite de Paris; Cornell University; Cornell University; Assistance Publique Hopitaux Paris (APHP); Hopital Universitaire Robert-Debre - APHP; UDICE-French Research Universities; Universite de Paris; University of Cape Town; CHU Lyon; League of European Research Universities - LERU; University of

Barcelona; IRCCS Bambino Gesu; UDICE-French Research Universities; League of European Research Universities - LERU; Sorbonne Universite
RP Nabbout, R (通讯作者), Necker Enfants Malad Hosp, Reference Ctr Rare Epilepsies, Dept Pediat Neurol, F-75015 Paris, France.
EM rimanabbout@aphp.fr
TC 18
Z9 18
PD NOV
PY 2020
VL 112
AR 107376
DI 10.1016/j.yebeh.2020.107376
WC Behavioral Sciences; Clinical Neurology; Psychiatry
ER

PT J
AU Salari, M
Etemadifar, M
Gharagozli, K
Etemad, K
Ashrafi, F
Ashourizadeh, H
AF Salari, Mehri
Etemadifar, Masoud
Gharagozli, Koroush
Etemad, Koorosh
Ashrafi, Farzad
Ashourizadeh, Helia
TI Incidence of anxiety in epilepsy during coronavirus disease (COVID-19) pandemic
SO EPILEPSY & BEHAVIOR
LA English
DT Article
DE Epilepsy; Anxiety; COVID-19; Pandemic
ID DISORDERS
AB Purpose: The coronavirus disease 2019 (COVID-19) pandemic has affected people globally, and people with chronic diseases are suffering more in maintaining their mental and physical health.
Method: This cross-sectional, case-control study assessed the anxiety level in people with epilepsy compared with the general population.
Results: The results showed that 13.5% of patients had experienced a severe level of anxiety, but the mean anxiety level between groups did not show significant difference.
Conclusion: Although still many aspects of the pandemic on people with epilepsy are yet to be determined, active investigation of psychological sequels of the pandemic is demanded. (C) 2020 Elsevier Inc. All rights reserved.
C1 [Salari, Mehri; Ashrafi, Farzad; Ashourizadeh, Helia] Shahid Beheshti Univ Med Sci,

Funct Neurosurg Res Ctr, Shohada Tajrish Neurosurg Ctr Excellence, Tehran, Iran.
[Etemadifar, Masoud] Isfahan Univ Med Sci, Med Sch, Dept Funct Neurosurg, Esfahan, Iran.
[Gharagozli, Koroush] Shahid Beheshti Univ Med Sci, Loghman Med & Educ Ctr, Tehran, Iran.
[Etemad, Koorosh] Shahid Beheshti Univ Med Sci, Sch Publ Hlth & Safety, Dept Epidemiol, Tehran, Iran.
C3 Shahid Beheshti University Medical Sciences; Isfahan University Medical Science; Shahid Beheshti University Medical Sciences; Shahid Beheshti University Medical Sciences
RP Ashourizadeh, H (通讯作者), Shohada Tajrish Hosp, Dept Neurol, Tehran, Iran.
EM ashourizadehhelia@sbmu.ac.ir
TC 9
Z9 9
PD NOV
PY 2020
VL 112
AR 107442
DI 10.1016/j.yebeh.2020.107442
WC Behavioral Sciences; Clinical Neurology; Psychiatry
ER

PT J
AU Saleem, T
Sheikh, N
Abbasi, MH
Javed, I
Khawar, MB
AF Saleem, Tayyaba
Sheikh, Nadeem
Abbasi, Muddasir Hassan
Javed, Iram
Khawar, Muhammad Babar
TI COVID-19 containment and its unrestrained impact on epilepsy management in resource-limited areas of Pakistan
SO EPILEPSY & BEHAVIOR
LA English
DT Article
DE COVID-19; Epilepsy; Outcome; Lockdown; Telemedicine
ID CHALLENGES
AB The current pandemic of coronavirus disease 2019 (COVID-19) that led to an unprecedented crisis with significant health, social, and economic repercussions presented more serious concerns for those living with some chronic conditions such as epilepsy. This study was aimed to find out impact of the COVID-19 pandemic on management of epilepsy. A cross-sectional study was conducted through telephone interviews, targeting 213 caregivers of pediatric patients with epilepsy, belonging to underserved areas of Faisalabad, Punjab, Pakistan. A simple questionnaire was designed to record

the responses of participants relevant to the direct and indirect effects of COVID-19 pandemic and their knowledge about possible ways that can be accessed for the management of epilepsy during an ongoing pandemic. The current study, which holds 77% of the respondents from rural areas and 23% from urban settings, showed that partial measures of lockdown taken to stop or slow the spread of COVID-19 resulted in adverse economic and health outcomes in the said population including cancelation of follow-up visits, worsening of seizures, job loss, burden of antiepileptic drugs (AEDs) costs, and discontinuation of medicines. Furthermore, knowledge about alternative ways to access health facilities was found very poor among caregivers. Income sources of poor people disrupted by lockdown can lead to unintentional nonadherence to medication, which is a clear picture of inequitable distribution of resources. This study highlights the major issues faced by the caregivers during this ongoing pandemic of COVID-19. (C) 2020 Elsevier Inc. All rights reserved.
C1 [Saleem, Tayyaba; Sheikh, Nadeem; Khawar, Muhammad Babar] Univ Punjab, Dept Zool, Cell & Mol Biol Lab, Lahore 54590, Pakistan.
[Abbasi, Muddasir Hassan] Univ Okara, Dept Zool, Renala Khurd 56300, Pakistan.
[Javed, Iram] Children Hosp & Inst Child Hlth Faisalabad, Dept Pediat Neurol, Faisalabad 38000, Pakistan.
[Khawar, Muhammad Babar] Chinese Acad Sci, Inst Zool, State Key Lab Stem Cell & Reprod Biol, Beijing, Peoples R China.
[Khawar, Muhammad Babar] Univ Chinese Acad Sci, Beijing 100049, Peoples R China.
C3 University of Punjab; Chinese Academy of Sciences; Institute of Zoology, CAS; Chinese Academy of Sciences; University of Chinese Academy of Sciences, CAS
RP Sheikh, N (通讯作者), Univ Punjab, Dept Zool, Cell & Mol Biol Lab, Lahore 54590, Pakistan.
EM nadeem.zool@pu.edu.pk
TC 6
Z9 6
PD NOV
PY 2020
VL 112
AR 107476
DI 10.1016/j.yebeh.2020.107476
WC Behavioral Sciences; Clinical Neurology; Psychiatry
ER

PT J
AU Sanchez-Larsen, A
Gonzalez-Villar, E
Diaz-Maroto, I
Layos-Romero, A
Martinez-Martin, A
Alcahut-Rodriguez, C
Grande-Martin, A
Sopelana-Garay, D
AF Sanchez-Larsen, Alvaro

Gonzalez-Villar, Esther
Diaz-Maroto, Inmaculada
Layos-Romero, Almudena
Martinez-Martin, Alvaro
Alcahut-Rodriguez, Cristian
Grande-Martin, Alberto
Sopelana-Garay, David
TI Influence of the COVID-19 outbreak in people with epilepsy: Analysis of a Spanish population (EPICOVID registry)
SO EPILEPSY & BEHAVIOR
LA English
DT Article
DE COVID-19; SARS-CoV-2; Coronavirus; Epilepsy; Seizure; Neurology
ID ANTIEPILEPTIC DRUGS; TELEMEDICINE
AB Background: The aim of this study was to have a better understanding of the influence of the coronavirus disease 2019 (COVID-19) pandemic in people with epilepsy (PWE) and to assess whether there have been changes in seizure control during the current COVID-19 outbreak, exploring the possible causes thereof.

Methods: This is an observational, retrospective study based on prospective data collection of 100 successive patients who attended an epilepsy outpatient clinic either face-to-face or telephonically during the months of the COVID-19 outbreak and national state of emergency.

Results: One hundred patients were included, 52% women, mean age 42.4 years. During the COVID-19 period, 27% of the patients presented an increase of >50% of seizure frequency. An increase of stress/anxiety (odds ratios (OR): 5.78; p - 0.008) and a prior higher seizure frequency (OR: 12.4; p = 0.001) were associated with worsening of seizures. Other risk factors were exacerbation of depression, sleep deprivation, less physical activity, and history of epilepsy surgery. Three patients had status epilepticus (SE) and one a duster of seizures. Likewise, 9% of patients improved their seizure control. Reduction in stress/anxiety (OR: 0.05; p = 0.03) and recent adjustment of antiepileptics (OR: 0.07; p = 0.01) acted as protecting factors.

Conclusions: A high proportion of PWE suffered a significant worsening of their seizure control during the months of the COVID-19 pandemic. Emotional distress due to home confinement was the main factor for the change in seizure control. Promoting physical activity and adequate sleep may minimize the potential impact of the pandemic in PWE. Ensuring correct follow-up can prevent decompensation in those PWE at high risk.
(C) 2020 Elsevier Inc. All rights reserved.
C1 [Sanchez-Larsen, Alvaro; Gonzalez-Villar, Esther; Diaz-Maroto, Inmaculada; Layos-Romero, Almudena; Martinez-Martin, Alvaro; Alcahut-Rodriguez, Cristian; Sopelana-Garay, David] Complejo Hosp Univ Albacete, Dept Neurol, Calle Hermanos Falco 37, E-02006 Albacete, Castilla La Man, Spain.

[Grande-Martin, Alberto] Complejo Hosp Univ Albacete, Dept Clin Neurophysiol, Albacete, Castilla La Man, Spain.
RP Sanchez-Larsen, A (通讯作者), Complejo Hosp Univ Albacete, Dept Neurol, Calle Hermanos Falco 37, E-02006 Albacete, Castilla La Man, Spain.
EM aasanchez@sescam.jccm.es
TC 22

Z9 22
PD NOV
PY 2020
VL 112
AR 107396
DI 10.1016/j.yebeh.2020.107396
WC Behavioral Sciences; Clinical Neurology; Psychiatry
ER

PT J
AU Semprino, M
   Fasulo, L
   Fortini, S
   Molina, CIM
   Gonzalez, L
   Ramos, PA
   Martinez, C
   Caraballo, R
AF Semprino, Marcos
   Fasulo, Lorena
   Fortini, Sebastian
   Martorell Molina, Catalina Isabel
   Gonzalez, Lara
   Alejandra Ramos, Paola
   Martinez, Carolina
   Caraballo, Roberto
TI Telemedicine, drug-resistant epilepsy, and ketogenic dietary therapies:
   A patient survey of a pediatric remote-care program during the COVID-19
   pandemic
SO EPILEPSY & BEHAVIOR
LA English
DT Article
DE Drug resistant epilepsy; Ketogenic diet; Telemedicine; Teleneurology;
   WhatsApp
ID MODIFIED ATKINS DIET; WHATSAPP
AB Objective: The purpose of this study was to assess parent satisfaction with the management of ketogenic diet therapies (KDTs) through telemedicine using WhatsApp as the main tool.

   Methods: Parent satisfaction was longitudinally evaluated through questionnaires. The survey was developed with Google Questionnaire forms and sent via WhatsApp. The questionnaire consisted of 13 items concerning the management of KDTs using telemedicine in the context of the coronavirus disease 2019 (COVID-19) pandemic.

   Our population of patients has limited financial resources and low levels of education. Given that many families did not have either computers or WIFI, or any other access to information or communication technology, WhatsApp was chosen as a tool as it was available on the cell phones of all families and the professionals.

   Results: Our survey showed that 96.3% of the parents were satisfied with the

management of KDTs through telemedicine. The main benefits observed were the possibility of continuing treatment during the COVID-19 pandemic and the ease of accessing the professional team from the comfort of their home. Overall. 72.2% of the families would recommend using telemedicine for KDTs in any situation regardless of the pandemic. None of the families reported that they would recommend against treatment by telemedicine. The availability of a social support network (parents WhatsApp group) coordinated by professionals from the KDT team was considered to be useful by most respondents (90%).

Conclusions: Our study suggests that management of children with DRE on KDTs through telemedicine is feasible, well accepted by the families, and probably as safe as conventional medicine. WhatsApp may be an interesting telemedicine tool to start and maintain KDTs. (C) 2020 Elsevier Inc. All rights reserved.
C1 [Semprino, Marcos; Fasulo, Lorena] Clin San Lucas, Dept Neurol, Neuquen, Argentina.
[Fortini, Sebastian; Gonzalez, Lara] Hosp Pediat Nino Jesus, Dept Neurol, San Miguel De Tucuman, Argentina.
[Martorell Molina, Catalina Isabel] Hosp Pediat Nino Jesus, Dept Pediat, San Miguel De Tucuman, Argentina.
[Semprino, Marcos; Fasulo, Lorena] Clin San Lucas, Dept Nutr, Neuquen, Argentina.
[Alejandra Ramos, Paola; Martinez, Carolina] Hosp Pediat Nino Jesus, Dept Nutr, San Miguel De Tucuman, Argentina.
[Caraballo, Roberto] Hosp Pediat Prof Dr Juan P Garrahan, Dept Neurol, Buenos Aires, DF, Argentina.
C3 Hospital de Pediatria Doctor Juan Garrahan
RP Caraballo, R (通讯作者), Combate los Pozos 1881, RA-1245 Buenos Aires, DF, Argentina.
EM rhcaraballo@arnet.com.ar
TC 12
Z9 12
PD NOV
PY 2020
VL 112
AR 107493
DI 10.1016/j.yebeh.2020.107493
WC Behavioral Sciences; Clinical Neurology; Psychiatry
ER

PT J
AU Van Hees, S
Fodjo, JNS
Wijtvliet, V
Van den Bergh, R
Villela, EFD
da Silva, CF
Weckhuysen, S
Colebunders, R
AF Van Hees, Stijn
Fodjo, Joseph Nelson Siewe
Wijtvliet, Veerle

Van den Bergh, Rafael
Villela, Edlaine Faria de Moura
da Silva, Carolina Ferreira
Weckhuysen, Sarah
Colebunders, Robert
TI Access to healthcare and prevalence of anxiety and depression in persons with epilepsy during the COVID-19 pandemic: A multicountry online survey
SO EPILEPSY & BEHAVIOR
LA English
DT Article
DE Epilepsy; Mental health; COVID-19; HADS; PHQ-9
ID QUALITY-OF-LIFE; HOSPITAL ANXIETY; DISORDERS; PEOPLE; SCALE; VALIDATION; INFLUENZA; SEVERITY; IMPACT; COHORT
AB Objective: The objective of this study was to assess access to healthcare and to estimate the prevalence of depression and anxiety among persons with epilepsy (PWE) during the ongoing coronavirus disease 2019 (COVID-19) pandemic.

Methods: We conducted a multicountry online survey among PWE Persons with epilepsy were invited to participate through various social media channels. The Hospital Anxiety and Depression Scale (HADS) and 9-item Patient Health Questionnaire (PHQ-9) scale were used to score anxiety and depression. Logistic regression modeling was used to investigate factors associated with anxiety and depression.

Results: Three hundred ninety-nine PWE were included (mean age: 38.22 +/- 12.09 years), the majority were female (802%) and living in high-income countries (832%). Two hundred three PWE reported symptoms of a cold since January 2020. Nine (25%) of the 36 PWE tested for COVID were positive. A total of 72 PWE (19.6%) reported problems to obtain antiseizure medication (ASM), which in 25% of cases was directly COVID-related. Of the 399 PWE, 201 (50.4%) screened positive for anxiety according to the HADS; 159 (39.8%) and 187 (46.9%) PWE screened positive for depression based on the HADS and PHQ-9 scale, respectively. Female gender and financial problems were associated with both depression and anxiety. A planned follow-up consultation with the treating physician was associated with a lower risk of depression, whereas difficulties to access ASM treatment increased the odds of depression. In 65/137 (47.4%) PWE with a planned follow-up visit with the treating physician, this consultation was canceled.

Conclusions: Innovative approaches are needed to ensure continuity in access to ASM treatment. Healthcare workers should ensure continued follow-up, either through inperson or telehealth appointments, to timely identify symptoms of anxiety and depression and act accordingly. (C) 2020 The Authors. Published by Elsevier Inc.
C1 [Van Hees, Stijn; Fodjo, Joseph Nelson Siewe; Van den Bergh, Rafael; Colebunders, Robert] Univ Antwerp, Global Hlth Inst, Antwerp, Belgium.

[Van Hees, Stijn; Wijtvliet, Veerle] Univ Antwerp, Lab Expt Med & Pediat, Antwerp, Belgium.

[Villela, Edlaine Faria de Moura; da Silva, Carolina Ferreira] Fed Univ Jatai, Sch Med, Hlth Sci Unit, Jatao, Go, Brazil.

[Weckhuysen, Sarah] Univ Hosp Antwerp, Dept Neurol, Edegem, Belgium.

[Weckhuysen, Sarah] Univ Antwerp, VIB Ctr Mol Neurol, Antwerp, Belgium.
C3 University of Antwerp; University of Antwerp; University of Antwerp; Flanders Institute for Biotechnology (VIB); University of Antwerp

RP Colebunders, R (通讯作者), Univ Antwerp, Gouverneur Kinsbergen Ctr, Global Hlth Inst, Doornstr 331, B-2610 Antwerp, Belgium.
TC 28
Z9 28
PD NOV
PY 2020
VL 112
AR 107350
DI 10.1016/j.yebeh.2020.107350
WC Behavioral Sciences; Clinical Neurology; Psychiatry
ER

PT J
AU von Wrede, R
Moskau-Hartmann, S
Baumgartner, T
Helmstaedter, C
Surges, R
AF von Wrede, Randi
Moskau-Hartmann, Susanna
Baumgartner, Tobias
Helmstaedter, Christoph
Surges, Rainer
TI Counseling of people with epilepsy via telemedicine: Experiences at a German tertiary epilepsy center during the COVID-19 pandemic
SO EPILEPSY & BEHAVIOR
LA English
DT Article
DE Epilepsy; Telemedicine; Onsite appointment; COVID-19
ID AMERICAN ACADEMY; WORK GROUP
AB Introduction: Driven by the challenges of alternative healthcare supply during the COVID-19 pandemic, acceptance and appreciation of telemedicine were assessed in a German tertiary epilepsy center.

Methods: Two hundred thirty-nine patients with epilepsy (53% female, 35% seizure-free, 97% on antiseizure medication) answered a structured audit on telemedical counseling as part of individual outpatients' care.

Results: Overall 82% of the participants were satisfied with the telemedical appointment. The telemedical appointment was rated equal to onsite appointments in means of time (91%), comprehensibility (94%), and opportunity to get answers to current questions (92%). It was evaluated as good as onsite appointments regarding comprehension of the disease (88%) and impact on following the physician's advice (82%). The participants considered immediate convenience and shortfall of travel expenses as advantages of telemedicine, whereas lack of personal contact and diagnostics (electroencephalogram [EEG] recordings, blood analysis) were seen as disadvantages. About 73% of the participants would appreciate the opportunity of future telemedical counseling, but the majority (75%) wished to have further appointments onsite.

Conclusions: Overall, people with epilepsy appear to be satisfied with telemedical

counseling. However, patients greatly appreciate the medical services onsite and consider telemedicine as an add-on service rather than a substitute to visits onsite.
(C) 2020 Elsevier Inc. All rights reserved.
C1 [von Wrede, Randi; Moskau-Hartmann, Susanna; Baumgartner, Tobias; Helmstaedter, Christoph; Surges, Rainer] Univ Bonn, Dept Epileptol, Med Ctr, Venusbetg Campus 1, D-53127 Bonn, Germany.
C3 University of Bonn
RP Surges, R (通讯作者), Univ Bonn, Dept Epileptol, Med Ctr, Venusbetg Campus 1, D-53127 Bonn, Germany.
EM rainer.surges@ukbonn.de
TC 29
Z9 29
PD NOV
PY 2020
VL 112
AR 107298
DI 10.1016/j.yebeh.2020.107298
WC Behavioral Sciences; Clinical Neurology; Psychiatry
ER

PT J
AU Willems, LM
Balcik, Y
Noda, AH
Siebenbrodt, K
Leimeister, S
McCoy, J
Kienitz, R
Kiyose, M
Reinecke, R
Schafer, JH
Zollner, JP
Bauer, S
Rosenow, F
Strzelczyk, A
AF Willems, Laurent M.
Balcik, Yunus
Noda, Anna H.
Siebenbrodt, Kai
Leimeister, Sina
McCoy, Jeannie
Kienitz, Ricardo
Kiyose, Makoto
Reinecke, Raphael
Schaefer, Jan-Hendrik
Zoellner, Johann Philipp
Bauer, Sebastian

Rosenow, Felix
Strzelczyk, Adam
TI SARS-CoV-2-related rapid reorganization of an epilepsy outpatient clinic from personal appointments to telemedicine services: A German single-center experience
SO EPILEPSY & BEHAVIOR
LA English
DT Article
DE COVID-19; Corona; Pandemic; Seizure; Anticonvulsant
ID ILAE COMMISSION; OPERATIONAL CLASSIFICATION; POSITION PAPER; COVID-19; GUIDELINES; IMPACT; COSTS
AB Introduction: When the SARS-CoV-2 pandemic reached Europe in 2020, a German governmental order forced clinics to immediately suspend elective care, causing a problem for patients with chronic illnesses such as epilepsy. Here, we report the experience of one clinic that converted its outpatient care from personal appointments to telemedicine services.

Methods: Documentations of telephone contacts and telemedicine consultations at the Epilepsy Center Frankfurt Rhine-Main were recorded in detail between March and May 2020 and analyzed for acceptance, feasibility, and satisfaction of the conversion from personal to telemedicine appointments from both patients' and medical professionals' perspectives.

Results: Telephone contacts for 272 patients (mean age: 38.7 years, range: 17-79 years, 55.5% female) were analyzed. Patient-rated medical needs were either very urgent (6.6%, n = 18), urgent (23.5%, n = 64), less urgent (29.8%, n 81), or nonurgent (39.3%, n = 107). Outpatient service cancelations resulted in a lack of understanding (9.6%, n = 26) or anger and aggression (2.9%, n = 8) in a minority of patients, while 88.6% (n = 241) reacted with understanding, or relief (33%, n= 9). Telemedicine consultations rather than a postponed face-to-face visit were requested by 109 patients (40.1%), and these requests were significantly associated with subjective threat by SARS-CoV-2 (p = 0.004), urgent or very urgent medical needs (p = 0.004), and female gender (p = 0.024). Telemedicine satisfaction by patients and physidans was high. Overall, 9.2% (n = 10) of patients reported general supply problems due to SARS-CoV-2, and 28.4% (n = 31) reported epilepsy-specific problems, most frequently related to prescriptions, or supply problems for antiseizure drugs (ASDs; 22.9%, n 25).

Conclusion: Understanding and acceptance of elective ambulatory visit cancelations and the conversion to telemedicine consultations was high during the coronavirus disease 2019 (COVID-19) lockdown. Patients who engaged in telemedicine consultations were highly satisfied, supporting the feasibility and potential of telemedicine during the COVID-19 pandemic and beyond. (C) 2020 Elsevier Inc. All rights reserved.
C1 [Willems, Laurent M.; Balcik, Yunus; Noda, Anna H.; Siebenbrodt, Kai; Leimeister, Sina; McCoy, Jeannie; Kienitz, Ricardo; Kiyose, Makoto; Reinecke, Raphael; Schaefer, Jan-Hendrik; Zoellner, Johann Philipp; Bauer, Sebastian; Rosenow, Felix; Strzelczyk, Adam] Univ Hosp, Epilepsy Ctr Frankfurt Rhine Main, Frankfurt, Germany.

[Willems, Laurent M.; Balcik, Yunus; Noda, Anna H.; Siebenbrodt, Kai; Leimeister, Sina; McCoy, Jeannie; Kienitz, Ricardo; Kiyose, Makoto; Reinecke, Raphael; Schaefer, Jan-Hendrik; Zoellner, Johann Philipp; Bauer, Sebastian; Rosenow, Felix; Strzelczyk, Adam] Univ Hosp, Dept Neurol, Frankfurt, Germany.

[Willems, Laurent M.; Balcik, Yunus; Noda, Anna H.; Siebenbrodt, Kai; Leimeister, Sina; McCoy, Jeannie; Kienitz, Ricardo; Kiyose, Makoto; Reinecke, Raphael; Schaefer, Jan-Hendrik; Zoellner, Johann Philipp; Bauer, Sebastian; Rosenow, Felix; Strzelczyk, Adam] Goethe Univ Frankfurt, Frankfurt, Germany.
[Willems, Laurent M.; Balcik, Yunus; Noda, Anna H.; Siebenbrodt, Kai; Leimeister, Sina; McCoy, Jeannie; Kienitz, Ricardo; Kiyose, Makoto; Zoellner, Johann Philipp; Bauer, Sebastian; Rosenow, Felix; Strzelczyk, Adam] Goethe Univ Frankfurt, LOEWE Ctr Personalized Translat Epilepsy Res CePT, Frankfurt, Germany.
C3 Goethe University Frankfurt; Goethe University Frankfurt Hospital;
Goethe University Frankfurt; Goethe University Frankfurt Hospital;
Goethe University Frankfurt; Goethe University Frankfurt
RP Strzelczyk, A (通讯作者), Goethe Univ Frankfurt, Epilepsy Ctr Frankfurt Rhine Main, Ctr Neurol & Neurosurg, Schleusenweg 2-16, D-60528 Frankfurt, Germany.
EM strzelczyk@med.uni-frankfurt.de
TC 14
Z9 14
PD NOV
PY 2020
VL 112
AR 107483
DI 10.1016/j.yebeh.2020.107483
WC Behavioral Sciences; Clinical Neurology; Psychiatry
ER

PT J
AU Achar, A
Ghosh, C
AF Achar, Aneesha
Ghosh, Chaitali
TI COVID-19-Associated Neurological Disorders: The Potential Route of CNS Invasion and Blood-Brain Barrier Relevance
SO CELLS
LA English
DT Review
DE CNS; COVID-19; SARS-CoV-2; blood-brain barrier; cerebrovascular; neurological disease
ID ASTROCYTE-ENDOTHELIAL INTERACTIONS; MULTIPLE-SCLEROSIS; COVID-19; RECEPTOR; INFECTION; CYTOKINES; SEIZURE; DISEASE; ENCEPHALITIS; DISRUPTION
AB Severe acute respiratory syndrome coronavirus 2 (SARS-CoV-2) is a novel human coronavirus that has sparked a global pandemic of the coronavirus disease of 2019 (COVID-19). The virus invades human cells through the angiotensin-converting enzyme 2 (ACE2) receptor-driven pathway, primarily targeting the human respiratory tract. However, emerging reports of neurological manifestations demonstrate the neuroinvasive potential of SARS-CoV-2. This review highlights the possible routes by which SARS-CoV-2 may invade the central nervous system (CNS) and provides insight into recent case reports of COVID-19-associated neurological disorders, namely ischaemic stroke,

encephalitis, encephalopathy, epilepsy, neurodegenerative diseases, and inflammatory-mediated neurological disorders. We hypothesize that SARS-CoV-2 neuroinvasion, neuroinflammation, and blood-brain barrier (BBB) dysfunction may be implicated in the development of the observed disorders; however, further research is critical to understand the detailed mechanisms and pathway of infectivity behind CNS pathogenesis.
C1 [Achar, Aneesha; Ghosh, Chaitali] Cleveland Clin, Lerner Res Inst, Dept Biomed Engn, Cerebrovasc Res, Cleveland, OH 44195 USA.
[Ghosh, Chaitali] Case Western Reserve Univ, Cleveland Clin, Lerner Coll Med, Dept Biomed Engn & Mol Med, Cleveland, OH 44195 USA.
C3 Cleveland Clinic Foundation; Case Western Reserve University; Cleveland Clinic Foundation
RP Ghosh, C (通讯作者), Cleveland Clin, Lerner Res Inst, Dept Biomed Engn, Cerebrovasc Res, Cleveland, OH 44195 USA.; Ghosh, C (通讯作者), Case Western Reserve Univ, Cleveland Clin, Lerner Coll Med, Dept Biomed Engn & Mol Med, Cleveland, OH 44195 USA.
EM ava28@case.edu; GHOSHC@ccf.org
TC 38
Z9 40
PD NOV
PY 2020
VL 9
IS 11
AR 2360
DI 10.3390/cells9112360
WC Cell Biology
ER

PT J
AU Sinha, R
Anand, V
Gupta, J
Singh, S
Gulati, S
AF Sinha, Rahul
Anand, Vaishakh
Gupta, Juhi
Singh, Sonali
Gulati, Sheffali
TI Infantile spasms and COVID-19: Challenges and solutions in resource-limited settings
SO EPILEPSY RESEARCH
LA English
DT Letter
DE epilepsy; infantile spasm; COVID-19; telemedicine
ID EPILEPSY
C1 [Sinha, Rahul; Anand, Vaishakh; Gupta, Juhi; Singh, Sonali; Gulati, Sheffali] All India Inst Med Sci AIIMS, Dept Pediat, Child Neurol Div, New Delhi, India.

C3 All India Institute of Medical Sciences (AIIMS) New Delhi
RP Gulati, S (通讯作者), AIIMS, Room 3056,3rd Floor Teaching Block, New Delhi, India.
EM drrahul_2000@yahoo.com; drvyshakhanandmp@gmail.com;
juhiguptadr@gmail.com; sonali2017doc@gmail.com; sheffaligulati@gmail.com
TC 1
Z9 1
PD NOV
PY 2020
VL 167
AR 106441
DI 10.1016/j.eplepsyres.2020.106441
WC Clinical Neurology
ER

PT J
AU Dono, F
Carrarini, C
Russo, M
De Angelis, MV
Anzellotti, F
Onofrj, M
Bonanni, L
AF Dono, Fedele
Carrarini, Claudia
Russo, Mirella
De Angelis, Maria Vittoria
Anzellotti, Francesca
Onofrj, Marco
Bonanni, Laura
TI New-onset refractory status epilepticus (NORSE) in post SARS-CoV-2
autoimmune encephalitis: a case report
SO NEUROLOGICAL SCIENCES
LA English
DT Article
DE COVID-19; Epilepsy; encephalitis; Status epilepticus; Case report
AB The 2019 new coronavirus (SARS-CoV-2) is a novel respiratory virus which has increasingly spread all over the world. Although the predominant clinical presentation is represented by respiratory symptoms, neurological manifestation of SARS-CoV-2 is being increasingly recognized. In the present report, we present a case of post SARS-CoV-2 autoimmune encephalitis associated with a new-onset refractory status epilepticus (NORSE).
C1 [Dono, Fedele; Carrarini, Claudia; Russo, Mirella; Onofrj, Marco; Bonanni, Laura] G DAnnunzio Univ Chieti Pescara, Dept Neurosci Imaging & Clin Sci, Chieti, Italy.
[De Angelis, Maria Vittoria] SS Annunziata Univ Hosp, Neurol Unit, Chieti, Italy.
[Anzellotti, Francesca] SS Annunziata Univ Hosp, Epilepsy Ctr, Dept Neurol, Neurol Unit, Chieti, Italy.
C3 G d'Annunzio University of Chieti-Pescara; G d'Annunzio University of

Chieti-Pescara; G d'Annunzio University of Chieti-Pescara
RP Bonanni, L (通讯作者), G DAnnunzio Univ Chieti Pescara, Dept Neurosci Imaging & Clin Sci, Chieti, Italy.
EM fedele.dono@alumni.unich.it; claudia.carrarini@alumni.unich.it;
mirella.russo@alumni.unich.it; mavidea@yahoo.com;
f.anzellotti@libero.it; onofrj@unich.it; l.bonanni@unich.it
TC 14
Z9 14
PD JAN
PY 2021
VL 42
IS 1
BP 35
EP 38
DI 10.1007/s10072-020-04846-z
EA NOV 2020
WC Clinical Neurology; Neurosciences
ER

PT J
AU Nakamoto, M
Carrazana, E
Viereck, J
Liow, K
AF Nakamoto, Max
Carrazana, Enrique
Viereck, Jason
Liow, Kore
TI Epilepsy in the time of COVID-19
SO ACTA NEUROLOGICA SCANDINAVICA
LA English
DT Letter
DE epilepsy; COVID-19; telemedicine; depression
C1 [Nakamoto, Max; Carrazana, Enrique; Liow, Kore] Univ Hawaii, John A Burns Sch Med, Honolulu, HI 96822 USA.
[Carrazana, Enrique; Viereck, Jason; Liow, Kore] Hawaii Pacific Neurosci, Comprehens Epilepsy Ctr, Honolulu, HI USA.
[Carrazana, Enrique; Viereck, Jason; Liow, Kore] Hawaii Pacific Neurosci, Video EEG Epilepsy Monitoring Unit, Honolulu, HI USA.
C3 University of Hawaii System
RP Carrazana, E (通讯作者), Univ Hawaii, John Burns Sch Med, Hawaii Pacific Neurosci, Honolulu, HI 96822 USA.
EM ecarrazana@aol.com
TC 4
Z9 4
PD MAR
PY 2021

VL 143
IS 3
BP 333
EP 335
DI 10.1111/ane.13360
EA NOV 2020
WC Clinical Neurology
ER

PT J
AU Gali, K
   Joshi, S
   Hueneke, S
   Katzenbach, A
   Radecki, L
   Calabrese, T
   Fletcher, L
   Trandafir, C
   Wilson, C
   Goyal, M
   Wusthoff, CJ
   Le Pichon, JB
   Corvalan, R
   Golson, A
   Hardy, J
   Smith, M
   Cook, E
   Bonkowsky, JL
AF Gali, Kari
   Joshi, Sucheta
   Hueneke, Sarah
   Katzenbach, Alexis
   Radecki, Linda
   Calabrese, Trisha
   Fletcher, Linda
   Trandafir, Cristina
   Wilson, Carey
   Goyal, Monisha
   Wusthoff, Courtney J.
   Le Pichon, Jean-Baptiste
   Corvalan, Rhonda
   Golson, April
   Hardy, Jessica
   Smith, Michael
   Cook, Elizabeth
   Bonkowsky, Joshua L.
TI Barriers, access and management of paediatric epilepsy with telehealth

SO JOURNAL OF TELEMEDICINE AND TELECARE
LA English
DT Article
DE Paediatric epilepsy; seizure; telemedicine; team-based care; subspecialty paediatrics; medical home; health-care transition; underserved; rural; quality improvement; learning collaborative
AB Access to paediatric neurology care is complex, resulting in significant wait times and negative patient outcomes. The goal of the American Academy of Pediatrics National Coordinating Center for Epilepsy's project, Access Improvement and Management of Epilepsy with Telehealth (AIM-ET), was to identify access and management challenges in the deployment of telehealth technology. AIM-ET organised four paediatric neurology teams to partner with primary-care providers (PCP) and their multidisciplinary teams. Telehealth visits were conducted for paediatric epilepsy patients. A post-visit survey assessed access and satisfaction with the telehealth visit compared to an in-person visit. Pre/post surveys completed by PCPs and neurologists captured telehealth visit feasibility, functionality and provider satisfaction. A provider focus group assessed facilitators and barriers to telehealth. Sixty-one unique patients completed 75 telehealth visits. Paired t-test analysis demonstrated that telehealth enhanced access to epilepsy care. It reduced self-reported out-of-pocket costs ($p<0.001$), missed school hours ($p<0.001$) and missed work hours ($p<0.001$), with 94% equal parent/caregiver satisfaction. Focus groups indicated developing and maintaining partnerships, institutional infrastructure and education as facilitators and barriers to telehealth. Telehealth shortened travelling distance, reduced expenses and time missed from school and work. Further, it provides significant opportunity in an era when coronavirus disease 2019 limits in-person clinics.
C1 [Gali, Kari] Cleveland Clin, 9500 Euclid Ave, Cleveland, OH 44195 USA.
[Joshi, Sucheta; Fletcher, Linda] Pediat Neurol Michigan Med, Ann Arbor, MI USA.
[Hueneke, Sarah; Katzenbach, Alexis; Calabrese, Trisha] Amer Acad Pediat, Itasca, IL USA.
[Radecki, Linda] RadeckiRes LLC, San Diego, CA USA.
[Trandafir, Cristina; Wilson, Carey; Bonkowsky, Joshua L.] Univ Utah, Dept Pediat, Sch Med, Div Pediat Neurol, Salt Lake City, UT 84112 USA.
[Trandafir, Cristina; Wilson, Carey; Bonkowsky, Joshua L.] Primary Childrens Med Ctr, Salt Lake City, UT USA.
[Goyal, Monisha; Corvalan, Rhonda] Univ Alabama Birmingham, Birmingham, AL USA.
[Wusthoff, Courtney J.] Stanford Univ, Stanford, CA 94305 USA.
[Le Pichon, Jean-Baptiste; Cook, Elizabeth] Childrens Mercy Kansas City, Kansas City, MO USA.
[Golson, April; Hardy, Jessica; Smith, Michael] Alabama Dept Publ Hlth, Montgomery, AL 36102 USA.
C3 Cleveland Clinic Foundation; American Academy of Pediatrics; Utah System of Higher Education; University of Utah; University of Alabama System; University of Alabama Birmingham; Stanford University; Children's Mercy Hospital
RP Gali, K (通讯作者), Cleveland Clin, 9500 Euclid Ave, Cleveland, OH 44195 USA.
EM kxg19@case.edu
TC 3

Z9 3
PD APR
PY 2022
VL 28
IS 3
BP 213
EP 223
AR 1357633X20969531
DI 10.1177/1357633X20969531
EA NOV 2020
WC Health Care Sciences & Services
ER

PT J
AU Fujimoto, A
Sato, K
Enoki, H
AF Fujimoto, Ayataka
Sato, Keishiro
Enoki, Hideo
TI Change in Patient Flow in the Epilepsy Care Network Due to Novel Coronavirus Infection: An Opportunity to Strengthen Local Interdisciplinary Epilepsy Care With General Physicians
SO FRONTIERS IN NEUROLOGY
LA English
DT Article
DE patient flow; local interdisciplinary epilepsy network; economic crisis; COVID-19; SARS-CoV-2
ID CLASSIFICATION; SYSTEM; IMPACT
AB Introduction: Novel coronavirus disease 2019 (COVID-19) infection caused by severe acute respiratory syndrome coronavirus 2 (SARS-CoV-2) is spreading worldwide. We hypothesized that patient flow in epilepsy care would change as a result of the COVID-19 pandemic. The purpose of this study was to compare the number of patients who visited our epilepsy center before and during the first peak of the pandemic.

Methods: We recorded the number of patients with epilepsy referred from general physicians (GPs) to our hospital (GP-H group), the number of patients who visited our hospital on a regular basis (R group), and the number of patients referred from our hospital to GPs (H-GP group) between July 2019 and June 2020.

Results: A total of 1,839 epilepsy patients made 4,197 visits to our hospital: 979 males and 860 females (age range, 0-94 years; mean age, 37.6 years; median age, 34 years). There were 433 patients in the GP-H group (247 before the pandemic, 186 during the first peak of the pandemic; $p = 0.008$). In the R group, 1,406 patients made 3,764 visits (1,992 visits before the pandemic, 1,772 during the first peak of the pandemic). In the H-GP group, 135 patients were referred to GPs (47 patients before the pandemic, 88 patients during the first peak of the pandemic; $p = 0.023$).

Conclusion: Patient flow in the epilepsy care network changed as a result of the COVID-19 pandemic. These changes might present an opportunity to strengthen local

interdisciplinary epilepsy care.
C1 [Fujimoto, Ayataka; Sato, Keishiro; Enoki, Hideo] Seirei Hamamatsu Gen Hosp, Comprehens Epilepsy Ctr, Hamamatsu, Shizuoka, Japan.
[Fujimoto, Ayataka] Seirei Hamamatsu Gen Hosp, Dept Neurosurg, Comprehens Epilepsy Ctr, Hamamatsu, Shizuoka, Japan.
[Sato, Keishiro] Seirei Hamamatsu Gen Hosp, Dept Neurol, Comprehens Epilepsy Ctr, Hamamatsu, Shizuoka, Japan.
[Enoki, Hideo] Seirei Hamamatsu Gen Hosp, Dept Pediat Neurol, Comprehens Epilepsy Ctr, Hamamatsu, Shizuoka, Japan.
RP Fujimoto, A (通讯作者)，Seirei Hamamatsu Gen Hosp, Comprehens Epilepsy Ctr, Hamamatsu, Shizuoka, Japan.; Fujimoto, A (通讯作者)，Seirei Hamamatsu Gen Hosp, Dept Neurosurg, Comprehens Epilepsy Ctr, Hamamatsu, Shizuoka, Japan.
EM afujimotoscienceacademy@gmail.com
TC 3
Z9 3
PD NOV 16
PY 2020
VL 11
AR 591423
DI 10.3389/fneur.2020.591423
WC Clinical Neurology; Neurosciences
ER

PT J
AU Blanco, EC
Manzanares, I
Centeno, M
Khawaja, M
Betran, O
Donaire, A
Carreno, M
AF Blanco, Estefania Conde
Manzanares, Isabel
Centeno, Maria
Khawaja, Mariam
Betran, Olga
Donaire, Antonio
Carreno, Mar
TI Epilepsy and lockdown: A survey of patients normally attending a Spanish centre
SO ACTA NEUROLOGICA SCANDINAVICA
LA English
DT Editorial Material
DE COVID-19; epilepsy; neuroepidemiology
ID PEOPLE
AB Background: Lockdown due to the SARS-CoV-2 pandemic became a challenge to maintain care for patients with epilepsy; we aimed to find out how the pandemic affected them.

Methods: We sent an online 22-item questionnaire to patients from our outpatient clinic, a reference centre in Spain for drug-resistant epilepsy, inquiring about the effects of lockdown, from March to May 2020.
Results: We sent the survey to 627 patients; 312 (58% women) sent a complete response and were included. Of all respondents, 57% took >2 antiseizure medications. One-third of respondents (29%) declared an associated cognitive or motor disability. A minority had confirmed infection with SARS-CoV-2 (1.92%). Seizure frequency remained like usual in 56% of patients, while 31.2% reported an increase. Less than 10% needed emergent assistance. Almost half reported anxiety or depression, and 25% increased behavioural disorders. Mood (F: 5.40; p: 0.002) and sleep disorders (F = 2.67; p: 0.05) were associated with increase in seizure frequency. Patients were able to contact their physicians when needed and were open to a future telematic approach to follow-up visits.
Conclusions: ySeizure frequency and severity remained unchanged in most patients during the lockdown. Mood and sleep disorders were common and associated with seizure worsening. Patients were open to telematic care in the future.
C1 [Blanco, Estefania Conde; Manzanares, Isabel; Centeno, Maria; Khawaja, Mariam; Betran, Olga; Donaire, Antonio; Carreno, Mar] Hosp Clin Barcelona, Inst Invest Biomed August Pi & Sunyer IDIBAPS, EpiCARE Network, Barcelona, Spain.
C3 League of European Research Universities - LERU; University of
Barcelona; Hospital Clinic de Barcelona; IDIBAPS
RP Carreno, M (通讯作者), Hosp Clin Barcelona, Epilepsy Unit, Dept Neurol, Carrer Villarroel 170, Barcelona 08036, Spain.
EM mcarreno@clinic.cat
TC 9
Z9 9
PD FEB
PY 2021
VL 143
IS 2
BP 206
EP 209
DI 10.1111/ane.13354
EA NOV 2020
WC Clinical Neurology
ER

PT J
AU Asadi-Pooya, AA
Simani, L
Shahisavandi, M
Barzegar, Z
AF Asadi-Pooya, Ali A.
Simani, Leila
Shahisavandi, Mina
Barzegar, Zohreh
TI COVID-19, de novo seizures, and epilepsy: a systematic review
SO NEUROLOGICAL SCIENCES

LA English
DT Review
DE Coronavirus; COVID-19; EEG; Epilepsy; Seizure
ID REFRACTORY STATUS EPILEPTICUS; EEG; ENCEPHALOPATHY; INFECTION; PATIENT; PEOPLE; TELEMEDICINE; MARKERS
AB Objective We discuss the evidence on the occurrence of de novo seizures in patients with COVID-19, the consequences of this catastrophic disease in people with epilepsy (PWE), and the electroencephalographic (EEG) findings in patients with COVID-19. Methods This systematic review was prepared according to the recommendations of the Preferred Reporting Items for Systematic Reviews and Meta-Analyses statement. MEDLINE, Scopus, and Embase from inception to August 15, 2020 were systematically searched. These key words were used: "COVID" AND "seizure" OR "epilepsy" OR "EEG" OR "status epilepticus" OR "electroencephalography". Results We could identify 62 related manuscripts. Many studies were case reports or case series of patients with COVID-19 and seizures. PWE showed more psychological distress than healthy controls. Many cases with new-onset focal seizures, serial seizures, and status epilepticus have been reported in the literature. EEG studies have been significantly ignored and underused globally. Conclusion Many PWE perceived significant disruption in the quality of care to them, and some people reported increase in their seizure frequency since the onset of the pandemic. Telemedicine is a helpful technology that may improve access to the needed care for PWE in these difficult times. De novo seizures may occur in people with COVID-19 and they may happen in a variety of forms. In addition to prolonged EEG monitoring, performing a through metabolic investigation, electrocardiogram, brain imaging, and a careful review of all medications are necessary steps. The susceptibility of PWE to contracting COVID-19 should be investigated further.
C1 [Asadi-Pooya, Ali A.; Shahisavandi, Mina; Barzegar, Zohreh] Shiraz Univ Med Sci, Epilepsy Res Ctr, Shiraz, Iran.
[Asadi-Pooya, Ali A.] Thomas Jefferson Univ, Dept Neurol, Jefferson Comprehens Epilepsy Ctr, Philadelphia, PA 19107 USA.
[Simani, Leila] Shahid Beheshti Univ Med Sci, Loghman Hakim Hosp, Skull Base Res Ctr, Tehran, Iran.
C3 Shiraz University of Medical Science; Jefferson University; Shahid Beheshti University Medical Sciences
RP Asadi-Pooya, AA (通讯作者)，Shiraz Univ Med Sci, Epilepsy Res Ctr, Shiraz, Iran.; Asadi-Pooya, AA (通讯作者)，Thomas Jefferson Univ, Dept Neurol, Jefferson Comprehens Epilepsy Ctr, Philadelphia, PA 19107 USA.
EM aliasadipooya@yahoo.com; l.simani62@gmail.com; shahisavandimina@gmail.com; zohrehbarzegar1375@gmail.com
TC 23
Z9 23
PD FEB
PY 2021
VL 42
IS 2
BP 415
EP 431
DI 10.1007/s10072-020-04932-2

EA NOV 2020
WC Clinical Neurology; Neurosciences
ER

PT J
AU Besnard, S
   Nardin, C
   Lyon, E
   Debroucker, T
   Arjmand, R
   Moretti, R
   Pochat, H
AF Besnard, Stephane
   Nardin, Clotilde
   Lyon, Elsa
   Debroucker, Thomas
   Arjmand, Roxana
   Moretti, Raffaella
   Pochat, Herve
TI Electroencephalographic Abnormalites in SARS-CoV-2 Patients
SO FRONTIERS IN NEUROLOGY
LA English
DT Article
DE confusion; epileptic seizure; virus; encephalopathy; electroencephalography
ID COVID-19; EEG
AB Viral infection with SARS-CoV-2 has a neurological tropism that may induce an encephalopathy. In this context, electroencephalographic exploration (EEG) is indicated as a diagnostic argument correlated with lumbar puncture, biology, and imaging. We performed a retrospective analysis of 42 patients explored by EEG and infected by COVID-19, according to the EEG abnormalities and clinical signs that motivated the examination. Confusion and epileptic seizures were the most common clinical indications, with 64% of the patients displaying these symptoms. The EEG was altered in 85% of the cases of confusion, in 57% of the cases of epileptic symptoms (general or focal seizure or prolonged loss of contact) and 20% of the cases of malaise or brief loss of consciousness. Nine EEG (21%) were in favor of an encephalopathy, two had de novo alterations in persistent consciousness and two had alterations in general states of confusion; one was very agitated and without history of epilepsy and combined eyelids clonia while a second one exhibited unconsciousness with left hemicorpus clonus. Two were being investigated for delayed awakening without sedation for more than 24 h. All of these patients were diagnosed COVID-19, some of them with associated mild to severe respiratory disorders. This work shows the interest of the EEG in exploring COVID-19 patients suffering from neurological or general symptoms looking for cerebral alteration.
C1 [Besnard, Stephane] CHU Caen, Explorat Fonct, Univ Hosp Caen Normandie, Caen, France.
   [Nardin, Clotilde; Debroucker, Thomas] Ctr Hosp Delafontaine, St Denis, France.
   [Lyon, Elsa] ELYOPE SAS, Paris, France.
   [Arjmand, Roxana] Ctr Hosp Sens, Yonne, Sens, France.

[Moretti, Raffaella] CHU Armand Trousseau, AP HP, Explorat Fonct Pediat, Paris, France.
[Pochat, Herve] SIGMA EEG SARL, Paris, France.
C3 CHU de Caen NORMANDIE; Universite de Caen Normandie; Assistance Publique Hopitaux Paris (APHP); Hopital Universitaire Armand-Trousseau - APHP; League of European Research Universities - LERU; UDICE-French Research Universities; Sorbonne Universite
RP Besnard, S (通讯作者), CHU Caen, Explorat Fonct, Univ Hosp Caen Normandie, Caen, France.
EM stephane.besnard@unicaen.fr
TC 3
Z9 3
PD NOV 26
PY 2020
VL 11
AR 582794
DI 10.3389/fneur.2020.582794
WC Clinical Neurology; Neurosciences
ER

PT J
AU Rosengard, JL
Donato, J
Ferastraoaru, V
Zhao, D
Molinero, I
Boro, A
Gursky, J
Correa, DJ
Galanopoulou, AS
Hung, CE
Legatt, AD
Patel, P
Rubens, E
Moshe, SL
Haut, S
AF Rosengard, Jillian L.
Donato, Jad
Ferastraoaru, Victor
Zhao, Dan
Molinero, Isaac
Boro, Alexis
Gursky, Jonathan
Correa, Daniel Jose
Galanopoulou, Aristea S.
Hung, Christine
Legatt, Alan D.

Patel, Puja
Rubens, Elayna
Moshe, Solomon L.
Haut, Sheryl
TI Seizure control, stress, and access to care during the COVID-19 pandemic in New York City: The patient perspective
SO EPILEPSIA
LA English
DT Article
DE care; COVID-19; epilepsy; pandemic; stress; teleneurology
ID EPILEPSY
AB Objective Our epilepsy population recently experienced the acute effects of the COVID-19 pandemic in New York City. Herein, we aimed to determine patient-perceived seizure control during the surge, specific variables associated with worsened seizures, the prevalence of specific barriers to care, and patient-perceived efficacy of epilepsy care delivered via telephone and live video visits during the pandemic.

Methods We performed a cross-sectional questionnaire study of adult epilepsy patients who had a scheduled appointment at a single urban Comprehensive Epilepsy Center (Montefiore Medical Center) between March 1, 2020 and May 31, 2020 during the peak of the COVID-19 pandemic in the Bronx. Subjects able to answer the questionnaire themselves in English or Spanish were eligible to complete a one-time survey via telephone or secure online platform (REDCap).

Results Of 1212 subjects screened, 675 were eligible, and 177 adequately completed the questionnaire. During the COVID-19 pandemic, 75.1% of patients reported no change in seizure control, whereas 17.5% reported that their seizure control had worsened, and 7.3% reported improvement. Subjects who reported worsened seizure control had more frequent seizures at baseline, were more likely to identify stress and headaches/migraines as their typical seizure precipitants, and were significantly more likely to report increased stress related to the pandemic. Subjects with confirmed or suspected COVID-19 did not report worsened seizure control. Nearly 17% of subjects reported poorer epilepsy care, and 9.6% had difficulty obtaining their antiseizure medications; these subjects were significantly more likely to report worse seizure control.

Significance Of the nearly 20% of subjects who reported worsened seizure control during the COVID-19 pandemic, stress and barriers to care appear to have posed the greatest challenge. This unprecedented pandemic exacerbated existing and created new barriers to epilepsy care, which must be addressed.
C1 [Rosengard, Jillian L.; Donato, Jad; Ferastraoaru, Victor; Molinero, Isaac; Boro, Alexis; Gursky, Jonathan; Correa, Daniel Jose; Galanopoulou, Aristea S.; Hung, Christine; Legatt, Alan D.; Patel, Puja; Rubens, Elayna; Moshe, Solomon L.; Haut, Sheryl] Montefiore Epilepsy Ctr, Saul R Korey Dept Neurol & Comprehens Einstein, Bronx, NY USA.

[Zhao, Dan] Albert Einstein Coll Med, Bronx, NY 10467 USA.

[Molinero, Isaac; Patel, Puja; Moshe, Solomon L.] Isabelle Rapin Div Child Neurol, Bronx, NY USA.

[Galanopoulou, Aristea S.; Moshe, Solomon L.] Dominick P Purpura Dept Neurosci, Bronx, NY USA.

[Moshe, Solomon L.] Montefiore Med Ctr, Albert Einstein Coll Med, Dept Pediat, Bronx,

NY 10467 USA.
C3 Yeshiva University; Albert Einstein College of Medicine; Montefiore
   Medical Center; Yeshiva University; Albert Einstein College of Medicine
RP Rosengard, JL (通讯作者), Montefiore Med Ctr, Dept Neurol, NW 002,111 East 210th St, Bronx, NY 10467 USA.
EM jrosenga@montefiore.org
TC 20
Z9 21
PD JAN
PY 2021
VL 62
IS 1
BP 41
EP 50
DI 10.1111/epi.16779
EA NOV 2020
WC Clinical Neurology
ER

PT J
AU Sham, L
   Ciccone, O
   Patel, AA
AF Sham, Lauren
   Ciccone, Ornella
   Patel, Archana A.
TI The COVID-19 pandemic and Community Health Workers: An opportunity to
   maintain delivery of care and education for families of children with
   epilepsy in Zambia
SO JOURNAL OF GLOBAL HEALTH
LA English
DT Editorial Material
DE care; COVID-19; epilepsy; pandemic; stress
C1 [Sham, Lauren; Patel, Archana A.] Harvard Med Sch, Boston Childrens Hosp, Div Epilepsy & Clin Neurophysiol, Dept Neurol, Boston, MA 02115 USA.
   [Ciccone, Ornella] Lusaka Childrens Hosp, Univ Teaching Hosp, Lusaka, Zambia.
   [Patel, Archana A.] Univ Zambia, Sch Med, Dept Paediat & Child Hlth, Lusaka, Zambia.
C3 Harvard University; Boston Children's Hospital; Harvard Medical School;
   University of Zambia; University of Zambia
RP Patel, AA (通讯作者), Boston Childrens Hosp, Dept Neurol, Div Epilepsy, 300 Longwood Ave,Fegan 9, Boston, MA 02115 USA.
EM archana.patel@childrens.harvard.edu
TC 0
Z9 1
PD DEC
PY 2020
VL 10

IS 2
AR 020329
DI 10.7189/jogh.10.020329
WC Public, Environmental & Occupational Health
ER

PT J
AU Davico, C
Marcotulli, D
Lux, C
Calderoni, D
Terrinoni, A
Di Santo, F
Ricci, F
Vittorini, R
Amianto, F
Urbino, A
Ferrara, M
Vitiello, B
AF Davico, Chiara
Marcotulli, Daniele
Lux, Caterina
Calderoni, Dario
Terrinoni, Arianna
Di Santo, Federica
Ricci, Federica
Vittorini, Roberta
Amianto, Federico
Urbino, Antonio
Ferrara, Mauro
Vitiello, Benedetto
TI Where have the children with epilepsy gone? An observational study of seizure-related accesses to emergency department at the time of COVID-19
SO SEIZURE-EUROPEAN JOURNAL OF EPILEPSY
LA English
DT Article
DE Covid-19; Children; Seizure; Epilepsy; Emergency; neurology
ID SEASONAL-VARIATION
AB Purpose: The COVID-19 pandemic and related lockdown measures drastically changed health care and emergency services utilization. This study evaluated trends in emergency department (ED) access for seizure-related reasons in the first 8 weeks of lockdown in Italy.

Methods: All ED accesses of children (<14 years of age) at two university hospitals, in Turin and Rome, Italy, between January 6, 2020 and April 21, 2020, were examined and compared with the corresponding periods of 2019.

Results: During the COVID-19 lockdown period (February 23-April 21, 2020), there was a 72 % decrease in all pediatric ED accesses over the corresponding 2019 period

(n = 3,395 vs n = 12,128), with a 38 % decrease in seizure-related accesses (n = 41 vs n = 66). The observed decrease of seizure-related ED accesses was not accompanied by significant changes in age, sex, type of seizure, or hospitalization rate after the ED visit.
Conclusion: The COVID-19 lockdown was accompanied by a sudden decrease in seizure-related hospital emergency visits. School closure, social distancing, reduced risk of infection, and increased parental supervision are some of the factors that might have contributed to the finding.
C1 [Davico, Chiara; Marcotulli, Daniele; Lux, Caterina; Ricci, Federica; Vittorini, Roberta; Vitiello, Benedetto] Univ Turin, Dept Publ Hlth & Pediat Sci, Sect Child & Adolescent Neuropsychiat, Piazza Polonia 94, I-10100 Turin, Italy.
[Calderoni, Dario; Terrinoni, Arianna; Di Santo, Federica; Ferrara, Mauro] Univ Roma La Sapienza, Sect Child & Adolescent Neuropsychiat, Rome, Italy.
[Amianto, Federico] Univ Turin, Dept Neurosci, Turin, Italy.
[Urbino, Antonio] Regina Margherita Hosp, Dept Child Care, Emergency Pediat, Turin, Italy.
C3 University of Turin; Sapienza University Rome; University of Turin;
A.O.U. Citta della Salute e della Scienza di Torino; Ospedale Infantile
Regina Margherita
RP Davico, C (通讯作者), Univ Turin, Dept Publ Hlth & Pediat Sci, Sect Child & Adolescent Neuropsychiat, Piazza Polonia 94, I-10100 Turin, Italy.
EM chiara.davico@unito.it
TC 8
Z9 8
PD DEC
PY 2020
VL 83
BP 38
EP 40
DI 10.1016/j.seizure.2020.09.025
WC Clinical Neurology; Neurosciences
ER

PT J
AU Tailby, C
Collins, AJ
Vaughan, DN
Abbott, DF
O'Shea, M
Helmstaedter, C
Jackson, GD
AF Tailby, Chris
Collins, Alana J.
Vaughan, David N.
Abbott, David F.
O'Shea, Marie
Helmstaedter, Christoph

Jackson, Graeme D.
TI Teleneuropsychology in the time of COVID-19: The experience of The Australian Epilepsy Project
SO SEIZURE-EUROPEAN JOURNAL OF EPILEPSY
LA English
DT Article
DE Cognitive assessment; Epilepsy; surgery; Telemedicine; Telepsychotherapy; Coronavirus; SARS-CoV-2
ID NEUROPSYCHOLOGICAL ASSESSMENT; EXECUTIVE FUNCTIONS; VERBAL FLUENCY; LOBE EPILEPSY; OLDER-ADULTS; MEMORY; ASSESSMENTS; TELEMEDICINE; FEASIBILITY; SURGERY
AB Purpose: Traditional neuropsychological testing carries elevated COVID-19 risk for both examinee and examiner. Here we describe how the pilot study of the Australian Epilepsy Project (AEP) has transitioned to teleneuropsychology (teleNP), enabling continued safe operations during the pandemic.

Methods: The AEP includes adults (age 18-60) with a first unprovoked seizure, new diagnosis of epilepsy or drug resistant focal epilepsy. Shortly after launching the study, COVID-related restrictions necessitated adaptation to teleNP, including delivery of verbal tasks via videoconference; visual stimulus delivery via document camera; use of web-hosted, computerised assessment; substitution of oral versions for written tests; online delivery of questionnaires; and discontinuation of telehealth incompatible tasks.

Results: To date, we have completed 24 teleNP assessments: 18 remotely (participant in own home) and six onsite (participant using equipment at research facility). Five face-to-face assessments were conducted prior to the transition to teleNP. Eight of 408 tests administered via teleNP (1.9 %) have been invalidated, for a variety of reasons (technical, procedural, environmental). Data confirm typical patterns of epilepsy-related deficits ($p < .05$) affecting processing speed, executive function, language and memory. Questionnaire responses indicate elevated rates of patients at high risk of mood (34 %) and anxiety disorder (38 %).

Conclusion: Research teleNP assessments reveal a typical pattern of impairments in epilepsy. A range of issues must be considered when introducing teleNP, such as technical and administrative set up, test selection and delivery, and cohort suitability. TeleNP enables large-scale neuropsychological research during periods of social distancing (and beyond), and offers an opportunity to expand the reach and breadth of neumpsychological services.
C1 [Tailby, Chris; Collins, Alana J.; Vaughan, David N.; Abbott, David F.; Jackson, Graeme D.] Florey Inst Neurosci & Mental Hlth, Austin Campus, 245 Burgundy St, Heidelberg, Vic 3084, Australia.

[Tailby, Chris; O'Shea, Marie] Austin Hlth, Dept Clin Neuropsychol, Heidelberg, Vic, Australia.

[Vaughan, David N.; Jackson, Graeme D.] Austin Hlth, Dept Neurol, Heidelberg, Vic, Australia.

[Abbott, David F.; Jackson, Graeme D.] Univ Melbourne, Fac Med Dent & Hlth Sci, Florey Dept Neurosci & Mental Hlth, Parkville, Vic, Australia.

[O'Shea, Marie] Univ Melbourne, Sch Psychol Sci, Parkville, Vic, Australia.

[Helmstaedter, Christoph] Univ Hosp Bonn UKB, Dept Epileptol, Bonn, Germany.

C3 Florey Institute of Neuroscience & Mental Health; Austin Research
Institute; Florey Institute of Neuroscience & Mental Health; Austin
Research Institute; Florey Institute of Neuroscience & Mental Health;
University of Melbourne; University of Melbourne; University of Bonn
RP Tailby, C (通讯作者), Florey Inst Neurosci & Mental Hlth, Austin Campus, 245 Burgundy St, Heidelberg, Vic 3084, Australia.
EM chris.tailby@florey.edu.au
TC 9
Z9 9
PD DEC
PY 2020
VL 83
BP 89
EP 97
DI 10.1016/j.seizure.2020.10.005
WC Clinical Neurology; Neurosciences
ER

PT J
AU Antony, AR
Haneef, Z
AF Antony, Arun Raj
Haneef, Zulfi
TI Systematic review of EEG findings in 617 patients diagnosed with
COVID-19
SO SEIZURE-EUROPEAN JOURNAL OF EPILEPSY
LA English
DT Review
DE COVID-19; EEG; Seizure; encephalitis; Encephalopathy; SARS CoV-2
ID INFECTION; DISEASE
AB Objective: We performed a systematic review of the literature to synthesize the data on EEG findings in COVID-19. Frontal EEG patterns are reported to be a characteristic finding in COVID-19 encephalopathy. Although several reports of EEG abnormalities are available, there is lack of clarity about typical findings.

Methods: Research databases were queried with the terms "COVID" OR "coronavirus" OR "SARS" AND "EEG". Available data was analyzed from 617 patients with EEG findings reported in 84 studies.

Results: The median age was 61.3 years (IQR 45-69, 33.3 % female). Common EEG indications were altered mental status (61.7 %), seizure-like events (31.2 %), and cardiac arrest (3.5 %). Abnormal EEG findings (n = 543, 88.0 %) were sub-classified into three groups: (1) Background abnormalities: diffuse slowing (n = 423, 68.6 %), focal slowing (n = 105, 17.0 %), and absent posterior dominant rhythm (n = 63, 10.2 %). (2) Periodic and rhythmic EEG patterns: generalized periodic discharges (n = 35, 5.7 %), lateralized/multifocal periodic discharges (n = 24, 3.9 %), generalized rhythmic activity (n = 32, 5.2 %). (3) Epileptiform changes: focal (n = 35, 5.7 %), generalized (n = 27, 4.4 %), seizures/status epilepticus (n = 34, 5.5 %). Frontal EEG patterns comprised of approximately a third of all findings. In studies that utilized continuous

EEG, 96.8 % (n = 243) of the 251 patients were reported to have abnormalities compared to 85.0 % (n = 311) patients who did not undergo continuous EEG monitoring (chi(2) = 22.8, p=<0.001).

Significance: EEG abnormalities are common in COVID-19 related encephalopathy and correlates with disease severity, preexisting neurological conditions including epilepsy and prolonged EEG monitoring. Frontal findings are frequent and have been proposed as a biomarker for COVID-19 encephalopathy.

C1 [Antony, Arun Raj] 9100 Babcock Blvd,Profess Bldg T, Pittsburgh, PA 15237 USA.
[Haneef, Zulfi] Baylor Coll Med, Dept Neurol, Houston, TX 77030 USA.
[Haneef, Zulfi] VA Houston Med Ctr, Neurol Care Line, Houston, TX 77030 USA.
C3 Baylor College of Medicine
RP Haneef, Z (通讯作者), Baylor Coll Med, Kellaway Sect Neumphysiol, Michael E DeBakey VA Med Ctr, Houston, TX 77030 USA.
EM zulfi.haneef@bcm.edu
TC 34
Z9 36
PD DEC
PY 2020
VL 83
BP 234
EP 241
DI 10.1016/j.seizure.2020.10.014
WC Clinical Neurology; Neurosciences
ER

PT J
AU Anand, SK
Macki, M
Culver, LG
Wasade, VS
Hendren, S
Schwalb, JM
AF Anand, Sharath Kumar
Macki, Mohamed
Culver, Lauren G.
Wasade, Vibhangini S.
Hendren, Samantha
Schwalb, Jason M.
TI Patient navigation in epilepsy care
SO EPILEPSY & BEHAVIOR
LA English
DT Editorial Material
DE COVID-19; Nurse navigation; Socioeconomic disparities
ID SUDDEN UNEXPECTED DEATH; SURGERY; DISPARITIES; MORTALITY; CANCER; RATES; NEED
AB The concept of patient navigation was first introduced in 1989 by the American Cancer Society and was first implemented in 1990 by Dr. Harold Freeman in Harlem, NY. The role

of a patient navigator (PN) is to coordinate care between the care team, the patient, and their family while also providing social support. In the last 30 years, patient navigation in oncological care has expanded internationally and has been shown to significantly improve patient care experience, especially in the United States cancer care system. Like oncology care, patients who require epilepsy care face socioeconomic and healthcare system barriers and are at significant risk of morbidity and mortality if their care needs are not met. Although shortcomings in epilepsy care are longstanding, the COVID-19 pandemic has exacerbated these issues as both patients and providers have reported significant delays in care secondary to the pandemic. Prior to the pandemic, preliminary studies had shown the potential efficacy of patient navigation in improving epilepsy care. Considering the evidence that such programs are helpful for severely disadvantaged cancer patients and in enhancing epilepsy care, we believe that professional societies should support and encourage PN programs for coordinated and comprehensive care for patients with epilepsy. (C) 2020 Elsevier Inc. All rights reserved.
C1 [Anand, Sharath Kumar; Culver, Lauren G.] Wayne State Univ, Sch Med, 540 E Canfield St, Detroit, MI 48201 USA.
[Macki, Mohamed; Schwalb, Jason M.] Henry Ford Hosp, Dept Neurosurg, 2799 W Grand Blvd, Detroit, MI 48202 USA.
[Wasade, Vibhangini S.] Henry Ford Hosp, Dept Neurol, 2799 W Grand Blvd, Detroit, MI 48202 USA.
[Wasade, Vibhangini S.] Wayne State Univ, Sch Med, Dept Neurol, 540 E Canfield St, Detroit, MI 48201 USA.
[Hendren, Samantha] Univ Michigan, Dept Surg, Div Colorectal Surg, 1500 E Med Ctr Dr, Ann Arbor, MI 48109 USA.
[Schwalb, Jason M.] Henry Ford Hlth Syst, Ctr Hlth Policy & Hlth Serv Res, 2799 W Grand Blvd, Detroit, MI 48202 USA.
C3 Wayne State University; Henry Ford Hospital; Henry Ford Hospital; Wayne State University; University of Michigan System; University of Michigan; Henry Ford Hospital
RP Anand, SK (通讯作者), Wayne State Univ, Sch Med, 540 E Canfield St, Detroit, MI 48201 USA.
EM asharath@med.wayne.edu
TC 3
Z9 3
PD DEC
PY 2020
VL 113
AR 107530
DI 10.1016/j.yebeh.2020.107530
WC Behavioral Sciences; Clinical Neurology; Psychiatry
ER

PT J
AU Lima, MC
Sander, M
Lunardi, MD

Ribeiro, LC
Rieger, DK
Lin, K
Moreira, JD
AF Lima, Maiara C.
Sander, Mariana
Lunardi, Mariana dos Santos
Ribeiro, Leticia C.
Rieger, Debora K.
Lin, Katia
Moreira, Julia D.
TI Challenges in telemedicine for adult patients with drug-resistant epilepsy undergoing ketogenic diet treatment during the COVID-19 pandemic in the public healthcare system in Brazil
SO EPILEPSY & BEHAVIOR
LA English
DT Article
DE Epilepsy; Ketogenic diet; Pandemic; COVID-19; Telemedicine
AB Hygienic and sanitary measures and social distancing policies implemented during the new coronavirus disease - COVID-19 - pandemic have altered the care and follow-up provided by healthcare professionals for patients with chronic diseases, including patients with epilepsy (PWEs). Telemedicine has become a solution for the healthcare of PWEs in many developed countries. In this short communication, we trace a particular perspective for the application of telemedicine for PWEs undergoing ketogenic diet (KD) treatment, considering the social and economic difficulties faced by healthcare teams in resource-poor countries, such as Brazil. During the pandemic, financial strain was the main impediment to following KD. The pandemic increased socioeconomic insecurity and access to KD-related products, as well as increasing anxiety in 71% of PWE, impacting their KD treatment follow-up. The challenges of telemedicine in Brazil include not only social and economic issues but also access to food, healthcare services, and education for the population, in addition to digital inclusion. (C) 2020 Elsevier Inc. All rights reserved.
C1 [Lima, Maiara C.; Rieger, Debora K.; Moreira, Julia D.] Univ Fed Santa Catarina UFSC, Postgrad Program Nutr, Florianopolis, SC, Brazil.
[Sander, Mariana] Univ Fed Santa Catarina UFSC, Nutr, Florianopolis, SC, Brazil.
[Lunardi, Mariana dos Santos; Lin, Katia] Univ Fed Santa Catarina UFSC, Postgrad Program Med Sci, Florianopolis, SC, Brazil.
[Ribeiro, Leticia C.] Fed Univ Santa Catarina UFSC, Dept Nutr, Florianopolis, SC, Brazil.
[Lima, Maiara C.; Sander, Mariana; Lunardi, Mariana dos Santos; Ribeiro, Leticia C.; Rieger, Debora K.; Lin, Katia; Moreira, Julia D.] CNPq Directory Res Grp, Translat Nutr Neurosci Working Grp, Florianopolis, SC, Brazil.
C3 Universidade Federal de Santa Catarina (UFSC); Universidade Federal de Santa Catarina (UFSC); Universidade Federal de Santa Catarina (UFSC); Universidade Federal de Santa Catarina (UFSC)
RP Moreira, JD (通讯作者)，Univ Fed Santa Catarina UFSC, Campus Trindade,Rua Delfino Conti S Numero, BR-88040970 Florianopolis, SC, Brazil.

EM juliamoreira@gmail.com
TC 5
Z9 5
PD DEC
PY 2020
VL 113
AR 107529
DI 10.1016/j.yebeh.2020.107529
WC Behavioral Sciences; Clinical Neurology; Psychiatry
ER

PT J
AU Si, Y
Sun, LQ
Sun, HB
Niu, YL
Mo, QN
AF Si, Yang
Sun, Lingqi
Sun, Hongbin
Niu, Yulong
Mo, Qianning
TI Epilepsy management during epidemic: A preliminary observation from western China
SO EPILEPSY & BEHAVIOR
LA English
DT Article
DE Epilepsy; Management; COVID-19; Epidemic
AB Objective: This study aimed to investigate whether the proposed model could manage patients with epilepsy (PWEs) during the coronavirus disease 2019 (COVID-19) outbreak.

Methods: We used a model to manage the PWEs during the outbreak. Questionnaire survey and hospital data were used to explore whether PWEs under our management were affected by the virus.

Results: A total of 118 (78.7%) PWEs completed the survey. During the "model period," 22.9% (27/118) of the respondents reported antiepileptic drug (AEDs) discontinuity, including six (22.2%) PWEs who failed to purchase AEDs. Of the patients, 40.7% (22/54) failed to attend ordinary clinic, which was higher than that during the "period before model" (7.9%, 5/63). The common causes were movement limits (77.3%) and appointment failure (54.5%). A shift from ordinary clinic toward remote consultation was observed. Of the PWEs, 15.7% (13/83) referred to online pharmacy. 87.5% (14/16) of emergencies related to epilepsy were timely treated. 48.3%of PWEs thought that the epidemic had an impact on accessing medical services. Hospital data indicated that a decline in ordinary clinic visit, inpatient, surgery, and emergency attendance was observed in January and February 2020 and an increase in March 2020, as the epidemic mitigated. By contrast, online clinic visit soared in February, when the outbreak hit hard. In addition, we found no cross-infection of COVID-19 in our hospital and respondents.

Conclusion: We demonstrated a much-needed model to manage the PWEs during the

outbreak. We believed that the core architecture of this model was suitable for the management of other chronic diseases. (C) 2020 Published by Elsevier Inc.
C1 [Si, Yang; Sun, Hongbin; Niu, Yulong; Mo, Qianning] Sichuan Acad Med Sci, Chengdu 610072, Sichuan, Peoples R China.
[Si, Yang; Sun, Hongbin; Niu, Yulong] Sichuan Prov Peoples Hosp, Dept Neurol, Chengdu 610072, Sichuan, Peoples R China.
[Si, Yang] Univ Elect Sci & Technol China, Chengdu, Sichuan, Peoples R China.
[Sun, Lingqi] Southwest Med Univ, Luzhou 646000, Sichuan, Peoples R China.
[Sun, Hongbin; Niu, Yulong] Sichuan Univ, Key Lab Bioresource & Ecoenvironm, Minist Educ, Coll Life Sci, Chengdu 610065, Sichuan, Peoples R China.
[Mo, Qianning] Sichuan Prov Peoples Hosp, Med Adm Dept, Chengdu 610072, Peoples R China.
C3 Sichuan Provincial People's Hospital; Sichuan Provincial People's Hospital; University of Electronic Science & Technology of China; Southwest Medical University; Sichuan University; Sichuan Provincial People's Hospital
RP Mo, QN (通讯作者), 32 W Sec 2,1st Ring Rd, Chengdu 610072, Peoples R China.
EM yulong.niu@hotmail.com; sarahmok_mqn@hotmail.com
TC 0
Z9 0
PD DEC
PY 2020
VL 113
AR 107528
DI 10.1016/j.yebeh.2020.107528
WC Behavioral Sciences; Clinical Neurology; Psychiatry
ER

PT J
AU Subotic, A
Pricop, DF
Josephson, CB
Patten, SB
Smith, EE
Roach, P
AF Subotic, Arsenije
Pricop, Diana F.
Josephson, Colin B.
Patten, Scott B.
Smith, Eric E.
Roach, Pamela
CA Calgary Comprehensive Epilepsy Pro
TI Examining the impacts of the COVID-19 pandemic on the well-being and virtual care of patients with epilepsy
SO EPILEPSY & BEHAVIOR
LA English
DT Article

DE Epilepsy; COVID-19; Pandemic; Well-being; care; Quality of life
ID QUALITY-OF-LIFE; SOCIAL SUPPORT; ADULT PATIENTS; STIGMA; PEOPLE; BURDEN
AB Objective: The emergence of SARS-CoV-2 (COVID-19) as a novel coronavirus resulted in a global pandemic that necessitated the implementation of social distancing measures. These public health measures may have affected the provision of care for patients with epilepsy. Social isolation may have also adversely affected well-being and quality of life due to informal and formal support networks becoming less accessible. The purpose of this qualitative study was to examine the lived experiences of patients with epilepsy and to see how their quality of life and healthcare has been affected by the COVID-19 pandemic.

Methods: From April 27 to May 15, 2020 we performed remote interviews with 18 participants who had virtual appointments with their healthcare providers and were enrolled in the Calgary Comprehensive Epilepsy Program registry. Interviews were recorded and transcribed, after which transcripts were analyzed and coded into relevant themes using NVivo 12.

Results: Three broad themes emerged throughout the interviews:1) impact of pandemic on informal and formal support systems; 2) impact of pandemic on healthcare provision; and 3) concerns about the impact of the pandemic on personal situations and society in the future. Participants reported anxiety and stress about decreased social engagement and activity cessations. Although face-to-face appointments were preferred, virtual care was well-received. Common concerns about the future included securing employment and burnout from balancing family responsibilities. Some patients also feared they would be stigmatized as society adapted to the situation.

Significance: This study highlights the need for additional research in anticipation of the implementation of remote medicine in the management and treatment of epilepsy. It also highlights the tenacity of those living with epilepsy during difficult periods despite social and familial pressures. Raising awareness during this time about the lives and experiences of epilepsy patients can help challenge misconceptions and stigma in the workplace and wider society. (C) 2020 Elsevier Inc. All rights reserved.
C1 [Subotic, Arsenije; Pricop, Diana F.; Josephson, Colin B.; Smith, Eric E.; Roach, Pamela] Univ Calgary, Hotchkiss Brain Inst, Calgary, AB, Canada.

[Josephson, Colin B.; Patten, Scott B.; Roach, Pamela] Univ Calgary, Dept Community Hlth Sci, Calgary, AB, Canada.

[Josephson, Colin B.; Roach, Pamela] Univ Calgary, OBrien Inst Publ Hlth, Calgary, AB, Canada.

[Subotic, Arsenije; Pricop, Diana F.; Josephson, Colin B.; Smith, Eric E.] Univ Calgary, Dept Clin Neurosci, Calgary, AB, Canada.

[Josephson, Colin B.] Univ Calgary, Ctr Hlth Informat, Calgary, AB, Canada.

[Patten, Scott B.] Univ Calgary, Cuthbertson & Fischer Chair Pediat Mental Hlth, Calgary, AB, Canada.

[Roach, Pamela] Univ Calgary, Dept Family Med, Calgary, AB, Canada.

C3 University of Calgary; University of Calgary; University of Calgary; University of Calgary; University of Calgary; University of Calgary; University of Calgary

RP Roach, P (通讯作者), Dept Family Med, HSC G012,3330 Hosp Dr NW, Calgary, AB T2N 4N1, Canada.

EM pamela.roach@ucalgary.ca

TC 9
Z9 9
PD DEC
PY 2020
VL 113
AR 107599
DI 10.1016/j.yebeh.2020.107599
WC Behavioral Sciences; Clinical Neurology; Psychiatry
ER

PT J
AU Trivisano, M
Specchio, N
Pietrafusa, N
Calabrese, C
Ferretti, A
Ricci, R
Renzetti, T
Raponi, M
Vigevano, F
AF Trivisano, Marina
Specchio, Nicola
Pietrafusa, Nicola
Calabrese, Costanza
Ferretti, Alessandro
Ricci, Riccardo
Renzetti, Tommaso
Raponi, Massimiliano
Vigevano, Federico
TI Impact of COVID-19 pandemic on pediatric patients with epilepsy - The caregiver perspective
SO EPILEPSY & BEHAVIOR
LA English
DT Article
DE Pediatric epilepsy; COVID-19; Telemedicine; Remote consultation; Lockdown
ID TELEMEDICINE; CARE
AB The recent COVID-19 pandemic has disrupted care systems around the world. We assessed how the COVID-19 pandemic affected children with epilepsy in Italy, where lockdown measures were applied from March 8 to May 4, 2020. We compiled an Italian-language online survey on changes to healthcare and views on telehealth. Invitations were sent to 6631 contacts of all patients diagnosed with epilepsy within the last 5 years at the BambinoGesu Children's Hospital in Rome. Of the 3321 responses received, 55.6% of patients were seizure-free for at least 1 year before the COVID-19-related lockdown, 74.4% used anti-seizure medications (ASMs), and 59.7% had intellectual disability. Only 10 patients (0.4%) became infected with Severe Acute Respiratory Syndrome Coronavirus 2 (SARS-CoV-2). Seizure frequency remained stable for most patients during the lockdown

period (increased in 13.2%; decreased in 20.3%), and seizure duration, use of rescue medications, and adherence to treatment were unchanged. Comorbidities were more affected (behavioral problems worsened in 35.8%; sleep disorder worsened in 17.0%). Visits were canceled/postponed for 41.0%, but 25.1% had remote consultation during the lockdown period (93.9% were satisfied). Most responders (67.2%) considered continued remote consultations advantageous. Our responses support that patients/caregivers are willing to embrace telemedicine for some scenarios. (C) 2020 Elsevier Inc. All rights reserved.
C1 [Trivisano, Marina; Specchio, Nicola; Pietrafusa, Nicola; Calabrese, Costanza; Ferretti, Alessandro] Bambino Gesu Childrens Hosp IRCCS, Dept Neurol Sci, Rare & Complex Epilepsy Unit, European Reference Network EpiCARE, Rome, Italy.
[Ricci, Riccardo; Raponi, Massimiliano] Bambino Gesu Childrens Hosp IRCCS, Med Direct, Rome, Italy.
[Renzetti, Tommaso; Vigevano, Federico] Bambino Gesu Childrens Hosp IRCCS, European Reference Network EpiCARE, Dept Neurol Sci, Rome, Italy.
C3 IRCCS Bambino Gesu; IRCCS Bambino Gesu; IRCCS Bambino Gesu
RP Specchio, N (通讯作者), Bambino Gesu Pediat Hosp, IRCCS, Rare & Complex Epilepsy Unit, Dept Neurosci, Pzza S Onofrio 4, I-00165 Rome, Italy.
EM nicola.specchio@opbg.net
TC 8
Z9 8
PD DEC
PY 2020
VL 113
AR 107527
DI 10.1016/j.yebeh.2020.107527
WC Behavioral Sciences; Clinical Neurology; Psychiatry
ER

PT J
AU Domingues, RB
Mantese, CE
Aquino, ED
Fantini, FGMM
do Prado, GF
Nitrini, R
AF Domingues, Renan Barros
Mantese, Carlos Eduardo
Aquino, Emanuelle da Silva
Malheiro Moraes Fantini, Francisca Goreth
do Prado, Gilmar Fernandes
Nitrini, Ricardo
TI Telemedicine in neurology: current evidence
SO ARQUIVOS DE NEURO-PSIQUIATRIA
LA English
DT Article
DE Telemedicine; Neurology; Coronavirus Infections; Teleneurology

ID RANDOMIZED CONTROLLED-TRIAL; MONTREAL COGNITIVE ASSESSMENT; ACUTE ISCHEMIC-STROKE; QUALITY-OF-LIFE; MULTIPLE-SCLEROSIS; NON-INFERIORITY; FOLLOW-UP; EPILEPSY; PILOT; CARE

AB Background: Telemedicine was first introduced in Neurology as a tool to facilitate access to acute stroke treatment. More recently, evidence has emerged of the use of telemedicine in several other areas of Neurology. With the advent of the COVID-19 pandemic and the need for social isolation, Brazilian authorities have expanded the regulation of the use of telemedicine, thus allowing the treatment of many patients with neurological diseases to be conducted with less risk of SARS-CoV-2 contamination. Objective: This study aimed to critically review the current evidence of the use, efficacy, safety, and usefulness of telemedicine in Neurology. Methods: A review of PubMed indexed articles was carried out by searching for the terms "telemedicine AND": "headache", "multiple sclerosis", "vestibular disorders", "cerebrovascular diseases","epilepsy","neuromuscular diseases","dementia", and "movement d isorders". The more relevant studies in each of these areas were critically analyzed. Results: Several articles were found and analyzed in each of these areas of Neurology. The main described contributions of telemedicine in the diagnosis and treatment of such neurological conditions were presented, indicating a great potential of use of this type of assistance in all these fields. Conclusion: Current evidence supports that teleneurology can be a tool to increase care for patients suffering from neurological diseases.

C1 [Domingues, Renan Barros; Mantese, Carlos Eduardo; Aquino, Emanuelle da Silva] Acad Brasileira Neurol, Comissao Aberta Telemed, Sao Paulo, SP, Brazil.
[Domingues, Renan Barros] Santa Casa Misericordia Sao Paulo, Dept Neurol, Sao Paulo, SP, Brazil.
[Mantese, Carlos Eduardo; Aquino, Emanuelle da Silva] Hosp Sirio Libanes, Telemed, Sao Paulo, SP, Brazil.
[Mantese, Carlos Eduardo] Hosp Mae Deus, Porto Alegre, RS, Brazil.
[Aquino, Emanuelle da Silva; Nitrini, Ricardo] Univ Sao Paulo, Dept Neurol, Sao Paulo, SP, Brazil.
[Malheiro Moraes Fantini, Francisca Goreth] Acad Brasileira Neurol, Comissao Exercicio Profiss, Sao Paulo, SP, Brazil.
[do Prado, Gilmar Fernandes] Acad Brasileira Neurol, Diretoria Execut, Sao Paulo, SP, Brazil.
[do Prado, Gilmar Fernandes] Univ Fed Sao Paulo, Dept Neurol, Sao Paulo, SP, Brazil.
[Nitrini, Ricardo] Acad Brasileira Neurol, Diretoria Cient, Sao Paulo, SP, Brazil.
C3 Hospital Sirio Libanes; Universidade de Sao Paulo; Universidade Federal de Sao Paulo (UNIFESP)
RP Domingues, RB (通讯作者)，Acad Brasileira Neurol, Comissao Aberta Telemed, Sao Paulo, SP, Brazil.; Domingues, RB (通讯作者)， Santa Casa Misericordia Sao Paulo, Dept Neurol, Sao Paulo, SP, Brazil.
EM contato@renandomingues.med.br

TC 10
Z9 11
PD DEC
PY 2020
VL 78

IS 12
BP 818
EP 826
DI 10.1590/0004-282X20200131
WC Neurosciences; Psychiatry
ER

PT J
AU Mostacci, B
Licchetta, L
Cacciavillani, C
Di Vito, L
Ferri, L
Menghi, V
Stipa, C
Avoni, P
Provini, F
Muccioli, L
Vignatelli, L
Mazzoni, S
Tinuper, P
Bisulli, F
AF Mostacci, Barbara
Licchetta, Laura
Cacciavillani, Carlotta
Di Vito, Lidia
Ferri, Lorenzo
Menghi, Veronica
Stipa, Carlotta
Avoni, Patrizia
Provini, Federica
Muccioli, Lorenzo
Vignatelli, Luca
Mazzoni, Stefania
Tinuper, Paolo
Bisulli, Francesca
TI The Impact of the COVID-19 Pandemic on People With Epilepsy. An Italian Survey and a Global Perspective
SO FRONTIERS IN NEUROLOGY
LA English
DT Article
DE COVID-19; emergency; epilepsy; survey; telemedicine
ID TELEMEDICINE; STRESS
AB Objectives: We explored the impact of the coronavirus disease-19 (COVID-19) emergency on the health of people with epilepsy (PwE). We also investigated their attitude toward telemedicine.
Methods: The PubMed database up to September 10, 2020 was searched for

questionnaire-based studies conducted in PwE during the COVID-19 emergency, and the literature retrieved was reviewed. In addition, all patients who had a telephone consultation with our center between May 7 and July 31, 2020 were invited to fill in a 57-item online questionnaire focusing on epilepsy and comorbidities, any changes in lifestyle or clinical conditions and any emergency-related problems arising during the COVID-19 emergency, and their views on telemedicine. Associations between variables were detected through X-2 test and Fisher's exact test. Univariate and multivariate logistic regression models were used to evaluate the effects of different factors on clinical conditions.

Results: Twelve studies met the literature search criteria. They showed that the rate of seizure worsening during the emergency ranged from 4 to 35% and was mainly correlated with epilepsy severity, sleep disturbances and COVID-19-related issues. Our questionnaire was filled in by 222 PwE or caregivers. One hundred (76.6%) reported unchanged clinical conditions, 25 (11.3%) an improvement, and 27 (12%) a deterioration. Reported clinical worsening was associated with a psychiatric condition and/or medication (OR = 12.59, $p < 0.001$), sleep disorders (OR = 8.41, $p = 0.001$), limited access to healthcare (OR = 4.71, $p = 0.016$), and experiencing seizures during the emergency (OR = 4.51, $p = 0.007$). Telemedicine was considered acceptable by 116 subjects (52.3%).

Conclusions: Most PwE did not experience a significant change in their clinical conditions during the COVID-19 emergency. However, severity of epilepsy, concomitant disability, comorbid psychiatric conditions, sleep disorders and limited access to healthcare may affect their health.
C1 [Mostacci, Barbara; Licchetta, Laura; Di Vito, Lidia; Stipa, Carlotta; Avoni, Patrizia; Provini, Federica; Vignatelli, Luca; Mazzoni, Stefania; Tinuper, Paolo; Bisulli, Francesca] IRCCS Ist Sci Neurol Bologna, Bologna, Italy.

[Mostacci, Barbara; Licchetta, Laura; Di Vito, Lidia; Stipa, Carlotta; Avoni, Patrizia; Provini, Federica; Vignatelli, Luca; Mazzoni, Stefania; Tinuper, Paolo; Bisulli, Francesca] ERN EpiCare, Bologna, Italy.

[Licchetta, Laura; Cacciavillani, Carlotta; Ferri, Lorenzo; Menghi, Veronica; Avoni, Patrizia; Provini, Federica; Muccioli, Lorenzo; Tinuper, Paolo; Bisulli, Francesca] Univ Bologna, Dept Biomed & Neuromotor Sci, Bologna, Italy.

C3 IRCCS Istituto delle Scienze Neurologiche di Bologna (ISNB); University of Bologna

RP Licchetta, L (通讯作者), IRCCS Ist Sci Neurol Bologna, Bologna, Italy.; Licchetta, L (通讯作者), ERN EpiCare, Bologna, Italy.; Licchetta, L (通讯作者), Univ Bologna, Dept Biomed & Neuromotor Sci, Bologna, Italy.

EM laura.licchetta2@unibo.it
TC 11

Z9 11

PD DEC 18

PY 2020

VL 11

AR 613719

DI 10.3389/fneur.2020.613719

WC Clinical Neurology; Neurosciences

ER

PT J
AU Kristoffersen, ES
Sandset, EC
Winsvold, BS
Faiz, KW
Storstein, AM
AF Kristoffersen, Espen Saxhaug
Sandset, Else Charlotte
Winsvold, Bendik Slagsvold
Faiz, Kashif Waqar
Storstein, Anette Margrethe
TI Experiences of telemedicine in neurological out-patient clinics during the COVID-19 pandemic
SO ANNALS OF CLINICAL AND TRANSLATIONAL NEUROLOGY
LA English
DT Article
DE COVID-19; telemedicine; epilepsy; out-patient
ID TRIAL
AB Objective: The COVID-19 pandemic has led to rapid changes in the delivery of medical care worldwide. The main objective of this survey was to investigate the initial experiences of neurologists with the use of telemedicine for different neurological conditions during the first phase of the COVID-19.Methods: All hospital-based neurologists in Norway (n = 400) were invited to a questionnaire survey by e-mail in April 2020. The study focused on telemedicine and all questions were answered with regard to the first weeks of the pandemic lockdown in Norway.Results:One-hundred and thirty-five neurologists responded. Overall, 87% reported a shift toward more telemedicine, with significantly more use of telephone than video consultations for both new referrals (54% vs. 30%, $P < 0.001$) and follow-ups (99% vs. 50%, $P < 0.001$). Respondents deemed it much more professionally satisfactory to conduct follow-up consultations by telephone, than to carry out consultations with new patients by telephone (85% vs. 13%, $P < 0.001$). Teleconsultations were better suited for headache and epilepsy patients as compared to multiple sclerosis and movement disorder patients. There was no significant difference between residents and senior consultants regarding how they experienced teleconsultations. Female neurologists found telemedicine better and more effective than male neurologists. Interpretation Telemedicine was rapidly implemented in Norwegian neurological departments during the first weeks of the COVID-19 pandemic. Teleconsultations were better suited for follow-ups than for new referrals, and better for headache and epilepsy patients as compared to multiple sclerosis and movement disorder patients.
C1 [Kristoffersen, Espen Saxhaug; Faiz, Kashif Waqar] Akershus Univ Hosp, Dept Neurol, POB 1000, N-1478 Lorenskog, Norway.
[Kristoffersen, Espen Saxhaug] Univ Oslo, Dept Gen Practice, Oslo, Norway.
[Sandset, Else Charlotte; Winsvold, Bendik Slagsvold] Oslo Univ Hosp, Dept Neurol, Oslo, Norway.
[Sandset, Else Charlotte] Norwegian Air Ambulance Fdn, Oslo, Norway.
[Winsvold, Bendik Slagsvold] Oslo Univ Hosp, Dept Res Innovat & Educ, Div Clin

Neurosci, Oslo, Norway.
[Storstein, Anette Margrethe] Haukeland Hosp, Dept Neurol, Bergen, Norway.
C3 University of Oslo; University of Oslo; University of Oslo; University of Oslo; University of Bergen; Haukeland University Hospital
RP Kristoffersen, ES (通讯作者), Akershus Univ Hosp, Dept Neurol, POB 1000, N-1478 Lorenskog, Norway.
EM e.s.kristoffersen@medisin.uio.no
TC 18
Z9 18
PD FEB
PY 2021
VL 8
IS 2
BP 440
EP 447
DI 10.1002/acn3.51293
EA DEC 2020
WC Clinical Neurology; Neurosciences
ER

PT J
AU Tao, DS
Zhong, TW
Wang, JL
AF Tao, Deshuang
Zhong, Tangwu
Wang, Juli
TI The Influence of Telemedicine and Compassionate Care on the Quality of Life and Mental Health of Patients with Epilepsy in Northeastern China During the COVID-19 Crisis
SO JOURNAL OF MULTIDISCIPLINARY HEALTHCARE
LA English
DT Article
DE telemedicine; care; QOLIE-31; GAD-7; COVID-19; epilepsy
ID EXPERIENCE; PEOPLE; TIME; TOOL
AB Purpose: To understand the influence of telemedicine and compassionate care on the quality of life and mental health of patients with epilepsy (PWE) in northeastern China during the COVID-19 crisis.
Patients and Methods: Physicians in the epilepsy department conducted a questionnaire survey on PWE on February 2020. The Quality Of Life In Epilepsy-31 (QOLIE-31), Generalized Anxiety Disorder 7-Item Scale (GAD-7) and Patient Health Questionnaire-9 (PHQ-9) were used. The intervention (IG) group received compassionate care and follow-up through telemedicine equipment every week, while the nonintervention (NIG) group did not receive. The questionnaire survey was conducted again three month later.
Results: Ninety patients were recruited: mean age 39.91 +/- 15.57 in the IG, 37.39 +/- 11.69 in the NIG, 46 (51.1%) were men. Twenty patients had difficulty in purchasing

antiepileptic drugs (AEDs). Seven patients reported seizure in the last 1 month. Only 1 patient (2.2%) consulted the emergency department. Up to 84 patients' lives were affected. Fifteen (33.3%) of the IG and 20 (44.4%) of the NIG patients stated that their family income had decreased, and among them, 13 (28.9%) in the IG group and 10 (22.2%) in the NIG group stated that they were unemployed. 3 months later, the interaction between groups and time of QOLIE-31 was significant, F (1, 88) = 16.996, p<0.001; the interaction between group and time on the PHQ-9 was significant, F (1, 88)= 14.992, p<0.001; the interaction between group and time on the QAD-7 was significant, F (1, 88)= 6.026, p<0.001.

Conclusion: Our study found that during the COVID-19 outbreak, when patients were in a lockdown, telemedicine and compassionate care were effective and successful in managing PWE in northeastern China. It is a valid method to decrease anxiety and depression and improve the patients' quality of life. Further research is necessary about compassionate care methods for PWE.
C1 [Tao, Deshuang; Zhong, Tangwu; Wang, Juli] Jiamusi Cent Hosp, Epilepsy Dept, Jiamusi City 154002, Heilongjiang, Peoples R China.

[Tao, Deshuang; Zhong, Tangwu] Jiamusi Univ, Jiamusi City 154007, Heilongjiang, Peoples R China.

C3 Jiamusi University

RP Wang, JL (通讯作者), Jiamusi Cent Hosp, Epilepsy Dept, Jiamusi City 154002, Heilongjiang, Peoples R China.

EM wjl198981@163.com
TC 0

Z9 0

PY 2021

VL 14

BP 3359

EP 3368

DI 10.2147/JMDH.S335240

WC Health Care Sciences & Services

ER

PT J

AU Moura, LMVR

Donahue, MA

Smith, JR

Dass, D

Sanches, PR

Ayub, N

McGraw, C

Zafar, SF

Cash, SS

Hoch, DB

AF Moura, Lidia M. V. R.

Donahue, Maria A.

Smith, Jason R.

Dass, Deepika

Sanches, Paula R.
Ayub, Neishay
McGraw, Christopher
Zafar, Sahar F.
Cash, Sydney S.
Hoch, Daniel B.
TI Telemedicine Can Support Measurable and High-Quality Epilepsy Care During the COVID-19 Pandemic
SO AMERICAN JOURNAL OF MEDICAL QUALITY
LA English
DT Article
DE telemedicine; health services research; care; electronic health records; epilepsy
ID FOLLOW-UP CARE; AMERICAN ACADEMY; ACTIVE EPILEPSY; ILAE COMMISSION; POSITION PAPER; UNITED-STATES; WORK GROUP; TELEHEALTH; CLASSIFICATION; SERVICES
AB Routine outpatient epilepsy care has shifted from in-person to telemedicine visits in response to safety concerns posed by the coronavirus disease 2019 (COVID-19) pandemic. But whether telemedicine can support and maintain standardized documentation of high-quality epilepsy care remains unknown. In response, the authors conducted a quality improvement study at a level 4 epilepsy center between January 20, 2019, and May 31, 2020. Weekly average completion proportion of standardized documentation used by a team of neurologists for adult patients for the diagnosis of epilepsy, seizure classification, and frequency were analyzed. By December 15, 2019, a 94% average weekly completion proportion of standardized epilepsy care documentation was achieved that was maintained through May 31, 2020. Moreover, during the period of predominately telemedicine encounters in response to the pandemic, the completion proportion was 90%. This study indicates that high completion of standardized documentation of seizure-related information can be sustained during telemedicine appointments for routine outpatient epilepsy care at a level 4 epilepsy center.
C1 [Moura, Lidia M. V. R.; Donahue, Maria A.; Smith, Jason R.; Dass, Deepika; Sanches, Paula R.; Ayub, Neishay; McGraw, Christopher; Zafar, Sahar F.; Cash, Sydney S.; Hoch, Daniel B.] Massachusetts Gen Hosp, Dept Neurol, Boston, MA 02114 USA.
[Moura, Lidia M. V. R.; McGraw, Christopher; Zafar, Sahar F.; Cash, Sydney S.; Hoch, Daniel B.] Harvard Med Sch, Dept Neurol, Boston, MA 02115 USA.
[Moura, Lidia M. V. R.] Harvard TH Chan Sch Publ Hlth, Dept Epidemiol, Boston, MA USA.
[Dass, Deepika] Massachusetts Gen Hosp, Mongan Inst, Hlth Policy Res Ctr, Boston, MA 02114 USA.
[Sanches, Paula R.] Hosp Israelita Albert Einstein, Dept Crit Care Med, Sao Paulo, Brazil.
C3 Harvard University; Massachusetts General Hospital; Harvard University; Harvard Medical School; Harvard University; Harvard T.H. Chan School of Public Health; Harvard University; Massachusetts General Hospital; Hospital Israelita Albert Einstein
RP Moura, LMVR (通讯作者), Harvard Med Sch, Dept Neurol, Massachusetts Gen Hosp, Wang 739D,55 Fruit St, Boston, MA 02114 USA.

EM lidia.moura@mgh.harvard.edu
TC 1
Z9 1
PD JAN-FEB
PY 2021
VL 36
IS 1
BP 5
EP 16
DI 10.1097/01.JMQ.0000733444.71245.6c
WC Health Care Sciences & Services
ER

PT J
AU Bergantin, LB
AF Bergantin, Leandro B.
TI The Interplay Among Epilepsy, Parkinson's Disease and Inflammation: Revisiting the Link through Ca2+/cAMP Signalling
SO CURRENT NEUROVASCULAR RESEARCH
LA English
DT Article
DE Epilepsy; Parkinson's disease; Ca2+/cAMP signalling; Ca2+ channel blockers; pharmacotherapy; COVID-19
ID CALCIUM ADMINISTRATION INCREASES; INTRANIGRAL INJECTION; TRANSGENIC MICE; SEIZURES; DEGENERATION; OVEREXPRESSION; MORTALITY; PREVENTS; CHANNELS; THERAPY
AB Background: Robust evidence has described that Parkinson's disease (PD) is associated with an increased risk for developing epileptic seizures. In fact, an interplay between PD and epilepsy has been of interest for many years. An emerging hypothesis is that inflammation could link both diseases.

Objective: Bearing in mind the experience of our group in the field of Ca2+/cAMP signalling pathways, this article discussed, beyond inflammation, the role of these signalling pathways in this link between PD and epilepsy.

Methods: Publications involving Ca2+/cAMP signalling pathways, PD, and epilepsy (alone or combined) were collected by searching PubMed and EMBASE.

Results: The comprehension of the interplay between PD and epilepsy could improve the drug therapy. In addition, a Ca2+ signalling dyshomeostasis due to Coronavirus disease 2019 (COVID-19), an emerging and rapidly evolving situation, has been reported.

Conclusion: Thus, this article also debated recent findings about therapeutics involving Ca2+ channel blockers for preventing Ca2+ signalling dyshomeostasis due to COVID-19, including the correlation among COVID-19, epilepsy, and PD.
C1 [Bergantin, Leandro B.] Univ Fed Sao Paulo, Dept Pharmacol, Escola Paulista Med, Rua Pedro Toledo 669, BR-04039032 Sao Paulo, SP, Brazil.
C3 Universidade Federal de Sao Paulo (UNIFESP)
RP Bergantin, LB (通讯作者)，Univ Fed Sao Paulo, Dept Pharmacol, Escola Paulista Med, Rua Pedro Toledo 669, BR-04039032 Sao Paulo, SP, Brazil.
EM leanbio39@yahoo.com.br

TC 0
Z9 0
PY 2021
VL 18
IS 1
BP 162
EP 168
DI 10.2174/1567202618666210603123345
WC Clinical Neurology; Neurosciences
ER

PT J
AU Zia, S
Khan, AN
Zaidi, KS
Ali, SE
AF Zia, Shafaq
Khan, Ali Nawaz
Zaidi, Khurram Shabih
Ali, Shan E.
TI Detection of Generalized Tonic Clonic Seizures and Falls in Unconstraint Environment Using Smartphone Accelerometer
SO IEEE ACCESS
LA English
DT Article
DE Sensors; Accelerometers; Wearable sensors; Sensor phenomena and characterization; Feature extraction; Support vector machines; Neurological disease; Accelerometer; activity recognition; biomedical signal processing; assisted living; machine learning
AB The detection of Generalized Tonic Clonic Seizures (GTCS) and Falls is of utmost importance due to the increase in prevalence of epilepsy and Sudden Death in Epileptic Patients during CoVID-19 pandemic, and prevention of serious injuries in Fall risk groups such as elderly requiring continuous monitoring for disease management and assisted living etc. Monitoring of Activities of Daily Living (ADLs) can assist in the detection of symptoms and onset of neurological disorders such as Alzheimer's, stroke, and epileptic seizures. With a host of embedded sensors, improved memory, enhanced processing capabilities and availability to masses, smartphones can be used for Human Activity Recognition (HAR) through continuous monitoring of ADLs. This paper presents a tri-axial accelerometer-based approach to detect and classify activities performed by individuals by applying machine learning algorithms including RF, J48, NB, LMT and SVM to movement data. Movement data is collected in real-time from the embedded accelerometer of a smartphone worn by individual on upper-left arm in unconstraint environment. It is pre-processed using time and frequency domain analysis and spatial domain features are computed. Supervised machine learning techniques are applied to classify ADLs into five classes based on the intensity of movements: Stationary, Light Ambulatory, Intense Ambulatory, GTCS and Falls. We also used training data from MyNeuroHealth dataset collected from 23 individuals including epilepsy patients. Based

on gathered results, Random Forest outperforms other classifiers with classification accuracy of 99.6% for stationary, 81.5% for light ambulatory, 99.8% for intense ambulatory and GTCS, and 97.2% for Falls corresponding to training data of 14000 samples. To date, activity classification in our system has been implemented on cloud instead of mobile phone application as subjects are using smartphones with dissimilar software and hardware specifications for assisted living applications.
C1 [Zia, Shafaq; Khan, Ali Nawaz; Zaidi, Khurram Shabih; Ali, Shan E.] COMSATS Univ Islamabad, Dept Elect & Comp Engn, Lahore Campus, Lahore 54000, Pakistan.
C3 COMSATS University Islamabad (CUI)
RP Zia, S (通讯作者), COMSATS Univ Islamabad, Dept Elect & Comp Engn, Lahore Campus, Lahore 54000, Pakistan.
EM s.zia@ieee.org
TC 0
Z9 0
PY 2021
VL 9
BP 39432
EP 39443
DI 10.1109/ACCESS.2021.3063765
WC Computer Science, Information Systems; Engineering, Electrical & Electronic; Telecommunications
ER

PT J
AU Sureka, RK
Gaur, V
Gupta, M
AF Sureka, Rajendra K.
Gaur, Vikas
Gupta, Medha
TI Impact of COVID-19 on People Suffering with Epilepsy
SO ANNALS OF INDIAN ACADEMY OF NEUROLOGY
LA English
DT Article
DE Anxiety; COVID-19; depression; epilepsy; seizure; survey
AB Background: Epilepsy is one of the most common problems in neurology clinical practice and currently we are in the midst of the coronavirus outbreak. The coronavirus pandemic is an epidemiological and psychological crisis, which is likely to affect persons with epilepsy. Objectives: This study was designed to evaluate the impact of COVID-19 pandemic on patients with epilepsy and effects on their mental health. Materials and Methods: This was a cross-sectional web-based survey carried out at the department of Neurology at a tertiary care hospital. A questionnaire was designed in the local language using Google Forms to assess basic knowledge regarding epilepsy, coronavirus, effects of COVID-19 and lockdown on epileptic patients and also effects on their mental health. The link to the online survey was distributed via WhatsApp messenger to epilepsy patients. Results: One hundred fifty-six cases were enrolled with 69.1% were below 34 years of age and male: female ratio was 1.2:1. Only 34.3% of the

participants were employed and 50% of patients had an income of less than Rs. 3000 per month. Of the patients enrolled, 20.5% reported the "devil" and superstitions as a cause of epilepsy and only 10% of patients thought that tantric (holy priest) could treat the disease better than doctors. 53.8% of patients worried about getting COVID-19 and could not stop thoughts about being infected by coronavirus bothering them. 30.3% patients had increased seizure frequency during COVID-19 pandemic, of which the most common reason was that they forgot to take regular antiepileptic drugs (22.7%) or they had faced difficulty in obtaining medicine due to lockdown (12.1%). During the pandemic, 17% of patients reported depression symptoms and another 21% reported anxiety symptoms. Conclusion: The current COVID-19 pandemic negatively affected patients with epilepsy and increased seizure frequency, depression, anxiety, unemployment, and financial difficulty in obtaining medication.
C1 [Sureka, Rajendra K.; Gupta, Medha] Mahatma Gandhi Med Coll, Dept Neurol, Jaipur, Rajasthan, India.
[Gaur, Vikas] Mahatma Gandhi Med Coll, Dept Psychiat, Jaipur, Rajasthan, India.
RP Sureka, RK (通讯作者), 47 Sanjay Marg, Jaipur 302001, Rajasthan, India.
EM rsureka@rediffmail.com
TC 4
Z9 4
PD JAN-FEB
PY 2021
VL 24
IS 1
BP 51
EP 55
DI 10.4103/aian.AIAN_623_20
WC Clinical Neurology
ER

PT J
AU Wanigasinghe, J
Jayawickrama, A
Hewawitharana, G
Munasinghe, J
Weeraratne, CT
Ratnayake, P
Wijesekara, DS
Fernando, S
Rupasinghe, P
AF Wanigasinghe, Jithangi
Jayawickrama, Ashan
Hewawitharana, Gemunu
Munasinghe, Jagath
Weeraratne, Chathurika T.
Ratnayake, Pyara
Wijesekara, Dimuthu S.
Fernando, Sanjaya

Rupasinghe, Priyanka
TI Experience during COVID-19 lockdown and self-managing strategies among caregivers of children with epilepsy: A study from low middle income country
SO SEIZURE-EUROPEAN JOURNAL OF EPILEPSY
LA English
DT Article
DE COVID-19; Caregivers; Children epilepsy; Stress; Self-management
AB Purpose: Abrupt halt of service provision due to pandemic state of COVID-19, significantly affected care of patients with chronic diseases like epilepsy; its impact being greater on caregivers of vulnerable groups such as children with epilepsy. We performed this study to describe difficulties posed by the lockdown to caregivers of children with epilepsy in a low-middle income country and describe their responses and self-management strategies to overcome difficulties and prepare for a recurrence. Method: A cross-sectional all-island survey was carried out at paediatric neurology centers in Sri Lanka. Data was gathered via a face-to-face interview after the lockdown period. Parental stress level was evaluated using a self rating Stress Assessment Questionnaire. Results: Caregivers of 140 children with epilepsy from seven centers served by paediatric neurologists were interviewed. Mean duration of epilepsy was 7.9 years(SD 4). Majority were on one (52.1 %) or two (20 %) anti seizure medications regularly. The pandemic did not affect epilepsy control in majority (87.3 %), however, signficant proportion faced difficulties over regular reviews and presecription refills. Despite diffícluties, 87.1 % of parents maintained dispensing anti-seizure medications to their child regularly. Caregivers demonstrated healthy self-management strategies such as awareness on medications and access methods to healthcare during lockdown and remained confident of accessability to services. Stress was experienced in < 5%. Conclusion: Lockdown status for COVID-19 did not significantly affect the control of epilepsy in children though it posed difficulties for regular reviews and obtaining medications. Self-management strategies will help caregivers to adopt to new-normal status and potential future outbreaks.
C1 [Wanigasinghe, Jithangi; Jayawickrama, Ashan] Univ Colombo, Dept Paediat, Fac Med, Colombo, Sri Lanka.
[Hewawitharana, Gemunu] Teaching Hosp, Karapitiya, Sri Lanka.
[Munasinghe, Jagath] Sirimavo Bandaranayaike Specialized Childrens Hos, Peradeniya, Sri Lanka.
[Weeraratne, Chathurika T.] Teaching Hosp, Anuradhapura, Sri Lanka.
[Ratnayake, Pyara] Lady Ridgeway Hosp Children, Colombo, Sri Lanka.
[Wijesekara, Dimuthu S.] Univ Sri Jayewardenepura, Dept Paediat, Fac Med Sci, Nugegoda, Sri Lanka.
[Fernando, Sanjaya] North Colombo Teaching Hosp, Ragama, Sri Lanka.
[Rupasinghe, Priyanka] Teaching Hosp, Batticaloa, Sri Lanka.
C3 University of Colombo; University Sri Jayewardenepura
RP Wanigasinghe, J (通讯作者), Univ Colombo, Dept Paediat, Fac Med, Colombo, Sri Lanka.
EM jithangi@gmail.com
TC 3
Z9 3
PD JAN

PY 2021
VL 84
BP 112
EP 115
DI 10.1016/j.seizure.2020.12.001
WC Clinical Neurology; Neurosciences
ER

PT J
AU Brisca, G
   Siri, L
   Olcese, C
   Brunenghi, BM
   Pirlo, D
   Mancardi, MM
AF Brisca, Giacomo
   Siri, Laura
   Olcese, Camilla
   Brunenghi, Bernadette Marre
   Pirlo, Daniela
   Mancardi, Maria Margherita
TI Is SARS-CoV-2 Infection a Risk for Potentiation of Epileptic Seizures in
   Children With Pre-existing Epilepsy?
SO PEDIATRIC NEUROLOGY
LA English
DT Letter
DE COVID-19; Epilepsy; SARS-CoV-2; Seizure
C1 [Brisca, Giacomo; Pirlo, Daniela] IRCCS Giannina Gaslini Inst, Subintens Care Unit, Genoa, Italy.
   [Siri, Laura; Mancardi, Maria Margherita] IRCCS Giannina Gaslini Inst, Unit Child Neuropsychiat, Clin & Surg Neurosci Dept, Genoa, Italy.
   [Olcese, Camilla; Brunenghi, Bernadette Marre] Univ Genoa, Dept Neurosci Rehabil Ophthalmol Genet Maternal &, Genoa, Italy.
C3 University of Genoa; IRCCS Istituto Giannina Gaslini; University of
   Genoa; IRCCS Istituto Giannina Gaslini; University of Genoa
RP Brisca, G (通讯作者), IRCCS Giannina Gaslini Inst, Subintens Care Unit, Genoa, Italy.
EM giacomobrisca@gaslini.org
TC 2
Z9 2
PD MAR
PY 2021
VL 116
BP 31
EP 31
DI 10.1016/j.pediatrneurol.2020.11.021
EA JAN 2021
WC Clinical Neurology; Pediatrics

ER

PT J
AU Feyissa, AM
AF Feyissa, Anteneh M.
TI Hold the Smartphone! Tele-epilepsy in a Post-COVID-19 World
SO MAYO CLINIC PROCEEDINGS
LA English
DT Editorial Material
DE COVID-19; Epilepsy; telemedicine
C1 [Feyissa, Anteneh M.] Mayo Clin, Dept Neurol, 4500 San Pablo Rd, Jacksonville, FL 32224 USA.
C3 Mayo Clinic
RP Feyissa, AM (通讯作者), Mayo Clin, Dept Neurol, 4500 San Pablo Rd, Jacksonville, FL 32224 USA.
EM Feyissa.Anteneh@mayo.edu
TC 2
Z9 2
PD JAN
PY 2021
VL 96
IS 1
BP 4
EP 6
DI 10.1016/j.mayocp.2020.11.010
EA JAN 2021
WC Medicine, General & Internal
ER

PT J
AU Kubota, T
Kuroda, N
AF Kubota, Takafumi
Kuroda, Naoto
TI Exacerbation of neurological symptoms and COVID-19 severity in patients with preexisting neurological disorders and COVID-19: A systematic review
SO CLINICAL NEUROLOGY AND NEUROSURGERY
LA English
DT Review
DE COVID-19; Neurological disease; Neurology; Epilepsy; Systematic; Review
ID DELIRIUM
AB Background: Patients with chronic diseases likely develop severe 2019 coronavirus disease (COVID-19). However, little is known about the effects of COVID-19 on patients with neurological disorders. We conducted a systematic review to evaluate the severity of COVID-19 and its effect on neurological symptoms in patients with preexisting neurological disorder and COVID-19.

Methods: We searched the MEDLINE (PubMed) and medRxiv databases for reports of patients with both preexisting neurological disorders and COVID-19. Studies reporting data on changes in the symptoms of preexisting neurological disorders and/or the severity of COVID-19 were included.

Results: Twenty-six articles with 2278 patients with preexisting neurological disorder and COVID-19 were identified. Of 232 patients, 74 (31.9 %) showed exacerbation of preexisting neurological symptoms of dementia (55/92; 59.5 %), Parkinson's disease (10/17; 58.8 %), epilepsy (1/1; 100 %), and unspecified neurological disorders (8/106; 7.5 %). Of 2168 patients, 478 (22.0 %) showed severe COVID-19 course. These included patients with cerebrovascular disease (86/445; 19.3 %), dementia (70/316; 22.2 %), Parkinson's disease (25/ 214; 11.7 %), multiple sclerosis (28/71; 39.4 %), spinal cord injury (5/7; 71.4 %), epilepsy (10/98; 10.2 %) and unspecified neurological disorders (254/1011; 25 %).

Conclusions: Patients with preexisting neurological disorders and COVID-19 may develop exacerbation of neurological symptoms and severe COVID-19. Clinicians should be aware of the risk of symptom exacerbation and severe COVID-19 in patients with preexisting neurological disease and should focus on the prevention and early care of COVID-19.
C1 [Kubota, Takafumi] Univ Hosp Cleveland, Med Ctr, Dept Neurol, Cleveland, OH 44106 USA.

[Kubota, Takafumi] Case Western Reserve Univ, Dept Neurol, Cleveland, OH 44106 USA.

[Kuroda, Naoto] Wayne State Univ, Dept Pediat, Detroit, MI 48202 USA.

[Kuroda, Naoto] Tohoku Univ, Grad Sch Med, Dept Epileptol, Sendai, Miyagi, Japan.

C3 Case Western Reserve University; Case Western Reserve University Hospital; University Hospitals of Cleveland; Case Western Reserve University; Wayne State University; Tohoku University

RP Kubota, T (通讯作者), Case Western Reserve Univ, Univ Hosp Cleveland, Med Ctr, Dept Neurol, 11100 Euclid Ave, Cleveland, OH 44106 USA.

EM takafumi.kubota.0612@gmail.com
TC 30

Z9 30

PD JAN

PY 2021

VL 200

AR 106349

DI 10.1016/j.clineuro.2020.106349

EA JAN 2021

WC Clinical Neurology; Surgery

ER

PT J

AU Asadi-Pooya, AA

Shahisavandi, M

Sadeghian, S

Nezafat, A

Nabavizadeh, SA

Barzegar, Z

AF Asadi-Pooya, Ali A.
Shahisavandi, Mina
Sadeghian, Saeid
Nezafat, Abdullah
Nabavizadeh, Seyed Ali
Barzegar, Zohreh
TI Is the risk of COVID-19 contraction increased in patients with epilepsy?
SO EPILEPSY & BEHAVIOR
LA English
DT Article
DE Coronavirus; COVID-19; Epilepsy; Seizure
AB Objective: The aim of the current study was to investigate the rates of contracting COVID-19 in various populations to provide evidence on the susceptibility of patients with epilepsy (PWE) to contracting symptomatic COVID-19.

Methods: We surveyed a random sample of three groups of people: patients with epilepsy, people with psychiatric problems, and a group of the general population. The survey included four general questions (age, sex, education, and medical/psychiatric problem) and four COVID-19 specific questions (contracting COVID-19, relatives with COVID-19, wearing a face mask, and frequent hand washings).

Results: Three hundred and fifty -eight people were surveyed (108 healthy individuals, 154 patients with epilepsy, and 96 patients with psychiatric problems). Thirty-eight (11%) people had a history of COVID-19 contraction. The only factor that had a significant association with COVID-19 contraction was a relative with COVID-19 (Odds Ratio: 5.82; 95% Confidence Interval: 2.85-11.86; p = 0.0001). Having epilepsy did not increase the risk of COVID-19 contraction.

Conclusion: Symptomatic COVID-19 does not seem to be more likely in PWE. The single most important factor associated with contracting COVID-19 is a close relative with this infection. Isolation of people with SARS-CoV-2 infection and observation of their close contacts may reduce the risk of secondary infections. (C) 2020 Elsevier Inc. All rights reserved.
C1 [Asadi-Pooya, Ali A.; Shahisavandi, Mina; Nezafat, Abdullah; Nabavizadeh, Seyed Ali; Barzegar, Zohreh] Shiraz Univ Med Sci, Epilepsy Res Ctr, Shiraz, Iran.

[Asadi-Pooya, Ali A.] Thomas Jefferson Univ, Jefferson Comprehens Epilepsy Ctr, Dept Neurol, Philadelphia, PA USA.

[Sadeghian, Saeid] Ahvaz Jundishapur Univ Med Sci, Golestan Med Educ & Res Ctr, Dept Pediat Neurol, Ahvaz, Iran.
C3 Shiraz University of Medical Science; Jefferson University; Ahvaz Jundishapur University of Medical Sciences (AJUMS)
RP Asadi-Pooya, AA (通讯作者), Shiraz Univ Med Sci, Epilepsy Res Ctr, Shiraz, Iran.
EM aliasadipooya@yahoo.com
TC 3
Z9 3
PD FEB
PY 2021
VL 115
AR 107734
DI 10.1016/j.yebeh.2020.107734

EA JAN 2021
WC Behavioral Sciences; Clinical Neurology; Psychiatry
ER

PT J
AU Gul, ZB
Atakli, HD
AF Gul, Zeynep Bastug
Atakli, Hayrunisa Dilek
TI Effect of the COVID-19 pandemic on drug compliance and stigmatization in patients with epilepsy
SO EPILEPSY & BEHAVIOR
LA English
DT Article
DE COVID-19; Drug compliance; Stigmatization; Epilepsy
ID STIGMA; INTERVENTIONS; ADHERENCE; PEOPLE
AB Objective: Pandemics like coronavirus disease 2019 (COVID-19) bring along many individual and social problems. We aimed to investigate what changes the COVID-19 pandemic can cause in patients with epilepsy on drug compliance and stigmatization.

Material and method: Modified Morisky Scale (MMS) and stigmatization scales were used between October and November 2019 to assess drug compliance and stigmatization in epilepsy patients. These scales were renewed in June and July 2020 in the same patient group to assess the impact of the epidemic on drug compliance and stigmatization in patients with epilepsy. Statistical analysis was performed using the statistical software SPSS 17.0 for Windows (SPSS, Inc). Demographic and clinical characteristics of the patients were recorded in SPSS. The interviews were conducted during the interictal period. Paired samples t-test was used to compare the stigma scale results of epilepsy patients before and during COVID-19. The Wilcoxon test was used to compare MMS groups before and during COVID-19.

Results: A total of 110 patients were included in the study. There was no significant difference between the pre-pandemic and pandemic period in epilepsy stigma scale used to evaluate stigmatization levels in patients. During the pandemic period, it was observed that patients had higher motivation and higher knowledge than before the pandemic ($p = 0.048$). There were seven patients (6.4%) whose seizure frequency increased during the pandemic period. There were two patients (1.8%) who had difficulty in accessing drugs during the pandemic period. In multivariate analysis, only parameter that predicted an increase in seizure frequency was the number of drugs used In the of COVID-19 period. In correlation analysis, a negative correlation was found between the stigma total score during COVID-19 period and education level.

Conclusion: A slight increase in the frequency of seizures was observed in our patients during the pandemic period, and no significant problem was experienced in accessing drugs. The COVID-19 pandemic made patients more motivated and informed in drug compliance in the patient group and had no effect on stigmatization. (C) 2020 Elsevier Inc. All rights reserved.
C1 [Gul, Zeynep Bastug; Atakli, Hayrunisa Dilek] Univ Hlth Sci, Dept Neurol, Bakirkoy Prof Dr Mazhar Osman Training & Res Hosp, Doktor Tevf Saglam Cd 25-2, TR-34147 Istanbul, Turkey.

C3 Istanbul Bakirkoy Mental Health & Neurology Training & Research Hospital
RP Gul, ZB (通讯作者), Univ Hlth Sci, Dept Neurol, Bakirkoy Prof Dr Mazhar Osman Training & Res Hosp, Doktor Tevf Saglam Cd 25-2, TR-34147 Istanbul, Turkey.
EM drzeynep34@hotmail.com
TC 8
Z9 8
PD JAN
PY 2021
VL 114
AR 107610
DI 10.1016/j.yebeh.2020.107610
EA JAN 2021
PN A
WC Behavioral Sciences; Clinical Neurology; Psychiatry
ER

PT J
AU Vancini, RL
de Lira, CAB
Gentil, P
Andrade, MS
AF Vancini, Rodrigo Luiz
Barbosa de Lira, Claudio Andre
Gentil, Paulo
Andrade, Marilia Santos
TI Neurological features of COVID-19 and epilepsy: Could neuromuscular assessment be a physical and functional marker?
SO EPILEPSY & BEHAVIOR
LA English
DT Letter
DE COVID-19; Epilepsy; marker
ID ONE-YEAR OUTCOMES; SURVIVORS; STRENGTH
C1 [Vancini, Rodrigo Luiz] Univ Fed Espirito Santo, Ctr Educ Fis & Desportos, Campus Univ,Ave Fernando Ferrari 514, BR-29075810 Vitoria, ES, Brazil.
[Barbosa de Lira, Claudio Andre; Gentil, Paulo] Univ Fed Goias, Fac Educ Fis & Danca, Lab Avaliacao Movimento Humano, Setor Fisiol Humana & Exercicio, Goiania, Go, Brazil.
[Andrade, Marilia Santos] Univ Fed Sao Paulo, Dept Fisiol, Sao Paulo, Brazil.
C3 Universidade Federal do Espirito Santo; Universidade Federal de Goias; Universidade Federal de Sao Paulo (UNIFESP)
RP Vancini, RL (通讯作者), Univ Fed Espirito Santo, Ctr Educ Fis & Desportos, Campus Univ,Ave Fernando Ferrari 514, BR-29075810 Vitoria, ES, Brazil.
EM rodrigoluizvancini@gmail.com
TC 0
Z9 0
PD JAN
PY 2021
VL 114

AR 107648
DI 10.1016/j.yebeh.2020.107648
EA JAN 2021
PN A
WC Behavioral Sciences; Clinical Neurology; Psychiatry
ER

PT J
AU Bonkowsky, JL
   Felling, RJ
   Grinspan, ZM
   Guerriero, RM
   Kosofsky, BE
   Lyons-Warren, AM
   deVeber, GA
AF Bonkowsky, Joshua L.
   Felling, Ryan J.
   Grinspan, Zachary M.
   Guerriero, Rejean M.
   Kosofsky, Barry E.
   Lyons-Warren, Ariel M.
   deVeber, Gabrielle A.
TI The Pediatric Neurology 2020 Research Workforce Survey: Optimism in a
   Time of Challenge
SO PEDIATRIC NEUROLOGY
LA English
DT Article
DE neurology; Career; Funding; Survey; Workforce; Physician-scientist
AB Background: The past decades have seen a transformational shift in the understanding and treatment for neurological diseases affecting infants and children. These advances have been driven in part by the pediatric neurology physician-scientist workforce and its efforts. However, pediatric neurology research faces substantial challenges from internal and external forces including work-life balance demands, COVID-19 pandemic effects, and research funding. Understanding the impact of these challenges on the perceptions, planning, and careers of pediatric neurology physician-scientists is needed to guide the research mission.
   Methods: Our objective was to survey the research challenges, goals, and priorities of pediatric neurologists. In 2020 we conducted a cross-sectional, 28-question survey emailed to 1,775 members of the Child Neurology Society.
   Results: One hundred fifty-one individuals responded to the survey. Most respondents were grant investigators (52%) and conducted clinical research (69%). Research areas included epilepsy (23%), neurodevelopmental and autism (16%), neurocritical care and stroke (11%), neurogenetics and neurometabolics (9%), neonatal neurology (8%), and others. The most common funding source was the National Institutes of Health (37%). Shared major research concerns were funding, utilization of remote technology, overcoming disparities, natural history and multicenter studies, global neurology, and diversification of the research portfolio. Commitment to continuing and increasing

research efforts was evident.
Conclusions: Our survey demonstrates obstacles for physician-scientist researchers in pediatric neurology, but it also shows optimism about continued opportunity. Creative approaches to address challenges will benefit the research mission, maximize the current and future pool of researchers, and help improve the lives of children with neurological disorders. Crown Copyright (C) 2020 Published by Elsevier Inc. All rights reserved.
C1 [Bonkowsky, Joshua L.] Univ Utah, Sch Med, Dept Pediat, Div Pediat Neurol, Salt Lake City, UT USA.
[Bonkowsky, Joshua L.] Primary Childrens Med Ctr, Intermt Healthcare, Salt Lake City, UT USA.
[Felling, Ryan J.] Johns Hopkins Sch Med, Div Pediat Neurol, Baltimore, MD USA.
[Grinspan, Zachary M.] New York Presbyterian Weill Cornell Med, Dept Populat Hlth Sci, New York, NY USA.
[Grinspan, Zachary M.; Kosofsky, Barry E.] New York Presbyterian Weill Cornell Med, Dept Pediat, New York, NY USA.
[Guerriero, Rejean M.] Washington Univ, Sch Med, Dept Neurol, Div Pediat & Dev Neurol, St Louis, MO 63110 USA.
[Lyons-Warren, Ariel M.] Texas Childrens Hosp, Baylor Coll Med, Div Child Neurol, Dept Pediat, Houston, TX 77030 USA.
[deVeber, Gabrielle A.] Hosp Sick Children, Div Neurol, Toronto, ON, Canada.
C3 Utah System of Higher Education; University of Utah; Intermountain Healthcare; Intermountain Medical Center; Johns Hopkins University; Johns Hopkins Medicine; Washington University (WUSTL); Baylor College of Medicine; University of Toronto; Hospital for Sick Children (SickKids)
RP deVeber, GA (通讯作者), Hosp Sick Children, Toronto, ON, Canada.
EM gabrielle.deveber@sickkids.ca
TC 1
Z9 1
PD MAR
PY 2021
VL 116
BP 62
EP 67
DI 10.1016/j.pediatrneurol.2020.11.020
EA JAN 2021
WC Clinical Neurology; Pediatrics
ER

PT J
AU Kuroda, N
AF Kuroda, Naoto
TI Epilepsy and COVID-19: Updated evidence and narrative review
SO EPILEPSY & BEHAVIOR
LA English
DT Review
DE Epilepsy; COVID-19; Antiepileptic drug; surgery; Neurological disease

ID INFECTION; EDUCATION; CHILDREN; EEG
AB The coronavirus disease 2019 (COVID-19) outbreak started in Wuhan, China, in late 2019 and rapidly spread globally. Vaccines have recently been developed and are being administered in some countries, but their widespread use is not yet sufficient; the battle against COVID-19 is protracted and people need to adapt to living under the influence of this disease. Epilepsy is a common chronic neurological condition characterized by spontaneous recurrence of unprovoked seizures. Various effects of COVID-19 on epilepsy have been studied in recent months. As clinicians, we need to keep up with daily updates in the evidence regarding interactions between COVID-19 and epilepsy. This review article summarizes the current evidence. Prospective studies on epilepsy and COVID-19 remain lacking. Most articles have comprised case reports, case series, retrospective studies, and recommendations/opinions that do not include data. However, summarizing these articles can identify the demands for research into COVID-19 and epilepsy by clarifying what is known and what remains unclear from current research.
(C) 2021 Elsevier Inc. All rights reserved.
C1 [Kuroda, Naoto] Wayne State Univ, Dept Pediat, 3901 Beaubien St, Detroit, MI 48201 USA.
[Kuroda, Naoto] Tohoku Univ, Dept Epileptol, Sch Med, Sendai, Miyagi, Japan.
C3 Wayne State University; Tohoku University
RP Kuroda, N (通讯作者), Wayne State Univ, Dept Pediat, 3901 Beaubien St, Detroit, MI 48201 USA.
EM naoto.kuroda@wayne.edu
TC 14
Z9 14
PD MAR
PY 2021
VL 116
AR 107785
DI 10.1016/j.yebeh.2021.107785
EA JAN 2021
WC Behavioral Sciences; Clinical Neurology; Psychiatry
ER

PT J
AU Wouk, J
Rechenchoski, DZ
Rodrigues, BCD
Ribelato, EV
Faccin-Galhardi, LC
AF Wouk, Jessica
Rechenchoski, Daniele Zendrini
Rodrigues, Bianca Cerqueira Dias
Ribelato, Elisa Vicente
Faccin-Galhardi, Ligia Carla
TI Viral infections and their relationship to neurological disorders
SO ARCHIVES OF VIROLOGY
LA English

DT Review
DE Epilepsy; COVID-19; Neurological disease; SARS-CoV-2
ID GUILLAIN-BARRE-SYNDROME; CENTRAL-NERVOUS-SYSTEM; INFLUENZA-A VIRUS; ALZHEIMERS-DISEASE; PARKINSONS-DISEASE; MULTIPLE-SCLEROSIS; NEURODEGENERATIVE DISEASES; HUMAN CYTOMEGALOVIRUS; HERPES; RISK
AB The chronic dysfunction of neuronal cells, both central and peripheral, a characteristic of neurological disorders, may be caused by irreversible damage and cell death. In 2016, more than 276 million cases of neurological disorders were reported worldwide. Moreover, neurological disorders are the second leading cause of death. Generally, the etiology of neurological diseases is not fully understood. Recent studies have related the onset of neurological disorders to viral infections, which may cause neurological symptoms or lead to immune responses that trigger these pathological signs. Currently, this relationship is mostly based on epidemiological data on infections and seroprevalence of patients who present with neurological disorders. The number of studies aiming to elucidate the mechanism of action by which viral infections may directly or indirectly contribute to the development of neurological disorders has been increasing over the years but these studies are still scarce. Comprehending the pathogenesis of these diseases and exploring novel theories may favor the development of new strategies for diagnosis and therapy in the future. Therefore, the objective of the present study was to review the main pieces of evidence for the relationship between viral infection and neurological disorders such as Alzheimer's disease, Parkinson's disease, Guillain-Barre syndrome, multiple sclerosis, and epilepsy. Viruses belonging to the families Herpesviridae, Orthomyxoviridae, Flaviviridae, and Retroviridae have been reported to be involved in one or more of these conditions. Also, neurological symptoms and the future impact of infection with SARS-CoV-2, a member of the family Coronaviridae that is responsible for the COVID-19 pandemic that started in late 2019, are reported and discussed.
C1 [Wouk, Jessica] Midwest State Univ, Postgrad Program Pharmaceut Sci, CEDETEG Campus, Guarapuava, Parana, Brazil.
[Rechenchoski, Daniele Zendrini; Rodrigues, Bianca Cerqueira Dias; Ribelato, Elisa Vicente; Faccin-Galhardi, Ligia Carla] Univ Estadual Londrina, Dept Microbiol, Biol Sci Ctr, Londrina, Parana, Brazil.
C3 Universidade Estadual de Londrina
RP Faccin-Galhardi, LC (通讯作者)，Univ Estadual Londrina, Dept Microbiol, Biol Sci Ctr, Londrina, Parana, Brazil.
EM lgalhardi@uel.br
TC 14
Z9 14
PD MAR
PY 2021
VL 166
IS 3
BP 733
EP 753
DI 10.1007/s00705-021-04959-6
EA JAN 2021
WC Virology

ER

PT J
AU Reilly, C
Muggeridge, A
Cross, JH
AF Reilly, Colin
Muggeridge, Amy
Cross, J. Helen
TI The perceived impact of COVID-19 and associated restrictions on young people with epilepsy in the UK: Young people and caregiver survey
SO SEIZURE-EUROPEAN JOURNAL OF EPILEPSY
LA English
DT Article
DE COVID-19; Epilepsy; Behaviour; Sleep
ID CHILDREN; STRESS
AB Purpose: To garner the views of young people with epilepsy and caregivers regarding the impact of COVID-19 and subsequent restrictions in the UK.

Mehods: An online survey was used to explore the views of young people with epilepsy (n = 71) and caregivers (n = 130) in June 2020. It included questions on the impact of the pandemic and associated restrictions on the child's epilepsy and on child and parental wellbeing.

Results: One in three young people and 29 % of caregivers reported that the young person's seizures had increased during the pandemic (only 10 % of young people and 8% of caregivers reported a decrease). Half of young people reported that they were more reluctant to go to hospital. Thirty-one percent of young people and 20 % of parents reported difficulties getting epilepsy medication whilst a significant minority of young people (18 %) and caregivers (25 %) reported that the young person had investigations/assessments cancelled by their hospital. The majority of young people reported their sleep (72 %), mood (64 %) and levels of physical activity (53 %) had deteriorated. Caregivers experienced increases in stress (55 %) and anxiety (52 %). Epilepsy nurses were seen as the most helpful support

Conclusions: Results indicate that the pandemic and associated restrictions have had a negative impact on young people with epilepsy. Perceived increases in seizures, reluctance to go to hospital and cancelled investigations are likely to impact on epilepsy management. The wider psychosocial impact is also likely to be significant with increases in child and caregiver mental health problems in an already vulnerable group.
C1 [Reilly, Colin; Muggeridge, Amy; Cross, J. Helen] Young Epilepsy, Res Dept, Lingfield RH7 6PW, Surrey, England.

[Reilly, Colin; Cross, J. Helen] UCL Great Ormond St Inst Child Hlth ICH, 30 Guilford St, London WC1N 1EH, England.

[Cross, J. Helen] Great Ormond St Hosp Sick Children, Great Ormond St, London WC1N 3JH, England.
C3 University of London; University College London; University of London; University College London; Great Ormond Street Hospital for Children NHS Foundation Trust

RP Reilly, C (通讯作者), Young Epilepsy, Res Dept, Lingfield RH7 6PW, Surrey, England.; Reilly, C (通讯作者), UCL Great Ormond St Inst Child Hlth ICH, 30 Guilford St, London WC1N 1EH, England.
EM creilly@youngepilepsy.org.uk
TC 9
Z9 9
PD FEB
PY 2021
VL 85
BP 111
EP 114
DI 10.1016/j.seizure.2020.12.024
WC Clinical Neurology; Neurosciences
ER

PT J
AU Giordano, A
Siciliano, M
De Micco, R
Sant'Elia, V
Russo, A
Tedeschi, G
Tessitore, A
AF Giordano, Alfonso
Siciliano, Mattia
De Micco, Rosa
Sant'Elia, Valeria
Russo, Antonio
Tedeschi, Gioacchino
Tessitore, Alessandro
TI Correlates of psychological distress in epileptic patients during the COVID-19 outbreak
SO EPILEPSY & BEHAVIOR
LA English
DT Article
DE Objective major life events; COVID-19; Psychological distress; Epilepsy
ID SEIZURES; ANXIETY; STRESS
AB Introduction: Following the severe consequences of the coronavirus disease 2019 (COVID-19) outbreak, on March 9th, 2020 the Italian Government implemented extraordinary measures to limit viral transmission, including restrictive quarantine measures. Psychological distress represents the seizure-precipitating factor most often reported by patients with epilepsy. To date, no studies have analyzed the role played by the different dimensions of psychological distress quarantine-induced in patients with epilepsy.

Materials and Methods: We included a total of 40 patients, 18 suffered from generalized, and 22 from focal epilepsy. The patients previously seen in the outpatient clinic during the pre-lockdown period between January and February 2020 were

reevaluated after the lockdown period. Psychological distress was evaluated by using the three subscales of Impact of Event Scale-Revised (IES-R). Finally, we employed logistic regression analyses to explore the demographic and clinical features associated to high scores on IES-R.
Results: Patients with higher scores on IES-R Intrusion and IES-R Avoidance subscales demonstrated an increased number of epileptic attacks compared to prelockdown period. Multivariate logistic regression analyses showed that a specific subgroup of patients (i.e., older, female with more anxious symptoms) are at higher risk of increased seizure frequency.
Conclusions: Our study confirmed that the frequency of epileptic seizures increased during lockdown when compared to pre-lockdown period. The early identification of patients more vulnerable to worsening is crucial to limit the risk of requiring hospital or clinical treatment during the COVID-19 outbreak. (C) 2020 Elsevier Inc. All rights reserved.
C1 [Giordano, Alfonso; Siciliano, Mattia; De Micco, Rosa; Sant'Elia, Valeria; Russo, Antonio; Tedeschi, Gioacchino; Tessitore, Alessandro] Univ Campania Luigi Vanvitelli, MRI Res Ctr Vanvitelli FISM, Dept Adv Med & Surg Sci, Piazza Miraglia 2, I-80138 Naples, Italy.
[Siciliano, Mattia] Univ Campania Luigi Vanvitelli, Dept Psychol, Viale Ellittico 31, I-81100 Caserta, Italy.
C3 Universita della Campania Vanvitelli; Universita della Campania Vanvitelli
RP Giordano, A (通讯作者), Univ Campania Luigi Vanvitelli, MRI Res Ctr Vanvitelli FISM, Dept Adv Med & Surg Sci, Piazza Miraglia 2, I-80138 Naples, Italy.
EM alfonso.giordano@unicampania.it
TC 7
Z9 7
PD FEB
PY 2021
VL 115
AR 107632
DI 10.1016/j.yebeh.2020.107632
WC Behavioral Sciences; Clinical Neurology; Psychiatry
ER

PT J
AU Datta, P
Barrett, W
Bentzinger, M
Jasinski, T
Jayagopal, LA
Mahoney, A
Pearon, C
Swaminathan, A
Vuppala, A
Samson, KK
Wang, HM

   Taraschenko, O
AF Datta, Proleta
   Barrett, Wattana
   Bentzinger, Monica
   Jasinski, Tracy
   Jayagopal, Lakshman Arcot
   Mahoney, Alexa
   Pearon, Crystal
   Swaminathan, Arun
   Vuppala, Aditya
   Samson, Kaeli K.
   Wang, Hongmei
   Taraschenko, Olga
TI Ambulatory care for epilepsy via telemedicine during the COVID-19
   pandemic
SO EPILEPSY & BEHAVIOR
LA English
DT Article
DE COVID-19; Pandemic; Telemedicine; Healthcare access; care
AB Objective: To assess feasibility, patient satisfaction, and financial advantages of telemedicine for epilepsy ambulatory care during the current COVID-19 pandemic.

Methods: The demographic and clinical characteristics of all consecutive patients evaluated via telemedicine at a level 4 epilepsy center between March 20 and April 20, 2020 were obtained retrospectively from electronic medical records. A telephone survey to assess patient satisfaction and preferences was conducted within one month following the initial visit.

Results: Among 223 telehealth patients, 85.7% used both synchronous audio and video technology. During the visits, 39% of patients had their anticonvulsants adjusted while 18.8% and 11.2% were referred to laboratory/diagnostic testing and specialty consults, respectively. In a post-visit survey, the highest degree of satisfaction with care was expressed by 76.9% of patients. The degree of satisfaction tended to increase the further a patient lived from the clinic ($p = 0.05$). Beyond the pandemic, 89% of patients reported a preference for continuing telemedicine if their epilepsy symptoms remained stable, while only 44.4% chose telemedicine should their symptoms worsen. Inclement weather and lack of transportation were factors favoring continued use of telemedicine. An estimated cost saving to patient attributed to telemedicine was $30.20 +/- 3.8 per visit.

Significance: Our findings suggest that epilepsy care via telemedicine provided high satisfaction and economic benefit, without compromising patients' quality of care, thereby supporting the use of virtual care during current and future epidemiological fallouts. Beyond the current pandemic, patients with stable seizure symptoms may prefer to use telemedicine for their epilepsy care. (C) 2020 Elsevier Inc. All rights reserved.
C1 [Datta, Proleta; Barrett, Wattana; Jasinski, Tracy; Jayagopal, Lakshman Arcot; Swaminathan, Arun; Vuppala, Aditya; Taraschenko, Olga] Univ Nebraska Med Ctr, Dept Neurol Sci, Omaha, NE 68198 USA.

[Bentzinger, Monica; Mahoney, Alexa; Pearon, Crystal] Nebraska Med Hosp, Omaha, NE USA.

[Samson, Kaeli K.] Univ Nebraska Med Ctr, Dept Biostat, Omaha, NE 68198 USA.
[Wang, Hongmei] Univ Nebraska Med Ctr, Dept Hlth Serv Res & Adm, Omaha, NE 68198 USA.
C3 University of Nebraska System; University of Nebraska Medical Center; University of Nebraska System; University of Nebraska Medical Center; University of Nebraska System; University of Nebraska Medical Center
RP Datta, P (通讯作者), Univ Nebraska Med Ctr, 988435 Nebraska Med Ctr, Omaha, NE 68198 USA.
EM proleta.datta@unmc.edu
TC 8
Z9 9
PD MAR
PY 2021
VL 116
AR 107740
DI 10.1016/j.yebeh.2020.107740
EA FEB 2021
WC Behavioral Sciences; Clinical Neurology; Psychiatry
ER

PT J
AU Friedrich, L
Sruk, A
Bielen, I
AF Friedrich, Latica
Sruk, Ana
Bielen, Ivan
TI Responses of people with epilepsy to the COVID-19 pandemic in the time of national lockdown
SO EPILEPSY & BEHAVIOR
LA English
DT Article
DE Epilepsy; COVID-19; Lockdown; Pandemic; Telemedicine
AB The aim of our study was to gather information on how people with epilepsy (PwE) responded to the COVID-19 pandemic during the national lockdown. An online questionnaire was therefore offered to the visitors of the Croatian Association for Epilepsy's website. The 22-items questionnaire was designed to acquire information from adults with epilepsy living in Croatia on demographic data, cognitive, emotional and behavioral responses to the pandemic, and communication problems between patients and their neurologists during the lockdown. Perceived anxiety and fears were expressed with the Likert scale (1-5) and the results of specific fears added to make the Total Fear Score. Results: Out of 186 respondents in total, only 2.8% did not comply with the lockdown measures, and all of those respondents stated that they did not feel any anxiety related to COVID-19. A canceled neurologist examination during the lockdown was significantly associated with pandemic-related anxiety (2.9 +/- 1.28 vs. 2.3 +/- 1.19, U = 3039, p = 0.001) and fears (Total Fear Score 31.4 +/- 9.70 vs. 28.4 +/- 9.79, U = 3341, p = 0.036), and 87.4% of respondents expressed the wish to communicate with

their neurologist, either by phone/video call (53.0%) or email (34.4%). Conclusion: We think the results of our survey show that the responses from PwE point to a social responsibility appropriate for the existing situation. During future pandemics, telemedicine could have an important role in tackling the fears and anxieties caused by the cancelation of examinations, which corresponds to the wishes expressed by the great majority of our respondents. (C) 2021 Elsevier Inc. All rights reserved.
C1 [Friedrich, Latica; Sruk, Ana; Bielen, Ivan] Univ Osijek, Sveti Duh Univ Hosp, Sch Med Josip Juraj Strossmayer, Dept Neurol, Sveti Duh 64, Zagreb, Croatia.
C3 University of JJ Strossmayer Osijek
RP Friedrich, L (通讯作者), Univ Osijek, Sveti Duh Univ Hosp, Sch Med Josip Juraj Strossmayer, Dept Neurol, Sveti Duh 64, Zagreb, Croatia.
EM latica@kbsd.hr
TC 3
Z9 3
PD MAR
PY 2021
VL 116
AR 107790
DI 10.1016/j.yebeh.2021.107790
EA FEB 2021
WC Behavioral Sciences; Clinical Neurology; Psychiatry
ER

PT J
AU Sun, MX
Ruan, XY
Li, YY
Wang, P
Zheng, SS
Shui, GY
Li, L
Huang, Y
Zhang, HM
AF Sun, Minxian
Ruan, Xiaoyun
Li, Yuanyuan
Wang, Pei
Zheng, Shasha
Shui, Guiying
Li, Li
Huang, Yan
Zhang, Hongmei
TI Clinical characteristics of 30 COVID-19 patients with epilepsy: A retrospective study in Wuhan
SO INTERNATIONAL JOURNAL OF INFECTIOUS DISEASES
LA English
DT Article

DE COVID-19; Epilepsy; Seizure; New-Onset; Recurrent; Complication;
Clinical outcomes

AB Objective: This study aims to present the clinical characteristics of 30 hospitalized cases with epileptic seizures and coronavirus disease 2019(COVID-19).

Methods: This is a retrospective observational research study. Clinical data were extracted from electronic medical records in 1550 patients with a laboratory-confirmed diagnosis of COVID-19, who were hospitalized in Wuhan Central Hospital, China, from 1 January to 31 April 2020. 30 COVID-19 patients with the diagnosis of epilepsy were enrolled. The clinical characteristics, complications, treatments, and clinical outcomes of 30 cases were collected and analyzed.

Result: Of 30 patients with a diagnosis of epilepsy and COVID-19, 13 patients (43.4%) had new-onset epileptic seizures without an epilepsy history(new-onset seizure group, NS group), ten patients(33.3%) had an epilepsy history with a recurrent epileptic seizure (recurrent seizure group, RS group) and seven patients(23.3%) had an epilepsy history but no seizure during the course of COVID-19 (epilepsy history group, EH group). Patients in the RS group had a larger number of other-neurological-disease histories than those in the NS and EH groups (7/10[70%] VS 1/13 [7.7%] VS 1/7[14.3%]); the difference between the RS group and NS group is significant ($P < 0.05$). Patients in the NE and RS groups suffered more severe/critical COVID-19 infection than patients in the EH group (10/13[76.9%] VS 6/10[60%] VS 1/7[14.3%]); the difference between the NS group and EH group is significant ($P < 0.05$). 36.7% of patients had one to five neurological complications, and 46.4% of patients had 6-10 neurological complications. The complications in patients with seizures (in the RS and NS groups) seem to be more than those without seizures (in the EH group), but it did not reach statistical significance. The proportion of antiepileptic drugs (AEDs) treatment before admission was higher in the EH group than in the RE group(7/7 [100%] VS 2/10 [20%], $P < 0.05$). The mortality of 30 patients with epilepsy and COVID-19 was 36.67%. The mortality of the NS group(38.5%) and the RS group(50%) were a little higher than in the EH group(14.3%). None of the convalescent patients had a recurrent seizure, and there were no more deaths in the 3-month followup after discharge.

Conclusions: COVID-19 patients with recurrent epileptic seizures had more underlying neurological diseases than patients who had an epilepsy history but without a seizure. Patients with new-onset and recurrent epileptic seizures suffered more severe/critical COVID-19, which may lead to a worse prognosis. If patients with epilepsy history continue using AEDs during COVID-19 pandemics, the risk of recurrent seizure may be reduced, and a good prognosis for patients with epilepsy history could be expected.
(C) 2020 The Author(s). Published by Elsevier Ltd on behalf of International Society for Infectious Diseases.
C1 [Sun, Minxian; Wang, Pei; Zheng, Shasha; Shui, Guiying; Li, Li; Zhang, Hongmei] Huazhong Univ Sci & Technol, Cent Hosp Wuhan, Tongji Med Coll, Dept Endocrinol, Wuhan 430021, Hubei, Peoples R China.

[Ruan, Xiaoyun; Li, Yuanyuan] Huazhong Univ Sci & Technol, Cent Hosp Wuhan, Tongji Med Coll, Dept Pharm, Wuhan 430021, Hubei, Peoples R China.

[Huang, Yan] Huazhong Univ Sci & Technol, Cent Hosp Wuhan, Tongji Med Coll, Dept Bidding, Wuhan 430021, Hubei, Peoples R China.

C3 Huazhong University of Science & Technology; Huazhong University of
Science & Technology; Huazhong University of Science & Technology

RP Zhang, HM (通讯作者), Huazhong Univ Sci & Technol, Cent Hosp Wuhan, Tongji Med Coll, Dept Endocrinol, Wuhan 430021, Hubei, Peoples R China.; Huang, Y (通讯作者), Huazhong Univ Sci & Technol, Cent Hosp Wuhan, Tongji Med Coll, Dept Bidding, Wuhan 430021, Hubei, Peoples R China.
EM 1700164748@qq.com; zhm7001@163.com
TC 7
Z9 8
PD FEB
PY 2021
VL 103
BP 647
EP 653
DI 10.1016/j.ijid.2020.09.1475
EA FEB 2021
WC Infectious Diseases
ER

PT J
AU Millevert, C
   Van Hees, S
   Fodjo, JNS
   Wijtvliet, V
   Villela, EFD
   Rosso, B
   Gil-Nagel, A
   Weckhuysen, S
   Colebunders, R
AF Millevert, Charissa
   Van Hees, Stijn
   Fodjo, Joseph Nelson Siewe
   Wijtvliet, Veerle
   de Moura Villela, Edlaine Faria
   Rosso, Barbara
   Gil-Nagel, Antonio
   Weckhuysen, Sarah
   Colebunders, Robert
TI Impact of COVID-19 on the lives and psychosocial well-being of persons with epilepsy during the third trimester of the pandemic: Results from an international, online survey
SO EPILEPSY & BEHAVIOR
LA English
DT Article
DE Epilepsy; COVID-19; Mental health; HADS; Telemedicine
ID GENERAL-POPULATION; DEPRESSION; ANXIETY; CARE
AB Objective: To evaluate the impact of the coronavirus disease 2019 (COVID-19) measures on the lives and psychosocial well-being of persons with epilepsy (PWE) during the third trimester of the COVID-19 pandemic.

Methods: A structured questionnaire investigating different aspects of the lives and psychosocial well-being of PWE during the COVID-19 pandemic was developed. Persons with epilepsy were invited via social media to anonymously respond to a secure web-based online questionnaire(www. icpcovid. com). Responses were collected between July 26th and December 3rd, 2020. Hospital anxiety and depression scales (HADS) were used to screen respondents for depression (HADS-D) and anxiety (HADS-A).

Results: Responses of 407 PWE were included in the analysis; 304 (74.7%) respondents were female and 245 (60.2%) living in Europe, 157 (38.6%) in South America, and 5 (1.2%) in Canada. Seventy-six (18.7%) reported a decrease of income during the COVID-19 lockdown, and 122 (30.0%) experienced difficulties in obtaining anti-seizure medication (ASM), mostly (72/122, 59.0%) due to unavailability. Seizure frequency increased in 122 (30.0%); 295 (72.5%) screened positive for anxiety, and 159 (39.1%) for depression. Hundred eighty-eight (46.2%) reported reluctance to seek medical care; 27.3% believed that epilepsy was associated with an increased risk of COVID-19 disease. Forty-six (74.2%) of 62 PWE who were followed up by telephone or video consult were satisfied with this consult. Fifty-five respondents, most (89.1%) of whom were from Europe, had also participated in a previous survey during the early months of the pandemic. In this subgroup, although there was no difference in prevalence of a positive screening for depression or anxiety, mean scores on HADS-A and HADS-D increased from 6.65 +/- 3.99 to 7.27 +/- 4.01 (p = 0.418), and from 5.84 +/- 4.43 to 6.60 +/- 4.45 (p = 0.371), respectively.

Conclusions: The COVID-19 pandemic continues to impact the psychosocial and somatic well-being of PWE. To minimize this impact, ensuring uninterrupted access to ASM is essential. Teleconsultations are valid alternatives for continued follow-up, but should include attention to psychosocial well-being. Persons with epilepsy should be more actively informed that epilepsy is not a risk factor for developing (more severe) COVID-19 disease. (C) 2021 Elsevier Inc. All rights reserved.
C1 [Millevert, Charissa; Weckhuysen, Sarah] Univ Hosp Antwerp, Dept Neurol, Antwerp, Belgium.

[Millevert, Charissa; Weckhuysen, Sarah] Univ Antwerp, VIB Ctr Mol Neurol, VIB, Antwerp, Belgium.

[Van Hees, Stijn; Fodjo, Joseph Nelson Siewe; Colebunders, Robert] Univ Antwerp, Global Hlth Inst, Antwerp, Belgium.

[Van Hees, Stijn; Wijtvliet, Veerle] Univ Antwerp, Lab Expt Med & Pediat, Antwerp, Belgium.

[de Moura Villela, Edlaine Faria] Fed Univ Jatai, Sch Med, Hlth Sci Unit, Goiania, Go, Brazil.

[Rosso, Barbara] Italian Hosp Buenos Aires, Dept Neurol, Buenos Aires, DF, Argentina.

[Gil-Nagel, Antonio] Hosp Ruber Int, Dept Neurol, Madrid, Spain.

C3 University of Antwerp; Flanders Institute for Biotechnology (VIB);
University of Antwerp; University of Antwerp; University of Antwerp;
Hospital Italiano de Buenos Aires; University of Buenos Aires

RP Colebunders, R (通讯作者), Univ Antwerp, Gouverneur Kinsbergen Ctr, Global Hlth Inst, Doornstr 331, B-2610 Antwerp, Belgium.

EM Robert.colebunders@uantwerpen.be

TC 9

Z9 9
PD MAR
PY 2021
VL 116
AR 107800
DI 10.1016/j.yebeh.2021.107800
EA FEB 2021
WC Behavioral Sciences; Clinical Neurology; Psychiatry
ER

PT J
AU Abokalawa, F
Ahmad, SF
Al-Hashel, J
Hassan, AM
Arabi, M
AF Abokalawa, Fathi
Ahmad, Samar Farouk
Al-Hashel, Jasem
Hassan, Ahmed Medhat
Arabi, Maher
TI The effects of coronavirus disease 2019 (COVID-19) pandemic on people
with epilepsy (PwE): an online survey-based study
SO ACTA NEUROLOGICA BELGICA
LA English
DT Article
DE Epilepsy; COVID-19; Pandemic; Seizure worsening; Sleep; Stress
ID SLEEP QUALITY; STRESS
AB During the unprecedented COVID-19 pandemic in 2020, the whole world faced an unusual health emergency. Medical care of chronic neurological diseases, such as Epilepsy, is being neglected. In this survey, we aimed to evaluate the impact of the COVID-19 pandemic on the care of people with Epilepsy (PwE) and to identify their risk factors for seizure worsening to direct better future medical care. We administered a web-based survey (submitted on August 5, 2020). It included socio-demographic, Epilepsy-related, and psychometric data (The Depression, Anxiety, and Stress Scale-21 Items(DASS21) and The Pittsburgh Sleep Quality Index (PSQI). Regression analysis identified predictors of seizure worsening. We collected responses from an online survey of PwE during the pandemic. Out of 151 responders, 71 patients complained of issues related to Epilepsy management and all of whom reached the treating physician and solved their problems. Sleep quality was compromised in 84 patients (55.6%). Two-thirds of the patients in our cohort (66.2%) reported depression, 72.2% reported anxiety, and 75.5% reported stress. Eight patients (5.3%) got COVID-19 infection, and only one patient suffered from mild worsening of the seizure. The main concerns were shortage of medications for 46 (30.5%) patients, getting Coronavirus infection for 67 (44.4%) patients, and seizure worsening for 32 (21.3%) patients. Thirty-five patients (23.2%) reported seizure worsening, which was best explained by retirement or jobless state, having moderate or severe stress, poor sleep quality, vagus nerve stimulation (VNS), fear of getting

COVID-19 infection, fear of worsening of seizures, or shortage of medication. During the current COVID-19 pandemic, a significant percentage of PwE experienced worsening of their seizures. This unusual, challenging experience clarifies the urgent need to establish telemedicine services and home-based management of Epilepsy, including ambulatory EEG, home video, and medication delivery to patients' homes to provide continuous medical care.
C1 [Abokalawa, Fathi; Ahmad, Samar Farouk; Al-Hashel, Jasem; Hassan, Ahmed Medhat; Arabi, Maher] Ibn Sina Hosp, Dept Neurol, Sabah Med Area, Kuwait, Kuwait.
[Ahmad, Samar Farouk] Minia Univ, Dept Neurol, Al Minya, Egypt.
[Al-Hashel, Jasem] Kuwait Univ, Hlth Sci Ctr, Dept Med, Kuwait, Kuwait.
C3 Egyptian Knowledge Bank (EKB); Minia University; Kuwait University
RP Abokalawa, F (通讯作者), Ibn Sina Hosp, Dept Neurol, Sabah Med Area, Kuwait, Kuwait.
EM dr_fathi2010@yahoo.com
TC 7
Z9 7
PD FEB
PY 2022
VL 122
IS 1
BP 59
EP 66
DI 10.1007/s13760-021-01609-1
EA FEB 2021
WC Clinical Neurology; Neurosciences
ER

PT J
AU Balestrini, S
Koepp, MJ
Gandhi, S
Rickman, HM
Shin, GY
Houlihan, CF
Anders-Cannon, J
Silvennoinen, K
Xiao, FL
Zagaglia, S
Hudgell, K
Ziomek, M
Haimes, P
Sampson, A
Parker, A
Cross, JH
Pardington, R
Nastouli, E
Swanton, C
Aitken, J

Allen, Z
Ambler, R
Ambrose, K
Ashton, E
Avola, A
Balakrishnan, S
Barns-Jenkins, C
Barr, G
Barrell, S
Basu, S
Beale, R
Beesley, C
Bhardwaj, N
Bibi, S
Bineva-Todd, G
Biswas, D
Blackman, MJ
Bonnet, D
Bowker, F
Broncel, M
Brooks, C
Buck, MD
Buckton, A
Budd, T
Burrell, A
Busby, L
Bussi, C
Butterworth, S
Byott, M
Byrne, F
Byrne, R
Caidan, S
Campbell, J
Canton, J
Cardoso, A
Carter, N
Carvalho, L
Carzaniga, R
Chandler, N
Chen, Q
Cherepanov, P
Churchward, L
Clark, G
Clayton, B
Gigli, CC
Collins, Z
Cottrell, S

Crawford, M
Cubitt, L
Cullup, T
Davies, H
Davis, P
Davison, D
Dearing, V
Debaisieux, S
Diaz-Romero, M
Dibbs, A
Diring, J
Driscoll, PC
D'Avola, A
Earl, C
Edwards, A
Ekin, C
Evangelopoulos, D
Faraway, R
Fearns, A
Ferron, A
Fidanis, E
Fitz, D
Fleming, J
Frampton, D
Frederico, B
Gaiba, A
Gait, A
Gamblin, S
Gartner, K
Gaul, L
Golding, HM
Goldman, J
Goldstone, R
Dominguez, BG
Gong, H
Grant, PR
Greco, M
Grobler, M
Guedan, A
Gutierrez, MG
Hackett, F
Hall, R
Halldorsson, S
Harris, S
Hashim, S
Hatipoglu, E
Healy, L

Heaney, J
Herbst, S
Hewitt, G
Higgins, T
Hindmarsh, S
Hirani, R
Hope, J
Horton, E
Hoskins, B
Howell, M
Howitt, L
Hoyle, J
Htun, MR
Hubank, M
Encabo, HH
Hughes, D
Hughes, J
Huseynova, A
Hwang, MS
Instrell, R
Jackson, D
Jamal-Hanjani, M
Jenkins, L
Jiang, M
Johnson, M
Jones, L
Kanu, N
Kassiotis, G
Kelly, G
Kiely, L
Teixeira, AKS
Kirk, S
Kjaer, S
Knuepfer, E
Komarov, N
Kotzampaltiris, P
Kousis, K
Krylova, T
Kucharska, A
Labrum, R
Lambe, C
Lappin, M
Lee, SA
Levett, A
Levett, L
Levi, M
Liu, HW

Loughlin, S
Lu, WT
MacRae, JI
Madoo, A
Marczak, JA
Martensson, M
Martinez, T
Marzook, B
Matthews, J
Matz, JM
McCall, S
McCoy, LE
McKay, F
McNamara, EC
Minutti, CM
Mistry, G
Molina-Arcas, M
Montaner, B
Montgomery, K
Moore, C
Moore, D
Moraiti, A
Moreira-Teixeira, L
Mukherjee, J
Naceur-Lombardelli, C
Nelson, A
Nicod, J
Nightingale, L
Nofal, S
Nurse, P
Nutan, S
Oedekoven, C
O'Garra, A
O'Leary, JD
Olsen, J
O'Neill, O
O'Reilly, N
Suarez, PO
Osborne, N
Pabari, A
Pajak, A
Papayannopoulos, V
Paraskevopoulou, SM
Patel, N
Patel, Y
Paun, O
Peat, N

Castano, LPB
Caballero, AP
Perez-Lloret, J
Perrault, MS
Perrin, A
Poh, R
Poirier, EZ
Polke, JM
Pollitt, M
Prieto-Godino, L
Proust, A
Puvirajasinghe, C
Queval, C
Ramachandran, V
Ramaprasad, A
Ratcliffe, P
Reed, L
Sousa, CRE
Richardson, K
Ridewood, S
Roberts, F
Roberts, R
Rodgers, A
Clavijo, PR
Rosa, A
Rossi, A
Roustan, C
Rowan, A
Sahai, E
Sait, A
Sala, K
Sanchez, E
Sanderson, T
Santucci, P
Sardar, F
Sateriale, A
Saunders, JA
Sawyer, C
Schlott, A
Schweighoffer, E
Segura-Bayona, S
Punatar, RS
Shahmanesh, M
Shaw, J
Dos Santos, MS
Silvestre, M
Singer, M

Snell, DM
Song, OR
Spyer, MJ
Steel, L
Strange, A
Sullivan, AE
Tan, MSY
Tautz-Davis, ZH
Taylor, E
Taylor, G
Taylor, HB
Taylor-Beadling, A
Subtil, FT
Torras, BT
Toolan-Kerr, P
Torelli, F
Toteva, T
Treeck, M
Trojer, H
Tsai, MHC
Turner, JMA
Turner, M
Ule, J
Ulferts, R
Vanloo, SP
Veeriah, S
Venkatesan, S
Vousden, K
Wack, A
Walder, C
Walker, PA
Wang, YR
Ward, S
Wenman, C
Williams, L
Williams, MJ
Wong, WK
Wright, J
Wu, M
Wynne, L
Xiang, Z
Yap, M
Zagalak, JA
Zecchin, D
Zillwood, R
Sander, JW
Sisodiya, SM

Carthiyaniamma, S
DeTisi, J
Dick, J
Hill, A
Kipper, K
Kullar, B
Norris, S
Rugg-Gunn, F
Salvatierra, R
Shaya, G
Sloan, A
Singh, P
Varley, J
Whatley, B
AF Balestrini, Simona
Koepp, Matthias J.
Gandhi, Sonia
Rickman, Hannah M.
Shin, Gee Yen
Houlihan, Catherine F.
Anders-Cannon, Jonny
Silvennoinen, Katri
Xiao, Fenglai
Zagaglia, Sara
Hudgell, Kirsty
Ziomek, Mariusz
Haimes, Paul
Sampson, Adam
Parker, Annie
Cross, J. Helen
Pardington, Rosemarie
Nastouli, Eleni
Swanton, Charles
Aitken, Jim
Allen, Zoe
Ambler, Rachel
Ambrose, Karen
Ashton, Emma
Avola, Alida
Balakrishnan, Samutheswari
Barns-Jenkins, Caitlin
Barr, Genevieve
Barrell, Sam
Basu, Souradeep
Beale, Rupert
Beesley, Clare
Bhardwaj, Nisha

Bibi, Shahnaz
Bineva-Todd, Ganka
Biswas, Dhruva
Blackman, Michael J.
Bonnet, Dominique
Bowker, Faye
Broncel, Malgorzata
Brooks, Claire
Buck, Michael D.
Buckton, Andrew
Budd, Timothy
Burrell, Alana
Busby, Louise
Bussi, Claudio
Butterworth, Simon
Byott, Matthew
Byrne, Fiona
Byrne, Richard
Caidan, Simon
Campbell, Joanna
Canton, Johnathan
Cardoso, Ana
Carter, Nick
Carvalho, Luiz
Carzaniga, Raffaella
Chandler, Natalie
Chen, Qu
Cherepanov, Peter
Churchward, Laura
Clark, Graham
Clayton, Bobbi
Gigli, Clementina Cobolli
Collins, Zena
Cottrell, Sally
Crawford, Margaret
Cubitt, Laura
Cullup, Tom
Davies, Heledd
Davis, Patrick
Davison, Dara
Dearing, Vicky
Debaisieux, Solene
Diaz-Romero, Monica
Dibbs, Alison
Diring, Jessica
Driscoll, Paul C.
D'Avola, Annalisa

Earl, Christopher
Edwards, Amelia
Ekin, Chris
Evangelopoulos, Dimitrios
Faraway, Rupert
Fearns, Antony
Ferron, Aaron
Fidanis, Efthymios
Fitz, Dan
Fleming, James
Frampton, Daniel
Frederico, Bruno
Gaiba, Alessandra
Gait, Anthony
Gamblin, Steve
Gartner, Kathleen
Gaul, Liam
Golding, Helen M.
Goldman, Jacki
Goldstone, Robert
Dominguez, Belen Gomez
Gong, Hui
Grant, Paul R.
Greco, Maria
Grobler, Mariana
Guedan, Anabel
Gutierrez, Maximiliano G.
Hackett, Fiona
Hall, Ross
Halldorsson, Steinar
Harris, Suzanne
Hashim, Sugera
Hatipoglu, Emine
Healy, Lyn
Heaney, Judith
Herbst, Susanne
Hewitt, Graeme
Higgins, Theresa
Hindmarsh, Steve
Hirani, Rajnika
Hope, Joshua
Horton, Elizabeth
Hoskins, Beth
Howell, Michael
Howitt, Louise
Hoyle, Jacqueline
Htun, Mint R.

Hubank, Michael
Encabo, Hector Huerga
Hughes, Deborah
Hughes, Jane
Huseynova, Almaz
Hwang, Ming-Shih
Instrell, Rachael
Jackson, Deborah
Jamal-Hanjani, Mariam
Jenkins, Lucy
Jiang, Ming
Johnson, Mark
Jones, Leigh
Kanu, Nnennaya
Kassiotis, George
Kelly, Gavin
Kiely, Louise
Teixeira, Anastacio King Spert
Kirk, Stuart
Kjaer, Svend
Knuepfer, Ellen
Komarov, Nikita
Kotzampaltiris, Paul
Kousis, Konstantinos
Krylova, Tammy
Kucharska, Ania
Labrum, Robyn
Lambe, Catherine
Lappin, Michelle
Lee, Stacey-Ann
Levett, Andrew
Levett, Lisa
Levi, Marcel
Liu, Hon Wing
Loughlin, Sam
Lu, Wei-Ting
MacRae, James, I
Madoo, Akshay
Marczak, Julie A.
Martensson, Mimmi
Martinez, Thomas
Marzook, Bishara
Matthews, John
Matz, Joachim M.
McCall, Samuel
McCoy, Laura E.
McKay, Fiona

McNamara, Edel C.
Minutti, Carlos M.
Mistry, Gita
Molina-Arcas, Miriam
Montaner, Beatriz
Montgomery, Kylie
Moore, Catherine
Moore, David
Moraiti, Anastasia
Moreira-Teixeira, Lucia
Mukherjee, Joyita
Naceur-Lombardelli, Cristina
Nelson, Aileen
Nicod, Jerome
Nightingale, Luke
Nofal, Stephanie
Nurse, Paul
Nutan, Savita
Oedekoven, Caroline
O’Garra, Anne
O’Leary, Jean D.
Olsen, Jessica
O’Neill, Olga
O’Reilly, Nicola
Suarez, Paula Ordonez
Osborne, Neil
Pabari, Amar
Pajak, Aleksandra
Papayannopoulos, Venizelos
Paraskevopoulou, Stavroula M.
Patel, Namita
Patel, Yogen
Paun, Oana
Peat, Nigel
Castano, Laura Peces-Barba
Caballero, Ana Perez
Perez-Lloret, Jimena
Perrault, Magali S.
Perrin, Abigail
Poh, Roy
Poirier, Enzo Z.
Polke, James M.
Pollitt, Marc
Prieto-Godino, Lucia
Proust, Alize
Puvirajasinghe, Clinda
Queval, Christophe

Ramachandran, Vijaya
Ramaprasad, Abhinay
Ratcliffe, Peter
Reed, Laura
Reis E Sousa, Caetano
Richardson, Kayleigh
Ridewood, Sophie
Roberts, Fiona
Roberts, Rowenna
Rodgers, Angela
Clavijo, Pablo Romero
Rosa, Annachiara
Rossi, Alice
Roustan, Chloe
Rowan, Andrew
Sahai, Erik
Sait, Aaron
Sala, Katarzyna
Sanchez, Emilie
Sanderson, Theo
Santucci, Pierre
Sardar, Fatima
Sateriale, Adam
Saunders, Jill A.
Sawyer, Chelsea
Schlott, Anja
Schweighoffer, Edina
Segura-Bayona, Sandra
Punatar, Rajvee Shah
Shahmanesh, Maryam
Shaw, Joe
Dos Santos, Mariana Silva
Silvestre, Margaux
Singer, Matthew
Snell, Daniel M.
Song, Ok-Ryul
Spyer, Moira J.
Steel, Louisa
Strange, Amy
Sullivan, Adrienne E.
Tan, Michele S. Y.
Tautz-Davis, Zoe H.
Taylor, Effie
Taylor, Gunes
Taylor, Harriet B.
Taylor-Beadling, Alison
Subtil, Fernanda Teixeira

Torras, Berta Terre
Toolan-Kerr, Patrick
Torelli, Francesca
Toteva, Tea
Treeck, Moritz
Trojer, Hadija
Tsai, Ming-Han C.
Turner, James M. A.
Turner, Melanie
Ule, Jernej
Ulferts, Rachel
Vanloo, Sharon P.
Veeriah, Selvaraju
Venkatesan, Subramanian
Vousden, Karen
Wack, Andreas
Walder, Claire
Walker, Philip A.
Wang, Yiran
Ward, Sophia
Wenman, Catharina
Williams, Luke
Williams, Matthew J.
Wong, Wai Keong
Wright, Joshua
Wu, Mary
Wynne, Lauren
Xiang, Zheng
Yap, Melvyn
Zagalak, Julian A.
Zecchin, Davide
Zillwood, Rachel
Sander, Josemir W.
Sisodiya, Sanjay M.
Carthiyaniamma, Santhakumari
DeTisi, Jane
Dick, Julie
Hill, Andrea
Kipper, Karin
Kullar, Birinder
Norris, Sarah
Rugg-Gunn, Fergus
Salvatierra, Rebecca
Shaya, Gabriel
Sloan, Astrid
Singh, Priyanka
Varley, James

Whatley, Ben
CA Crick Covid Consortium CCC
TI Clinical outcomes of COVID-19 in long-term care facilities for people with epilepsy
SO EPILEPSY & BEHAVIOR
LA English
DT Article
DE SARS-CoV-2; Vulnerable people; Surveillance; Prevention; Care
AB In this cohort study, we aim to compare outcomes from coronavirus disease 2019 (COVID-19) in people with severe epilepsy and other co-morbidities living in long-term care facilities which all implemented early preventative measures, but different levels of surveillance.

During 25-week observation period (16 March-6 September 2020), we included 404 residents (118 children), and 1643 caregivers. We compare strategies for infection prevention, control, and containment, and related outcomes, across four UK long-term care facilities. Strategies included early on-site enhancement of preventative and infection control measures, early identification and isolation of symptomatic cases, contact tracing, mass surveillance of asymptomatic cases and contacts. We measured infection rate among vulnerable people living in the facilities and their caregivers, with asymptomatic and symptomatic cases, including fatality rate.

We report 38 individuals (17 residents) who tested severe acute respiratory syndrome coronavirus 2 (SARS-CoV-2)-positive, with outbreaks amongst residents in two facilities. At Chalfont Centre for Epilepsy (CCE), 10/98 residents tested positive: two symptomatic (one died), eight asymptomatic on weekly enhanced surveillance; 2/275 caregivers tested positive: one symptomatic, one asymptomatic. At St Elizabeth's (STE), 7/146 residents tested positive: four symptomatic (one died), one positive during hospital admission for symptoms unrelated to COVID-19, two asymptomatic on one-off testing of all 146 residents; 106/601 symptomatic caregivers were tested, 13 positive. In addition, during two cycles of systematically testing all asymptomatic carers, four tested positive. At The Meath (TM), 8/80 residents were symptomatic but none tested; 26/250 caregivers were tested, two positive. At Young Epilepsy (YE), 8/80 children were tested, all negative; 22/517 caregivers were tested, one positive.

Infection outbreaks in long-term care facilities for vulnerable people with epilepsy can be quickly contained, but only if asymptomatic individuals are identified through enhanced surveillance at resident and caregiver level. We observed a low rate of morbidity and mortality, which confirmed that preventative measures with isolation of suspected and confirmed COVID-19 residents can reduce resident-to-resident and resident-to-caregiver transmission. Children and young adults appear to have lower infection rates. Even in people with epilepsy and multiple co-morbidities, we observed a high percentage of asymptomatic people suggesting that epilepsy-related factors (anti-seizure medications and seizures) do not necessarily lead to poor outcomes. (C) 2020 Elsevier Inc. All rights reserved.
C1 [Balestrini, Simona; Koepp, Matthias J.; Silvennoinen, Katri; Xiao, Fenglai; Zagaglia, Sara; Sander, Josemir W.; Sisodiya, Sanjay M.] UCL Queen Sq Inst Neurol, Dept Clin & Expt Epilepsy, London WC1N 3BG, England.

[Balestrini, Simona; Koepp, Matthias J.; Anders-Cannon, Jonny; Sander, Josemir W.; Sisodiya, Sanjay M.] Chalfont Ctr Epilepsy CCE, Gerrards Cross SL9 0RJ, Bucks, England.

[Gandhi, Sonia; Swanton, Charles] Francis Crick Inst, London NW1 1AT, England.
[Gandhi, Sonia; Swanton, Charles] UCL Canc Inst, London WC1E 6DD, England.
[Gandhi, Sonia; Rickman, Hannah M.; Shin, Gee Yen; Houlihan, Catherine F.; Nastouli, Eleni; Swanton, Charles] NHS Fdn Trust, Univ Coll London Hosp, Dept Virol, London NW1 2PG, England.
[Hudgell, Kirsty; Ziomek, Mariusz; Haimes, Paul; Sampson, Adam] St Elizabeth STE, Much Hadham SG10 6EW, Herts, England.
[Parker, Annie] Meath TM, Westbrook Rd, Godalming GU7 2QH, Surrey, England.
[Cross, J. Helen] Great Ormond St Hosp GOSH, London WC1N 1EH, England.
[Cross, J. Helen] UCL Inst Child Hlth, London WC1N 1EH, England.
[Cross, J. Helen; Pardington, Rosemarie] Young Epilepsy YE, Lingfield RH7 6PW, Surrey, England.
[Nastouli, Eleni] UCL GOS Inst Child Hlth, Dept Populat Policy & Practice, London WC1N 1EH, England.
[Sander, Josemir W.] Stichting Epilepsie Instellingen Nederland SEIN, Heemstede, Netherlands.
C3 University of London; University College London; Francis Crick Institute; University of London; University College London; Oxford University Hospitals NHS Foundation Trust; University College London Hospitals NHS Foundation Trust; University of London; University College London; University of London; University College London; Great Ormond Street Hospital for Children NHS Foundation Trust; University of London; University College London; University of London; University College London
RP Koepp, MJ; Sander, JW (通讯作者), UCL Queen Sq Inst Neurol, Dept Clin & Expt Epilepsy, London WC1N 3BG, England.
EM m.koepp@ucl.ac.uk; l.sander@ucl.ac.uk
TC 5
Z9 5
PD FEB
PY 2021
VL 115
AR 107602
DI 10.1016/j.yebeh.2020.107602
EA FEB 2021
WC Behavioral Sciences; Clinical Neurology; Psychiatry
ER

PT J
AU Banks, J
Corrigan, D
Grogan, R
El-Naggar, H
White, M
Doran, E
Synnott, C
Fitzsimons, M

Delanty, N
Doherty, CP
AF Banks, Jack
Corrigan, Derek
Grogan, Roger
El-Naggar, Hany
White, Maire
Doran, Elisabeth
Synnott, Cara
Fitzsimons, Mary
Delanty, Norman
Doherty, Colin P.
TI LoVE in a time of CoVID: Clinician and patient experience using telemedicine for chronic epilepsy management
SO EPILEPSY & BEHAVIOR
LA English
DT Article
DE Telemedicine; Electronic Patient Records; Digital Health; eHealth; Remote Care
AB As part of our ongoing interest in patient- and family-centered care in epilepsy, we began, before the onset of the CoVID-19 pandemic, to evaluate the concerns and preferences of those delivering and receiving care via telemedicine. CoVID-19 arrived and acted as an unexpected experiment in nature, catalyzing telemedicine's widespread implementation across many disciplines of medicine. The arrival of CoVID-19 in Ireland gave us the opportunity to record these perceptions pre- and post-CoVID. Data were extracted from the National Epilepsy Electronic Patient Record (EEPR). Power BI Analytics collated data from two epilepsy centers in Dublin. Analysis of data on reasons for using the telephone support line was conducted. A subset of patients and clinicians who attended virtual encounters over both periods were asked for their perception of telemedicine care through a mixed methods survey. Between 23rd December 2019 and 23rd March 2020 (pre-CoVID era), a total of 1180 patients were seen in 1653 clinical encounters. As part of a telemedicine pilot study, 50 of these encounters were scheduled virtual telephone appointments. Twenty eight surveys were completed by clinicians and 18 by patients during that period. From 24th March 2020 to 24th June 2020, 1164 patients were seen in 1693 encounters of which 729 (63%) patients were seen in 748 scheduled virtual encounters. 118 clinician impressions were captured through an online survey and 75 patients or carers completed a telephone survey during the post-CoVID era. There was no backlog of appointments or loss of care continuity forced by the pandemic. Clinicians expressed strong levels of satisfaction, but some doubted the suitability of new patients to the service or candidates for surgery receiving care via telemedicine. Patients reported positive experiences surrounding telephone appointments comparing them favorably to face-to-face encounters. The availability of a shared EEPR demonstrated no loss of care contact for patients with epilepsy. The survey showed that telemedicine is seen as an effective and satisfactory method of delivering chronic outpatient care. (C) 2020 The Authors. Published by Elsevier Inc.
C1 [Banks, Jack; Corrigan, Derek; El-Naggar, Hany; White, Maire; Fitzsimons, Mary; Delanty, Norman; Doherty, Colin P.] Royal Coll Surgeons Ireland, FutureNeuro SFI Res

Ctr, 123 St Stephens Green, Dublin 2, Ireland.
[Banks, Jack; Doherty, Colin P.] Acad Unit Neurol, Trinity Coll Dublin, Sch Med, Dublin 2, Ireland.
[Grogan, Roger; El-Naggar, Hany; White, Maire; Delanty, Norman] Beaumont Hosp, Dept Neurol, Dublin 9, Ireland.
[Doran, Elisabeth; Synnott, Cara; Doherty, Colin P.] St James Hosp, Dept Neurol, Dublin 8, Ireland.
C3 Royal College of Surgeons - Ireland; League of European Research
Universities - LERU; Trinity College Dublin; League of European Research
Universities - LERU; Trinity College Dublin
RP Banks, J (通讯作者), St James Hosp, Trinity Ctr Hlth Sci, Room 1-05, Dublin 8, Ireland.
EM banksja@tcd.ie
TC 10
Z9 10
PD FEB
PY 2021
VL 115
AR 107675
DI 10.1016/j.yebeh.2020.107675
EA FEB 2021
WC Behavioral Sciences; Clinical Neurology; Psychiatry
ER

PT J
AU Dozieres-Puyravel, B
Auvin, S
AF Dozieres-Puyravel, Blandine
Auvin, Stephane
TI Usefulness, limitations, and parental opinion about teleconsultation for
rare pediatric epilepsies
SO EPILEPSY & BEHAVIOR
LA English
DT Article
DE CoviD-19; Epilepsy; Teleconsultation
AB Aim: Evaluation of the usefulness and the parental opinion about teleconsultation (TC) for rare pediatric epilepsies.
Method: One-month prospective survey of consecutive TCs. All clinics on site have been turned into TC in the context of COVID-19 pandemic. The physicians quoted all TCs while the parents expressed their opinion though an invitation for an online questionnaire.
Results: We included 151 TCs (145 patients) among the 259 epilepsy TCs done during the study period. The parental questionnaire has been answered 105 times. The physicians felt confident to organize a TC for the next visit of 74.8% of the children, but some limits were identified such as the absence of physical examination, weight, and psychomotor development evaluation.
The physicians felt more confident for a new TC in older patients (9.5 +/- 5.5 years versus 5.3 +/- 4.3 years) and in stable patients (73.8% confident for instable, 82.8%

for stable). Parents were satisfied with TC feeling that it answered health issues in a better manner than a clinic pinpointing the gain of time and the absence of travel. However, half of them would prefer a clinic for the next appointment.

Interpretation: Teleconsultation seems useful answering the patients' needs according to both physicians and families. Despite some limitations, it is most likely that TCs become a new part of the clinical activities in rare pediatric epilepsy centers.
(C) 2020 Elsevier Inc. All rights reserved.
C1 [Dozieres-Puyravel, Blandine; Auvin, Stephane] Hop Robert Debre, AP HP, Serv Neurol Pediat, Paris, France.

[Dozieres-Puyravel, Blandine; Auvin, Stephane] Univ Paris, INSERM NeuroDiderot, Paris, France.

C3 Assistance Publique Hopitaux Paris (APHP); Hopital Universitaire Robert-Debre - APHP; UDICE-French Research Universities; Universite de Paris; UDICE-French Research Universities; Universite de Paris

RP Auvin, S (通讯作者), CHU Hop Robert Debre, Serv Neurol Pediat, 48 Blvd Serurier, F-75935 Paris 19, France.

EM stephane.auvin@aphp.fr
TC 0
Z9 0
PD FEB
PY 2021
VL 115
AR 107656
DI 10.1016/j.yebeh.2020.107656
EA FEB 2021
WC Behavioral Sciences; Clinical Neurology; Psychiatry
ER

PT J
AU Leung, WCY
Lau, EHY
Kwan, P
Chang, RSK
AF Leung, William C. Y.
Lau, Eric H. Y.
Kwan, Patrick
Chang, Richard Shek-kwan
TI Impact of COVID-19 on seizure-related emergency attendances and hospital admissions - A territory-wide observational study
SO EPILEPSY & BEHAVIOR
LA English
DT Article
DE COVID-19; Pandemic; Epilepsy; Seizure; Help-seeking behavior
AB This is a territory-wide study to investigate the impact of coronavirus disease 2019 (COVID-19) pandemic on Accident and Emergency Department (A&E) attendances and acute ward admissions for seizures. Adult patients who presented to the A&E with seizures from January 23, 2020 to March 24, 2020 (study period) were included and compared with

parallel intervals from 2015 to 2019 (control periods). Preexisting time trend in control periods and potential changes during COVID-19 were analyzed by Poisson, negative and logistic regression models.
Accident and Emergency Department attendances and ward admissions for seizures decreased significantly during the COVID-19 pandemic. A total of 319 and 230 recorded ward admissions and A&E attendances for seizures were identified during the study period in 2020, compared with 494 and 343 per annum, respectively in the control periods. The ratio of acute ward admission per A&E attendance for seizures did not change significantly. Intensive care utility and mortality rates remained stable.
For some patients, delaying medical attention due to fear of nosocomial COVID-19 cross-infection may lead to severe or even life-threatening consequences. This change in medical help-seeking behavior calls for new medical care models to meet the service gap. Education to patients with epilepsy and their caregivers is of utmost importance during this pandemic. (C) 2020 Elsevier Inc. All rights reserved.
C1 [Leung, William C. Y.; Chang, Richard Shek-kwan] Univ Hong Kong, Queen Mary Hosp, Dept Med, Div Neurol, Hong Kong, Peoples R China.
[Lau, Eric H. Y.] Univ Hong Kong, Li Ka Shing Fac Med, Sch Publ Hlth, Hong Kong, Peoples R China.
[Kwan, Patrick] Monash Univ, Alfred Hosp, Cent Clin Sch, Dept Neurosci, Melbourne, Vic, Australia.
C3 University of Hong Kong; University of Hong Kong; Florey Institute of Neuroscience & Mental Health; Monash University
RP Chang, RSK (通讯作者), Queen Mary Hosp, Dept Med, Pokfulam, 4-F Professorial Block, Hong Kong, Peoples R China.
EM changsk@ha.org.hk
TC 7
Z9 7
PD FEB
PY 2021
VL 115
AR 107497
DI 10.1016/j.yebeh.2020.107497
EA FEB 2021
WC Behavioral Sciences; Clinical Neurology; Psychiatry
ER

PT J
AU Pasca, L
Zanaboni, MP
Grumi, S
Totaro, M
Ballante, E
Varesio, C
De Giorgis, V
AF Pasca, Ludovica
Zanaboni, Martina Paola
Grumi, Serena

Totaro, Martina
Ballante, Elena
Varesio, Costanza
De Giorgis, Valentina
TI Impact of COVID-19 pandemic in pediatric patients with epilepsy with neuropsychiatric comorbidities: A telemedicine evaluation
SO EPILEPSY & BEHAVIOR
LA English
DT Article
DE Epilepsy; Neuropsychiatric comorbidities; COVID-19; Anxiety; Depression; Learning disabilities
ID DISORDERS; ANXIETY
AB Objective: The objective of this study was to evaluate care needs, emotional and behavioral changes, and parental stress indices in a cohort of pediatric patients with epilepsy with neurocognitive and emotional comorbidities at the time of the coronavirus disease 2019 (COVID-19) pandemic.

Methods: This is a prospective observational study involving pediatric patients with epilepsy with neurocognitive and emotional comorbidities. Included patients were admitted to our hospital between August 2019 and February 2020 for epilepsy and neuropsychiatric assessment, and Child Behavior Checklist (CBCL) questionnaires were filled in by parents. Those patients and their families accepted to participate in a phone follow-up visit in April-May 2020 and to refill CBCL and Parenting Stress Index-Short Form (PSI-SF) questionnaires.

Descriptive statistics for demographic and clinical data, CBCL questionnaire scores before and during the COVID-19 pandemic, and PSI-SF scores have been computed. Moreover, results of a short phone survey on the psychological burden during COVID lockdown have been reported.

Results: This study provides the parental-proxy report of emotional and behavioral profile changes of 23 pediatric patients with epilepsy and neurocognitive and emotional comorbidities during the COVID-19 pandemic. Concerns for therapy monitoring at the time of lockdown emerged in 43% of families, and 30% of patients showed worries for an altered contact with the referring medical team.

Patients with neurocognitive comorbidities were more likely to exhibit behavioral problems, especially externalizing problems compared with patients with a diagnosis of anxiety/depression.

Conclusion: Our data suggest the importance to monitor disease trajectory and behavior and affective symptoms with telehealth strategies to provide effective care to patients and their families. (C) 2020 Elsevier Inc. All rights reserved.
C1 [Pasca, Ludovica; Zanaboni, Martina Paola; Grumi, Serena; Totaro, Martina; Varesio, Costanza; De Giorgis, Valentina] IRCSS Mondino Fdn, Dept Child Neurol & Psychiat, Via Mondino 2, I-27100 Pavia, PV, Italy.

[Pasca, Ludovica; Totaro, Martina] Univ Pavia, Dept Brain & Behav Neurosci, Pavia, Italy.

[Ballante, Elena] IRCCS Mondino Fdn, BioData Sci Ctr, Pavia, Italy.
C3 University of Pavia; IRCCS Fondazione Casimiro Mondino
RP Varesio, C (通讯作者), IRCSS Mondino Fdn, Dept Child Neurol & Psychiat, Via Mondino 2, I-27100 Pavia, PV, Italy.

EM ludovica.pasca01@universitadipavia.it; martinapaola.zanaboni@mondino.it;
martina.totaro01@universitadipavia.it;
elena.ballante01@universitadipavia.it; costanza.varesio@mondino.it;
valentina.degiorgis@mondino.it
TC 10
Z9 10
PD FEB
PY 2021
VL 115
AR 107519
DI 10.1016/j.yebeh.2020.107519
EA FEB 2021
WC Behavioral Sciences; Clinical Neurology; Psychiatry
ER

PT J
AU Puteikis, K
Jasionis, A
Mameniskiene, R
AF Puteikis, Kristijonas
Jasionis, Arminas
Mameniskiene, Ruta
TI Recalling the COVID-19 lockdown: Insights from patients with epilepsy
SO EPILEPSY & BEHAVIOR
LA English
DT Article
DE COVID-19; Epilepsy; Lockdown; Seizure exacerbation; Telemedicine
AB Purpose: The purpose of our study was to explore health changes among people with epilepsy (PWE) during a national COVID-19 lockdown in the context of patients' clinical characteristics and their experience of receiving epilepsy-related medical services.

Methods: A questionnaire was distributed for adult PWE both online and at a tertiary epilepsy center after the end of a national lockdown in Lithuania. PWE were asked to evaluate their health status during the lockdown and estimate changes in their seizure patterns. Additional questions concerned the accessibility and quality of epilepsy-related consultations.

Results: The study sample consisted of 143 PWE (59 [41.3%] male, mean age 35.1 +/- 13.4 years), 94 (65.7%) completed the survey in person, 49 (34.3%) - online. A deterioration in reported physical and mental health during lockdown was observed ($Z = -4.604$, $p < 0.0001$ and $Z = -4.253$, $p < 0.0001$, respectively) and 22 (15.4%) PWE reported seizure exacerbation. In an ordinal logistic regression model (analysis of data from all participants), baseline seizure frequency ($b = 0.413$, $p = 0.031$), reported physical health before lockdown ($b = -0.462$, $p = 0.031$) and the ease of proper antiepileptic drug (AED) use during the imposed restrictions ($b = -0.535$, $p = 0.006$) were statistically significant variables associated with changes in seizure frequency. The latter were not affected by modifications in AED use (Mann-Whitney $U = 1127.0$, $p = 0.307$) irrespective of the data collection method.

With teleconsultations being predominant during the lockdown, an overall decline

in the quality of epilepsy-related consultations was observed (Z = -2.895, p = 0.004). Among all participants, 46 (32.2%) lost an epilepsy-related consultation or medical service because of the lockdown. This loss was found to be associated with seizure exacerbation (Mann-Whitney U = 1622.5, p = 0.046).
Conclusion: Our study indicates that a national COVID-19 lockdown may have led to worse seizure control and health status in some PWE. Easy access to AEDs and their appropriate use may be especially useful to prevent seizure exacerbation during strict COVID-19 restrictions. The quality and accessibility of remote epilepsy-related consultations was suboptimal and may require further improvement during disruption of in-person services. (C) 2020 Published by Elsevier Inc.
C1 [Puteikis, Kristijonas] Vilnius Univ, Fac Med, Vilnius, Lithuania.
[Jasionis, Arminas; Mameniskiene, Ruta] Vilnius Univ, Ctr Neurol, Santariskiu G 2, LT-08661 Vilnius, Lithuania.
C3 Vilnius University; Vilnius University
RP Mameniskiene, R (通讯作者), Vilnius Univ, Ctr Neurol, Santariskiu G 2, LT-08661 Vilnius, Lithuania.
EM ruta.mameniskiene@santa.lt
TC 10
Z9 10
PD FEB
PY 2021
VL 115
AR 107573
DI 10.1016/j.yebeh.2020.107573
EA FEB 2021
WC Behavioral Sciences; Clinical Neurology; Psychiatry
ER

PT J
AU Stafstrom, CE
Sun, LR
Kossoff, EH
Dabrowski, AK
Singhi, S
Kelley, SA
AF Stafstrom, Carl E.
Sun, Lisa R.
Kossoff, Eric H.
Dabrowski, Ania K.
Singhi, Samata
Kelley, Sarah A.
TI Diagnosing and managing childhood absence epilepsy by telemedicine
SO EPILEPSY & BEHAVIOR
LA English
DT Article
DE Child; Absence epilepsy; Hyperventilation; Seizure; Telemedicine;
Ethosuximide

AB The diagnosis of childhood absence epilepsy (CAE) is typically based on history and description of spells, supported by an office-based positive hyperventilation test and confirmed by routine electroencephalography (EEG). In the current coronavirus disease 2019 (COVID-19) pandemic, many pediatric neurologists have switched to telemedicine visits for nonemergent outpatient evaluations. We present a series of children diagnosed as having CAE on the basis of a positive hyperventilation test performed during remote televisits. Several of these children were begun on treatment for CAE prior to obtaining an EEG, with significant seizure reduction. Our series documents the feasibility of CAE diagnosis and management by telemedicine. (C) 2020 Elsevier Inc. All rights reserved.
C1 [Stafstrom, Carl E.; Sun, Lisa R.; Kossoff, Eric H.; Dabrowski, Ania K.; Singhi, Samata; Kelley, Sarah A.] Johns Hopkins Univ, Sch Med, Dept Neurol, Div Pediat Neurol, Baltimore, MD 21205 USA.
C3 Johns Hopkins University
RP Stafstrom, CE (通讯作者), Johns Hopkins Univ Hosp, Div Pediat Neurol, Rubenstein 2157,200 N Wolfe St, Baltimore, MD 21287 USA.
EM cstafst1@jhmi.edu
TC 2
Z9 2
PD FEB
PY 2021
VL 115
AR 107404
DI 10.1016/j.yebeh.2020.107404
EA FEB 2021
WC Behavioral Sciences; Clinical Neurology; Psychiatry
ER

PT J
AU Thorpe, J
Ashby, S
Hallab, A
Ding, D
Andraus, M
Dugan, P
Perucca, P
Costello, D
French, JA
O'Brien, TJ
Depondt, C
Andrade, DM
Sengupta, R
Delanty, N
Jette, N
Newton, CR
Brodie, MJ
Devinsky, O

Cross, JH
Sander, JW
Hanna, J
Sen, A
AF Thorpe, Jennifer
Ashby, Samantha
Hallab, Asma
Ding, Ding
Andraus, Maria
Dugan, Patricia
Perucca, Piero
Costello, Daniel
French, Jacqueline A.
O'Brien, Terence J.
Depondt, Chantal
Andrade, Danielle M.
Sengupta, Robin
Delanty, Norman
Jette, Nathalie
Newton, Charles R.
Brodie, Martin J.
Devinsky, Orrin
Cross, J. Helen
Sander, Josemir W.
Hanna, Jane
Sen, Arjune
CA COVID-19 Epilepsy COV-E Study Grp
TI Evaluating risk to people with epilepsy during the COVID-19 pandemic: Preliminary findings from the COV-E study
SO EPILEPSY & BEHAVIOR
LA English
DT Article
DE Coronavirus; Chronic illness; Mental health; Seizure; SUDEP
AB The COVID-19 pandemic has caused global anguish unparalleled in recent times. As cases rise, increased pressure on health services, combined with severe disruption to people's everyday lives, can adversely affect individuals living with chronic illnesses, including people with epilepsy. Stressors related to disruption to healthcare, finances, mental well-being, relationships, schooling, physical activity, and increased isolation could increase seizures and impair epilepsy self-management.

We aim to understand the impact that COVID-19 has had on the health and well-being of people with epilepsy focusing on exposure to increased risk of seizures, associated comorbidity, and mortality. We designed two online surveys with one addressing people with epilepsy directly and the second for caregivers to report on behalf of a person with epilepsy.

The survey is ongoing and has yielded 463 UK-based responses by the end of September 2020. Forty percent of respondents reported health changes during the pandemic (n = 185). Respondents cited a change in seizures (19%, n = 88), mental health difficulties

(34%, n = 161), and sleep disruption (26%, n = 121) as the main reasons. Thirteen percent found it difficult to take medication on time. A third had difficulty accessing medical services (n = 154), with 8% having had an appointment canceled (n = 39). Only a small proportion reported having had discussions about epilepsy-related risks, such as safety precautions (16%, n = 74); mental health (29%, n = 134); sleep (30%, n = 140); and Sudden Unexpected Death in Epilepsy (SUDEP; 15%, n = 69) in the previous 12 months.

These findings suggest that people with epilepsy are currently experiencing health changes, coupled with inadequate access to services. Also, there seems to be a history of poor risk communication in the months preceding the pandemic. As the UK witnesses a second COVID-19 wave, those involved in healthcare delivery must ensure optimal care is provided for people with chronic conditions, such as epilepsy, to ensure that avoidable morbidity and mortality is prevented during the pandemic, and beyond. (C) 2020 Elsevier Inc. All rights reserved.
C1 [Thorpe, Jennifer; Newton, Charles R.; Sen, Arjune] John Radcliffe Hosp, NIHR Biomed Res Ctr, Nuffield Dept Clin Neurosci, Oxford Epilepsy Res Grp, Oxford OX3 9DU, England.

[Thorpe, Jennifer; Ashby, Samantha; Hanna, Jane] SUDEP Act, 18 Newbury St, Wantage OX12 8DA, Oxon, England.

[Hallab, Asma] Humboldt Univ, Freie Univ Berlin, Charite Universitatsmed Berlin, Dept Nucl Med, Berlin, Germany.

[Hallab, Asma] Berlin Inst Hlth, Berlin, Germany.

[Ding, Ding] Fudan Univ Huashan Hosp, Inst Neurol, Shanghai, Peoples R China.

[Andraus, Maria] Univ Fed Rio de Janeiro, Clementino Fraga Filho Univ Hosp, Dept Internal Med, Neurol Serv,Epilepsy Program, Rio De Janeiro, Brazil.

[Dugan, Patricia; French, Jacqueline A.; Devinsky, Orrin] NYU, Dept Neurol, Grossman Sch Med, New York, NY USA.

[Perucca, Piero; O'Brien, Terence J.] Monash Univ, Dept Neurosci, Alfred Hosp, Cent Clin Sch, Melbourne, Vic, Australia.

[Perucca, Piero; O'Brien, Terence J.] Univ Melbourne, Royal Melbourne Hosp, Dept Med, Melbourne, Vic, Australia.

[Perucca, Piero; O'Brien, Terence J.] Univ Melbourne, Royal Melbourne Hosp, Dept Neurol, Melbourne, Vic, Australia.

[Costello, Daniel] Cork Univ Hosp & Coll Med & Hlth, Univ Coll Cork, Epilepsy Serv, Cork, Ireland.

[Depondt, Chantal] Univ Libre Bruxelles, Dept Neurol, Hop Erasme, Brussels, Belgium.

[Andrade, Danielle M.] Univ Toronto, Toronto Western Hosp, Adult Epilepsy Genet Program, Toronto, ON, Canada.

[Sengupta, Robin] Inst Neurosci, Kolkata, India.

[Delanty, Norman] Beaumont Hosp, Royal Coll Surg Ireland, FutureNeuro Res Ctr, Dublin, Ireland.

[Delanty, Norman] Royal Coll Surgeons Ireland, Sch Pharm & Biomol Sci, FutureNeuro Res Ctr, Dublin, Ireland.

[Jette, Nathalie] Icahn Sch Med Mt Sinai, Dept Neurol, New York, NY USA.

[Newton, Charles R.] Univ Oxford, Univ Dept Psychiat, Oxford, England.

[Brodie, Martin J.] West Glasgow Ambulatory Care Hosp Yorkhill, Epilepsy Unit, Glasgow, Lanark, Scotland.

[Cross, J. Helen] UCL NIHR BRC Great Ormond St Inst Child Hlth, London, England.

[Cross, J. Helen] Young Epilepsy, St Piers Lane, Lingfield RH7 6P, England.

[Sander, Josemir W.] UCL Queen Sq Inst Neurol, Queen Sq, London WC1N 3BG, England.
[Sander, Josemir W.] Chalfont Ctr Epilepsy, Gerrards Cross SL9 0RJ, England.
[Sander, Josemir W.] Stichting Epilepsie Instellingen Nederland SEIN, Heemstede, Netherlands.
C3 League of European Research Universities - LERU; University of Oxford; Free University of Berlin; Humboldt University of Berlin; Charite Universitatsmedizin Berlin; Universidade Federal do Rio de Janeiro; New York University; Florey Institute of Neuroscience & Mental Health; Monash University; Royal Melbourne Hospital; University of Melbourne; Royal Melbourne Hospital; University of Melbourne; University College Cork; Universite Libre de Bruxelles; University of Toronto; University Health Network Toronto; Royal College of Surgeons - Ireland; Royal College of Surgeons - Ireland; Icahn School of Medicine at Mount Sinai; League of European Research Universities - LERU; University of Oxford; University of London; University College London
RP Sen, A (通讯作者), John Radcliffe Hosp, Dept Neurol, 3rd Floor,West Wing, Oxford OX3 9DU, England.
EM arjune.sen@ndcn.ox.ac.uk
TC 14
Z9 14
PD FEB
PY 2021
VL 115
AR 107658
DI 10.1016/j.yebeh.2020.107658
EA FEB 2021
WC Behavioral Sciences; Clinical Neurology; Psychiatry
ER

PT J
AU Balestrini, S
Wilson, G
Eldred, C
Evans, H
Sisodiya, SM
AF Balestrini, Simona
Wilson, Galia
Eldred, Claire
Evans, Helen
Sisodiya, Sanjay M.
TI The impact of COVID-19 in Dravet syndrome: A UK survey
SO ACTA NEUROLOGICA SCANDINAVICA
LA English
DT Article
DE epilepsy; infections; seizure
ID VACCINATION; DISEASE; SEIZURES; RISK
AB Objectives To understand the risks, impact and outcome of COVID-19 in people affected

by Dravet Syndrome (DS).
Materials and Methods An anonymous cross-sectional online survey was conducted between June 17 and July 13, 2020, addressed to families of people with DS.
Results A total of 116 responses were collected, from families of children (n = 86; 74%) and adults (30; 26%) with DS. The majority (106; 91%) were shielded at the family home during lockdown. Symptoms compatible with COVID-19 were reported in 22 (19%) individuals. Only four individuals with symptoms had a PCR swab test, none of which was positive. Only one symptomatic person had antibody testing (but not swab testing), which was positive. One person had repeatedly positive swab tests whilst in hospital for renal failure, but had no typical symptoms of COVID-19. In 50% of people with DS who developed possible or probable COVID-19 symptoms, seizure worsening was reported, in terms of increased seizure frequency or duration or both. Medical attention was required in 9/22 (41%), all of whom were children.
Conclusions In this cohort of people with DS, we observed an infection rate, determined by compatible symptoms, of 19%, with no deaths and benign outcome in most cases despite the underlying complex epilepsy although children often required medical attention. Early adoption of preventative measures, including testing of symptomatic individuals, regular surveillance for people living in residential care facilities, and shielding of individuals with comorbidities increasing the risk of severe outcome, may limit the impact of COVID-19.
C1 [Balestrini, Simona; Sisodiya, Sanjay M.] UCL Queen Sq Inst Neurol, Dept Clin & Expt Epilepsy, London WC1N 3BG, England.
[Balestrini, Simona; Sisodiya, Sanjay M.] Chalfont Ctr Epilepsy, Gerrards Cross, England.
[Wilson, Galia; Eldred, Claire; Evans, Helen] Dravet Syndrome UK DSUK, London, England.
[Wilson, Galia; Eldred, Claire; Evans, Helen] Dravet Syndrome European Federat DSEF, London, England.
C3 University of London; University College London
RP Balestrini, S (通讯作者), UCL Queen Sq Inst Neurol, Dept Clin & Expt Epilepsy, London WC1N 3BG, England.
EM s.balestrini@ucl.ac.uk
TC 2
Z9 2
PD APR
PY 2021
VL 143
IS 4
BP 389
EP 395
DI 10.1111/ane.13405
EA FEB 2021
WC Clinical Neurology
ER

PT J
AU Gao, Y

Chen, YM
Liu, M
Niu, MM
Song, ZW
Yan, ML
Tian, JH
AF Gao, Ya
Chen, Yamin
Liu, Ming
Niu, Mingming
Song, Ziwei
Yan, Meili
Tian, Jinhui
TI Nervous system diseases are associated with the severity and mortality of patients with COVID-19: a systematic review and meta-analysis
SO EPIDEMIOLOGY AND INFECTION
LA English
DT Review
DE Cerebrovascular disease; COVID-19; meta-analysis; mortality; nervous system disease; severe illness
ID CLINICAL CHARACTERISTICS; HOSPITALIZED-PATIENTS; CORONAVIRUS; WUHAN; INFECTION; OUTCOMES; PERFORMANCE; PNEUMONIA; PROVINCE; STROKE
AB Coronavirus disease 2019 (COVID-19) has become a global pandemic. Previous studies showed that comorbidities in patients with COVID-19 are risk factors for adverse outcomes. This study aimed to clarify the association between nervous system diseases and severity or mortality in patients with COVID-19. We performed a systematic literature search of four electronic databases and included studies reporting the prevalence of nervous system diseases in COVID-19 patients with severe and non-severe disease or among survivors and non-survivors. The included studies were pooled into a meta-analysis to calculate the odds ratio (OR) with 95% confidence intervals (95%CI). We included 69 studies involving 17 879 patients. The nervous system diseases were associated with COVID-19 severity (OR = 3.19, 95%CI: 2.37 to 4.30, $P < 0.001$) and mortality (OR = 3.75, 95%CI: 2.68 to 5.25, $P < 0.001$). Specifically, compared with the patients without cerebrovascular disease, patients with cerebrovascular disease infected with COVID-19 had a higher risk of severity (OR = 3.10, 95%CI: 2.21 to 4.36, $P < 0.001$) and mortality (OR = 3.45, 95% CI: 2.46 to 4.84, $P < 0.001$). Stroke was associated with severe COVID-19 disease (OR = 1.95, 95%CI: 1.11 to 3.42, P = 0.020). No significant differences were found for the prevalence of epilepsy (OR = 1.00, 95%CI: 0.42 to 2.35, P = 0.994) and dementia (OR = 2.39, 95%CI: 0.55 to 10.48, P = 0.247) between non-severe and severe COVID-19 patients. There was no significant association between stroke (OR = 1.79, 95%CI: 0.76 to 4.23, P = 0.185), epilepsy (OR = 2.08, 95%CI: 0.08 to 50.91, P = 0.654) and COVID-19 mortality. In conclusion, nervous system diseases and cerebrovascular disease were associated with severity and mortality of patients with COVID-19. There might be confounding factors that influence the relationship between nervous system diseases and COVID-19 severity as well as mortality.
C1 [Gao, Ya; Chen, Yamin; Liu, Ming; Niu, Mingming; Song, Ziwei; Yan, Meili; Tian, Jinhui] Lanzhou Univ, Sch Basic Med Sci, Evidence Based Med Ctr, Lanzhou 730000, Peoples R China.

[Gao, Ya; Chen, Yamin; Liu, Ming; Niu, Mingming; Song, Ziwei; Yan, Meili] Lanzhou Univ, Sch Nursing, Evidence Based Nursing Ctr, Lanzhou 730000, Peoples R China.
[Tian, Jinhui] Lanzhou Univ, Key Lab Evidence Based Med & Knowledge Translat G, Lanzhou 730000, Peoples R China.
C3 Lanzhou University; Lanzhou University; Lanzhou University
RP Tian, JH (通讯作者), Lanzhou Univ, Sch Basic Med Sci, Evidence Based Med Ctr, Lanzhou 730000, Peoples R China.; Tian, JH (通讯作者), Lanzhou Univ, Key Lab Evidence Based Med & Knowledge Translat G, Lanzhou 730000, Peoples R China.
EM tianjh@lzu.edu.cn
TC 1
Z9 1
PD FEB 15
PY 2021
VL 149
AR e66
DI 10.1017/S0950268821000376
WC Public, Environmental & Occupational Health; Infectious Diseases
ER

PT J
AU Asadi-Pooya, AA
Emami, A
Akbari, A
Javanmardi, F
AF Asadi-Pooya, Ali A.
Emami, Amir
Akbari, Ali
Javanmardi, Fatemeh
TI COVID-19 presentations and outcome in patients with epilepsy
SO ACTA NEUROLOGICA SCANDINAVICA
LA English
DT Article
DE coronavirus; COVID; epilepsy; mortality; seizure
AB Objective To determine whether patients with epilepsy (PWE) are particularly over-represented in a very large cohort of patients with COVID-19. We also investigated whether COVID-19 is associated with a different clinical picture or a more severe course of illness in PWE (compared with others).
Methods All consecutive patients who referred to and admitted at healthcare facilities anywhere in Fars province (located in the south of Iran with a population of 4,851,000 people) from February 19, 2020 until November 20, 2020 were included.
Results A total of 37,968 patients were studied. Eighty-two patients (0.2%) had pre-existing epilepsy. Seizures were significantly more frequent among PWE as a presenting manifestation of COVID-19 compared with that in people without epilepsy (Odds Ratio = 27; p = 0.0001). Furthermore, PWE less often reported cough (significantly) and more often had gastrointestinal symptoms (vomiting and anorexia; as trends) compared with those in people without epilepsy. Patients with epilepsy were not differently likely to be intubated or admitted at ICUs. Case fatality rates were not

different between the two groups [9.8% in PWE and 8.5% in people without epilepsy; p = 0.690].

Conclusion Patients with epilepsy are not susceptible to contracting COVID-19 more than other individuals. Furthermore, COVID-19 in PWE is not associated with a more severe illness or a poorer prognosis. However, PWE and COVID-19 may present somewhat differently than others with such an illness. Why PWE less often present with cough and more often present with gastrointestinal symptoms is not clear yet and should be investigated and clarified in the future studies.

C1 [Asadi-Pooya, Ali A.] Shiraz Univ Med Sci, Epilepsy Res Ctr, Shiraz, Iran.
[Asadi-Pooya, Ali A.] Thomas Jefferson Univ, Dept Neurol, Jefferson Comprehens Epilepsy Ctr, Philadelphia, PA USA.
[Emami, Amir; Javanmardi, Fatemeh] Shiraz Univ Med Sci, Burn & Wound Healing Res Ctr, Shiraz, Iran.
[Akbari, Ali] Shiraz Univ Med Sci, Sch Med, Dept Anesthesiol, Shiraz, Iran.
C3 Shiraz University of Medical Science; Jefferson University; Shiraz University of Medical Science; Shiraz University of Medical Science
RP Asadi-Pooya, AA (通讯作者), Shiraz Univ Med Sci, Epilepsy Res Ctr, Shiraz, Iran.
EM aliasadipooya@yahoo.com
TC 9
Z9 9
PD JUN
PY 2021
VL 143
IS 6
BP 624
EP 628
DI 10.1111/ane.13404
EA FEB 2021
WC Clinical Neurology
ER

PT J
AU Boronat, S
AF Boronat, Susana
TI Neurologic Care of COVID-19 in Children
SO FRONTIERS IN NEUROLOGY
LA English
DT Review
DE SARS-CoV-2; seizure; epilepsy; neurocognitive; behavior; stress; child abuse
ID MULTISYSTEM INFLAMMATORY SYNDROME; ADOLESCENT; LESIONS
AB Most children with SARS-CoV-2 infection have relatively mild clinical symptoms without fever or pneumonia, although severe cases with multiple-organ failure have been reported. Neurological symptoms, which have been mainly reported in adults, are very rare in children. This article will review 2 different aspects of neurological involvement related to this infection in children. In the first part, we will review the neurological abnormalities reported in children caused by this viral infection.

Adults frequently report muscle pain, headache, anosmia, dysgeusia, and occasionally more severe central or peripheral nervous system damage. Neurological involvement seems infrequent in children, although some cases have been reported. In the second part, we will discuss the COVID-19 pandemic impact on the healthcare system of some countries, causing collateral damage to general pediatric care and in particular to those children affected with chronic diseases, mainly neurological conditions, including autism, intellectual disability, attention deficit and hyperactivity disorder (ADHD), neuromuscular disorders, cerebral palsy, and epilepsy, and patients needing neurosurgical procedures.
C1 [Boronat, Susana] Hosp Santa Creu & Sant Pau, Pediat Neurol, Barcelona, Spain.
C3 Hospital of Santa Creu i Sant Pau
RP Boronat, S (通讯作者), Hosp Santa Creu & Sant Pau, Pediat Neurol, Barcelona, Spain.
EM sboronat@santpau.cat
TC 4
Z9 4
PD FEB 18
PY 2021
VL 11
AR 613832
DI 10.3389/fneur.2020.613832
WC Clinical Neurology; Neurosciences
ER

PT J
AU Mueller, TM
Kostev, K
Gollwitzer, S
Lang, JD
Stritzelberger, J
Westermayer, V
Reindl, C
Hamer, HM
AF Mueller, Tamara M.
Kostev, Karel
Gollwitzer, Stephanie
Lang, Johannes D.
Stritzelberger, Jenny
Westermayer, Vivien
Reindl, Caroline
Hamer, Hajo M.
TI The impact of the coronavirus disease (COVID-19) pandemic on outpatient epilepsy care: An analysis of physician practices in Germany
SO EPILEPSY & BEHAVIOR
LA English
DT Article
DE COVID-19; Epilepsy; care; Anti-epileptic drug
AB Objective: To gain insight into epilepsy care during coronavirus disease (COVID-19)

pandemic, we analyzed prescription data of a large cohort of persons with epilepsy (PWE) during lockdown in Germany.

Methods: Information was obtained from the Disease Analyzer database, which collects anonymous demographic and medical data from practice computer systems of general practitioners (GP) and neurologists (NL) throughout Germany. We retrospectively compared prescription data for anti-seizure medication (ASM) and physicians' notes of "known" and "new" PWE from January 2020 until May 2020 with the corresponding months in the three preceding years 2017-2019. Adherence was estimated by calculating the proportion of patients with follow-up prescriptions within 90 days after initial prescriptions in January or February. We additionally analyzed hospital referrals of PWE. The significance level was set to 0.01 to adjust for multiple comparisons.

Results: A total of 52,844 PWE were included. Anti-seizure medication prescriptions for known PWE increased in March 2020 (GP + 36%, NL + 29%; P < 0.01). By contrast, a decrease in prescriptions to known and new PWE was observed in April and significantly in May 2020 ranging from -16% to -29% (P < 0.01). The proportion of PWE receiving follow-up prescriptions was slightly higher in 2020 (73.5%) than in 2017-2019 (70.7%, P = 0.001). General practitioners and NL referred fewer PWE to hospitals in March 2020 (GP: -30%, P < 0.01; NL: -12%), April 2020 (GP: -29%, P < 0.01; NL: -37%), and May 2020 (GP: -24%, P < 0.01; NL: -16%).

Conclusion: Adherence of known PWE to ASM treatment appeared to remain stable during lockdown in Germany. However, this study revealed findings which point to reduced care for newly diagnosed PWE as well as fewer hospital admissions. These elements may warrant consideration during future lockdown situations. (C) 2021 Elsevier Inc. All rights reserved.
C1 [Mueller, Tamara M.; Gollwitzer, Stephanie; Lang, Johannes D.; Stritzelberger, Jenny; Westermayer, Vivien; Reindl, Caroline; Hamer, Hajo M.] Univ Hosp Erlangen, Epilepsy Ctr, Dept Neurol, Schwabachanlage 6, D-91054 Erlangen, Germany.

[Kostev, Karel] Main Airport Ctr, Epidemiol, IQVIA, Unterschweinstiege 2-14, D-60549 Frankfurt, Germany.

C3 University of Erlangen Nuremberg; IQVIA

RP Mueller, TM (通讯作者), Univ Hosp Erlangen, Dept Neurol, Schwabachanlage 6, D-91054 Erlangen, Germany.

EM tamara.mueller@uk-erlangen.de
TC 7

Z9 7

PD APR

PY 2021

VL 117

AR 107833

DI 10.1016/j.yebeh.2021.107833

EA FEB 2021

WC Behavioral Sciences; Clinical Neurology; Psychiatry

ER

PT J

AU Koh, MY

Lim, KS

Fong, SL
Khor, SB
Tan, CT
AF Koh, May-Yi
Lim, Kheng-Seang
Fong, Si-Lei
Khor, Si-Bao
Tan, Chong-Tin
TI Impact of COVID-19 on quality of life in people with epilepsy, and a multinational comparison of clinical and psychological impacts
SO EPILEPSY & BEHAVIOR
LA English
DT Article
DE COVID-19; Epilepsy; Seizure control; Quality of life; Depression; Anxiety
AB Background: This study aimed to determine the relationship among the clinical, logistic, and psychological impacts of COVID-19 on people with epilepsy (PWE), and the impact of COVID-19 on the quality of life.

Method: This is a cross-sectional anonymized web-based study on PWE, using an online questionnaire to assess the clinical, logistic, and psychological impacts of COVID-19, including Hospital Anxiety Depression Scale (HADS) and Quality of Life in Epilepsy Inventory (QOLIE-31).

Result: 461 patients were recruited, with a mean age of 39.21 +/- 15.88 years, majority female (50.1%), with focal epilepsy (54.0%), and experienced seizures at least once yearly (62.5%). There were 13.0% experienced seizure worsening during COVID-19 period, which were associated with baseline seizures frequency >= 1 per month (32.0% vs. 6.2%, $p < 0.001$), worries of seizure worsening (18.0% vs. 10.9%, $p < 0.001$), difficulty to go emergency unit (24.4% vs. 10.4%, $p < 0.001$), AEDs ran out of stock (23.2% vs. 11.6%, $p < 0.05$), self-adjustment of AED dosages (26.4% vs. 11.3%, $p < 0.001$), inadequate sleep (22.4% vs. 9.2%, $p < 0.001$), and stress (23.4% vs.10.1%, $p < 0.01$). Participants experiencing seizure worsening reported greater anxiety (8.10 +/- 5.011 vs. 4.84 +/- 3.989, $p < 0.001$) and depression (6.05 +/- 3.868 vs. 3.86 +/- 3.589, $p < 0.001$). Logistic regression showed baseline seizures frequency >1 per month (OR, 14.10) followed by anxiety (OR, 3.90), inadequate sleep (OR, 0.37), and treated in UMMC (OR, 0.31) as the predictors for seizure worsening during COVID-19 period. Poorer total QOLIE-31 score was noted in those with seizure worsening (48.01 +/- 13.040 vs. 62.15 +/- 15.222, $p < 0.001$). Stepwise regression highlighted depression as the main negative predictor for quality of life (beta = -0.372, $p < 0.001$), followed by anxiety (b = -0.345, $p < 0.001$).

Conclusion: A significant number of PWE experienced seizure worsening during COVID-19 period, which was related to the clinical, logistic, and psychological factors. Quality of life was affected by the seizure worsening and the psychological stress.
(C) 2021 Elsevier Inc. All rights reserved.
C1 [Koh, May-Yi; Lim, Kheng-Seang; Fong, Si-Lei; Khor, Si-Bao; Tan, Chong-Tin] Univ Malaya, Fac Med, Dept Med, Div Neurol, Kuala Lumpur, Malaysia.
C3 Universiti Malaya
RP Lim, KS (通讯作者), Univ Malaya, Med Ctr, Neurol Lab, Menara Selatan, 6th Floor, Kuala Lumpur 50603, Malaysia.

EM kslimum@gmail.com
TC 7
Z9 7
PD APR
PY 2021
VL 117
AR 107849
DI 10.1016/j.yebeh.2021.107849
EA FEB 2021
WC Behavioral Sciences; Clinical Neurology; Psychiatry
ER

PT J
AU Modi, AC
Patel, AD
Stevens, J
Smith, G
Huszti, H
Guilfoyle, SM
Mara, CA
Schmidt, M
Wagner, JL
AF Modi, Avani C.
Patel, Anup D.
Stevens, Jack
Smith, Gigi
Huszti, Heather
Guilfoyle, Shanna M.
Mara, Constance A.
Schmidt, Matthew
Wagner, Janelle L.
TI The psychosocial impact of COVID-19 within the first six months of the pandemic on youth with epilepsy and their caregivers
SO EPILEPSY & BEHAVIOR
LA English
DT Article
DE Pandemic; Adjustment; Children; Seizure; Distance learning; Self-management
AB Objectives: We assessed the impact of COVID-19 on children with epilepsy and their families, focusing on epilepsy management, family routines, learning, and adherence to Centers for Disease Control and Prevention (CDC) pandemic guidelines (e.g., social distancing, mask wearing) within the first six months of the pandemic. Group differences in COVID-19 impact on families were also examined based on race and ethnicity, being medically and/or geographically underserved, and insurance status.

Methods: Participants (n = 131) included children with epilepsy and their families from two clinical trials. The Impact of COVID-19 on Pediatric Epilepsy Management (ICPEM) measure was developed and administered to caregivers online from April 2020 to September 2020 across four large pediatric hospitals. Administration of the ICPEM occurred both

during routine study assessments and an additional acute time point to obtain information early in the pandemic (e.g., April and May 2020). Descriptive statistics and t-tests were used for analyses.

Results: Data indicate minor to moderate impact of COVID-19 on pediatric epilepsy management. Caregivers of children with epilepsy reported the most impact on education and social functioning. Adherence to CDC guidelines was reported to be high. Those having public insurance reported greater difficulties obtaining daily anti-seizure medications compared to those with private insurance.

Conclusions: This study presents important initial data regarding the impact of COVID-19 epilepsy management and daily functioning in children with epilepsy and their families. While the acute impact of COVID-19 restrictions appear to be mild to moderate, it is unclear what the long-term impact of the pandemic will be on families of children with epilepsy. (C) 2021 Elsevier Inc. All rights reserved.
C1 [Modi, Avani C.; Guilfoyle, Shanna M.; Mara, Constance A.] Cincinnati Childrens Hosp Med Ctr, Div Behav Med & Clin Psychol, 3333 Burnet Ave, Cincinnati, OH 45229 USA.

[Modi, Avani C.; Guilfoyle, Shanna M.; Mara, Constance A.] Univ Cincinnati, Coll Med, Cincinnati, OH 45221 USA.

[Patel, Anup D.; Stevens, Jack] Nationwide Childrens Hosp, 700 Childrens Dr,Near East Off Bldg,3rd Floor, Columbus, OH 43205 USA.

[Patel, Anup D.; Stevens, Jack] Ohio State, Dept Pediat, 700 Childrens Dr,Near East Off Bldg,3rd Floor, Columbus, OH 43205 USA.

[Smith, Gigi; Wagner, Janelle L.] Med Univ South Carolina, Coll Nursing, Colcock Hall,19 Bee St,MSC 002, Charleston, SC 29425 USA.

[Huszti, Heather] Childrens Hosp Orange Cty, Dept Pediat Psychol, 1120 W La Veta Ave,Ste 470, Orange, CA 92868 USA.

[Schmidt, Matthew] Univ Florida, Coll Educ, 2423 Normal Hall 2-206,POB 117048, Gainesville, FL 32611 USA.

C3 Cincinnati Children's Hospital Medical Center; University of Cincinnati; Ohio State University; Ohio State University; Medical University of South Carolina; Childrens Hospital of Orange County; State University System of Florida; University of Florida

RP Modi, AC (通讯作者)，Univ Cincinnati, Ctr Treatment Adherence & Self Management, Div Behav Med & Clin Psychol, Coll Med,Cincinnati Childrens Hosp Med Ctr, 3333 Burnet Ave MLC 7039, Cincinnati, OH 45229 USA.

EM avani.modi@cchmc.org
TC 2

Z9 2

PD APR

PY 2021

VL 117

AR 107855

DI 10.1016/j.yebeh.2021.107855

EA FEB 2021

WC Behavioral Sciences; Clinical Neurology; Psychiatry

ER

PT J

AU Grande, E
Tufo, T
Ciavarro, M
Di Muccio, I
Fuggetta, F
Silvestri, M
Bevacqua, G
Lanzone, J
Assenza, G
AF Grande, Eleonora
Tufo, Tommaso
Ciavarro, Marco
Di Muccio, Ines
Fuggetta, Filomena
Silvestri, Martina
Bevacqua, Giuseppina
Lanzone, Jacopo
Assenza, Giovanni
TI The Impact of COVID-19 Lockdown on People With Epilepsy and Vagal Nerve Stimulation
SO FRONTIERS IN NEUROLOGY
LA English
DT Article
DE epilepsy; vagal nerve stimulator; COVID-19; mental health; telemedicine
AB Objectives: Restrictive measures adopted during the COVID-19 pandemic, in order to limit contagion, have had a severe impact on mental health. The burden of lockdown has been particularly heavy on patients with chronic neurologic diseases such as People with Epilepsy (PwE). Our survey aims to describe the struggles and needs of Drug-Resistant (DR) PwE with implanted Vagal Nerve Stimulator (VNS) during the first wave of the COVID-19 lockdown in order to find strategies that help patients cope with present or future periods of restriction.

Methods: We collected answers from 30 respondents who underwent an online survey including socio-demographic and clinical information and COVID-19-related information. Depression, anxiety symptoms, and sleep quality were investigated in patients through BDI II, GAD-7, and the PSQI scale.

Results: In all, 46% of our sample reported an increase in the number of seizures; the entire sample complained of epilepsy-related issues (medication availability, VSN adjustments, anxiety, sleep disturbance); one out of three participants reported major epilepsy issues felt urgent; 30% had to postpone scheduled examination. Significantly higher scores for depression and anxiety scales were found in patients who perceived seizure frequency worsening and reported major epilepsy-related issues.

Conclusion: Preliminary findings showed that the first lockdown influenced the clinical and psychological status of PwE and was related to seizures worsening. The lack of medical assistance and control on VNS therapy left patients to cope with the situation without a chance to contact a specialist. We discuss how a wider implementation of telemedicine programs could facilitate remote assistance of PwE with a VNS implant.

C1 [Grande, Eleonora] Univ Gabriele dAnnunzio Chieti Pescara, Dept Neurosci Imaging & Clin Sci, Chieti, Italy.
[Tufo, Tommaso; Fuggetta, Filomena; Silvestri, Martina] Catholic Univ, Policlin A Gemelli Fdn Ist Ricovero & Cura Caratt, Neurosurg, Rome, Italy.
[Ciavarro, Marco] Neuromed Ist Ricovero & Cura Carattere Sci, Pozzilli, Italy.
[Di Muccio, Ines] AOSG Moscati, Neurosurg, Avellino, Italy.
[Bevacqua, Giuseppina] Sapienza Univ Rome, Dept Human Neurosci, Rome, Italy.
[Lanzone, Jacopo; Assenza, Giovanni] Univ Campus Biomed Roma, Dept Med, Neurol Neurophysiol & Neurobiol Unit, Rome, Italy.
C3 G d'Annunzio University of Chieti-Pescara; Catholic University of the Sacred Heart; IRCCS Policlinico Gemelli; IRCCS Neuromed; San Giuseppe Moscati Hospital; Sapienza University Rome; University Campus Bio-Medico - Rome Italy
RP Lanzone, J (通讯作者), Univ Campus Biomed Roma, Dept Med, Neurol Neurophysiol & Neurobiol Unit, Rome, Italy.
EM jacopo.lanzone@gmail.com
TC 4
Z9 4
PD FEB 26
PY 2021
VL 12
AR 640581
DI 10.3389/fneur.2021.640581
WC Clinical Neurology; Neurosciences
ER

PT J
AU Brambilla, I
Aibar, JA
Hallet, AS
Bibic, I
Cardenal-Munoz, E
Prpic, I
Darra, F
Specchio, N
Nabbout, R
AF Brambilla, Isabella
Aibar, Jose Angel
Hallet, Anne Sophie
Bibic, Irena
Cardenal-Munoz, Elena
Prpic, Igor
Darra, Francesca
Specchio, Nicola
Nabbout, Rima
TI Impact of the COVID-19 lockdown on patients and families with Dravet syndrome

SO EPILEPSIA OPEN
LA English
DT Article
DE behavior disorders; COVID-19; Dravet syndrome; pandemics; social isolation; telemedicine
AB We explored the impact of coronavirus virus 2019 (COVID-19) pandemic on patients with Dravet syndrome (DS) and their family. With European patient advocacy groups (PAGs), we developed an online survey in 10 languages to question health status, behavior, personal protection, and health services before and after lockdown. Approximately 538 European PAG members received electronic invitations. Survey ran from April 14, to May 17, 2020, with 219 answers; median age 9 year 10 months. Protection against infection was highly used prior to COVID-19, but 88% added facemask-use according to pandemic recommendations. Only one patient was tested positive for COVID-19. Most had stable epilepsy during lockdown, and few families (4%) needed emergency care during lockdown. However, behavior disorder worsened in over one-third of patients, regardless of epilepsy changes. Half of appointments scheduled prior to lockdown were postponed; 12 patients (11%) had appointments fulfilled; and 39 (36%) had remote consultations. Responders welcomed remote consultations. Half of responders were unsatisfied with psychological remote support as only few (21 families) received this support. None of the five of patient in clinical trials stopped investigational treatment. Prior adoption of protective measures against general infection might have contributed to avoiding COVID-19 infections. Protocols for the favored remote contact ought to now be prepared.
C1 [Brambilla, Isabella] Dravet Italia Onlus, Milan, Italy.
[Brambilla, Isabella; Darra, Francesca] Res Ctr Pediat Epilepsies Verona, Verona, Italy.
[Aibar, Jose Angel; Cardenal-Munoz, Elena] Dravet Syndrome Fdn Spain, Madrid, Spain.
[Hallet, Anne Sophie] Alliance Syndrome Dravet, Malesherbes, France.
[Bibic, Irena] Dravet Sindrom Hrvatska, Split, Croatia.
[Prpic, Igor] Univ Rijeka, Univ Hosp Rijeka, Referral Ctr Childhood Epilepsy & Convuls Disorde, Dept Child Neurol,Med Fac, Rijeka, Croatia.
[Darra, Francesca] Univ Verona, Dept Surg Sci Dent Gynecol & Pediat, Child Neuropsychiat, Verona, Italy.
[Specchio, Nicola] IRCCS, Bambino Gesu Childrens Hosp, European Reference Network EpiCARE, Rare & Complex Epilepsy Unit,Dept Neurosci, Rome, Italy.
[Nabbout, Rima] Univ Paris, Necker Enfants MaladesUniv Hosp, Ctr Reference Epilepsies Rares, Dept Pediat Neurol,Inst Imagine,U1163,EPICARE Eur, Paris, France.
C3 University of Rijeka; University of Verona; IRCCS Bambino Gesu; UDICE-French Research Universities; Universite de Paris
RP Nabbout, R (通讯作者), Necker Enfants Malad Hosp, Reference Ctr Rare Epilepsies, Dept Pediat Neurol, 149 Rue Sevres, F-75015 Paris, France.
EM rimanabbout@yahoo.com
TC 4
Z9 4
PD MAR
PY 2021
VL 6

IS 1
BP 216
EP 224
DI 10.1002/epi4.12464
WC Clinical Neurology; Neurosciences
ER

PT J
AU Sandoval, F
   Julio, K
   Mendez, G
   Valderas, C
   Echeverria, AC
   Perinetti, MJ
   Suarez, NM
   Barraza, G
   Pinera, C
   Alarcon, M
   Samaniego, F
   Quesada-Rios, P
   Robles, C
   Izquierdo, G
AF Sandoval, Francisca
   Julio, Katherine
   Mendez, Gaston
   Valderas, Carolina
   Echeverria, Alejandra C.
   Perinetti, Maria Jose
   Suarez, N. Mario
   Barraza, Gonzalo
   Pinera, Cecilia
   Alarcon, Macarena
   Samaniego, Fernando
   Quesada-Rios, Pia
   Robles, Carlos
   Izquierdo, Giannina
TI Neurologic Features Associated With SARS-CoV-2 Infection in Children: A
   Case Series Report
SO JOURNAL OF CHILD NEUROLOGY
LA English
DT Article
DE neurologic; children; SARS-CoV-2; COVID-19; multiple sclerosis; encephalopathy
AB Introduction:
   Although multiple neurologic manifestations associated with SARS-CoV-2 infection have been described in adults, there is little information about those presented in children. Here, we described neurologic manifestations associated with COVID-19 in the pediatric population.

Methods:
Retrospective case series report. We included patients younger than 18 years, admitted with confirmed SARS-CoV-2 infection and neurologic manifestations at our hospital in Santiago, Chile. Demographics, clinical presentations, laboratory results, radiologic and neurophysiological studies, treatment, and outcome features were described. Cases were described based on whether they presented with predominantly central or peripheral neurologic involvement.
Results:
Thirteen of 90 (14.4%) patients admitted with confirmed infection presented with new-onset neurologic symptoms and 4 patients showed epilepsy exacerbation. Neurologic manifestations ranged from mild (headache, muscle weakness, anosmia, ageusia), to severe (status epilepticus, Guillain-Barre syndrome, encephalopathy, demyelinating events).
Conclusions:
We found a wide range of neurologic manifestations in children with confirmed SARS-CoV-2 infection. In general, neurologic symptoms were resolved as the systemic presentation subsided. It is essential to recognize and report the main neurologic manifestations related to this new infectious disease in the pediatric population. More evidence is needed to establish the specific causality of nervous system involvement.
C1 [Sandoval, Francisca; Julio, Katherine; Mendez, Gaston; Valderas, Carolina; Echeverria, Alejandra C.; Perinetti, Maria Jose; Suarez, N. Mario; Alarcon, Macarena; Samaniego, Fernando; Quesada-Rios, Pia] Hosp Dr Exequiel Gonzalez Cortes, Dept Neurol, Gran Ave Jose Miguel Carrera 3300, Santiago, Region Metropol, Chile.
[Barraza, Gonzalo; Alarcon, Macarena] Univ Santiago Chile, Fac Med, Santiago, Chile.
[Barraza, Gonzalo] Hosp Dr Exequiel Gonzalez Cortes, Electromyog & Evoked Potentials Unit, Santiago, Region Metropol, Chile.
[Pinera, Cecilia; Izquierdo, Giannina] Hosp Dr Exequiel Gonzalez Cortes, Infect Dis Unit, Santiago, Region Metropol, Chile.
[Pinera, Cecilia; Izquierdo, Giannina] Univ Chile, Fac Med, Santiago, Chile.
[Robles, Carlos] Hosp Dr Exequiel Gonzalez Cortes, Dept Radiol, Santiago, Region Metropol, Chile.
C3 Universidad de Chile; Universidad de Santiago de Chile; Universidad de Chile; Universidad de Chile; Universidad de Chile; Universidad de Chile
RP Sandoval, F (通讯作者), Hosp Dr Exequiel Gonzalez Cortes, Dept Neurol, Gran Ave Jose Miguel Carrera 3300, Santiago, Region Metropol, Chile.
EM francisca.sandoval.alvarez@gmail.com
TC 9
Z9 9
PD SEP
PY 2021
VL 36
IS 10
BP 853
EP 866
AR 0883073821989164
DI 10.1177/0883073821989164
EA MAR 2021

WC Clinical Neurology; Pediatrics
ER

PT J
AU Rathore, C
Baheti, N
Bansal, AR
Jabeen, SA
Gopinath, S
Jagtap, S
Patil, S
Suryaprabha, T
Jayalakshmi, S
Ravat, S
Nayak, DS
Prakash, S
Rana, K
Jaiswal, SK
Khan, FR
Murthy, JM
Radhakrishnan, K
AF Rathore, Chaturbhuj
Baheti, Neeraj
Bansal, Atma Ram
Jabeen, Shaik Afshan
Gopinath, Siby
Jagtap, Sujit
Patil, Sandeep
Suryaprabha, Turaga
Jayalakshmi, Sita
Ravat, Sangeeta
Nayak, Dinesh S.
Prakash, Sanjay
Rana, Kaushik
Jaiswal, Shyam K.
Khan, Fayaz R.
Murthy, Jagarlapudi Mk
Radhakrishnan, Kurupath
TI Impact of COVID-19 pandemic on epilepsy practice in India: A tripartite survey
SO SEIZURE-EUROPEAN JOURNAL OF EPILEPSY
LA English
DT Article
DE COVID-19; Lockdown; Epilepsy; Teleconsultation
ID TELEMEDICINE; ACADEMY; CARE
AB Objective: To assess the impact of ongoing COVID-19 pandemic on epilepsy care in India.

Methods: We conducted a three-part survey comprising neurologists, people with epilepsy (PWE), and 11 specialized epilepsy centers across India. We sent two separate online survey questionnaires to Indian neurologists and PWE to assess the epilepsy practice, seizures control, and access to care during the COVID-19 pandemic. We collected and compared the data concerning the number of PWE cared for and epilepsy procedures performed during the 6 months periods preceding and following COVID-19 lockdown from epilepsy centers.

Results: The survey was completed by 453 neurologists and 325 PWE. One third of the neurologist reported >50 % decline in outdoor visits by PWE and EEG recordings. The cumulative data from 11 centers showed 65-70 % decline in the number of outdoor patients, video-EEG monitoring, and epilepsy surgery. Working in a hospital admitting COVID-19 patients and use of teleconsultation correlated with this decline. Half of PWE had postponed their planned outpatient visits and EEG. Less than 10 % of PWE missed their antiseizure medicines (ASM) or had seizures due to the nonavailability of ASM. Seizure control remained unchanged or improved in 92 % PWE. Half of the neurologists started using teleconsultation during the pandemic. Only 4% of PWE were afflicted with COVID-19 infection. Conclusions: Despite significant decline in the number of PWE visiting hospitals, their seizure control and access to ASMs were not affected during the COVID-19 pandemic in India. Risk of COVID-19 infection in PWE is similar to general population.
C1 [Rathore, Chaturbhuj; Prakash, Sanjay; Rana, Kaushik] Sumandeep Vidyapeeth, Dept Neurol, Smt BK Shah Med Inst & Res Ctr, Vadodara 391760, Gujarat, India.

[Baheti, Neeraj] Dr GM Taori Cent India Inst Med Sci, Dept Neurol, Nagpur, Maharashtra, India.

[Bansal, Atma Ram] Medanta Medicity Hosp, Dept Neurol, Gurugram, India.

[Jabeen, Shaik Afshan; Suryaprabha, Turaga] Nizams Inst Med Sci, Dept Neurol, Hyderabad, India.

[Gopinath, Siby] Amrita Inst Med Sci, Dept Neurol, Kochi, Kerala, India.

[Jagtap, Sujit; Patil, Sandeep] Deenanath Mangeshkar Hosp & Res Ctr, Dept Neurol, Pune, Maharashtra, India.

[Jagtap, Sujit; Patil, Sandeep] Bharati Vidyapeeth Med Coll, Pune, Maharashtra, India.

[Jayalakshmi, Sita] Krishna Inst Med Sci, Dept Neurol, Hyderabad, India.

[Ravat, Sangeeta] Seth GS Med Coll, Dept Neurol, Mumbai, Maharashtra, India.

[Ravat, Sangeeta] KEM Hosp Murnbai, Mumbai, Maharashtra, India.

[Nayak, Dinesh S.] Gleneagles Global Hlth City, Dept Neurol, Chennai, Tamil Nadu, India.

[Jaiswal, Shyam K.; Murthy, Jagarlapudi Mk] Care Hosp, Dept Neurol, Hyderabad, India.

[Khan, Fayaz R.] King Abdulaziz Univ, Fac Med Rehabil Sci, Dept Phys Therapy, Jeddah, Saudi Arabia.

[Radhakrishnan, Kurupath] Avitis Inst Med Sci, Dept Neurol, Nemmara, Kerala, India.

C3 Sumandeep Vidyapeeth; Nizam's Institute of Medical Sciences; Amrita Vishwa Vidyapeetham; Amrita Vishwa Vidyapeetham Kochi; Bharati Vidyapeeth Deemed University; Seth Gordhandas Sunderdas Medical College & King Edward Memorial Hospital; King Abdulaziz University

RP Rathore, C (通讯作者), Sumandeep Vidyapeeth, Dept Neurol, Smt BK Shah Med Inst &

Res Ctr, Vadodara 391760, Gujarat, India.
EM cbrathore@gmail.com
TC 2
Z9 2
PD MAR
PY 2021
VL 86
BP 60
EP 67
DI 10.1016/j.seizure.2020.12.025
WC Clinical Neurology; Neurosciences
ER

PT J
AU Leone, M
   Ciccacci, F
   Orlando, S
   Petrolati, S
   Guidotti, G
   Majid, NA
   Tolno, VT
   Sagno, J
   Thole, D
   Corsi, FM
   Bartolo, M
   Marazzi, MC
AF Leone, Massimo
   Ciccacci, Fausto
   Orlando, Stefano
   Petrolati, Sandro
   Guidotti, Giovanni
   Majid, Noorjehan Abdul
   Tolno, Victor Tamba
   Sagno, JeanBaptiste
   Thole, Darlington
   Corsi, Fabio Massimo
   Bartolo, Michelangelo
   Marazzi, Maria Cristina
TI Pandemics and Burden of Stroke and Epilepsy in Sub-Saharan Africa:
   Experience from a Longstanding Health Programme
SO INTERNATIONAL JOURNAL OF ENVIRONMENTAL RESEARCH AND PUBLIC HEALTH
LA English
DT Review
DE sub-Saharan Africa; stroke; epilepsy; HIV; AIDS; COVID-19; medical
   education; retention; pandemic; care disruption; DREAM programme
ID MIDDLE-INCOME COUNTRIES; ANTIEPILEPTIC DRUGS; MALAWIAN ADULTS;
   RISK-FACTORS; HYPERTENSION; EXPENDITURE; MORTALITY; SERVICES; HIV;

IMPOVERISHMENT
AB Eighty percent of people with stroke live in low- to middle-income nations, particularly in sub-Saharan Africa (SSA) where stroke has increased by more than 100% in the last decades. More than one-third of all epilepsy-related deaths occur in SSA. HIV infection is a risk factor for neurological disorders, including stroke and epilepsy. The vast majority of the 38 million people living with HIV/AIDS are in SSA, and the burden of neurological disorders in SSA parallels that of HIV/AIDS. Local healthcare systems are weak. Many standalone HIV health centres have become a platform with combined treatment for both HIV and noncommunicable diseases (NCDs), as advised by the United Nations. The COVID-19 pandemic is overwhelming the fragile health systems in SSA, and it is feared it will provoke an upsurge of excess deaths due to the disruption of care for chronic diseases such as HIV, TB, hypertension, diabetes, and cerebrovascular disorders. Disease Relief through Excellent and Advanced Means (DREAM) is a health programme active since 2002 to prevent and treat HIV/AIDS and related disorders in 10 SSA countries. DREAM is scaling up management of NCDs, including neurologic disorders such as stroke and epilepsy. We described challenges and solutions to address disruption and excess deaths from these diseases during the ongoing COVID-19 pandemic.
C1 [Leone, Massimo] Fdn Carlo Besta IRCCS Neurol Inst, I-20133 Milan, Italy.
[Ciccacci, Fausto] Univ Hlth Sci, UniCamillus St Camillus Int, I-00100 Rome, Italy.
[Orlando, Stefano] Univ Roma Tor Vergata, I-00100 Rome, Italy.
[Petrolati, Sandro] San Camillo Hosp, Dept Cardiosci, I-00100 Rome, Italy.
[Guidotti, Giovanni] Azienda Sanit Locale ASL Roma 1 Reg Lazio, I-00100 Rome, Italy.
[Majid, Noorjehan Abdul] Community S Egidio DREAM Program, Maputo 1102, Mozambique.
[Tolno, Victor Tamba; Sagno, JeanBaptiste] Community S Egidio DREAM Program, Blantyre 312224, Malawi.
[Thole, Darlington] Community S Egidio DREAM Program, Balaka 302100, Malawi.
[Corsi, Fabio Massimo] Salvator Mundi Int Hosp Neurol, I-00100 Rome, Italy.
[Bartolo, Michelangelo] San Giovanni Addolorata Hosp, Telemed Dept, I-00100 Rome, Italy.
[Marazzi, Maria Cristina] Libera Univ Maria SS Assunta, I-00100 Rome, Italy.
C3 University of Rome Tor Vergata; Azienda Ospedaliera San Giovanni Addolorata; Universita LUMSA
RP Leone, M (通讯作者), Fdn Carlo Besta IRCCS Neurol Inst, I-20133 Milan, Italy.
EM massimo.leone@istituto-besta.it; fausto.ciccacci@gmail.com; stefano.orlando@dreameurope.org; sandropetrolati@gmail.com; giovanni.guidotti@dreamsantegidio.net; noorjehanmagid@dream.org.mz; tolnovictortamba@gmail.com; sagnojb@gmail.com; darlthole@gmail.com; fmcorsi@gmail.com; mbartolo@hsangiovanni.roma.it; mcmarazzi@gmail.com
TC 3
Z9 3
PD MAR
PY 2021
VL 18
IS 5
AR 2766
DI 10.3390/ijerph18052766

WC Environmental Sciences; Public, Environmental & Occupational Health
ER

PT J
AU Nair, PP
   Aghoram, R
   Thomas, B
   Bharadwaj, B
   Chinnakali, P
AF Nair, Pradeep Pankajakshan
   Aghoram, Rajeswari
   Thomas, Bitty
   Bharadwaj, Balaji
   Chinnakali, Palanivel
TI Video teleconsultation services for persons with epilepsy during
   COVID-19 pandemic: An exploratory study from public tertiary care
   hospital in Southern India on feasibility, satisfaction, and
   effectiveness
SO EPILEPSY & BEHAVIOR
LA English
DT Article
DE Epilepsy; Synchronous methods; Mobile video teleconsultation;
   Feasibility; Acceptability; Effectiveness
AB Purpose: During the COVID-19 pandemic, there is a large unmet need for follow-up services, particularly for chronic diseases such as epilepsy. Alternative methods to reach these people have become necessary. We assessed the feasibility, satisfaction, and effectiveness of video teleconsultation using mobile phones for managing persons with epilepsy (PWEs) on follow-up at a tertiary care center in the southern part of India.

   Patients and methods: We included PWEs aged 18 years and over who have been evaluated in person within the past six months, with details available in electronic health records (EHRs), and advised regular follow-up after getting telephonic consent. We excluded those requiring emergency care and those seeking teleconsultation for new symptoms. Participants were sent a message in English and in the local language about the possibility of a video teleconsultation. If willing, they were informed about the date, time, and technical requirements such as smartphones, browsing facilities, etc. Feasibility and effectiveness were assessed. Satisfaction/acceptability was assessed using Telemedicine Satisfaction Questionnaire.

   Results: From June 2020 to October 2020, we selected 336 PWEs after screening 1100 records, and we tried video teleconsultation in 141 (41.8%) PWEs. We achieved successful video connections in 95 (28.2%) and audio consultations in 46 (13.6%). The median duration for calling the participants, making successful connections, and consultation was 8 (5-14) min. The majority required two (47.4%) or three (32.6%) attempts. Sixty-five PWEs (68.4%) used caretaker's mobile phones. We gave prescriptions to all, and 18 received new drugs. Out of 95 PWEs, 90% either 'agreed' or 'strongly agreed' on 12 out of 14 telemedicine satisfaction questions.

   Conclusion: Although we need to make video teleconsultation more feasible, our

synchronous mobile video teleconsultation model is an effective and acceptable method to follow up PWEs. This real-time model has the advantage that it does not require any mobile application to be downloaded and installed. Further studies are needed to evaluate methods to improve the reach of these services particularly to vulnerable groups of the population. (C) 2021 Elsevier Inc. All rights reserved.
C1 [Nair, Pradeep Pankajakshan; Aghoram, Rajeswari; Thomas, Bitty] Jawaharlal Inst Postgrad Med Educ & Res JIPMER, Neurol, Pondicherry, India.
[Bharadwaj, Balaji] Jawaharlal Inst Postgrad Med Educ & Res JIPMER, Dept Psychiat, Pondicherry, India.
[Chinnakali, Palanivel] Jawaharlal Inst Postgrad Med Educ & Res JIPMER, Dept Prevent & Social Med, Pondicherry, India.
C3 Jawaharlal Institute of Postgraduate Medical Education & Research;
Jawaharlal Institute of Postgraduate Medical Education & Research;
Jawaharlal Institute of Postgraduate Medical Education & Research
RP Nair, PP (通讯作者), Jawaharlal Inst Postgrad Med Educ & Res JIPMER, Neurol, Pondicherry, India.
EM drpradeeppnair17@gmail.com
TC 1
Z9 1
PD APR
PY 2021
VL 117
AR 107863
DI 10.1016/j.yebeh.2021.107863
EA MAR 2021
WC Behavioral Sciences; Clinical Neurology; Psychiatry
ER

PT J
AU D'Orsi, G
Mazzeo, F
Ravida, D
Di Claudio, MT
Sabetta, A
Lalla, A
Sbrizzi, S
Avolio, C
AF D'Orsi, Giuseppe
Mazzeo, Francesca
Ravida, Domenico
Di Claudio, Maria Teresa
Sabetta, Annarita
Lalla, Alessandra
Sbrizzi, Stefania
Avolio, Carlo
TI The effect of quarantine due to Covid-19 pandemic on seizure frequency
in 102 adult people with epilepsy from Apulia and Basilicata regions,

Southern Italy
SO CLINICAL NEUROLOGY AND NEUROSURGERY
LA English
DT Article
DE Quarantine; COVID-19 infection; Epilepsy; Seizure frequency; Stress
AB Objective: following the COVID-19 pandemic, a quarantine was imposed to all of regions Italy by 9th March until 3rd May 2020. We investigated the effect of COVID-19 infection and quarantine on seizure frequency in adult people with epilepsy (PwE) of Apulia and Basilicata regions, Southern Italy.

Methods: This is an observational, retrospective study based on prospective data collection of 102 successive PWE. The frequency of seizures was evaluated during pre-quarantine (January- February), quarantine (March?April), and post?quarantine period (May-June), while PwE were divided into A) cases responding to treatment with < 1 seizure per year; B) cases responding to treatment with 2?5 seizure per year; C) cases with drugresistant epilepsy with < 4 seizures per month; D) cases with drug-resistant epilepsy with 5?10 seizures per month. PwE underwent several self-report questionnaires regarding therapeutic compliance, mood, stress and sleep during quarantine period.

Results: Approximately 50 % of PwE showed seizure frequency changes (22.55 % an increase and 27.45 % a reduction) during quarantine. Seizure frequency significantly (p < 0.05) increased in PwE responding to treatment with < 1 seizure per year, while significantly (p < 0.05) reduced in PwE with drug-resistant epilepsy with 5?10 seizures per month. The data was not influenced by therapeutic adherence, sleep and depression. The analysis of anxiety showed a moderate level of anxiety in PwE responding to treatment with < 1 seizure per year, while moderate stress was perceived by all PwE. Seizure frequency changes were related to quarantine, but not to COVID-19 infection. In fact, unlike other regions of Italy, particularly Northern Italy, Apulia and Basilicata regions were less affected by COVID-19 infection, and almost all PwE recognized the quarantine as a stressful event. Emotional distress and anxiety due to social isolation, but also the relative reduction of triggers for epileptic seizures were the most important factors for changes in seizure frequency.

Conclusions: Our study adds to the growing concern that the indirect effects of COVID-19 pandemic will far outstrip the direct consequences of the infection.
C1 [D'Orsi, Giuseppe; Mazzeo, Francesca; Di Claudio, Maria Teresa; Sabetta, Annarita; Lalla, Alessandra; Avolio, Carlo] SC Neurol Univ, Policlin Riuniti, Epilepsy Ctr, Foggia, Italy.

[Ravida, Domenico] Ente Osped Cantonale, Ist Pediatr Svizzera Italiana, Bellinzona, Switzerland.

[Sbrizzi, Stefania] Univ Foggia, Dept Phys Med & Rehabil, Policlin Riuniti, Foggia, Italy.
C3 University of Foggia
RP D'Orsi, G (通讯作者), SC Neurol Univ, Dept Neurol Sci, Epilepsy Ctr, Policlin Riuniti, Via Luigi Pinto 1, I-71100 Foggia, Italy.
EM giudorsi@yahoo.it
TC 3
Z9 3
PD APR

PY 2021
VL 203
AR 106592
DI 10.1016/j.clineuro.2021.106592
EA MAR 2021
WC Clinical Neurology; Surgery
ER

PT J
AU Asadi-Pooya, AA
Nezafat, A
Sadeghian, S
Shahisavandi, M
Nabavizadeh, SA
Barzegar, Z
AF Asadi-Pooya, Ali A.
Nezafat, Abdullah
Sadeghian, Saeid
Shahisavandi, Mina
Nabavizadeh, Seyed Ali
Barzegar, Zohreh
TI Mask Wearing Hesitancy During the COVID-19 Pandemic in South Iran
SO DISASTER MEDICINE AND PUBLIC HEALTH PREPAREDNESS
LA English
DT Article; Early Access
DE coronavirus; COVID-19; epilepsy; mask; seizure
AB Purpose: The aim of the current study was to investigate the prevalence of face mask wearing among different groups of people in south Iran. We also investigated the associations between mask wearing hesitancy and various factors. Methods: We surveyed a sample (convenience sampling) of 5 groups of people: general population, people with epilepsy, people with diabetes mellitus (DM), people with cardiac problems, and people with psychiatric problems. The survey included 4 general questions (age, sex, education, and medical/psychiatric problem) and 4 coronavirus disease 2019 (COVID-19)-specific questions (contracting COVID-19, relatives with COVID-19, wearing a face mask while in crowded places, and the frequency of daily hand washings). Results: A total of 582 people (153 people with epilepsy, 127 patients with DM, 98 people with cardiac problems, 96 patients with psychiatric disorders, and 108 healthy individuals) participated. Twenty-eight (4.8%) people expressed that they do not wear a face mask when at crowded places. A lower education and less frequent daily hand washings had associations with mask wearing hesitancy. Conclusions: Mask wearing hesitancy is a concern during a respiratory viral disease pandemic. Paying attention to personal variables, especially if they are modifiable (eg, education and hygiene), is probably productive and practical in promoting mask wearing culture.
C1 [Asadi-Pooya, Ali A.; Nezafat, Abdullah; Shahisavandi, Mina; Nabavizadeh, Seyed Ali; Barzegar, Zohreh] Shiraz Univ Med Sci, Epilepsy Res Ctr, Shiraz, Iran.
[Asadi-Pooya, Ali A.] Thomas Jefferson Univ, Dept Neurol, Jefferson Comprehens Epilepsy Ctr, Philadelphia, PA 19107 USA.

[Sadeghian, Saeid] Ahvaz Jundishapur Univ Med Sci, Goiestan Med Educ & Res Ctr, Dept Pediat Neurol, Ahvaz, Iran.
C3 Shiraz University of Medical Science; Jefferson University; Ahvaz Jundishapur University of Medical Sciences (AJUMS)
RP Asadi-Pooya, AA (通讯作者), Shiraz Univ Med Sci, Epilepsy Res Ctr, Shiraz, Iran.; Asadi-Pooya, AA (通讯作者), Thomas Jefferson Univ, Dept Neurol, Jefferson Comprehens Epilepsy Ctr, Philadelphia, PA 19107 USA.
EM aliasadipooya@yahoo.com
TC 1
Z9 1
AR PII S1935789321000720
DI 10.1017/dmp.2021.72
EA MAR 2021
WC Public, Environmental & Occupational Health
ER

PT J
AU Neshige, S
Aoki, S
Shishido, T
Morino, H
Iida, K
Maruyama, H
AF Neshige, Shuichiro
Aoki, Shiro
Shishido, Takeo
Morino, Hiroyuki
Iida, Koji
Maruyama, Hirofumi
TI Socio-economic impact on epilepsy outside of the nation-wide COVID-19 pandemic area
SO EPILEPSY & BEHAVIOR
LA English
DT Article
DE Depression; Epidemic; Insomnia; Psychiatric nonepileptic seizure; Social isolation
ID RISK; SEIZURES; STRESS; PEOPLE; LIFE
AB Objective: To identify people with epilepsy (PWE) who required extensive care before the novel coronavirus disease 2019 (COVID-19) pandemic that had world-wide impacts on medical care and on socioeconomic conditions.

Methods: Consecutive PWE who were treated at the epilepsy center of Hiroshima University Hospital, which was located in the COVID-19 non-pandemic area, between March 2019 and August 2020 were enrolled. We evaluated clinical and socioeconomic factors that were associated with seizure exacerbation (an increase in seizure frequency) during the first 6 months after the COVID-19 pandemic started compared with the previous 6 months.

Results: Among the 196 PWE who were evaluated (mean age was 37.8 +/- 16.2 years),

there were 33 PWE (16.8%) whose seizure frequency had increased after the pandemic began. People with epilepsy with a seizure increase showed a significant association with living alone (p < 0.001), a higher seizure frequency (p < 0.001), negative findings on MRI (p = 0.020), history of dissociative seizure (p < 0.001), mood disorders (p < 0.001), insomnia (p < 0.001), and high psychological stress levels (p = 0.024) at baseline compared with PWE without seizure exacerbation. Multivariate logistic regression analysis revealed that "living alone" (odds ratio (OR) 3.69; 95%CI 1.29-10.52), "high seizure frequency at baseline" (OR 4.53; 95%CI 1.63-12.57), and "comorbidity of insomnia" (OR 9.55; 95%CI 3.71-24.55) were independently associated with seizure exacerbation.

Conclusions: Even in the non-pandemic area, PWE had seizure exacerbation, suggesting that clinicians should screen patients' mental health before the outbreak to provide care, reduce the burden, and prevent social isolation in PWE. This should be addressed particularly in patients with medically refractory seizures with insomnia who live alone. (C) 2021 Elsevier Inc. All rights reserved.
C1 [Neshige, Shuichiro; Aoki, Shiro; Shishido, Takeo; Morino, Hiroyuki; Maruyama, Hirofumi] Hiroshima Univ, Dept Clin Neurosci & Therapeut, Grad Sch Biomed & Hlth Sci, Hiroshima, Japan.

[Neshige, Shuichiro; Iida, Koji; Maruyama, Hirofumi] Hiroshima Univ Hosp, Epilepsy Ctr, Hiroshima, Japan.

[Iida, Koji] Hiroshima Univ, Grad Sch Biomed & Hlth Sci, Dept Neurosurg, Hiroshima, Japan.

C3 Hiroshima University; Hiroshima University; Hiroshima University

RP Neshige, S (通讯作者), Hiroshima Univ, Grad Sch Biomed & Hlth Sci, Dept Clin Neurosci & Therapeut, Minami Ku, 1-2-3 Kasumi, Hiroshima 7348551, Japan.

EM s-neshige@hiroshima-u.ac.jp
TC 3

Z9 3

PD APR

PY 2021

VL 117

AR 107886

DI 10.1016/j.yebeh.2021.107886

EA MAR 2021

WC Behavioral Sciences; Clinical Neurology; Psychiatry

ER

PT J

AU Manganotti, P
Furlanis, G
Ajcevic, M
Moras, C
Bonzi, L
Pesavento, V
Stella, AB

AF Manganotti, Paolo
Furlanis, Giovanni

Ajcevic, Milos
Moras, Cristina
Bonzi, Lucia
Pesavento, Valentina
Buoite Stella, Alex
TI Intravenous immunoglobulin response in new-onset refractory status epilepticus (NORSE) COVID-19 adult patients
SO JOURNAL OF NEUROLOGY
LA English
DT Article
DE COVID-19; NORSE; Intravenous immunoglobulin; Epilepsy; Encephalitis
ID NMDA RECEPTOR ENCEPHALITIS
AB Neurological manifestations may be common in COVID-19 patients. They may include several syndromes, such as a suggested autoimmune abnormal response, which may result in encephalitis and new-onset refractory status epilepticus (NORSE). Quickly recognizing such cases and starting the most appropriate therapy is mandatory due to the related rapid worsening and bad outcomes. This case series describes two adult patients admitted to the university hospital and positive to novel coronavirus 2019 (SARS-CoV-2) infection who developed drug-resistant status epilepticus. Both patients underwent early electroencephalography (EEG) assessment, which showed a pathological EEG pattern characterized by general slowing, rhythmic activity and continuous epileptic paroxysmal activity. A suspected autoimmune etiology, potentially triggered by SARS-CoV-2 infection, encouraged a rapid work-up for a possible autoimmune encephalitis diagnosis. Therapeutic approach included the administration of 0.4 g/kg intravenous immunoglobulin, which resulted in a complete resolution of seizures after 5 and after 10 days, respectively, without adverse effects and followed by a normalization of the EEG patterns.
C1 [Manganotti, Paolo; Furlanis, Giovanni; Ajcevic, Milos; Buoite Stella, Alex] Univ Trieste, Clin Unit Neurol, Dept Med Surg & Hlth Sci, Trieste Univ Hosp ASUGI, Str Fiume, I-44734149 Trieste, Italy.
[Ajcevic, Milos] Univ Trieste, Dept Engn & Architecture, Via Alfonso Valerio 10, Trieste, Italy.
[Moras, Cristina] Univ Trieste, Trieste Univ Hosp ASUGI, Dept Med Surg & Hlth Sci, Unit Internal Med, Str Fiume, I-44734149 Trieste, Italy.
[Bonzi, Lucia; Pesavento, Valentina] Univ Trieste, Trieste Univ Hosp ASUGI, Rehabil Unit, Dept Med Surg & Hlth Sci, Via Giuseppe Lorenzo Gatteri 25-1, Trieste, Italy.
C3 University of Trieste; University of Trieste; University of Trieste; University of Trieste
RP Manganotti, P (通讯作者), Univ Trieste, Clin Unit Neurol, Dept Med Surg & Hlth Sci, Trieste Univ Hosp ASUGI, Str Fiume, I-44734149 Trieste, Italy.
EM pmanganotti@units.it
TC 11
Z9 11
PD OCT
PY 2021
VL 268
IS 10

BP 3569
EP 3573
DI 10.1007/s00415-021-10468-y
EA MAR 2021
WC Clinical Neurology
ER

PT J
AU Puteikis, K
Mameniskiene, R
AF Puteikis, Kristijonas
Mameniskiene, Ruta
TI Epilepsy care and COVID-19: A cross-sectional online survey from Lithuania
SO ACTA NEUROLOGICA SCANDINAVICA
LA English
DT Editorial Material
DE COVID-19; electroencephalography; epilepsy; lockdown; telehealth; vaccine
AB Background: Changes in epilepsy care during the COVID-19 pandemic required to reassess the patient-specialist interaction in the context of telehealth and future vaccination campaigns.

Aims of the study: The aims were to outline changes in neurologists' experience when providing care for patients with epilepsy (PWE) and to investigate how neurologists perceive telehealth and vaccination.

Methods: We conducted an anonymous cross-sectional online survey among members of the Lithuanian Association of Neurology.

Results: We received 104 completed forms by adult (74, 71.15%) and pediatric neurologists (30, 28.85%). A decrease in epilepsy consultations was noted by 76 (73.1%) specialists, and up to 26 (25.0%) could not provide diagnostic tests at a usual rate. Most respondents (99, 95.2%) would recommend the COVID-19 vaccine for patients at risk. Telehealth was valued as a useful tool in epilepsy care, especially if combined with timely diagnostic and treatment options (Kruskal-Wallis chi-square = 10.392, $p = .034$ and $F[4,99] = 3.125$, $p = .018$, respectively). According to 85 (81.7%) respondents, video calls could substitute in-person visits in at least half of all consultations.

Conclusions: Despite disrupted epilepsy care, neurologists may benefit from telehealth when providing services for PWE and become vaccination advocates to mitigate the spread of preventable infections.
C1 [Puteikis, Kristijonas] Vilnius Univ, Fac Med, Vilnius, Lithuania.
[Mameniskiene, Ruta] Vilnius Univ, Ctr Neurol, Santariskiu 2, LT-08661 Vilnius, Lithuania.
C3 Vilnius University; Vilnius University
RP Mameniskiene, R (通讯作者), Vilnius Univ, Ctr Neurol, Santariskiu 2, LT-08661 Vilnius, Lithuania.
EM ruta.mameniskiene@santa.lt
TC 1
Z9 1
PD JUN

PY 2021
VL 143
IS 6
BP 666
EP 672
DI 10.1111/ane.13409
EA MAR 2021
WC Clinical Neurology
ER

PT J
AU Dono, F
   Nucera, B
   Lanzone, J
   Evangelista, G
   Rinaldi, F
   Speranza, R
   Troisi, S
   Tinti, L
   Russo, M
   Di Pietro, M
   Onofrj, M
   Bonanni, L
   Assenza, G
   Vollono, C
   Anzellotti, F
   Brigo, F
AF Dono, Fedele
   Nucera, Bruna
   Lanzone, Jacopo
   Evangelista, Giacomo
   Rinaldi, Fabrizio
   Speranza, Rino
   Troisi, Serena
   Tinti, Lorenzo
   Russo, Mirella
   Di Pietro, Martina
   Onofrj, Marco
   Bonanni, Laura
   Assenza, Giovanni
   Vollono, Catello
   Anzellotti, Francesca
   Brigo, Francesco
TI Status epilepticus and COVID-19: A systematic review
SO EPILEPSY & BEHAVIOR
LA English
DT Review

DE Epilepsy; Status epilepticus; SARS-CoV-2; infection; Pandemic
ID NONCONVULSIVE STATUS EPILEPTICUS; REFRACTORY STATUS EPILEPTICUS;
CORONAVIRUS DISEASE; EEG FINDINGS; INFECTION; PATIENT; PAY
AB Purpose: In March 2020, the World Health Organization declared the SARS-CoV-2 infection-related coronavirus Disease (COVID-19) a pandemic. During the first and second waves of the pandemic spread, there have been several reports of COVID-19-associated neurological manifestations, including acute seizures and status epilepticus (SE). In this systematic review, we summarized the available data on clinical features, diagnosis, and therapy of COVID-19-related SE.

Methods: We performed a systematic search of the literature to identify data on demographics, clinical, neurophysiological, and neuroradiological data of patients with COVID-19-related SE. We used regression models (linear or logistic) with a stepwise forward method to identify features associated with mortality or severity of SE.

Results: Thirty-nine articles were included with a total of 47 cases of SE associated with COVID-19. Age, time between the acute respiratory phase of SARS-CoV-2 infection and SE onset, and hospitalization correlated with a higher SE severity as assessed by quantitative validated scales.

Conclusions: SE can be a neurological manifestation of SARS-CoV-2 infection. Although a possible association between SE and COVID-19 has been reported, the exact mechanisms are still not fully understood. Systemic inflammatory syndrome due to cytokine release could play a role in COVID-19-related SE. (C) 2021 Elsevier Inc. All rights reserved.
C1 [Dono, Fedele; Evangelista, Giacomo; Speranza, Rino; Russo, Mirella; Di Pietro, Martina; Onofrj, Marco; Bonanni, Laura] G DAnnunzio Univ Chieti Pescara, Dept Neurosci Imaging & Clin Sci, Chieti, Italy.

[Nucera, Bruna; Rinaldi, Fabrizio; Brigo, Francesco] Hosp Merano SABES ASDAA, Dept Neurol, Merano, Italy.

[Lanzone, Jacopo] Fdn Europea Ric Biomed Onlus, Trescore Balneario, Italy.

[Troisi, Serena] Santobono Pausilipon Childrens Hosp, Dept Neurosci, Pediat Neurol, Naples, Italy.

[Tinti, Lorenzo] San Gerardo Hosp, Dept Neurol, Monza, Italy.

[Assenza, Giovanni] Univ Campus Biomed Rome, Dept Med, Unit Neurol Neurophysiol Neurobiol, Rome, Italy.

[Vollono, Catello] Catholic Univ, Policlin Univ Agostino Gemelli IRCCS, Unit Neurophysiopathol & Sleep Med, Dipartimento Sci Invecchiamento Neurol Ortoped &, Rome, Italy.

[Anzellotti, Francesca] SS Annunziata Hosp, Neurol Clin, Epilepsy Ctr, Chieti, Italy.
C3 G d'Annunzio University of Chieti-Pescara; San Gerardo Hospital;
University Campus Bio-Medico - Rome Italy; Catholic University of the
Sacred Heart; IRCCS Policlinico Gemelli
RP Anzellotti, F (通讯作者), SS Annunziata Hosp, Neurol Clin, Epilepsy Ctr, Chieti, Italy.
EM f.anzellotti@libero.it
TC 12
Z9 13
PD MAY

PY 2021
VL 118
AR 107887
DI 10.1016/j.yebeh.2021.107887
EA MAR 2021
WC Behavioral Sciences; Clinical Neurology; Psychiatry
ER

PT J
AU Danoun, OA
   Zillgitt, A
   Hill, C
   Zutshi, D
   Harris, D
   Osman, G
   Marawar, R
   Rath, S
   Syed, MJ
   Affan, M
   Schultz, L
   Wasade, VS
AF Danoun, Omar A.
   Zillgitt, Andrew
   Hill, Chloe
   Zutshi, Deepti
   Harris, David
   Osman, Gamaleldin
   Marawar, Rohit
   Rath, Subhendu
   Syed, Maryam J.
   Affan, Muhammad
   Schultz, Lonni
   Wasade, Vibhangini S.
TI Real-world impact of antiepileptic drug combinations with versus without
   perampanel on healthcare resource utilization in patients with epilepsy
   in the United States
SO EPILEPSY & BEHAVIOR
LA English
DT Article
DE Perampanel; Epilepsy; Antiepileptic drug; Combination therapy
ID TRIPHASIC WAVES; CONTINUOUS EEG; CONTINUOUS ELECTROENCEPHALOGRAPHY;
   STATUS EPILEPTICUS; SEIZURES; COVID-19; TERMINOLOGY; PATTERNS
AB Objectives: Combination regimens of antiepileptic drugs (AEDs) with various mechanisms of action (MOA) are commonly used in patients with refractory epilepsy. However, outcomes related to combination AEDs with novel MOA, such as perampanel (PER), are not well described. This study compared healthcare resource utilization (HRU) among recipients of PER-based combinations versus recipients of other non-PER-based

combinations.
Methods: This retrospective study used claims data from the Symphony Health's IDV (R) (Integrated Dataverse) database (August 2012 to July 2018). Patients were aged >= 12 years with epilepsy or non-febrile convulsions, were treated with AED combinations, and had >= 12 and >= 6 months pre- and post-index date, respectively (date of initiation of the second AED in the combination). AEDs were categorized based on MOA: selective non-competitive antagonist of AMPA receptors (i.e., PER), sodium channel blocker (SC), synaptic vesicle protein 2A binding (SV2), and gamma-aminobutyric acid analog (G). Patients were then classified into MOA-based cohorts: PER + SC, PER + SV2, PER + G, SC + SC, SC + SV2, SC + G, SV2 + G, and G + G. HRU outcomes were evaluated during follow-up and compared between PER-based cohorts and non-PER-based cohorts.
Results: On average, patients in the PER + SC (N = 3,592), PER + SV2 (N = 2,200), and PER + G (N = 1,313) cohorts were younger and had a lower Quan-Charlson comorbidity index than those in non-PER-based cohorts. PER + SC and PER + SV2 users had significantly fewer all-cause hospitalizations than non-PER-based users (adjusted RR range: 0.66-0.89, all P < 0.05), while PER + G recipients had fewer all-cause hospitalizations than recipients of SV2 + G and G + G (adjusted RR range: 0.92-0.94). Similar trends were observed for epilepsy-related hospitalizations. Across all comparisons, PER-based combinations were associated with significantly lower rates of all-cause clinic/office/outpatient visits relative to non-PERbased combinations (adjusted RR range: 0.69-0.86, all P < 0.05).
Significance: Results showed that patients treated with PER-based combinations had fewer all-cause and epilepsy-related hospitalizations, and fewer all-cause clinic/office/outpatient visits compared with patients treated with most other non-PER-based combinations. (C) 2021 The Authors. Published by Elsevier Inc.
C1 [Danoun, Omar A.; Schultz, Lonni; Wasade, Vibhangini S.] Henry Ford Hlth Syst, Dept Neurol, 2799 W Grand Blvd, Detroit, MI 48202 USA.
[Zillgitt, Andrew] Beaumont Hlth Adult Comprehens Epilepsy Ctr, Dept Neurol, Royal Oak, MI USA.
[Hill, Chloe; Harris, David; Rath, Subhendu] Univ Michigan, Dept Neurol, Comprehens Epilepsy Program, Ann Arbor, MI USA.
[Zutshi, Deepti; Marawar, Rohit; Syed, Maryam J.] Wayne State Univ, Sch Med, Comprehens Epilepsy Ctr, Detroit Med Ctr, Detroit, MI USA.
[Osman, Gamaleldin] Mayo Clin, Dept Neurol, Rochester, MN USA.
[Affan, Muhammad] Univ Minnesota, Dept Neurol, Minneapolis, MN 55455 USA.
[Schultz, Lonni] Henry Ford Hlth Syst, Dept Publ Hlth Sci, Detroit, MI 48202 USA.
[Zutshi, Deepti; Marawar, Rohit; Syed, Maryam J.; Wasade, Vibhangini S.] Wayne State Univ, Sch Med, Dept Neurol, Detroit, MI 48201 USA.
C3 Henry Ford Hospital; University of Michigan System; University of
Michigan; Detroit Medical Center; Wayne State University; Mayo Clinic;
University of Minnesota System; University of Minnesota Twin Cities;
Henry Ford Hospital; Wayne State University
RP Danoun, OA (通讯作者), Henry Ford Hlth Syst, Dept Neurol, 2799 W Grand Blvd, Detroit, MI 48202 USA.
EM Odanoun1@hfhs.org
TC 5
Z9 6

PD MAY
PY 2021
VL 118
AR 107927
DI 10.1016/j.yebeh.2021.107923
EA MAR 2021
WC Behavioral Sciences; Clinical Neurology; Psychiatry
ER

PT J
AU Gravel, V
Boucher, O
Citherlet, D
Hebert-Seropian, B
Bouthillier, A
Nguyen, DK
AF Gravel, Victoria
Boucher, Olivier
Citherlet, Daphne
Hebert-Seropian, Benjamin
Bouthillier, Alain
Dang Khoa Nguyen
TI The impact of COVID-19 pandemic on frail health systems of low- and middle-income countries: The case of epilepsy in the rural areas of the Bolivian Chaco
SO EPILEPSY & BEHAVIOR
LA English
DT Article
DE Epilepsy; Bolivia; COVID-19; Health system
ID QUALITY-OF-LIFE; TEMPORAL-LOBE EPILEPSY; PSYCHIATRIC COMORBIDITY; HIPPOCAMPAL VOLUME; RESECTIVE SURGERY; RISK-FACTORS; DEPRESSION; ANXIETY; LOBECTOMY; SEIZURE
AB Introduction: The Coronavirus disease 2019 (COVID-19) has put some health systems under pressure, especially in low- and middle-income countries. We aimed at evaluating the impact of COVID-19 emergency on the management of people with epilepsy (PWE) living in the rural communities of the Gran Chaco area of the Plurinational State of Bolivia.

Materials and methods: We selected a sample of PWE living in the rural communities of the Bolivian Chaco. A standardized questionnaire was developed, consisting of six questions addressing drug availability, drug discontinuation, personnel responsible for drug retrieval during the lockdown, and the presence of seizures in the two months preceding the interview. Questionnaires were administered by community health workers of the rural health centers in September 2020.

Results: Seventy PWE (38 men, 54.3%; mean age 26.9 +/- 16.7) were interviewed. During the lockdown the large majority of them (n = 51, 73.9%) reported an irregular medication intake mainly due to the lack of antiseizure medications in the local health posts, leading to an increase in seizure frequency.

Conclusion: The COVID-19 pandemic has unmasked the frailty of the Bolivian health

system, especially for the management of chronic diseases such as epilepsy in the rural communities. (C) 2021 Elsevier Inc. All rights reserved.
C1 [Gravel, Victoria; Boucher, Olivier] Univ Montreal, Dept Psychol, Montreal, PQ, Canada.
[Gravel, Victoria; Boucher, Olivier; Citherlet, Daphne; Hebert-Seropian, Benjamin; Bouthillier, Alain; Dang Khoa Nguyen] Ctr Hosp Univ Montreal, Ctr Rech, Montreal, PQ, Canada.
[Boucher, Olivier] Ctr Hosp Univ Montreal, Serv Psychol, Montreal, PQ, Canada.
[Citherlet, Daphne; Dang Khoa Nguyen] Univ Montreal, Dept Neurosci, Montreal, PQ, Canada.
[Hebert-Seropian, Benjamin] Univ Quebec Montreal, Dept Psychol, Montreal, PQ, Canada.
[Bouthillier, Alain] Ctr Hosp Univ Montreal, Serv Neurochirurg, Montreal, PQ, Canada.
[Dang Khoa Nguyen] Ctr Hosp Univ Montreal, Serv Neurol, Montreal, PQ, Canada.
C3 Universite de Montreal; Universite de Montreal; Universite de Montreal;
Universite de Montreal; University of Quebec; University of Quebec
Montreal; Universite de Montreal; Universite de Montreal
RP Nguyen, DK (通讯作者), Ctr Hosp Univ Montreal CHUM, Serv Neurol, 900 Rue St Denis, Montreal, PQ H2X 0A9, Canada.
EM d.nguyen@umontreal.ca
TC 0
Z9 0
PD MAY
PY 2021
VL 118
AR 107917
DI 10.1016/j.yebeh.2021.107919
EA MAR 2021
WC Behavioral Sciences; Clinical Neurology; Psychiatry
ER

PT J
AU Rosellini, I
Vianello, M
Ghazaryan, A
Silvia, VG
Palmieri, A
Giopato, F
Vitaliani, R
Fuccaro, M
Terrin, A
Rigoni, MT
Pietrobon, F
Bonifati, DM
AF Rosellini, Irene
Vianello, Marika

Ghazaryan, Anna
Silvia, Vittoria Guidoni
Palmieri, Anna
Giopato, Federico
Vitaliani, Roberta
Fuccaro, Matteo
Terrin, Alberto
Rigoni, Maria Teresa
Pietrobon, Francesco
Bonifati, Domenico Marco
TI Virtual visits for chronic neurologic disorders during COVID-19 pandemic
SO NEUROLOGICAL SCIENCES
LA English
DT Article
DE Telemedicine; Neurology; COVID-19; care; Chronic neurologic disorders
AB Background COVID-19 pandemic has boosted telemedicine in medical clinical practice. Experiences in the management of chronic neurological disorders are limited and scattered. The aim of the study was to evaluate feasibility and efficacy of virtual visit for chronic neurological disorders during COVID-19 pandemic. Methods All patients scheduled for a visit during the lockdown period were contacted. The patients fell into four categories: (1) long-term follow-up, the patient was re-scheduled; (2) visit was necessary, teleconsultation was accepted; (3) problem was solved by phone call; and (4) visit was necessary and teleconsultation was not feasible, then visit was maintained. Google Meet was used. During the virtual visit, neurological examination was performed, and demographic and clinical characteristics were recorded. Results At the end of May 2020, 184 virtual visits for 178 patients were performed for the following diseases: myasthenia gravis (47 patients), multiple sclerosis (79), epilepsy (12), headache (6), and parkinsonism (34). The patients were 70 males and 108 females with a mean age of 53.5 years (range 13-90). During virtual visit, we were able to obtain a satisfactory neurological examination. Conclusions We demonstrated feasibility and effectiveness of virtual visit in the management of a large group of patients with common chronic neurological disorders.
C1 [Rosellini, Irene; Vianello, Marika; Ghazaryan, Anna; Silvia, Vittoria Guidoni; Palmieri, Anna; Giopato, Federico; Vitaliani, Roberta; Fuccaro, Matteo; Terrin, Alberto; Rigoni, Maria Teresa; Bonifati, Domenico Marco] Ca Foncello Hosp, Dept Neurocardiovasc, Neurol Unit, I-31100 Treviso, Italy.
[Pietrobon, Francesco] AULSS 2 Treviso, Sociohlth Dist South, Treviso, Italy.
C3 ULSS 2 Marca TV; Ospedale Ca’ Foncello Treviso
RP Bonifati, DM (通讯作者), Ca Foncello Hosp, Dept Neurocardiovasc, Neurol Unit, I-31100 Treviso, Italy.
EM domenicomarco.bonifati@aulss2.veneto.it
TC 6
Z9 6
PD JUL
PY 2021
VL 42
IS 7

BP 2607
EP 2610
DI 10.1007/s10072-021-05212-3
EA MAR 2021
WC Clinical Neurology; Neurosciences
ER

PT J
AU Olivo, S
Cheli, M
Dinoto, A
Stokelj, D
Tomaselli, M
Manganotti, P
AF Olivo, S.
Cheli, M.
Dinoto, A.
Stokelj, D.
Tomaselli, M.
Manganotti, P.
TI Telemedicine during the SARS-Cov-2 pandemic lockdown: Monitoring stress and quality of sleep in patients with epilepsy
SO EPILEPSY & BEHAVIOR
LA English
DT Article
DE Epilepsy; COVID-19; Sleep; Stress; Telemedicine
ID PARTIAL-ONSET SEIZURES; NEWLY-DIAGNOSED EPILEPSY; RANDOMIZED PHASE-III; ADJUNCTIVE PERAMPANEL; TREATMENT PATTERNS; MANAGEMENT; DRUGS
AB SARS-CoV-2 pandemic heavily hit the western healthcare system saturating the hospital beds in wards and clogging the emergency departments. To avoid the collapse of Italian hospitals, office visits to outpatients were limited to emergencies and the general population went in a lockdown state. Physicians had to approach new problems in the management of chronic patients who could not leave their homes. In our experience as epilepsy clinic, the use of telemedicine was of crucial importance for monitoring our patients: phone call during lockdown let us monitor the stability of our 38 patients and psychometric parameters and habits that could influence seizures frequency. In particular, we found that in our patients, sleep quality was low resulting in high daily sleepiness and associated high stress levels. Secondly, we found an increase in daily screen hours and an association with daily sleepiness. In conclusion, we report our experience in managing people with epilepsy during the lockdown, underlining the utility of telemedicine as a valid monitoring tool and the necessity of a psychometric and behavioral screening. (C) 2021 Published by Elsevier Inc.
C1 [Olivo, S.; Cheli, M.; Dinoto, A.; Stokelj, D.; Tomaselli, M.; Manganotti, P.] Univ Trieste, Cattinara Univ Hosp ASUGI, Dept Med Surg & Hlth Sci, Clin Unit Neurol, Str Fiume 447, I-34149 Trieste, Italy.
C3 University of Trieste
RP Manganotti, P (通讯作者), Univ Trieste, Cattinara Univ Hosp ASUGI, Dept Med Surg

& Hlth Sci, Clin Unit Neurol, Str Fiume 447, I-34149 Trieste, Italy.
EM pmanganotti@units.it
TC 2
Z9 2
PD MAY
PY 2021
VL 118
AR 107864
DI 10.1016/j.yebeh.2021.107927
EA MAR 2021
WC Behavioral Sciences; Clinical Neurology; Psychiatry
ER

PT J
AU Correale, C
   Tondo, I
   Falamesca, C
   Capitello, TG
   Vigevano, F
   Specchio, N
   Cappelletti, S
AF Correale, C.
   Tondo, I.
   Falamesca, C.
   Capitello, T. Grimaldi
   Vigevano, F.
   Specchio, N.
   Cappelletti, S.
TI Depression and anxiety in hospitalized children with epilepsy during
   COVID-19 pandemic: Preliminary findings of a cross-sectional study
SO EUROPEAN PSYCHIATRY
LA English
DT Meeting Abstract
DE COVID-19; Epilepsy; Anxiety; Depression
C1 [Correale, C.; Tondo, I.; Falamesca, C.; Capitello, T. Grimaldi] Bambino Gesu Children Hosp, Clin Psychol, Rome, Italy.
   [Vigevano, F.; Specchio, N.; Cappelletti, S.] Bambino Gesu Children Hosp, Neurol Sci, Rome, Italy.
C3 IRCCS Bambino Gesu; IRCCS Bambino Gesu
TC 0
Z9 0
PD APR
PY 2021
VL 64
SU 1
SI SI
MA EPV0195

BP S670
EP S671
DI 10.1192/j.eurpsy.2021.1780
WC Psychiatry
ER

PT J
AU Godoy-Santin, J
Grau, SB
Nunez, F
Aguilar, C
Gutierrez, D
Miranda, H
Rubio, PS
Ramos, B
Garcia, AL
Andresen, M
Mellado, P
AF Godoy-Santin, Jaime
Bravo Grau, Sebastian
Nunez, Felipe
Aguilar, Cecilia
Gutierrez, Diego
Miranda, Hector
Sandoval Rubio, Patricio
Ramos, Bernardita
Garcia, Lorena A.
Andresen, Max
Mellado, Patricio
TI Neurology and COVID-19: Case Series of Neurological Complications in 96 patients Admitted at a University Hospital
SO REVISTA MEDICA DE CHILE
LA Spanish
DT Article
DE COVID-19; Delirium; Neurology; Seizure; Stroke
AB Background: There are multisystemic consequences secondary to SARS-CoV-2 infection. Aim: To characterize neurological complications in patients admitted due to SARS-CoV-2 infection. Methods: Review of medical records of patients aged over 15 years with COVID-19 evaluated by the neurology team between April and August 2020 at a university hospital. Severity of the infection, referral reasons, neurological diagnoses and laboratory results were registered. The diagnoses were defined by consensus among the members of the hospital neurology group. Cerebrovascular and inflammatory diseases of the central and peripheral nervous system were defined as "probably associated" or "possibly associated" to COVID-19. Results: Ninety-six patients had at least 1 new neurological complication. 74% were admitted due to pneumonia and 20% due to a neurological disease. The most common reasons for neurological referral were impaired consciousness (39%), focal neurological deficit (24%), headache (9%) and seizures (5%).

The most relevant neurological diagnoses were delirium in 48 patients, stroke in 24, critical illness polyneuropathy and myopathy in 17, seizures in 14, brachial plexopathy in 3, compressive neuropathies in 5, encephalitis in 1, possible vasculitis in 1 and Guillain-Barre syndrome in 1. Stroke and epilepsy were associated with increased length of hospital stay, but without differences in mortality. Conclusions: The spectrum of neurological complications of COVID-19 is wide. There are clinical entities typical of critically ill patients and also diseases associated directly and indirectly with the SARS-CoV2 infection.
C1 [Godoy-Santin, Jaime; Aguilar, Cecilia; Gutierrez, Diego; Miranda, Hector; Sandoval Rubio, Patricio; Mellado, Patricio] Pontificia Univ Catolica Chile, Dept Neurol, Escuela Med, Santiago, Chile.
[Bravo Grau, Sebastian] Pontificia Univ Catolica Chile, Dept Radiol, Escuela Med, Santiago, Chile.
[Andresen, Max] Pontificia Univ Catolica Chile, Dept Med Intens, Escuela Med, Santiago, Chile.
[Godoy-Santin, Jaime] Pontificia Univ Catolica Chile, Grp Trabajo AIRR UC, Fac Med, Santiago, Chile.
[Nunez, Felipe; Ramos, Bernardita; Garcia, Lorena A.] Pontificia Univ Catolica Chile, Escuela Med, Santiago, Chile.
C3 Pontificia Universidad Catolica de Chile; Pontificia Universidad
Catolica de Chile; Pontificia Universidad Catolica de Chile; Pontificia
Universidad Catolica de Chile; Pontificia Universidad Catolica de Chile
RP Mellado, P (通讯作者), Dept Neurol, Diagonal Paraguay 362,5 Piso, Santiago, Chile.
EM pmelladotalesnik@gmail.com
TC 1
Z9 1
PD APR
PY 2021
VL 149
IS 4
BP 527
EP 532
WC Medicine, General & Internal
ER

PT J
AU Tedrus, GMDS
da Silva, JFCP
Barros, GS
AF de Almeida Souza Tedrus, Gloria Maria
Cloclet Pio da Silva, Joao Fernando
Barros, Gabriel Santaterra
TI The impact of COVID-19 on patients with epilepsy
SO ARQUIVOS DE NEURO-PSIQUIATRIA
LA English
DT Article
DE Coronavirus Infections; Pandemics; Epilepsy; Seizure

AB Background: The COVID-19 pandemic and social distancing can have adverse impacts on adult people with epilepsy (PWE). Objective: To investigate the seizure frequency, the perceived well-being, and the presence of anxiety symptoms in PWE during the COVID-19 pandemic period. Methods: Data from a questionnaire on the repercussions of COVID-19 were analyzed in relation to the clinical variables of 114 PWE, with a significance level of p<0.05. Results: There were 26 cases of COVID-19 in PWE and/or family members (22.8%). During the pandemic period, 11 PWE (9.6%) reported an increase in seizures, but unrelated to COVID-19. Also, the number of crises in PWE with previous depressive disorders increased, with differences between epilepsies. Symptoms of depression, impaired well-being, and concern for their lifestyle were significant in PWE with a previous diagnosis of depression. Impaired well-being, increased anxiety, nervousness, and tiredness, and the concern with being infected were mentioned by a high number of PWE in the pandemic. Conclusion: Seizure frequency increased during the pandemic period, a finding associated with clinical variables of epilepsy. PWE with depression had worse perceived well-being. Changes in well-being and increased anxiety and nervousness were frequent in the pandemic.
C1 [de Almeida Souza Tedrus, Gloria Maria] Pontificia Univ Catolica Campinas, Programa Posgrad Cienciad Saude, Campinas, SP, Brazil.
[Cloclet Pio da Silva, Joao Fernando; Barros, Gabriel Santaterra] Pontificia Univ Catolica Campinas, Fac Med, Campinas, SP, Brazil.
C3 Pontificia Universidade Catolica de Campinas; Pontificia Universidade Catolica de Campinas
RP da Silva, JFCP (通讯作者), Pontificia Univ Catolica Campinas, Fac Med, Campinas, SP, Brazil.
EM joao.fcps@puccampinas.edu.br
TC 1
Z9 1
PD APR
PY 2021
VL 79
IS 4
BP 310
EP 314
DI 10.1590/0004-282X-ANP-2020-0517
WC Neurosciences; Psychiatry
ER

PT J
AU Casassa, C
Moss, R
Goldenholz, DM
AF Casassa, Charles
Moss, Robert
Goldenholz, Daniel M.
TI Epilepsy during the COVID-19 pandemic lockdown: a US population survey
SO EPILEPTIC DISORDERS
LA English

DT Article
DE SARS-CoV-2; pandemic; epilepsy; seizure; antiepileptic drug; epidemiology
ID GUIDELINE DEVELOPMENT; AMERICAN ACADEMY; UNITED-STATES; RISK-FACTORS; SUBCOMMITTEE; PEOPLE
AB Objective. This study sought to understand issues facing people with epilepsy (PWE) during the lockdown period of the COVID-19 pandemic in the United States.

Methods. We conducted a cross-sectional study using a 20-question survey that used Seziure Tracker.com, sent to eligible PWE and their caregivers on May 6th, 2020. Questions about demographics and medical history were used to calculate COVID mortality risk odds ratios (OR) compared to a low baseline risk group.

Results. In total, 505 responses were collected. Of these, 71% reported no change in seizure rates and 25% reported an increase in seizures, which they attributed primarily to disrupted sleep (63%) and decreased exercise (42%). Mortality risks from COVID-19 had median OR of 1.67, ranging 1.00-906.98. Fear about hospitalization (53%) and concern for loved ones (52%) were prominent concerns. Of the respondents, 5% reported stopping or reducing anti-seizure medications due to problems communicating with doctors, access or cost. Lower-risk COVID patients reported more fear of hospitalization (55% versus 38%, $p<0.001$) and anxiety about medication access (43% versus 28%, $p=0.03$) compared with higher-risk COVID patients. Increased anxiety was reported in 47%, and increased depression in 28%. Ten percent without generalized convulsions and 8% with did not know anything about epilepsy devices (VNS, RNS, DBS).

Significance. The COVID-19 pandemic presents unique challenges to PWE, including increased seizure rates, problems with access and cost of life-saving medications. Those with lower COVID-19 risk may have been marginalized more than those with higher risk. Efforts to protect PWE during major public health emergencies should take these findings into account.
C1 [Casassa, Charles] Loma Linda Univ, Div Epilepsy, Dept Neurol, Med Ctr, 11370 Anderson St,Suite B100, Loma Linda, CA 92354 USA.

[Moss, Robert] SeizureTracker LLC, Springfield, VA USA.

[Goldenholz, Daniel M.] Harvard Med Sch, Div Epilepsy, Beth Israel Deaconess Med Ctr, Dept Neurol, Boston, MA 02115 USA.
C3 Loma Linda University; Harvard University; Beth Israel Deaconess Medical Center; Harvard Medical School
RP Casassa, C (通讯作者), Loma Linda Univ, Div Epilepsy, Dept Neurol, Med Ctr, 11370 Anderson St,Suite B100, Loma Linda, CA 92354 USA.
EM cmc2454@gmail.com
TC 2
Z9 2
PD APR
PY 2021
VL 23
IS 2
BP 257
EP 267
DI 10.1684/epd.2021.1259
WC Clinical Neurology
ER

PT J
AU Sun, LQ
Mo, QN
Sun, HB
Niu, YL
Si, Y
AF Sun, Lingqi
Mo, Qianning
Sun, Hongbin
Niu, Yulong
Si, Yang
TI Depression in patients with epilepsy during the COVID-19 pandemic based on longitudinal self-reporting
SO EPILEPTIC DISORDERS
LA English
DT Article
DE COVID-19; depression; epilepsy
ID RISK-FACTORS; VALIDATION
AB Objective. The current study screened major depression in people with epilepsy (PWE) during the epidemic of the novel coronavirus-related disease COVID-19, in order to identify whether the outbreak generated negative psychological impact on PWE.

Methods. A Chinese version of the Neurological Disorders Depression Inventory for Epilepsy (C-NDDI-E), a self-reporting depression inventory, was applied for rapid detection of major depression. Assessment was carried out online during three different periods (prior to, during, and after the outbreak of COVID-19), with the aim of identifying changes in prevalence of depression and associated risk factors.

Results. A total of 158 PWE were recruited into the study (48.7% female). The questionnaire completion rates were 94.3% and 70.9% during and after the outbreak, respectively. The prevalence of depression prior to the epidemic, as the baseline, was 34.8% and increased to 42.3% during the period of the epidemic. Towards the end of the outbreak, the prevalence declined towards the baseline (36.6%). Factors such as living alone (OR = 4.022, 95% CI: 1.158-13.971, P = 0.028) and active seizures before the epidemic (OR = 2.993, 95% CI: 1.197-7.486, P = 0.019) were associated with depression during the epidemic. Monotherapy appeared to be protective against depression (OR = 0.105, 95% CI: 0.047-0.235, P <0.001).

Significance. Our results suggest that the pandemic exerts negative influence on PWE's mental health. Depression is one of the common psychological disorders that needs greater attention during this extraordinary period.
C1 [Sun, Lingqi] Southwest Med Univ, Luzhou 646000, Sichuan, Peoples R China.
[Mo, Qianning; Sun, Hongbin; Si, Yang] Sichuan Acad Med Sci, Chengdu 610072, Peoples R China.
[Mo, Qianning] Sichuan Prov Peoples Hosp, Med Adm Dept, Chengdu 610072, Peoples R China.
[Sun, Hongbin; Si, Yang] Sichuan Prov Peoples Hosp, Dept Neurol, Chengdu 610072, Sichuan, Peoples R China.
[Niu, Yulong] Sichuan Univ, Coll Life Sci, Key Lab Bioresource & Ecoenvironm, Minist

Educ, Chengdu 610065, Sichuan, Peoples R China.
[Si, Yang] Univ Elect Sci & Technol China, Chengdu, Sichuan, Peoples R China.
C3 Southwest Medical University; Sichuan Provincial People's Hospital; Sichuan Provincial People's Hospital; Sichuan Provincial People's Hospital; Sichuan University; University of Electronic Science & Technology of China
RP Niu, YL (通讯作者), Sichuan Univ, 24 S Sec 1,1st Ring Rd, Chengdu 610065, Peoples R China.; Si, Y (通讯作者), 32 W Sec 2,1st Ring Rd, Chengdu 610072, Peoples R China.
EM yulong.niu@hotmail.com; yangsi_neuroscience@hotmail.com
TC 2
Z9 2
PD APR
PY 2021
VL 23
IS 2
BP 268
EP 273
DI 10.1684/epd.2021.1263
WC Clinical Neurology
ER

PT J
AU Nussbaum, NL
Young, SR
DeLeon, RC
Engelmann, ML
Schraegle, WA
AF Nussbaum, Nancy L.
Young, Stephanie R.
DeLeon, Rosario C.
Engelmann, Morgan L.
Schraegle, William A.
TI The future is now: pediatric neuropsychological presurgical epilepsy evaluation in the age of COVID-19
SO EPILEPTIC DISORDERS
LA English
DT Article
DE pre-surgical planning; neuropsychology; epilepsy; virtual assessment; COVID-19; telemedicine
ID DIAGNOSTIC METHODS COMMISSION; TASK-FORCE; EXPECTATIONS; SURGERY
AB Objective. The objective of this brief report is to review an assessment paradigm for conducting virtual neuropsychological pre-surgical evaluations in the context of the COVID-19 pandemic.
Methods. A multidisciplinary epilepsy team at a Level 4 epilepsy center within a large children's academic medical center convened to discuss the challenges and possible solutions for Phase II evaluations for pediatric patients with pharmacoresistant epilepsy during the COVID-19 pandemic. The neuropsychologists

explored evidence-based methods of virtual evaluation and developed a systematic decision-making process for youth requiring a Phase II evaluation.
Results. We propose models of assessment which prioritize teleneuropsychology when possible to reduce the risk of infection: (1) evaluation with directly administered tests through a completely virtual format; (2) virtual/in-person hybrid evaluation; and (3) clinical observation/interview in a virtual format supplemented by survey data. These models are illustrated by three cases.
Significance. Using virtual assessment models, the team was able to meet the urgent patient care needs and coiled useful data while minimizing the risk of virus spread. The paradigms presented may be useful examples for other multidisciplinary surgical teams interested in incorporating teleneuropsychology into their practices.
C1 [Nussbaum, Nancy L.; DeLeon, Rosario C.; Schraegle, William A.] Univ Texas Hlth Austin Pediat Neurosci Dell Child, Dept Neurol, 1600 W 38th St,Suite 320, Austin, TX 78759 USA.
[Young, Stephanie R.] Univ Colorado, Dept Pediat, Neurol Sect, Sch Med, Denver, CO 80202 USA.
[Engelmann, Morgan L.] Univ Texas Austin, Dept Educ Psychol, Austin, TX 78712 USA.
C3 University of Colorado System; University of Colorado Anschutz Medical Campus; University of Colorado Denver; University of Texas System; University of Texas Austin
RP Nussbaum, NL (通讯作者), Univ Texas Hlth Austin Pediat Neurosci Dell Child, Dept Neurol, 1600 W 38th St,Suite 320, Austin, TX 78759 USA.
EM nnussbaum@ascension.org
TC 1
Z9 1
PD APR
PY 2021
VL 23
IS 2
BP 274
EP 280
DI 10.1684/epd.2021.1274
WC Clinical Neurology
ER

PT J
AU Kariyappa, M
Govindarajan, V
Kommalur, A
AF Kariyappa, Mallesh
Govindarajan, Varun
Kommalur, Anitha
TI Acute Leukoencephalopathy with Restricted Diffusion in an Infant with Severe COVID-19 and Dengue Coinfection Progressing to West Syndrome
SO JOURNAL OF TROPICAL PEDIATRICS
LA English
DT Article

DE COVID19; SARS-CoV2; ALERD; infantile dengue; West Syndrome; ADEM
AB COVID-19 pandemic is increasingly being recognized in infants and some develop cytokine storm mediated tissue damage. We report 5-month-old infant presenting with fever, refusal of feeds, developing altered sensorium and convulsions during the hospital course, tested positive for SARS-CoV2 RT-PCR in second week of illness. Her serology was also Dengue positive. She had features of cytokine storm and her MRI Brain suggested acute demyelinating encephalomyelitis (ADEM). She was treated with high-dose methylprednisolone followed oral prednisolone, under antibiotics cover. Infant improved gradually over 3 weeks duration following a stormy hospital course. On follow-up, infant showed delayed motor milestones with epileptic spasms and hysparrhthymia on EEG, progressing to develop secondary West syndrome. Features of acute encephalopathy, hypercytokinemia and restricted diffusion on DWI-MRI, with post-encephalopathic epilepsy, pointed to a differential of ADEM-acute leukoencephalopathy with restricted diffusion (ALERD) as the primary diagnosis; establishing ALERD as a possible neurological complication of COVID-19 infection in infants.
C1 [Kariyappa, Mallesh; Govindarajan, Varun; Kommalur, Anitha] Bangalore Med Coll & Res Inst, Dept Paediat, Bengaluru 560002, Karnataka, India.
C3 Bangalore Medical College & Research Institute (BMCRI)
RP Govindarajan, V (通讯作者), Bangalore Med Coll & Res Inst, Vanivilas Hosp, Dept Paediat, KR Rd, Bengaluru 560002, Karnataka, India.
EM varunuma@gmail.com
TC 0
Z9 0
PD APR
PY 2021
VL 67
IS 2
AR fmab026
DI 10.1093/tropej/fmab026
WC Pediatrics; Tropical Medicine
ER

PT J
AU Puteikis, K
   Mameniskiene, R
AF Puteikis, Kristijonas
   Mameniskiene, Ruta
TI Factors Associated with COVID-19 Vaccine Hesitancy among People with
   Epilepsy in Lithuania
SO INTERNATIONAL JOURNAL OF ENVIRONMENTAL RESEARCH AND PUBLIC HEALTH
LA English
DT Article
DE COVID-19; epilepsy; influenza; vaccine hesitancy
ID SEIZURES; RISK; IMMUNIZATION
AB The purpose of our study was to determine the willingness to be vaccinated against COVID-19 and factors associated with vaccine hesitancy among people with epilepsy (PWE).

In December 2020, we performed an online cross-sectional survey of PWE and their caregivers in Lithuania before the rollout of COVID-19 vaccines to the public. The study sample consisted of 111 respondents (44 (39.6%) male, median age 25 years (range 1 to 70)). From 58 PWE who personally responded to the survey, 27 (46.6%) would be willing to be vaccinated against COVID-19. Among the 53 caregivers, 18 (34.0%) would accept the person they care for to be vaccinated. Willingness to be vaccinated was associated with receiving an influenza shot in 2020 (odds ratio (OR) = 9.17, 95% confidence interval (CI = 1.15-73.47), the beliefs that vaccines are generally safe (OR = 7.90, 95% CI = 2.43-25.74) and that they are the only convenient way to gain immunity (OR = 3.91, 95% CI = 1.02-15.05). Respondents were hesitant to accept the COVID-19 vaccine if they thought it could cause the infection (OR = 0.14, 95% CI = 0.04-0.49). COVID-19 vaccine hesitancy is frequent among PWE and their caregivers. It is probably related to erroneous beliefs about their safety and mechanism of action.
C1 [Puteikis, Kristijonas] Vilnius Univ, Fac Med, LT-03101 Vilnius, Lithuania.
[Mameniskiene, Ruta] Vilnius Univ, Ctr Neurol, LT-08661 Vilnius, Lithuania.
C3 Vilnius University; Vilnius University
RP Mameniskiene, R (通讯作者), Vilnius Univ, Ctr Neurol, LT-08661 Vilnius, Lithuania.
EM Kristijonas.puteikis@mf.stud.vu.lt; ruta.mameniskiene@santa.lt
TC 7
Z9 7
PD APR
PY 2021
VL 18
IS 8
AR 4374
DI 10.3390/ijerph18084374
WC Environmental Sciences; Public, Environmental & Occupational Health
ER

PT J
AU Costa, AM
Marchio, M
Bruni, G
Bernabei, SM
Cavalieri, S
Bondi, M
Biagini, G
AF Costa, Anna-Maria
Marchio, Maddalena
Bruni, Giulia
Bernabei, Silvia Maria
Cavalieri, Silvia
Bondi, Marina
Biagini, Giuseppe
TI Evaluation of E-Health Applications for Paediatric Patients with Refractory Epilepsy and Maintained on Ketogenic Diet
SO NUTRIENTS

LA English
DT Article
DE COVID-19 pandemic; dietary management; drug resistant epilepsy; E-health applications; Italy; ketogenic diet; smart technology; smartphone app
ID MANAGEMENT; QUESTIONNAIRE; TECHNOLOGIES; SATISFACTION; CHILDREN
AB E-health technologies improve healthcare quality and disease management. The aim of this study was to develop a ketogenic diet management app as well as a website about this dietary treatment and to evaluate the benefits of giving caregivers access to various web materials designed for paediatric patients with refractory epilepsy. Forty families participated in the questionnaire survey, from January 2016 to March 2016. All caregivers were exposed to paper-based materials about the ketogenic diet, whereas only 22 received the app, called KetApp, and videos produced by dieticians. Caregivers with free access to web materials were more satisfied than the others with the informative material provided by the centre (p <= 0.001, Mann-Whitney test). Indeed, they showed a better attitude towards treatment, and they became more aware of dietary management in comparison to the control group (p <= 0.001). Moreover, caregivers provided with web materials were stimulated to pursue the treatment (p = 0.002) and to introduce it to their children and other people (p = 0.001). Additionally, caregivers supplied with web materials were more willing to help other families in choosing the ketogenic diet (p = 0.004). Overall, these findings indicate that web materials are beneficial for caregivers of paediatric patients with refractory epilepsy in our centres. Thus, the use of e-health applications could be a promising tool in the daily aspects of ketogenic diet management, and it is especially of value in the attempt to start or maintain the diet during the ongoing COVID-19 pandemic crisis.
C1 [Costa, Anna-Maria; Marchio, Maddalena; Biagini, Giuseppe] Univ Modena & Reggio Emilia, Dept Biomed Metab & Neural Sci, Lab Expt Epileptol, I-41125 Modena, Italy.
[Bruni, Giulia] AOU Meyer Hosp Florence, I-50139 Florence, Italy.
[Bernabei, Silvia Maria] Bambino Gesu Pediat Hosp, Dept Paediat Specialties & Liver Kidney Transplan, UO Nutr Rehabil, I-00165 Rome, Italy.
[Cavalieri, Silvia] Univ Verona, Dept Foreign Languages & Literatures, I-37129 Verona, Italy.
[Bondi, Marina] Univ Modena & Reggio Emilia, Dept Studies Language & Culture, I-41121 Modena, Italy.
[Biagini, Giuseppe] Univ Modena & Reggio Emilia, Ctr Neurosci & Neurotechnol, I-41125 Modena, Italy.
C3 Universita di Modena e Reggio Emilia; IRCCS Bambino Gesu; University of Verona; Universita di Modena e Reggio Emilia; Universita di Modena e Reggio Emilia
RP Marchio, M (通讯作者), Univ Modena & Reggio Emilia, Dept Biomed Metab & Neural Sci, Lab Expt Epileptol, I-41125 Modena, Italy.
EM annamaria.costa@unimore.it; dietista.maddalenamarchio@gmail.com; giulia.bruni@meyer.it; silviamaria.bernabei@opbg.net; silvia.cavalieri@univr.it; marina.bondi@unimore.it; gbiagini@unimore.it
TC 10
Z9 10
PD APR
PY 2021

VL 13
IS 4
AR 1240
DI 10.3390/nu13041240
WC Nutrition & Dietetics
ER

PT J
AU Magdy, R
Kishk, NA
Fouad, AM
Alsayyad, E
AF Magdy, Rehab
Kishk, Nirmeen A.
Fouad, Amr M.
Alsayyad, Enas
TI Risk estimation of SUDEP during COVID-19 pandemic era in a tertiary referral center
SO EPILEPSY RESEARCH
LA English
DT Article
DE SUDEP; COVID-19; Active epilepsy; Drug resistant epilepsy
ID SUDDEN UNEXPECTED DEATH; EPILEPSY
AB Objective: No data exist regarding the impact of the lockdown due to the COVID-19 pandemic on the risk factors of sudden unexpected death in epilepsy (SUDEP). This study aimed to stratify risk factors of SUDEP in relation to COVID-19 lockdown, among patients with epilepsy (PWE) in Cairo University epilepsy unit (CUEU). Therefore, we can detect risk factors and mitigate such factors in the second wave of the virus. Methods: an observational, cross-sectional study carried on 340 Egyptian patients with active epilepsy. Individual risk identification and stratification was done by using The SUDEP and seizure Safety Checklist, after which sharing risk knowledge to PWE and their caregivers was undertaken. Results: The mean age of patients was 29.72 +/- 12.12. The median of the static factors was 4 (IQR 3-5) whereas, the median of the modifiable factors was 2 (IQR 1-3). Epilepsy emergencies (serial seizures or status epilepticus) were reported in 24.1 % of patients, for which non-compliance was the commonest cause, followed by deferral of epilepsy surgery for patients with drug resistant epilepsy (DRE). Stepwise logistic regression analysis showed that use of anxiolytic medications, non-compliance, keeping patients with DRE on dual anti-seizure medications (ASMs), or adding third medication increased the odds of increased seizure frequency by 2.7, 3.5, 16.6 and 6.1 times, respectively. Conclusion: Some COVID-19 related issues had influenced the risk of seizure worsening including postponing epilepsy surgery for patients with DRE, non-compliance, and psychiatric comorbidities. Special attention should be paid to these issues to mitigate the risk of SUDEP.
C1 [Magdy, Rehab; Kishk, Nirmeen A.; Fouad, Amr M.; Alsayyad, Enas] Cairo Univ, Kasr Al Ainy Fac Med, Dept Neurol, Cairo, Egypt.
C3 Egyptian Knowledge Bank (EKB); Cairo University
RP Magdy, R (通讯作者)，Cairo Univ, Kasr Al Ainy Fac Med, Dept Neurol, Cairo, Egypt.

EM rehab.m.hassan@kasralainy.edu.eg
TC 0
Z9 0
PD JUL
PY 2021
VL 173
AR 106625
DI 10.1016/j.eplepsyres.2021.106625
EA APR 2021
WC Clinical Neurology
ER

PT J
AU Flores-Silva, FD
Garcia-Grimshaw, M
Valdes-Ferrer, SI
Vigueras-Hernandez, AP
Dominguez-Moreno, R
Tristan-Samaniego, DP
Michel-Chavez, A
Gonzalez-Duarte, A
Vega-Boada, FA
Reyes-Melo, I
Jimenez-Ruiz, A
Chavez-Martinez, OA
Rebolledo-Garcia, D
Marche-Fernandez, OA
Sanchez-Torres, S
Garcia-Ramos, G
Cantu-Brito, C
Chiquete, E
AF Flores-Silva, Fernando Daniel
Garcia-Grimshaw, Miguel
Valdes-Ferrer, Sergio Ivan
Vigueras-Hernandez, Alma Poema
Dominguez-Moreno, Rogelio
Tristan-Samaniego, Dioselina Panama
Michel-Chavez, Anaclara
Gonzalez-Duarte, Alejandra
Vega-Boada, Felipe A.
Reyes-Melo, Isael
Jimenez-Ruiz, Amado
Chavez-Martinez, Oswaldo Alan
Rebolledo-Garcia, Daniel
Marche-Fernandez, Osvaldo Alexis
Sanchez-Torres, Samantha
Garcia-Ramos, Guillermo

Cantu-Brito, Carlos
Chiquete, Erwin
TI Neurologic manifestations in hospitalized patients with COVID-19 in Mexico City
SO PLOS ONE
LA English
DT Article
DE COVID-19; Neurologic manifestation; SARS-CoV-2; in-hospital; epilepsy; Seizure
AB Background
The coronavirus disease 2019 (COVID-19) is a systemic entity that frequently implies neurologic features at presentation and complications during the disease course. We aimed to describe the characteristics and predictors for developing in-hospital neurologic manifestations in a large cohort of hospitalized patients with COVID-19 in Mexico City.
Methods
We analyzed records from consecutive adult patients hospitalized from March 15 to June 30, 2020, with moderate to severe COVID-19 confirmed by reverse transcription real-time polymerase chain reaction (rtRT-PCR) for the severe acute respiratory syndrome coronavirus 2 (SARS-CoV-2). Neurologic syndromes were actively searched by a standardized structured questionnaire and physical examination, confirmed by neuroimaging, neurophysiology of laboratory analyses, as applicable.
Results
We studied 1,072 cases (65% men, mean age 53.2 +/- 13 years), 71 patients had pre-existing neurologic diseases (diabetic neuropathy: 17, epilepsy: 15, history of ischemic stroke: eight, migraine: six, multiple sclerosis: one, Parkinson disease: one), and 163 (15.2%) developed a new neurologic complication. Headache (41.7%), myalgia (38.5%), dysgeusia (8%), and anosmia (7%) were the most common neurologic symptoms at hospital presentation. Delirium (13.1%), objective limb weakness (5.1%), and delayed recovery of mental status after sedation withdrawal (2.5%), were the most common new neurologic syndromes. Age, headache at presentation, preexisting neurologic disease, invasive mechanical ventilation, and neutrophil/lymphocyte ratio >= 9 were independent predictors of new in-hospital neurologic complications.
Conclusions
Even after excluding initial clinical features and pre-existing comorbidities, new neurologic complications in hospitalized patients with COVID-19 are frequent and can be predicted from clinical information at hospital admission.
C1 [Flores-Silva, Fernando Daniel; Garcia-Grimshaw, Miguel; Valdes-Ferrer, Sergio Ivan; Vigueras-Hernandez, Alma Poema; Dominguez-Moreno, Rogelio; Tristan-Samaniego, Dioselina Panama; Michel-Chavez, Anaclara; Gonzalez-Duarte, Alejandra; Vega-Boada, Felipe A.; Reyes-Melo, Isael; Chavez-Martinez, Oswaldo Alan; Rebolledo-Garcia, Daniel; Marche-Fernandez, Osvaldo Alexis; Sanchez-Torres, Samantha; Garcia-Ramos, Guillermo; Cantu-Brito, Carlos; Chiquete, Erwin] Inst Nacl Ciencias Med & Nutr Salvador Zubiran, Dept Neurol & Psychiat, Mexico City, DF, Mexico.
[Valdes-Ferrer, Sergio Ivan] Feinstein Inst Med Res, Ctr Biomed Sci, Manhasset, NY USA.
[Jimenez-Ruiz, Amado] Western Univ, Stroke Dementia & Heart Dis Lab, London, ON, Canada.

C3 Instituto Nacional de Ciencias Medicas y Nutricion Salvador Zubiran - Mexico; Northwell Health; Western University (University of Western Ontario)
RP Cantu-Brito, C; Chiquete, E (通讯作者), Inst Nacl Ciencias Med & Nutr Salvador Zubiran, Dept Neurol & Psychiat, Mexico City, DF, Mexico.
EM carloscantu_brito@hotmail.com; erwin.chiquetea@incmnsz.mx
TC 12
Z9 12
PD APR 8
PY 2021
VL 16
IS 4
AR e0247433
DI 10.1371/journal.pone.0247433
WC Multidisciplinary Sciences
ER

PT J
AU Agrawal, M
Tripathi, M
Samala, R
Doddamani, R
Ramanujan, B
Chandra, PS
AF Agrawal, Mohit
Tripathi, Manjari
Samala, Raghu
Doddamani, Ramesh
Ramanujan, Bhargavi
Chandra, P. Sarat
TI Epilepsy surgery in COVID times-a unique conundrum
SO CHILDS NERVOUS SYSTEM
LA English
DT Article
DE Coronavirus; Drug resistant epilepsy; Endoscopic hemispherotomy; Infection
ID PEOPLE
AB The COVID-19 pandemic has forced hospitals to prioritize admissions. Epilepsy surgeries have been postponed at most centers. As the pandemic continues with no definite end in sight in the near future, the question arises until when such patients should be denied appropriate treatment. A 12-year-old child with left-sided Rasmussen's encephalitis with drug refractory epilepsy (DRE) presented at the height of the pandemic, with worsening of seizure frequency from 4-5/day to 20/day, with new-onset epilepsia partialis continua. She demonstrated features of progressive cognitive decline. The pros and cons of operating during the pandemic were discussed with the parents by a multidisciplinary team. She underwent endoscopic left hemispherotomy. Postoperatively she became seizure free but developed hospital-acquired mild COVID infection for which she was treated accordingly. Chosen cases of severe DRE, as the one illustrated above,

who are deemed to benefit from surgery by a multidisciplinary team of physicians, should be re-categorized into the most severe class of patients and scheduled for surgery as soon as possible. The risk benefit ratio of the seizures being mitigated by surgery on one hand and possibility of acquiring COVID infection during hospital stay has to be balanced and a decision made accordingly.
C1 [Agrawal, Mohit; Samala, Raghu; Doddamani, Ramesh; Chandra, P. Sarat] All India Inst Med Sci, Neurosci Ctr, Dept Neurosurg, Room 607, New Delhi 110029, India.
[Tripathi, Manjari; Ramanujan, Bhargavi] All India Inst Med Sci, Dept Neurol, New Delhi, India.
C3 All India Institute of Medical Sciences (AIIMS) New Delhi; All India Institute of Medical Sciences (AIIMS) New Delhi
RP Chandra, PS (通讯作者), All India Inst Med Sci, Neurosci Ctr, Dept Neurosurg, Room 607, New Delhi 110029, India.
EM saratpchandra3@gmail.com
TC 0
Z9 0
PD OCT
PY 2021
VL 37
IS 10
BP 3219
EP 3224
DI 10.1007/s00381-021-05048-4
EA APR 2021
WC Clinical Neurology; Pediatrics; Surgery
ER

PT J
AU Celik, H
Acikel, SB
Ozdemir, FMA
Aksoy, E
Oztoprak, U
Cucu, E
Kucur, O
Ceylan, N
Yuksel, D
AF Celik, Halil
Acikel, Sadettin Burak
Ozdemir, Fatih Mehmet Akif
Aksoy, Erhan
Oztoprak, Ulkuhan
Cucu, Ergin
Kucur, Ozge
Ceylan, Nesrin
Yuksel, Deniz
TI Evaluation of the Anxiety Level of Mothers of Children with Epilepsy

during the COVID-19 Pandemic Period
SO EUROPEAN NEUROLOGY
LA English
DT Article
DE COVID-19; Pandemic; Child epilepsy; anxiety
AB Background and Aim: Although anyone can be affected by the COVID-19 pandemic, it may cause additional concern for people with chronic conditions. Epilepsy is the most common neurological disease in childhood and adolescence. The aim of this study was to determine anxiety levels among the mothers of children under follow-up for epilepsy in our clinic during the COVID-19 pandemic. Methods: The study group consisted of the mothers of epilepsy patients who were under follow-up in the pediatric neurology outpatient clinic of the tertiary care center and were scheduled for a routine examination during the COVID-19 pandemic. The mothers' anxiety levels according to the Beck Anxiety Inventory and their opinions about COVID-19 in relation to their child were assessed and compared based on whether the mother/patient attended their appointments in person and whether the child had frequent or infrequent seizures. Results: There was no statistically significant difference in anxiety level between the mothers of 64 children with epilepsy who attended their appointment during the pandemic and those of the mothers of 52 who did not attend their appointment. However, the mothers of children with frequent seizures had significantly higher anxiety levels. Conclusion: Anxiety level of mothers whose children have frequent seizures was significantly higher compared to mothers whose children have infrequent seizures. It is important to be aware about this point and using telemedicine approach in suitable population and postpone routine outpatient follow-up appointments as much as possible.
C1 [Celik, Halil; Ozdemir, Fatih Mehmet Akif; Aksoy, Erhan; Oztoprak, Ulkuhan; Cucu, Ergin; Kucur, Ozge; Ceylan, Nesrin; Yuksel, Deniz] Univ Hlth Sci, Matern & Childrens Hlth & Dis Training & Res Hosp, Dept Pediat Neurol DY, Ankara, Turkey.
[Acikel, Sadettin Burak] Univ Hlth Sci, Matern & Childrens Hlth & Dis Training & Res Hosp, Child & Adolescent Psychiat Dept, Ankara, Turkey.
C3 University of Health Sciences Turkey; University of Health Sciences Turkey
RP Celik, H (通讯作者), Univ Hlth Sci, Matern & Childrens Hlth & Dis Training & Res Hosp, Dept Pediat Neurol DY, Ankara, Turkey.
EM celikdrh@gmail.com
TC 0
Z9 0
PD MAY
PY 2021
VL 84
IS 3
BP 192
EP 199
DI 10.1159/000514826
EA APR 2021
WC Clinical Neurology; Neurosciences
ER

PT J
AU Shulman, JG
   Ford, T
   Cervantes-Arslanian, AM
AF Shulman, Julie G.
   Ford, Thomas
   Cervantes-Arslanian, Anna M.
TI Neurologic Emergencies during the Coronavirus Disease 2019 Pandemic
SO NEUROLOGIC CLINICS
LA English
DT Article
DE COVID-19; Neurologic emergencies; Cerebrovascular disease; Thrombolysis;
   Seizure
ID ACUTE ISCHEMIC-STROKE; MANAGEMENT; COAGULOPATHY; COVID-19; EPILEPSY
C1 [Shulman, Julie G.; Ford, Thomas; Cervantes-Arslanian, Anna M.] Boston Univ, Dept Neurol, Sch Med, 72 East Concord St,Suite C3, Boston, MA 02118 USA.
   [Cervantes-Arslanian, Anna M.] Boston Univ, Dept Neurosurg, Sch Med, 725 Albany St,Suite 7C, Boston, MA 02118 USA.
   [Cervantes-Arslanian, Anna M.] Boston Univ, Dept Med Infect Dis, Sch Med, 801 Massachusetts Ave,2nd Floor, Boston, MA 02118 USA.
C3 Boston University; Boston University; Boston University
RP Shulman, JG (通讯作者), Boston Univ, Dept Neurol, Sch Med, 72 East Concord St,Suite C3, Boston, MA 02118 USA.
EM Julie.Shulman@bmc.org
TC 0
Z9 0
PD MAY
PY 2021
VL 39
IS 2
BP 671
EP 687
DI 10.1016/j.ncl.2021.02.007
EA APR 2021
WC Clinical Neurology; Neurosciences
ER

PT J
AU Lallana, S
   Fonseca, E
   Restrepo, JL
   Quintana, M
   Seijo-Raposo, I
   Abraira, L
   Santamarina, E
   Alvarez-Sabin, J
   Toledo, M

AF Lallana, Sofia
Fonseca, Elena
Restrepo, Juan Luis
Quintana, Manuel
Seijo-Raposo, Ivan
Abraira, Laura
Santamarina, Estevo
Alvarez-Sabin, Jose
Toledo, Manuel
TI Medium-term effects of COVID-19 pandemic on epilepsy: A follow-up study
SO ACTA NEUROLOGICA SCANDINAVICA
LA English
DT Article
DE COVID-19; depression; epilepsy; pandemic; SARS-CoV-2; Seizure; telemedicine
ID HOSPITAL ANXIETY; HEALTH-CARE; DEPRESSION; PEOPLE; PREVALENCE; IMPACT
AB Objective To analyze the medium-term impact of the COVID-19 pandemic on epilepsy patients, focusing on psychological effects and seizure control.

Methods Prospective follow-up study to evaluate the medium-term effects of the COVID-19 pandemic on a cohort of epilepsy patients from a tertiary hospital previously surveyed during the first peak of the pandemic. Between July 1, 2020, and August 30, 2020, the patients answered an online 19-item questionnaire, HADS, and PSIQ scales. Short- and medium-term effects of the pandemic confinement and the perception of telemedicine were compared.

Results 153 patients completed the questionnaire, mean +/- SD age, 47.6 +/- 19.3 years; 49.7% women. Depression was reported by 43 patients, significantly more prevalent than in the short-term analysis (29.2% vs. 19.7%; p = .038). Anxiety (38.1% vs. 36.1%; p = 0.749) and insomnia (28.9% vs. 30.9%, p = .761) remained highly prevalent. Seventeen patients reported an increase in seizure frequency (11.1% vs. 9.1%, p = .515). The three factors independently associated with an increase in seizure frequency in the medium term were drug-resistant epilepsy (odds ratio [OR] = 8.2, 95% CI 2.06-32.52), depression (OR = 6.46, 95% CI 1.80-23.11), and a reduction in income (OR = 5.47, 95% CI 1.51-19.88). A higher proportion of patients found telemedicine unsatisfactory (11.2% vs. 2.4%), and a lower percentage (44.8% vs. 56.8%) found it very satisfactory (p = .005).

Conclusions Depression rates increased significantly after the first wave. Depression, drug-resistant epilepsy, and a reduction in family income were independent risk factors for an increased seizure frequency. Perception of telemedicine worsened, indicating need for re-adaptation.
C1 [Lallana, Sofia; Restrepo, Juan Luis; Alvarez-Sabin, Jose] Vall dHebron Univ Hosp, Neurol Dept, Barcelona, Spain.

[Fonseca, Elena; Quintana, Manuel; Seijo-Raposo, Ivan; Abraira, Laura; Santamarina, Estevo; Toledo, Manuel] Vall dHebron Univ Hosp, Neurol Dept, Epilepsy Unit, Barcelona, Spain.

[Fonseca, Elena; Quintana, Manuel; Seijo-Raposo, Ivan; Abraira, Laura; Santamarina, Estevo; Toledo, Manuel] Univ Autonoma Barcelona, Med Dept, Barcelona, Spain.
C3 Hospital Universitari Vall d'Hebron; Hospital Universitari Vall d'Hebron; Autonomous University of Barcelona

RP Fonseca, E (通讯作者)，Vall dHebron Univ Hosp, Neurol Dept, Epilepsy Unit, Passeig Vall dHebron 119-129, Barcelona 08035, Spain.
EM e.fonseca@vhebron.net
TC 0
Z9 0
PD JUL
PY 2021
VL 144
IS 1
BP 99
EP 108
DI 10.1111/ane.13439
EA APR 2021
WC Clinical Neurology
ER

PT J
AU Vossler, DG
AF Vossler, David G.
TI COVID-19 Incidence and Death Rate in Epilepsy: Too Early to Tell?
SO EPILEPSY CURRENTS
LA English
DT Editorial Material
DE COVID-19; Incidence; Death Rate; Epilepsy
TC 0
Z9 0
PD JUL
PY 2021
VL 21
IS 4
BP 261
EP 263
AR 15357597211014195
DI 10.1177/15357597211014195
EA APR 2021
WC Clinical Neurology
ER

PT J
AU Clary, HMM
Wan, MY
Conner, K
Brenes, GA
Kimball, J
Kim, E
Duncan, P
Snively, BM

AF Clary, Heidi M. Munger
Wan, Mingyu
Conner, Kelly
Brenes, Gretchen A.
Kimball, James
Kim, Esther
Duncan, Pamela
Snively, Beverly M.
TI Outcomes of seizures, status epilepticus, and EEG findings in critically ill patient with COVID-19
SO EPILEPSY & BEHAVIOR
LA English
DT Article
DE COVID-19; Coronavirus; SARs-CoV-2; EEG; Functional outcomes; Seizure
ID GENERALIZED ANXIETY DISORDER; MAJOR DEPRESSION; SCREENING TOOLS; RAPID DETECTION; EPILEPSY; INSTRUMENTS
AB Objective: Severe acute respiratory syndrome coronavirus 2 (SARS-CoV-2) infection has a myriad of neurological manifestations and its effects on the nervous system are increasingly recognized. Seizures and status epilepticus (SE) are reported in the novel coronavirus disease (COVID-19), both new onset and worsening of existing epilepsy; however, the exact prevalence is still unknown. The primary aim of this study was to correlate the presence of seizures, status epilepticus, and specific critical care EEG patterns with patient functional outcomes in those with COVID-19.

Methods: This is a retrospective, multicenter cohort of COVID-19-positive patients in Southeast Michigan who underwent electroencephalography (EEG) from March 12th through May 15th, 2020. All patients had confirmed nasopharyngeal PCR for COVID-19. EEG patterns were characterized per 2012 ACNS critical care EEG terminology. Clinical and demographic variables were collected by medical chart review. Outcomes were divided into recovered, recovered with disability, or deceased.

Results: Out of the total of 4100 patients hospitalized with COVID-19, 110 patients (2.68%) had EEG during their hospitalization; 64% were male, 67% were African American with mean age of 63 years (range 20-87). The majority (70%) had severe COVID-19, were intubated, or had multi-organ failure. The median length of hospitalization was 26.5 days (IQR = 15 to 44 days). During hospitalization, of the patients who had EEG, 21.8% had new-onset seizure including 7% with status epilepticus, majority (87.5%) with no prior epilepsy. Forty-nine (45%) patients died in the hospital, 46 (42%) recovered but maintained a disability and 15 (14%) recovered without a disability. The EEG findings associated with outcomes were background slowing/attenuation (recovered 60% vs recovered/disabled 96% vs died 96%, $p < 0.001$) and normal (recovered 27% vs recovered/disabled 0% vs died 1%, $p < 0.001$). However, these findings were no longer significant after adjusting for severity of COVID-19.

Conclusion: In this large multicenter study from Southeast Michigan, one of the early COVID-19 epicenters in the US, none of the EEG findings were significantly correlated with outcomes in critically ill COVID-19 patients. Although seizures and status epilepticus could be encountered in COVID-19, the occurrence did not correlate with the patients' functional outcome. (C) 2021 Elsevier Inc. All rights reserved.
C1 [Clary, Heidi M. Munger; Conner, Kelly; Kim, Esther; Duncan, Pamela] Wake Forest

Sch Med, Dept Neurol, Winston Salem, NC 27101 USA.
[Wan, Mingyu] Wake Forest Univ, Neurosci Grad Program, Winston Salem, NC 27101 USA.
[Brenes, Gretchen A.] Wake Forest Sch Med, Sect Gerontol & Geriatr Med, Dept Internal Med, Winston Salem, NC 27101 USA.
[Kimball, James] Wake Forest Sch Med, Dept Psychiat, Winston Salem, NC 27101 USA.
[Snively, Beverly M.] Wake Forest Sch Med, Dept Biostat & Data Sci, Winston Salem, NC 27101 USA.
C3 Wake Forest University; Wake Forest University; Wake Forest University;
Wake Forest University; Wake Forest University
RP Clary, HMM (通讯作者), 1 Med Ctr Blvd, Winston Salem, NC 27157 USA.
EM hmungerc@wakehealth.edu; MingyuWa-n2021@u.northwestern.edu;
kconner@wakehealth.edu; gbrenes@wakehealth.edu; jkimball@wakehealth.edu;
EKKim@mednet.ucla.edu; pduncan@wakehealth.edu; bmel-len@wakehealth.edu
TC 0
Z9 0
PD MAY
PY 2021
VL 118
AR 107923
WC Behavioral Sciences; Clinical Neurology; Psychiatry
ER

PT J
AU Zeng, C
Meng, HM
Zhu, YL
Yao, LF
Lian, YJ
Zhu, YM
Zhang, M
Dai, YW
Wang, K
Han, X
Li, L
Zhang, LF
Xu, HQ
Yao, DA
Luo, XM
Jiang, W
Wang, XH
Zhao, CS
Chen, YM
Deng, XJ
Liu, CR
Li, F
Song, YM
Wu, Y

Liao, WP
Wang, FR
Zhu, SQ
Xiao, B
Wang, Q
Long, LL
AF Zeng, Chang
Meng, Hongmei
Zhu, Yulan
Yao, Lifen
Lian, Yajun
Zhu, Yanmei
Zhang, Min
Dai, Yuwei
Wang, Kang
Han, Xiong
Li, Ling
Zhang, Lifang
Xu, Huiqing
Yao, Dongai
Luo, Xinmin
Jiang, Wen
Wang, Xiahong
Zhao, Chuansheng
Chen, Yangmei
Deng, Xuejun
Liu, Chaorong
Li Feng
Song, Yanmin
Wu, Yuan
Liao, Weiping
Wang, Furong
Zhu, Suiqiang
Xiao, Bo
Wang, Qun
Long, Lili
TI Correlation of Seizure Increase and COVID-19 Outbreak in Adult Patients with Epilepsy: Findings and Suggestions from a Nationwide Multi-centre Survey in China
SO SEIZURE-EUROPEAN JOURNAL OF EPILEPSY
LA English
DT Article
DE COVID-19; Epilepsy; Seizure control; Mental health
ID ANXIETY; VALIDATION; WITHDRAWAL; DISORDERS; PEOPLE; IMPACT; DEATH; SARS
AB Objectives: To investigate the impact of the COVID-19 outbreak on the behaviours, mental health and seizure control of adult patients with epilepsy (PWE) and to identify the correlation of seizure increase and the COVID-19 outbreak to guide the medical care

of individuals with epilepsy during a public health crisis.

Methods: This study was conducted at 28 centres from February 2020 to April 2020. Participants filled out a 62 item online survey including sociodemographic, COVID-19-related, epilepsy-related and psychological variables and were divided into two groups based on whether their seizure frequency increased during the COVID-19 pandemic. Chi-square tests and t-tests were used to test differences in significant characteristics. Multiple logistic regression analyses were used to identify risk factors for seizure worsening.

Results: A total of 1,237 adult PWE were enrolled for analysis. Of this sample, 31 (8.33%) patients experienced an increase in seizures during the pandemic. Multivariate logistic regression suggested that feeling nervous about the pandemic (P < 0.05), poor quality of life (P = 0.001), drug reduction/withdrawal (P = 0.032), moderate anxiety during the COVID-19 outbreak (P = 0.046) and non-seizure free before the COVID-19 outbreak (P < 0.05) were independently related to seizure increase during the pandemic.

Conclusions: During the COVID-19 pandemic, PWE with poor quality of life and mental status, as well as AED reduction/withdrawal, were more likely to experience seizure increase. This observation highlights the impor-tance of early identification of the population at high risk of seizure worsening and implementation of preventive strategies during the pandemic.

Results: A total of 1,237 adult PWE were enrolled for analysis. Of this sample, 31 (8.33%) patients experienced an increase in seizures during the pandemic. Multivariate logistic regression suggested that feeling nervous about the pandemic (P < 0.05), poor quality of life (P = 0.001), drug reduction/withdrawal (P = 0.032), moderate anxiety during the COVID-19 outbreak (P = 0.046) and non-seizure free before the COVID-19 outbreak (P < 0.05) were independently related to seizure increase during the pandemic.
Conclusions: During the COVID-19 pandemic, PWE with poor quality of life and mental status, as well as AED reduction/withdrawal, were more likely to experience seizure increase. This observation highlights the importance of early identification of the population at high risk of seizure worsening and implementation of preventive
C1 [Zeng, Chang] Cent South Univ, Xiangya Hosp, Hlth Management Ctr, Changsha, Peoples R China.

[Meng, Hongmei] First Hosp Jilin Univ, Dept Neurol, Changchun, Peoples R China.

[Zhu, Yulan; Zhu, Yanmei] Harbin Med Univ, Dept Neurol, Affiliated Hosp 2, Harbin, Peoples R China.

[Yao, Lifen] Harbin Med Univ, Dept Neurol, Affiliated Hosp 1, Harbin, Peoples R China.

[Lian, Yajun] Zhengzhou Univ, Dept Neurol, Affiliated Hosp 1, Zhengzhou, Peoples R China.

[Zhang, Min; Dai, Yuwei; Liu, Chaorong; Li Feng; Xiao, Bo; Long, Lili] Cent South Univ, Xiangya Hosp, Dept Neurol, 87 Xiangya Rd, Changsha 410008, Peoples R China.

[Wang, Kang] Zhejiang Univ, Affiliated Hosp 1, Coll Med, Dept Neurol, Hangzhou, Peoples R China.

[Han, Xiong] Henan Prov Peoples Hosp, Dept Neurol, Zhengzhou, Peoples R China.

[Li, Ling] Shandong Univ, Dept Neurol, Qilu Hosp, Jinan, Peoples R China.

[Zhang, Lifang] Changzhi Med Coll, Dept Neurol, Changzhi Peoples Hosp, Changzhi, Peoples R China.

[Xu, Huiqing] Wenzhou Med Univ, Dept Neurol, Affiliated Hosp 1, Wenzhou, Peoples

R China.
[Yao, Dongai] Wuhan Univ, Dept Neurol, Zhongnan Hosp, Wuhan, Peoples R China.
[Luo, Xinmin] Nanchang Univ, Dept Neurol, Affiliated Hosp 2, Nanchang, Jiangxi, Peoples R China.
[Jiang, Wen] Air Force Mil Med Univ, Xijing Hosp, Dept Neurol, Xian, Peoples R China.
[Wang, Xiahong] Zhengzhou Second Hosp, Dept Neurol, Zhengzhou, Peoples R China.
[Zhao, Chuansheng] China Med Univ, Dept Neurol, Hosp 1, Shenyang, Peoples R China.
[Chen, Yangmei] Chongqing Med Univ, Dept Neurol, Affiliated Hosp 2, Chongqing, Peoples R China.
[Deng, Xuejun] Huazhong Univ Sci & Technol, Dept Neurol, Union Hosp, Tongji Med Coll, Wuhan, Peoples R China.
[Song, Yanmin] Cent South Univ, Xiangya Hosp, Dept Emergency, Changsha, Peoples R China.
[Wu, Yuan] Guangxi Med Univ, Dept Neurol, Affiliated Hosp 1, Nanning, Peoples R China.
[Liao, Weiping] Guangzhou Med Univ Neurosci, Key Lab Neurogenet & Channelopathies Guangdong In, Dept Neurol, Affiliated Hosp 1, Guangzhou, Peoples R China.
[Liao, Weiping] Minist Educ China, Guangzhou, Peoples R China.
[Wang, Furong; Zhu, Suiqiang] Huazhong Univ Sci & Technol, Dept Neurol, Tongji Hosp, Tongji Med Coll, Wuhan, Peoples R China.
[Wang, Qun] Capital Med Univ, Beijing Tiantan Hosp, Dept Neurol, Beijing, Peoples R China.
C3 Central South University; Jilin University; Harbin Medical University; Harbin Medical University; Zhengzhou University; Central South University; Zhejiang University; Zhengzhou University; Shandong University; Changzhi Medical College; Wenzhou Medical University; Wuhan University; Nanchang University; Air Force Military Medical University; China Medical University; Chongqing Medical University; Huazhong University of Science & Technology; Central South University; Guangxi Medical University; Ministry of Education, China; Huazhong University of Science & Technology; Capital Medical University
RP Long, LL (通讯作者), Cent South Univ, Xiangya Hosp, Dept Neurol, 87 Xiangya Rd, Changsha 410008, Peoples R China.; Wang, Q (通讯作者), Capital Med Univ, Beijing Tiantan Hosp, 119 South 4th Ring West Rd, Beijing, Peoples R China.
EM qwang64@163.com; longlili1982@126.com
TC 5
Z9 5
PD MAY
PY 2021
VL 88
BP 102
EP 108
DI 10.1016/j.seizure.2021.03.029
WC Clinical Neurology; Neurosciences
ER

PT J

AU Ayatollahi, P
   Tarazi, A
   Wennberg, R
AF Ayatollahi, Parisa
   Tarazi, Apameh
   Wennberg, Richard
TI Possible Autoimmune Encephalitis with Claustrum Sign in case of Acute
   SARS-CoV-2 Infection
SO CANADIAN JOURNAL OF NEUROLOGICAL SCIENCES
LA English
DT Letter
DE COVID-19; Epilepsy; encephalitis; Magnetic resonance imaging (MRI); Seizure;
   Steroid immunotherapy
C1 [Ayatollahi, Parisa] Yazd Univ Med Sci, Shahid Sadoughi Hosp, Dept Neurol, Yazd, Iran.
   [Tarazi, Apameh] Univ Toronto, Toronto Western Hosp, Div Neurol, Dept Med, Toronto, ON, Canada.
   [Wennberg, Richard] Univ Toronto, Toronto Western Hosp, Div Neurol, Dept Med, Toronto, ON, Canada.
C3 University of Toronto; University Health Network Toronto; University of
   Toronto; University Health Network Toronto
RP Wennberg, R (通讯作者), Toronto Western Hosp, 399 Bathurst St, Toronto, ON M5T 2S8, Canada.
EM richard.wennberg@uhn.ca
TC 9
Z9 9
PD MAY
PY 2021
VL 48
IS 3
BP 430
EP 432
DI 10.1017/cjn.2020.209
WC Clinical Neurology
ER

PT J
AU de Lima, FS
   Issa, N
   Seibert, K
   Davis, J
   Wlodarski, R
   Klein, S
   El Ammar, F
   Wu, SS
   Rose, S
   Warnke, P

Tao, J
AF Santos de Lima, Fabiane
Issa, Naoum
Seibert, Kaitlin
Davis, Jared
Wlodarski, Richard
Klein, Sara
El Ammar, Faten
Wu, Shasha
Rose, Sandra
Warnke, Peter
Tao, James
TI Epileptiform activity and seizures in patients with COVID-19
SO JOURNAL OF NEUROLOGY NEUROSURGERY AND PSYCHIATRY
LA English
DT Letter
DE epilepsy; EEG; infectious diseases; MRI
C1 [Santos de Lima, Fabiane; Issa, Naoum; Seibert, Kaitlin; Davis, Jared; Wlodarski, Richard; Klein, Sara; El Ammar, Faten; Wu, Shasha; Rose, Sandra; Warnke, Peter; Tao, James] Univ Chicago, Dept Neurol, 5841 S Maryland Ave, Chicago, IL 60637 USA.
C3 University of Chicago
RP Tao, J (通讯作者), Univ Chicago, Div Biol Sci, Chicago, IL 60637 USA.
EM jtao@neurology.bsd.uchicago.edu
TC 8
Z9 8
PD MAY
PY 2021
VL 92
IS 5
BP 565
EP 566
DI 10.1136/jnnp-2020-324337
WC Clinical Neurology; Psychiatry; Surgery
ER

PT J
AU Bosak, M
Mazurkiewicz, I
Wezyk, K
Slowik, A
Turaj, W
AF Bosak, Magdalena
Mazurkiewicz, Iwona
Wezyk, Kamil
Slowik, Agnieszka
Turaj, Wojciech
TI COVID-19 among patients with epilepsy: Risk factors and course of the

disease
SO EPILEPSY & BEHAVIOR
LA English
DT Article
DE COVID-19; SARS-CoV-2; Epilepsy; Risk factor; Seizure
AB Introduction: The study assessed the prevalence and risk factors for SARS-CoV-2 infection in patients with epilepsy (PWE). Additionally, the course of COVID-19 and its impact on seizure control was investigated.

Material and methods: Subjects with definite (confirmed by positive RT-PCR nasopharyngeal swab or serum anti-SARS-CoV-2 antibodies) and probable COVID-19 were identified via telephone survey among PWE treated at the university epilepsy clinic.

Results: Of 252 screened subjects, 17 (6.7%) had definite and 14 (5.5%) probable COVID-19. The percentage of PWE with definite COVID-19 was much higher than the percentage of subjects with confirmed COVID-19 in Polish general population (3.65%). In the heterogenous population of PWE, including patients with drug-resistant epilepsy, physical/intellectual disability, and comorbidities, we were not able to identify any risk factors for contracting COVID-19. The course of infection was mild or moderate in all subjects, not requiring oxygen therapy or respiratory support. The most common symptoms were fever, fatigue, headaches, muscle aches, and loss of smell/taste and continued for approximately 7- 21 days, except for loss of smell/taste which lasted usually several weeks. Seizure exacerbation was noted in only one pregnant patient with confirmed COVID-19 and it was likely related to decreased serum level of levetiracetam in the third trimester.

Conclusion: The study provided reassuring findings related to the low risk of seizure exacerbation in PWE during the course of COVID-19. Patients with epilepsy may be at increased risk of SARS-CoV-2 infection. Epilepsy characteristics are not likely to modify the risk of COVID-19. (c) 2021 Elsevier Inc. All rights reserved.
C1 [Bosak, Magdalena; Slowik, Agnieszka; Turaj, Wojciech] Jagiellonian Univ Med Coll, Dept Neurol, Fac Med, Jakubowskiego 2 St, PL-30688 Krakow, Poland.
[Mazurkiewicz, Iwona; Wezyk, Kamil] Univ Hosp, Krakow, Poland.
C3 Jagiellonian University; Collegium Medicum Jagiellonian University
RP Bosak, M (通讯作者), Jagiellonian Univ Med Coll, Dept Neurol, Fac Med, Jakubowskiego 2 St, PL-30688 Krakow, Poland.
EM magdalena.bosak@uj.edu.pl
TC 3
Z9 3
PD JUL
PY 2021
VL 120
AR 107996
DI 10.1016/j.yebeh.2021.107996
EA MAY 2021
WC Behavioral Sciences; Clinical Neurology; Psychiatry
ER

PT J
AU Niimi, K

Fujimoto, A
Sato, K
Enoki, H
Okanishi, T
AF Niimi, Keiko
Fujimoto, Ayataka
Sato, Keishiro
Enoki, Hideo
Okanishi, Tohru
TI Patients With Epilepsy Who Underwent Epilepsy Surgery During the COVID-19 Pandemic Showed Less Depressive Tendencies
SO FRONTIERS IN NEUROLOGY
LA English
DT Article
DE Zung self-rating depression scale; pandemic; epilepsy; surgery; COVID-19; SARS-CoV-2
ID SYMPTOMS; FLU
AB Introduction: Our hypothesis in this study was that differences might exist between patients with epilepsy (PWE) who underwent epilepsy surgery before and within the period of the coronavirus disease 2019 (COVID-19) pandemic. The purpose of this study was to compare results of the Zung Self-Rating Depression Scale (SDS) between PWE who underwent epilepsy surgery before and during the pandemic period.

Methods: Participants were PWE who underwent open cranial epilepsy surgery between February 2019 and February 2021 in our hospital. Patients who underwent surgery in the first half of this period, between February 2019 and January 2020, were defined as the pre-pandemic period group (pre-Group) and those treated in the second half, between February 2020 and February 2021, were categorized as the pandemic period group (within-Group). All patients completed the SDS before surgery, and scores were compared between groups.

Results: SDS score was significantly higher in the pre-Group than in the within-Group ($p = 0.037$). Other factors, including age ($p = 0.51$), sex ($p = 0.558$), epilepsy duration from onset to SDS score evaluation ($p = 0.190$), seizure frequency ($p = 0.794$), number of anti-seizure medications ($p = 0.787$), and intelligence quotient ($p = 0.871$) did not differ significantly between groups.

Conclusion: SDS score was higher in the pre-pandemic group than in the within-pandemic group, which may indicate that PWE with less-positive outlooks may be less likely to seek medical attention during stressful periods.
C1 [Niimi, Keiko; Fujimoto, Ayataka; Sato, Keishiro; Enoki, Hideo; Okanishi, Tohru] Seirei Hamamatsu Gen Hosp, Comprehens Epilepsy Ctr, Hamamatsu, Shizuoka, Japan.
RP Fujimoto, A (通讯作者), Seirei Hamamatsu Gen Hosp, Comprehens Epilepsy Ctr, Hamamatsu, Shizuoka, Japan.
EM afujimotoscienceacademy@gmail.com
TC 1
Z9 1
PD MAY 4
PY 2021
VL 12

AR 677828
DI 10.3389/fneur.2021.677828
WC Clinical Neurology; Neurosciences
ER

PT J
AU Rios-Pohl, L
Franco, M
Gonzalez, M
AF Rios-Pohl, Loreto
Franco, Macarena
Gonzalez, Magdalena
TI Hyperventilation maneuver during EEG in children with epilepsy after the COVID-19 pandemic. Is a routine procedure necessary?
SO EPILEPSIA OPEN
LA English
DT Article
DE absence childhood epilepsy; clinical seizure; generalized epileptiform discharges; COVID-19; SARS-CoV-2; EEG; hyperventilation
ID ILAE COMMISSION; CLASSIFICATION; ACTIVATION; SEIZURES
AB Objective Hyperventilation (HV) is one of the main and basic activation methods during ambulatory electroencephalogram (EEG), unless medical reasons contraindicate it. During the COVID-19 pandemic, with the high risk of human-to-human infection, local guidelines and recommendations have been developed that suggest not to perform the HV maneuver routinely. Our objective was to characterize patients who present positive HV in an epilepsy center.

Methods We analyzed retrospectively all the ambulatory EEGs performed during one year in our specialized ambulatory child and adolescent epilepsy center, and describe patients with positive maneuver.

Results A total of 305 EEGs were performed. Patients under 3 years and 11 months were excluded as well as all patients that did not fill up the criteria for epilepsy diagnosis. From the 252 EEGs that were included in the study, 194 EEGs (77%) were classified as abnormal and 58 (23%) as normal. From these same 252 EEGs, 150 EEG finished correctly the HV maneuver. Physiological slowing response was found in 54 EEGs (36%), no changes (negative) in 83 (55%), and abnormal response (positive) in 13 EEGs (9%). The 13 HV-positive EEGs showed 4 patients with an increase of epileptiform activity, 3 patients experienced an increase of basal preregistered abnormal slowing, and 6 EEGs showed trigger of bilaterally synchronous and symmetric 2-4 Hz spike-and-slow wave discharges and absences. None of these last 6 patients needed more than 3 minutes to elicit the paroxysmal discharge.

Significance Based on these findings and according with other studies, the low positivity and high specificity of the HV maneuver support the idea that HV could be excluded during the COVID-19 pandemic situation, and also reevaluate whether it could be changed to a complementary maneuver, restricted only for cases where absence epilepsy is suspected. Larger studies will be needed to reaffirm this proposal.
C1 [Rios-Pohl, Loreto; Gonzalez, Magdalena] Clin Integral Epilepsia Infantojuvenil, Camino el Alba 9500, Santiago, Las Condes, Chile.

[Franco, Macarena; Gonzalez, Magdalena] Hosp San Pablo, Serv Neurol Infantil, Coquimbo, Chile.
[Gonzalez, Magdalena] Hosp Clin San Borja Arriaran, Santiago, Chile.
C3 Universidad de Chile
RP Rios-Pohl, L (通讯作者), Clin Integral Epilepsia Infantojuvenil, Camino el Alba 9500, Santiago, Las Condes, Chile.
EM lrios@clinicaepilepsia.cl
TC 0
Z9 0
PD JUN
PY 2021
VL 6
IS 2
BP 437
EP 442
DI 10.1002/epi4.12493
EA MAY 2021
WC Clinical Neurology; Neurosciences
ER

PT J
AU Manzo, ML
Galati, C
Gallo, C
Santangelo, G
Marino, A
Guccione, F
Pitino, R
Raieli, V
AF Manzo, Maria Laura
Galati, Cristina
Gallo, Cristina
Santangelo, Giuseppe
Marino, Antonio
Guccione, Fulvio
Pitino, Renata
Raieli, Vincenzo
TI ADEM post-Sars-CoV-2 infection in a pediatric patient with Fisher-Evans syndrome
SO NEUROLOGICAL SCIENCES
LA English
DT Article
DE Headache; Epilepsy; ADEM; Fisher-Evans Syndrome; Autoimmune disease
AB Introduction Sars-CoV-2 is a single-strained RNA virus belonging to Coronaviridae's family. In pediatric age, the majority of patients is asymptomatic; however, several neurological manifestations associated with Sars-CoV-2 infection have been detected in a percentage of cases ranging from 17.3 to 36.4%. Acute disseminated

encephalomyelitis (ADEM) has been recently included among the potential complications of Sars-Cov2 infection. The available data regarding pediatric patient show only one case. Case report We present a case regarding a 6-year-old patient suffering from Fisher-Evans syndrome who was given sirolimus and thalidomide therapy. After 10 days since the first positive nasopharyngeal swab for Sars-CoV-2, in which he had no symptoms, he presented an episode of generalized tonic-clonic seizure with spontaneous resolution. The patient underwent MRI which showed the typical picture of acute disseminated encephalomyelitis. His clinical course was favorable, with a good response to cortisone therapy and a progressive improvement of the neuroradiological and electroencephalographic picture. Conclusions According to our knowledge, this is the second case of an acute disseminated encephalomyelitis following SARS-CoV-2 infection in a pediatric patient, characterized by monosymptomatic onset, in which the immunosuppressive therapy practiced for the Fisher-Evans syndrome has probably contributed to a favorable evolution of ADEM, in contrast to other case described in the literature.
C1 [Manzo, Maria Laura; Galati, Cristina; Marino, Antonio; Guccione, Fulvio] Univ Palermo, Child Neuropsychiat Sch, Piazzale Clin, I-90100 Palermo, Italy.
[Gallo, Cristina] UOC Neuroradiol ARNAS Civ, Piazzale Nicola Leotta 4, I-90100 Palermo, Italy.
[Santangelo, Giuseppe; Pitino, Renata; Raieli, Vincenzo] ISMEP PO Cristina ARNAS Civ, Child Neuropsychiat Unit, Via Benedettini 1, I-90100 Palermo, Italy.
C3 University of Palermo
RP Manzo, ML (通讯作者), Univ Palermo, Child Neuropsychiat Sch, Piazzale Clin, I-90100 Palermo, Italy.
EM marialauramanzo@yahoo.it; cgalati.cg@gmail.com; crilla.gallo@gmail.com;
giuseppe.santangelo@arnascivico.it; antoniomarino94@tiscali.it;
fulvio.guc@gmail.com; renapiti@gmail.com; vincenzoraieli@gmail.com
TC 1
Z9 1
PD OCT
PY 2021
VL 42
IS 10
BP 4293
EP 4296
DI 10.1007/s10072-021-05311-1
EA MAY 2021
WC Clinical Neurology; Neurosciences
ER

PT J
AU Strizovic, S
Vojvodic, N
Kovacevic, M
Pejovic, A
Bukumiric, Z
Sokic, D

Ristic, AJ
AF Strizovic, Selena
Vojvodic, Nikola
Kovacevic, Masa
Pejovic, Aleksa
Bukumiric, Zoran
Sokic, Dragoslav
Ristic, Aleksandar J.
TI Influence of COVID-19 pandemic on quality of life in patients with epilepsy-Follow-up study
SO EPILEPSY & BEHAVIOR
LA English
DT Article
DE Quality of life; QOLIE-31; NDDI-E; Depression; Epilepsy; COVID-19
ID DEPRESSION
AB Objective: To perform a follow-up study of the quality of life in patients with epilepsy in the era of the COVID-19 crisis. Methods: Two months before the first case of the COVID-19 in Serbia, we obtained the Serbian Version of Quality of Life Inventory for Epilepsy 31 (SVQOLIE-31) and Neurological Disorders Depression Inventory for Epilepsy scores (SVNDDI-E) for another study. We retested the same patients one year after in COVID19 pandemic. In addition to SVQOLIE-31, and SVNDDI-E we used a generic questionnaire compiled from items related to the COVID-19. Results: We retested 97 out of 118 patients (82.2%) for the follow-up analysis. The average age was 36.1 +/- 12.2 (range: 18-69), and 49 were women (50.5%). The median duration of epilepsy was 13 years (range: 1.5-48). The structural etiology of epilepsy was noted in 41 (42.3%), unknown etiology in 41 (42.3%), and genetic etiology in 15 (15.4%) patients. Fewer patients (27.8%) experienced at least one seizure three months before follow-up testing when compared to patients who experienced at least one seizure three months in initial testing (36.0%) (p = 0.15). All patients reported full compliance with anti seizure medication in the follow-up. The SVQOLIE-31 score during the COVID-19 pandemic visit (64.5 +/- 14.6) was significantly lower than the SVQOLIE-31 score before the pandemic (p < 0.001). The SVNDDI-E score during the COVID-19 pandemic (10.5 +/- 3.5) was significantly higher than the SVNDDIE score before it (p < 0.001). Multiple linear regression analyses revealed fear of seizures, and fear of a reduction in household income, significantly associated with SVQOLIE-31 and SVNDDI-E overall score. These variables accounted for 66% and 27% of the variance of SVQOLIE-31 and SVNDDI-E overall score. Significance: Lower quality of life, higher prevalence of depression, healthcare availability issues, and perceived fears during pandemic all suggest COVID-19 has negatively impacted lives of patients with epilepsy. (c) 2021 Elsevier Inc. All rights reserved.
C1 [Strizovic, Selena] Univ Belgrade, Fac Med, Belgrade, Serbia.
[Vojvodic, Nikola; Kovacevic, Masa; Pejovic, Aleksa; Sokic, Dragoslav; Ristic, Aleksandar J.] Univ Belgrade, Univ Clin Ctr Serbia, Fac Med, Clin Neurol, Dr Subot 6, Belgrade 11000, Serbia.
[Bukumiric, Zoran] Univ Belgrade, Fac Med, Med Stat & Informat, Belgrade, Serbia.
C3 University of Belgrade; Clinical Centre of Serbia; University of Belgrade; University of Belgrade

RP Ristic, AJ (通讯作者), Univ Belgrade, Univ Clin Ctr Serbia, Fac Med, Clin Neurol, Dr Subot 6, Belgrade 11000, Serbia.
EM aristic@eunet.rs
TC 4
Z9 4
PD AUG
PY 2021
VL 121
AR 108026
DI 10.1016/j.yebeh.2021.108026
EA MAY 2021
WC Behavioral Sciences; Clinical Neurology; Psychiatry
ER

PT J
AU Fagbemigun, R
   Benchluch, A
   Le, K
   Diaz, PL
   Lewis, EC
AF Fagbemigun, Richard
   Benchluch, Ashley
   Le, Kevin
   Diaz, Patrick L.
   Lewis, Evan Cole
TI Implementation of a Virtual Rapid Access Epilepsy Clinic
SO CANADIAN JOURNAL OF NEUROLOGICAL SCIENCES
LA English
DT Letter; Early Access
DE Rapid access; Virtual medicine; VEC; COVID-19; Epilepsy
C1 [Fagbemigun, Richard; Benchluch, Ashley; Le, Kevin; Diaz, Patrick L.; Lewis, Evan Cole] Neurol Ctr Toronto, 491 Eglinton Ave West, Suite 100, Toronto, ON M5N 1A8, Canada.
RP Lewis, EC (通讯作者), Neurol Ctr Toronto, 491 Eglinton Ave West, Suite 100, Toronto, ON M5N 1A8, Canada.
EM evan.lewis@neurologycentretoronto.com
TC 0
Z9 0
DI 10.1017/cjn.2021.111
EA MAY 2021
WC Clinical Neurology
ER

PT J
AU Jobst, BC
   Conner, KR
   Coulter, D
   Fried, I

Guilfoyle, S
Hirsch, LJ
Hogan, RE
Hopp, JL
Naritoku, D
Plueger, M
Schevon, C
Smith, G
Valencia, I
Gaillard, WD
AF Jobst, Barbara C.
Conner, Kelly R.
Coulter, Douglas
Fried, Itzhak
Guilfoyle, Shanna
Hirsch, Lawrence J.
Hogan, R. Edward
Hopp, Jennifer L.
Naritoku, Dean
Plueger, Madona
Schevon, Catherine
Smith, Gigi
Valencia, Ignacio
Gaillard, William Davis
TI Highlights From AES2020, a Virtual American Epilepsy Society Experience
SO EPILEPSY CURRENTS
LA English
DT Article
DE annual meeting; epilepsy; treatment; education; cognition
ID SURGERY; STIMULATION; NETWORKS; PATTERNS; CHILDREN; SEIZURES; OUTCOMES; ACADEMY; CORTEX; MEMORY
AB Due to COVID-19 a live, in-person meeting was not possible for the American Epilepsy Society in 2020. An alternative, virtual event, the AES2020, was held instead. AES2020 was a great success with 4679 attendees from 70 countries. The educational content was outstanding and spanned the causes, treatments, and outcomes from epileptic encephalopathy to the iatrogenicity of epilepsy interventions to neurocognitive disabilities to the approach to neocortical epilepsies. New gene therapy approaches such as antisense oligonucleotide treatment for Dravet syndrome were introduced and neuromodulation devices were discussed. There were many other topics discussed in special interest groups and investigators' workshops. A highlight was having a Nobel prize winner speak about memory processing. Human intracranial electrophysiology contributes insights into memory processing and complements animal work. In a special COVID symposium, the impact of COVID on patients with epilepsy was reviewed. Telehealth has been expanded rapidly and may be well suited for some parts of epilepsy care. In summary, the epilepsy community was alive and engaged despite being limited to a virtual platform.
C1 [Jobst, Barbara C.] Dartmouth Hitchcock Med Ctr, 1 Med Ctr Dr, Lebanon, NH 03756

USA.
[Conner, Kelly R.] Wake Forest Sch Med, Winston Salem, NC USA.
[Coulter, Douglas] Childrens Hosp Philadelphia, Philadelphia, PA 19104 USA.
[Fried, Itzhak] Univ Calif Los Angeles, Los Angeles, CA USA.
[Guilfoyle, Shanna] Cincinnati Childrens Hosp Med Ctr, Cincinnati, OH 45229 USA.
[Hirsch, Lawrence J.] Yale Univ, Sch Med, New Haven, CT USA.
[Hogan, R. Edward] Washington Univ, St Louis, MO 63110 USA.
[Hopp, Jennifer L.] Univ Maryland, Med Ctr, Baltimore, MD 21201 USA.
[Naritoku, Dean] Univ S Alabama, Mobile, AL USA.
[Plueger, Madona] Barrow Neurol Inst, Phoenix, AZ 85013 USA.
[Schevon, Catherine] Columbia Univ, New York, NY USA.
[Smith, Gigi] MUSC, Charleston, SC USA.
[Valencia, Ignacio] St Christopher Hosp Children, Philadelphia, PA USA.
[Gaillard, William Davis] Childrens Natl Med Ctr, Washington, DC 20010 USA.
C3 Dartmouth College; Wake Forest University; University of Pennsylvania; Childrens Hospital of Philadelphia; University of California System; University of California Los Angeles; Cincinnati Children's Hospital Medical Center; Yale University; Washington University (WUSTL); University System of Maryland; University of Maryland Baltimore; University of South Alabama; Barrow Neurological Institute; Columbia University; Medical University of South Carolina; Children's National Health System
RP Jobst, BC (通讯作者), Dartmouth Hitchcock Med Ctr, 1 Med Ctr Dr, Lebanon, NH 03756 USA.
EM barbara.c.jobst@hitchcock.org
TC 0
Z9 0
PD JUL
PY 2021
VL 21
IS 4
BP 303
EP 310
AR 15357597211018219
DI 10.1177/15357597211018219
EA MAY 2021
WC Clinical Neurology
ER

PT J
AU Jain, S
Potschka, H
Chandra, PP
Tripathi, M
Vohora, D
AF Jain, Shreshta
Potschka, Heidrun

Chandra, P. Prarthana
Tripathi, Manjari
Vohora, Divya
TI Management of COVID-19 in patients with seizures: Mechanisms of action of potential COVID-19 drug treatments and consideration for potential drug-drug interactions with anti-seizure medications
SO EPILEPSY RESEARCH
LA English
DT Article
DE COVID-19 candidate drugs; Seizure; Drug-drug interaction; Antiviral drugs; Immunomodulatory or anti-inflammatory; drug; NSAIDs
ID NONSTEROIDAL ANTIINFLAMMATORY DRUGS; CHLOROQUINE THERAPY; ANTIEPILEPTIC DRUGS; VALPROIC ACID; CORONAVIRUS; IVERMECTIN; INHIBITOR; INFECTION; CYTOCHROME-P450; VIRUS
AB In regard to the global pandemic of COVID-19, it seems that persons with epilepsy (PWE) are not more vulnerable to get infected by SARS-CoV-2, nor are they more susceptible to a critical course of the disease. However, management of acute seizures in patients with COVID-19 as well as management of PWE and COVID19 needs to consider potential drug-drug interactions between antiseizure drugs and candidate drugs currently assessed as therapeutic options for COVID-19. Repurposing of several licensed and investigational drugs is discussed for therapeutic management of COVID-19. While for none of these approaches, efficacy and tolerability has been confirmed yet in sufficiently powered and controlled clinical studies, testing is ongoing with multiple clinical trials worldwide. Here, we have summarized the possible mechanisms of action of drugs currently considered as potential therapeutic options for COVID-19 management along with possible and confirmed drugdrug interactions that should be considered for a combination of antiseizure drugs and COVID-19 candidate drugs. Our review suggests that potential drug-drug interactions should be taken into account with drugs such as chloroquine/hydroxychloroquine and lopinavir/ritonavir while remdesivir and tocilizumab may be less prone to clinically relevant interactions with ASMs.
C1 [Jain, Shreshta; Vohora, Divya] Jamia Hamdard, Dept Pharmacol, Sch Pharmaceut Educ & Res, New Delhi 110062, India.
[Potschka, Heidrun] Ludwig Maximilians Univ LMU, Inst Pharmacol Toxicol & Pharm, Koniginstr 16, D-80539 Munich, Germany.
[Chandra, P. Prarthana] Hamdard Inst Med Sci & Res, New Delhi, India.
[Tripathi, Manjari] All India Inst Med Sci, Dept Neurol, New Delhi, India.
C3 Jamia Hamdard University; League of European Research Universities - LERU; University of Munich; Jamia Hamdard University; All India Institute of Medical Sciences (AIIMS) New Delhi
RP Vohora, D (通讯作者), Jamia Hamdard, Dept Pharmacol, Sch Pharmaceut Educ & Res, New Delhi 110062, India.
EM dvohora@jamiahamdard.ac.in
TC 2
Z9 2
PD AUG
PY 2021
VL 174

AR 106675
DI 10.1016/j.eplepsyres.2021.106675
EA MAY 2021
WC Clinical Neurology
ER

PT J
AU Kuroda, N
AF Kuroda, Naoto
TI Estimated effect of COVID-19 vaccine in people with epilepsy
SO EPILEPSY & BEHAVIOR
LA English
DT Letter
DE effect; COVID-19; vaccine; epilepsy
C1 [Kuroda, Naoto] Wayne State Univ, Dept Pediat, 3901 Beaubien St, Detroit, MI 48201 USA.
   [Kuroda, Naoto] Tohoku Univ, Dept Epileptol, Grad Sch Med, Sendai, Miyagi, Japan.
C3 Wayne State University; Tohoku University
RP Kuroda, N (通讯作者), Wayne State Univ, Dept Pediat, 3901 Beaubien St, Detroit, MI 48201 USA.
EM naoto.kuroda@wayne.edu
TC 0
Z9 0
PD AUG
PY 2021
VL 121
AR 108072
DI 10.1016/j.yebeh.2021.108072
EA MAY 2021
WC Behavioral Sciences; Clinical Neurology; Psychiatry
ER

PT J
AU Asadi-Pooya, AA
   Sahraian, A
   Badv, RS
   Sahraian, MA
AF Asadi-Pooya, Ali A.
   Sahraian, Ali
   Badv, Reza Shervin
   Sahraian, Mohamad Ali
TI Physicians' opinions on the necessity of COVID-19 vaccination in
   patients with epilepsy
SO EPILEPTIC DISORDERS
LA English
DT Article
DE coronavirus; COVID; epilepsy; seizure; vaccine

ID UNITED-STATES; PEOPLE
AB Objective. The aim of the current study was to investigate the opinions of neurologists and psychiatrists in Iran on the necessity of COVID-19 vaccination in patients with epilepsy (PWE). These data can help policy makers understand the concerns of these healthcare professionals.

Methods. This was a survey study. On September 1st, 2020 we sent a questionnaire (using Google-forms) to all neurologists and psychiatrists in Iran via WhatsApp. The survey included three general questions (age, sex, and discipline) and six COVID-specific questions.

Results. In total, 202 physicians participated in this study (116 neurologists and 86 psychiatrists). Of the participants, 27% believed that PWE are at increased risk of contracting COVID-19. The majority (74%) of the participants would confidently recommend COVID-19 vaccine to their patients. However, only 49% of the physicians would recommend such a vaccine to all patients; others would consider it in special populations only. The overwhelming majority (91%) of the participants would recommend COVID-19 vaccine only when a reliable vaccine becomes available. Many physicians would trust a vaccine that is approved by the World Health Organization (WHO) (46%) or a vaccine that is approved by the Food and Drug Administration (FDA-USA) (34%).

Significance. Physicians have concerns on the issue of the necessity of COVID-19 vaccine in PWE. The most important concern is the reliability of a vaccine and in this regard, two health agencies, the WHO and the FDA, are the most trusted organizations to approve a vaccine against COVID-19.
C1 [Asadi-Pooya, Ali A.; Sahraian, Ali] Shiraz Univ Med Sci, Epilepsy Res Ctr, Shiraz, Iran.

[Asadi-Pooya, Ali A.] Thomas Jefferson Univ, Dept Neurol, Jefferson Comprehens Epilepsy Ctr, Philadelphia, PA 19107 USA.

[Badv, Reza Shervin] Univ Tehran Med Sci, Childrens Med Ctr, Pediat Ctr Excellence, Tehran, Iran.

[Sahraian, Mohamad Ali] Univ Tehran Med Sci, Multiple Sclerosis Res Ctr, Tehran, Iran.

C3 Shiraz University of Medical Science; Jefferson University; Tehran University of Medical Sciences; Tehran University of Medical Sciences

RP Asadi-Pooya, AA (通讯作者), Shiraz Univ Med Sci, Epilepsy Res Ctr, Shiraz, Iran.

EM aliasadipooya@yahoo.com
TC 1

Z9 1

PD JUN

PY 2021

VL 23

IS 3

BP 485

EP 489

DI 10.1684/epd.2021.1282

WC Clinical Neurology

ER

PT J

AU Martinez, AG
   Planchuelo-Gomez, A
   Alba, V
   Martinez-Dubarbie, F
   Vivancos, J
   De Toledo, M
AF Gonzalez Martinez, A.
   Planchuelo-Gomez, A.
   Alba, V.
   Martinez-Dubarbie, F.
   Vivancos, J.
   De Toledo, M.
TI Longitudinal reduction of quality of life in patients with epilepsy and
   no seizure increase during the COVID-19 pandemic
SO EUROPEAN JOURNAL OF NEUROLOGY
LA English
DT Meeting Abstract
DE COVID-19; quality of life; Epilepsy; Seizure
C1 [Planchuelo-Gomez, A.] Univ Valladolid, Imaging Proc Lab, Valladolid, Spain.
   [Martinez-Dubarbie, F.] Hosp Marques Valdecilla, Dept Neurol, Santander, Spain.
   [Vivancos, J.] Hosp Univ La Princesa, Dept Neurol, Madrid, Spain.
   [De Toledo, M.] Dept Neurol, Madrid, Spain.
C3 Universidad de Valladolid; Hospital Universitario Marques de Valdecilla
   (HUMV); Hospital de La Princesa
TC 0
Z9 0
PD JUN
PY 2021
VL 28
SU 1
MA OPR-064
BP 104
EP 104
WC Clinical Neurology; Neurosciences
ER

PT J
AU Requiao, L
   Moreno, B
   Paiva, A
AF Requiao, L.
   Moreno, B.
   Paiva, A.
TI The impact of telemedicine for patients with epilepsy during the
   COVID-19 pandemic
SO EUROPEAN JOURNAL OF NEUROLOGY
LA English

DT Meeting Abstract
DE telemedicine; epilepsy; COVID-19
TC 0
Z9 0
PD JUN
PY 2021
VL 28
SU 1
MA EPO-525
BP 769
EP 769
WC Clinical Neurology; Neurosciences
ER

PT J
AU Volkers, N
Wiebe, S
Asadi-Pooya, AA
Balagura, G
Gomez-Iglesias, P
Guekht, A
Hall, J
Ikeda, A
Jette, N
Kishk, NA
Murphy, P
Perucca, E
Perez-Poveda, JC
Sanya, EO
Trinka, E
Zhou, D
Cross, JH
AF Volkers, Nancy
Wiebe, Samuel
Asadi-Pooya, Ali Akbar
Balagura, Ganna
Gomez-Iglesias, Patricia
Guekht, Alla
Hall, Julie
Ikeda, Akio
Jette, Nathalie
Kishk, Nirmeen A.
Murphy, Peter
Perucca, Emilio
Perez-Poveda, Juan Carlos
Sanya, Emmanuel O.
Trinka, Eugen

Zhou, Dong
Cross, J. Helen
TI The initial impact of the SARS-CoV-2 pandemic on epilepsy research
SO EPILEPSIA OPEN
LA English
DT Review
DE COVID-19; epilepsy; care; epilepsy research; pandemic; virtual working
ID CARE
AB The COVID-19 pandemic has changed the face of many practices throughout the world. Through necessity to minimize spread and provide clinical care to those with severe disease, focus has been on limiting face-to-face contact. Research in many areas has been put on hold. We sought to determine the impact of the COVID-19 pandemic on epilepsy research from international basic science and clinical researchers. Responses to five questions were solicited through a convenience sample by direct email and through postings on the ILAE social media accounts and an ILAE online platform (utilizing Slack). Information was collected from 15 respondents in 11 countries by email or via Zoom interviews between May 19, 2020, and June 4, 2020. Several themes emerged including a move to virtual working, project delays with laboratory work halted and clinical work reduced, funding concerns, a worry about false data with regard to COVID research and concern about research time lost. However, a number of positive outcomes were highlighted, not least the efficiency of online working and other adaptations that could be sustained in the future.
C1 [Volkers, Nancy; Hall, Julie] Int League Epilepsy, Flower Mound, TX USA.
[Wiebe, Samuel] Univ Calgary, Cumming Sch Med, Dept Clin Neurosci, Calgary, AB, Canada.
[Wiebe, Samuel] Univ Calgary, Cumming Sch Med, Dept Community Hlth Sci, Calgary, AB, Canada.
[Asadi-Pooya, Ali Akbar] Shiraz Univ Med Sci, Epilepsy Res Ctr, Shiraz, Iran.
[Asadi-Pooya, Ali Akbar] Thomas Jefferson Univ, Dept Neurol, Jefferson Comprehens Epilepsy Ctr, Philadelphia, PA 19107 USA.
[Balagura, Ganna] IRCCS G Gaslini Inst, Pediat Neurol & Muscular Dis Unit, Genoa, Italy.
[Balagura, Ganna] Univ Genoa, Dept Neurosci Rehabil Ophthalmol Genet Maternal &, Genoa, Italy.
[Gomez-Iglesias, Patricia] Univ Complutense Madrid, Hosp Clin San Carlos, Hlth Res Inst San Carlos IdISCC, Dept Neurol,Epilepsy Unit, Madrid, Spain.
[Guekht, Alla] Moscow Res & Clin Ctr Neuropsychiat, Moscow, Russia.
[Guekht, Alla] Russian Natl Res Med Univ, Dept Neurol Neurosurg & Med Genet, Moscow, Russia.
[Ikeda, Akio] Kyoto Univ, Grad Sch Med, Dept Epilepsy Movement Disorders & Physiol, Kyoto, Japan.
[Jette, Nathalie] Icahn Sch Med Mt Sinai, Dept Neurol, Div Hlth Outcomes & Knowledge Translat Res, New York, NY 10029 USA.
[Kishk, Nirmeen A.] Cairo Univ, Fac Med, Neurol Dept, Cairo, Egypt.
[Murphy, Peter] Epilepsy Ireland, Dublin, Ireland.
[Perucca, Emilio] Univ Pavia, Dept Internal Med & Therapeut, Div Clin & Expt Pharmacol, Pavia, Italy.

[Perucca, Emilio] IRCCS Mondino Fdn, Pavia, Italy.
[Perucca, Emilio; Trinka, Eugen; Cross, J. Helen] ERN EpiCARE, Zagreb, Croatia.
[Perez-Poveda, Juan Carlos] Xavierian Univ, Fac Med, Neurosci Dept, Bogota, Colombia.
[Perez-Poveda, Juan Carlos] Hosp Univ San Ignacio, Bogota, Colombia.
[Sanya, Emmanuel O.] Univ Ilorin, Teaching Hosp, Neurol Div, Med Dept, Ilorin, Nigeria.
[Trinka, Eugen] Paracelsus Med Univ, Christian Doppler Univ Hosp, Ctr Cognit Neurosci, Dept Neurol, Salzburg, Austria.
[Trinka, Eugen] Private Univ Hlth Sci Med Informat & Technol, UMIT, Inst Publ Hlth Med Decis Making & HTA, Hall In Tirol, Austria.
[Zhou, Dong] Sichuan Univ, West China Hosp, Dept Neurol, Chengdu, Peoples R China.
[Cross, J. Helen] UCL NIHR BRC Great Ormond St Inst Child Hlth, Programme Dev Neurosci, London, England.
[Cross, J. Helen] Young Epilepsy Lingfield, London, England.
[Cross, J. Helen] Great Ormond St Hosp Sick Children, London, England.
C3 University of Calgary; University of Calgary; Shiraz University of Medical Science; Jefferson University; University of Genoa; IRCCS Istituto Giannina Gaslini; University of Genoa; Complutense University of Madrid; Hospital Clinico San Carlos; Pirogov Russian National Research Medical University; Kyoto University; Icahn School of Medicine at Mount Sinai; Egyptian Knowledge Bank (EKB); Cairo University; University of Pavia; IRCCS Fondazione Casimiro Mondino; Hospital Universitario San Ignacio; University of Ilorin; Paracelsus Private Medical University; Sichuan University; University of London; University College London; Great Ormond Street Hospital for Children NHS Foundation Trust
RP Cross, JH (通讯作者), UCL Great Ormond St Inst Child Hlth, Prince Waless Chair Childhood Epilepsy, 30 Guilford St, London WC1N 1EH, England.
EM h.cross@ucl.ac.uk
TC 1
Z9 1
PD JUN
PY 2021
VL 6
IS 2
BP 255
EP 265
DI 10.1002/epi4.12471
WC Clinical Neurology; Neurosciences
ER

PT J
AU Fitzgerald, MP
Kaufman, MC
Massey, SL
Fridinger, S

Prelack, M
Ellis, C
Ortiz-Gonzalez, X
Fried, LE
DiGiovine, MP
Melamed, S
Malcolm, M
Banwell, B
Stephenson, D
Witzman, SM
Gonzalez, A
Dlugos, D
Kessler, SK
Goldberg, EM
Abend, NS
Helbig, I
AF Fitzgerald, Mark P.
Kaufman, Michael C.
Massey, Shavonne L.
Fridinger, Sara
Prelack, Marisa
Ellis, Colin
Ortiz-Gonzalez, Xilma
Fried, Lawrence E.
DiGiovine, Marissa P.
Melamed, Susan
Malcolm, Marissa
Banwell, Brenda
Stephenson, Donna
Witzman, Stephanie M.
Gonzalez, Alexander
Dlugos, Dennis
Kessler, Sudha Kilaru
Goldberg, Ethan M.
Abend, Nicholas S.
Helbig, Ingo
CA CHOP Pediat Epil
TI Assessing seizure burden in pediatric epilepsy using an electronic medical record-based tool through a common data element approach
SO EPILEPSIA
LA English
DT Article
DE common data elements; epilepsy outcomes; health care disparities; seizure; telemedicine
ID HEALTH RECORD; DISPARITIES; COVID-19
AB Objective Improvement in epilepsy care requires standardized methods to assess disease severity. We report the results of implementing common data elements (CDEs)

to document epilepsy history data in the electronic medical record (EMR) after 12 months of clinical use in outpatient encounters.

Methods Data regarding seizure frequency were collected during routine clinical encounters using a CDE-based form within our EMR. We extracted CDE data from the EMR and developed measurements for seizure severity and seizure improvement scores. Seizure burden and improvement was evaluated by patient demographic and encounter variables for in-person and telemedicine encounters.

Results We assessed a total of 1696 encounters in 1038 individuals with childhood epilepsies between September 6, 2019 and September 11, 2020 contributed by 32 distinct providers. Childhood absence epilepsy (n = 121), Lennox-Gastaut syndrome (n = 86), and Dravet syndrome (n = 42) were the most common epilepsy syndromes. Overall, 43% (737/1696) of individuals had at least monthly seizures, 17% (296/1696) had a least daily seizures, and 18% (311/1696) were seizure-free for >12 months. Quantification of absolute seizure burden and changes in seizure burden over time differed between epilepsy syndromes, including high and persistent seizure burden in patients with Lennox-Gastaut syndrome. Individuals seen via telemedicine or in-person encounters had comparable seizure frequencies. Individuals identifying as Hispanic/Latino, particularly from postal codes with lower median household incomes, were more likely to have ongoing seizures that worsened over time.

Significance Standardized documentation of clinical data in childhood epilepsies through CDE can be implemented in routine clinical care at scale and enables assessment of disease burden, including characterization of seizure burden over time. Our data provide insights into heterogeneous patterns of seizure control in common pediatric epilepsy syndromes and will inform future initiatives focusing on patient-centered outcomes in childhood epilepsies, including the impact of telemedicine and health care disparities.
C1 [Fitzgerald, Mark P.; Kaufman, Michael C.; Massey, Shavonne L.; Fridinger, Sara; Prelack, Marisa; Ellis, Colin; Ortiz-Gonzalez, Xilma; Fried, Lawrence E.; DiGiovine, Marissa P.; Melamed, Susan; Malcolm, Marissa; Banwell, Brenda; Stephenson, Donna; Witzman, Stephanie M.; Dlugos, Dennis; Kessler, Sudha Kilaru; Goldberg, Ethan M.; Abend, Nicholas S.; Helbig, Ingo] Childrens Hosp Philadelphia, Div Neurol, Philadelphia, PA 19104 USA.

[Fitzgerald, Mark P.; Kaufman, Michael C.; Massey, Shavonne L.; Ellis, Colin; Ortiz-Gonzalez, Xilma; Goldberg, Ethan M.; Helbig, Ingo] Childrens Hosp Philadelphia, Epilepsy NeuroGenet Initiat ENGIN, Philadelphia, PA 19104 USA.

[Fitzgerald, Mark P.; Massey, Shavonne L.; Fridinger, Sara; Prelack, Marisa; Ellis, Colin; Ortiz-Gonzalez, Xilma; Fried, Lawrence E.; DiGiovine, Marissa P.; Banwell, Brenda; Stephenson, Donna; Dlugos, Dennis; Kessler, Sudha Kilaru; Goldberg, Ethan M.; Abend, Nicholas S.; Helbig, Ingo; CHOP Pediat Epil] Univ Penn, Dept Neurol, Perelman Sch Med, Philadelphia, PA 19104 USA.

[Kaufman, Michael C.; Gonzalez, Alexander; Helbig, Ingo] Childrens Hosp Philadelphia, Dept Biomed & Hlth Informat DBHi, Philadelphia, PA 19104 USA.

C3 University of Pennsylvania; Childrens Hospital of Philadelphia;
University of Pennsylvania; Childrens Hospital of Philadelphia;
University of Pennsylvania; University of Pennsylvania; Childrens Hospital of Philadelphia

RP Helbig, I (通讯作者), Univ Penn, Perelman Sch Med, Childrens Hosp Philadelphia, Div

Neurol, Philadelphia, PA 19104 USA.
EM helbigi@email.chop.edu
TC 2
Z9 2
PD JUL
PY 2021
VL 62
IS 7
BP 1617
EP 1628
DI 10.1111/epi.16934
EA JUN 2021
WC Clinical Neurology
ER

PT J
AU Guilhoto, LM
   Mosini, AC
   Susemihl, MA
   Pinto, LF
AF Guilhoto, Laura Maria
   Mosini, Amanda Cristina
   Susemihl, Maria Alice
   Pinto, Lecio Figueira
TI COVID-19 and epilepsy: How are people with epilepsy in Brazil?
SO EPILEPSY & BEHAVIOR
LA English
DT Article
DE Epilepsy; Survey; COVID-19; Quality of life
AB Purpose: During COVID-19 pandemic the global population is facing an important psychosocial distress. The aim of this study was to evaluate how people with epilepsy (PWE) in Brazil is dealing with the pandemic, in relation to seizure frequency, access to antiseizure medicines (ASM), medical follow-up, and well-being. Methods: An online questionnaire survey among PWE (group 1) and caregivers (group 2) was applied in the social networks of the Brazilian Association of Epilepsy, the official Brazilian chapter of the International Bureau for Epilepsy. The questionnaire was composed of 46 generic questions in four areas, namely, demographics and baseline clinical data as well as epilepsy and quality-of-life impact by COVID19 pandemic based on the domains of the abbreviated World Health Organization Quality of Life (WHOQOL-BREF) instrument. Results: The questionnaire was answered by 464 participants including 380 (81.9%) PWE (78.7% female; age 34.3 yrs.; +/- 9.76) and 84 (18.1%) caregivers (patients' age 14.1 yrs.; +/- 10.30). During the COVID-19 pandemic, 36.8% of PWE and 36.4% of caregivers reported difficulties in accessing the epilepsy healthcare provider, and visits occurred normally only in 29.7% of PWE and in 34.5% of the caregiver acute accent s group. Telehealth was not provided for 66.6% of group 1 and for 58.5% of group 2. Lack of availability of ASM was reported by 21.9% of PWE and 28.0% of caregivers in public dispensing units and by 19.2% and 17.8%, respectively, in private pharmacies. Increase

in seizures during pandemic was mentioned by 26.3% and 27.9% of groups 1 and 2, respectively. Patients who had increase in seizure frequency had more frequently reported problems with treatment and in quality-of-life concepts. Fear of having a more severe COVID-19 presentation because of epilepsy was reported by 74.5% of PWE and by 89.8% of caregivers. Dissatisfaction with current health status was reported by 36.7% and 38.1% in groups 1 and 2, respectively, and that the support from others has decreased (56.1% and 66.1%, in groups 1 and 2) during the pandemic. The factors with higher Odds Ratio of increase in seizure frequency during pandemic were age >41 yrs., treatment in public healthcare system, drug-resistant epilepsy, adversities in getting ASM in public dispensing units, difficulties with prescription renewals, current financial problems and belief that epilepsy or ASM are risk factors for contracting COVID-19. Conclusion: During COVID-19 pandemic in Brazil, PWE and caregivers reported increase in seizures in one-fourth of the patients and several difficulties, namely problems in accessing the healthcare system including ASM dispensation, telehealth, and fear of having a more severe COVID-19 because of epilepsy. There were also physical, psychological, and social concerns which affected quality-of-life-related aspects in this population. These facts may increase treatment gap in epilepsy in Brazil as well in other developing countries. (c) 2021 Elsevier Inc. All rights reserved.
C1 [Guilhoto, Laura Maria; Mosini, Amanda Cristina; Susemihl, Maria Alice; Pinto, Lecio Figueira] Assoc Brasileira Epilepsia, Sao Paulo, Brazil.
[Mosini, Amanda Cristina] Univ Fed Sao Paulo EPM Unifesp, Dept Fisiol, Escola Paulista Med, Sao Paulo, Brazil.
[Guilhoto, Laura Maria; Mosini, Amanda Cristina] EPM Unifesp, Programa Posgrad Neurociencias & Neurol, Sao Paulo, Brazil.
[Guilhoto, Laura Maria] Univ Sao Paulo, Hosp Univ, Sao Paulo, Brazil.
[Pinto, Lecio Figueira] HC FMUSP, Div Clin Neurol, Sao Paulo, Brazil.
C3 Universidade Federal de Sao Paulo (UNIFESP); Universidade Federal de Sao Paulo (UNIFESP); Universidade de Sao Paulo; Universidade de Sao Paulo
RP Guilhoto, LM (通讯作者), Univ Fed Sao Paulo, Rua Pedro de Toledo 650, Andar Terreo, Sala 11, Sao Paulo, SP, Brazil.
EM lauragui@gmail.com
TC 1
Z9 1
PD SEP
PY 2021
VL 122
AR 108115
DI 10.1016/j.yebeh.2021.108115
EA JUN 2021
WC Behavioral Sciences; Clinical Neurology; Psychiatry
ER

PT J
AU Aladdin, Y
Shirah, B
AF Aladdin, Yasser
Shirah, Bader

TI New-onset refractory status epilepticus following the ChAdOx1 nCoV-19 vaccine
SO JOURNAL OF NEUROIMMUNOLOGY
LA English
DT Article
DE nCoV-19; COVID-19; Vaccine; Seizure; Status epilepticus
AB Coronavirus is a novel human pathogen causing fulminant respiratory syndrome (COVID-19). Developing an effective and reliable vaccine was emergently pursued to control the dramatic spread of the global pandemic. The standard stages for vaccine development were unprecedentedly accelerated over a few months. We report a case of new-onset refractory status epilepticus (NORSE) after receiving the first dose of the ChAdOx1 nCoV-19 vaccine. We attribute the occurrence of NORSE to the vaccine due to the temporal relationship and the lack of risk factors for epilepsy in the patient. This report adds to the literature a possible rare side effect of a COVID-19 vaccine and contributes to the extremely limited literature on potential neurological side effects of viral vector vaccines. Healthcare providers should be aware of the possibility of post-vaccination epilepsy. The patient had recurrent seizures that were refractory to conventional antiepileptic drug therapy with a dramatic response to immunotherapy with pulse steroids and plasmapheresis. This likely reflects an underlying autoimmune mechanism in the genesis of post-vaccination generalized seizures without fever. Further research is needed to probe and study the exact mechanism at a more molecular level.
C1 [Aladdin, Yasser] King Abdul Aziz Med City, Jeddah, Saudi Arabia.
[Aladdin, Yasser; Shirah, Bader] King Saud Bin Abdulaziz Univ Hlth Sci, Jeddah, Saudi Arabia.
[Aladdin, Yasser; Shirah, Bader] King Abdullah Int Med Res Ctr, Jeddah, Saudi Arabia.
C3 King Saud Bin Abdulaziz University for Health Sciences
RP Aladdin, Y (通讯作者), King Saud Bin Abdulaziz Univ Hlth Sci, King Abdulaziz Med City, POB 12723, Jeddah 21483, Saudi Arabia.
EM Yasseraladdin@yahoo.com
TC 3
Z9 3
PD AUG 15
PY 2021
VL 357
AR 577629
DI 10.1016/j.jneuroim.2021.577629
EA JUN 2021
WC Immunology; Neurosciences
ER

PT J
AU von Wrede, R
Pukropski, J
Moskau-Hartmann, S
Surges, R
Baumgartner, T

AF von Wrede, Randi
Pukropski, Jan
Moskau-Hartmann, Susanna
Surges, Rainer
Baumgartner, Tobias
TI COVID-19 vaccination in patients with epilepsy: First experiences in a German tertiary epilepsy center
SO EPILEPSY & BEHAVIOR
LA English
DT Article
DE Epilepsy; COVID-19; SARS-CoV-2; Vaccine; Adverse effect
AB Introduction: Due to the high demand for information regarding COVID-19 vaccination in people with epilepsy (PWE), we assessed the symptoms and seizure control of PWE following their COVID-19 vaccination. Methods: All adult patients who were treated at our center were asked to report on their vaccination status and, if vaccinated, about their experiences following their first COVID-19 vaccination with regard to adverse effects and seizure control. Results: Fifty-four PWE have already received their first vaccination against COVID-19 (27 female, 20% seizure free, 96<% on antiseizure medication) and were included in the study. Two-thirds tolerated the vaccines generally either very well or well. Thirty-three percent reported general vaccination adverse effects. The most frequently reported general adverse effects were, in descending order, headache, fatigue and fever, and shivering. With regard to epilepsy-related adverse effects, one patient reported increased seizure frequency one day after the first COVID-19 vaccination was administered, and one reported the occurrence of a new seizure type. None of the patients reported a status epilepticus or aggravation of preexisting adverse effects. Conclusions: Our data suggest that vaccination against COVID-19 appears to be well tolerated in PWE, supporting the recommendation of vaccination to PWE. (c) 2021 Elsevier Inc. All rights reserved.
C1 [von Wrede, Randi; Pukropski, Jan; Moskau-Hartmann, Susanna; Surges, Rainer; Baumgartner, Tobias] Univ Hosp Bonn, Dept Epileptol, Venusberg Campus 1, D-53127 Bonn, Germany.
C3 University of Bonn
RP von Wrede, R (通讯作者), Univ Hosp Bonn, Dept Epileptol, Venusberg Campus 1, D-53127 Bonn, Germany.
EM randi.von.wrede@ukbonn.de
TC 5
Z9 5
PD SEP
PY 2021
VL 122
AR 108160
DI 10.1016/j.yebeh.2021.108160
EA JUN 2021
WC Behavioral Sciences; Clinical Neurology; Psychiatry
ER

PT J

AU Dewanjee, S
Vallamkondu, J
Kalra, RS
Puvvada, N
Kandimalla, R
Reddy, PH
AF Dewanjee, Saikat
Vallamkondu, Jayalakshmi
Kalra, Rajkumar Singh
Puvvada, Nagaprasad
Kandimalla, Ramesh
Reddy, P. Hemachandra
TI Emerging COVID-19 Neurological Manifestations: Present Outlook and Potential Neurological Challenges in COVID-19 Pandemic
SO MOLECULAR NEUROBIOLOGY
LA English
DT Article; Early Access
DE COVID-19; SARS-CoV-2; CNS; PNS; Neuroinvasion; COVID-19; Neurological complication
ID GUILLAIN-BARRE-SYNDROME; MYASTHENIA-GRAVIS; CLINICAL CHARACTERISTICS; MULTIPLE-SCLEROSIS; ALZHEIMERS-DISEASE; COMPLICATIONS; SARS-COV-2; ACE2; INFECTION; SEVERITY
AB The unremitting coronavirus disease 2019 (COVID-19) pandemic caused by the novel severe acute respiratory syndrome coronavirus 2 (SARS-CoV-2) marked a year-long phase of public health adversaries and has severely compromised healthcare globally. Early evidence of COVID-19 noted its impact on the pulmonary and cardiovascular functions, while multiple studies in recent time shed light on its substantial neurological complications, though a comprehensive understanding of the cause(s), the mechanism(s), and their neuropathological outcomes is scarce. In the present review, we conferred evidence of neurological complications in COVID-19 patients and shed light on the SARS-CoV-2 infection routes including the hematogenous, direct/neuronal, lymphatic tissue or cerebrospinal fluid, or infiltration through infected immune cells, while the underlying mechanism of SARS-CoV-2 invasion to the central nervous system (CNS) was also discussed. In an up-to-date manner, we further reviewed the impact of COVID-19 in developing diverse neurologic manifestations associated with CNS, peripheral nervous system (PNS), skeletal muscle, and also pre-existing neurological diseases, including Alzheimer's disease, Parkinson's disease, multiple sclerosis, epilepsy, and myasthenia gravis. Furthermore, we discussed the involvement of key factors including age, sex, comorbidity, and disease severity in exacerbating the neurologic manifestations in COVID-19 patients. An outlook of present therapeutic strategies and state of existing challenges in COVID-19 management was also accessed. Conclusively, the present report provides a comprehensive review of COVID-19-related neurological complications and emphasizes the need for their early clinical management in the ongoing COVID-19 pandemic.
C1 [Dewanjee, Saikat] Jadavpur Univ, Dept Pharmaceut Technol, Adv Pharmacognosy Res Lab, Kolkata 700032, India.
[Vallamkondu, Jayalakshmi] Natl Inst Technol, Warangal 506004, Telangana, India.
[Kalra, Rajkumar Singh] Natl Inst Adv Ind Sci & Technol, AIST INDIA DAILAB, Higashi

1-1-1, Tsukuba, Ibaraki 3058565, Japan.
[Puvvada, Nagaprasad; Kandimalla, Ramesh] CSIR Indian Inst Technol, Appl Biol, Uppal Rd, Hyderabad 50000, Telangana, India.
[Kandimalla, Ramesh] Kakatiya Med Coll, Dept Biochem, Warangal 506007, Telangana, India.
[Reddy, P. Hemachandra] Texas Tech Univ, Hlth Sci Ctr, Lubbock, TX 79430 USA.
[Reddy, P. Hemachandra] Texas Tech Univ, Hlth Sci Ctr, Neurosci & Pharmacol, Lubbock, TX 79430 USA.
[Reddy, P. Hemachandra] Texas Tech Univ, Hlth Sci Ctr, Dept Neurol, Sch Med, Lubbock, TX 79430 USA.
[Reddy, P. Hemachandra] Texas Tech Univ, Hlth Sci Ctr, Publ Hlth Dept, Grad Sch Biomed Sci, Lubbock, TX 79430 USA.
[Reddy, P. Hemachandra] Texas Tech Univ, Hlth Sci Ctr, Dept Speech Language & Hearing Sci, Sch Hlth Profess, Lubbock, TX 79430 USA.
[Kalra, Rajkumar Singh] Okinawa Inst Sci & Technol Grad Univ, Immune Signal Unit, 1919-1 Tancha, Onna Son, Okinawa 9040495, Japan.
C3 Jadavpur University; National Institute of Technology (NIT System); National Institute of Technology Warangal; National Institute of Advanced Industrial Science & Technology (AIST); Kakatiya Medical College; Texas Tech University System; Texas Tech University; Texas Tech University Health Science Center; Texas Tech University System; Texas Tech University; Texas Tech University Health Science Center; Texas Tech University System; Texas Tech University; Texas Tech University Health Science Center; Texas Tech University System; Texas Tech University; Texas Tech University Health Science Center; Texas Tech University System; Texas Tech University; Texas Tech University Health Science Center; Okinawa Institute of Science & Technology Graduate University
RP Dewanjee, S (通讯作者), Jadavpur Univ, Dept Pharmaceut Technol, Adv Pharmacognosy Res Lab, Kolkata 700032, India.; Kandimalla, R (通讯作者), CSIR Indian Inst Technol, Appl Biol, Uppal Rd, Hyderabad 50000, Telangana, India.; Kandimalla, R (通讯作者), Kakatiya Med Coll, Dept Biochem, Warangal 506007, Telangana, India.; Reddy, PH (通讯作者), Texas Tech Univ, Hlth Sci Ctr, Lubbock, TX 79430 USA.; Reddy, PH (通讯作者), Texas Tech Univ, Hlth Sci Ctr, Neurosci & Pharmacol, Lubbock, TX 79430 USA.; Reddy, PH (通讯作者), Texas Tech Univ, Hlth Sci Ctr, Dept Neurol, Sch Med, Lubbock, TX 79430 USA.; Reddy, PH (通讯作者), Texas Tech Univ, Hlth Sci Ctr, Publ Hlth Dept, Grad Sch Biomed Sci, Lubbock, TX 79430 USA.; Reddy, PH (通讯作者), Texas Tech Univ, Hlth Sci Ctr, Dept Speech Language & Hearing Sci, Sch Hlth Profess, Lubbock, TX 79430 USA.
EM saikat.dewanjee@jadavpuruniversity.in; vlakshmij@gmail.com; rajskalra@hotmail.com; ramesh.kandimalla@gmail.com; hemachandra.reddy@ttuhsc.edu
TC 10
Z9 10
DI 10.1007/s12035-021-02450-6
EA JUN 2021
WC Neurosciences
ER

PT J
AU Gonzalez-Martinez, A
Planchuelo-Gomez, A
Campos, AV
Martinez-Dubarbie, F
Vivancos, J
De Toledo-Heras, M
AF Gonzalez-Martinez, Alicia
Planchuelo-Gomez, Alvaro
Vieira Campos, Alba
Martinez-Dubarbie, Francisco
Vivancos, Jose
De Toledo-Heras, Maria
TI Medium-term changes in patients with epilepsy during the COVID-19 pandemic
SO ACTA NEUROLOGICA SCANDINAVICA
LA English
DT Article
DE anxiety; COVID-19; epilepsy; pandemic; quality of life; sleep
ID QUALITY-OF-LIFE; SLEEP; DEPRESSION; QUARANTINE
AB Objectives The novel coronavirus disease (COVID-19) pandemic has led to social distancing measures and impaired medical care of chronic neurological diseases, including epilepsy, which may have adversely affected well-being and quality of life of patients with epilepsy (PWE). The objective of this study is to evaluate the impact of the COVID-19 pandemic in the levels of anxiety, depression, somnolence, and quality of life using validated scales in PWE in real-life clinical practice. Materials & Methods Self-administered scales of anxiety disorders (GAD-7), depression (NDDI-E), somnolence (Epworth Sleepiness Scale; ESS), and quality of life (QOLIE-31-P) in PWE treated in a Refractory Epilepsy Unit were longitudinally analyzed. Data were collected before the beginning (December 2019 - March 2020) and during the COVID-19 pandemic (September 2020-January 2021). Results 158 patients (85 from the first round and 73 from the second round) 45.0 +/- 17.3 years of age, 43.2% women, epilepsy duration 23.0 +/- 14.9 years, number of antiepileptic drugs 2.1 +/- 1.4, completed the survey. Significant longitudinal reduction of QOLIE-31-P (from 58.9 +/- 19.7 to 56.2 +/- 16.2, p = .035) and GAD-7 scores (from 8.8 +/- 6.2 to 8.3 +/- 5.9, corrected p = .024) was identified. No statistically significant longitudinal changes in the number of seizures (from 0.9 +/- 1.9 to 2.5 +/- 6.2, p = .125) or NDDI-E scores (from 12.3 +/- 4.3 to 13.4 +/- 4.4, p = .065) were found. Significant longitudinal increase of ESS (from 4.9 +/- 3.7 to 7.4 +/- 4.9, p = .001) was found. Conclusions During the COVID-19 pandemic, quality of life and anxiety levels were lower in PWE, and sleepiness levels were raised, without seizure change.
C1 [Gonzalez-Martinez, Alicia; Vivancos, Jose] Hosp Univ La Princesa, Neurol Dept, Madrid, Spain.
[Gonzalez-Martinez, Alicia; Vieira Campos, Alba; Vivancos, Jose; De Toledo-Heras, Maria] Inst Invest Sanitaria La Princesa, Madrid, Spain.
[Planchuelo-Gomez, Alvaro] Univ Valladolid, Imaging Proc Lab, Valladolid, Spain.
[Vieira Campos, Alba; De Toledo-Heras, Maria] Hosp Univ La Princesa, Neurol Dept,

Epilepsy Unit, Madrid, Spain.
[Martinez-Dubarbie, Francisco] Hosp Marques Valdecilla, Neurol Dept, Santander, Spain.
C3 Universidad de Valladolid; Hospital de La Princesa; Hospital
Universitario Marques de Valdecilla (HUMV)
RP Gonzalez-Martinez, A (通讯作者), Hosp Univ Princesa, Calle Diego Leon 62, Madrid 28006, Spain.
EM alicia.gonzalez.martinez@live.com
TC 0
Z9 0
PD OCT
PY 2021
VL 144
IS 4
BP 450
EP 459
DI 10.1111/ane.13481
EA JUN 2021
WC Clinical Neurology
ER

PT J
AU Aleboyeh, S
Appireddy, R
Winston, GP
Lomax, LB
Shukla, G
AF Aleboyeh, Sallya
Appireddy, Ramana
Winston, Gavin P.
Lomax, Lysa Boisse
Shukla, Garima
TI Virtual epilepsy clinics - A Canadian Comprehensive Epilepsy Center
experience pre-COVID and during the COVID-19 pandemic period
SO EPILEPSY RESEARCH
LA English
DT Article
DE Epilepsy; Virtual care; eVisits; COVID-19
ID TELEMEDICINE; CARE; ACCESS; NEUROLOGY
AB Objective: The objective of this study is to assess the role of prior experience with virtual care (through e-visits) in maintaining continuity in ambulatory epilepsy care during an unprecedented pandemic situation, comparing in person versus e-visit clinic uptake.
Methods: This is an observational study on virtual epilepsy care (through e-visits) over two years, during a pre-COVID period (14 months) continuing into the COVID-19 pandemic period (10 months). For a small initial section of patients seen during the study period a physician survey and a patient satisfaction survey were completed (n

= 53). Outcomes of eVisits were analyzed using descriptive statistics.
Results: Median numbers of epilepsy clinic visits conducted during the COVID-19 period (27.5 new and 113 follow up) remained similar to the median uptake during the pre-COVID period (28 new and 116 follow up). Prior experience with e-visits for epilepsy yielded smooth transition into the pandemic period, with several other advantages. The majority of eVisits were successful despite technical difficulties and major components of history and management were still easily implemented. Results from patient surveys supported that a significant amount of time and money were saved, which was in keeping with our health-economic analysis.
Conclusion: Our study is one of the first few reports of fully integrated virtual care in a comprehensive epilepsy clinic starting much before start of the COVID-19 pandemic. The results of our study support the feasibility of using virtual care to deliver specialized outpatient care in a comprehensive epilepsy center.
C1 [Aleboyeh, Sallya; Appireddy, Ramana; Winston, Gavin P.; Lomax, Lysa Boisse; Shukla, Garima] Queens Univ, Div Neurol, Dept Med, Kingston, ON, Canada.
C3 Queens University - Canada
RP Shukla, G (通讯作者), Queens Univ, Dept Med, 76 Stuart St, 02 704, Connell 7, Kingston, ON K7L 2V7, Canada.
EM garima.shukla@queensu.ca
TC 1
Z9 1
PD OCT
PY 2021
VL 176
AR 106689
DI 10.1016/j.eplepsyres.2021.106689
EA JUL 2021
WC Clinical Neurology
ER

PT J
AU Kuroda, N
Fujimoto, A
AF Kuroda, Naoto
Fujimoto, Ayataka
TI Considering temporality in causal relationship between seizure worsening and psychological stress in patients with epilepsy during the COVID-19 pandemic: A systematic review
SO EPILEPSY & BEHAVIOR
LA English
DT Review
DE COVID-19; Seizure control; Epilepsy; Causality; Temporality; Stress; Hill's criteria
AB Objective: To investigate whether published studies that identified a causal relationship between psychological stress and seizure worsening in patients with epilepsy during the coronavirus disease 2019 (COVID-19) pandemic considered the temporality of Hill's criteria. Method: A systematic review approach was used to

comprehensively search MEDLINE, CENTRAL, EMBASE, and ClinicalTrials.gov databases for relevant studies. Studies that reported an association between psychological stress and seizure worsening in patients with epilepsy during the COVID-19 pandemic were included accordingly. The quality of assessments in each study was evaluated and an assessment for considering temporality in the causal relationship between the two events in each study was carried out. Results: Seventeen studies were included in the analysis. Most (14/17) were cross-sectional studies and only four out of these 17 studies (23.5%) considered temporality in the causality. Further, these four studies did not consider temporality in the study design, they only described it as a limitation. Conclusion: We found that many articles reported a causal relationship between psychological stress and seizure worsening without considering temporality. As both researchers and readers, we need to consider temporality when interpreting the causal relationship between increased psychological stress and seizure worsening in patients with epilepsy during the COVID-19 pandemic. (c) 2021 Elsevier Inc. All rights reserved.
C1 [Kuroda, Naoto] Wayne State Univ, Dept Pediat, 3901 Beaubien St, Detroit, MI 48201 USA.
[Kuroda, Naoto] Tohoku Univ, Grad Sch Med, Dept Epileptol, Sendai, Miyagi, Japan.
[Fujimoto, Ayataka] Seirei Hamamatsu Gen Hosp, Comprehens Epilepsy Ctr, Hamamatsu, Shizuoka, Japan.
C3 Wayne State University; Tohoku University
RP Kuroda, N (通讯作者), Wayne State Univ, Dept Pediat, 3901 Beaubien St, Detroit, MI 48201 USA.
EM naoto.kuroda@wayne.edu
TC 4
Z9 4
PD SEP
PY 2021
VL 122
AR 108184
DI 10.1016/j.yebeh.2021.108184
EA JUL 2021
WC Behavioral Sciences; Clinical Neurology; Psychiatry
ER

PT J
AU Lunardi, MD
de Carvalho, RM
Carneiro, RAVD
Giacomini, F
Valente, KD
Lin, K
AF Lunardi, Mariana Dos Santos
de Carvalho, Rachel Marin
Veiga Domingues Carneiro, Raquel Alencastro
Giacomini, Felipe
Valente, Kette D.
Lin, Katia

TI Patients with epilepsy during the COVID-19 pandemic: Depressive symptoms and their association with healthcare access

SO EPILEPSY & BEHAVIOR
LA English
DT Article
DE COVID-19; Depression; Epilepsy; care; Antiepileptic drug

AB Background: The coronavirus disease 2019 (COVID-19) outbreak impacted the lives of worldwide people with epilepsy (PWE) in various aspects, particularly in those countries most significantly affected by this pandemic, such as Brazil. We aimed to investigate the prevalence of depressive symptoms in PWE and their correlation with epilepsy features and access to treatment. Methods: PWE were invited to answer a cross-sectional online-based survey to assess and rate depressive symptoms using the NDDI-E during the first year of the COVID-19 pandemic and its relation to multiple lifestyles epilepsy clinical aspects. Results: A total of 490 PWE were recruited. The prevalence of depressive symptoms during the COVID-19 pandemic was 35.3% (cutoff score > 15 on NDDI-E). The factors associated with higher NDDI-E scores were: female sex, increased seizure frequency, barriers to access to their treating physician and anti seizure medication, and unemployment. Regarding the pandemic impact on PWE healthcare, 29.2% reported restricted access to their medication, 46.1% barriers to access their physicians, 94.2% had their consultations canceled due to the pandemic, and 28.4% had seizure worsening in this period. Conclusion: The COVID-19 pandemic affected PWE access to the healthcare system. Depressive symptoms were more severe in patients with higher seizure frequency who had difficulties obtaining proper medical care. The COVID-19 pandemic may impact the healthcare and mental wellbeing of patients with chronic diseases such as epilepsy. Nevertheless, prospective studies on epilepsy and COVID-19 are still lacking. (c) 2021 Elsevier Inc. All rights reserved.

C1 [Lunardi, Mariana Dos Santos; Lin, Katia] Fed Univ Santa Catarina UFSC, Med Sci Grad Program, Florianopolis, SC, Brazil.

[de Carvalho, Rachel Marin] Coordinat Improvement Higher Educ Personnel CAPES, Sao Paulo, SP, Brazil.

[Veiga Domingues Carneiro, Raquel Alencastro; Giacomini, Felipe; Lin, Katia] Fed Univ Santa Catarina UFSC, Neurol Div, Florianopolis, SC, Brazil.

[Valente, Kette D.] Univ Sao Paulo, Psychiat Dept, Clin Hosp, Med Sch HCFMUSP, Sao Paulo, SP, Brazil.

C3 Universidade Federal de Santa Catarina (UFSC); Universidade Federal de Santa Catarina (UFSC); Universidade de Sao Paulo

RP Lunardi, MD (通讯作者), Univ Fed Santa Catarina, Hosp Univ HU, Dept Clin Med, 3 Andar,Rua Maria Flora Pausewang S-N,Cidade Univ, BR-88040900 Florianopolis, SC, Brazil.

EM marianalunardi1408@gmail.com

TC 2
Z9 2
PD SEP
PY 2021
VL 122
AR 108178
DI 10.1016/j.yebeh.2021.108178

EA JUL 2021
WC Behavioral Sciences; Clinical Neurology; Psychiatry
ER

PT J
AU Carneiro, RAVD
   Lunardi, MD
   Giacomini, FMU
   Rieger, DK
   Moreira, JD
   da Silva, LCR
   Sampaio, LPB
   Lin, K
AF Veiga Domingues Carneiro, Raquel Alencastro
   Lunardi, Mariana Dos Santos
   Uberna Giacomini, Felipe Matheus
   Rieger, Debora Kurrle
   Moreira, Julia Dubois
   Ribeiro da Silva, Leticia Carina
   Brito Sampaio, Leticia Pereira
   Lin, Katia
TI Challenges faced by people with epilepsy on ketogenic diet therapy and
   their caregivers during the COVID-19 pandemic in Brazil
SO EPILEPSY & BEHAVIOR
LA English
DT Article
DE Epilepsy; Ketogenic diet; COVID-19; Pandemic; SARS-CoV-2; Online survey
ID DRUG-RESISTANT EPILEPSY; TASK-FORCE; DEPRESSION; ANXIETY
AB Background: Although ketogenic diet therapy (KDT) is a well-established, nonpharmacologic therapeutic option for patients with pharmacoresistant epilepsy, its availability is still not widespread. The COVID-19 pandemic may have further restricted the access of people with pharmacoresistant epilepsy (PWE) to KDT. Thus, we evaluated the experiences of Brazilian PWE and their caregivers during the first year of the pandemic.
   Methods: An online self-assessed survey containing 25 questions was distributed via social media to be answered by PWE treated with KDT or their caregivers through Google Forms from June 2020 to January 2021. Mental health was assessed using the DASS and NDDI-E scales.
   Results: Fifty adults (>18 yo), of whom 68% were caregivers, answered the survey. During the pandemic, 40% faced adversities in accessing their usual healthcare professionals and 38% in obtaining anti-seizure medication (ASM). Despite these issues, 66% of those on KDT could comply with their treatment. Those struggling to maintain KDT (34%) named these obstacles mainly: diet costs, social isolation, food availability, and carbohydrate craving due to anxiety or stress. An increase in seizure frequency was observed in 26% of participants, positively associated with difficulties in obtaining ASM [X-2 (1, N = 48) = 6.55; p = 0.01], but not with KDT compliance issues.
   Conclusions: People with pharmacoresistant epilepsy and undergoing KDT, as well as

their caregivers, faced additional challenges during the COVID-19 pandemic, not only difficulties in accessing healthcare and KDT maintenance but also on seizure control and mental health. (C) 2021 Elsevier Inc. All rights reserved.
C1 [Veiga Domingues Carneiro, Raquel Alencastro; Lunardi, Mariana Dos Santos; Uberna Giacomini, Felipe Matheus; Lin, Katia] Fed Univ Santa Catarina UFSC, Med Sci Grad Program, Florianopolis, SC, Brazil.
[Lunardi, Mariana Dos Santos; Lin, Katia] Fed Univ Santa Catarina UFSC, Neurol Div, Florianopolis, SC, Brazil.
[Rieger, Debora Kurrle; Moreira, Julia Dubois] Fed Univ Santa Catarina UFSC, Grad Program Nutr, Florianopolis, SC, Brazil.
[Rieger, Debora Kurrle; Moreira, Julia Dubois; Ribeiro da Silva, Leticia Carina; Lin, Katia] CNPq Directory Res Grp, Translat Nutr Neurosci Working Grp, Florianopolis, SC, Brazil.
[Ribeiro da Silva, Leticia Carina] Fed Univ Santa Catarina UFSC, Dept Nutr, Florianopolis, SC, Brazil.
[Brito Sampaio, Leticia Pereira] FCMUSP, Sao Paulo, Brazil.
C3 Universidade Federal de Santa Catarina (UFSC); Universidade Federal de Santa Catarina (UFSC); Universidade Federal de Santa Catarina (UFSC); Universidade Federal de Santa Catarina (UFSC)
RP Carneiro, RAVD (通讯作者), Hosp Univ HU UFSC, Dept Clin Med, 3 Andar,Rua Maria Flora Pausewang S-N,Cidade Univ, BR-88040900 Florianopolis, SC, Brazil.
EM raquel.alencastro.c@gmail.com
TC 1
Z9 1
PD SEP
PY 2021
VL 122
AR 108193
DI 10.1016/j.yebeh.2021.108193
EA JUL 2021
WC Behavioral Sciences; Clinical Neurology; Psychiatry
ER

PT J
AU Munoz, EC
Nabbout, R
Guerrero, SB
Herguedas, JL
Villanueva, V
Aibar, JA
AF Munoz, Elena Cardenal
Nabbout, Rima
Guerrero, Susana Boronat
Herguedas, Julian Lara
Villanueva, Vicente
Aibar, Jose Angel
TI Impact of COVID-19 on Spanish patients with Dravet syndrome and their

caregivers: consequences of lockdown
SO REVISTA DE NEUROLOGIA
LA Spanish
DT Article
DE COVID-19; Dravet symdrome; Emergency situation; Healthcare assistance; lockdown; Telemedicine
ID COMORBIDITIES; MANAGEMENT; SEVERITY; FEATURES
AB Introduction. The COVID-19 pandemic caused a state of alarm in Spain in March 2020. The necessary approach to the care of patients with Dravet syndrome (DS) makes them and their caregivers a vulnerable group in emergency situations.

Objectives. To explore the impact of the COVID-19 pandemic on the management and condition of Spanish patients with DS and their caregivers and families.

Materials and methods. Analysis of data belonging to Spanish families taken from a European online survey (14 April-17 May 2020). It included data on DS patients, on the disease and on caregivers before and after lockdown during the state of alarm.

Results. Sixty-nine Spanish families participated; average age of patients: 12.6 years. Except in 19% of the cases that were isolated, protective/isolation measures for patients were followed without increasing. Epilepsy remained stable, with no medication or resource/personnel availability issues. Sleep-wake pattern (61%) and behavior (41%) of patients changed. Behavior change was associated with seizures during lockdown and with caregiver emotional state (changes in 76%). Psychological support was offered to only 9% of caregivers. Thirty-eight per cent of patients did not receive remote care.

Conclusions. The experience gathered during the lockdown has allowed the detection of points of improvement to ensure the proper management of DS and to keep the situation of patients and caregivers stable. All of this with a prominent role of telemedicine.
C1 [Munoz, Elena Cardenal; Aibar, Jose Angel] Fdn Sindrome Dravet, Madrid, Spain.

[Herguedas, Julian Lara] Hosp Univ Puerta Hierro, Unidad Neuropediat, Madrid, Spain.

[Guerrero, Susana Boronat] Hosp Santa Creu & Sant Pau, Serv Pediat, Barcelona, Spain.

[Villanueva, Vicente] Hosp Univ & Politecn La Fe, Unidad Epilepsia Refractaria, Serv Neurol, Valencia, Spain.

[Nabbout, Rima] Univ Paris, Ctr Referencia Epilepslas Raras, Dept Neurol Pediat, Neker Enfants,Inst Imagine, Paris, France.
C3 Hospital Puerta de Hierro-Majadahonda; Hospital of Santa Creu i Sant Pau; Hospital Universitari i Politecnic La Fe; UDICE-French Research Universities; Universite de Paris
RP Munoz, EC (通讯作者), Doctor Fleming 30,1 Izqulerda, E-28036 Madrid, Spain.
EM elena.cardenal@dravetfoundation.eu
TC 1
Z9 1
PD JUL 16
PY 2021
VL 73
IS 2
BP 57
EP 65
DI 10.33588/rn.7302.2021006

WC Clinical Neurology
ER

PT J
AU Chiu, TGA
   Leung, WCY
   Zhang, QQ
   Lau, EHY
   Ho, RWH
   Chan, HSS
   Chang, RSK
AF Chiu, Ting Gee Annie
   Leung, William C. Y.
   Zhang, Qiqi
   Lau, Eric H. Y.
   Ho, Ryan Wui-hang
   Chan, Hoi-Shan Sophelia
   Chang, Richard Shek-kwan
TI Changes in pediatric seizure-related emergency department attendances
   during COVID-19-A territory-wide observational study
SO JOURNAL OF THE FORMOSAN MEDICAL ASSOCIATION
LA English
DT Article
DE COVID19; Epilepsy; Respiratory tract; infections; Medical help
   seek-behaviour; Febrile seizures
ID HONG-KONG; EPIDEMIC; CHILDREN
AB A territory-wide retrospective observational study was conducted in Hong Kong between January 23 to April 22, 2020 to demonstrate changes in pediatric seizure-related accident and emergency department (A&E) visits during the COVID-19 pandemic. Parallel periods from 2015 to 2019 were used as control. All-cause A&E attendances in all paediatric age groups decreased significantly during the study period. Seizure-related attendances decreased across all pediatric age-groups in 2020 (RR 0.379, 95% CI 0.245-0.588), with a disproportionately large decrease in the 0-6 years age group (RR 0.303, 95% CI 0.174-0.52 6) compared with the 7-18 years age group (RR 0.534, 95% CI 0.393-0.719). Decrease in RTI-related A&E attendances was also more drastic in the 0-6 age group. The two time trends are congruent in the 0-6 years but not the 7-18 years age group. Such a trend is suggestive of the usefulness of infection control measures in seizure prevention, especially amongst young children.
C1 [Chiu, Ting Gee Annie; Chan, Hoi-Shan Sophelia] Univ Hong Kong, Li Ka Shing Fac Med, Queen Mary Hosp, Dept Paediat & Adolescent Med, Hong Kong, Peoples R China.
   [Leung, William C. Y.; Chang, Richard Shek-kwan] Univ Hong Kong, Li Ka Shing Fac Med, Queen Mary Hosp, Div Neurol,Dept Med, Hong Kong, Peoples R China.
   [Zhang, Qiqi; Lau, Eric H. Y.] Univ Hong Kong, Li Ka Shing Fac Med, Sch Publ Hlth, Hong Kong, Peoples R China.
   [Ho, Ryan Wui-hang] Univ Hong Kong, Li Ka Shing Fac Med, Dept Med, Queen Mary Hosp, Hong Kong, Peoples R China.
C3 University of Hong Kong; University of Hong Kong; University of Hong

Kong; University of Hong Kong
RP Chang, RSK (通讯作者)，Queen Mary Hosp, Dept Med, Pokfulam, 4-F,Professorial Block, Hong Kong, Peoples R China.
EM changsk@ha.org.hk
TC 2
Z9 2
PD AUG
PY 2021
VL 120
IS 8
BP 1647
EP 1651
DI 10.1016/j.jfma.2020.11.006
EA JUL 2021
WC Medicine, General & Internal
ER

PT J
AU Koh, MY
Lim, KS
Fong, SL
Khor, SB
Tan, CT
AF Koh, May-Yi
Lim, Kheng-Seang
Fong, Si-Lei
Khor, Si-Bao
Tan, Chong-Tin
TI Impact of COVID-19 pandemic on people with epilepsy: An interventional study using early physical consultation
SO EPILEPSY & BEHAVIOR
LA English
DT Article
DE COVID-19; Epilepsy; Anxiety; Depression; Accessibility to clinical service
AB Background: Telehealth use is limited in developing countries. Therefore, a modified approach with early physical consultation was designed and applied in our hospital. This study aimed to determine the efficacy of this early physical consultation in reducing the clinical and psychological impacts of coronavirus disease-19 (COVID-19), which enabled insight into its global feasibility. Method: Participants were contacted and offered early physical consultation with a neurologist. Patients who participated in the Phase 1 study on the impacts of the COVID-19 pandemic on people with epilepsy and treated in our hospital were recruited. Clinical and psychological outcomes of COVID-19 were assessed with the Hospital Anxiety Depression Scale (HADS) and Quality of Life in Epilepsy Inventory (QOLIE-31). Result: A total of 312 patients completed this study with a mean age of 39.13 +/- 16.13 years, majority female (51.0%), and experienced seizures at least once yearly (64.7%). There was 12.6% who experienced

seizure worsening related to the COVID-19 pandemic. After receiving early clinical intervention, 30.8% achieved better seizure control with another 51.1% had no seizure occurrence. The mean HADS anxiety score improved immediately post-intervention (5.27 +/- 4.32 vs. 4.79 +/- 4.26, p < 0.01), and at 2-week post-intervention (5.58 +/- 4.46 vs. 4.73 +/- 3.95, p < 0.01). The mean HADS depression score also improved immediately post-intervention (4.12 +/- 3.69 vs. 3.84 +/- 3.76, p < 0.05) and at 2-week post-intervention (4.38 +/- 3.81 vs. 3.73 +/- 3.63, p < 0.05). The intervention resulted in significant improvement in energy fatigue and social function subscales in QOLIE-31 but a reduction in cognitive and medication effects sub scales. Conclusion: Early physical consultation with stringent precautionary measures is feasible and effective in improving the psychological outcome during COVID-19 pandemic. (c) 2021 Elsevier Inc. All rights reserved.
C1 [Koh, May-Yi; Lim, Kheng-Seang; Fong, Si-Lei; Khor, Si-Bao; Tan, Chong-Tin] Univ Malaya, Fac Med, Dept Med, Div Neurol, Kuala Lumpur, Malaysia.
C3 Universiti Malaya
RP Lim, KS (通讯作者)，Univ Malaya, Med Ctr, Menara Selatan, Neurol Lab, 6th Floor, Kuala Lumpur 50603, Malaysia.
EM kslimum@gmail.com
TC 1
Z9 1
PD SEP
PY 2021
VL 122
AR 108215
DI 10.1016/j.yebeh.2021.108215
EA JUL 2021
WC Behavioral Sciences; Clinical Neurology; Psychiatry
ER

PT J
AU Sato, K
Mano, T
Niimi, Y
Iwata, A
Toda, T
Iwatsubo, T
AF Sato, Kenichiro
Mano, Tatsuo
Niimi, Yoshiki
Iwata, Atsushi
Toda, Tatsushi
Iwatsubo, Takeshi
TI The impact of COVID-19 pandemic on the utilization of ambulatory care for patients with chronic neurological diseases in Japan: Evaluation of an administrative claims database
SO BIOSCIENCE TRENDS
LA English

DT Article
DE COVID-19; care; chronic neurological disease; administrative claims data; telemedicine
AB The COVID-19 pandemic has affected not only the emergency medical system, but also patients' regular ambulatory care, as such decrease in the number of patients visiting outpatient clinics decreased in 2020 than in 2019, or the ban lifting of subsequent visits by telephone for outpatient clinics since March 2020 in lieu of ambulatory care for chronic diseases. In this context, we investigate the impact of the COVID-19 pandemic on ambulatory care at Japanese outpatient clinics for patients with chronic neurological diseases during 2020. We collected data from the administrative claims database (DeSC database) covering more than 1 million individuals. Serial changes in the frequency of subsequent outpatient visits to clinics or hospitals (excluding large hospitals) for chronic ambulatory care of epilepsy, migraine, Parkinson's disease (PD), and Alzheimer's disease (AD) in 2020 were measured. As a result, since April 2020, the monthly outpatient visits for epilepsy, PD, and AD decreased slightly but significantly (approximately 0.90 in relative risk [RR]) but visits for migraine increased (RR = 1.15). Telephone visit was most frequently used in April-May, in less than 5% of monthly outpatient clinic visits for the examined neurological diseases. Outpatient visits for migraine treatment were more likely to be done by telephone than in case of other diseases (adjusted Odds ratio = 2.08). These results suggest that the impact of COVID-19 pandemic on regular ambulatory care for several chronic neurological diseases yielded different effect depending on the disease, in terms of the frequency or type of outpatient visits.
C1 [Sato, Kenichiro; Iwatsubo, Takeshi] Univ Tokyo, Grad Sch Med, Dept Neuropathol, Tokyo, Japan.
[Sato, Kenichiro; Mano, Tatsuo; Toda, Tatsushi] Univ Tokyo Hosp, Dept Neurol, Tokyo, Japan.
[Niimi, Yoshiki; Iwatsubo, Takeshi] Univ Tokyo Hosp, Unit Early & Exploratory Clin Dev, Tokyo, Japan.
[Iwata, Atsushi] Tokyo Metropolitan Geriatr Ctr Hosp, Dept Neurol, Tokyo, Japan.
C3 University of Tokyo; University of Tokyo; University of Tokyo
RP Iwata, A (通讯作者), Tokyo Metropolitan Geriatr Ctr Hosp, Dept Neurol, Itabashi Ku, 35-2 Sakaecho, Tokyo 1730015, Japan.
EM iwata-tky@umin.ac.jp
TC 1
Z9 1
PD AUG
PY 2021
VL 15
IS 4
BP 219
EP 230
DI 10.5582/bst.2021.01194
WC Biology
ER

PT J

AU Beniczky, S
Husain, A
Ikeda, A
Alabri, H
Cross, JH
Wilmshurst, J
Seeck, M
Focke, N
Braga, P
Wiebe, S
Schuele, S
Trinka, E
AF Beniczky, Sandor
Husain, Aatif
Ikeda, Akio
Alabri, Haifa
Cross, J. Helen
Wilmshurst, Jo
Seeck, Margitta
Focke, Niels
Braga, Patricia
Wiebe, Samuel
Schuele, Stephan
Trinka, Eugen
TI Importance of access to epilepsy monitoring units during the COVID-19 pandemic: consensus statement of the International League Against Epilepsy and the International Federation of Clinical Neurophysiology
SO EPILEPTIC DISORDERS
LA English
DT Article
DE COVID-19; epilepsy monitoring units (EMUs); ILAE; IFCN; video-EEG
ID EEG; MISDIAGNOSIS; SURGERY; MORTALITY; CHILDREN
AB Restructuring of healthcare services during the COVID-19 pandemic has led to lockdown of epilepsy monitoring units (EMUs) in many hospitals. The ad-hoc taskforce of the International League Against Epilepsy (ILAE) and the International Federation of Clinical Neurophysiology (IFCN) highlights the detrimental effect of postponing video-EEG monitoring of patients with epilepsy and other paroxysmal events. The taskforce calls for action for continued functioning of EMUs during emergency situations, such as the COVID-19 pandemic. Long-term video-EEG monitoring is an essential diagnostic service. Access to video-EEG monitoring of the patients in the EMUs must be given high priority. Patients should be screened for COVID-19, before admission, according to the local regulations. Local policies for COVID-19 infection control should be adhered to during the video-EEG monitoring. In cases of differential diagnosis in which reduction of antiseizure medication is not required, home video-EEG monitoring should be considered as an alternative in selected patients.
C1 [Beniczky, Sandor] Aarhus Univ Hosp, Dept Clin Neurophysiol, Neurol, Aarhus, Denmark.

[Beniczky, Sandor] Danish Epilepsy Ctr, Dianalund, Denmark.
[Husain, Aatif] Duke Univ, Med Ctr, Dept Neurol, Durham, NC USA.
[Husain, Aatif] Vet Affairs Med Ctr, Neurodiagnost Ctr, Durham, NC USA.
[Ikeda, Akio] Kyoto Univ, Dept Epilepsy Movement Disorders & Physiol, Grad Sch Med, Kyoto, Japan.
[Alabri, Haifa] Sultan Qaboos Univ, Sultan Qaboos Univ Hosp, Dept Med, Neurol Unit, Muscat, Oman.
[Cross, J. Helen] Great Ormond St Hosp Sick Children, UCL NIHR BRC Great Ormond St Inst Child Hlth, London, England.
[Cross, J. Helen] ERN EpiCARE, Bron, France.
[Cross, J. Helen] Young Epilepsy Lingfield, Dormansland, England.
[Wilmshurst, Jo] Univ Cape Town, Red Cross War Mem Childrens Hosp, Neurosci Inst, Dept Paediat Neurol, Cape Town, South Africa.
[Seeck, Margitta] Univ Geneva, Med Fac, Univ Hosp Geneva, EEG & Epilepsy Unit, Geneva, Switzerland.
[Focke, Niels] Univ Med Ctr, Dept Neurol, Gottingen, Germany.
[Braga, Patricia] Univ Republica, Hosp Clin, Fac Med, Inst Neurol, Montevideo, Uruguay.
[Wiebe, Samuel] Univ Calgary, Dept Clin Neurosci, Calgary, AB, Canada.
[Schuele, Stephan] Northwestern Univ, Dept Neurol, Feinberg Sch Med, Chicago, IL 60611 USA.
[Trinka, Eugen] Paracelsus Med Univ, Christian Doppler Univ Hosp, Dept Neurol, Salzburg, Austria.
[Trinka, Eugen] Ctr Cognit Neurosci, Salzburg, Austria.
[Trinka, Eugen] Christian Doppler Univ Hosp, Neurosci Inst, Salzburg, Austria.
[Trinka, Eugen] UMIT Univ Hlth Sci Med Informat & Technol, Dept Publ Hlth Hlth Serv Res & Hlth Technol Asses, Hall In Tirol, Austria.
C3 Aarhus University; Duke University; US Department of Veterans Affairs; Veterans Health Administration (VHA); Kyoto University; Sultan Qaboos University; University of London; University College London; Great Ormond Street Hospital for Children NHS Foundation Trust; University of Cape Town; League of European Research Universities - LERU; University of Geneva; University of Gottingen; Universidad de la Republica, Uruguay; University of Calgary; Northwestern University; Feinberg School of Medicine; Paracelsus Private Medical University; UMIT - Private University for Health Sciences, Medical Informatics & Technology GmbH
RP Beniczky, S (通讯作者), Danish Epilepsy Ctr, Clin Neurophysiol, Visbys Alle 5, DK-4293 Dianalund, Denmark.
EM sbz@filadelfia.dk
TC 1
Z9 1
PD AUG
PY 2021
VL 23
IS 4
BP 533
EP 536

DI 10.1684/epd.2021.1292
WC Clinical Neurology
ER

PT J
AU Sahin, S
   Karsidag, S
   Cinar, N
   Ates, MF
   Demir, S
   Eren, F
   Neyal, A
   Ak, AK
   Tokcaer, AB
   Ataoglu, EE
   Akkaya, SN
   Demirel, EA
   Koc, F
   Ozturk, S
   Firat, YE
   Okluoglu, T
   Togrol, E
   Erdemoglu, AK
   Ergin, N
   Sayin, R
   Demir, A
   Yilmaz, SE
AF Sahin, Sevki
   Karsidag, Sibel
   Cinar, Nilgun
   Ates, Miruna Florentina
   Demir, Serkan
   Eren, Fettah
   Neyal, Abdurrahman
   Kisabay Ak, Aysin
   Bora Tokcaer, Ayse
   Erkoc Ataoglu, Esra
   Akkaya, Seda Nur
   Aciman Demirel, Esra
   Koc, Filiz
   Ozturk, Serefnur
   Ekmekyapar Firat, Yasemin
   Okluoglu, Tugba
   Togrol, Erdem
   Erdemoglu, Ali Kemal
   Ergin, Nesrin
   Sayin, Refah

Demir, Aysegul
Yilmaz, Sueda Ecem
TI The Impact of the COVID-19 Lockdown on the Quality of Life in Chronic Neurological Diseases: The Results of a COVQoL-CND Study
SO EUROPEAN NEUROLOGY
LA English
DT Article
DE COVID-19; Lockdown; Posttraumatic stress; Quality of life; Neurological disease
ID PSYCHOMETRIC PROPERTIES; EVENT SCALE; IES-R; QUARANTINE; OUTBREAK
AB Background: Coronavirus disease 2019 (COVID-19) pandemic and lockdown period may induce an impairment in quality of life (QoL), disruption in treatment (DIT), and posttraumatic stress disorder (PTSD) in chronic neurological diseases (CNDs). To reach this information, a multicenter, cross-sectional study (COVQoL-CND) was planned. Parkinson's disease (PD), headache (HA), multiple sclerosis (MS), epilepsy (EP), polyneuropathy (PNP), and cerebrovascular disease (CVD) were selected as the CND. Methods: The COVQoL-CND study includes demographic data, the World Health Organization Quality of Life short form (WHOQOL-BREF), and Impact of Event Scale-Revised (IES-R) forms. Results: The mean age of a total of 577 patients was 49 +/- 17 (19-87 years), and the ratio of female/male was 352/225. The mean age of patients with PD, HA, MS, EP, PNP, and CVD were 65 +/- 11, 39 +/- 12, 38 +/- 10, 47 +/- 17, 61 +/- 12, and 60 +/- 15 years, respectively. The IES-R scores were found to be higher in the younger group, those with comorbid disease, contacted with CO-VID-19 patients, or diagnosed with COVID-19. In the group with a high IES-R score, the rate of DIT was found to be high. IES-R scores were negatively correlated with QoL. IES-R total scores were found highest in the CVD group and lowest in the PD group. The ratio of DIT was found highest in the PNP group and the lowest in the EP group. Contact with CO-VID-19 patients was high in the EP and HA group. Conclusions: The results of the COVQoL-CND study showed that lockdown causes posttraumatic stress and deterioration in the QoL in CND.
C1 [Sahin, Sevki; Karsidag, Sibel; Cinar, Nilgun; Ates, Miruna Florentina] Maltepe Univ, Sch Med, Dept Neurol, Istanbul, Turkey.
[Demir, Serkan] Univ Hlth Sci, Sancaktepe Res & Training Hosp, Dept Neurol, Istanbul, Turkey.
[Eren, Fettah; Ozturk, Serefnur; Yilmaz, Sueda Ecem] Selcuk Univ, Sch Med, Dept Neurol, Konya, Turkey.
[Neyal, Abdurrahman] Dr Ersin Arslan Res & Training Hosp, Gaziantep, Turkey.
[Kisabay Ak, Aysin] Celal Bayar Univ, Sch Med, Dept Neurol, Manisa, Turkey.
[Bora Tokcaer, Ayse; Erkoc Ataoglu, Esra; Akkaya, Seda Nur] Gazi Univ, Sch Med, Dept Neurol, Ankara, Turkey.
[Erkoc Ataoglu, Esra; Aciman Demirel, Esra] Bulent Ecevit Univ, Fac Med, Dept Neurol, Zonguldak, Turkey.
[Koc, Filiz] Cukurova Univ, Fac Med, Dept Neurol, Adana, Turkey.
[Ekmekyapar Firat, Yasemin] SANKO Univ, Sch Med, Dept Neurol, Gaziantep, Turkey.
[Okluoglu, Tugba] Hlth Sci Univ, Istanbul Educ Res Hosp, Dept Neurol, Istanbul, Turkey.
[Togrol, Erdem] Univ Hlth Sci, Hamidiye Sch Med, Sultan Abdulhamid Han Res & Training Hosp, Dept Neurol, Istanbul, Turkey.

[Erdemoglu, Ali Kemal] Erdemoglu Neurol & Pain Clin, Ankara, Turkey.
[Ergin, Nesrin] Pamukkale Univ, Med Fac, Dept Neurol, Denizli, Turkey.
[Sayin, Refah] Ufuk Univ, Sch Med, Dept Neurol, Ankara, Turkey.
[Demir, Aysegul] Konya City Hosp, Dept Neurol, Konya, Turkey.
C3 Maltepe University; University of Health Sciences Turkey; Selcuk University; Dr. Ersin Arslan Education & Research Hospital; Celal Bayar University; Gazi University; Bulent Ecevit University; Cukurova University; Gaziantep University; Sanko University; Istanbul Training & Research Hospital; University of Health Sciences Turkey; University of Health Sciences Turkey; Pamukkale University; Ufuk University
RP Sahin, S (通讯作者), Maltepe Univ, Sch Med, Dept Neurol, Istanbul, Turkey.
EM drsahin@gmail.com
TC 1
Z9 1
PD NOV
PY 2021
VL 84
IS 6
BP 450
EP 459
DI 10.1159/000517380
EA AUG 2021
WC Clinical Neurology; Neurosciences
ER

PT J
AU Babtain, F
Atteyah, D
Milyani, H
Banjer, T
Alqadi, K
Baeesa, S
Al Said, Y
AF Babtain, Fawzi
Atteyah, Daniah
Milyani, Haneen
Banjer, Tasnim
Alqadi, Khalid
Baeesa, Saleh
Al Said, Youssef
TI The safety and efficacy of modifying the admission protocol to the epilepsy monitoring unit in response to the COVID-19 pandemic
SO EPILEPSY & BEHAVIOR
LA English
DT Article
DE Antiepileptic drug; COVID-19; Elective admission; Epilepsy monitoring unit; Seizure; Epilepsy

ID EEG; WITHDRAWAL; IMPACT

AB Purpose: The coronavirus disease 2019 (COVID-19) pandemic has impacted admission to epilepsy mon-itoring units (EMUs) for classification and presurgical evaluation of patients with refractory epilepsy. We modified the EMU admission protocol via anti-seizure medications (ASM) withdrawal implemented one day before admission; thus, we aimed to evaluate the efficacy and safety of this modified protocol. Methods: In January 2021, we initiated ASM tapering 24 h before-rather than on the first day after-EMU admission, contrasting with the previous protocol. We retrospectively reviewed EMU admissions between January and April of 2018, 2019, and 2021, and identified the time required to record the first seizure, and EMU yield to confirm or change the epilepsy classification. We also evaluated the safety of the modified protocol, by monitoring the seizure frequency for up to 5 months after the discharge from the hospital. Results: One hundred four patients were included (mean age: 30 years, men: 43%); excluding a longer disease duration and abundance of normal routine electro-encephalogram (EEG) in patients admitted before the pandemic, no differences were observed in patients' characteristics. On average, it took 41 h and 21 h to record the first seizure using the standard and modified protocols, respectively ($p < 0.001$, 95% CI: 10-30). Other characteristics were investigated both before and after the COVID-19 pandemic, and epilepsy classifications were confirmed twice using the modified protocol (OR = 2.4, $p = 0.04$, 95% CI: 1.1-5.5). Multivariate regression analysis confirmed the shorter time to record the first seizure using the modified admission protocol (23 h less, $p < 0.001$; 95% CI: 12-34). Finally, 36 (86%) patients admitted during the pandemic exhibited no increase in seizure frequency after the discharge from the hospital. Conclusions: Initiating ASM withdrawal one day before EMU admission was deemed to be an efficient and safe way to confirm epilepsy classification and significantly decrease the length of hospital stay. Ultimately, this will shorten the long waiting list for EMU admission created by the COVID-19 pandemic. (c) 2021 Elsevier Inc. All rights reserved.

C1 [Babtain, Fawzi; Atteyah, Daniah; Milyani, Haneen; Banjer, Tasnim; Alqadi, Khalid; Baeesa, Saleh; Al Said, Youssef] King Faisal Specialist Hosp & Res Ctr, Dept Neurosci, Al Rawdah Rd,POB 40047, Jeddah 21499, Saudi Arabia.

C3 King Faisal Specialist Hospital & Research Center

RP Babtain, F (通讯作者), King Faisal Specialist Hosp & Res Ctr, Dept Neurosci, Al Rawdah Rd,POB 40047, Jeddah 21499, Saudi Arabia.

EM fbabtain@kfshrc.edu.sa

TC 1

Z9 1

PD SEP

PY 2021

VL 122

AR 108229

DI 10.1016/j.yebeh.2021.108229

EA AUG 2021

WC Behavioral Sciences; Clinical Neurology; Psychiatry

ER

PT J

AU Belluzzo, M
Nilo, A
Valente, M
Gigli, GL
AF Belluzzo, Marco
Nilo, Annacarmen
Valente, Mariarosaria
Gigli, Gian Luigi
TI New-onset status epilepticus in SARS-CoV-2 infection: a case series
SO NEUROLOGICAL SCIENCES
LA English
DT Article
DE Status epilepticus; SARS-CoV-2; Epilepsy; EEG; Recurrent SE
ID NMDA RECEPTOR ENCEPHALITIS
AB Background Neurological manifestations of COVID-19 infection are well recognized. Seizures and status epilepticus (SE) have been reported as possible manifestations and/or complications of SARS-CoV-2 infection at different disease stages, but few data are known about the type, severity, treatment response, and recurrence. Methods Single-center retrospective case series. Results This case series describes four COVID-19-positive patients admitted to an Italian University Hospital, who developed status epilepticus during the active phase of disease, independently from the severity of respiratory symptoms. Two of them presented a relapse after resolution of the acute viral infection, a feature that has not been previously reported. Conclusions Although a possible association between SE and COVID-19 has been reported, the exact etiopathogenetic mechanism remains still not understood. Our series adds new insights to shed further light on this controversial issue.
C1 [Belluzzo, Marco] S Maria Della Misericordia Univ Hosp, Dept Neurosci, Neurol Unit, Piazzale S Maria della Misericordia 15, I-33100 Udine, Italy.
[Nilo, Annacarmen; Valente, Mariarosaria; Gigli, Gian Luigi] S Maria Della Misericordia Univ Hosp, Dept Neurosci, Clin Neurol Unit, Udine, Italy.
[Valente, Mariarosaria; Gigli, Gian Luigi] Univ Udine, Med Sch, Dept Med, Udine, Italy.
C3 Hospital Santa Maria della Misericordia; Hospital Santa Maria della Misericordia; University of Udine
RP Belluzzo, M (通讯作者), S Maria Della Misericordia Univ Hosp, Dept Neurosci, Neurol Unit, Piazzale S Maria della Misericordia 15, I-33100 Udine, Italy.
EM marco.belluzzo@hotmail.com
TC 0
Z9 0
PD MAR
PY 2022
VL 43
IS 3
BP 2015
EP 2020
DI 10.1007/s10072-021-05536-0
EA AUG 2021

WC Clinical Neurology; Neurosciences
ER

PT J
AU Song, PH
Cao, D
Li, SR
Wang, R
Wang, YP
Lin, H
AF Song, Penghui
Cao, Dan
Li, Siran
Wang, Rong
Wang, Yuping
Lin, Hua
TI Effects of hyperventilation with face mask on brain network in patients with epilepsy
SO EPILEPSY RESEARCH
LA English
DT Article
DE COVID-19; Hyperventilation; Electroencephalogram; Face mask; Cortical excitability
ID ACUTE-HYPOXIA; EEG; ORGANIZATION
AB Objectives: During the ongoing pandemic of COVID-19, wearing face masks was recommended, including patients with epilepsy doing the hyperventilation (HV) test during electroencephalogram (EEG) examination somewhere. However, evidence was still limited about the effect of HV with face mask on cortical excitability of patients with epilepsy. The motivation of this work is to make use of the graph theory of EEG to characterize the cortical excitability of patients with epilepsy when they did HV under the condition wearing a surgical face mask.

Methods: We recruited 19 patients with epilepsy and 17 normal controls. All of participants completed two HV experiments, including HV with face mask (HV+) and HV without a mask (HV). The interval was 30 min and the sequence was random. Each experiment consisted of three segments: resting EEG, EEG of HV, and EEG of postHV. EEG were recorded successively during each experiment. Participants were asked to evaluate the discomfort degree using a questionnaire when every HV is completed.

Results: All of the participants felt more uncomfortable after HV + . Moreover, not only HV decreased smallworldness index in patients with epilepsy, but also HV + significantly increased the clustering coefficient in patients with epilepsy. Importantly, the three-way of Mask*HV*Epilepsy showed interaction in the clustering coefficient in the delta band, as well as in the path length and the small-worldness index in the theta band.

Conclusions: The results of this study indicated that patients with epilepsy showed the increased excitability of brain network during HV + . We should pay more attention to the adverse effect on brain network excitability caused by HV + in patients with epilepsy. In the clinical practice under the COVID-19 pandemic, it is important that

the wearing face mask remain cautious for the individuals with epilepsy when they carried out HV behavior such as exercise (e.g., running, etc.).
C1 [Song, Penghui; Li, Siran; Wang, Yuping; Lin, Hua] Capital Med Univ, Xuanwu Hosp, Dept Neurol, Beijing 100053, Peoples R China.
[Song, Penghui; Wang, Rong] Capital Med Univ, Xuanwu Hosp, Cent Lab, Beijing 100053, Peoples R China.
[Song, Penghui; Wang, Rong] Beijing Geriatr Med Res Ctr, Beijing 100053, Peoples R China.
[Song, Penghui; Wang, Yuping; Lin, Hua] Beijing Key Lab Neuromodulat, Beijing 100053, Peoples R China.
[Song, Penghui; Wang, Yuping; Lin, Hua] Capital Med Univ, Ctr Epilepsy, Beijing Inst Brain Disorders, Beijing 100053, Peoples R China.
[Cao, Dan] Chinese Acad Sci, Brainnetome Ctr, Inst Automat, Beijing 100190, Peoples R China.
[Cao, Dan] Chinese Acad Sci, Natl Lab Pattern Recognit, Inst Automat, Beijing 100190, Peoples R China.
C3 Capital Medical University; Capital Medical University; Capital Medical University; Chinese Academy of Sciences; Institute of Automation, CAS; Chinese Academy of Sciences; Institute of Automation, CAS
RP Lin, H (通讯作者), Capital Med Univ, Xuanwu Hosp, Dept Neurol, Beijing 100053, Peoples R China.
EM linhua@ccmu.edu.cn
TC 0
Z9 0
PD OCT
PY 2021
VL 176
AR 106741
DI 10.1016/j.eplepsyres.2021.106741
EA AUG 2021
WC Clinical Neurology
ER

PT J
AU Beniczky, S
Husain, A
Ikeda, A
Alabri, H
Cross, JH
Wilmshurst, J
Seeck, M
Focke, N
Braga, P
Wiebe, S
Schuele, S
Trinka, E
AF Beniczky, Sandor

Husain, Aatif
Ikeda, Akio
Alabri, Haifa
Cross, J. Helen
Wilmshurst, Jo
Seeck, Margitta
Focke, Niels
Braga, Patricia
Wiebe, Samuel
Schuele, Stephan
Trinka, Eugen
TI Importance of access to epilepsy monitoring units during the COVID-19 pandemic: Consensus statement of the International League against epilepsy and the International Federation of Clinical Neurophysiology
SO CLINICAL NEUROPHYSIOLOGY
LA English
DT Review
DE COVID-19; Epilepsy; Epilepsy monitoring unit; Video-EEG
ID EEG; SURGERY; MISDIAGNOSIS; MORTALITY
AB Restructuring of healthcare services during the COVID-19 pandemic has led to lockdown of Epilepsy Monitoring Units (EMUS) in many hospitals. The ad-hoc taskforce of the International League Against Epilepsy (ILAE) and the International Federation of Clinical Neurophysiology (IFCN) highlights the detrimental effect of postponing video-EEG monitoring of patients with epilepsy and other paroxysmal events. The taskforce calls for action to continue functioning of Epilepsy Monitoring Units during emergency situations, such as the COVID-19 pandemic. Long-term video-EEG monitoring is an essential diagnostic service. Access to video-EEG monitoring of the patients in the EMUS must be given high priority. Patients should be screened for COVID-19, before admission, according to the local regulations. Local policies for COVID-19 infection control should be adhered to during the video-EEG monitoring. In cases of differential diagnosis where reduction of antiseizure medication is not required, consider home video-EEG monitoring as an alternative in selected patients. (C) 2021 International Federation of Clinical Neurophysiology Inc. and International League Against Epilepsy. Published by Elsevier B.V. All rights reserved.
C1 [Beniczky, Sandor] Aarhus Univ Hosp, Dept Clin Neurophysiol, Neurol, Aarhus, Denmark.
[Beniczky, Sandor] Danish Epilepsy Ctr, Dianalund, Denmark.
[Husain, Aatif] Duke Univ, Dept Neurol, Med Ctr, Durham, NC USA.
[Husain, Aatif] Vet Affairs Med Ctr, Neurodiagnost Ctr, Durham, NC USA.
[Ikeda, Akio] Kyoto Univ, Grad Sch Med, Dept Epilepsy Movement Disorders & Physiol, Kyoto, Japan.
[Alabri, Haifa] Sultan Qaboos Univ, Sultan Qaboos Univ Hosp, Dept Med, Neurol Unit, Muscat, Oman.
[Cross, J. Helen] Great Ormond St Hosp Sick Children, UCL NIHR BRC Great Ormond St Inst Child Hlth, London, England.
[Cross, J. Helen] ERN EpiCARE, London, England.
[Cross, J. Helen] Young Epilepsy Lingfield, Dormansland, Lingfield, England.

[Wilmshurst, Jo] Univ Cape Town, Red Cross War Mem Childrens Hosp, Neurosci Inst, Dept Paediat Neurol, Rondebosch, South Africa.
[Seeck, Margitta] Univ Geneva, Univ Hosp Geneva, EEG & Epilepsy Unit, Med Fac, Geneva, Switzerland.
[Focke, Niels] Univ Med Ctr, Dept Neurol, Gttingen, Germany.
[Braga, Patricia] Univ Republica, Hosp Clin, Fac Med, Inst Neurol, Montevideo, Uruguay.
[Wiebe, Samuel] Univ Calgary, Dept Clin Neurosci, Calgary, AB, Canada.
[Schuele, Stephan] Northwestern Univ, Feinberg Sch Med, Dept Neurol, Chicago, IL 60611 USA.
[Trinka, Eugen] Paracelsus Med Univ, Christian Doppler Univ Hosp, Dept Neurol, Salzburg, Austria.
[Trinka, Eugen] Ctr Cognit Neurosci, Salzburg, Austria.
[Trinka, Eugen] Christian Doppler Univ Hosp, Neurosci Inst, Affiliated EpiCARE Partner, Salzburg, Austria.
[Trinka, Eugen] UMIT Univ Hlth Sci Med Informat & Technol, Dept Publ Hlth Hlth Serv Res & Hlth Technol Asses, Hall In Tirol, Austria.
C3 Aarhus University; Duke University; US Department of Veterans Affairs; Veterans Health Administration (VHA); Kyoto University; Sultan Qaboos University; University of London; University College London; Great Ormond Street Hospital for Children NHS Foundation Trust; University of Cape Town; League of European Research Universities - LERU; University of Geneva; Universidad de la Republica, Uruguay; University of Calgary; Northwestern University; Feinberg School of Medicine; Paracelsus Private Medical University; UMIT - Private University for Health Sciences, Medical Informatics & Technology GmbH
RP Beniczky, S (通讯作者), Visby Alle 5, DK-4293 Dianalund, Denmark.
EM sbz@filadelfia.dk
TC 1
Z9 1
PD SEP
PY 2021
VL 132
IS 9
BP 2248
EP 2250
DI 10.1016/j.clinph.2021.05.001
EA AUG 2021
WC Clinical Neurology; Neurosciences
ER

PT J
AU Rosengard, JL
Ferastraoaru, V
Donato, J
Haut, SR
AF Rosengard, Jillian L.

Ferastraoaru, Victor
Donato, Jad
Haut, Sheryl R.
TI Psychogenic nonepileptic seizures during the COVID-19 pandemic in New York City - A distinct response from the epilepsy experience
SO EPILEPSY & BEHAVIOR
LA English
DT Article
DE Psychogenic nonepileptic seizures (PNES); Functional seizures; Epilepsy; Stress; COVID-19; Pandemic
AB Although psychogenic nonepileptic seizures (PNES) are a common neurologic condition, there remains a paucity of literature on the COVID-19 pandemic's effect on these patients. Using a cross-sectional questionnaire study, our group examined the experience of patients with PNES at a single Comprehensive Epilepsy Center in New York City, the epicenter of the initial COVID-19 outbreak in the United States. Among our cohort of 18 subjects with PNES, 22.2% reported an improvement in seizure control during the peak of the COVID-19 pandemic in New York City. Compared to the cohort of subjects with epilepsy without PNES, subjects with PNES were significantly more likely to report an improvement ($p = 0.033$). Our findings signal that sleep and stress may be relevant variables in both conditions that should be further investigated and potentially intervened upon. Larger dedicated studies of patients with PNES are needed to understand the impact of the pandemic's widespread societal effects on these patients. (c) 2021 Elsevier Inc. All rights reserved.
C1 [Rosengard, Jillian L.; Ferastraoaru, Victor; Donato, Jad; Haut, Sheryl R.] Montefiore Epilepsy Ctr, Saul R Korey Dept Neurol & Comprehens Einstein, Bronx, NY USA.
RP Rosengard, JL (通讯作者), Montefiore Med Ctr, Dept Neurol, NW 002,111 East 210th St, Bronx, NY 10467 USA.
EM jrosenga@montefiore.org
TC 1
Z9 1
PD OCT
PY 2021
VL 123
AR 108255
DI 10.1016/j.yebeh.2021.108255
EA AUG 2021
WC Behavioral Sciences; Clinical Neurology; Psychiatry
ER

PT J
AU Cross, JH
Kwon, CS
Asadi-Pooya, AA
Balagura, G
Gomez-Iglesias, P
Guekht, A
Hall, J

Ikeda, A
Kishk, NA
Murphy, P
Kissani, N
Naji, Y
Perucca, E
Perez-Poveda, JC
Sanya, EO
Trinka, E
Zhou, D
Wiebe, S
Jette, N
AF Cross, J. Helen
Kwon, Churl-Su
Asadi-Pooya, Ali Akbar
Balagura, Ganna
Gomez-Iglesias, Patricia
Guekht, Alla
Hall, Julie
Ikeda, Akio
Kishk, Nirmeen A.
Murphy, Peter
Kissani, Najib
Naji, Yahya
Perucca, Emilio
Perez-Poveda, Juan Carlos
Sanya, Emmanuel O.
Trinka, Eugen
Zhou, Dong
Wiebe, Samuel
Jette, Nathalie
CA ILAE Task Forces COVID-
TI Epilepsy care during the COVID-19 pandemic
SO EPILEPSIA
LA English
DT Article
DE COVID-19; people with epilepsy; psychological distress; telemedicine
ID DISTRESS; PEOPLE
AB The coronavirus disease 2019 (COVID-19) pandemic has affected the care of all patients around the world. The International League Against Epilepsy (ILAE) COVID-19 and Telemedicine Task Forces examined, through surveys to people with epilepsy (PWE), caregivers, and health care professionals, how the pandemic has affected the well-being, care, and services for PWE. The ILAE included a link on their website whereby PWE and/or their caregivers could fill out a survey (in 11 languages) about the impact of the COVID-19 pandemic, including access to health services and impact on mental health, including the 6-item Kessler Psychological Distress Scale. An anonymous link was also provided whereby health care providers could report cases of new-onset seizures or an

exacerbation of seizures in the context of COVID-19. Finally, a separate questionnaire aimed at exploring the utilization of telehealth by health care professionals since the pandemic began was available on the ILAE website and also disseminated to its members. Seventeen case reports were received; data were limited and therefore no firm conclusions could be drawn. Of 590 respondents to the well-being survey (422 PWE, 166 caregivers), 22.8% PWE and 27.5% caregivers reported an increase in seizure frequency, with difficulty in accessing medication and health care professionals reported as barriers to care. Of all respondents, 57.1% PWE and 21.5% caregivers had severe psychological distress (k score >13), which was significantly higher among PWE than caregivers (p<0.01). An increase in telemedicine use during the COVID-19 pandemic was reported by health care professionals, with 40% of consultations conducted by this method. Although 74.9% of health care providers thought that this impacted positively, barriers to care were also identified. As we move forward, there is a need to ensure ongoing support and care for PWE to prevent a parallel pandemic of unmet health care needs.
C1 [Cross, J. Helen] Great Ormond St Hosp Sick Children, UCL NIHR BRC Great Ormond St Inst Child Hlth, Programme Dev Neurosci, London, England.

[Cross, J. Helen] Young Epilepsy Lingfield, London, England.

[Kwon, Churl-Su; Jette, Nathalie] Icahn Sch Med Mt Sinai, Dept Neurol, Div Hlth Outcomes & Knowledge Translat Res, New York, NY 10029 USA.

[Asadi-Pooya, Ali Akbar] Shiraz Univ Med Sci, Epilepsy Res Ctr, Shiraz, Iran.

Thomas Jefferson Univ, Dept Neurol, Jefferson Comprehens Epilepsy Ctr, Philadelphia, PA 19107 USA.

[Balagura, Ganna] Univ Genoa, IRCCS G Gaslini Inst, Dept Neurosci Rehabil Ophthalmol Genet & Maternal, Pediat Neurol & Muscular Dis Unit, Genoa, Italy.

[Gomez-Iglesias, Patricia] Univ Complutense Madrid, Hlth Res Inst San Carlos IdISCC, Hosp Clin San Carlos, Dept Neurol,Epilepsy Unit, Madrid, Spain.

[Guekht, Alla] Moscow Res & Clin Ctr Neuropsychiat, Moscow, Russia.

[Guekht, Alla] Russian Natl Res Med Univ, Dept Neurol Neurosurg & Med Genet, Moscow, Russia.

[Hall, Julie] Int League Epilepsy, Flower Mound, TX USA.

[Ikeda, Akio] Kyoto Univ, Grad Sch Med, Dept Epilepsy Movement Disorders & Physiol, Kyoto, Japan.

[Kishk, Nirmeen A.] Cairo Univ, Neurol Dept, Fac Med, Cairo, Egypt.

[Murphy, Peter] Epilepsy Ireland, Dublin, Ireland.

[Kissani, Najib; Naji, Yahya] Univ Cadi Ayyad, Marrakech Med Sch, Neurosci Res Lab, Marrakech, Morocco.

[Kissani, Najib; Naji, Yahya] Univ Teaching Hosp Mohammed VI, Neurol Dept, Marrakech, Morocco.

[Perucca, Emilio] Univ Pavia, Dept Internal Med & Therapeut, Div Clin & Expt Pharmacol, Pavia, Italy.

[Perucca, Emilio] IRCCS Mondino Fdn, Pavia, Italy.

[Perucca, Emilio] ERN EpiCARE, Pavia, Italy.

[Perez-Poveda, Juan Carlos] Xavierian Univ, Fac Med, Neurosci Dept, Bogota, Colombia.

[Perez-Poveda, Juan Carlos] Hosp Univ San Ignacio, Bogota, Colombia.

[Sanya, Emmanuel O.] Univ Ilorin Teaching Hosp, Med Dept, Neurol Div, Ilorin, Kwara

State, Nigeria.
[Trinka, Eugen] Paracelsus Med Univ, Christian Doppler Univ Hosp, Ctr Cognit Neurosci, Dept Neurol, Salzburg, Austria.
[Trinka, Eugen] Paracelsus Med Univ, Ctr Cognit Neurosci, Christian Doppler Univ Hosp, Neurosci Inst, Salzburg, Austria.
[Trinka, Eugen] Private Univ Hlth Sci, Med Informat & Technol, Med Decis Making & HTA, Inst Publ Hlth,UMIT, Hall In Tirol, Austria.
[Zhou, Dong] Sichuan Univ, West China Hosp, Dept Neurol, Chengdu, Peoples R China.
[Wiebe, Samuel] Univ Calgary, Cumming Sch Med, Dept Clin Neurosci, Calgary, AB, Canada.
[Wiebe, Samuel] Univ Calgary, Cumming Sch Med, Dept Community Hlth Sci, Calgary, AB, Canada.
C3 University of London; University College London; Great Ormond Street
Hospital for Children NHS Foundation Trust; Icahn School of Medicine at
Mount Sinai; Shiraz University of Medical Science; Jefferson University;
University of Genoa; IRCCS Istituto Giannina Gaslini; Complutense
University of Madrid; Hospital Clinico San Carlos; Pirogov Russian
National Research Medical University; Kyoto University; Egyptian
Knowledge Bank (EKB); Cairo University; Cadi Ayyad University of
Marrakech; University of Pavia; IRCCS Fondazione Casimiro Mondino;
Hospital Universitario San Ignacio; University of Ilorin; Paracelsus
Private Medical University; Paracelsus Private Medical University;
Sichuan University; University of Calgary; University of Calgary
RP Cross, JH (通讯作者), UCL Great Ormond St Inst Child Hlth, Childhood Epilepsy, 30 Guilford St, London WC1N 1EH, England.
EM h.cross@ucl.ac.uk
TC 4
Z9 4
PD OCT
PY 2021
VL 62
IS 10
BP 2322
EP 2332
DI 10.1111/epi.17045
EA AUG 2021
WC Clinical Neurology
ER

PT J
AU Sin, R
Struncova, D
AF Sin, Robin
Struncova, Denisa
TI Status epilepticus as a complication after COVID-19 mRNA-1273 vaccine: A
case report
SO WORLD JOURNAL OF CLINICAL CASES

LA English
DT Article
DE SARS-CoV-2; COVID-19; mRNA vaccine; Complication of vaccination; Status epilepticus; Case report
ID WUHAN
AB BACKGROUND We present a rare case of status epilepticus in a 56-year-old man which arose as a complication after vaccination with the coronavirus disease 2019 (COVID-19) mRNA-1273 vaccine. The patient's history included well-compensated secondary epilepsy. The root cause of the situation was a fever which had developed as a side effect of the vaccination. CASE SUMMARY A 56-year-old man received the first dose of mRNA-1273 vaccine against the severe acute respiratory syndrome-coronavirus-2. The vaccine was administered intramuscularly (100 mg, 0.5 mL). The next morning the man was found to be suffering from fever and headaches while at the same time experiencing general weakness. He lost consciousness suddenly and experienced generalized clonic seizures which turned into status epilepticus. When the Emergency Medical Service arrived the patient was unconscious with spontaneous breathing and generalized clonic seizures. It was necessary to administer diazepam repeatedly. It was also necessary to administer high doses of levetiracetam and temporary propofol. The status epilepticus was brought under control approximately 90 min after the patient's transport to the Emergency Department. A follow-up electroencephalogram no longer revealed abnormal indications of epileptic fit. The patient was temporarily hospitalized in the Intensive Care Unit and after seven days care was discharged without any further apparent effects. CONCLUSION There is currently no specific treatment against COVID-19. Therefore, the benefits of COVID-19 vaccine protection outweigh the risks.
C1 [Sin, Robin] Charles Univ Prague, Univ Hosp Pilsen, Fac Med Pilsen, Dept Infect Dis & Travel Med, Plzen 30599, Czech Republic.
[Sin, Robin] Emergency Med Serv Pilsen Reg, Dept Med, Plzen 30100, Czech Republic.
[Struncova, Denisa] Univ Hosp Plzen, Dept Anaesthesiol & Intens Care Med, Alej Svobody 80, Plzen 30460, Czech Republic.
[Struncova, Denisa] Masaryk Univ, Fac Med, Brno 62500, Czech Republic.
C3 Charles University Prague; University Hospital Plzen; Masaryk University Brno
RP Struncova, D (通讯作者), Univ Hosp Plzen, Dept Anaesthesiol & Intens Care Med, Alej Svobody 80, Plzen 30460, Czech Republic.
EM denisastruncova@seznam.cz
TC 1
Z9 1
PD AUG 26
PY 2021
VL 9
IS 24
BP 7218
EP 7223
DI 10.12998/wjcc.v9.i24.7218
WC Medicine, General & Internal
ER

PT J
AU Blenkinsop, S
Foley, A
Schneider, N
Willis, J
Fowler, HJ
Sisodiya, SM
AF Blenkinsop, Stephen
Foley, Aideen
Schneider, Natascha
Willis, Joseph
Fowler, Hayley J.
Sisodiya, Sanjay M.
TI Carbon emission savings and short-term health care impacts from telemedicine: An evaluation in epilepsy
SO EPILEPSIA
LA English
DT Article
DE climate change; global heating; neurology; pandemic
ID ENERGY; HOME
AB Objective: Health systems make a sizeable contribution to national emissions of greenhouse gases that contribute to global climate change. The UK National Health Service is committed to being a net zero emitter by 2040, and a potential contribution to this target could come from reductions in patient travel. Achieving this will require actions at many levels. We sought to determine potential savings and risks over the short term from telemedicine through virtual clinics.

Methods: During the severe acute respiratory syndrome coronavirus 2 (SARS-2-CoV) pandemic, scheduled face-to-face epilepsy clinics at a specialist site were replaced by remote teleclinics. We used a standard methodology applying conversion factors to calculate emissions based on the total saved travel distance. A further conversion factor was used to derive emissions associated with electricity consumption to deliver remote clinics from which net savings could be calculated. Patients' records and clinicians were interrogated to identify any adverse clinical outcomes.

Results: We found that enforced telemedicine delivery for over 1200 patients resulted in the saving of similar to 224 000 km of travel with likely avoided emissions in the range of 35 000-40 000 kg carbon dioxide equivalent (CO(2)e) over a six and half month period. Emissions arising directly from remote delivery were calculated to be <200 kg CO(2)e (similar to 0.5% of those for travel), representing a significant net reduction of greenhouse gas emissions. Only one direct adverse outcome was identified, with some additional benefits identified anecdotally.

Significance: The use of telemedicine can make a contribution toward reduced emissions in the health care sector and, in the delivery of specialized epilepsy services, had minimal adverse clinical outcomes over the short term. However, these outcomes will likely vary with clinic locations, medical specialties and conditions.
C1 [Blenkinsop, Stephen; Fowler, Hayley J.] Newcastle Univ, Sch Engn, Newcastle Upon Tyne, Tyne & Wear, England.
[Foley, Aideen] Univ London, Dept Geog, Birkbeck Coll, London, England.

[Schneider, Natascha; Willis, Joseph; Sisodiya, Sanjay M.] UCL Queen Sq Inst Neurol, Dept Clin & Expt Epilepsy, London WC1N 3BG, England.
[Sisodiya, Sanjay M.] Chalfont Ctr Epilepsy, Gerrards Cross, Bucks, England.
C3 Newcastle University - UK; University of London; Birkbeck University London; University of London; University College London
RP Sisodiya, SM (通讯作者), UCL Queen Sq Inst Neurol, Dept Clin & Expt Epilepsy, London WC1N 3BG, England.
EM s.sisodiya@ucl.ac.uk
TC 0
Z9 0
PD NOV
PY 2021
VL 62
IS 11
BP 2732
EP 2740
DI 10.1111/epi.17046
EA AUG 2021
WC Clinical Neurology
ER

PT J
AU Mishra, VN
Pathak, A
Chaurasia, RN
Kumar, A
Joshi, D
Singh, VK
AF Mishra, Vijaya Nath
Pathak, Abhishek
Chaurasia, Rameshwar Nath
Kumar, Anand
Joshi, Deepika
Singh, Varun Kumar
TI Observations in a Virtual Telephone and WhatsApp Video-Enabled Neurology Clinic During Lockdown in Varanasi, India - A Preliminary Report
SO NEUROLOGY INDIA
LA English
DT Article
DE COVID-19; lockdown; smartphone; teleneurology; WhatsApp
ID TELEMEDICINE; TELESTROKE; TELENEUROLOGY; COVID-19; STROKE; CARE; IMPLEMENTATION; THROMBOLYSIS; EFFICACY; FUTURE
AB Background: Globally, social distancing has been practiced during the ongoing coronavirus disease 2019 (COVID-19) pandemic to prevent the transmission of the virus. One of the measures to ensure social distancing and restricting the movements has been national lockdown, to break the chain of transmission. Telemedicine is a cost-effective measure to provide medical services to remote underserved areas.

Objective: The present study aimed to evaluate the efficacy and acceptability of teleconsultation as an alternative option to in-person consultation in providing continued medical care for neurology patients during the national lockdown period of the COVID-19 pandemic.
Materials and Methods: The clinical demographic profile, frequency of different neurological disorders, and treatment details of the patients attending the teleneurology consultation (TNCO) outpatient department (OPD) at Sir Sunderlal Hospital (S.S.H.), Institute of Medical Sciences (I.M.S.), BHU, Varanasi, India, were recorded in a prespecified pro forma.
Results: A total of 1,567 patients attended the TNCO OPD over 90 days. The average patient attendance was 35 per day, and 72% were males. Out of these, 77% of patients were from the same district, and the majority of patients (68%) were regularly followed up in-person by the neurology OPD. The most common illness for consultation was epilepsy (19%) followed by low backache and stroke (18% each). The satisfaction rate among the patients with respect to teleservices was high (90%).
Conclusion: TNCO seems to be as effective as in-person OPD in the management of neurological disorders. During the lockdown due to the COVID-19 pandemic, avoiding physical visits through TNCO may reduce the spread of the virus. Parallel tele-OPD with routine OPD is a good option in the future.
C1 [Mishra, Vijaya Nath; Pathak, Abhishek; Chaurasia, Rameshwar Nath; Kumar, Anand; Joshi, Deepika; Singh, Varun Kumar] Banaras Hindu Univ, Inst Med Sci, Dept Neurol, Varanasi 221005, Uttar Pradesh, India.
C3 Banaras Hindu University (BHU)
RP Singh, VK (通讯作者), Banaras Hindu Univ, Inst Med Sci, Dept Neurol, Varanasi 221005, Uttar Pradesh, India.
EM mailurvarun@gmail.com
TC 0
Z9 0
PD SEP-OCT
PY 2021
VL 69
IS 5
BP 1234
EP 1240
DI 10.4103/0028-3886.329546
WC Neurosciences
ER

PT J
AU Pountney, L
Prince, L
Wong-Spracklen, V
Takon, I
Conium, J
AF Pountney, L.
Prince, L.
Wong-Spracklen, V

Takon, I
Conium, J.
TI Remote Consultations for Paediatric Epilepsy during COVID-19 Pandemic - Parent's Perspective
SO ANNALS OF NEUROLOGY
LA English
DT Meeting Abstract
DE Epilepsy; COVID-19; Equity; Diversity; Inclusion
TC 0
Z9 0
PD SEP
PY 2021
VL 90
SU 26
MA 231
BP S124
EP S125
WC Clinical Neurology; Neurosciences
ER

PT J
AU Andraus, M
Thorpe, J
Tai, XY
Ashby, S
Hallab, A
Ding, D
Dugan, P
Perucca, P
Costello, D
French, JA
O'Brien, TJ
Depondt, C
Andrade, DM
Sengupta, R
Delanty, N
Jette, N
Newton, CR
Brodie, MJ
Devinsky, O
Cross, JH
Li, LM
Silvado, C
Moura, L
Cosenza, H
Messina, JP
Hanna, J

Sander, JW
Sen, A
AF Andraus, Maria
Thorpe, Jennifer
Tai, Xin You
Ashby, Samantha
Hallab, Asma
Ding, Ding
Dugan, Patricia
Perucca, Piero
Costello, Daniel
French, Jacqueline A.
O'Brien, Terence J.
Depondt, Chantal
Andrade, Danielle M.
Sengupta, Robin
Delanty, Norman
Jette, Nathalie
Newton, Charles R.
Brodie, Martin J.
Devinsky, Orrin
Cross, J. Helen
Li, Li M.
Silvado, Carlos
Moura, Luis
Cosenza, Harvey
Messina, Jane P.
Hanna, Jane
Sander, Josemir W.
Sen, Arjune
CA COVID-19 Epilepsy COV-E Study Grp
TI Impact of the COVID-19 pandemic on people with epilepsy: Findings from the Brazilian arm of the COV-E study
SO EPILEPSY & BEHAVIOR
LA English
DT Article
DE Non-communicable disease; Seizures; SUDEP; Epilepsy; risk; Coronavirus
AB The COVID-19 pandemic has had an unprecedented impact on people and healthcare services. The disruption to chronic illnesses, such as epilepsy, may relate to several factors ranging from direct infection to secondary effects from healthcare reorganization and social distancing measures. Objectives: As part of the COVID-19 and Epilepsy (COV-E) global study, we ascertained the effects of COVID-19 on people with epilepsy in Brazil, based on their perspectives and those of their caregivers. We also evaluated the impact of COVID-19 on the care delivered to people with epilepsy by healthcare workers. Methods: We designed separate online surveys for people with epilepsy and their caregivers. A further survey for healthcare workers contained additional assessments of changes to working patterns, productivity, and concerns for

those with epilepsy under their care. The Brazilian arm of COV-E initially collected data from May to November 2020 during the country's first wave. We also examined national data to identify the Brazilian states with the highest COVID-19 incidence and related mortality. Lastly, we applied this geographic grouping to our data to explore whether local disease burden played a direct role in difficulties faced by people with epilepsy. Results: Two hundred and forty-one people returned the survey, 20% were individuals with epilepsy (n = 48); 22% were caregivers (n = 53), and 58% were healthcare workers (n = 140). Just under half (43%) of people with epilepsy reported health changes during the pandemic, including worsening seizure control, with specific issues related to stress and impaired mental health. Of respondents prescribed anti- seizure medication, 11% reported difficulty taking medication on time due to problems acquiring pre-scriptions and delayed or canceled medical appointments. Only a small proportion of respondents reported discussing significant epilepsy-related risks in the previous 12 months. Analysis of national COVID-19 data showed a higher disease burden in the states of Sao Paulo and Rio de Janeiro compared to Brazil as a whole. There were, however, no geographic differences observed in survey responses despite variability in the incidence of COVID-19. Conclusion: Our findings suggest that Brazilians with epilepsy have been adversely affected by COVID-19 by factors beyond infection or mortality. Mental health issues and the importance of optimal communi- cation are critical during these difficult times. Healthcare services need to find nuanced approaches and learn from shared international experiences to provide optimal care for people with epilepsy as the direct burden of COVID-19 improves in some countries. In contrast, others face resurgent waves of the pandemic. (c) 2021 Published by Elsevier Inc.
C1 [Andraus, Maria] Univ Fed Rio de Janeiro, Clementino Fraga Filho Univ Hosp, Fac Med, Dept Internal Med,Neurol Serv,Epilepsy Program, Rio De Janeiro, RJ, Brazil.

[Thorpe, Jennifer; Tai, Xin You; Newton, Charles R.; Sen, Arjune] John Radcliffe Hosp, NIHR Biomed Res Ctr, Nuffield Dept Clin Neurosci, Oxford Epilepsy Res Grp, Oxford OX3 9DU, England.

[Thorpe, Jennifer; Ashby, Samantha; Hanna, Jane] SUDEP Act, 18 Newbury St, Wantage OX12 8DA, Oxon, England.

[Hallab, Asma] Charite Univ Med Berlin, Campus Benjamin Franklin, Berlin, Germany.

[Hallab, Asma] Free Univ Berlin, Campus Benjamin Franklin, Berlin, Germany.

[Hallab, Asma] Humboldt Univ, Campus Benjamin Franklin, Berlin, Germany.

[Hallab, Asma] Berlin Inst Hlth, Dept Psychiat & Psychotherapy, Campus Benjamin Franklin, Berlin, Germany.

[Ding, Ding] Fudan Univ, Inst Neurol, Huashan Hosp, Shanghai, Peoples R China.

[Dugan, Patricia; French, Jacqueline A.; Devinsky, Orrin] NYU, Grossman Sch Med, Dept Neurol, New York, NY 10003 USA.

[Perucca, Piero; O'Brien, Terence J.] Monash Univ, Alfred Hosp, Cent Clin Sch, Dept Neurosci, Melbourne, Vic, Australia.

[Perucca, Piero; O'Brien, Terence J.] Univ Melbourne, Royal Melbourne Hosp, Dept Med, Melbourne, Vic, Australia.

[Perucca, Piero; O'Brien, Terence J.] Univ Melbourne, Royal Melbourne Hosp, Dept Neurol, Melbourne, Vic, Australia.

[Costello, Daniel] Univ Coll Cork, Cork Univ Hosp, Epilepsy Serv, Cork, Ireland.

[Costello, Daniel] Univ Coll Cork, Coll Med & Hlth, Cork, Ireland.

[Depondt, Chantal] Univ Libre Bruxelles, Dept Neurol, Hop Erasme, Brussels, Belgium.

[Andrade, Danielle M.] Univ Toronto, Toronto Western Hosp, Adult Epilepsy Genet Program, Toronto, ON, Canada.
[Sengupta, Robin] Inst Neurosci, Kolkata, India.
[Delanty, Norman] Beaumont Hosp, Dublin, Ireland.
[Delanty, Norman] Royal Coll Surgeons Ireland, FutureNeuro Res Ctr, Sch Pharm & Biomol Sci, Dublin, Ireland.
[Jette, Nathalie] Icahn Sch Med Mt Sinai, Dept Neurol, New York, NY 10029 USA.
[Newton, Charles R.] Univ Oxford, Univ Dept Psychiat, Oxford, England.
[Brodie, Martin J.] West Glasgow Ambulatory Care Hosp Yorkhill, Epilepsy Unit, Glasgow, Lanark, Scotland.
[Cross, J. Helen] UCL NIHR BRC Great Ormond St Inst Child Hlth, London, Scotland.
[Cross, J. Helen] Young Epilepsy, St Piers Lane, Dormansland RH7 6P, Lingfield, Scotland.
[Li, Li M.] Univ Estadual Campinas, Brazilian Inst Neurosci & Neurotechnol, Sch Med Sci, Dept Neurol, Campinas, SP, Brazil.
[Silvado, Carlos] Univ Fed Parana, Comprehens Epilepsy Program, EEG Epilepsy Unit, Hosp Clin, Curitiba, Parana, Brazil.
[Moura, Luis; Cosenza, Harvey] Univ Fed Rio de Janeiro, Fuzzy Log Lab Labfuzzy, Prod Engn Program, Coordinat Post Grad Engn Programs COPPE, Rio De Janeiro, RJ, Brazil.
[Cosenza, Harvey] Fluminense Fed Univ UFF, Sci & Technol Inst ICT, Dept Engn REG, Campus Rio das Ostras, Niteroi, RJ, Brazil.
[Messina, Jane P.] Univ Oxford, Sch Geog & Environm, Oxford, England.
[Sander, Josemir W.] UCL Queen Sq Inst Neurol, Queen Sq, London WC1N 3BG, England.
[Sander, Josemir W.] Chalfont Ctr Epilepsy, Gerrards Cross SL9 0RJ, England.
[Sander, Josemir W.] Stichting Epilepsie Instellingen Nederland SEIN, Heemstede, Netherlands.
[Messina, Jane P.] Univ Oxford, Oxford Sch Global & Area Studies, Oxford, England.
C3 Universidade Federal do Rio de Janeiro; League of European Research Universities - LERU; University of Oxford; Free University of Berlin; Humboldt University of Berlin; Charite Universitatsmedizin Berlin; Free University of Berlin; Humboldt University of Berlin; Charite Universitatsmedizin Berlin; Free University of Berlin; Humboldt University of Berlin; Charite Universitatsmedizin Berlin; Free University of Berlin; Humboldt University of Berlin; Charite Universitatsmedizin Berlin; Fudan University; New York University; Florey Institute of Neuroscience & Mental Health; Monash University; Royal Melbourne Hospital; University of Melbourne; Royal Melbourne Hospital; University of Melbourne; University College Cork; University College Cork; Universite Libre de Bruxelles; University of Toronto; University Health Network Toronto; Royal College of Surgeons - Ireland; Icahn School of Medicine at Mount Sinai; League of European Research Universities - LERU; University of Oxford; Universidade Estadual de Campinas; Universidade Federal do Parana; Universidade Federal do Rio de Janeiro; Universidade Federal Fluminense; League of European Research Universities - LERU; University of Oxford; University of London; University College London; League of European Research Universities - LERU; University of Oxford

RP Sen, A (通讯作者)，John Radcliffe Hosp, Dept Neurol, 3rd Floor,West Wing, Oxford OX3 9DU, England.
EM arjune.sen@ndcn.ox.ac.uk
TC 1
Z9 1
PD OCT
PY 2021
VL 123
AR 108261
DI 10.1016/j.yebeh.2021.108261
EA SEP 2021
WC Behavioral Sciences; Clinical Neurology; Psychiatry
ER

PT J
AU Karazniewicz-Lada, M
Glowka, AK
Mikulska, AA
Glowka, FK
AF Karazniewicz-Lada, Marta
Glowka, Anna K.
Mikulska, Aniceta A.
Glowka, Franciszek K.
TI Pharmacokinetic Drug-Drug Interactions among Antiepileptic Drugs, Including CBD, Drugs Used to Treat COVID-19 and Nutrients
SO INTERNATIONAL JOURNAL OF MOLECULAR SCIENCES
LA English
DT Review
DE antiepileptic drug; cannabidiol; pharmacokinetics; biotransformation; therapeutic levels; clinical interactions
ID PARTIAL-ONSET SEIZURES; VALPROIC ACID; CLINICAL PHARMACOKINETICS; ESLICARBAZEPINE ACETATE; ADULT PATIENTS; EPILEPSY; CARBAMAZEPINE; STIRIPENTOL; CANNABIDIOL; TOPIRAMATE
AB Anti-epileptic drugs (AEDs) are an important group of drugs of several generations, ranging from the oldest phenobarbital (1912) to the most recent cenobamate (2019). Cannabidiol (CBD) is increasingly used to treat epilepsy. The outbreak of the SARS-CoV-2 pandemic in 2019 created new challenges in the effective treatment of epilepsy in COVID-19 patients. The purpose of this review is to present data from the last few years on drug-drug interactions among of AEDs, as well as AEDs with other drugs, nutrients and food. Literature data was collected mainly in PubMed, as well as google base. The most important pharmacokinetic parameters of the chosen 29 AEDs, mechanism of action and clinical application, as well as their biotransformation, are presented. We pay a special attention to the new potential interactions of the applied first-generation AEDs (carbamazepine, oxcarbazepine, phenytoin, phenobarbital and primidone), on decreased concentration of some medications (atazanavir and remdesivir), or their compositions (darunavir/cobicistat and lopinavir/ritonavir) used in the treatment of COVID-19 patients. CBD interactions with AEDs are clearly defined. In addition,

nutrients, as well as diet, cause changes in pharmacokinetics of some AEDs. The understanding of the pharmacokinetic interactions of the AEDs seems to be important in effective management of epilepsy.
C1 [Karazniewicz-Lada, Marta; Mikulska, Aniceta A.; Glowka, Franciszek K.] Poznan Univ Med Sci, Dept Phys Pharm & Pharmacokinet, PL-60781 Poznan, Poland.
[Glowka, Anna K.] Poznan Univ Med Sci, Dept Bromatol, PL-60354 Poznan, Poland.
C3 Poznan University of Medical Sciences; Poznan University of Medical Sciences
RP Glowka, FK (通讯作者), Poznan Univ Med Sci, Dept Phys Pharm & Pharmacokinet, PL-60781 Poznan, Poland.
EM mkaraz@ump.edu.pl; aglowka@ump.edu.pl; amikulska@ump.edu.pl; glowka@ump.edu.pl
TC 1
Z9 1
PD SEP
PY 2021
VL 22
IS 17
AR 9582
DI 10.3390/ijms22179582
WC Biochemistry & Molecular Biology; Chemistry, Multidisciplinary
ER

PT J
AU Whitney, R
RamachandranNair, R
AF Whitney, Robyn
RamachandranNair, Rajesh
TI Prioritizing Seizure Safety and SUDEP Counseling in People With Epilepsy and Their Caregivers During the COVID-19 Pandemic
SO PEDIATRIC NEUROLOGY
LA English
DT Editorial Material
DE SUDEP; Seizure safety; Counseling; Epilepsy; COVID-19
C1 [Whitney, Robyn; RamachandranNair, Rajesh] McMaster Univ, Dept Paediat, Div Neurol, Comprehens Epilepsy Program, Hamilton, ON, Canada.
C3 McMaster University
RP Whitney, R (通讯作者), McMaster Univ, Dept Paediat, Div Neurol, Comprehens Epilepsy Program, Hamilton, ON, Canada.
EM whitner@mcmaster.ca
TC 1
Z9 1
PD OCT
PY 2021
VL 123
BP 102
EP 103

DI 10.1016/j.pediatrneurol.2021.04.006
EA SEP 2021
WC Clinical Neurology; Pediatrics
ER

PT J
AU Clayton, LM
   Balestrini, S
   Cross, JH
   Wilson, G
   Eldred, C
   Evans, H
   Koepp, MJ
   Sisodiya, SM
AF Clayton, Lisa M.
   Balestrini, Simona
   Cross, J. Helen
   Wilson, Galia
   Eldred, Claire
   Evans, Helen
   Koepp, Matthias J.
   Sisodiya, Sanjay M.
TI The impact of SARS-CoV-2 vaccination in Dravet syndrome: A UK survey
SO EPILEPSY & BEHAVIOR
LA English
DT Article
DE Dravet syndrome; Vaccine; COVD-19; SARS-CoV-2; Seizure; Side effect
ID SEIZURES; FEATURES
AB Background: The COVID-19 pandemic led to the urgent need for accelerated vaccine development. Approved vaccines have proved to be safe and well tolerated across millions of people in the general population. Dravet syndrome (DS) is a severe, early onset, developmental and epileptic encephalopathy. Vaccination is a precipitating factor for seizures. While there is no evidence that vaccine-precipitated seizures lead to adverse outcomes in people with DS, fear surrounding vaccination can remain for caregivers of people with DS, in some cases resulting in rejection of recommended vaccinations, leaving individuals more vulnerable to the relevant infections. A greater understanding of the safety profile of the severe acute respiratory syndrome coronavirus 2 (SARS-CoV-2) vaccination in this vulnerable group will help provide guidance for caregivers and clinicians when considering vaccination. Methods: A cross-sectional survey regarding COVID-19 and SARS-CoV-2 vaccine, in people with DS, was conducted by Dravet Syndrome UK (DSUK). Concomitantly, a review of individuals with DS who had recently received the SARS-CoV-2 vaccine, and who are resident at the Chalfont Centre for Epilepsy (CCE), or attend epilepsy clinics at the National Hospital for Neurology and Neurosurgery (NHNN), was undertaken. Results: Thirty-eight people completed the DSUK survey. Thirty-seven percent of caregivers reported being concerned about someone with DS receiving the SARS-CoV-2 vaccine; with some reporting that they would decline a vaccine when offered. Seventy-seven percent had not received any advice from a healthcare

professional about the SARS-CoV-2 vaccination. 18/38 were eligible for SARS-CoV-2 vaccination, of whom nine had received their first vaccine dose. Combining the results of the DSUK survey and the review of individuals monitored at CCE or NHNN, fifteen people with DS had received their first dose of the SARS-CoV-2 vaccine. 11/15 (73%) reported at least one side effect, the most common being fatigue (6/15; 40%) and fever (6/15; 40%). Three individuals (20%) reported an increase in seizure frequency after the first vaccine dose. No increase in seizure frequency or duration was reported after the second dose. Conclusion: Overall, these results suggest that SARS-CoV-2 vaccines are safe and well tolerated in individuals with DS, as they are in most people without DS. In most people with DS, SARS-CoV-2 vaccine does not appear to be associated with an increase in the frequency or duration of seizures, even in those who develop fever post-vaccination. Many caregivers are concerned about a person with DS receiving a SARSCoV-2 vaccine, with some reporting that they would decline a SARS-CoV-2 vaccine when offered. It is crucial that healthcare professionals are proactive in providing accurate information regarding the risks and benefits of vaccination in this population, given the potential for serious outcomes from infection. (c) 2021 Elsevier Inc. All rights reserved.
C1 [Clayton, Lisa M.; Balestrini, Simona; Koepp, Matthias J.; Sisodiya, Sanjay M.] UCL Queen Sq Inst Neurol, Dept Clin & Expt Epilepsy, Box 29,Queen, London WC1N 3BG, England.

[Clayton, Lisa M.; Balestrini, Simona; Koepp, Matthias J.; Sisodiya, Sanjay M.] Chalfont Ctr Epilepsy, Buckinghamshire SL9 0RJ, England.

[Balestrini, Simona] Meyer Children Hosp, Neurosci Dept, Viale Gaetano Pieraccini, I-2450139 Florence, Italy.

[Cross, J. Helen] UCL NIHR BRC Great Ormond St Inst Child Hlth, 30 Guilford St, London WC1N 1EH, England.

[Cross, J. Helen] Young Epilepsy, Lingfield RH7 6PW, England.

[Wilson, Galia; Eldred, Claire; Evans, Helen] Dravet Syndrome UK DSUK, Member Dravet Syndrome European Federat DSEF, Reg Char 1128289, POB 756, Chesterfield S43 9EB, England.

C3 University of London; University College London

RP Sisodiya, SM (通讯作者), UCL Queen Sq Inst Neurol, Dept Clin & Expt Epilepsy, Box 29,Queen, London WC1N 3BG, England.; Sisodiya, SM (通讯作者), Chalfont Ctr Epilepsy, Buckinghamshire SL9 0RJ, England.

EM Lisa.clayton@ucl.ac.uk; s.balestrini@ucl.ac.uk; h.cross@ucl.ac.uk; galia.w@dravet.org.uk; claire.e@dravet.org.uk; helen.e@dravet.org.uk; m.koepp@ucl.ac.uk; s.sisodiya@ucl.ac.uk

TC 3

Z9 3

PD NOV

PY 2021

VL 124

AR 108258

DI 10.1016/j.yebeh.2021.108258

EA SEP 2021

WC Behavioral Sciences; Clinical Neurology; Psychiatry

ER

PT J
AU Kubota, T
Kuroda, N
AF Kubota, Takafumi
Kuroda, Naoto
TI Association between telemedicine and incidence of status epilepticus during the COVID-19 pandemic
SO EPILEPSY & BEHAVIOR
LA English
DT Article
DE COVID-19; Telemedicine; Status epilepticus; Seizure; Epilepsy
ID RISK-FACTORS; EPILEPSY
AB Objective: We aimed to investigate the association between telemedicine and the incidence of status epilepticus (SE) in patients with epilepsy (PWE) during the coronavirus disease 2019 (COVID-19) pandemic using a large population database in the United States. Methods: We performed a retrospective analysis of a private, cloud-based healthcare platform (Explorys Inc., Cleveland, Ohio, USA). We compared each of the previously reported risk factors for SE, such as child, male, and refractory epilepsy, using the chi-square test or Fisher's exact test in two groups: PWE with SE or without SE. We determined whether telemedicine could be a risk factor for the incidence of SE using multivariate binary logistic regression analysis incorporating statistically significant variables in the chisquare test or Fisher's exact test ($p < 0.05$). Statistical significance was set at $p < 0.05$. Results: We identified 1600 PWE with SE and 61,700 PWE without SE from May 2020 to May 2021. The proportion of children, males, refractory epilepsy, and telemedicine was higher in PWE with SE than in PWE without SE (children: 21.9% vs. 17.7%, $p < 0.001$; male: 52.5% vs. 48.2%, $p = 0.001$; refractory epilepsy: 20.6% vs. 8.2%, $p < 0.001$; telemedicine: 42.5% vs. 23.6%, $p < 0.001$). The multivariate binary logistic regression model identified four significant variables as follows: child (odds ratio [OR], 1.32; 95% confidence interval [CI], 1.17-1.50), male (OR, 1.19; 95% CI, 1.07-1.31), refractory epilepsy (OR, 2.44; 95% CI, 2.15-2.77), and telemedicine (OR, 2.29; 95% CI, 2.07-2.54). Conclusion: Telemedicine might be associated with an increased risk of SE in PWE during the COVID-19 pandemic. (c) 2021 Elsevier Inc. All rights reserved.
C1 [Kubota, Takafumi] Case Western Reserve Univ, Univ Hosp Cleveland, Med Ctr, Dept Neurol, Cleveland, OH 44106 USA.
[Kubota, Takafumi] Tohoku Univ, Grad Sch Med, Dept Neurol, Sendai, Miyagi, Japan.
[Kuroda, Naoto] Wayne State Univ, Dept Pediat, Detroit, MI 48202 USA.
[Kuroda, Naoto] Tohoku Univ, Grad Sch Med, Dept Epileptol, Sendai, Miyagi, Japan.
C3 Case Western Reserve University; Case Western Reserve University Hospital; University Hospitals of Cleveland; Tohoku University; Wayne State University; Tohoku University
RP Kubota, T (通讯作者), Tohoku Univ, Grad Sch Med, Dept Neurol, Aoba Ku, 1-1 Seiryo Machi, Sendai, Miyagi 9808574, Japan.
EM takafumi.kubota.c7@tohoku.ac.jp
TC 5
Z9 5
PD NOV

PY 2021
VL 124
AR 108303
DI 10.1016/j.yebeh.2021.108303
EA SEP 2021
WC Behavioral Sciences; Clinical Neurology; Psychiatry
ER

PT J
AU Kuroda, N
Fujimoto, A
AF Kuroda, Naoto
Fujimoto, Ayataka
TI Response to: "Healthy brain-muscle interface in epilepsy and COVID-19: Increased muscle effort is the alternative"
SO EPILEPSY & BEHAVIOR
LA English
DT Letter
DE Epilepsy; seizure; COVID-19; Quality of life
C1 [Kuroda, Naoto] Wayne State Univ, Dept Pediat, Detroit, MI 48202 USA.
[Kuroda, Naoto] Tohoku Univ, Grad Sch Med, Dept Epileptol, Sendai, Miyagi, Japan.
[Fujimoto, Ayataka] Seirei Hamamatsu Gen Hosp, Comprehens Epilepsy Ctr, Hamamatsu, Shizuoka, Japan.
C3 Wayne State University; Tohoku University
RP Kuroda, N (通讯作者), Wayne State Univ, Childrens Hosp Michigan, Dept Pediat, 3901 Beaubien St, Detroit, MI 48201 USA.
EM naoto.kuroda@wayne.edu
TC 0
Z9 0
PD OCT
PY 2021
VL 123
AR 108301
DI 10.1016/j.yebeh.2021.108301
EA SEP 2021
WC Behavioral Sciences; Clinical Neurology; Psychiatry
ER

PT J
AU Kuroda, N
Kubota, T
AF Kuroda, Naoto
Kubota, Takafumi
TI Psychological impact of the COVID-19 pandemic for patients with epilepsy: A systematic review and meta-analysis
SO EPILEPSY & BEHAVIOR
LA English

DT Review
DE Epilepsy; COVID-19; Mental health; Depression; Novel coronavirus disease
ID DEPRESSION; ANXIETY; PEOPLE; HEALTH; PHQ-9
AB Objective: To investigate psychological comorbidities in patients with epilepsy during the coronavirus disease 2019 (COVID-19) pandemic.
Method: A systematic review and meta-analysis approach was used to comprehensively search MEDLINE, CENTRAL, EMBASE, and ClinicalTrials.gov databases for relevant studies. Studies that reported psycholog-ical stress in patients with epilepsy during the COVID-19 pandemic were included. Psychological comor-bidities were defined as anxiety, depression, and sleep disturbance. Pooled proportions of psychological comorbidities with 95% confidence intervals (CIs) were assessed using a random-effects model. The qual-ity of assessment for each study, heterogeneity between the studies, and publication bias were also eval-uated.
Results: A total of 28 studies with 7959 patients/caregivers were included in the meta-analysis. The pooled proportions of anxiety/worry, depression/bad mood, and sleep disturbance were 38.9% (95% CI: 31.3-46.7); I-2 = 97%; p < 0.01, 30.9% (95% CI: 23.3-38.9), I-2 = 97%; p < 0.01, and 36.5% (95% CI: 28.3-45.1), I-2 = 97%, p < 0.01, respectively.
Conclusion: Although the heterogeneity was high, our results showed a relatively high incidence of psy-chological comorbidities. Therefore, clinicians need to intervene early in the stress of patients with epilepsy to prevent worsening of stress, which can result in seizure worsening. (C) 2021 Elsevier Inc. All rights reserved.
C1 [Kuroda, Naoto] Wayne State Univ, Dept Pediat, 3901 Beaubien St, Detroit, MI 48201 USA.
[Kuroda, Naoto] Tohoku Univ, Grad Sch Med, Dept Epileptol, Sendai, Miyagi, Japan.
[Kubota, Takafumi] Tohoku Univ, Grad Sch Med, Dept Neurol, Sendai, Miyagi, Japan.
C3 Wayne State University; Tohoku University; Tohoku University
RP Kuroda, N (通讯作者), Wayne State Univ, Dept Pediat, 3901 Beaubien St, Detroit, MI 48201 USA.
EM naoto.kuroda@wayne.edu
TC 0
Z9 0
PD NOV
PY 2021
VL 124
AR 108340
DI 10.1016/j.yebeh.2021.108340
EA SEP 2021
WC Behavioral Sciences; Clinical Neurology; Psychiatry
ER

PT J
AU Wojewodka, G
Gulliford, MC
Ashworth, M
Richardson, MP
Ridsdale, L

AF Wojewodka, Gabriella
Gulliford, Martin C.
Ashworth, Mark
Richardson, Mark P.
Ridsdale, Leone
TI Epilepsy and mortality: a retrospective cohort analysis with a nested case-control study identifying causes and risk factors from primary care and linkage-derived data
SO BMJ OPEN
LA English
DT Article
DE epilepsy; epidemiology; risk factor; cause
ID PREMATURE MORTALITY; PSYCHIATRIC COMORBIDITY; SELF-MANAGEMENT; DATA RESOURCE; LONG-TERM; PEOPLE; DEATH; EMERGENCY; VALIDATION; QUALITY
AB Objectives People with epilepsy (PWE) have a higher mortality rate than the general population. Epilepsy-related deaths have increased despite all-cause mortality decreasing in the general population pre-COVID-19. We hypothesised that clinical and lifestyle factors may identify people more at risk. Design We used a retrospective cohort study to explore cause of death and a nested case-control study to identify risk factors. Setting We explored factors associated with mortality using primary care population data from 1 April 2004 to 31 March 2014. Data were obtained from the Clinical Practice Research Datalink which compiles anonymised patient data from primary care in the UK. Cause of death data was supplemented from the Office of National Statistics when available. Participants The analysis included 70 431 PWE, with 11 241 registered deaths. Results The number of deaths within the database increased by 69% between the first and last year of the study. Epilepsy was considered as a contributing cause in approximately 45% of deaths of PWE under 35. Factors associated with increased risk of death included attendance at emergency departments and/or emergency admissions (OR 3.48, 95% CI 3.19 to 3.80), antiepileptic drug (AED) polytherapy (2 AEDs: OR 1.60, 95% CI 1.51 to 1.71; 3 AEDs: OR 2.06, 95% CI 1.86 to 2.29; 4+AEDs: OR 2.62, 95% CI 2.23 to 3.08), status epilepticus (OR 2.78, 95% CI 1.64 to 4.71), depression (OR 1.67, 95% CI 1.57 to 1.76) and injuries (OR 1.54, 95% CI 1.43 to 1.67). No seizures in the prior year (OR 0.52, 95% CI 0.41 to 0.65). Conclusion Our results add to existing evidence that deaths in epilepsy are increasing. Future studies could focus on identifying PWE at high risk and addressing them with clinical interventions or better self-management. Identifying specific risk factors for younger people should be a priority as epilepsy may be a factor in close to half of deaths of PWE under 35 years of age.
C1 [Wojewodka, Gabriella; Ridsdale, Leone] Kings Coll London, Inst Psychiat Psychol & Neurosci, Dept Basic & Clin Neurosci, London, England.
[Gulliford, Martin C.; Ashworth, Mark] Kings Coll London, Sch Populat Hlth & Environm Sci, London, England.
[Gulliford, Martin C.] Guys & St Thomas NHS Fdn Trust, NIHR Biomed Res Ctr, London, England.
[Gulliford, Martin C.] Kings Coll London, London, England.
[Richardson, Mark P.] Kings Coll London, Inst Psychiat Psychol & Neurosci, London, England.
C3 University of London; King's College London; University of London;

King's College London; Guy's & St Thomas' NHS Foundation Trust;
   University of London; King's College London; University of London;
   King's College London
RP Wojewodka, G (通讯作者), Kings Coll London, Inst Psychiat Psychol & Neurosci, Dept Basic & Clin Neurosci, London, England.
EM gabriella.wojewodka@kcl.ac.uk
TC 0
Z9 0
PD OCT
PY 2021
VL 11
IS 10
AR e052841
DI 10.1136/bmjopen-2021-052841
WC Medicine, General & Internal
ER

PT J
AU Rahman, MH
   Rana, HK
   Peng, SL
   Kibria, MG
   Islam, MZ
   Mahmud, SMH
   Moni, MA
AF Rahman, Md Habibur
   Rana, Humayan Kabir
   Peng, Silong
   Kibria, Md Golam
   Islam, Md Zahidul
   Mahmud, S. M. Hasan
   Moni, Mohammad Ali
TI Bioinformatics and system biology approaches to identify
   pathophysiological impact of COVID-19 to the progression and severity of
   neurological diseases
SO COMPUTERS IN BIOLOGY AND MEDICINE
LA English
DT Article
DE Bioinformatics; Transcriptomic analysis; COVID-19; Neurological
   diseases; Pathways; Ontology; Proteins; Semantic similarity
ID MULTIPLE-SCLEROSIS; ALZHEIMERS-DISEASE; BRAIN INFLAMMATION; PATHWAY;
   ASSOCIATION; METABOLISM; MICROGLIA; CELLS; GEO; APP
AB The Coronavirus Disease 2019 (COVID-19) still tends to propagate and increase the occurrence of COVID-19 across the globe. The clinical and epidemiological analyses indicate the link between COVID-19 and Neurological Diseases (NDs) that drive the progression and severity of NDs. Elucidating why some patients with COVID-19 influence the progression of NDs and patients with NDs who are diagnosed with COVID-19 are becoming

increasingly sick, although others are not is unclear. In this research, we investigated how COVID-19 and ND interact and the impact of COVID-19 on the severity of NDs by performing transcriptomic analyses of COVID-19 and NDs samples by developing the pipeline of bioinformatics and network-based approaches. The transcriptomic study identified the contributing genes which are then filtered with cell signaling pathway, gene ontology, protein-protein interactions, transcription factor, and microRNA analysis. Identifying hub-proteins using protein-protein interactions leads to the identification of a therapeutic strategy. Additionally, the incorporation of comorbidity interactions score enhances the identification beyond simply detecting novel biological mechanisms involved in the pathophysiology of COVID-19 and its NDs comorbidities. By computing the semantic similarity between COVID-19 and each of the ND, we have found gene-based maximum semantic score between COVID-19 and Parkinson's disease, the minimum semantic score between COVID-19 and Multiple sclerosis. Similarly, we have found gene ontology-based maximum semantic score between COVID-19 and Huntington disease, minimum semantic score between COVID-19 and Epilepsy disease. Finally, we validated our findings using gold-standard databases and literature searches to determine which genes and pathways had previously been associated with COVID-19 and NDs.
C1 [Rahman, Md Habibur] Islamic Univ, Dept Comp Sci & Engn, Kushtia 7003, Bangladesh.
[Rana, Humayan Kabir] Green Univ Bangladesh, Dept Comp Sci & Engn, Dhaka, Bangladesh.
[Peng, Silong] Chinese Acad Sci, Univ Chinese Acad Sci, Inst Automat, Beijing 100190, Peoples R China.
[Kibria, Md Golam] Univ Calgary, Schulich Sch Engn, Dept Chem & Petr Engn, Calgary, AB, Canada.
[Islam, Md Zahidul] Nagoya Univ, Grad Sch Engn, Dept Elect, Nagoya, Aichi, Japan.
[Mahmud, S. M. Hasan] Amer Int Univ Bangladesh, Dept Comp Sci, Dhaka, Bangladesh.
[Moni, Mohammad Ali] Univ Queensland, Fac Hlth & Behav Sci, Sch Hlth & Rehabil Sci, St Lucia, Qld 4072, Australia.
C3 Islamic University; Chinese Academy of Sciences; Institute of Automation, CAS; University of Chinese Academy of Sciences, CAS; University of Calgary; Nagoya University; American International University Bangladesh (AIUB); University of Queensland
RP Moni, MA (通讯作者), Univ Queensland, Fac Hlth & Behav Sci, Sch Hlth & Rehabil Sci, St Lucia, Qld 4072, Australia.
EM m.moni@uq.edu.au
TC 0
Z9 0
PD NOV
PY 2021
VL 138
AR 104859
DI 10.1016/j.compbiomed.2021.104859
EA OCT 2021
WC Biology; Computer Science, Interdisciplinary Applications; Engineering, Biomedical; Mathematical & Computational Biology
ER

PT J
AU Vancini, RL
   Andrade, MS
   Knechtle, B
   Nikolaidis, PT
   de Lira, CAB
AF Vancini, Rodrigo Luiz
   Andrade, Marilia Santos
   Knechtle, Beat
   Nikolaidis, Pantelis Theodoros
   de Lira, Claudio Andre Barbosa
TI Healthy brain-muscle interface in epilepsy and COVID-19: Increased
   muscle effort is the alternative
SO EPILEPSY & BEHAVIOR
LA English
DT Letter
DE Epilepsy; seizure; COVID-19; physical exercise
C1 [Vancini, Rodrigo Luiz] Univ Fed Espirito Santo, Ctr Educ Fis & Desportos, Vitoria, ES, Brazil.
   [Andrade, Marilia Santos] Univ Fed Sao Paulo, Dept Fisiol, Sao Paulo, Brazil.
   [de Lira, Claudio Andre Barbosa] Univ Fed Goias, Setor Fisiol Humana & Exercicio, Lab Avaliacao Movimento Humano, Fac Educ Fis & Danca, Goiania, Go, Brazil.
   [Knechtle, Beat] Univ Zurich, St Gallen & Inst Primary Care, Medbase St Gallen Vadianplatz, Zurich, Switzerland.
   [Nikolaidis, Pantelis Theodoros] Univ West Attica, Sch Hlth & Caring Sci, Aigaleo, Greece.
C3 Universidade Federal do Espirito Santo; Universidade Federal de Sao
   Paulo (UNIFESP); Universidade Federal de Goias; League of European
   Research Universities - LERU; University of Zurich; University of West
   Attica
RP Vancini, RL (通讯作者), Univ Fed Espirito Santo UFES, Ctr Educ Fis & Desportos CEFD, Campus Univ,Av Fernando Ferrari 514, BR-29075810 Vitoria, ES, Brazil.
EM rodrigoluizvancini@gmail.com
TC 1
Z9 1
PD OCT
PY 2021
VL 123
AR 108267
WC Behavioral Sciences; Clinical Neurology; Psychiatry
ER

PT J
AU Teng, T
   Sareidaki, DE
   Chemaly, N
   Bar, C

Coste-Zeitoun, D
Kuchenbuch, M
Nabbout, R
AF Teng, T.
Sareidaki, D. E.
Chemaly, N.
Bar, C.
Coste-Zeitoun, D.
Kuchenbuch, M.
Nabbout, R.
TI Physician and patient satisfaction with the switch to remote outpatient encounters in epilepsy clinics during the Covid-19 pandemic
SO SEIZURE-EUROPEAN JOURNAL OF EPILEPSY
LA English
DT Article
DE Telemedicine; EEG; Children; neurology; Follow-up
ID TELEMEDICINE; FEASIBILITY; IMPACT; CARE
AB Purpose: Analyzing parents' and physicians' opinions regarding phone-based encounters in emergency shifts of a French pediatric epilepsy center compared to traditional face-to-face encounters during the first lockdown of the COVID-19 pandemic

Methods: Prospective monocentric study on remote encounters at Necker rare epilepsy reference center from March 20th, 2020 to April 23rd, 2020 due to lockdown measures. This study was conducted with a survey based on 5-point Likert scales (LS-2/2) designed for both parents and physicians. We compared first versus follow-up encounters as well as physicians' and parents' opinions.

Results: We had a total of 224 responses, among which 204 were completed by physicians (91%) and 173 (84,4%) by parents. Twenty five were first encounters (14,2%). Physicians pointed out the need for clinical examination (42.6%), mainly for first encounters (p=0.0004). Physicians rated the quality of communication lower (p=0.003) as their capacity to answer parents' questions (p=0.004). They were significantly less satisfied with remote encounters compared to parents (p<10(-4)). We identified six urgent (2.9%) and 50 semi-urgent (24%) situations requiring programming face-to-face encounter during or shortly after the lockdown.

Conclusion: Remote encounters could be a helpful practice for pediatric patients with epilepsy in emergency situations such as pandemics. It allowed the identification and prioritization of emergency situations. Physicians were less positive than parents. We raised the possible use of remote encounters in association to face-to-face encounters for routine follow-up of pediatric patients with epilepsy.
C1 [Teng, T.; Sareidaki, D. E.; Chemaly, N.; Bar, C.; Coste-Zeitoun, D.; Kuchenbuch, M.; Nabbout, R.] Hop Necker Enfants Malad, AP HP, Dept Pediat Neurol, Reference Ctr Rare Epilepsies, Paris, France.

[Teng, T.; Sareidaki, D. E.; Chemaly, N.; Bar, C.; Coste-Zeitoun, D.; Kuchenbuch, M.; Nabbout, R.] ERN EpiCARE, Paris, France.

[Kuchenbuch, M.; Nabbout, R.] Univ Paris, INSERM, Lab Translat Res Neurol Disorders, UMR 1163,Imagine Inst, Paris, France.
C3 Assistance Publique Hopitaux Paris (APHP); Hopital Universitaire Necker-Enfants Malades - APHP; UDICE-French Research Universities;

Universite de Paris; Institut National de la Sante et de la Recherche
Medicale (Inserm); UDICE-French Research Universities; Universite de
Paris
RP Nabbout, R (通讯作者), Necker Enfants Malades Hosp, Dept Pediat Neurol, Reference Ctr Rare Epilepsies, 149 Rue Sevres, F-75015 Paris, France.
EM rimanabbout@aphp.com
TC 2
Z9 2
PD OCT
PY 2021
VL 91
BP 60
EP 65
DI 10.1016/j.seizure.2021.05.013
WC Clinical Neurology; Neurosciences
ER

PT J
AU Qiao, S
Zhang, RR
Yang, TT
Wang, ZH
Fang, XQ
Fang, CY
Geng, JH
Zhang, DM
Qu, LX
Cao, LL
Han, T
Liu, XW
AF Qiao, Shan
Zhang, Ran-ran
Yang, Ting-ting
Wang, Zhi-hao
Fang, Xi-qin
Fang, Chun-yan
Geng, Jian-hong
Zhang, Dong-mei
Qu, Li-xin
Cao, Li-li
Han, Tao
Liu, Xue-wu
TI Attitudes to Being Vaccinated Against COVID-19: A Survey of People With
Epilepsy in China
SO FRONTIERS IN NEUROLOGY
LA English
DT Article

DE COVID-19; epilepsy; vaccine; vaccine hesitation; vaccination willingness
ID WILLINGNESS; SAFETY; EFFICACY
AB Objective: We conducted a survey to assess vaccination coverage, vaccination willingness, and variables associated with vaccination hesitancy to provide evidence on coronavirus disease (COVID-19) vaccination strategies.

Methods: This anonymous questionnaire study conducted a multicenter, cross-sectional survey of outpatients and inpatients with epilepsy (PWE) registered in epilepsy clinics, in 2021, in 10 hospitals in seven cities of Shandong Province.

Results: A total of 600 questionnaires were distributed, and 557 valid questionnaires were returned. A total of 130 people were vaccinated against COVID-19. Among 427 unvaccinated participants, 69.32% (296/427) were willing to receive the COVID-19 vaccine in the future, and the remaining 30.68% (131/427) were unwilling to receive vaccination. Most (89.9%) of the participants believed that the role of vaccination was crucial in response to the spread of COVID-19. A significant association was found between willingness to receive the COVID-19 vaccine and the following variables: age, marital status, level of education, occupation, residence, seizure type, and seizure control after antiepileptic drug therapy. It is noteworthy that education level, living in urban areas, and seizure freedom were significantly related to willingness to receive COVID-19 vaccination.

Conclusions: Vaccination is a key measure for the prevention and control of COVID-19, and most PWE are willing to be vaccinated. Vaccine safety, effectiveness, and accessibility are essential in combatting vaccine hesitation and increasing vaccination rates.
C1 [Qiao, Shan] Shandong Univ, Cheeloo Coll Med, Sch Basic Med Sci, Dept Med Genet, Jinan, Peoples R China.

[Qiao, Shan] Shandong First Med Univ, Affiliated Hosp 1, Dept Neurol, Jinan, Peoples R China.

[Qiao, Shan] Shandong Prov Qianfoshan Hosp, Jinan, Peoples R China.

[Zhang, Ran-ran; Yang, Ting-ting; Wang, Zhi-hao; Fang, Xi-qin; Cao, Li-li; Liu, Xue-wu] Shandong Univ, Dept Neurol, Qilu Hosp, Cheeloo Coll Med, Jinan, Peoples R China.

[Fang, Chun-yan] Zhucheng Peoples Hosp, Dept Neurol, Weifang, Peoples R China.

[Geng, Jian-hong] Weifang Med Coll, Affiliated Hosp, Dept Neurol, Weifang, Peoples R China.

[Zhang, Dong-mei] Linyi Peoples Hosp, Dept Neurol, Linyi, Shandong, Peoples R China.

[Qu, Li-xin] Dezhou Peoples Hosp, Dept Neurol, Dezhou, Peoples R China.

[Han, Tao] Shandong Univ, Shandong Prov Hosp, Dept Neurol, Jinan, Peoples R China.

[Liu, Xue-wu] Shandong Univ, Inst Epilepsy, Jinan, Peoples R China.

C3 Shandong University; Shandong First Medical University & Shandong Academy of Medical Sciences; Shandong First Medical University & Shandong Academy of Medical Sciences; Shandong University; Weifang Medical University; Shandong First Medical University & Shandong Academy of Medical Sciences; Shandong University; Shandong University

RP Liu, XW (通讯作者), Shandong Univ, Dept Neurol, Qilu Hosp, Cheeloo Coll Med, Jinan, Peoples R China.; Liu, XW (通讯作者), Shandong Univ, Inst Epilepsy, Jinan, Peoples R China.

EM snlxw1966@163.com
TC 0

Z9 0
PD OCT 5
PY 2021
VL 12
AR 743110
DI 10.3389/fneur.2021.743110
WC Clinical Neurology; Neurosciences
ER

PT J
AU Katyal, J
Rashid, H
Tripathi, M
Sood, M
AF Katyal, Jatinder
Rashid, Haroon
Tripathi, Manjari
Sood, Mamta
TI Prevalence of depression and suicidal ideation in persons with epilepsy during the COVID-19 pandemic: A longitudinal study from India
SO EPILEPSY & BEHAVIOR
LA English
DT Article
DE COVID-19; Seizure; Persons with epilepsy (PWE); Depression; Suicidal ideation; Mini International Neuropsychiatric; Interview (MINI); Antiepileptic drug
ID TELEPHONE; DISORDERS; DIAGNOSIS; ANXIETY; PEOPLE
AB Objectives: COVID-19 pandemic has disrupted healthcare services for chronic disorders such as epilepsy. In this study, the impact of COVID-19 pandemic on persons with epilepsy (PWE) with regard to their seizure control, depression status, and medication adherence was assessed. Methods: After ethical clearance, 449 PWE who had been previously evaluated for depression at All India Institute of Medical Sciences (AIIMS), New Delhi, India, were telephonically revaluated using Mini International Neuropsychiatric Interview and surveyed for source of medication and medication adherence over past 6 months. The prevalence and the association of depression, suicidality, and seizures during pandemic with different PWE variables were determined. Results: Out of 449 PWE, 70.6% responded. 19.9% were diagnosed positive for depression as per MINI while suicidal ideation was observed in 5.4%. Seventy six (23.9%) PWE reported seizures during pandemic. The incidence was greater in females, unemployed, previously uncontrolled epilepsy, polytherapy, altered use of medications, and depressed PWE. Seizure during pandemic, increased seizure frequency, previous history of depression, and altered use of medications were all significantly associated with depression during COVID-19 pandemic (2.6-95%CI, 1.45-4.73; 1.9-95%CI, 1.01-3.57; 8.8-95%CI, 4.54- 17.21; 2.9-95%CI, 1.19-7.24), and polytherapy (2.9-95%CI, 0.92-9.04), seizures during pandemic (3.9- 95%CI, 1.45-10.53) and previous history of depression and suicidality, were related with suicidal ideation. Conclusion: COVID-19 pandemic-induced disruptions can be detrimental for PWE, and restoring services to the

precovid levels as well as putting appropriate continuity plans in place for care of PWE should be a priority. (c) 2021 Elsevier Inc. All rights reserved.
C1 [Katyal, Jatinder; Rashid, Haroon] All India Inst Med Sci, Dept Pharmacol, Neuropharmacol Lab, New Delhi 110029, India.
[Tripathi, Manjari] All India Inst Med Sci, Dept Neurol, New Delhi 110029, India.
[Sood, Mamta] All India Inst Med Sci, Dept Psychiat, New Delhi 110029, India.
C3 All India Institute of Medical Sciences (AIIMS) New Delhi; All India Institute of Medical Sciences (AIIMS) New Delhi; All India Institute of Medical Sciences (AIIMS) New Delhi
RP Katyal, J (通讯作者), All India Inst Med Sci, Dept Pharmacol, New Delhi 110029, India.
EM jatinderkatyal.aiims@gmail.com
TC 1
Z9 1
PD NOV
PY 2021
VL 124
AR 108342
DI 10.1016/j.yebeh.2021.108342
EA OCT 2021
WC Behavioral Sciences; Clinical Neurology; Psychiatry
ER

PT J
AU Fasolato, D
Carlucci, V
Mozzetta, S
Cagnin, A
AF Fasolato, Davide
Carlucci, Valentina
Mozzetta, Stefano
Cagnin, Annachiara
TI Probable autoimmune encephalitis presenting with epilepsy, fever and ageusia: A possible COVID-19-related event?
SO JOURNAL OF THE NEUROLOGICAL SCIENCES
LA English
DT Meeting Abstract
DE encephalitis; epilepsy; COVID-19
CT 25th World Congress of Neurology (WCN)
CY OCT 03-07, 2021
CL Rome, ITALY
TC 0
Z9 0
PD OCT
PY 2021
VL 429
SU S
MA 118856

BP 35
EP 36
DI 10.1016/j.jns.2021.118856
EA OCT 2021
WC Clinical Neurology; Neurosciences
ER

PT J
AU Bannikova, V
   Odintsova, G
   Koloteva, A
AF Bannikova, Valentina
   Odintsova, Galina
   Koloteva, Anna
TI Epilepsy and COVID-19: Is infection correlated to an increased risk of
   seizure recurrence?
SO JOURNAL OF THE NEUROLOGICAL SCIENCES
LA English
DT Meeting Abstract
DE Epilepsy; COVID-19; infection; seizure; recurrence; risk
CT 25th World Congress of Neurology (WCN)
CY OCT 03-07, 2021
CL Rome, ITALY
C1 [Bannikova, Valentina] Pavlov First St Petersburg State Med Univ, Med Fac, St Petersburg, Russia.
   [Odintsova, Galina; Koloteva, Anna] Polenov Russian Neurosurg Res Inst, Minist Hlth Russian Federat, Branch federally Funded Inst, Almazov Natl Med Res Ctr,Epileptol, St Petersburg, Russia.
C3 Pavlov First Saint Petersburg State Medical University; Almazov National
   Medical Research Centre; Ministry of Health of the Russian Federation
TC 0
Z9 0
PD OCT
PY 2021
VL 429
SU S
MA 119113
BP 143
EP 143
DI 10.1016/j.jns.2021.119113
EA OCT 2021
WC Clinical Neurology; Neurosciences
ER

PT J
AU Odintsova, G
   Bannikova, V

Koloteva, A
   Nezdorovin, O
AF Odintsova, Galina
   Bannikova, Valentina
   Koloteva, Anna
   Nezdorovin, Oleg
TI COVID-19 and epilepsy: Disease or vaccination?
SO JOURNAL OF THE NEUROLOGICAL SCIENCES
LA English
DT Meeting Abstract
DE COVID-19; epilepsy; vaccine
CT 25th World Congress of Neurology (WCN)
CY OCT 03-07, 2021
CL Rome, ITALY
C1 [Odintsova, Galina; Bannikova, Valentina; Koloteva, Anna; Nezdorovin, Oleg] Almazov Natl Med Reseacher Ctr, Epileptol, St Petersburg, Russia.
TC 0
Z9 0
PD OCT
PY 2021
VL 429
SU S
MA 119121
BP 146
EP 146
DI 10.1016/j.jns.2021.119121
EA OCT 2021
WC Clinical Neurology; Neurosciences
ER

PT J
AU Choudhary, N
   Kharbanda, P
   Baishya, J
   Chakravarty, K
AF Choudhary, Neetu
   Kharbanda, Parampreet
   Baishya, Jitupam
   Chakravarty, Kamalesh
TI Effect of COVID-19 pandemic on people with epilepsy (PWE): Experience
   from a territory care centre from India
SO JOURNAL OF THE NEUROLOGICAL SCIENCES
LA English
DT Meeting Abstract
DE Effect; COVID-19; PWE; epilepsy
CT 25th World Congress of Neurology (WCN)
CY OCT 03-07, 2021

CL Rome, ITALY
C1 [Choudhary, Neetu; Kharbanda, Parampreet; Baishya, Jitupam; Chakravarty, Kamalesh] Postgrad Inst Med Educ & Res, Neurol, Chandigarh, India.
C3 Post Graduate Institute of Medical Education & Research (PGIMER),
   Chandigarh
TC 0
Z9 0
PD OCT
PY 2021
VL 429
SU S
MA 119128
BP 148
EP 149
DI 10.1016/j.jns.2021.119128
EA OCT 2021
WC Clinical Neurology; Neurosciences
ER

PT J
AU Chavez-Castillo, M
   Gratton, M
   Nouri, M
   Andrade, A
AF Chavez-Castillo, Melissa
   Gratton, Michelle
   Nouri, Maryam
   Andrade, Andrea
TI The impact of the COVID-19 pandemic on children and families undergoing
   epilepsy surgery
SO JOURNAL OF THE NEUROLOGICAL SCIENCES
LA English
DT Meeting Abstract
DE impact; COVID-19; children; epilepsy; surgery
CT 25th World Congress of Neurology (WCN)
CY OCT 03-07, 2021
CL Rome, ITALY
C1 [Chavez-Castillo, Melissa; Gratton, Michelle; Nouri, Maryam] Childrens Hosp, London Hlth Sci Ctr, Paediat Neurol, London, ON, Canada.
   [Andrade, Andrea] Western Univ, Schulich Sch Med & Dent, Paediat, London, ON, Canada.
C3 London Health Sciences Centre; Western University (University of Western
   Ontario); Western University (University of Western Ontario)
TC 0
Z9 0
PD OCT
PY 2021
VL 429

SU S
MA 119863
DI 10.1016/j.jns.2021.119863
EA OCT 2021
WC Clinical Neurology; Neurosciences
ER

PT J
AU Koloteva, A
   Odintsova, G
AF Koloteva, Anna
   Odintsova, Galina
TI Structural epilepsies in patients with coronavirus infection on
   admission to an urban emergency hospital (Pokrovskaya City Hospital, in
   St. Petersburg)
SO JOURNAL OF THE NEUROLOGICAL SCIENCES
LA English
DT Meeting Abstract
DE etiology; epilepsy; coronavirus; epilepsy; seizure
CT 25th World Congress of Neurology (WCN)
CY OCT 03-07, 2021
CL Rome, ITALY
C1 [Koloteva, Anna] Polenov Neurosurg Inst, Almazov Natl Res Ctr, St Petersburg, Russia.
   [Odintsova, Galina] Minist Hlth Russian Federat, Epileptol, Polenov Russian Neurosurg Res Inst, Branch Federally Funded Inst,Almazov Natl Med Res, St Petersburg, Russia.
C3 Ministry of Health of the Russian Federation
TC 0
Z9 0
PD OCT
PY 2021
VL 429
SU S
MA 119845
DI 10.1016/j.jns.2021.119845
EA OCT 2021
WC Clinical Neurology; Neurosciences
ER

PT J
AU Olivo, S
   Cheli, M
   Dinoto, A
   Stokelj, D
   Tomaselli, M
   Manganotti, P
AF Olivo, Sasha

Cheli, Marta
Dinoto, Alessandro
Stokelj, David
Tomaselli, Marinella
Manganotti, Paolo
TI Telemedicine during the SARS-COV-2 pandemic lockdown: Monitoring stress and quality of sleep in patients with epilepsy
SO JOURNAL OF THE NEUROLOGICAL SCIENCES
LA English
DT Meeting Abstract
DE Telemedicine; SARS-COV-2; stress; sleep; epilepsy
CT 25th World Congress of Neurology (WCN)
CY OCT 03-07, 2021
CL Rome, ITALY
C1 [Olivo, Sasha; Cheli, Marta; Dinoto, Alessandro; Stokelj, David; Tomaselli, Marinella; Manganotti, Paolo] Univ Trieste, Cattinara Univ Hosp, Dept Med Surg & Hlth Sci, Neurol Unit, Trieste, Italy.
C3 University of Trieste
TC 0
Z9 0
PD OCT
PY 2021
VL 429
SU S
MA 117845
DI 10.1016/j.jns.2021.117845
EA OCT 2021
WC Clinical Neurology; Neurosciences
ER

PT J
AU Asadi-Pooya, AA
Barzegar, Z
Sadeghian, S
Nezafat, A
Shahisavandi, M
Nabavizadeh, SA
AF Asadi-Pooya, Ali A.
Barzegar, Zohreh
Sadeghian, Saeid
Nezafat, Abdullah
Shahisavandi, Mina
Nabavizadeh, Seyed Ali
TI COVID-19 Vaccine Hesitancy Among Patients With Epilepsy or Other Chronic Conditions
SO DISASTER MEDICINE AND PUBLIC HEALTH PREPAREDNESS
LA English

DT Article; Early Access
DE coronavirus; COVID-19; epilepsy; hesitancy; vaccine
AB Objectives: The aim of this study was to investigate the opinions of different groups of people in Iran on their willingness to receive a coronavirus disease 2019 (COVID-19) vaccine. Methods: In this cross-sectional study, we surveyed a sample (based on consecutive referrals) of 5 groups of people in late 2020: a group of the general population from Shiraz (without a history of any chronic medical or psychiatric problems), patients with epilepsy, patients with diabetes mellitus (DM), patients with cardiac problems, and patients with psychiatric problems. The survey included 4 general questions and 3 COVID-19-specific questions. Results: A total of 582 people participated. In total, 66 (11.3%) people expressed that they were not willing to receive a COVID-19 vaccine. Psychiatric disorders (odds ratio [OR]: 3.15; 95% confidence interval [CI]: 1.31-7.60; P = 0.006) and male sex (OR: 2.10; 95% CI: 1.23-3.58; P = 0.010) were significantly associated with COVID-19 vaccine hesitancy. Conclusion: Vaccine hesitancy is a global issue. Patients with psychiatric disorders had the highest rate of vaccine hesitancy. Previous studies have shown that depression and anxiety are associated with a reduced adherence to the recommended medical advice. Why male sex is associated with vaccine hesitancy is not clear. Researchers should investigate the rates and the factors affecting the vaccine hesitancy in their corresponding communities.
C1 [Asadi-Pooya, Ali A.; Barzegar, Zohreh; Nezafat, Abdullah; Shahisavandi, Mina; Nabavizadeh, Seyed Ali] Shiraz Univ Med Sci, Epilepsy Res Ctr, Shiraz, Iran.
[Asadi-Pooya, Ali A.] Thomas Jefferson Univ, Jefferson Comprehens Epilepsy Ctr, Dept Neurol, Philadelphia, PA 19107 USA.
[Sadeghian, Saeid] Ahvaz Jundishapur Univ Med Sci, Dept Pediat Neurol, Golestan Med Educ & Res Ctr, Ahvaz, Iran.
C3 Shiraz University of Medical Science; Jefferson University; Ahvaz Jundishapur University of Medical Sciences (AJUMS)
RP Asadi-Pooya, AA (通讯作者), Shiraz Univ Med Sci, Epilepsy Res Ctr, Shiraz, Iran.; Asadi-Pooya, AA (通讯作者), Thomas Jefferson Univ, Jefferson Comprehens Epilepsy Ctr, Dept Neurol, Philadelphia, PA 19107 USA.
EM aliasadipooya@yahoo.com
TC 0
Z9 0
AR PII S1935789321003116
DI 10.1017/dmp.2021.311
EA OCT 2021
WC Public, Environmental & Occupational Health
ER

PT J
AU Sanchez-Larsen, A
Conde-Blanco, E
Viloria-Alebesque, A
Buendia, CSV
Oltra, TE
Alvarez-Noval, A

Aledo-Serrano, A
Martin-Garcia, R
Ramos-Araque, ME
Campos, D
Valle-Penacoba, G
Sierra-Gomez, A
De Ceballos-Cerrajeria, P
Agundez-Sarasola, M
Khawaja, M
Hampel, KG
Serra-Martinez, M
Arbos-Barber, C
Gomez-Ibanez, A
Villino-Boquete, R
Cabezudo-Garcia, P
Rodriguez-Lavado, I
Principe, A
Sopelana-Garay, D
AF Sanchez-Larsen, Alvaro
Conde-Blanco, Estefania
Viloria-Alebesque, Alejandro
Sanchez-Vizcaino Buendia, Cristina
Espinosa Oltra, Tatiana
Alvarez-Noval, Amanda
Aledo-Serrano, Angel
Martin-Garcia, Raquel
Ramos-Araque, Maria E.
Campos, Dulce
Valle-Penacoba, Gonzalo
Sierra-Gomez, Alicia
De Ceballos-Cerrajeria, Pablo
Agundez-Sarasola, Marta
Khawaja, Mariam
Hampel, Kevin G.
Serra-Martinez, Maria
Arbos-Barber, Clara
Gomez-Ibanez, Asier
Villino-Boquete, Rafael
Cabezudo-Garcia, Pablo
Rodriguez-Lavado, Ignacio
Principe, Alessandro
Sopelana-Garay, David
TI COVID-19 prevalence and mortality in people with epilepsy: A nation-wide multicenter study
SO EPILEPSY & BEHAVIOR
LA English
DT Article

DE COVID-19; Coronavirus; SARS-CoV-2; Prevalence; Epilepsy; Seizure
ID ILAE COMMISSION; POSITION PAPER; CLASSIFICATION
AB Background: To assess the prevalence, severity, and mortality of COVID-19 in people with epilepsy (PWE) and evaluate seizure control in PWE during and after COVID-19. Methods: Retrospective, observational, multicenter study conducted in 14 hospitals. Medical records of randomly selected PWE followed at neurology outpatient clinics were reviewed. Proportion of PWE with a positive test for SARS-CoV-2 during 2020 was calculated. Risk factors associated with COVID-19 and its morbimortality were evaluated. Results: 2751 PWE were included, mean age 48.8 years (18-99), 72.4% had focal epilepsy, and 35% were drug-refractory. COVID-19 prevalence in PWE was 5.53%, while in the Spanish population was 4.26%. Proportion of admissions to hospital, ICU, and deaths in PWE were 17.1%, 2%, and 4.61% of COVID-19 cases, while in Spanish population were 10.81%, 0.95%, and 2.57%, respectively. A severe form of COVID-19 occurred in 11.8%; dyslipidemia, institutionalization at long-term care facilities, intellectual disability, and older age were associated risk factors. Older age, hypertension, dyslipidemia, cardiac disease, and institutionalization were associated with mortality from COVID-19. Seizure control was stable in 90.1% of PWE during acute COVID-19, while 8.6% reported an increase in seizure frequency. During post-COVID-19 follow-up, 4.6% reported seizure control worsening.
C1 [Sanchez-Larsen, Alvaro; Sopelana-Garay, David] Albacete Univ Hosp Complex, Dept Neurol, C Hermanos Falco 37, E-02002 Albacete, Spain.
[Conde-Blanco, Estefania] Hosp Clin Barcelona, Inst Invest Biomed August Pi & Sunyer IDIBAPS, EpiCARE European Reference Network Epilepsy, Epilepsy Program,Dept Neurol, Barcelona, Spain.
[Viloria-Alebesque, Alejandro] Hosp Gen Def, Inst Invest Sanitaria IIS Aragon, Dept Neurol, Zaragoza, Spain.
[Sanchez-Vizcaino Buendia, Cristina; Espinosa Oltra, Tatiana] Cartagena Univ Hosp Complex, Dept Neurol, Murcia, Spain.
[Alvarez-Noval, Amanda] Ribera Povisa Hosp, Dept Neurol, Vigo, Spain.
[Aledo-Serrano, Angel] Ruber Int Hosp, Dept Neurol, Epilepsy Program, Madrid, Spain.
[Martin-Garcia, Raquel] Puerta de Hierro Univ Hosp, Dept Neurol, Madrid, Spain.
[Ramos-Araque, Maria E.] Univ Assistance Complex Salamanca, Inst Invest Biomed Salamanca IBSAL, Salamanca, Spain.
[Ramos-Araque, Maria E.; Sierra-Gomez, Alicia] Univ Assistance Complex Salamanca, Dept Neurol, Salamanca, Spain.
[Campos, Dulce; Valle-Penacoba, Gonzalo] Valladolid Univ Clin Hosp, Dept Neurol, Valladolid, Spain.
[De Ceballos-Cerrajeria, Pablo; Agundez-Sarasola, Marta] Urduliz Hosp, Dept Neurol, Bilbao, Spain.
[Khawaja, Mariam] Hosp Clin Barcelona, Dept Neurol, Epilepsy Program, Barcelona, Spain.
[Hampel, Kevin G.] La Fe Univ Hosp, Dept Neurol, Valencia, Spain.
[Serra-Martinez, Maria; Arbos-Barber, Clara] Son Llatzer Univ Hosp, Dept Neurol, Mallorca, Spain.
[Gomez-Ibanez, Asier] Clin Univ Navarra, Dept Neurol, Madrid, Spain.
[Villino-Boquete, Rafael] Clin Univ Navarra, Dept Neurol, Pamplona, Spain.
[Cabezudo-Garcia, Pablo; Rodriguez-Lavado, Ignacio] Reg Univ Hosp Malaga, Dept

Neurol, Inst Invest Biomed Malaga IBIMA, Malaga, Spain.
[Principe, Alessandro] Univ Pompeu Fabra UPF, Inst Hosp Mar Invest Med IMIM, Hosp Mar, Epilepsy Unit,Dept Neurol, Barcelona, Spain.
C3 League of European Research Universities - LERU; University of Barcelona; Hospital Clinic de Barcelona; IDIBAPS; Hospital Puerta de Hierro-Majadahonda; Consejo Superior de Investigaciones Cientificas (CSIC); CSIC-USAL - Instituto de Biologia Molecular y Celular del Cancer de Salamanca (IBMCC); League of European Research Universities - LERU; University of Barcelona; Hospital Clinic de Barcelona; Hospital Universitari Son Llatzer; University of Navarra; University of Navarra; Universidad de Malaga; Institut Hospital del Mar d'Investigacions Mediques (IMIM); Hospital del Mar; Pompeu Fabra University
RP Sanchez-Larsen, A (通讯作者), Albacete Univ Hosp Complex, Dept Neurol, C Hermanos Falco 37, E-02002 Albacete, Spain.
EM aa.sanchezlarsen@gmail.com
TC 1
Z9 1
PD DEC
PY 2021
VL 125
AR 108379
DI 10.1016/j.yebeh.2021.108379
EA OCT 2021
WC Behavioral Sciences; Clinical Neurology; Psychiatry
ER

PT J
AU Massoud, F
Ahmad, SF
Hassan, AM
Alexander, KJ
Al-Hashel, J
Arabi, M
AF Massoud, Fathi
Ahmad, Samar Farouk
Hassan, Ahmed Medhat
Alexander, K. J.
Al-Hashel, Jasem
Arabi, Maher
TI Safety and tolerability of the novel 2019 coronavirus disease (COVID-19) vaccines among people with epilepsy (PwE): A cross-sectional study
SO SEIZURE-EUROPEAN JOURNAL OF EPILEPSY
LA English
DT Article
DE Epilepsy; COVID-19; Vaccine; Safety; people with epilepsy
ID VACCINATION; PERTUSSIS; SEIZURES; HISTORY
AB Background: People with epilepsy (PwE) were concerned about the safety of the novel

2019 Coronavirus Disease (COVID-19) vaccines.
Objective: This study aimed to assess the side effects experienced by PwE following vaccination with COVID-19 vaccines and to identify the causes of vaccine hesitation.
Methods: We administered a questionnaire to PwE, who visited the epilepsy clinic at Ibn Sina Hospital in Kuwait during the first two working weeks of April 2021. It included socio-demographic, epilepsy status, and vaccination data. In addition, we asked those who were not vaccinated yet about the reasons and their plan.
Results: A total of 111 PwE were surveyed, with 82 being vaccinated and 29 being unvaccinated. Out of the 82 vaccinated, 66 (80.5%) reported at least one side effect. Patients who received the Pfizer BioNTech mRNA vaccine (BNT162b2) (first, second dosage); and the Oxford-AstraZenecaa chimpanzee adenovirus-vectored vaccine (ChAdOx1nCoV-19) (first dose) had the following reactions: Pain at the injection site (40%, 67.6%), 43.8%, fatigue (47%, 32.4%), 46.9%, Headache (33.3%, 35.3%), 34.4% and Myalgia (40%, 35%), 50% respectively. Local site effects, including pain (67.6% vs. 40%, p = < 0.001) and redness (26.5% vs 6.7%, p = 0.019), were more statistically significantly after the second dose of BNT162b2 vaccine compared to the first dose of the same vaccine. While there was no significant difference in systemic side effects frequencies between the two doses of the BNT162b2 vaccine. The systemic side effects were more statistically significantly after the first dose of ChAdOx1nCoV-19 compared to the first dose of the BNT162b2 vaccine and those included fever (56.3% vs 13.3%, p = < 0.001), chills (37.5% vs 6.7%, p = < 0.001), myalgia (50% vs 40%, p = < 0.001) and arthralgia (25% vs 6.7%, p = 0.021). The local site reactions were not significantly different between the first doses of both vaccines. Among the subgroup who had vaccine-related side effects, 66.7% were females, 90.9% were 55 or younger, 63.6% were on polytherapy, 74% had side effects for one day or less, and 95% were symptoms free by the end of the first-week post-vaccination. Symptoms were mild in 68% of the patients and moderate in 29.3%. Most patients (93.9%) did not report seizure worsening after vaccination. The relative risk of seizure worsening after the first and second doses of BNT162b2 and the first dose of ChAdOx1nCoV-19 vaccines was 1.027 (95% CI 0.891-1.183), 1.019 (95% CI 0.928-1.119), and 1.026 (95% CI 0.929-1.134) respectively. After the first dose of BNT162b2, one patient reported the development of status epilepticus. Among the non-vaccinated group, 34.9% were still indecisive, while 37.9% rejected the vaccination. Fear of adverse effects (42.9%) and fear of epilepsy worsening (23.8%) were the main reasons for vaccine hesitation.
Conclusions: This study shows that the two vaccines under consideration (BNT162b2 and ChAdOx1nCoV-19) have a good safety profile and a low risk of epilepsy worsening among a cohort of PwE in Kuwait.
C1 [Massoud, Fathi; Ahmad, Samar Farouk; Hassan, Ahmed Medhat; Alexander, K. J.; Al-Hashel, Jasem; Arabi, Maher] Ibn Sina Hosp, Dept Neurol, Sabah Med Area, Kuwait, Kuwait.
[Ahmad, Samar Farouk] Minia Univ, Dept Neuropsychiat, Al Minya, Egypt.
[Al-Hashel, Jasem] Kuwait Univ, Hlth Sci Ctr, Dept Med, Jabriya, Kuwait.
C3 Egyptian Knowledge Bank (EKB); Minia University; Kuwait University
RP Massoud, F (通讯作者), Ibn Sina Hosp, Dept Neurol, Sabah Med Area, Kuwait, Kuwait.
EM dr_fathi2010@yahoo.com
TC 5
Z9 5

PD NOV
PY 2021
VL 92
BP 2
EP 9
DI 10.1016/j.seizure.2021.08.001
WC Clinical Neurology; Neurosciences
ER

PT J
AU Kurd, M
Hashavya, S
Benenson, S
Gilboa, T
AF Kurd, Mohammad
Hashavya, Saar
Benenson, Shmuel
Gilboa, Tal
TI Seizures as the main presenting manifestation of acute SARS-CoV-2 infection in children
SO SEIZURE-EUROPEAN JOURNAL OF EPILEPSY
LA English
DT Article
DE Seizure; Status epilepticus; COVID-19; SARS-CoV-2
ID STATUS EPILEPTICUS; COVID-19; COHORT
AB Objectives: To explore the rate, characteristics, risk factors, and prognosis of children presenting with seizures as the main symptom of acute COVID-19 (coronavirus disease 2019). Methods: We conducted a systematic retrospective study to identify all children who presented to the emergency departments of a tertiary academic medical center between March 1st and December 31st 2020 and had a SARSCoV-2 infection based on RT-PCR (reverse transcription-polymerase chain reaction) from nasopharyngeal swab. Clinical and demographic data were extracted from the electronic medical records and reviewed. Results: Total of 175 children were diagnosed with acute SARS-CoV-2 infection in the emergency departments during the study period. Of those, 11 presented with seizures. Age ranged from six months to 17 years and 4 were girls. Five presented with status epilepticus and responded to loading doses of anti-seizure medications. Six had fever. Seven had prior history of neurological disorder. Full recovery was the rule. Significance: Unlike in adults, seizures occur early and may be the main manifestation of acute COVID-19 in children. Seizures, including status epilepticus, may occur without fever even in children with no history of epilepsy and are not associated with severe disease. A high index of suspicion is required for early diagnosis thus infection control measures can be taken.
C1 [Kurd, Mohammad; Gilboa, Tal] Hadassah Med Ctr, Pediat Neurol Unit, IL-91200 Jerusalem, Israel.
[Hashavya, Saar; Benenson, Shmuel; Gilboa, Tal] Hebrew Univ Jerusalem, Sch Med, Jerusalem, Israel.
[Hashavya, Saar] Hadassah Med Ctr, Pediat Emergency Dept, Jerusalem, Israel.

[Benenson, Shmuel] Hadassah Med Ctr, Unit Infect Prevent & Control, Jerusalem, Israel.
C3 Hebrew University of Jerusalem; Hadassah University Medical Center;
Hebrew University of Jerusalem; Hebrew University of Jerusalem; Hadassah
University Medical Center; Hebrew University of Jerusalem; Hadassah
University Medical Center
RP Gilboa, T (通讯作者), Hadassah Med Ctr, Pediat Neurol Unit, IL-91200 Jerusalem, Israel.
EM talg@hadassah.org.il
TC 2
Z9 2
PD NOV
PY 2021
VL 92
BP 89
EP 93
DI 10.1016/j.seizure.2021.08.017
WC Clinical Neurology; Neurosciences
ER

PT J
AU Al Momani, M
Almomani, BA
Sweidan, P
Al-Qudah, A
Aburahma, S
Arafeh, Y
AF Al Momani, Miral
Almomani, Basima A.
Sweidan, Philip
Al-Qudah, Aladdin
Aburahma, Samah
Arafeh, Yusra
TI Impact of COVID-19 pandemic on pediatric patients with epilepsy in
Jordan: The caregiver perspective
SO SEIZURE-EUROPEAN JOURNAL OF EPILEPSY
LA English
DT Article
DE COVID-19; Pandemic; Epilepsy; Children; Seizure control
AB Objectives: The recent COVID-19 pandemic has disrupted care systems around the world. We assessed the impact of COVID-19 lockdown on the care of pediatric patients with epilepsy in Jordan. Potential predictors for seizure control during COVID-19 outbreaks were investigated.

Methods: A cross- sectional survey was conducted on pediatric patients with epilepsy in Jordan, between January and February 2021, via online questionnaires. The collected data included demographic information, epilepsyrelated characteristics, views of caregivers and changes in seizure control during COVID-19 outbreak.

Results: A total number of 672 subjects were screened, 276 were eligible, and 154 completed the questionnaire adequately. Two thirds of caregivers (66.2%) reported that the COVID -19 outbreaks prevented their child from getting proper epilepsy care and 28.6% reported difficulty giving the drugs to their child on time because of loss of daily routine. In addition, more than half (55.8%) reported difficulty obtaining antiseizure medicines (ASMs). On the other hand, 77.3% of caregivers reported that seizure status remained unchanged or improved for their children during the COVID-19 and 22.7% reported worsened seizure control. The number of antiseizure medicines taken by patients ($p < 0.001$), age ($p = 0.032$), residency area ($p = 0.013$) and the difficulty in giving the medicine during COVID-19 pandemic ($p = 0.002$) were the major factors influencing the seizure worsening experienced by patients.

Conclusion: Almost one of every five patients reported worsened seizure control during the outbreak of COVID-19 in Jordan. Moreover, two thirds of caregivers reported poor epilepsy care. This finding highlights the need to implement organized and efficient telemedicine programs devoted to epilepsy care.
C1 [Al Momani, Miral] Jordan Univ Sci & Technol, Fac Med, Dept Pediat & Neonatol, Irbid, Jordan.
[Almomani, Basima A.] Jordan Univ Sci & Technol, Fac Pharm, Dept Clin Pharm, Irbid, Jordan.
[Sweidan, Philip; Arafeh, Yusra] Jordan Univ Sci & Technol, Fac Med, Irbid, Jordan.
[Al-Qudah, Aladdin] Jordan Univ Sci & Technol, Fac Dent, Dept Conservat Dent, Irbid, Jordan.
[Aburahma, Samah] Jordan Univ Sci & Technol, Fac Med, Dept Neurosci, Irbid, Jordan.
C3 Jordan University of Science & Technology; Jordan University of Science & Technology; Jordan University of Science & Technology; Jordan University of Science & Technology; Jordan University of Science & Technology
RP Al Momani, M (通讯作者), Jordan Univ Sci & Technol, Fac Med, Dept Pediat & Neonatol, Irbid, Jordan.
EM maalmomani3@just.edu.jo; baalmomani1@just.edu.jo; philipsweidan@yahoo.com; aqudah@just.edu.jo; samahk@just.edu.jo; theyusraarafeh@gmail.com
TC 0
Z9 0
PD NOV
PY 2021
VL 92
BP 100
EP 105
DI 10.1016/j.seizure.2021.08.011
WC Clinical Neurology; Neurosciences
ER

PT J
AU Yeni, K
Tulek, Z
Ozer, A

Cavusoglu, A
Sirin, G
Baykan, B
Bebek, N
AF Yeni, Kubra
Tulek, Zeliha
Ozer, Arif
Cavusoglu, Aysel
Sirin, Gorkem
Baykan, Betul
Bebek, Nerses
TI The effect of fear of COVID-19 on quality of life in patients with
epilepsy
SO EPILEPSIA
LA English
DT Meeting Abstract
DE effect; fear; COVID-19; quality of life; epilepsy
CT 34th International Epilepsy Congress
CY AUG 28-SEP 01, 2021
CL ELECTR NETWORK
C1 [Yeni, Kubra] Ondokuz Mayis Univ, Fac Hlth Sci, Samsun, Turkey.
[Tulek, Zeliha] Istanbul Univ Cerrahpasa, Florence Nightingale Fac Nursing, Istanbul, Turkey.
[Ozer, Arif] Fac Educ, Dept Guidance & Psychol Counseling, Ankara, Turkey.
[Cavusoglu, Aysel; Sirin, Gorkem; Baykan, Betul; Bebek, Nerses] Istanbul Univ, Istanbul Fac Med, Istanbul, Turkey.
C3 Ondokuz Mayis University; Istanbul University - Cerrahpasa; Istanbul
University
TC 0
Z9 0
PD NOV
PY 2021
VL 62
SU 3
MA 471
BP 331
EP 331
WC Clinical Neurology
ER

PT J
AU Rider, F
Hauser, WA
Solomatin, Y
Yakovlev, A
Guekht, A
AF Rider, Flora

Hauser, W. Allen
   Solomatin, Yuri
   Yakovlev, Alexander
   Guekht, Alla
TI Incidence, severity and outcomes of COVID-19 in elderly people with
   epilepsy in Moscow: case-control study
SO EPILEPSIA
LA English
DT Meeting Abstract
DE Incidence; severity; outcomes; COVID-19; elderly people; epilepsy
CT 34th International Epilepsy Congress
CY AUG 28-SEP 01, 2021
CL ELECTR NETWORK
C1 [Rider, Flora; Solomatin, Yuri; Yakovlev, Alexander; Guekht, Alla] Moscow Res & Clin Ctr, Neuropsychiat Healthcare Dept, Moscow, Russia.
   [Hauser, W. Allen] Columbia Univ, Mailman Sch Publ Hlth, New York, NY USA.
   [Yakovlev, Alexander] Russian Acad Sci, Inst Higher Nervous Act & Neurophysiol, Moscow, Russia.
   [Guekht, Alla] Pirogov Russian Natl Res Med Univ, Moscow, Russia.
C3 Columbia University; Institute of Higher Nervous Activity &
   Neurophysiology of RAS; Russian Academy of Sciences; Pirogov Russian
   National Research Medical University
TC 0
Z9 0
PD NOV
PY 2021
VL 62
SU 3
MA 643
BP 5
EP 5
WC Clinical Neurology
ER

PT J
AU Yeni, K
   Tulek, Z
   Ozer, A
   Cavusoglu, A
   Sirin, G
   Baykan, B
   Bebek, N
AF Yeni, Kubra
   Tulek, Zeliha
   Ozer, Arif
   Cavusoglu, Aysel
   Sirin, Gorkem

Baykan, Betul
   Bebek, Nerses
TI COVID-19 pandemic in Istanbul: Seizure Frequency and Psychosocial
   Outcomes in Patients with Epilepsy
SO EPILEPSIA
LA English
DT Meeting Abstract
DE COVID-19; Seizure; Frequency; Psychosocial Outcomes; Epilepsy
CT 34th International Epilepsy Congress
CY AUG 28-SEP 01, 2021
CL ELECTR NETWORK
C1 [Yeni, Kubra] Ondokuz Mayis Univ, Fac Hlth Sci, Samsun, Turkey.
   [Tulek, Zeliha] Istanbul Univ Cerrahpasa, Florence Nightingale Fac Nursing, Istanbul, Turkey.
   [Ozer, Arif] Fac Educ, Dept Guidance & Psychol Counseling, Ankara, Turkey.
   [Cavusoglu, Aysel; Sirin, Gorkem; Baykan, Betul; Bebek, Nerses] Istanbul Univ, Istanbul Fac Med, Istanbul, Turkey.
C3 Ondokuz Mayis University; Istanbul University - Cerrahpasa; Istanbul
   University
TC 0
Z9 0
PD NOV
PY 2021
VL 62
SU 3
MA 496
BP 14
EP 15
WC Clinical Neurology
ER

PT J
AU Kaufman, M
   Fitzgerald, M
   Massey, S
   Fridinger, S
   Prelack, M
   Elis, C
   Ortiz-Gonzalez, X
   Fried, L
   DiGiovine, M
   Melamed, S
   Malcolm, M
   Banwell, B
   Stephenson, D
   Witzman, S
   Gonzalez, A

Dlugos, D
Kessler, S
Goldberg, E
Abend, N
Helbig, I
AF Kaufman, Michael
Fitzgerald, Mark
Massey, Shavonne
Fridinger, Sara
Prelack, Marisa
Elis, Colin
Ortiz-Gonzalez, Xilma
Fried, Lawrence
DiGiovine, Marissa
Melamed, Susan
Malcolm, Marissa
Banwell, Brenda
Stephenson, Donna
Witzman, Stephanie
Gonzalez, Alexander
Dlugos, Dennis
Kessler, Sudha
Goldberg, Ethan
Abend, Nicholas
Helbig, Ingo
TI Telemedicine, health disparities and seizure control in pediatric epilepsy during the COVID-19 pandemic
SO EPILEPSIA
LA English
DT Meeting Abstract
DE Telemedicine; seizure; epilepsy; COVID-19
CT 34th International Epilepsy Congress
CY AUG 28-SEP 01, 2021
CL ELECTR NETWORK
C1 [Kaufman, Michael; Fitzgerald, Mark; Massey, Shavonne; Fridinger, Sara; Prelack, Marisa; Elis, Colin; Ortiz-Gonzalez, Xilma; Fried, Lawrence; DiGiovine, Marissa; Melamed, Susan; Malcolm, Marissa; Banwell, Brenda; Stephenson, Donna; Witzman, Stephanie; Dlugos, Dennis; Kessler, Sudha; Goldberg, Ethan; Abend, Nicholas; Helbig, Ingo] Childrens Hosp Philadelphia, Div Neurol, Philadelphia, PA 19104 USA.
[Kaufman, Michael; Fitzgerald, Mark; Massey, Shavonne; Elis, Colin; Ortiz-Gonzalez, Xilma; Goldberg, Ethan; Helbig, Ingo] Childrens Hosp Philadelphia, Epilepsy NeuroGenet Initiat ENGIN, Philadelphia, PA 19104 USA.
[Kaufman, Michael; Gonzalez, Alexander; Helbig, Ingo] Childrens Hosp Philadelphia, Dept Biomed & Hlth Informat DBHi, Philadelphia, PA 19104 USA.
[Fitzgerald, Mark; Massey, Shavonne; Fridinger, Sara; Prelack, Marisa; Elis, Colin; Ortiz-Gonzalez, Xilma; Fried, Lawrence; DiGiovine, Marissa; Banwell, Brenda; Stephenson, Donna; Dlugos, Dennis; Kessler, Sudha; Goldberg, Ethan; Abend, Nicholas;

Helbig, Ingo] Univ Penn, Perelman Sch Med, Dept Neurol, Philadelphia, PA 19104 USA.
C3 University of Pennsylvania; Childrens Hospital of Philadelphia;
University of Pennsylvania; Childrens Hospital of Philadelphia;
University of Pennsylvania; Childrens Hospital of Philadelphia;
University of Pennsylvania
TC 0
Z9 0
PD NOV
PY 2021
VL 62
SU 3
MA 1163
BP 25
EP 25
WC Clinical Neurology
ER

PT J
AU Joseph, S
Valdes, LDI
Sibat, HF
AF Joseph, Sibi
Valdes, Lourdes de Fatima Ibanez
Sibat, Humberto Foyaca
TI Management of epilepsy secondary to neurocysticercosis during the
coronavirus pandemic-a novel approach
SO EPILEPSIA
LA English
DT Meeting Abstract
DE Management; epilepsy; neurocysticercosis; COVID-19
CT 34th International Epilepsy Congress
CY AUG 28-SEP 01, 2021
CL ELECTR NETWORK
C1 [Joseph, Sibi; Valdes, Lourdes de Fatima Ibanez; Sibat, Humberto Foyaca] Walter Sisulu Univ, Nelson Mandela Acad Hosp, Mthatha, South Africa.
C3 University of Kwazulu Natal; Walter Sisulu University
TC 0
Z9 0
PD NOV
PY 2021
VL 62
SU 3
MA 248
BP 56
EP 56
WC Clinical Neurology
ER

PT J
AU Burd, S
   Bokitko, T
   Mironov, M
   Rubleva, Y
   Kukina, N
   Tairova, R
AF Burd, Sergey
   Bokitko, Tatyana
   Mironov, Mikhail
   Rubleva, Yulia
   Kukina, Nina
   Tairova, Raisa
TI The influence of the transferred new coronavirus infection (COVID-19) on
   the course of epilepsy and bioelectric activity of the brain
SO EPILEPSIA
LA English
DT Meeting Abstract
DE influence; COVID-19; course; epilepsy; brain
CT 34th International Epilepsy Congress
CY AUG 28-SEP 01, 2021
CL ELECTR NETWORK
C1 [Burd, Sergey; Bokitko, Tatyana; Mironov, Mikhail; Rubleva, Yulia; Kukina, Nina; Tairova, Raisa] Fed Med Biol Agcy, Fed State Budgetary Inst, Fed Ctr Brain Res & Neurotechnol, Moscow, Russia.
   [Burd, Sergey] Pirogov Russian Natl Res Med Univ, Minist Hlth Russian Federat, Fed State Autonomous Educ Inst Higher Educ, Moscow, Russia.
C3 Ministry of Health of the Russian Federation; Pirogov Russian National
   Research Medical University
TC 0
Z9 0
PD NOV
PY 2021
VL 62
SU 3
MA 810
BP 71
EP 71
WC Clinical Neurology
ER

PT J
AU Fagbemigun, R
   Benchluch, A
   Le, K
   Diaz, P

   Muratore, C
   Aniol, S
   Lewis, E
AF Fagbemigun, Richard
   Benchluch, Ashley
   Le, Kevin
   Diaz, Patrick
   Muratore, Christina
   Aniol, Stephanie
   Lewis, Evan
TI Innovating Epilepsy Care during the COVID-19 Pandemic: The Virtual Rapid
   Access Epilepsy Clinic
SO EPILEPSIA
LA English
DT Meeting Abstract
DE Epilepsy; Care; COVID-19
CT 34th International Epilepsy Congress
CY AUG 28-SEP 01, 2021
CL ELECTR NETWORK
C1 [Fagbemigun, Richard; Benchluch, Ashley; Le, Kevin; Diaz, Patrick; Lewis, Evan] Neurol Ctr Toronto, Toronto, ON, Canada.
   [Muratore, Christina; Aniol, Stephanie] Epilepsy Toronto, Toronto, ON, Canada.
TC 0
Z9 0
PD NOV
PY 2021
VL 62
SU 3
MA 1211
BP 79
EP 79
WC Clinical Neurology
ER

PT J
AU Arabi, M
   Farouk, SA
   Al Hashel, J
   Alexander, J
   Hassan, AM
   Abokalawa, F
AF Arabi, Maher
   Farouk, Samar Ahmad
   Al Hashel, Jassem
   Alexander, John
   Hassan, Ahmed Medhat
   Abokalawa, Fathi

TI Safety and tolerability of COVID-19 vaccine among Patients with Epilepsy
   (PWE) in a tertiary hospital in kuwait: A patient Survey
SO EPILEPSIA
LA English
DT Meeting Abstract
DE Safety; tolerability; COVID-19; vaccine; Patients with Epilepsy (PWE) ; EPILEPSY
CT 34th International Epilepsy Congress
CY AUG 28-SEP 01, 2021
CL ELECTR NETWORK
C1 [Arabi, Maher; Farouk, Samar Ahmad; Al Hashel, Jassem; Alexander, John; Hassan, Ahmed Medhat; Abokalawa, Fathi] Ibn Sina Hosp, Kuwait, Kuwait.
TC 0
Z9 0
PD NOV
PY 2021
VL 62
SU 3
MA 1270
BP 80
EP 80
WC Clinical Neurology
ER

PT J
AU Pejovic, A
   Ristic, A
   Vojvodic, N
   Kovacevic, M
   Djukic, T
   Bascarevic, V
   Sokic, D
AF Pejovic, Aleksa
   Ristic, Aleksandar
   Vojvodic, Nikola
   Kovacevic, Masa
   Djukic, Tijana
   Bascarevic, Vladimir
   Sokic, Dragoslav
TI COVID-19 infection in patients operated due to intractable epilepsy -
   very few reasons for serious consternation
SO EPILEPSIA
LA English
DT Meeting Abstract
DE COVID-19; infection; drug resistant epilepsy
CT 34th International Epilepsy Congress
CY AUG 28-SEP 01, 2021
CL ELECTR NETWORK

C1 [Pejovic, Aleksa; Ristic, Aleksandar; Vojvodic, Nikola; Kovacevic, Masa; Djukic, Tijana; Sokic, Dragoslav] Univ Belgrade, Neurol Clin, Clin Ctr Serbia, Fac Med, Belgrade, Serbia.
[Bascarevic, Vladimir] Univ Belgrade, Clin Neurosurg, Clin Ctr Serbia, Fac Med, Belgrade, Serbia.
C3 Clinical Centre of Serbia; University of Belgrade; Clinical Centre of Serbia; University of Belgrade
TC 0
Z9 0
PD NOV
PY 2021
VL 62
SU 3
MA 536
BP 190
EP 191
WC Clinical Neurology
ER

PT J
AU Chavez-Castillo, M
Gratton, MK
Nouri, M
Andrade, A
AF Chavez-Castillo, Melissa
Gratton, Michelle Kregel
Nouri, Maryam
Andrade, Andrea
TI The impact of the Covid-19 pandemic on children and families undergoing epilepsy surgery
SO EPILEPSIA
LA English
DT Meeting Abstract
DE impact; Covid-19; children; epilepsy; surgery
CT 34th International Epilepsy Congress
CY AUG 28-SEP 01, 2021
CL ELECTR NETWORK
C1 [Chavez-Castillo, Melissa; Gratton, Michelle Kregel; Nouri, Maryam; Andrade, Andrea] London Hlth Sci Ctr, Pediat Neurol, London, ON, Canada.
C3 London Health Sciences Centre
TC 0
Z9 0
PD NOV
PY 2021
VL 62
SU 3
MA 790

BP 315
EP 316
WC Clinical Neurology
ER

PT J
AU Budikayanti, A
   Indrawati, LA
   Wiratman, W
   Octaviana, F
AF Budikayanti, Astri
   Indrawati, Luh Ari
   Wiratman, Winnugroho
   Octaviana, Fitri
TI Incidence of status epilepticus in people with epilepsy during covid-19
   pandemic
SO EPILEPSIA
LA English
DT Meeting Abstract
DE Incidence; status epilepticus; epilepsy; covid-19; people with epilepsy
CT 34th International Epilepsy Congress
CY AUG 28-SEP 01, 2021
CL ELECTR NETWORK
C1 [Budikayanti, Astri; Indrawati, Luh Ari; Wiratman, Winnugroho; Octaviana, Fitri]
Univ Indonesia, Cipto Mangunkusumo Gen Hosp, Fac Med, Jakarta, Indonesia.
C3 University of Indonesia
TC 0
Z9 0
PD NOV
PY 2021
VL 62
SU 3
MA 835
BP 316
EP 317
WC Clinical Neurology
ER

PT J
AU Reka, E
   Xhelili, M
   Grabova, S
   Kruja, J
AF Reka, Eni
   Xhelili, Malbora
   Grabova, Serla
   Kruja, Jera

TI Epilepsy during the COVID-19 pandemic: Our Experience from a single
   center
SO EPILEPSIA
LA English
DT Meeting Abstract
DE epilepsy; covid-19
CT 34th International Epilepsy Congress
CY AUG 28-SEP 01, 2021
CL ELECTR NETWORK
C1 [Reka, Eni; Xhelili, Malbora; Kruja, Jera] Univ Med, Fac Med, Ttirana, Tirana, Albania.
   [Grabova, Serla; Kruja, Jera] UHC Mother Teresa, Tirana, Albania.
TC 0
Z9 0
PD NOV
PY 2021
VL 62
SU 3
MA 795
BP 316
EP 316
WC Clinical Neurology
ER

PT J
AU Pasca, L
   Zanaboni, MP
   Totaro, M
   Grumi, S
   Ballante, E
   Totaro, C
   Celario, M
   Varesio, C
   de Giorgis, V
AF Pasca, Ludovica
   Zanaboni, Martina Paola
   Totaro, Martina
   Grumi, Serena
   Ballante, Elena
   Totaro, Chiara
   Celario, Massimiliano
   Varesio, Costanza
   de Giorgis, Valentina
TI Rethinking epilepsy trajectories and needs during COVID-19 pandemic
SO EPILEPSIA
LA English
DT Meeting Abstract

DE trajectory; epilepsy; covid-19
CT 34th International Epilepsy Congress
CY AUG 28-SEP 01, 2021
CL ELECTR NETWORK
C1 [Pasca, Ludovica; Zanaboni, Martina Paola; Totaro, Martina; Grumi, Serena; Ballante, Elena; Totaro, Chiara; Celario, Massimiliano; Varesio, Costanza; de Giorgis, Valentina] Neurol Inst Fdn Casimiro Mondino, Child Neuropsychiat, Pavia, Italy.
[Pasca, Ludovica; Totaro, Martina; Celario, Massimiliano; Varesio, Costanza] Univ Pavia, Brain & Behav, Pavia, Italy.
[Ballante, Elena] Univ Pavia, Math, Pavia, Italy.
C3 University of Pavia; University of Pavia
TC 0
Z9 0
PD NOV
PY 2021
VL 62
SU 3
MA 915
BP 317
EP 317
WC Clinical Neurology
ER

PT J
AU Bein, N
Casales, F
Vargas, AG
Mannucci, NP
Cialdella, L
Consalvo, D
AF Bein, Natali
Casales, Federico
Garcia Vargas, Augusto
Piris Mannucci, Natalia
Cialdella, Lucia
Consalvo, Damian
TI Impact of COVID-19 pandemic on patients with epilepsy in Argentina
SO EPILEPSIA
LA English
DT Meeting Abstract
DE Impact; COVID-19; epilepsy
CT 34th International Epilepsy Congress
CY AUG 28-SEP 01, 2021
CL ELECTR NETWORK
C1 [Bein, Natali; Casales, Federico; Garcia Vargas, Augusto; Consalvo, Damian] Sanatorio Arcos, Neurol & Neurosurg Inst, Buenos Aires, DF, Argentina.
[Piris Mannucci, Natalia; Cialdella, Lucia; Consalvo, Damian] Ramos Mejia Hosp,

Neurol, Buenos Aires, DF, Argentina.
C3 Hospital Ramos Mejia; University of Buenos Aires
TC 0
Z9 0
PD NOV
PY 2021
VL 62
SU 3
MA 936
BP 318
EP 318
WC Clinical Neurology
ER

PT J
AU Bintoro, AC
Husna, M
Khosama, H
Octaviana, F
Gunadharma, S
Kustiowati, E
Gelgel, AM
Kusumastuti, K
Budikayanti, A
AF Bintoro, Aris Catur
Husna, Machlusil
Khosama, Herlyani
Octaviana, Fitri
Gunadharma, Suryani
Kustiowati, Endang
Gelgel, Anna Marita
Kusumastuti, Kurnia
Budikayanti, Astri
TI Indonesian neurologist understanding and experience in epilepsy care
during covid-19 pandemic
SO EPILEPSIA
LA English
DT Meeting Abstract
DE epilepsy; care; covid-19
CT 34th International Epilepsy Congress
CY AUG 28-SEP 01, 2021
CL ELECTR NETWORK
C1 [Bintoro, Aris Catur; Kustiowati, Endang] Univ Diponegoro, Dr Kariadi Gen Hosp, Fac Med, Semarang, Indonesia.
[Husna, Machlusil] Univ Brawijaya, Dr Saiful Anwar Gen Hosp, Fac Med, Malang, Indonesia.
[Khosama, Herlyani] Univ Sam Ratulangi, Prof Dr RD Kandou Gen Hosp, Fac Med, Manado,

Indonesia.
[Octaviana, Fitri; Budikayanti, Astri] Univ Indonesia, Cipto Mangunkusumo Gen Hosp, Fac Med, Jakarta, Indonesia.
[Gunadharma, Suryani] Univ Padjadjaran, Dr Hasan Sadikin Gen Hosp, Fac Med, Bandung, Indonesia.
[Gelgel, Anna Marita] Univ Udayana, Sanglah Gen Hosp, Fac Med, Bali, Indonesia.
[Kusumastuti, Kurnia] Univ Airlangga, Dr Soetomo Gen Hosp, Fac Med, Surabaya, Indonesia.
C3 Diponegoro University; Brawijaya University; Universitas Sam Ratulangi; University of Indonesia; Dr Hasan Sadikin General Hospital; Universitas Padjadjaran; Sanglah General Hospital; Universitas Udayana; Airlangga University; Dr Soetomo General Hospital Surabaya
TC 0
Z9 0
PD NOV
PY 2021
VL 62
SU 3
MA 965
BP 318
EP 319
WC Clinical Neurology
ER

PT J
AU Correale, C
Cappelletti, S
Falamesca, C
Tondo, I
Nicoli, MS
Santato, F
De Palma, L
Vigevano, F
Specchio, N
AF Correale, Cinzia
Cappelletti, Simona
Falamesca, Chiara
Tondo, Ilaria
Nicoli, Maria Sole
Santato, Francesca
De Palma, Luca
Vigevano, Federico
Specchio, Nicola
TI Pediatric patients with epilepsy showed elevate rate of anxious depressive symptoms during COVID-19 pandemic: Preliminary findings of monocentric a cross-sectional study
SO EPILEPSIA

LA English
DT Meeting Abstract
DE epilepsy; depression; COVID-19; cross-sectional study
CT 34th International Epilepsy Congress
CY AUG 28-SEP 01, 2021
CL ELECTR NETWORK
C1 [Correale, Cinzia; Cappelletti, Simona; Falamesca, Chiara; Tondo, Ilaria; Nicoli, Maria Sole; Santato, Francesca; De Palma, Luca; Vigevano, Federico; Specchio, Nicola] Bambino Gesu Pediat Hosp, Rome, Italy.
C3 IRCCS Bambino Gesu
TC 0
Z9 0
PD NOV
PY 2021
VL 62
SU 3
MA 1135
BP 319
EP 319
WC Clinical Neurology
ER

PT J
AU Villagomez, EMG
   Santos-Peyret, A
   Thompson, A
AF Gonzalez Villagomez, Emilia Montserrat
   Santos-Peyret, Andrea
   Thompson, Arnold
TI Attitude towards Telemedicine use in patients with epilepsy during the
   pandemic COVID-19 in Mexico, according to a survey
SO EPILEPSIA
LA English
DT Meeting Abstract
DE Telemedicine; epilepsy; COVID-19
CT 34th International Epilepsy Congress
CY AUG 28-SEP 01, 2021
CL ELECTR NETWORK
C1 [Gonzalez Villagomez, Emilia Montserrat; Santos-Peyret, Andrea; Thompson, Arnold] Natl Inst Neurol & Neurosurg, Epilepsy Clin, Mexico City, DF, Mexico.
TC 0
Z9 0
PD NOV
PY 2021
VL 62
SU 3
MA 1222

BP 320
EP 321
WC Clinical Neurology
ER

PT J
AU de Carvalho, RM
   Lunardi, MDS
   Carneiro, RAVD
   Alessi, R
   Giacomini, F
   Lin, K
   Valente, K
AF de Carvalho, Rachel Marin
   Lunardi, Mariana dos Santos
   Veiga Domingues Carneiro, Raquel Alencastro
   Alessi, Ruda
   Giacomini, Felipe
   Lin, Katia
   Valente, Kette
TI Depressive symptoms in patients with epilepsy during COVID-19 pandemic
   and its correlation with adequate medical care
SO EPILEPSIA
LA English
DT Meeting Abstract
DE Depression; epilepsy; COVID-19; care
CT 34th International Epilepsy Congress
CY AUG 28-SEP 01, 2021
CL ELECTR NETWORK
C1 [de Carvalho, Rachel Marin; Valente, Kette] Univ Sao Paulo, Fac Med, Sao Paulo, Brazil.
   [Lunardi, Mariana dos Santos; Veiga Domingues Carneiro, Raquel Alencastro; Giacomini, Felipe; Lin, Katia] Univ Fed Santa Catarina, Neurol & Neurophysiol, Florianopolis, SC, Brazil.
   [Alessi, Ruda] Fac Med ABC, Neurol, Santo Andre, SP, Brazil.
C3 Universidade de Sao Paulo; Universidade Federal de Santa Catarina
   (UFSC); Faculdade de Medicina do ABC
TC 0
Z9 0
PD NOV
PY 2021
VL 62
SU 3
MA 590
BP 324
EP 324
WC Clinical Neurology

ER

PT J
AU Lu, L
   Xiong, WX
   Mu, J
   Zhang, Q
   Zhang, HS
   Zou, L
   Li, WM
   He, L
   Sander, JW
   Zhou, D
AF Lu, Lu
   Xiong, Weixi
   Mu, Jie
   Zhang, Qi
   Zhang, Hesheng
   Zou, Ling
   Li, Weimin
   He, Li
   Sander, Josemir W.
   Zhou, Dong
TI The impact of COVID-19 vaccines in patients with epilepsy: A review
SO EPILEPSIA
LA English
DT Meeting Abstract
DE impact; COVID-19; vaccine; epilepsy
CT 34th International Epilepsy Congress
CY AUG 28-SEP 01, 2021
CL ELECTR NETWORK
C1 [Lu, Lu; Xiong, Weixi; Mu, Jie; Zhang, Qi; Zhang, Hesheng; He, Li; Sander, Josemir W.; Zhou, Dong] Sichuan Univ, Dept Neurol, West China Hosp, Chengdu, Peoples R China.
   [Zou, Ling] Sichuan Univ, West China Hosp, Int Off, Dept Radiol, Chengdu, Peoples R China.
   [Li, Weimin] Sichuan Univ, West China Hosp, Dept Pulm & Crit Care Med, Chengdu, Peoples R China.
   [Sander, Josemir W.] Univ Coll London Hosp, Biomed Res Ctr, NIHR, Inst Neurol, UCL Queen Sq, London, England.
   [Sander, Josemir W.] Chalfont Ctr Epilepsy, Chalfont St Peter, London, England.
   [Sander, Josemir W.] Stichting Epilepsie Instellingen Nederland SEIN, Heemstede, Netherlands.
C3 Sichuan University; Sichuan University; Sichuan University; University
   College London Hospitals NHS Foundation Trust; University of London;
   King's College London; University College London
TC 0
Z9 0

PD NOV
PY 2021
VL 62
SU 3
MA 664
BP 332
EP 333
WC Clinical Neurology
ER

PT J
AU Lattanzi, S
   Leitinger, M
   Rocchi, C
   Salvemini, S
   Matricardi, S
   Brigo, F
   Meletti, S
   Trinka, E
AF Lattanzi, Simona
   Leitinger, Markus
   Rocchi, Chiara
   Salvemini, Sergio
   Matricardi, Sara
   Brigo, Francesco
   Meletti, Stefano
   Trinka, Eugen
TI Unraveling the enigma of new-onset refractory status epilepticus: a
   systematic review of aetiologies
SO EUROPEAN JOURNAL OF NEUROLOGY
LA English
DT Review
DE Febrile-infection-related epilepsy syndrome; infantile
   hemiconvulsion-hemiplegia and epilepsy syndrome; NORSE; seizure; status
   epilepticus
ID EPILEPSY SYNDROME FIRES; NMDA RECEPTOR ENCEPHALITIS; ACUTE
   ENCEPHALOPATHY; CASE SERIES; SEIZURES; NORSE; EPIDEMIOLOGY; ALCOHOLICS;
   MUTATIONS; COVID-19
AB Background and purpose New-onset refractory status epilepticus (NORSE) is a clinical presentation, neither a specific diagnosis nor a clinical entity. It refers to a patient without active epilepsy or other pre-existing relevant neurological disorder, with a NORSE without a clear acute or active structural, toxic or metabolic cause. This study reviews the currently available evidence about the aetiology of patients presenting with NORSE and NORSE-related conditions. Methods A systematic search was carried out for clinical trials, observational studies, case series and case reports including patients who presented with NORSE, febrile-infection-related epilepsy syndrome or the infantile hemiconvulsion-hemiplegia and epilepsy syndrome. Results Four hundred and

fifty records were initially identified, of which 197 were included in the review. The selected studies were retrospective case-control (n = 11), case series (n = 83) and case reports (n = 103) and overall described 1334 patients both of paediatric and adult age. Aetiology remains unexplained in about half of the cases, representing the so-called 'cryptogenic NORSE'. Amongst adult patients without cryptogenic NORSE, the most often identified cause is autoimmune encephalitis, either non-paraneoplastic or paraneoplastic. Infections are the prevalent aetiology of paediatric non-cryptogenic NORSE. Genetic and congenital disorders can have a causative role in NORSE, and toxic, vascular and degenerative conditions have also been described. Conclusions Far from being a unitary condition, NORSE is a heterogeneous and clinically challenging presentation. The development and dissemination of protocols and guidelines to standardize diagnostic work-up and guide therapeutic approaches should be implemented. Global cooperation and multicentre research represent priorities to improve the understanding of NORSE.
C1 [Lattanzi, Simona; Rocchi, Chiara; Salvemini, Sergio] Marche Polytech Univ, Dept Expt & Clin Med, Neurol Clin, Via Conca 71, I-60020 Ancona, Italy.
[Leitinger, Markus; Trinka, Eugen] Paracelsus Med Univ, Christian Doppler Univ Hosp, Ctr Cognit Neurosci, Dept Neurol, Salzburg, Austria.
[Leitinger, Markus; Trinka, Eugen] Paracelsus Med Univ, Christian Doppler Univ Hosp, Ctr Cognit Neurosci, Neurosci Inst, Salzburg, Austria.
[Matricardi, Sara] Childrens Hosp G Salesi, Dept Child Neuropsychiat, Ancona, Italy.
[Brigo, Francesco] Univ Verona, Dept Neurosci Biomed & Movement Sci, Verona, Italy.
[Brigo, Francesco] Franz Tappeiner Hosp, Div Neurol, Merano, BZ, Italy.
[Meletti, Stefano] AOU Modena, Neurol Unit, OCB Hosp, Modena, Italy.
[Meletti, Stefano] Univ Modena & Reggio Emilia, Ctr Neurosci & Neurotechnol, Dept Biomed Metab & Neural Sci, Modena, Italy.
[Trinka, Eugen] Univ Hlth Sci, Hlth Serv Res & HTA, Med Informat & Technol, Publ Hlth, Hall In Tirol, Austria.
C3 Marche Polytechnic University; Paracelsus Private Medical University; Paracelsus Private Medical University; University of Verona; Ospedale Franz Tappeiner; Universita di Modena e Reggio Emilia
RP Lattanzi, S (通讯作者), Marche Polytech Univ, Dept Expt & Clin Med, Neurol Clin, Via Conca 71, I-60020 Ancona, Italy.
EM alfierelattanzisimona@gmail.com
TC 1
Z9 1
PD FEB
PY 2022
VL 29
IS 2
BP 626
EP 647
DI 10.1111/ene.15149
EA NOV 2021
WC Clinical Neurology; Neurosciences
ER

PT J
AU Li, P
Lin, JH
Wu, CM
Huang, SS
Zhu, SQ
AF Li, Ping
Lin, Jiahe
Wu, Chunmei
Huang, Shanshan
Zhu, Suiqiang
TI The impact of social factors, especially psychological worries on anxiety and depression in patients with epilepsy
SO EPILEPSY & BEHAVIOR
LA English
DT Article
DE Epilepsy; Clinical anxiety; depression; Social variables; COVID-19
ID QUALITY-OF-LIFE; PSYCHIATRIC COMORBIDITY; HEALTH; COVID-19; PREVALENCE; ATTITUDES; PEOPLE; SAMPLE; RISK
AB Background: Social factors are believed to affect mental health in patients with epilepsy (PWE). However, there is still a lack of sufficient manifest proof, given the difficulty of exposing PWE to relatively consis-tent natural social environments with a low or high level of social interaction to study their significant role. Methods: This single-center, longitudinal study was conducted via online questionnaires during the coro-navirus disease 2019. PWE were recruited from downtown Wuhan and surrounding areas. The Patient Health Questionnaire-9 and Generalized Anxiety Disorder-7 were used to assess psychological status. Results: We analyzed 588 questionnaires completed by 294 PWE who participated in the dual survey. Under lockdown and reopening, the prevalence of anxiety was 13.6%/22.5%, and the prevalence of depres-sion was 19.4%/34.0%. Raising children and seizure-related characteristics, including uncontrolled sei-zures, seizure exacerbation, seizure frequency > 2/m, and changes in drug regimen, were risk factors in the first and second surveys. A high education level (OR =1.946, 95% CI = 1.191-3.182), low life satis-faction (OR =1.940, 95% CI = 1.007-3.737), worry about unanticipated seizures (OR = 2.147, 95% CI = 1.049-4.309), and worry about purchasing medication outside (OR = 2.063, 95% CI = 1.060-4.016) were risk factors for higher scores after reopening. Worry about unanticipated seizures (OR = 3.012, 95% CI = 1.302-6.965) and in-person medical consultation (OR = 2.319, 95% CI = 1.262-4.261) were related to newly diagnosed patients with psychological disorder after reopening. Conclusions: We identified an associatin between social variables and epileptic psychiatric comorbidities. (c) 2021 Elsevier Inc. All rights reserved.
C1 [Li, Ping; Lin, Jiahe; Wu, Chunmei; Huang, Shanshan; Zhu, Suiqiang] Huazhong Univ Sci & Technol, Tongji Hosp, Tongji Med Coll, Dept Neurol, 1095 Jiefang Ave, Wuhan, Peoples R China.
C3 Huazhong University of Science & Technology
RP Huang, SS; Zhu, SQ (通讯作者), Huazhong Univ Sci & Technol, Tongji Hosp, Tongji Med Coll, Dept Neurol, 1095 Jiefang Ave, Wuhan, Peoples R China.
EM shanahuang3@gmail.com; zhusuiqiang180616@163.com

TC 0
Z9 0
PD DEC
PY 2021
VL 125
AR 108376
DI 10.1016/j.yebeh.2021.108376
EA NOV 2021
WC Behavioral Sciences; Clinical Neurology; Psychiatry
ER

PT J
AU Samanta, D
Elumalai, V
Desai, VC
Hoyt, ML
AF Samanta, Debopam
Elumalai, Vimala
Desai, Vidya C.
Hoyt, Megan Leigh
TI Conceptualization and implementation of an interdisciplinary clinic for children with drug-resistant epilepsy during the COVID-19 pandemic
SO EPILEPSY & BEHAVIOR
LA English
DT Article
DE Epilepsy; Implementation Science; QI; Pediatrics; Quality; Schedule
ID INTRACTABLE EPILEPSY; IMPROVE QUALITY; UNITED-STATES; NO-SHOWS; CARE; SURGERY; TRENDS; AGE; US
AB Objective: To describe the rapid conceptualization and implementation of an interdisciplinary epilepsy clinic for children with drug-resistant epilepsy (DRE) at Arkansas Children's Hospital (ACH) during the COVID 19 pandemic. Methods: Focusing on care design and care coordination for children with DRE, multiple stakeholder groups decided to implement a clinic after the systematic rating of constructs present in a theoretical meta-analytic framework. Based on the projected success, the new interdisciplinary clinic (composed of an epileptologist, a neurosurgeon, and a neuropsychologist and coordinated by a full-time nurse) was established. Clinic operations were further refined through discussions with patients, families, and care providers. We collected data retrospectively (August 2020 to June 2021) to determine referral patterns, clinic scheduling metrics, patient characteristics, clinical recommendations, and epilepsy quality metrics. Results: Of the 32 Consolidated Framework for Implementation Research constructs assessed, 24 were positively rated to predict a high probability of successful implementation of the clinic. For approximately 100 patient visits, appearance and usage rates were >75%, yielding a clinic utilization rate of approximately 60%. Among 76 unique patients (average age of 12 years, 60% focal epilepsy), 39 patients (51.3%) were deemed eligible for epilepsy surgery evaluation. The majority of the patients (53.9%) were advised for additional diagnostic testing, and 31.6% of patients were scheduled for vagus nerve stimulation.

More patients (33%) had changes in their existing anti-seizure medication (ASM) regimen rather than an addition of a new ASM (7.9%). Standardized epilepsy quality measures showed >80% to 90% adherence in 3 (reproductive counseling, depression and anxiety screening, documentation of seizure frequency) out of 4 metrics. Significance: This is the first study to show that an interdisciplinary clinic can be a valuable attribute of care models in high-need children with DRE by enabling comprehensive one-stop service for diagnostic evaluation, surgical consideration, and brief assessment of psychiatric comorbidities without compromising consensus-based best practices. (c) 2021 Elsevier Inc. All rights reserved.
C1 [Samanta, Debopam] Univ Arkansas Med Sci, Dept Pediat, Div Neurol, Little Rock, AR USA.
[Elumalai, Vimala; Hoyt, Megan Leigh] Arkansas Childrens Hosp, Div Neurol, Little Rock, AR USA.
[Desai, Vidya C.] Univ Arkansas Med Sci, Coll Pharm, Little Rock, AR USA.
C3 University of Arkansas System; University of Arkansas Medical Sciences; Arkansas Children's Hospital; University of Arkansas System; University of Arkansas Medical Sciences
RP Samanta, D (通讯作者), 1 Childrens Way, Little Rock, AR 72202 USA.
EM dsamanta@uams.edu
TC 1
Z9 1
PD DEC
PY 2021
VL 125
AR 108403
DI 10.1016/j.yebeh.2021.108403
EA NOV 2021
WC Behavioral Sciences; Clinical Neurology; Psychiatry
ER

PT J
AU Tashakori-Miyanroudi, M
Souresrafil, A
Hashemi, P
Ehsanzadeh, SJ
Farrahizadeh, M
Behroozi, Z
AF Tashakori-Miyanroudi, Mahsa
Souresrafil, Aghdas
Hashemi, Paria
Ehsanzadeh, Seyed Jafar
Farrahizadeh, Maryam
Behroozi, Zahra
TI Prevalence of depression, anxiety, and psychological distress in patients with epilepsy during COVID-19: A systematic review
SO EPILEPSY & BEHAVIOR
LA English

DT Review
DE Epilepsy; COVID-19; Depression; Anxiety; Psychological distress
ID SLEEP-DEPRIVATION; PEOPLE; STRESS; IMPACT
AB Objective: COVID-19 pandemic disease has profound consequences for physical and mental health. In this regard, health care for chronic diseases, especially epilepsy is neglected The purpose of this systematic review study was to investigate the epidemic effect of COVID-19 on increasing the prevalence of mental disorders such as depression, anxiety, and sleep disorders in people with epilepsy (PWE). Methods: We systematically searched MEDLINE, Cochrane, Embase, Web of science, Scopus, and Psych info databases for studies that estimate the prevalence of mental disorders in PWE during the COVID19 until December 2020. Inclusion criteria included samples of population, with a confirmed diagnosis of epilepsy. Results: Irrespective of PWE or people without epilepsy (PWOE), all experienced stress and anxiety during COVID-19 pandemic. Most of the studies showed that PWE and even PWOE during the pandemic, suffer from depression. The highest rate of depression was attributed to female PWE with financial problems (66.7%) and the lowest rate of depression in PWE was reported in 8.6%. 7.1-71.2% and 28.2% of patients reported sleep disorders and insomnia, respectively. Less than 2% experienced a sleep improvement. Limitations: Due to a large amount of heterogeneities across the results, we could not evaluate the exact rate of prevalence in spite of using effective measures. Conclusions: People with epilepsy were considered as a susceptible group to the impact of the pandemic. Therefore, great attention should be paid to PWE and adequate psychological supports provided in this period to relieve or inhibit risks to mental health in PWE. (c) 2021 Elsevier Inc. All rights reserved.
C1 [Tashakori-Miyanroudi, Mahsa] Iran Univ Med Sci, Dept Physiol, Fac Med, Tehran, Iran.
[Souresrafil, Aghdas] Iran Univ Med Sci, Hlth Management & Econ Res Ctr, Tehran, Iran.
[Hashemi, Paria] Kurdistan Univ Med Sci, Res Inst Hlth Dev, Cellular & Mol Res Ctr, Sanandaj, Iran.
[Ehsanzadeh, Seyed Jafar] Iran Univ Med Sci, Sch Hlth Management & Informat Sci, English Language Dept, Tehran, Iran.
[Farrahizadeh, Maryam] Iran Univ Med Sci, Sch Adv Technol Med, Dept Neurosci, Tehran, Iran.
[Behroozi, Zahra] Iran Univ Med Sci, Dept Physiol, Fac Med, Tehran, Iran.
C3 Iran University of Medical Sciences; Iran University of Medical Sciences; Kurdistan University of Medical Sciences; Iran University of Medical Sciences; Iran University of Medical Sciences; Iran University of Medical Sciences
RP Behroozi, Z (通讯作者), Iran Univ Med Sci, Dept Med Physiol, Fac Med, Tehran, Iran.
EM behroozi_2010@yahoo.com
TC 2
Z9 2
PD DEC
PY 2021
VL 125
AR 108410
DI 10.1016/j.yebeh.2021.108410
EA NOV 2021

WC Behavioral Sciences; Clinical Neurology; Psychiatry
ER

PT J
AU Li, N
Chu, CJ
Lin, WH
AF Li, Nan
Chu, Chaojia
Lin, Weihong
TI A Survey of Hesitancy and Response to the COVID-19 Vaccine Among Patients With Epilepsy in Northeast China
SO FRONTIERS IN NEUROLOGY
LA English
DT Article
DE COVID-19; vaccine hesitancy; epilepsy; side effect; China
ID ILAE COMMISSION; DEFINITION
AB Objective: This study was conducted to investigate the hesitancy and response of people with epilepsy (PWE) to the coronavirus disease 2019 (COVID-19) vaccine.Methods: We conducted an online survey among PWE in northeast China about hesitancy and response to the COVID-19 vaccine. Their demographic background and symptomatic data about epilepsy were also recorded, and we analyzed the epilepsy-related risk factors in delaying the vaccine.Results: In total, 357 patients with confirmed epilepsy were included in the survey, and only 38 (11%) patients received the COVID-19 vaccine. Fear of aggravating epilepsy (58%, n = 185), discouragement from health workers for epilepsy (22%, n = 70), and fear of patients of other unknown serious side effects (13%, n = 42) were the main reasons for delaying vaccination. A higher seizure frequency was the only epilepsy-related risk factor in delaying the vaccine (OR = 1.104, 95% CI: 0.988-1.233). None of the vaccinated patients reported that the vaccine aggravated their epilepsy.Significance: Understanding concerns about the COVID-19 vaccine among PWE could help to improve health education and promote the establishment of an immune barrier.
C1 [Li, Nan; Chu, Chaojia; Lin, Weihong] First Hosp Jilin Univ, Dept Neurol, Changchun, Peoples R China.
C3 Jilin University
RP Lin, WH (通讯作者), First Hosp Jilin Univ, Dept Neurol, Changchun, Peoples R China.
EM linwh@jlu.edu.cn
TC 0
Z9 0
PD NOV 17
PY 2021
VL 12
AR 778618
DI 10.3389/fneur.2021.778618
WC Clinical Neurology; Neurosciences
ER

PT J
AU Lu, L
Zhang, Q
Xiao, J
Zhang, YY
Peng, W
Han, X
Chen, SL
Yang, D
Sander, JW
Zhou, D
Xiong, WX
AF Lu, Lu
Zhang, Qi
Xiao, Jing
Zhang, Yingying
Peng, Wei
Han, Xiong
Chen, Shengli
Yang, Dan
Sander, Josemir W.
Zhou, Dong
Xiong, Weixi
TI COVID-19 vaccine take-up rate and safety in adults with epilepsy: Data from a multicenter study in China
SO EPILEPSIA
LA English
DT Article
DE real-world investigation; SARS-CoV-2; seizure; vaccine
AB Objective This study was undertaken to investigate the COVID-19 vaccine uptake rate and possible postvaccination effects in adults with epilepsy. Methods We invited adults with epilepsy attending three centers in China from July 24 to August 31, 2021 to participate in this study. We also asked age- and sex-matched controls among people attending for other chronic neuropsychiatric conditions and healthy controls accompanying people with illness attending the hospitals to participate. We excluded people who, under the national guidelines, had evident contradictions to vaccination. Participants were interviewed face-to-face using questionnaires. Vaccine uptake and postvaccine adverse events among the people with epilepsy were compared with those with neuropsychiatric conditions and controls. We also compared the willingness and reasons for hesitancy among unvaccinated participants. Results We enrolled 981 people, of whom 491 had epilepsy, 217 had other neuropsychiatric conditions, and 273 were controls. Forty-two percent of those with epilepsy had had the first dose of a vaccine, compared with 93% of controls and 84% of the people with neuropsychiatric conditions ($p < .0001$). The majority (93.8%) of those immunized had inactivated vaccines. Among the unvaccinated people with epilepsy, 59.6% were willing to have the vaccine. Their main reasons for hesitation were potential adverse effects (53.3%) and concerns about losing seizure control (47.0%). The incidence of adverse events in the epilepsy group was

similar to controls. Nineteen people with epilepsy reported an increase in seizure frequency. No episode of status epilepticus or prolonged seizures was reported. Two controls had their first-ever seizure, which was unlikely related to the vaccine. Significance The vaccine uptake rate in people with epilepsy was lower than in their same-age controls. The postvaccination effect was no higher than in controls. We found no evidence suggesting worsening seizures after vaccination. Measurement and education focused on increasing the vaccination rate in epilepsy are warranted.
C1 [Lu, Lu; Zhang, Qi; Xiao, Jing; Zhang, Yingying; Peng, Wei; Sander, Josemir W.; Zhou, Dong; Xiong, Weixi] Sichuan Univ, West China Hosp, Dept Neurol, Chengdu 610041, Sichuan, Peoples R China.

[Lu, Lu; Zhang, Qi; Xiao, Jing; Zhang, Yingying; Peng, Wei; Sander, Josemir W.; Zhou, Dong; Xiong, Weixi] Sichuan Univ, Inst Brain Sci & Brain Inspired Technol West Chin, Chengdu 610041, Sichuan, Peoples R China.

[Han, Xiong] Zhengzhou Univ, Henan Prov Peoples Hosp, Dept Neurol, Peoples Hosp, Zhengzhou, Peoples R China.

[Chen, Shengli; Yang, Dan] Chongqing Univ Three Gorges Hosp, Dept Neurol, Chongqing, Peoples R China.

[Sander, Josemir W.] Univ Coll London Queen Sq, Biomed Res Ctr, Univ Coll London Hosp, Natl Inst Hlth Res,Inst Neurol, London, England.

[Sander, Josemir W.] Chalfont Ctr Epilepsy, Gerrards Cross, England.

[Sander, Josemir W.] Stichting Epilepsie Instellingen Nederland SEIN, Heemstede, Netherlands.

C3 Sichuan University; Sichuan University; Zhengzhou University; University College London Hospitals NHS Foundation Trust; University of London; University College London

RP Zhou, D; Xiong, WX (通讯作者), Sichuan Univ, West China Hosp, Dept Neurol, Chengdu 610041, Sichuan, Peoples R China.; Zhou, D; Xiong, WX (通讯作者), Sichuan Univ, Inst Brain Sci & Brain Inspired Technol West Chin, Chengdu 610041, Sichuan, Peoples R China.

EM zhoudong66@yahoo.de; 502216168@qq.com

TC 1

Z9 1

PD JAN

PY 2022

VL 63

IS 1

BP 244

EP 251

DI 10.1111/epi.17138

EA NOV 2021

WC Clinical Neurology

ER

PT J

AU Siahaan, YMT

Ketaren, RJ

Hartoyo, V

Hariyanto, TI

AF Siahaan, Yusak Mangara Tua
Ketaren, Retno Jayantri
Hartoyo, Vinson
Hariyanto, Timotius Ivan
TI Epilepsy and the risk of severe coronavirus disease 2019 outcomes: A systematic review, meta-analysis, and meta-regression
SO EPILEPSY & BEHAVIOR
LA English
DT Review
DE COVID-19; Epilepsy; Seizure; Neurology
ID COVID-19; SEIZURES
AB Background: Patients with epilepsy experience seizures, which have been reported to increase and worsen during the coronavirus disease (COVID-19) pandemic. However, the association between epilepsy and COVID-19 outcomes remains unclear. The aim of this study was to analyze whether patients with epilepsy have an increased risk of having poor COVID-19 outcomes.

Methods: We comprehensively evaluated potential articles extracted from the medRxiv, Europe PMC, and PubMed databases until June 30, 2021, using selected keywords. All published studies on epilepsy and COVID-19 were selected. We used the Review Manager 5.4 and Comprehensive Meta-Analysis 3 software for statistical analysis.

Results: Thirteen studies with 67,131 patients with COVID-19 were included in the analysis. Evaluation of the collated data revealed an association between epilepsy and increased severity of COVID-19 (OR, 1.69; 95%CI: 1.11-2.59; p = 0.010; I-2 = 29%; random-effect modeling) and mortality from COVID-19 (OR, 1.71; 95%CI: 1.14-2.56; p = 0.010; I-2 = 53%; random-effect modeling). The results also showed that the association between epilepsy and increased risk of developing severe COVID-19 is influenced by sex and neurodegenerative disease.

Conclusions: The findings of this study suggest that patients with epilepsy are at risk of having poor COVID-19 outcomes. Patients with epilepsy need special attention and should be prioritized for administration of the COVID-19 vaccine. (C) 2021 Elsevier Inc. All rights reserved.
C1 [Siahaan, Yusak Mangara Tua; Ketaren, Retno Jayantri; Hartoyo, Vinson] Pelita Harapan Univ, Fac Med, Dept Neurol, Karawaci 15811, Tangerang, Indonesia.
[Hariyanto, Timotius Ivan] Pelita Harapan Univ, Fac Med, Blvd Jendral Sudirman St, Karawaci 15811, Tangerang, Indonesia.
C3 Universitas Pelita Harapan; Universitas Pelita Harapan
RP Hariyanto, TI (通讯作者), Pelita Harapan Univ, Fac Med, Blvd Jendral Sudirman St, Karawaci 15811, Tangerang, Indonesia.
EM timotius.hariyanto95@gmail.com
TC 0
Z9 0
PD DEC
PY 2021
VL 125
AR 108437
DI 10.1016/j.yebeh.2021.108437
EA NOV 2021

WC Behavioral Sciences; Clinical Neurology; Psychiatry
ER

PT J
AU Antonazzo, IC
Fornari, C
Maumus-Robert, S
Cei, E
Paoletti, O
Conti, S
Cortesi, PA
Mantovani, LG
Gini, R
Mazzaglia, G
AF Antonazzo, Ippazio Cosimo
Fornari, Carla
Maumus-Robert, Sandy
Cei, Eleonora
Paoletti, Olga
Conti, Sara
Cortesi, Paolo Angelo
Mantovani, Lorenzo Giovanni
Gini, Rosa
Mazzaglia, Giampiero
TI Impact of COVID-19 Lockdown, during the Two Waves, on Drug Use and Emergency Department Access in People with Epilepsy: An Interrupted Time-Series Analysis
SO INTERNATIONAL JOURNAL OF ENVIRONMENTAL RESEARCH AND PUBLIC HEALTH
LA English
DT Article
DE COVID-19; epilepsy; antiepileptic drug; people with epilepsy; time-series analysis
ID STRESS; SEIZURES; INCREASE; CHILDREN; RISK
AB Background: In 2020, during the COVID-19 pandemic, Italy implemented two national lockdowns aimed at reducing virus transmission. We assessed whether these lockdowns affected anti-seizure medication (ASM) use and epilepsy-related access to emergency departments (ED) in the general population. Methods: We performed a population-based study using the healthcare administrative database of Tuscany. We defined the weekly time series of prevalence and incidence of ASM, along with the incidence of epilepsy-related ED access from 1 January 2018 to 27 December 2020 in the general population. An interrupted time-series analysis was used to assess the effect of lockdowns on the observed outcomes. Results: Compared to pre-lockdown, we observed a relevant reduction of ASM incidence (0.65; 95% Confidence Intervals: 0.59-0.72) and ED access (0.72; 0.64-0.82), and a slight decrease of ASM prevalence (0.95; 0.94-0.96). During the post-lockdown the ASM incidence reported higher values compared to pre-lockdown, whereas ASM prevalence and ED access remained lower. Results also indicate a lower impact of the second lockdown for both ASM prevalence (0.97; 0.96-0.98)

and incidence (0.89; 0.80-0.99). Conclusion: The lockdowns implemented during the COVID-19 outbreaks significantly affected ASM use and epilepsy-related ED access. The potential consequences of these phenomenon are still unknown, although an increased incidence of epilepsy-related symptoms after the first lockdown has been observed. These findings emphasize the need of ensuring continuous care of epileptic patients in stressful conditions such as the COVID-19 pandemic.
C1 [Antonazzo, Ippazio Cosimo; Fornari, Carla; Cei, Eleonora; Conti, Sara; Cortesi, Paolo Angelo; Mantovani, Lorenzo Giovanni; Mazzaglia, Giampiero] Univ Milano Bicocca, Res Ctr Publ Hlth CESP, I-20900 Monza, Italy.

[Maumus-Robert, Sandy] Univ Bordeaux, Bordeaux Populat Hlth Res Ctr, Team Pharmacoepidemiol, Inserm U1219, F-33000 Bordeaux, France.

[Paoletti, Olga; Gini, Rosa] Reg Agcy Healthcare Serv Tuscany, Epidemiol Unit, I-50141 Florence, Italy.

[Cortesi, Paolo Angelo; Mantovani, Lorenzo Giovanni] IRCCS MultiMed, Value Based Healthcare Unit, I-20099 Sesto San Giovanni, Italy.

C3 University of Milano-Bicocca; Institut National de la Sante et de la Recherche Medicale (Inserm); UDICE-French Research Universities; Universite de Bordeaux; IRCCS Multimedica

RP Fornari, C (通讯作者), Univ Milano Bicocca, Res Ctr Publ Hlth CESP, I-20900 Monza, Italy.

EM ippazio.antonazzo@unimib.it; carla.fornari@unimib.it; sandy.robert@u-bordeaux.fr; e.cei@campus.unimib.it; olga.paoletti@ars.toscana.it; sara.conti@unimib.it; paolo.cortesi@unimib.it; lorenzo.mantovani@unimib.it; rosa.gini@ars.toscana.it; giampiero.mazzaglia@unimib.it

TC 1
Z9 1
PD DEC
PY 2021
VL 18
IS 24
AR 13253
DI 10.3390/ijerph182413253
WC Environmental Sciences; Public, Environmental & Occupational Health
ER

PT J
AU Motoki, A
Akamatsu, N
Fumuro, T
Miyoshi, A
Tanaka, H
Hagiwara, K
Ohara, S
Kamada, T
Shigeto, H
Murai, H

AF Motoki, Ayako
Akamatsu, Naoki
Fumuro, Tomoyuki
Miyoshi, Ayako
Tanaka, Hideaki
Hagiwara, Koichi
Ohara, Shinji
Kamada, Takashi
Shigeto, Hiroshi
Murai, Hiroyuki
TI Comparison of Acute Withdrawal and Slow Taper of Antiseizure Medications during Video Electroencephalographic Monitoring: Efficacy for Shortening of Hospital Stay
SO JOURNAL OF CLINICAL MEDICINE
LA English
DT Article
DE epilepsy; video-EEG monitoring; drug withdrawal; dose tapering; COVID-19
ID SEIZURE CLUSTERS; ADVERSE EVENTS; EPILEPSY; SAFETY
AB Antiepileptic medications (ASMs) are withdrawn at the epilepsy monitoring unit to facilitate seizure recordings. The effect of rapid tapering of ASMs on the length of hospital stay has not been well documented. We compared the mean length of hospital stay between patients who underwent acute ASM withdrawal and slow dose tapering during long-term video electroencephalography (EEG) monitoring. We retrospectively investigated 57 consecutive patients admitted to the epilepsy monitoring unit regarding the mean length of hospital stay in the acute ASM withdrawal group (n = 30) and slow-taper group (n = 27). In the acute-withdrawal group, all ASMs were discontinued once the patients were admitted. In the slow-taper group, the doses of ASMs were gradually reduced by 15-30% daily. We also evaluated the safety of the acute-withdrawal and slow-taper protocols. The mean lengths of hospital stay were 3.8 +/- 1.92 and 5.2 +/- 0.69 days in the acute-withdrawal and slow-taper groups, respectively (p < 0.005). No severe adverse events, including status epilepticus, were observed. Acute ASM withdrawal has the advantage of significantly reducing the length of hospital stay over slow tapering, without any severe adverse effects.
C1 [Motoki, Ayako; Akamatsu, Naoki; Murai, Hiroyuki] Int Univ Hlth & Welf, Grad Sch Med, Dept Clin Med Sci, Tokyo 1078402, Japan.
[Akamatsu, Naoki; Miyoshi, Ayako; Tanaka, Hideaki; Hagiwara, Koichi; Ohara, Shinji; Kamada, Takashi; Shigeto, Hiroshi] Fukuoka Sanno Hosp, Epilepsy Ctr, Fukuoka 8140001, Japan.
[Akamatsu, Naoki; Murai, Hiroyuki] Int Univ Hlth & Welf, Sch Med, Dept Neurol, Narita 2868686, Japan.
[Fumuro, Tomoyuki] Int Univ Hlth & Welf, Sch Med Sci Okawa, Dept Lab Med, Okawa 3248501, Japan.
[Shigeto, Hiroshi] Kyushu Univ, Div Med Technol, Fukuoka 8190395, Japan.
C3 International University of Health & Welfare; International University of Health & Welfare; International University of Health & Welfare; Kyushu University
RP Akamatsu, N (通讯作者), Int Univ Hlth & Welf, Grad Sch Med, Dept Clin Med Sci, Tokyo

1078402, Japan.; Akamatsu, N (通讯作者), Fukuoka Sanno Hosp, Epilepsy Ctr, Fukuoka 8140001, Japan.; Akamatsu, N (通讯作者), Int Univ Hlth & Welf, Sch Med, Dept Neurol, Narita 2868686, Japan.
EM 18m3021@g.iuhw.ac.jp; akamatsu@iuhw.ac.jp; fumuro@iuhw.ac.jp;
aya.m@live.jp; hideaki08seven@gmail.com; hagiwarakyu@gmail.com;
shinji.ohara@gmail.com; kamada_takashi@kouhoukai.or.jp;
shigeto217@gmail.com; murai@iuhw.ac.jp
TC 0
Z9 0
PD DEC
PY 2021
VL 10
IS 24
AR 5972
DI 10.3390/jcm10245972
WC Medicine, General & Internal
ER

PT J
AU Bussieres, EL
Malboeuf-Hurtubise, C
Meilleur, A
Mastine, T
Herault, E
Chadi, N
Montreuil, M
Genereux, M
Camden, C
AF Bussieres, Eve-Line
Malboeuf-Hurtubise, Catherine
Meilleur, A.
Mastine, Trinity
Herault, Elodie
Chadi, Nicholas
Montreuil, Marjorie
Genereux, Melissa
Camden, Chantal
CA Team
TI Consequences of the COVID-19 Pandemic on Children's Mental Health: A Meta-Analysis
SO FRONTIERS IN PSYCHIATRY
LA English
DT Review
DE COVID-19; children; mental health; meta-analysis; impact
ID IMPACT; LOCKDOWN
AB Background: The COVID-19 pandemic has exacerbated mental health problems in many individuals, including children. Children with pre-existing socio-demographic or

developmental risk factors may be particularly vulnerable to the negative effects of the pandemic and associated public health preventive measures.
Objective: This systematic review and meta-analysis explored the impacts of the COVID-19 pandemic on the mental health of children aged 5-13 years-old, while highlighting the specific difficulties experienced by children with neurodevelopmental issues or chronic health conditions.
Methods: A systematic search of the published literature was conducted in Medline, ERIC, PsycINFO, and Google Scholar, followed by a quantitative meta-analysis of the eligible studies.
Results: Out of the 985 articles identified, 28 empirical studies with prospective or retrospective longitudinal data were included in the quantitative synthesis. COVID-19 lockdown measures were associated with negative general mental health outcomes among children ($g = 0.28$, $p < 0.001$, and $k = 21$), but of small magnitude. Sleep habits were also changed during the pandemic, as sleep duration significantly increased in children ($g = 0.32$; $p = 0.004$, and $k = 9$). Moreover, results did not differ between children from the general population and those from clinical populations such as children with epilepsy, oncology, neurodevelopmental disorders, or obesity. Effect sizes were larger in European vs. Asian countries.
Conclusions: Studies included in this review suggest that children's mental health was generally negatively impacted during the COVID-19 pandemic. More research is needed to understand the long-term effects of the COVID-19 pandemic on children's mental health and the influence of specific risks factors as they evolve over time.
C1 [Bussieres, Eve-Line] Univ Quebec Trois Rivieres, Dept Psychol, Trois Rivieres, PQ, Canada.
[Malboeuf-Hurtubise, Catherine; Mastine, Trinity] Bishops Univ, Dept Psychol, Sherbrooke, PQ, Canada.
[Meilleur, A.] Univ Quebec Montreal, Dept Psychol, Montreal, PQ, Canada.
[Herault, Elodie] Ctr Hosp Univ Sherbrooke, Ctr Rech, Sherbrooke, PQ, Canada.
[Chadi, Nicholas] Univ Montreal, St Justine Univ Hosp Ctr, Div Adolescent Med, Dept Pediat, Montreal, PQ, Canada.
[Montreuil, Marjorie] McGill Univ, Ingram Sch Nursing, Montreal, PQ, Canada.
[Genereux, Melissa; Camden, Chantal] Univ Sherbrooke, Dept Psychol, Sherbrooke, PQ, Canada.
C3 University of Quebec; University of Quebec Trois Rivieres; Bishops University; University of Quebec; University of Quebec Montreal; University of Sherbrooke; Universite de Montreal; McGill University; University of Sherbrooke
RP Bussieres, EL (通讯作者), Univ Quebec Trois Rivieres, Dept Psychol, Trois Rivieres, PQ, Canada.
EM eve-line.bussieres@uqtr.ca
TC 0
Z9 0
PD DEC 1
PY 2021
VL 12
AR 691659
DI 10.3389/fpsyt.2021.691659
WC Psychiatry

ER

PT J
AU Brown, ND
Dastjerdi, M
Herrmann, P
Loeb, J
Tang-Wai, R
Veltman, J
Losey, TE
AF Brown, Nikoli D.
Dastjerdi, Mohammad
Herrmann, Paul
Loeb, Joshua
Tang-Wai, Richard
Veltman, Jennifer
Losey, Travis E.
TI Elective inpatient video-EEG monitoring during the COVID-19 pandemic
SO EPILEPTIC DISORDERS
LA English
DT Article
DE COVID-19; SARS-CoV-2; epilepsy monitoring unit; EEG
ID EPILEPSY
AB Objective. To evaluate the safety and feasibility of admission for elective videoEEG monitoring during the SARS-CoV-2 pandemic. Methods. We performed a retrospective review of elective inpatient epilepsy monitoring unit admissions at our institution from May 3rd, 2020 to August 12th, 2020. All patients were screened by telephone for symptoms concerning infection or recent diagnosis of SARS-CoV-2 or excess medical risk prior to admission. Patients deemed eligible for admission underwent testing via a nasopharyngeal swab for SARS-CoV-2 within three days of admission, and were directed to self-quarantine between testing and admission. Results. The community seven-day case rate for SARS-CoV-2 (new cases per 100,000 population) ranged from 2.8 to 28.9 during the study period in our region. A total of 95 patients (63 adults and 32 children) were admitted. One adult patient developed mild SARS-CoV-2 infection and one adult patient tested positive for asymptomatic SARS-CoV-2 infection. Significance. These findings illustrate that inpatient epilepsy monitoring can be safely performed in carefully selected patients when appropriate processes are in place, even in the setting of the SARS-CoV-2 pandemic. There is a risk of nosocomial spread, and the potential benefits of admission should be balanced against the risks of infection.
C1 [Brown, Nikoli D.] Loma Linda Univ, Sch Med, Loma Linda, CA 92354 USA.
[Dastjerdi, Mohammad; Losey, Travis E.] Loma Linda Univ, Dept Neurol, 11175 Campus St CP 11108, Loma Linda, CA 92354 USA.
[Herrmann, Paul] Loma Linda Univ, Dept Pathol, Loma Linda, CA 92354 USA.
[Loeb, Joshua; Tang-Wai, Richard] Loma Linda Univ, Dept Pediat, Div Child Neurol, Loma Linda, CA 92354 USA.
[Veltman, Jennifer] Loma Linda Univ, Dept Internal Med, Div Infect Dis, Loma Linda, CA 92354 USA.

C3 Loma Linda University; Loma Linda University; Loma Linda University;
Loma Linda University; Loma Linda University
RP Losey, TE (通讯作者)，Loma Linda Univ, Dept Neurol, 11175 Campus St CP 11108, Loma Linda, CA 92354 USA.
EM tlosey@llu.edu
TC 0
Z9 0
PD DEC
PY 2021
VL 23
IS 6
BP 875
EP 878
DI 10.1684/epd.2021.1354
WC Clinical Neurology
ER

PT J
AU Sanabria-Sanchinel, AA
Escobar-Pineda, ES
Oliveros, I
Perdomo-Mendizabal, AL
Lara-Giron, JC
Vega-Zeissig, E
Leon-Aldana, JA
AF Sanabria-Sanchinel, Abel A.
Escobar-Pineda, Edwin S.
Oliveros, Isabel
Perdomo-Mendizabal, Ana L.
Lara-Giron, Juan C.
Vega-Zeissig, Eleonora
Leon-Aldana, Jorge A.
TI Telemedicine and epilepsy: healthcare experience of a national reference center during the COVID-19 pandemic
SO REVISTA DE NEUROLOGIA
LA Spanish
DT Article
DE COVID-19; Epilepsy; seizure; care; Pandemic; Telemedicine
AB Introduction. Countries worldwide are having to cope with the COVID-19 pandemic caused by SARS-CoV-2. The burden on their national health systems is currently at unprecedented levels. Telemedicine care was initiated at an early stage in our centre.

Patients and methods. We conducted a descriptive and retrospective study to evaluate the usefulness of telemedicine during lockdown in our centre. Patients included in the study had a clinical diagnosis of epilepsy, with two visits via telemedicine, who had been followed up for at least six months during the normal situation prior to the COVID-19 pandemic and two face-to-face consultations during the same period.

Results. A total of 115 patients were included. The average age was 29 years, 53%

were males, 52.2% had focal epilepsy, 58.3% with a structural causation and 57.4% had difficult-to-treat epilepsy. The mean number of seizures prior to lockdown was 9.73/month and 6.54/month during lockdown. The number of patients who were seizure-free when lockdown ended was higher than that observed in the phase before it began: 54 versus 45 out of 115.

Conclusions. Telemedicine is a very useful strategy for monitoring the course, progress and therapeutic changes in epileptic patients in the short and medium term. The reduction in the seizure frequency can be sustained in the medium term, not only in the short term as corroborated in previous studies. Telemedicine allows access to virtually all patients and closer monitoring.
C1 [Sanabria-Sanchinel, Abel A.; Escobar-Pineda, Edwin S.; Leon-Aldana, Jorge A.] Univ San Carlos, Ctr Univ Metropolitan, Fac Ciencias Med, Serv Neurol, Ciudad De Guatemala, Guatemala.

[Sanabria-Sanchinel, Abel A.; Leon-Aldana, Jorge A.] Univ San Carlos, Ctr Univ Metropolitan, Fac Ciencias Med, Serv Epileptol, Ciudad De Guatemala, Guatemala.

[Oliveros, Isabel] Univ San Carlos, Ctr Univ Metropolitan, Fac Ciencias Med, Serv Psiquiatria, Ciudad De Guatemala, Guatemala.

[Lara-Giron, Juan C.] Univ San Carlos, Ctr Univ Metropolitan, Fac Ciencias Med, Serv Neurocirugia & Cirugia Epilepsia, Ciudad De Guatemala, Guatemala.

[Vega-Zeissig, Eleonora] Univ San Carlos, Ctr Univ Metropolitan, Fac Ciencias Med,Serv Neurofisiol, Ctr Epilepsia & Neurocirugia Func Humana, Ciudad De Guatemala, Guatemala.

[Perdomo-Mendizabal, Ana L.] Univ San Carlos, Ctr Univ Metropolitan, Fac Ciencias Med, Unidad Invest, Ciudad De Guatemala, Guatemala.

C3 Universidad de San Carlos de Guatemala; Universidad de San Carlos de Guatemala; Universidad de San Carlos de Guatemala; Universidad de San Carlos de Guatemala; Universidad de San Carlos de Guatemala

RP Sanabria-Sanchinel, AA (通讯作者), Ctr Epilepsia & Neurocirugia Func Humana, 7a,Calle A 1-62 Zona 10, Ciudad De Guatemala 01010, Guatemala.

EM abelsanabria2000@yahoo.es
TC 0
Z9 0
PD DEC
PY 2021
VL 73
IS 11
BP 390
EP 393
DI 10.33588/rn.7311.2021324
WC Clinical Neurology
ER

PT J
AU Menon, S
Sander, JW
AF Menon, Sonia

Sander, Josemir W.
TI Effects of the COVID-19 pandemic on medication adherence: In the case of antiseizure medications, A scoping review
SO SEIZURE-EUROPEAN JOURNAL OF EPILEPSY
LA English
DT Review
DE Epilepsy; Antiepileptic drug; COVID-19; Chronic disease; management
ID SOMATIC COMORBIDITY; EPILEPSY; THERAPY; SAFE
AB Since early 2020, an unprecedented public global health emergency caused by coronavirus (COVID-19) resulted in national governments' imposing confinement measures. Lockdowns and isolation during pandemics complicate disease management and medication adherence. Chronic conditions, such as epilepsy, require linear adherence patterns to prevent breakthrough seizures and to reduce the risk of sudden unexpected death. Limited access to health care facilities for routine care and medicines management further hampers this. Social isolation exacerbates stress, depression and decreases social support, which may combine to reduce adherence to antiseizure medication (ASM) during the pandemic. Methods: We conducted a literature scoping review to explore ASM adherence among people with epilepsy, noninfected or infected SARS-CoV-2 or recovered from COVID-19 during the pandemic and explore risk factors for adherence. We search Pubmed for articles up to 16 September 2021. Search terms included the thematic of ASM adherence and COVID-19. We adhered to the PRISMA guidelines for reporting scoping reviews. Results: Six articles were retained after the screening, which covered four overarching themes: change of ASM compliance and as risk factors, lack of follow-up, difficulties accessing ASM, and behavioural risk factors. Our review underscores the lack of evidence on ASM adherence among people with epilepsy infected or recovered from COVID-19. No study retrieved took place in a low-income setting, warranting a cautionary approach to be employed when extrapolating findings on a global scale. Recommendations for practice: Missing information on past SARS-CoV2 infections impact people with epilepsy precludes exploring a direct effect of SARS-CoV2 on ASM adherence. A more comprehensive chronic disease model based on the burden of co-cardiovascular and neuro-behavioural comorbidities should be envisaged for this population in preparation for future pandemics. A monitoring algorithm needs to be in place to establish a telemedicine framework and community pharmacists' potential to contribute to the model recognised.
C1 [Menon, Sonia] Univ Paris, INSERM, Ctr Res Epidemiol & Stat CRESS, F-75004 Paris, France.
[Menon, Sonia] Cochrane France, F-75004 Paris, France.
[Sander, Josemir W.] Sichuan Univ, West China Hosp, Dept Neurol, Chengdu, Peoples R China.
[Sander, Josemir W.] NIHR Univ Coll London Hosp, Biomed Res Ctr, UCL Queen Sq Inst Neurol, London WC1N 3BG, England.
[Sander, Josemir W.] Chalfont Ctr Epilepsy, Gerrards Cross, Bucks, England.
[Sander, Josemir W.] Stichting Epilepsie Instellingen Nederland SEIN, Heemstede, Netherlands.
C3 Institut National de la Sante et de la Recherche Medicale (Inserm); UDICE-French Research Universities; Universite de Paris; Sichuan University; University College London Hospitals NHS Foundation Trust; University of London; University College London

RP Menon, S (通讯作者), Univ Paris, INSERM, Ctr Res Epidemiol & Stat CRESS, F-75004 Paris, France.
EM soniasimonemenon@gmail.com
TC 0
Z9 0
PD DEC
PY 2021
VL 93
BP 81
EP 87
DI 10.1016/j.seizure.2021.10.009
WC Clinical Neurology; Neurosciences
ER

PT J
AU Abdel-Bakky, MS
Amin, E
Faris, TM
Abdellatif, AAH
AF Abdel-Bakky, Mohamed S.
Amin, Elham
Faris, Tarek M.
Abdellatif, Ahmed A. H.
TI Mental depression: Relation to different disease status, newer treatments and its association with COVID-19 pandemic
SO MOLECULAR MEDICINE REPORTS
LA English
DT Review
DE depression; neurotransmitters; molecular mechanism; COVID-19; latest treatments; mental health
ID NOREPINEPHRINE REUPTAKE INHIBITOR; GROWTH-FACTOR CONTENT; MAJOR DEPRESSION; DOUBLE-BLIND; BIPOLAR DISORDER; NEUROTROPHIC FACTOR; ANTIDEPRESSANT TREATMENT; PREFRONTAL CORTEX; NITRIC-OXIDE; RISK-FACTOR
AB The present study aimed to review major depression, including its types, epidemiology, association with different diseases status and treatments, as well as its correlation with the current COVID-19 pandemic. Mental depression is a common disorder that affects most individuals at one time or another. During depression, there are changes in mood and behavior, accompanied by feelings of defeat, hopelessness, or even suicidal thoughts. Depression has a direct or indirect relation with a number of other diseases including Alzheimer's disease, stroke, epilepsy, diabetes, cardiovascular disease and cancer. In addition, antidepressant drugs have several side effects including sedation, increased weight, indigestion, sexual dysfunction, or a decrease in blood pressure. Stopping medication may cause a relapse of the symptoms of depression and pose a risk of attempted suicide. The pandemic of COVID-19 has affected the mental health of individuals, including patients, individuals contacting patients and medical staff with a number of mental disorders that may adversely affect the immune ability of their bodies. Some of the drugs currently included in the protocols for treating COVID-19

may negatively affect the mental health of patients. Evidence accumulated over the years indicates that serotonin (5HT) deficiencies and norepinephrine (NE) in the brain can lead to mental depression. Drugs that increase levels of NE and 5HT are commonly used in the treatment of depression. The common reason for mood disorders, including mania and bipolar disease are not clearly understood. It is assumed that hyperactivity in specific parts of the brain and excessive activity of neurotransmitters may be involved. Early diagnosis and developing new treatment strategies are essential for the prevention of the severe consequences of depression. In addition, extensive research should be directed towards the investigation of the mental health disturbances occurring during and/or after COVID-19 infection. This may lead to the incorporation of a suitable antidepressant into the current treatment protocols.
C1 [Abdel-Bakky, Mohamed S.] Qassim Univ, Coll Pharm, Dept Pharmacol & Toxicol, Buraydah 51452, Qassim, Saudi Arabia.
[Abdel-Bakky, Mohamed S.] Al Azhar Univ, Fac Pharm Boys, Dept Pharmacol & Toxicol, Cairo 11884, Egypt.
[Amin, Elham] Beni Suef Univ, Fac Pharm, Dept Pharmacognosy, Bani Suwayf 62514, Egypt.
[Amin, Elham] Qassim Univ, Coll Pharm, Dept Med Chem & Pharmacognosy, Buraydah 52471, Qassim, Saudi Arabia.
[Faris, Tarek M.] Al Azhar Univ, Coll Pharm, Dept Pharmaceut & Ind Pharm, Cairo 11884, Egypt.
[Abdellatif, Ahmed A. H.] Qassim Univ, Coll Pharm, Dept Pharmaceut, A2 Almelida St, Buraydah 51452, Qassim, Saudi Arabia.
[Abdellatif, Ahmed A. H.] Al Azhar Univ, Fac Pharm, Dept Pharmaceut & Ind Pharm, Assiut 71524, Egypt.
C3 Qassim University; Egyptian Knowledge Bank (EKB); Al Azhar University;
Egyptian Knowledge Bank (EKB); Beni Suef University; Qassim University;
Egyptian Knowledge Bank (EKB); Al Azhar University; Qassim University;
Egyptian Knowledge Bank (EKB); Al Azhar University
RP Abdellatif, AAH (通讯作者)，Qassim Univ, Coll Pharm, Dept Pharmaceut, A2 Almelida St, Buraydah 51452, Qassim, Saudi Arabia.
EM a.abdellatif@qu.edu.sa
TC 1
Z9 1
PD DEC
PY 2021
VL 24
IS 6
AR 839
DI 10.3892/mmr.2021.12479
WC Oncology; Medicine, Research & Experimental
ER

PT J
AU Kubota, T
Kuroda, N
Horinouchi, T

Ikegaya, N
Kitazawa, Y
Kodama, S
Kuramochi, I
Matsubara, T
Nagino, N
Neshige, S
Soga, T
Takayama, Y
Sone, D
AF Kubota, Takafumi
Kuroda, Naoto
Horinouchi, Toru
Ikegaya, Naoki
Kitazawa, Yu
Kodama, Satoshi
Kuramochi, Izumi
Matsubara, Teppei
Nagino, Naoto
Neshige, Shuichiro
Soga, Temma
Takayama, Yutaro
Sone, Daichi
TI Barriers to telemedicine among physicians in epilepsy care during the COVID-19 pandemic: A national-level cross-sectional survey in Japan
SO EPILEPSY & BEHAVIOR
LA English
DT Article
DE Telemedicine; Epilepsy; Seizure; Barrier; COVID-19
ID HEALTH; FEASIBILITY; TELEHEALTH; CHILDREN
AB Objective: This study aimed to investigate the factors affecting the unwillingness of physicians involved in epilepsy care to continue telemedicine during the coronavirus disease 2019 (COVID-19) pandemic in Japan. Method: This was a national-level cross-sectional survey initiated by Japan Young Epilepsy Section (YESJapan) which is a national chapter of The Young Epilepsy Section of the International League Against Epilepsy (ILAE-YES). We asked physicians who conducted telemedicine in patients with epilepsy (PWE) during the COVID-19 pandemic at four clinics and 21 hospitals specializing in epilepsy care in Japan from March 1 to April 30, 2021. The following data were collected: (1) participant profile, (2) characteristics of PWE treated by telemedicine, and (3) contents and environmental factors of telemedicine. Statistically significant variables ($p < 0.05$) in the univariate analysis were analyzed in a multivariate binary logistic regression model to detect the independently associated factors with the unwillingness to continue telemedicine. Result: Among the 115 respondents (response rate: 64%), 89 were included in the final analysis. Of them, 60 (67.4%) were willing to continue telemedicine, and 29 (32.6%) were unwilling. In the univariate binary logistic regression analysis, age (Odds ratio [OR] = 1.84, 95% confidence interval [CI] 1.10-3.09, p = 0.02), psychiatrist (OR = 5.88, 95% CI

2.15-16.08, p = 0.001), hospital (OR = 0.10, 95% CI 0.01-0.94, p = 0.04), the number of COVID-19 risk factors in the participant (OR = 2.88, 95% CI 1.46-5.69, p = 0.002), the number of COVID-19 risk factors in the cohabitants (OR = 2.52, 95% CI 1.05-6.01, p = 0.04), COVID-19 epidemic area (OR = 4.37, 95% CI 1.18-16.20, p = 0.03), consultation time during telemedicine (OR = 2.51, 95% CI 1.32- 4.76, p = 0.005), workload due to telemedicine (OR = 4.17, 95% CI 2.11-8.24, p < 0.001) were statistically significant. In the multivariate binary logistic regression analysis, workload due to telemedicine (OR = 4.93, 95% CI 1.96-12.35) was independently associated with the unwillingness to continue teleme-dicine. Conclusion: This national-level cross-sectional survey found that workload due to telemedicine among physicians involved in epilepsy care was independently associated with the unwillingness to continue telemedicine. (c) 2021 Elsevier Inc. All rights reserved.
C1 [Kubota, Takafumi; Kuroda, Naoto; Horinouchi, Toru; Ikegaya, Naoki; Kitazawa, Yu; Kodama, Satoshi; Kuramochi, Izumi; Matsubara, Teppei; Nagino, Naoto; Neshige, Shuichiro; Soga, Temma; Takayama, Yutaro; Sone, Daichi] Japan Young Epilepsy Sect, Kodaira, Tokyo, Japan.

[Kubota, Takafumi] Case Western Reserve Univ, Univ Hosp, Univ Hosp, Dept Neurol, Cleveland, OH 44106 USA.

[Kubota, Takafumi; Soga, Temma] Tohoku Univ, Grad Sch Med, Dept Neurol, Aoba Ku, 2-1 Seiryo Machi, Sendai, Miyagi 9808575, Japan.

[Kuroda, Naoto; Soga, Temma] Tohoku Univ, Grad Sch Med, Dept Epileptol, Sendai, Miyagi, Japan.

[Kuroda, Naoto] Wayne State Univ, Childrens Hosp Michigan, Detroit Med Ctr, Dept Pediat, Detroit, MI USA.

[Horinouchi, Toru] Hokkaido Univ, Grad Sch Med, Dept Psychiat & Neurol, Sapporo, Hokkaido, Japan.

[Ikegaya, Naoki] Yokohama City Univ, Grad Sch Med, Dept Neurosurg, Kanazawa Ku, 3-9 Fukuura, Yokohama, Kanagawa, Japan.

[Kitazawa, Yu] Yokohama City Univ, Dept Neurol & Stroke Med, Yokohama, Kanagawa, Japan.

[Kodama, Satoshi] Univ Tokyo, Grad Sch Med, Dept Neurol, Tokyo, Japan.

[Kuramochi, Izumi] Saitama Med Univ, Saitama Med Ctr, Dept Psychiat, Kawagoe, Saitama, Japan.

[Matsubara, Teppei] Massachusetts Gen Hosp, Athinoula Martinos Ctr Biomed Imaging, Charlestown, MA USA.

[Nagino, Naoto] TMG Asaka Med Ctr, Epilepsy Ctr, Saitama, Japan.

[Neshige, Shuichiro] Hiroshima Univ, Grad Sch Biomed & Hlth Sci, Dept Clin Neurosci & Therapeut, Hiroshima, Hiroshima, Japan.

[Takayama, Yutaro] Natl Ctr Hosp, Natl Ctr Neurol & Psychiat, Dept Neurosurg, Kodaira, Tokyo, Japan.

[Sone, Daichi] UCL Inst Neurol, Dept Clin & Expt Epilepsy, London, England.

C3 Case Western Reserve University; University Hospitals of Cleveland; Tohoku University; Tohoku University; Children's Hospital of Michigan; Detroit Medical Center; Wayne State University; Hokkaido University; Yokohama City University; Yokohama City University; University of Tokyo; Saitama Medical University; Harvard University; Massachusetts General Hospital; Hiroshima University; National Center for Neurology &

Psychiatry - Japan; University of London; University College London
RP Kubota, T (通讯作者), Tohoku Univ, Grad Sch Med, Dept Neurol, Aoba Ku, 2-1 Seiryo Machi, Sendai, Miyagi 9808575, Japan.; Ikegaya, N (通讯作者), Yokohama City Univ, Grad Sch Med, Dept Neurosurg, Kanazawa Ku, 3-9 Fukuura, Yokohama, Kanagawa, Japan.
EM takafumi.kubota.c7@tohoku.ac.jp; nikegaya@yokohama-cu.ac.jp
TC 0
Z9 0
PD JAN
PY 2022
VL 126
AR 108487
DI 10.1016/j.yebeh.2021.108487
EA DEC 2021
WC Behavioral Sciences; Clinical Neurology; Psychiatry
ER

PT J
AU Nakornchai, T
Conci, E
Hensiek, A
Brown, JWL
AF Nakornchai, Tagore
Conci, Elena
Hensiek, Anke
Brown, J. William L.
TI Clinician and patient experience of neurology telephone consultations during the COVID-19 pandemic
SO POSTGRADUATE MEDICAL JOURNAL
LA English
DT Article; Early Access
DE adult neurology; neurology; telemedicine
ID TELEMEDICINE
AB Background Telephone consultations are already employed in specific neurological settings. At Cambridge University Hospitals, the COVID-19 pandemic initially prompted almost all face-to-face appointments to be delivered by telephone, providing a uniquely unselected population to assess. Objectives We explored patient and clinician experience of telephone consultations; and whether telephone consultations might be preferable for preidentifiable subgroups of patients after the pandemic. Methods Clinicians delivering neurological consultations converted to telephone between April and July 2020 were invited to complete a questionnaire following each consult (430 respondents) and the corresponding patients were subsequently surveyed (290 respondents). The questionnaires assessed clinician and patient goal achievement (and the reasons for any dissatisfaction). Clinicians also described consultation duration (in comparison to face to face) while patients detailed comparative convenience and preference. Results The majority of clinicians (335/430, 78%) and patients (227/290, 78%) achieved their consultation goals by telephone, particularly during follow-up consultations (clinicians 272/329, 83%, patients 176/216, 81%) and in some disease

subgroups (eg, seizures/epilepsy (clinicians 114/122 (93%), patients 71/81 (88%)). 95% of telephone consultations were estimated to take the same or less time than an equivalent face-to-face consultation. Most patients found telephone consultations convenient (69%) with 149/211 (71%) indicating they would like telephone or video consultations to play some role in their future follow-up. Conclusion Telephone consultations appear effective, convenient and popular in prespecified subgroups of neurological outpatients. Further work comparing telephone, video and face-to-face consultations across multiple centres is now needed.
C1 [Nakornchai, Tagore; Brown, J. William L.] Univ Cambridge, Dept Clin Neurosci, Cambridge, Cambs, England.
[Nakornchai, Tagore; Conci, Elena; Hensiek, Anke] Cambridge Univ Hosp NHS Fdn Trust, Dept Neurol, Cambridge, England.
C3 League of European Research Universities - LERU; University of
Cambridge; League of European Research Universities - LERU; University
of Cambridge
RP Nakornchai, T (通讯作者), Univ Cambridge, Dept Clin Neurosci, Cambridge, Cambs, England.
EM tagore.nakornchai@nhs.net
TC 0
Z9 0
DI 10.1136/postgradmedj-2021-141234
EA DEC 2021
WC Medicine, General & Internal
ER

PT J
AU Santoso, IB
Adrianto, Y
Sensusiati, AD
Wulandari, DP
Purnama, IKE
AF Santoso, Irwan Budi
Adrianto, Yudhi
Sensusiati, Anggraini Dwi
Wulandari, Diah Puspito
Purnama, I. Ketut Eddy
TI Ensemble Convolutional Neural Networks With Support Vector Machine for
Epilepsy Classification Based on Multi-Sequence of Magnetic Resonance
Images
SO IEEE ACCESS
LA English
DT Article
DE Epilepsy; Brain modeling; Convolutional neural networks; Magnetic
resonance imaging; Feature extraction; Predictive models; Transfer
learning; Convolutional neural network; ensemble; epilepsy; magnetic
resonance image; support vector machine
ID COVID-19

AB Classification of brain abnormalities as a pathological cue of epilepsy based on magnetic resonance (MR) images is essential for diagnosis. There are some types of brain structural abnormalities as a pathological cue of epilepsy. To identify it, a neurologist can involve some sequence of MR images at a time. Existing algorithms for abnormalities classification usually involve only one or two sequences of MR images. In this paper, we proposed ensemble convolutional neural networks with a support vector machine (SVM) scheme to classify brain abnormalities (epilepsy) vs. non-epilepsy based on the axial multi-sequence of MR images. The convolutional neural network (CNN) models on the proposed method are base-learner models with different architectures and have low parameters. The performance improvement on the proposed method is made by combining the output of the base-learner models and the combination of predictions from these models. The combination of predictions uses majority voting, weighted majority voting, and weighted average. Henceforth, the combined output becomes input in the meta-learning process with SVM for the final classification. The dataset for evaluation is the axial multi-sequences of MR images that include abnormal brain structures causing epilepsy and non-epilepsy with various subjects' histories. The experimental results show the proposed method can obtain an accuracy average and F-1-score of 86.37% and 90.75%, respectively, and an improvement of accuracy of 6.7%-18.19% against the CNN models on the base-learner and 2.54%-2.65% against the combination of predictions. With these results, the proposed architecture also provides better performance compared to the two existing CNN architectures.
C1 [Santoso, Irwan Budi; Wulandari, Diah Puspito; Purnama, I. Ketut Eddy] Inst Teknol Sepuluh Nopember, Dept Elect Engn, Surabaya 60111, Indonesia.

[Santoso, Irwan Budi] Univ Islam Negeri Maulana Malik Ibrahim Malang, Dept Informat Engn, Malang 65144, Indonesia.

[Adrianto, Yudhi] Univ Airlangga, Dept Neurol, Surabaya 60115, Indonesia.

[Sensusiati, Anggraini Dwi] Univ Airlangga, Dept Radiol, Surabaya 60115, Indonesia.

[Wulandari, Diah Puspito; Purnama, I. Ketut Eddy] Inst Teknol Sepuluh Nopember, Dept Comp Engn, Surabaya 60111, Indonesia.

C3 Institut Teknologi Sepuluh Nopember; Airlangga University; Airlangga University; Institut Teknologi Sepuluh Nopember

RP Purnama, IKE (通讯作者), Inst Teknol Sepuluh Nopember, Dept Elect Engn, Surabaya 60111, Indonesia.; Purnama, IKE (通讯作者), Inst Teknol Sepuluh Nopember, Dept Comp Engn, Surabaya 60111, Indonesia.

EM ketut@te.its.ac.id

TC 0

Z9 0

PY 2022

VL 10

BP 32034

EP 32048

DI 10.1109/ACCESS.2022.3159923

WC Computer Science, Information Systems; Engineering, Electrical & Electronic; Telecommunications

ER

PT J

AU Chan-Nguyen, S
Ritsma, B
Nguyen, L
Srivastava, S
Shukla, G
Appireddy, R
AF Chan-Nguyen, Sophy
Ritsma, Benjamin
Nguyen, Lisa
Srivastava, Siddhartha
Shukla, Garima
Appireddy, Ramana
TI Virtual Care Access and Health Equity during the COVID-19 Pandemic, a qualitative study of patients with chronic diseases from Canada
SO DIGITAL HEALTH
LA English
DT Article
DE Care; COVID-19; chronic disease; epilepsy
ID INCORPORATING INTERSECTIONALITY; CHALLENGES
AB Objectives The COVID-19 pandemic has led to the widespread uptake of virtual care in Canada; however, virtual care may also create new barriers to health care. The purpose of this paper was to explore patient perceptions and concerns around virtual care access. Methods Between February and April 2020, we conducted semi-structured interviews with participants from four chronic disease clinics (stroke, epilepsy, amyotrophic lateral sclerosis, obstetrics medicine) in a mid-sized academic hospital in Southern Ontario, Canada. Consecutive sampling was done by including the patients receiving virtual care in those months. Caregivers were invited to participate in the event that patients were unable to participate in the interview. Thematic analysis was employed to identify overarching themes, and codes were reviewed and refined using a consensus process. Results We interviewed 31 participants (27 patients, four caregivers) that had taken part in virtual care. Our findings suggested that the COVID-19 pandemic served to isolate participants and had negatively impacted their access to health care. However, virtual care did provide a safe avenue for patients to receive care and served as a reassuring option during the pandemic. Low technological literacy and access were identified as barriers to virtual care. Greater awareness and patient engagement is needed in future research to improve access. Conclusion Certain populations can be disproportionately affected by differential access to virtual care. Future studies should examine how social determinants intersect to impact virtual health care access in different patient populations.
C1 [Chan-Nguyen, Sophy] Queens Univ, Dept Family Med, Kingston, ON, Canada.
[Ritsma, Benjamin] Queens Univ, Dept Phys Med & Rehabil, Kingston, ON, Canada.
[Nguyen, Lisa; Srivastava, Siddhartha; Shukla, Garima; Appireddy, Ramana] Queens Univ, Dept Med, Kingston, ON, Canada.
C3 Queens University - Canada; Queens University - Canada; Queens University - Canada
RP Appireddy, R (通讯作者), Queens Univ, Dept Med, Kingston, ON, Canada.
EM mrra@queensu.ca

TC 0
Z9 0
PD JAN
PY 2022
VL 8
AR 20552076221074486
DI 10.1177/20552076221074486
WC Health Care Sciences & Services; Health Policy & Services; Public, Environmental & Occupational Health; Medical Informatics
ER

PT J
AU Daniels, H
Lacey, AS
Mikadze, D
Akbari, A
Fonferko-Shadrach, B
Hollinghurst, J
Lyons, RA
Rees, MI
Sawhney, IMS
Powell, RH
Kerr, MP
Pickrell, WO
AF Daniels, Helen
Lacey, Arron S.
Mikadze, David
Akbari, Ashley
Fonferko-Shadrach, Beata
Hollinghurst, Joe
Lyons, Ronan A.
Rees, Mark, I
Sawhney, Inder M. S.
Powell, Robert H.
Kerr, Michael P.
Pickrell, W. Owen
TI Epilepsy mortality in Wales during COVID-19
SO SEIZURE-EUROPEAN JOURNAL OF EPILEPSY
LA English
DT Article
DE Data linkage; Electronic health records; Pandemic; COVID-19
AB Purpose: The COVID-19 pandemic has increased mortality worldwide and those with chronic conditions may have been disproportionally affected. However, it is unknown whether the pandemic has changed mortality rates for people with epilepsy. We aimed to compare mortality rates in people with epilepsy in Wales during the pandemic with pre-pandemic rates. Methods: We performed a retrospective study using individual-level linked population-scale anonymised electronic health records. We identified deaths in

people with epilepsy (DPWE), i.e. those with a diagnosis of epilepsy, and deaths associated with epilepsy (DAE), where epilepsy was recorded as a cause of death on death certificates. We compared death rates in 2020 with average rates in 2015-2019 using Poisson models to calculate death rate ratios. Results: There were 188 DAE and 628 DPWE in Wales in 2020 (death rates: 7.7/100,000/year and 25.7/100,000/ year). The average rates for DAE and DPWE from 2015 to 2019 were 5.8/100,000/year and 23.8/100,000/year, respectively. Death rate ratios (2020 compared to 2015-2019) for DAE were 1.34 (95%CI 1.14-1.57, p<0.001) and for DPWE were 1.08 (0.99-1.17, p = 0.09). The death rate ratios for non-COVID deaths (deaths without COVID mentioned on death certificates) for DAE were 1.17 (0.99-1.39, p = 0.06) and for DPWE were 0.96 (0.87-1.05, p = 0.37). Conclusions: The significant increase in DAE in Wales during 2020 could be explained by the direct effect of COVID-19 infection. Non-COVID-19 deaths have not increased significantly but further work is needed to assess the longer-term impact.
C1 [Daniels, Helen; Lacey, Arron S.; Mikadze, David; Akbari, Ashley; Fonferko-Shadrach, Beata; Hollinghurst, Joe; Lyons, Ronan A.; Rees, Mark, I; Sawhney, Inder M. S.; Powell, Robert H.; Pickrell, W. Owen] Swansea Univ, Med Sch, Swansea SA2 8PP, W Glam, Wales.
[Sawhney, Inder M. S.; Powell, Robert H.; Pickrell, W. Owen] Morriston Hosp, Swansea Bay Univ Hlth Board, Swansea, W Glam, Wales.
[Rees, Mark, I] Univ Sydney, Fac Med & Hlth, Sydney, NSW, Australia.
[Kerr, Michael P.] Cardiff Univ, Inst Psychol Med & Clin Neurosci, Sch Med, Cardiff, Wales.
C3 Swansea University; Morriston Hospital; University of Sydney; Cardiff University
RP Daniels, H (通讯作者), Swansea Univ, Med Sch, Swansea SA2 8PP, W Glam, Wales.
EM h.daniels@swansea.ac.uk; a.s.lacey@swansea.ac.uk; 970837@swansea.ac.uk; a.akbari@swansea.ac.uk; b.fonferko-shadrach@swansea.ac.uk; j.hollinghurst@swansea.ac.uk; r.a.lyons@swansea.ac.uk; mark.rees@sydney.edu.au; i.m.s.sawhney@swansea.ac.uk; Robert.powell@wales.nhs.uk; kerrmp@cardiff.ac.uk; w.o.pickrell@swansea.ac.uk
TC 0
Z9 0
PD JAN
PY 2022
VL 94
BP 39
EP 42
DI 10.1016/j.seizure.2021.11.017
WC Clinical Neurology; Neurosciences
ER

PT J
AU Horvath, RA
Suto, Z
Cseke, B
Schranz, D
Darnai, G

Kovacs, N
Janszky, I
Janszky, J
AF Horvath, Reka A.
Suto, Zsofia
Cseke, Balazs
Schranz, Daniel
Darnai, Gergely
Kovacs, Norbert
Janszky, Imre
Janszky, Jozsef
TI Epilepsy is overrepresented among young people who died from COVID-19: Analysis of nationwide mortality data in Hungary
SO SEIZURE-EUROPEAN JOURNAL OF EPILEPSY
LA English
DT Article
DE Epilepsy; COVID-19; Age; Death; Intellectual disability; Hungary
ID LONG-TERM; ADULT EPILEPSY; PREVALENCE; SEIZURES; DEATH
AB Background: Studies examining epilepsy as a COVID-related death risk have come to conflicting conclusions. Our aim was to assess the prevalence of epilepsy among COVID-related deaths in Hungary. Methods: Each COVID-19 infection case is required to be reported on a daily basis to the National Public Health Center of Hungary. This online report includes the beginning and end of the infection, as well as information on comorbidities. Death during infection is regarded as COVID-related. The anonymized data of each deceased patient are published on an information website (www.koronavirus.gov.hu) and provides up-to-date information on each patient with the date of death, the patient's sex, age, and chronic illness. Results: There were 11,968 patients who died of COVID-19 in Hungary between 13 March 2020 and 23 January 2021. Among 11,686 patients with no missing values for comorbidities, 255 patients had epilepsy (2.2%). Ep-ilepsy was much more common among those who died at a young age: 9.3% of those who died under the age of 50 had epilepsy, compared with only 1.3% in those over the age of 80. The younger an age group was, the higher was the prevalence of epilepsy. Conclusion: Patients who died of COVID-19 under the age of 50 were 10 to 20 times more likely to have epilepsy than what would have been expected from epidemiological data. Our results highlight the need for increased protection of young people with epilepsy from COVID-19 infection and the development of a vaccination strategy accordingly.
C1 [Horvath, Reka A.; Suto, Zsofia; Cseke, Balazs; Schranz, Daniel; Darnai, Gergely; Kovacs, Norbert; Janszky, Jozsef] Univ Pecs, Med Sch, Dept Neurol, Ret U 2, H-7623 Pecs, Hungary.
[Darnai, Gergely; Kovacs, Norbert; Janszky, Jozsef] MTA PTE Clin Neurosci MRI Res Grp, Ifjusag U 20, H-7624 Pecs, Hungary.
[Darnai, Gergely] Univ Pecs, Med Sch, Dept Behav Sci, Ret U 2, H-7623 Pecs, Hungary.
[Janszky, Imre] Norwegian Univ Sci & Technol, Fac Med & Hlth Sci, Dept Publ Hlth & Nursing, Hakon Jarls Gate 11 & Mauritz Hanssens Gate, N-7491 Trondheim, Norway.
[Janszky, Imre] St Olavs Hosp, Reg Ctr Hlth Care Improvement, Trondheim, Norway.
C3 University of Pecs; University of Pecs; Norwegian University of Science

& Technology (NTNU); Norwegian University of Science & Technology (NTNU)
RP Janszky, J (通讯作者), Univ Pecs, Med Sch, Dept Neurol, Ret U 2, H-7623 Pecs, Hungary.
EM horvath.reka@pte.hu; suto.zsofia@pte.hu; cseke.balazs@pte.hu; schranz.daniel@pte.hu; darnai.gergely@pte.hu; kovacs.norbert@pte.hu; imre.janszky@ntnu.no; janszky.jozsef@pte.hu
TC 0
Z9 0
PD JAN
PY 2022
VL 94
BP 136
EP 141
DI 10.1016/j.seizure.2021.11.013
WC Clinical Neurology; Neurosciences
ER

PT J
AU Fine, AL
Wong-Kisiel, LC
Nickels, KC
Wirrell, EC
AF Fine, Anthony L.
Wong-Kisiel, Lily C.
Nickels, Katherine C.
Wirrell, Elaine C.
TI Masking for School-Age Children With Epilepsy: We Do Have Consensus!: Masking for Children with Epilepsy
SO JOURNAL OF CHILD NEUROLOGY
LA English
DT Article
DE epilepsy; children; mask; COVID-19
ID COVID-19
AB Introduction: This study was designed to assess current recommendations from child neurologists and epileptologists on masking for school-age children with epilepsy.
Methods: A 7-item survey was created and sent out to members of the Child Neurology Society and Pediatric Epilepsy Research Consortium in August of 2021 to assess current practice and provider recommendations on masking.
Results: One hundred four individuals participated with representation from all regions of the United States. Masking was recommended by 95.1%, with 63.4% (n=66) noting exception of those with severe intellectual disability, autism, and behavioral problems. Of those who write exemption letters, 54% write these <5% of the time. Only 3% reported potential adverse events associated with masking.
Conclusion: Nearly all respondents recommended masking for school-age children with epilepsy. Potential risks of masking and adverse events were low. Improved guidance on masking is needed to ensure academic success of our patients with epilepsy.
C1 [Fine, Anthony L.; Wong-Kisiel, Lily C.; Nickels, Katherine C.; Wirrell, Elaine C.] Mayo Clin, Rochester, MN 55905 USA.

C3 Mayo Clinic
RP Fine, AL (通讯作者), Mayo Clin, Dept Neurol, Div Child & Adolescent Neurol, 200 First St SW, Rochester, MN 55905 USA.; Fine, AL (通讯作者), Mayo Clin, Dept Neurol, Div Epilepsy, 200 First St SW, Rochester, MN 55905 USA.
EM Fine.anthony@mayo.edu
TC 0
Z9 0
PD FEB
PY 2022
VL 37
IS 2
BP 127
EP 132
AR 08830738211063684
DI 10.1177/08830738211063684
EA JAN 2022
WC Clinical Neurology; Pediatrics
ER

PT J
AU Pellinen, J
   Holmes, MG
AF Pellinen, Jacob
   Holmes, Manisha Gupte
TI Evaluation and Treatment of Seizures and Epilepsy During the COVID-19
   Pandemic
SO CURRENT NEUROLOGY AND NEUROSCIENCE REPORTS
LA English
DT Review
DE Convulsions; Clinical neurology; Telemedicine; EEG; Encephalography
ID STATUS EPILEPTICUS; CONTINUOUS ELECTROENCEPHALOGRAPHY; COHORT
AB Purpose of Review Seizures, including status epilepticus, have been reported in association with acute COVID-19 infection. People with epilepsy (PWE) have suffered from seizure exacerbations during the pandemic. This article reviews the data for clinical and electrographic seizures associated with COVID-19, technical EEG considerations for reducing risk of transmission, and factors contributing to seizure exacerbations in PWE as well as strategies to address this issue.
   Recent Findings An increasing number of studies of larger cohorts, accounting for a variety of variables and often utilizing EEG with standardized terminology, are assessing the prevalence of seizures in hospitalized patients with acute COVID-19 infections, and gaining insight into the prevalence of seizures and their effect on outcomes. Additionally, recent studies are evaluating the effect of the pandemic on PWE, barriers faced, and the usefulness of telehealth.
   Summary Although there is still much to learn regarding COVID-19, current studies help in assessing the risk of seizures, guiding EEG utilization, and optimizing the use of telehealth during the pandemic.
C1 [Pellinen, Jacob] Univ Colorado, Sch Med, Dept Neurol, Aurora, CO USA.

[Holmes, Manisha Gupte] NYU, Sch Med, Comprehens Epilepsy Ctr, New York, NY 10012 USA.
C3 University of Colorado System; University of Colorado Anschutz Medical Campus; New York University
RP Holmes, MG (通讯作者), NYU, Sch Med, Comprehens Epilepsy Ctr, New York, NY 10012 USA.
EM Jacob.Pellinen@cuanschutz.edu; Manisha.Holmes@nyulangone.org
TC 0
Z9 0
PD JAN
PY 2022
VL 22
IS 1
BP 11
EP 17
DI 10.1007/s11910-022-01174-x
EA JAN 2022
WC Clinical Neurology; Neurosciences
ER

PT J
AU Gul, ZB
AF Gul, Zeynep Bastug
TI Depression and sexual functions in epilepsy patients: Comparison before and during the COVID-19 pandemic
SO ANNALES MEDICO-PSYCHOLOGIQUES
LA English
DT Article
DE COVID-19; Depression; Epilepsy; Sexual function
ID EXPERIENCES SCALE; DYSFUNCTION; DISORDERS; STIGMA
AB Objective. - The new coronavirus disease 2019 (COVID-19) is a major health problem with a high rate of spread. We aimed to investigate herein the effects of the COVID-19 outbreak on depression and sexual dysfunction in patients with epilepsy.

Material and Methods. - One hundred and sixteen epilepsy patients admitted to our hospital from October-November 2019 were evaluated for sexual functions and depression using the Arizona Sexual Experiences Scale (ASEX) and Beck Depression Scale, respectively. These scales were re-applied in June 2020 and July 2020 to assess the impact of the outbreak on sexual function and depression in the same group of patients. The demographic and clinical characteristics of the patients were recorded and analyzed in SPSS.

Results. - During the pandemic period, the total Beck Depression Scale values increased significantly in the patients with epilepsy compared to the pre-pandemic period (P = 0.048), and depressive symptoms showed an increasing trend (P = 0.032). Although an increase in sexual dysfunction was also recorded, it was not statistically significant compared to the pre-pandemic period. In eight patients (6.9%), seizure frequency increased during the pandemic period. In the multivariate analysis, the only parameter that predicted the increase in seizure frequency was the number of drugs used.

The Beck Depression Scale values were positively correlated with total male/female ASEX values, age, marital status, duration of illness, and seizure frequency.
Conclusion. - The COVID-19 outbreak caused an increase in the tendency to depression in epilepsy patients and has also had a negative effect on sexual function. During public health outbreaks, clinicians should focus not only on seizure control in patients with epilepsy but also on their mental health. (C) 2021 Elsevier Masson SAS. All rights reserved.
C1 [Gul, Zeynep Bastug] Univ Hlth Sci, Bakirkoy Training & Res Hosp Psychiat & Neurol Di, Dept Neurol, Doktor Tevf Saglam Cd 25-2, TR-34147 Bakirkoy Istanbul, Turkey.
C3 Istanbul Bakirkoy Mental Health & Neurology Training & Research Hospital
RP Gul, ZB (通讯作者), Univ Hlth Sci, Bakirkoy Training & Res Hosp Psychiat & Neurol Di, Dept Neurol, Doktor Tevf Saglam Cd 25-2, TR-34147 Bakirkoy Istanbul, Turkey.
EM drzeynep34@hotmail.com
TC 2
Z9 2
PD FEB
PY 2022
VL 180
IS 2
BP 127
EP 132
DI 10.1016/j.amp.2021.02.006
WC Pharmacology & Pharmacy; Psychiatry; Psychology; Psychology, Multidisciplinary
ER

PT J
AU Wai, AKC
Wong, CKH
Wong, JYH
Xiong, X
Chu, OCK
Wong, MS
Tsui, MSH
Rainer, TH
AF Wai, Abraham K. C.
Wong, Carlos K. H.
Wong, Janet Y. H.
Xiong, Xi
Chu, Owen C. K.
Wong, Man S.
Tsui, Matthew S. H.
Rainer, Timothy H.
TI Changes in Emergency Department Visits, Diagnostic Groups, and 28-Day Mortality Associated With the COVID-19 Pandemic: A Territory-Wide, Retrospective, Cohort Study
SO ANNALS OF EMERGENCY MEDICINE

LA English
DT Article
DE COVID-19; Cohort Study; epilepsy
ID IMPACT
AB Study objective: We aimed to evaluate and characterize the scale and relationships of emergency department (ED) visits and excess mortality associated with the early phase of the COVID-19 pandemic in the territory of Hong Kong.

Methods: We conducted a territory-wide, retrospective cohort study to compare ED visits and the related impact of the COVID-19 pandemic on mortality. All ED visits at 18 public acute hospitals in Hong Kong between January 1 and August 31 of 2019 (n=1,426,259) and 2020 (n=1,035,562) were included. The primary outcome was all-cause mortality in the 28 days following an ED visit. The secondary outcomes were weekly number of ED visits and diagnosis-specific mortality.

Results: ED visits decreased by 27.4%, from 1,426,259 in 2019 to 1,035,562 in 2020. Overall period mortality increased from 28,686 (2.0%) in 2019 to 29,737 (2.9%) in 2020. The adjusted odds ratio for 28-day, all-cause mortality in the pandemic period of 2020 relative to 2019 was 1.26 (95% confidence interval 1.24 to 1.28). Both sexes, age more than 45 years, all triage categories, all social classes, all ED visit periods, epilepsy (odds ratio 1.58, 95% confidence interval 1.20 to 2.07), lower respiratory tract infection, and airway disease had higher adjusted ORs for all-cause mortality.

Conclusion: A significant reduction in ED visits in the first 8 months of the COVID-19 pandemic was associated with an increase in deaths certified in the ED. The government must make provisions to encourage patients with alarming symptoms, mental health conditions, and comorbidities to seek timely emergency care, regardless of the pandemic.
C1 [Wai, Abraham K. C.; Chu, Owen C. K.; Rainer, Timothy H.] Univ Hong Kong, Li Ka Shing Fac Med, Emergency Med Unit, Hong Kong, Peoples R China.

[Wai, Abraham K. C.; Tsui, Matthew S. H.; Rainer, Timothy H.] Queen Mary Hosp, Accid & Emergency Dept, Hong Kong, Peoples R China.

[Wong, Carlos K. H.; Xiong, Xi] Univ Hong Kong, Li Ka Shing Fac Med, Ctr Safe Medicat Practice & Res, Dept Pharmacol & Pharm, Hong Kong, Peoples R China.

[Wong, Carlos K. H.] Univ Hong Kong, Li Ka Shing Fac Med, Dept Family Med & Primary Care, Hong Kong, Peoples R China.

[Wong, Janet Y. H.] Univ Hong Kong, Li Ka Shing Fac Med, Sch Nursing, Hong Kong, Peoples R China.

[Wong, Man S.] Hong Kong Polytech Univ, Dept Land Surveying & Geoinformat, Hong Kong, Peoples R China.

C3 University of Hong Kong; University of Hong Kong; University of Hong Kong; University of Hong Kong; University of Hong Kong; Hong Kong Polytechnic University

RP Wong, CKH (通讯作者), Univ Hong Kong, Li Ka Shing Fac Med, Ctr Safe Medicat Practice & Res, Dept Pharmacol & Pharm, Hong Kong, Peoples R China.; Wong, CKH (通讯作者), Univ Hong Kong, Li Ka Shing Fac Med, Dept Family Med & Primary Care, Hong Kong, Peoples R China.

EM carlosho@hku.hk
TC 1
Z9 1

PD FEB
PY 2022
VL 79
IS 2
BP 148
EP 157
DI 10.1016/j.annemergmed.2021.09.424
WC Emergency Medicine
ER

PT J
AU Skinner, HJ
Casares, M
Wombles, C
Brooks, K
Hussain, A
Seo, JH
Gireesh, ED
Claudio, AO
Lee, KH
Hill, M
Westerveld, M
AF Skinner, Holly J.
Casares, Maritsa
Wombles, Christina
Brooks, Kereen
Hussain, Ammar
Seo, Joo Hee
Gireesh, Elakkat D.
Claudio, Angel O.
Lee, Ki H.
Hill, Michelle
Westerveld, Michael
TI Comparison of care accessibility, costs, and quality with face-to-face and telehealth epilepsy clinic visits
SO EPILEPSY & BEHAVIOR
LA English
DT Article
DE Telemedicine; COVID-19; Seizure
AB During the COVID-19 pandemic, restrictions on reimbursement for telehealth visits were lifted and this visit type was suddenly available to patients around the United States of America. Telehealth visits offer potential cost savings for patients and families, which may vary by region of the world studied. Also, aggressiveness of the care patients receive may differ, and patients or families may be more likely to choose one visit type over another based on seizure control.

This is a prospective face-to-face clinic versus telehealth clinic visit comparison study involving patients with seizures, their legal guardians, and caretakers who

attend clinic. We compared travel distance, work-related factors, childcare, satisfaction of care, changes in seizure medication or diagnostics tests ordered, and willingness to cancel appointments to better understand the behavioral patterns of patients, caretakers, and providers.

Our results indicate that many patients and families still prefer in-person interactions with their medical providers. Patient and family satisfaction levels were equal with both visit types. No significant difference was seen in medical management between face-to-face and telehealth visits. Also, prior seizure control did not dictate the type of visit chosen. Telehealth participants were significantly more willing to cancel appointments if asked to switch to face-to-face then face-to-face participants asked to complete telehealth visits. Surprisingly, we found that patients and families choosing telehealth were not statistically more likely to be employed or take less time off work. Also, distance from home to office was not significantly shorter for participants choosing face-to-face visits.

Offering a combination of telehealth and face-to-face visits appears to be the optimal strategy in caring for patients with controlled and uncontrolled seizure disorders to ensure adherence with clinic visits and satisfaction with care. Our study suggests that providers are equally willing to adjust medications or order additional diagnostic testing regardless of visit type. Patients and families may be less likely to cancel telehealth visits than face-to-face visits; this finding may translate to improved seizure control and long-term decreased cost of care. (C) 2021 Elsevier Inc. All rights reserved.
C1 [Skinner, Holly J.; Wombles, Christina; Brooks, Kereen; Hussain, Ammar; Seo, Joo Hee; Gireesh, Elakkat D.; Claudio, Angel O.; Lee, Ki H.; Hill, Michelle] Advent Hlth Orlando Epilepsy, Orlando, FL USA.
[Casares, Maritsa] Advent Hlth Orlando Neurosci Inst, Orlando, FL USA.
[Westerveld, Michael] Advent Hlth Orlando Neuropsychol, Orlando, FL USA.
RP Skinner, HJ (通讯作者), AHMG Epilepsy Orlando, 615 E Princeton St, Suite 540, Orlando, FL 32803 USA.
EM holly.skinner.do@adventhealth.com
TC 0
Z9 0
PD FEB
PY 2022
VL 127
AR 108510
DI 10.1016/j.yebeh.2021.108510
WC Behavioral Sciences; Clinical Neurology; Psychiatry
ER

PT J
AU Assenza, G
Ricci, L
Lanzone, J
Boscarino, M
Vico, C
Narducci, F

Sancetta, B
Di Lazzaro, V
Tombini, M
AF Assenza, Giovanni
Ricci, Lorenzo
Lanzone, Jacopo
Boscarino, Marilisa
Vico, Carlo
Narducci, Flavia
Sancetta, Biagio
Di Lazzaro, Vincenzo
Tombini, Mario
TI Understanding and managing the impact of the COVID-19 pandemic and lockdown on patients with epilepsy
SO EXPERT REVIEW OF NEUROTHERAPEUTICS
LA English
DT Review
DE COVID-19; antiepileptic drug; epilepsy; anxiety; depression; sleep; telemedicine
ID DRUG-INTERACTIONS; CEREBRAL MALARIA; SEIZURES; PEOPLE; CHLOROQUINE; TELEMEDICINE; INCREASE
AB Introduction The coronavirus disease 2019 (COVID-19) pandemic represented a relevant issue for people with epilepsy (PwE). Medical care and social restrictions exposed PwE to a high risk of seizure worsening. Medical institutions answered to the pandemic assuring only emergency care and implementing a remote assistance that highlighted the technological obsolescence of the medical care paradigms for PwE. Area covered We reviewed the literature on the COVID-19-related factors influencing the epilepsy course, from the evidence of seizure risk in severe acute respiratory syndrome coronavirus-2 (SARS-CoV-2) infected PwE to anti-Sars-Cov-2 drugs interactions with antiseizure medications and the perceived changes of seizures in PwE. Expert opinion COVID-19 pandemic was a problematic experience for PwE. We must make treasure of the lessons learned during this period of social restrictions and employ the recent technological advances to improve PwE assistance, in particular telemedicine and electronic media for patients' education.
C1 [Assenza, Giovanni; Ricci, Lorenzo; Boscarino, Marilisa; Vico, Carlo; Narducci, Flavia; Sancetta, Biagio; Di Lazzaro, Vincenzo; Tombini, Mario] Univ Campus Biomed Rome, Unit Neurol Neurophysiol Neurobiol, Dept Med, Via Alvaro Portillo 21, I-00128 Rome, Italy.
[Lanzone, Jacopo] IRCCS Salvatore Maugeri Fdn, Inst Milan, Neurorehabil Dept, Milan, Italy.
[Lanzone, Jacopo] Univ Roma Tor Vergata, Dept Syst Med, Neurosci, Rome, Italy.
C3 University Campus Bio-Medico - Rome Italy; Istituti Clinici Scientifici Maugeri IRCCS; University of Rome Tor Vergata
RP Assenza, G (通讯作者), Univ Campus Biomed Rome, Unit Neurol Neurophysiol Neurobiol, Dept Med, Via Alvaro Portillo 21, I-00128 Rome, Italy.
EM g.assenza@unicampus.it
TC 0

Z9 0
PD FEB 1
PY 2022
VL 22
IS 2
BP 145
EP 153
DI 10.1080/14737175.2022.2031984
EA FEB 2022
WC Clinical Neurology; Pharmacology & Pharmacy
ER

PT J
AU Ozdemir, HN
Dere, B
Gokcay, F
Gokcay, A
AF Ozdemir, Huseyin Nezih
Dere, Birgul
Gokcay, Figen
Gokcay, Ahmet
TI Are COVID-19 vaccines safe for people with epilepsy? A cross-sectional study
SO NEUROLOGICAL SCIENCES
LA English
DT Article; Early Access
DE Epilepsy; COVID-19; Vaccine; Safety; people with epilepsy
ID ILAE COMMISSION; SEIZURES
AB Background COVID-19 disease was first seen in December 2019 and was declared a pandemic soon after. To fight the pandemic, there is an immense need for effective vaccines. The purposes of our study were to investigate the effect of coronavirus vaccines on seizures in people with epilepsy (PWE) and assess the adverse events of COVID-19 vaccine in PWE.

Methods This was a cross-sectional study. We included epilepsy patients who got vaccinated with two or three doses at least 1 month earlier. We gathered the data using a standardized form. The form contained questions about patients' demographic features, clinical features, and information about the vaccination and its adverse events. The questionnaire included questions about epilepsy-related adverse events.

Results We included 178 people with epilepsy in our study. The frequency of adverse events was lower than clinical studies of the vaccines. The mean number of seizures in the month before the vaccination was 1.62, between the doses was 1.61, and after vaccination was 1.64. There was no significant difference in the number of monthly seizures before the vaccination, the month between the doses, or the month after the vaccination ($p = 0.46$).

Conclusions The vaccines under consideration in our study were tolerated well by the epilepsy patients. The vaccines did not affect the monthly number of seizures of the PWE. A small number of patients had more seizures than normal after vaccination.

We think that benefits of the vaccines outweigh the slightly increased possibility of having a seizure after vaccination.
C1 [Ozdemir, Huseyin Nezih] Necip Fazil City Hosp, Dept Neurol, TR-46050 Kahramanmaras, Turkey.
[Dere, Birgul; Gokcay, Figen; Gokcay, Ahmet] Ege Univ, Med Sch, Dept Neurol, TR-35100 Izmir, Turkey.
C3 Kahramanmaras Necip Fazil City Hospital; Ege University
RP Ozdemir, HN (通讯作者), Necip Fazil City Hosp, Dept Neurol, TR-46050 Kahramanmaras, Turkey.
EM huseyinnezihozdemir@gmail.com
TC 0
Z9 0
DI 10.1007/s10072-022-05956-6
EA FEB 2022
WC Clinical Neurology; Neurosciences
ER

PT J
AU Yeni, K
Tulek, Z
Ozer, A
Cavusoglu, A
Inan, GS
Baykan, B
Bebek, N
AF Yeni, Kubra
Tulek, Zeliha
Ozer, Arif
Cavusoglu, Aysel
Inan, Gorkem Sirin
Baykan, Betul
Bebek, Nerses
TI The effect of fear of COVID-19 on quality of life in patients with epilepsy
SO NEUROLOGY ASIA
LA English
DT Article
DE Epilepsy; fear; anxiety; depression; quality of life
ID DEPRESSION; ANXIETY; PEOPLE; ADULTS; ASSOCIATION; INVENTORY; SYMPTOMS; IMPACT
AB Objective: In the COVID-19 pandemic period, the effect of COVID-19 fear on quality of life was uncertain. This present study examined the effect of fear of COVID-19 on quality of life in patients with epilepsy through anxiety and depression. Methods: This single-center, cross-sectional study was conducted with a total of 319 adult patients with epilepsy. The mean age of the patients was 36.0 (+/- 11.1) years, and 53% were male. The mean duration of diagnosis was 16.0 (+/- 10.6) years, and more than half of the patients (52.4%) were using two and more antiepileptic drugs. Data were collected

online between 15 December 2020 and 5 January 2021. COVID-19 Fear Scale, Beck Depression Inventory, Worry and Anxiety Questionnaire, and Epilepsy Quality of Life Scale-10 were used for data collection. Serial mediation analysis was conducted to determine the effect of COVID-19 fear on quality of life. Results: Although there was a significant correlation between them, the fear of COVID-19 did not directly affect the quality of life. The fear of COVID-19 increased anxiety and depression respectively, the increase in the mediators also decreased the quality of life. Conclusion: The fear of COVID-19 experienced by patients with epilepsy did not have a significant direct effect on the quality of life. However, it was found that it had an indirect effect on the quality of life by increasing mental health problems such as anxiety and depression, the indirect effect was mostly on anxiety.
C1 [Yeni, Kubra] Ondokuz Mayis Univ, Fac Hlth Sci, OMU Kurupelit Kampusu, TR-55200 Samsun, Turkey.
[Tulek, Zeliha] Istanbul Univ Cerrahpasa, Florence Nightingale Fac Nursing, Istanbul, Turkey.
[Ozer, Arif] Hacettepe Univ, Fac Educ, Dept Guidance & Psychol Counseling, Ankara, Turkey.
[Cavusoglu, Aysel; Inan, Gorkem Sirin; Baykan, Betul; Bebek, Nerses] Istanbul Univ, Istanbul Fac Med, Dept Neurol, Istanbul, Turkey.
[Baykan, Betul; Bebek, Nerses] Istanbul Univ, Epilepsy Res Ctr, Istanbul, Turkey.
C3 Ondokuz Mayis University; Istanbul University - Cerrahpasa; Hacettepe University; Istanbul University; Istanbul University
RP Yeni, K (通讯作者), Ondokuz Mayis Univ, Fac Hlth Sci, OMU Kurupelit Kampusu, TR-55200 Samsun, Turkey.
EM akdagyeni@hotmail.com
TC 0
Z9 0
PD MAR
PY 2022
VL 27
IS 1
BP 109
EP 116
DI 10.54029/2022jzi
WC Clinical Neurology
ER

PT J
AU Choudhary, N
Chakravarty, K
Kharbanda, PS
Lal, V
Baishya, J
AF Choudhary, Neetu
Chakravarty, Kamalesh
Kharbanda, Parampreet S.
Lal, Vivek

Baishya, Jitupam
TI Satisfaction and effectiveness of tele-medicine in follow-up of people with epilepsy in a resource-poor setting during COVID-19
SO EPILEPSY & BEHAVIOR
LA English
DT Article
DE Telemedicine; People with epilepsy; epilepsy
ID TELEMEDICINE; TELEHEALTH; OUTCOMES
AB Background: Effectiveness of different tele-medicine strategies varies in different medical conditions. Use of basic tele-medicine strategy like mobile health (m-health) can be an effective option in different med-ical conditions in a resource-poor setting.

Aims: To study effectiveness and satisfaction of tele-medicine among persons with epilepsy (PWE) in a developing nation during COVID-19 pandemic.

Methods: Persons with epilepsy aged 18 years or more who have attended epilepsy clinic at least once physically and were asked for regular follow-up were included. A cross-sectional telephonic survey was conducted to assess effectiveness of tele-medicine over past 1 year. Satisfaction was assessed by tele-medicine satisfaction questionnaire.

Result: 31.9% of PWE have used tele-medicine facility in last 1 year and 58.2% were unaware of the avail-ability of such a facility. Among those who utilized tele-medicine, 95.3% were able to explain their con-cerns satisfactorily during tele-consultation and change in prescription was done in 42.8%. None experienced any new adverse event. Overall, more than 95% were satisfied with tele-consultation and more than 80% wanted to use it again.

Conclusion: Even basic tele-medicine strategies can be a very effective and satisfactory mode of follow-up for PWE in resource-poor settings. Steps should be undertaken to make people aware of the availability of such a facility. (C) 2022 Elsevier Inc. All rights reserved.
C1 [Choudhary, Neetu; Chakravarty, Kamalesh; Kharbanda, Parampreet S.; Lal, Vivek; Baishya, Jitupam] Postgrad Inst Med Educ & Res, Dept Neurol, Chandigarh, India.
C3 Post Graduate Institute of Medical Education & Research (PGIMER), Chandigarh
RP Baishya, J (通讯作者), Postgrad Inst Med Educ & Res, Dept Neurol, Chandigarh, India.
EM jtpm1999asm@gmail.com
TC 0
Z9 0
PD MAR
PY 2022
VL 128
AR 108569
DI 10.1016/j.yebeh.2022.108569
WC Behavioral Sciences; Clinical Neurology; Psychiatry
ER

PT J
AU Gumisiriza, N
Kamoen, O

Boven, A
Dusabimana, A
Nono, D
Musisi, S
Colebunders, R
AF Gumisiriza, Nolbert
Kamoen, Olivia
Boven, Annelies
Dusabimana, Alfred
Nono, Denis
Musisi, Seggane
Colebunders, Robert
TI Impact of the COVID-19 pandemic on persons with epilepsy in Uganda: A descriptive cross-sectional study
SO EPILEPSY & BEHAVIOR
LA English
DT Article
DE Epilepsy; COVID-19; Lockdown; Depression; Anxiety; Uganda
ID PEOPLE; COMORBIDITIES; DEPRESSION; PREVALENCE; PHQ-9
AB Objective: To evaluate the impact of the coronavirus disease 2019 (COVID-19) pandemic on the disease course, lives, and psychosocial wellbeing of persons with epilepsy (PWE) in Uganda. Methods: From April 2021 till May 2021, we carried out a descriptive cross-sectional study at four hospitals located in four regions of Uganda. PWE presenting at the study sites were offered a structured questionnaire in the local language. We used the PHQ-9 questionnaire to screen for depression and the GAD-7 to screen for anxiety. Univariate and multivariable logistic regression was used to investigate factors associated with anxiety and depression. Results: A total of 370 responses were collected. The median age of the respondents was 20.5 years (IQR 15-29), and 51.9% were males. During the lockdown period, the seizure frequency increased in 87 (23.5%) PWE. Various forms of physical and psychological violence were inflicted upon 106 (28.6%) PWE. Fiftyeight (15.7%) screened positive for anxiety and 65 (17.6%) positive for depression. Both increased seizure frequency and experienced violence were associated with experiencing depression and anxiety. Conclusion: The COVID-19 pandemic and lockdown impacted seizure frequency and the psychosocial wellbeing of PWE in Uganda. Increased seizure frequency was associated with higher rates of anxiety and depression. This underlines the importance of continued follow-up of PWE and a low threshold to screen for depression, anxiety, and domestic violence. (c) 2021 Elsevier Inc. All rights reserved.
C1 [Gumisiriza, Nolbert] Kabale Univ, Dept Mental Hlth, Sch Med, Kabale, Uganda.
[Kamoen, Olivia] Heilig Hart Ziekenhuis, Dept Neurol, Lier, Belgium.
[Boven, Annelies; Dusabimana, Alfred; Colebunders, Robert] Univ Antwerp, Global Hlth Inst, Antwerp, Belgium.
[Nono, Denis] Makerere Univ, AfriChild Ctr Study African Child, Kampala, Uganda.
[Musisi, Seggane] Makerere Univ, Dept Psychiat, Coll Hlth Sci, Kampala, Uganda.
C3 University of Antwerp; Makerere University; Makerere University
RP Colebunders, R (通讯作者), Univ Antwerp, Global Hlth Inst, Gouverneur Kinsbergen Ctr, Doornstr 331, B-2610 Antwerp, Belgium.

EM robert.colebunders@uantwerpen.be
TC 0
Z9 0
PD MAR
PY 2022
VL 128
AR 108536
DI 10.1016/j.yebeh.2021.108536
WC Behavioral Sciences; Clinical Neurology; Psychiatry
ER

PT J
AU Sammarra, I
Martino, I
Caligiuri, ME
Giugno, A
Fortunato, F
Labate, A
Gambardella, A
AF Sammarra, Ilaria
Martino, Iolanda
Caligiuri, Maria Eugenia
Giugno, Alessia
Fortunato, Francesco
Labate, Angelo
Gambardella, Antonio
TI The impact of one-year COVID-19 containment measures in patients with mesial temporal lobe epilepsy: A longitudinal survey-based study
SO EPILEPSY & BEHAVIOR
LA English
DT Article
DE SARS-CoV-2; Mesial temporal lobe epilepsy; Longitudinal survey; COVID-19 pandemic impact; Neuropsychology
ID PSYCHOMETRIC PROPERTIES; ABNORMALITIES; SCALE
AB Background: We assessed levels of depression, anxiety, stress, anhedonia, somatization, psychological distress, sleep, and life quality in patients with mesial temporal lobe epilepsy (MTLE) after one year of containment measures started in Italy to stem the COVID-19 pandemic. Methods: We consecutively enrolled 51 patients with MTLE, administering an online survey that compared the year before and after the COVID-19 propagation. We analyzed clinical data (e.g., seizure frequency, life quality) and neuropsychological assessment through Somatic Symptom Scale-8 (SSS-8), Beck Depression Inventory (BDI-2), State-Trait Anxiety Inventory (STAI-Y), Depression, Anxiety and Stress Scale (DASS-21), Pittsburgh Sleep Quality Index (PSQI), Snaith-Hamilton Pleasure Scale (SHAPS), Impact of Event Scale-Revised (IES-R). The BDI-2 and STAI-Y scores were compared to those acquired in the same patients before the COVID-19 outbreak. Results: Comparing our population with MTLE before and after COVID-19 outbreak, we found a significant worsening in life quality ($p = 0.03$), SSS-8 ($p = 0.001$), BDI-2 ($p = 0.032$),

and STAI-Y scores (p < 0.001). After one year of pandemic, 88.2% of patients obtained pathological scores at PSQI, 19.6% at SHAPS, 29.4% at IES-R. Reduction of life quality correlated with anxiety, depression, stress, and somatization. Higher levels of anhedonia correlated with stress, depression, and anxiety. Somatization correlated with depression, anxiety, and sleep quality. Distress levels correlated with anxiety, somatization, and depression. Conclusions: We demonstrated a significant worsening of depression, anxiety, life quality, and somatization in patients with MTLE after one year of COVID-19 beginning. Concomitantly, results suggest that the pandemic had a negative impact on sleep quality, psychological distress, and anhedonia, but not on epilepsy itself. (c) 2022 Elsevier Inc. All rights reserved.
C1 [Sammarra, Ilaria; Martino, Iolanda; Giugno, Alessia; Fortunato, Francesco; Labate, Angelo; Gambardella, Antonio] Magna Graecia Univ Catanzaro, Inst Neurol, Dept Med & Surg Sci, Catanzaro, Italy.
[Caligiuri, Maria Eugenia; Gambardella, Antonio] Magna Graecia Univ Catanzaro, Neurosci Res Ctr, Dept Med & Surg Sci, Catanzaro, Italy.
[Gambardella, Antonio] CNR, Neuroimaging Res Unit, Inst Mol Bioimaging & Physiol, Catanzaro, Italy.
C3 Magna Graecia University of Catanzaro; Magna Graecia University of Catanzaro; Consiglio Nazionale delle Ricerche (CNR); Istituto di Bioimmagini e Fisiologia Molecolare (IBFM-CNR)
RP Labate, A; Gambardella, A (通讯作者)，Magna Graecia Univ Catanzaro, Inst Neurol, I-88100 Catanzaro, Italy.
EM francescofortunato@unicz.it; labate@unicz.it; a.gambardella@unicz.it
TC 0
Z9 0
PD MAR
PY 2022
VL 128
AR 108600
DI 10.1016/j.yebeh.2022.108600
WC Behavioral Sciences; Clinical Neurology; Psychiatry
ER

PT J
AU Noyman, I
Ekstein, D
Fahoum, F
Herskovitz, M
Linder, I
Ben Zeev, B
Eyal, S
AF Noyman, Iris
Ekstein, Dana
Fahoum, Firas
Herskovitz, Moshe
Linder, Ilan
Ben Zeev, Bruria

Eyal, Sara
TI Using nirmatrelvir/ritonavir in patients with epilepsy: An update from the Israeli chapter of the International League Against Epilepsy
SO EPILEPSIA
LA English
DT Editorial Material; Early Access
DE antiepileptic drug; antiseizure medications; COVID-19; CYP3A4; drug-drug interactions
ID RITONAVIR
AB Presented herein are recommendations for use of nirmatrelvir/ritonavir in patients with epilepsy, as issued by the Steering Committee of the Israeli chapter of the International League Against Epilepsy. The recommendations suggest that patients on moderate-to-strong enzyme-inducing antiseizure medications (ASMs) and everolimus should not be treated with nirmatrelvir/ritonavir; rectal diazepam may be used as an alternative to buccal midazolam; doses of ASMs that are cytochrome P450 (CYP3A4) substrates might be adjusted; and patients treated with combinations of nirmatrelvir/ritonavir and ASMs that are CYP3A4 substrates or lamotrigine should be monitored for drug efficacy and adverse drug reactions.
C1 [Noyman, Iris] Soroka Univ Med Ctr, Pediat Neurol Unit, Beer Sheva, Israel.
[Noyman, Iris] Ben Gurion Univ Negev, Fac Hlth Sci, Beer Sheva, Israel.
[Ekstein, Dana] Hadassah Med Org, Agnes Ginges Ctr Human Neurogenet, Dept Neurol, Jerusalem, Israel.
[Ekstein, Dana] Hebrew Univ Jerusalem, Fac Med, Jerusalem, Israel.
[Fahoum, Firas] Tel Aviv Sourasky Med Ctr, Neurol Div, Epilepsy & EEG Unit, Tel Aviv, Israel.
[Fahoum, Firas; Ben Zeev, Bruria] Tel Aviv Univ, Sackler Fac Med, Tel Aviv, Israel.
[Herskovitz, Moshe] Rambam Hlth Care Campus, Dept Neurol, Haifa, Israel.
[Herskovitz, Moshe] Technion Fac Med, Haifa, Israel.
[Linder, Ilan] Barzilai Govt Hosp, Pediat Epilepsy & Neurol Serv, Ashqelon, Israel.
[Ben Zeev, Bruria] Safra Pediat Hosp, Sheba Med Ctr, Pediat Neurol Unit, Tel Hashomer, Israel.
[Eyal, Sara] Hebrew Univ Jerusalem, Fac Med, Sch Pharm, Inst Drug Res, IL-91120 Jerusalem, Israel.
C3 Ben Gurion University; Soroka Medical Center; Ben Gurion University; Hebrew University of Jerusalem; Hadassah University Medical Center; Hebrew University of Jerusalem; Tel Aviv University; Sackler Faculty of Medicine; Tel Aviv Sourasky Medical Center; Shamir Medical Center (Assaf Harofeh); Tel Aviv University; Sackler Faculty of Medicine; Rambam Health Care Campus; Technion Israel Institute of Technology; Rappaport Faculty of Medicine; Ben Gurion University; Barzilai Medical Center; Chaim Sheba Medical Center; Hebrew University of Jerusalem
RP Eyal, S (通讯作者), Hebrew Univ Jerusalem, Fac Med, Sch Pharm, Inst Drug Res, IL-91120 Jerusalem, Israel.
EM sara.eyal@mail.huji.ac.il
TC 0
Z9 0
DI 10.1111/epi.17212

EA MAR 2022
WC Clinical Neurology
ER

PT J
AU Papadopoulou, S
Pavlidou, E
Argyris, G
Flouda, T
Koukoutsidi, P
Krikonis, K
Shah, SD
Chirosca-Vasileiou, D
Boussios, S
AF Papadopoulou, Soultana
Pavlidou, Efterpi
Argyris, Georgios
Flouda, Thaleia
Koukoutsidi, Panagiota
Krikonis, Konstantinos
Shah, Sidrah
Chirosca-Vasileiou, Dana
Boussios, Stergios
TI Epilepsy and Diagnostic Dilemmas: The Role of Language and Speech-Related Seizures
SO JOURNAL OF PERSONALIZED MEDICINE
LA English
DT Review
DE epilepsy; seizure; language-induced epilepsy; stuttering; psychogenic nonepileptic seizures
ID READING EPILEPSY; CHILDREN; SIGN; EEG
AB Although the impact of epilepsy on expressive language is heavily discussed, researched, and scientifically grounded, a limited volume of research points in the opposite direction. What about the causal relationship between disorder-related language activities and epileptic seizures? What are the possible diagnostic dilemmas that experts in the field of speech-language pathology, neurology, and related fields face? How far has research gone in investigating psychogenic nonepileptic seizures, the misdiagnosis of which can be a thorny issue for clinicians and a detrimental factor for the patients' health? In order to address these questions, the study at hand focuses on a common, ever-intensified (by the COVID-19 pandemic) speech disorder-stuttering, and explores the pathophysiological and psychogenic background of the phenomenon. It also looks at the role of stuttering as a contributing factor to the appearance of epileptic seizures, in the hope of drawing attention to the complexity and importance of precise detection of stuttering-induced epilepsy, as a specific subcategory of language-induced epilepsy.
C1 [Papadopoulou, Soultana; Pavlidou, Efterpi] Univ Hosp Ioannina, Dept Speech & Language Therapy, Ioannina 45111, Greece.

[Argyris, Georgios] ENT Private Med Off, Ioannina 45444, Greece.
[Koukoutsidi, Panagiota] Univ Patras, Sch Med, Biomed Engn, Patras 26331, Greece.
[Krikonis, Konstantinos] DatAnalysis, Stat & Res Design Co, Ioannina 45221, Greece.
[Shah, Sidrah] Guys & St Thomas Hosp, Dept Palliat Care, London SE1 9RT, England.
[Chirosca-Vasileiou, Dana] Medway NHS Fdn Trust, Dept Neurol, Windmill Rd, Gillingham ME7 5NY, England.
[Boussios, Stergios] Medway NHS Fdn Trust, Dept Med Oncol, Windmill Rd, Gillingham ME7 5NY, England.
[Boussios, Stergios] Kings Coll London, Fac Life Sci & Med, Sch Canc & Pharmaceut Sci, London SE1 9RT, England.
[Boussios, Stergios] AELIA Org, 9th Km Thessaloniki Thermi, Thessaloniki 57001, Greece.
C3 University Hospital Ioannina; University of Patras; Guy's & St Thomas' NHS Foundation Trust; University of London; King's College London
RP Boussios, S (通讯作者), Medway NHS Fdn Trust, Dept Med Oncol, Windmill Rd, Gillingham ME7 5NY, England.; Boussios, S (通讯作者), Kings Coll London, Fac Life Sci & Med, Sch Canc & Pharmaceut Sci, London SE1 9RT, England.; Boussios, S (通讯作者), AELIA Org, 9th Km Thessaloniki Thermi, Thessaloniki 57001, Greece.
EM soultpap@yahoo.gr; efterpi.pavlidou@gmail.com; argyrisgiwrgos@gmail.com; thaleia.flouda@yahoo.gr; drosfp@gmail.com; krikonis@yahoo.com; sidrah.shah@nhs.net; d.chirosca@nhs.net; stergiosboussios@gmail.com
TC 0
Z9 0
PD APR
PY 2022
VL 12
IS 4
AR 647
DI 10.3390/jpm12040647
WC Health Care Sciences & Services; Medicine, General & Internal
ER

PT J
AU Blank, LJ
AF Blank, Leah J.
TI Socioeconomic disparities in epilepsy care
SO CURRENT OPINION IN NEUROLOGY
LA English
DT Review
DE care; epilepsy; socioeconomic disparity
ID PEDIATRIC EPILEPSY; UNDERUTILIZATION; SURGERY; ACCESS; ADULTS; TRENDS
AB Purpose of review Epilepsy is a common, chronic neurologic disease with continued disparities in care. The COVID-19 pandemic and recent social movements have drawn greater attention to social determinants of health and our progress (or lack thereof) toward delivering more equitable care. Recent findings Recent studies continue to document racial and economic disparities in diagnosis, treatment, and overall care of epilepsy and associated conditions. Notably, an increasing number of studies are

attempting to design healthcare pathways and other interventions to improve access and equity in epilepsy care. The present literature highlights the importance of identifying and addressing the particular needs of vulnerable persons with epilepsy. Practitioners and researchers should continue to develop interventions aimed at improving care for all patients and, crucially, measure the impact of their changes to ensure that any interventions are truly advancing health equity.
C1 [Blank, Leah J.] Dept Populat Hlth Sci & Policy, Dept Neurol, Div Hlth Outcomes & Knowledge Translat, New York, NY USA.
RP Blank, LJ (通讯作者)，Icahn Sch Med Mt Sinai, One Gustave L Levy Pl,Annenberg 14-10,Box 1137, New York, NY 10029 USA.
EM leah.blank@mssm.edu
TC 0
Z9 0
PD APR
PY 2022
VL 35
IS 2
BP 169
EP 174
DI 10.1097/WCO.0000000000001031
WC Clinical Neurology; Neurosciences
ER

PT J
AU Anuszkiewicz, K
Stogowski, P
Zawadzka, M
Waszak, P
Sokolewicz, E
Dulak, NA
Dzwilewski, K
Jazdzewska, K
Karbowiak, K
Karlinska, D
Marczak, A
Niebrzydowska, A
Niebrzydowski, B
Pasierbska, E
Sadowska, A
Szczesna, M
Szczesny, PS
Szerszenowicz, A
Sztramski, K
Radziwon, J
Tkaczuk, M
Ziolkowska, K
Mazurkiewicz-Beldzinska, M

AF Anuszkiewicz, Karolina
Stogowski, Piotr
Zawadzka, Marta
Waszak, Przemyslaw
Sokolewicz, Ewa
Dulak, Natalia Aleksandra
Dzwilewski, Kamil
Jazdzewska, Karolina
Karbowiak, Kamila
Karlinska, Daria
Marczak, Anna
Niebrzydowska, Anna
Niebrzydowski, Bartosz
Pasierbska, Ewa
Sadowska, Agnieszka
Szczesna, Malgorzata
Szczesny, Piotr Stanislaw
Szerszenowicz, Anna
Sztramski, Kamil
Radziwon, Jakub
Tkaczuk, Magdalena
Ziolkowska, Kinga
Mazurkiewicz-Beldzinska, Maria
TI COVID-19 pandemic influence on epilepsy course in pediatric patients
SO EPILEPSY & BEHAVIOR
LA English
DT Article
DE Epilepsy; Change; Children; COVID-19; Telemedicine; Satisfaction
ID TELEMEDICINE; CARE
AB Introduction: In 2020, Coronavirus Disease 2019 (COVID-19) was declared as a global pandemic. Self reported stress, anxiety, and insomnia, which are believed to be common triggers for epilepsy, are more likely to occur. We aimed to establish the influence of COVID-19 pandemic itself on changes in the daily life routine related to pandemic on epilepsy course in pediatric patients. The unique form of clinical care which is telemedicine was also taken into consideration. We wanted to evaluate patients' satisfaction with telemedicine and if changing stationary visits into telemedicine influenced epilepsy course in our patients. Methods: Patients, who attended developmental neurology outpatient clinic in the period March- December 2020 were collected. As patients were minors, legal guardians were asked to fill out the questionnaire. Patients were divided according to the outcome into three groups: those with a worsened, stable, or improved course of epilepsy during the pandemic. Appropriate statistical tests for two-group and multi-group comparisons have been implemented. Post hoc p values were also calculated. Results: Four hundred and two questionnaires were collected. Most of the patients had a stable course of epilepsy during the pandemic; in 13% of participants an improvement has been observed, worsening of the disease was seen in 16% of patients. Age, sex, type of epilepsy, number of seizure incidents before pandemic, and duration of the disease had no statistically significant connection with

changes in the course of the disease. Behavioral changes and altered sleep patterns were found to be more common in the worsened group. Fifty-eight percent of patients were satisfied with telemedicine. Poorer satisfaction was connected with less frequent visits, cancellation of scheduled appointments, and lack of help in case of need in an emergency situation. Conclusion: Epilepsy course in pediatric patients seems to be stable during COVID-19 pandemic. Sleep disturbances and changes in a child's behavior may be related to increase in seizure frequency. Telemedicine is an effective tool for supervising children with epilepsy. Patients should be informed about possible ways of getting help in urgent cases. (c) 2022 Elsevier Inc. All rights reserved.
C1 [Anuszkiewicz, Karolina; Stogowski, Piotr; Zawadzka, Marta; Sokolewicz, Ewa; Dulak, Natalia Aleksandra; Dzwilewski, Kamil; Jazdzewska, Karolina; Karbowiak, Kamila; Karlinska, Daria; Marczak, Anna; Niebrzydowska, Anna; Niebrzydowski, Bartosz; Pasierbska, Ewa; Sadowska, Agnieszka; Szczesna, Malgorzata; Szczesny, Piotr Stanislaw; Szerszenowicz, Anna; Sztramski, Kamil; Radziwon, Jakub; Tkaczuk, Magdalena; Ziolkowska, Kinga; Mazurkiewicz-Beldzinska, Maria] Med Univ Gdansk, Dept Dev Neurol, Debinki 7 St, PL-80952 Gdansk, Poland.

[Waszak, Przemyslaw] Dept Dev Psychiat Psychot & Geriatr Disorders, Dept Hyg & Epidemiol, Debinki 7 St, PL-80952 Gdansk, Poland.

C3 Medical University Gdansk

RP Anuszkiewicz, K (通讯作者), Med Univ Gdansk, Dept Dev Neurol, Debinki 7 St, PL-80952 Gdansk, Poland.

EM karolina.anuszkiewicz@gumed.edu.pl
TC 0
Z9 0
PD APR
PY 2022
VL 129
AR 108581
DI 10.1016/j.yebeh.2022.108581
WC Behavioral Sciences; Clinical Neurology; Psychiatry
ER

PT J
AU Cokley, JA
Gidal, BE
Keller, JA
Vossler, DG
AF Cokley, Jon A.
Gidal, Barry E.
Keller, Joy A.
Vossler, David G.
TI Paxlovid (TM) Information From US Food & Drug Administration (FDA) and Guidance for AES Members
SO EPILEPSY CURRENTS
LA English
DT Article; Early Access
DE Paxlovid (TM); ritonavir; antiepileptic drug; drug interactions; adverse effect;

epilepsy; seizure; COVID-19

AB This American Epilepsy Society (AES) official statement provides information and preliminary guidance to Society members related to the U.S. Food & Drug Administration (FDA) December 22, 2021 Emergency Use Authorization for Paxlovid (TM) for the oral treatment of mild to moderate COVID-19 in adults and children (>= 12 years and weighing >= 40 kg). Paxlovid is likely to be widely prescribed, and important considerations for patients on antiseizure medications (ASMs) include key contraindications and potential toxicity or dose adjustments while taking Paxlovid. This statement highlights concerns and provides information about their pharmacologic basis. Of particular concern, concomitant use of Paxlovid with the ASMs carbamazepine, phenobarbital, phenytoin, and primidone is contraindicated, because they are strong inducers of the CYP3A4 isozyme that metabolizes Paxlovid and thereby could cause loss of virologic response and development of resistance. Alternate oral or intravenous COVID-19 treatments should be considered. A second concern is that Paxlovid may increase the plasma concentrations of many ASMs, because it inhibits the CYP3A4 isozyme. ASMs that are metabolized, at least in part, by CYP3A4 include cannabidiol, carbamazepine, clobazam, clonazepam, diazepam, ethosuximide, everolimus, felbamate, lacosamide, midazolam, oxcarbazepine, perampanel, stiripentol, tiagabine, and zonisamide. Patients receiving these medications may warrant closer monitoring while being treated with Paxlovid.

C1 [Cokley, Jon A.] Texas Childrens Hosp, Dept Pharm, Houston, TX 77030 USA.
[Gidal, Barry E.] Univ Wisconsin, Sch Pharm, 425 N Charter St, Madison, WI 53706 USA.
[Keller, Joy A.] Amer Epilepsy Soc, Dept Clin Act, 35 S LaSalle St, Chicago, IL 60603 USA.
[Vossler, David G.] Univ Washington Syst, Dept Neurol, Renton, WA USA.
C3 Baylor College of Medicine; University of Wisconsin System; University of Wisconsin Madison
RP Keller, JA (通讯作者), Amer Epilepsy Soc, Dept Clin Act, 35 S LaSalle St, Chicago, IL 60603 USA.
EM jkeller@aesnet.org

TC 0
Z9 0
DI 10.1177/15357597221088415
EA APR 2022
WC Clinical Neurology
ER

PT J
AU Yavuz, ENV
Altindag, E
Tuzun, E
Baykan, B
AF Yavuz, Ebru Nur Vanli
Altindag, Ebru
Tuzun, Erdem
Baykan, Betul

TI Do the neurologists recognize autoimmune epilepsy well enough? What is the effect of the pandemic on this matter?
SO NEUROLOGICAL SCIENCES
LA English
DT Article; Early Access
DE Autoimmune; epilepsy; Anti-neuronal antibodies; Neurologist; Awareness; COVID-19
ID NMDA RECEPTOR ENCEPHALITIS; TEMPORAL-LOBE EPILEPSY; NEURONAL AUTOANTIBODIES; DISORDERS; SEIZURES
AB Introduction The concept of "autoimmune epilepsy" (AE) has been emphasized more frequently through the recent increase in recognition of various autoantibodies specific to neuronal proteins.

Aims To evaluate the attitudes of neurologists in regard to AE, to review the differential diagnosis, treatment options, and to reveal the effect of COVID-19 on this matter.

Methods A detailed questionnaire prepared for AE was sent to neurologists via social media and WhatsApp after the approval of the Ethics Committee. The responses of 245 respondents working in different settings were analyzed, and the group with 15 years or less experience in neurology was statistically compared to the group with more than 15 years of experience.

Results Awareness and knowledge levels on AE seemed high in all groups, while 11% had never thought about AE during the differential diagnosis in real life. Before starting treatment, 20% thought that the autoantibody result should definitely support it, and 77.6% reported that they did not recognize AE well. Participants stated that satisfactory guidelines for diagnosis and treatment (88.2%) and widespread laboratory support (83.7%) were lacking. Neurologists with less experience and those working outside of training hospitals get more often consultation from an experienced clinician while diagnosing and conduct more detailed investigations at the diagnosis stage ($p = 0.0025$, $p = 0.0001$).

Conclusion This first survey study conducted in a large group of neurologists on the attitudes for the concept of AE suggested that postgraduate education, and diagnostic and treatment guidelines should be organized and antibody screening tests need to be better disseminated.
C1 [Yavuz, Ebru Nur Vanli] Demiroglu Bilim Univ, Fac Med, Dept Neurol, Buyukdere Caddesi 120, TR-34394 Sisli, Turkey.

[Altindag, Ebru] Istanbul Florence Nightingale Hosp, Dept Neurol, Istanbul, Turkey.

[Tuzun, Erdem] Istanbul Univ, Aziz Sancar Res Inst Expt Med, Dept Neurosci, Istanbul, Turkey.

[Baykan, Betul] Istanbul Univ, Istanbul Fac Med, Dept Neurol, Istanbul, Turkey.
C3 Grup Florence Nigtingale Hospital Turkey; Istanbul University; Istanbul University
RP Yavuz, ENV (通讯作者), Demiroglu Bilim Univ, Fac Med, Dept Neurol, Buyukdere Caddesi 120, TR-34394 Sisli, Turkey.
EM ebruvanli@gmail.com
TC 0
Z9 0
DI 10.1007/s10072-022-06044-5

EA APR 2022
WC Clinical Neurology; Neurosciences
ER

PT J
AU Sirikarn, P
Tiamkao, S
Tiamkao, S
AF Sirikarn, Prapassara
Tiamkao, Siriporn
Tiamkao, Somsak
TI The effects of COVID-19 measures on the hospitalization of patients with epilepsy and status epilepticus in Thailand: An interrupted time series analysis
SO EPILEPSIA OPEN
LA English
DT Article; Early Access
DE COVID-19; hospital admission; noncommunicable diseases; public measures; seizure
ID MENTAL-HEALTH
AB Objective To investigate the effect of Coronavirus disease 2019 (COVID-19) measures on the hospitalization of patients with epilepsy and status epilepticus (SE). Methods This interrupted time series design included data from the Thai Universal Coverage Scheme electronic database between January 2017 and September 2020. The monthly hospitalization rate of epilepsy and SE was calculated by the number of hospitalizations divided by the midyear population. Segmented regression fitted by ordinary least squares (OLS) was used to detect the immediate and overtime effects of COVID-19 measures on the hospitalization rate. Results During January 2017 and September 2020, the numbers of epilepsy and SE patients admitted to the hospital were 129 402 and 15 547 episodes, respectively. The monthly trend of the hospitalization rate in epilepsy decreased immediately after the COVID-19 measure (0.739 per 100 000 population [95% CI: 0.219 to 1.260]). In particular, the number of children declined to 1.178 per 100 000 population, and the number of elderly individuals dropped to 0.467 per 100 000 population, while there was a nonstatistically significant change in SE. Significance COVID-19 measures reduced the hospital rate in epilepsy, particularly in children and adults. However, there was no change in SE patients.
C1 [Sirikarn, Prapassara] Khon Kaen Univ, Fac Publ Hlth, Dept Epidemiol & Biostat, Khon Kaen, Thailand.
[Sirikarn, Prapassara; Tiamkao, Siriporn; Tiamkao, Somsak] Khon Kaen Univ, Integrated Epilepsy Res Grp, Khon Kaen, Thailand.
[Tiamkao, Siriporn] Khon Kaen Univ, Fac Med, Dept Pharmacol, Khon Kaen, Thailand.
[Tiamkao, Somsak] Khon Kaen Univ, Fac Med, Dept Med, Div Neurol, 123 Mittraphap Rd, Khon Kaen 40002, Thailand.
C3 Khon Kaen University; Khon Kaen University; Khon Kaen University; Khon Kaen University
RP Tiamkao, S (通讯作者), Khon Kaen Univ, Fac Med, Dept Med, Div Neurol, 123 Mittraphap Rd, Khon Kaen 40002, Thailand.
EM somtia@kku.ac.th

TC 0
Z9 0
DI 10.1002/epi4.12600
EA APR 2022
WC Clinical Neurology; Neurosciences
ER

EF
